
JOURNAL INFORMATION
==============================
NLM Title Abbreviation: J Physiol Sci
Journal ID: J Physiol Sci
NLM Journal ID: 101262417

The Journal of Physiological Sciences

ISSN: 1880-6546
EISSN: 1880-6562

ARTICLE INFORMATION
==============================
PMCID: PMC8373421
DOI: 10.1186/s12576-021-00809-x
Article ID: 809
Article version: 1
Subjects: Meeting Abstracts

Proceedings of the Joint Meeting of the 126th Annual Meeting of The Japanese Association of Anatomists and the 98th Annual Meeting of The Physiological Society of Japan

Electronic publication date: 2021 Aug 19
Print publication date: 2021
Volume: 71
Issue: Suppl 1
Electronic Location ID: 24
Copyright: © The Author(s) 2021
License: Open AccessThis article is licensed under a Creative Commons Attribution 4.0 International License, which permits use, sharing, adaptation, distribution and reproduction in any medium or format, as long as you give appropriate credit to the original author(s) and the source, provide a link to the Creative Commons licence, and indicate if changes were made. The images or other third party material in this article are included in the article's Creative Commons licence, unless indicated otherwise in a credit line to the material. If material is not included in the article's Creative Commons licence and your intended use is not permitted by statutory regulation or exceeds the permitted use, you will need to obtain permission directly from the copyright holder. To view a copy of this licence, visit http://creativecommons.org/licenses/by/4.0/.
License URL: https://creativecommons.org/licenses/by/4.0/

issue-copyright-statement: © The Author(s) 2021

==============================
### Special Lectures

### Special Lecture 1
### (S. Hagiwara Memorial Lecture)

(March 28, Sun. 11：10～12：10, Room1)

### SL1

### Neural circuit mechanism of functional recovery after brain and spinal cord injury

Tadashi Isa (*Department of Neuroscience, Graduate School of Medicine, Kyoto University Institute for the Advanced Study of Human Biology [WPI-ASHBi], Kyoto University*)

The central nervous system is composed of multi-layered sensory-motor loops. Damage of a particular system could be compensated by residual neural systems. Such mechanisms may underlie the functional recovery after the brain and spinal cord injury through neurorehabilitative training. Here, ability of the recovered subject would be constrained by the neural systems which take over the damaged system. However, the mechanisms are still largely unexplored. In this lecture, I will summarize our studies over the last two decades on the neural circuit mechanism underlying the recovery of dexterous hand movements after the spinal cord injury and eye movements/cognitive functions after damage to the primary visual cortex in macaque monkey model, which share many properties such as neural structures and skeletomotor systems in common with humans. As a general principle, the latent circuits which are not in full operation in the intact state appear to be uncovered and take over the functions of the impaired system. I will argue on the possible mechanism underlying such compensatory mechanisms.

### Special Lecture 2

(March 29, Mon. 11：10～12：10, Room1)

### SL2

### Working Career for Researchers in This Age of Longevity

Mariko Bando (*Chancellor, Showa Women´s University*)

### Special Lecture 3

(March 29, Mon. 11：10～12：10, Room1)

### SL3

### The formation of the Japanese population revealed by ancient genomes.

Ken-ichi Shinoda (*Senior Director in Research, National Museum of Nature and Science*)

The origin of modern Japanese has been a subject of debate among human geneticists, archaeologists, and anthropologists. Since the 1990s, DNA retrieved from ancient human remains has offered an approach that complements morphometric analysis and contributed modern genetic data to understand the relationships among past populations. In earlier studies, mitochondrial DNA was analyzed owing to technical limitations. However, next-generation sequencing technology has enabled reconstruction of the nuclear genome of ancient human individuals. Genome-wide analyses of single nucleotide polymorphisms provide a powerful tool for estimating population ancestry.

In this presentation, we report the formation of the Japanese population using ancient genome data. Our studies suggest that the DNA of modern Japanese consists of both the Jomon people and the immigrant Yayoi people. It has also become clear that most of the DNA of the mainland Japanese population is inherited from the Yayoi people. Ancient genome analysis in Japan has had a great impact not only on the fields of anthropology and archaeology, but also on all disciplines that consider both Japanese and Japanese cultures.

### Special Lecture 4
### (S. Tahara Memorial Lecture)

(March 29, Mon. 13：10～14：10, Room1)

### SL4

### Studies on the mechanisms underlying skeletal muscle hypertrophy and atrophy and their application to resistance exercise regimens.

Naokata Ishii (*Laboratory of Health Dynamics Studies, The University of Tokyo*)

Skeletal muscles change their size and physiological properties according to the mechanical and/or nutritional environment. Resistance training-induced hypertrophy with strength gain is a typical example. To obtain an insight into the mechanisms underlying the adaptation of skeletal muscle to resistance training, we have studied acute and chronic changes occurring in muscle fibers after exercise stimulus, using our original exercise model with rodents. Particular attention has been focused on the relations between exercise parameters and changes in mTOR (mechanistic target of rapamycin) signal transduction pathway, protein synthesis/degradation and ribosome content. As one of the findings, we have reported that mechanical impulse (time under force) is more important than force amplitude to activate the mTOR pathway and protein synthesis. Also, we found that ribosome biogenesis is activated at the early stage of training period. These findings provide useful information for determining variables such as intensity, volume, movement speed, and rest period between sessions in human exercise regimens. For example, the significance of force-generating time gives a theoretical basis of "low-intensity slow movement training" which has been expected to be a potent countermeasure against aging-related muscle atrophy (sarcopenia) and lifestyle-related diseases such as diabetes.

### Special Lecture 5

(March 30, Tue. 11：10〜12：10, Room1)

### SL5

### Medical procedure assistance by artificial intelligence toward future medicine

Kensaku Mori (*Graduate School of Informatics, Nagoya University*)

In this talk, we will discuss future medicine using artificial intelligence (AI) technology, especially pattern recognition techniques using machine learning. AI techniques are now widely used in various scenes in our life. Smartphone camera now recognizes our faces to make focusing accurate and adds some effects in pictures based on AI techniques. Face recognition-based building entry systems are now implemented in many buildings and even at the passport control at airports. Medicine is also one of big applications of AI techniques. AI can recognize medical images including endoscopic images, CT images, MR images, US images and microscopic images. AI-assisted diagnostic or surgical assistance is achieved and utilized in the clinical field. For example, our research group has recently developed AI endoscopy system for colonic polyp examination. This system automatically detects colonic polyp frames form colonoscopy video frames and gives some cautions to physicians. If a physician finds colonic polyp and take super-magnified endoscopic images of colonic polyps' surface, the system shows pathological-type classification results. These assistances are performed in real-time and assist colonoscopists. Thus, AI has now started to change the scene of the clinical field. Medical staff will collaborate with AI-guided systems in very near future. This talk will discuss future medicine from the viewpoint of symbiosis of medical staff, patients and AI.

### Special Lecture 6

(March 30, Tue. 13：10〜14：10, Room1)

### SL6

### In the Beginning was the Synapses: Manipulation of Synapses to Understand and Treat Brain Dysfunctions.

Michisuke Yuzaki (*Dept. Physiology, Keio University School of Medicine*)

The human brain contains ~$10^{14}$ synapses within a vast network of neurons. Many psychiatric and neurological disorders, such as schizophrenia and Alzheimer's diseases, are likely caused by synaptic dysfunctions. Thus, it is crucial to clarify the mechanisms by which synaptic connections are established, maintained and modified. Changes in neuronal activities lead to functional synaptic plasticity, long-term potentiation (LTP) and depression (LTD). However, it is not completely clear how LTP/LTD are causally related to specific neuronal circuit functions associated with behavioral changes. Synapses also undergo structural changes, which likely support longer-term connectivity modifications. Although many synaptic organizers, such as neurexin and neuroligin, have been identified, it remains largely unclear how they support synaptic functions *in vivo*. To better understand mechanisms underlying functional and structural changes at synapses *in vivo*, we have been developing optogenetic tools that can modulate LTP/LTD at specific synapses, and synthetic synaptic organizers that can induce synapse formation in vivo. As Dr. Feynman said, what we cannot create, we do not understand nor treat.

The Journal of Physiological Sciences (2021) 17 (Suppl. 1): 24
https://doi.org/10.1186/s12576-021-00809-x
Published: 19 August 2021

### Joint Session

### Joint Session1

### The regulation and functions of extracellular vesicles

(March 28, Sun. 9：00～11：00, Room1)

### JS-1
**Development of immuno-regulatory methods using designer exosomes**

Rikinari Hanayama[1] ([1]*WPI-NanoLSI, Kanazawa Univ.*)

Exosomes are small extracellular vesicles secreted by various cells and are attracting attention as a new mediator for intercellular communication. In particular, it has been shown that exosomes are involved in various biological phenomena such as the regulation of cancer progression and immune responses, and therefore, drug discovery targeting or using exosomes is greatly anticipated. In recent years, it has become possible to express a target protein on exosomes produced by cultured cells by gene transfer, and by applying this technology, we are developing designer exosomes to control the immune system. By using our designer exosome with enhanced immunoregulatory function, we can expect to achieve superior effects that cannot be seen with the administration of current immunoregulatory factors. Therefore, we are aiming to develop innovative immunoregulation methods for the treatment of various diseases by further improving the designer exosomes, which have been impossible to achieve with conventional technologies. (COI:No)

### JS-2
**Exosome as a novel diagnostic and therapeutic target for cancer**

Takahiro Ochiya[1] ([1]*Dept Mol Celll Med, Tokyo Medical Univ, Tokyo, Japan*)

In the past several years, the importance of microRNA (miRNA) in cancer cells has been recognized. Dysregulation of miRNAs leads to the cancer development, meaning that expression profile of miRNAs can be used as cancer biomarker. Currently, the contribution of extracellular vesicles (EVs) and their miRNAs to cancer development is widely documented, and they hold promise for use in new methods for diagnosing cancer and monitoring tumorigenesis. Therapeutic targeting of molecules involved in EV production and secretion from cancer cells may prevent or delay cancer recurrence. Following therapeutic applications are possible: 1) inhibition of cancer cell EV production, 2) interference of EV uptake by recipient cells, and 3) elimination of circulating cancer cell?derived EVs. These therapeutic strategies will prevent the delivery of EVs from cancer cells to microenvironmental cells, leading to the development of a novel anticancer drugs. Our current progress on exploring cancer-specific EVs secretion pathways by using several library-screening methods and targeting inhibition of these molecules in animal preclinical studies will be discussed. (COI:Properly Declared)

### JS-3
**Signal peptides as a possible and novel biomarker for extracellular vesicles**

Makoto Sawada[1] ([1]*Brain Function, RIEM, Nagoya Univ*)

Extracellular vesicles (EVs) are attracting attention as structures that contain functional molecules such as microRNAs and are responsible for information transmission between cells, however, how they act on recipient cells remains unclear. We found that signal peptides (SPs) existed in EVs by our uniquely developed hot melt-mass spectrometry. We also demonstrated that SPs can migrate in EVs in the supernatant of cells that inducibly produce the SPs. SPs play a key role in targeting and membrane insertion of secretory and membrane proteins. Since such hydrophobic short peptides are difficult to analyze by ordinary methods because of their instability and stickiness, revelation of biological functions other than the above mentioned has not much progressed, and it has long been a puzzle that SPs have the high degree of sequence and length variation. However, recently the basis for an amazing complexity and versastility of SPs' function has been revealed. Furthemore, each SP is specific to a protein, much like an ID tag for each protein, therefore, profiling of SPs in EVs can contribute to presumption of the biological functions and origin of EVs as a possible new biomarker. (COI:Properly Declared)

### JS-4
**Musculoskeletal system and extracellular vesicles**

Hiroshi Kaji[1] ([1]*Physiol Regener Med·Kindai Univ Facult Med*)

Roles of extracellular vesicles (EVs) in the musculoskeletal system have been clarified at the basic science level. Previous studies revealed that EVs produced by mesenchymal stem cells enhances bone/cartilage repair and regeneration. Moreover, osteoblast- or osteoclast-derived EVs regulate bone remodeling in mice. On the other hand, the interactions between skeletal muscle and bone have been recently noted. We reported that EVs secreted from mouse muscle cells suppress osteoclast formation, which might be involved in the muscle/bone relationships physiologically. Although EVs contain various proteins or microRNAs, our data suggested that some microRNAs might be related to EV effects on osteoclast formation. In addition, our preliminary study suggested that mechanical stress modulates EV effects on osteoclast formation, and muscle derived EVs might have the activity on bone repair/regeneration after bone injury in mice. The clarification of physiological and pathophysiological roles of muscle- or bone-derived EVs may lead to the development of novel treatment tool for various musculoskeletal disease. (COI:No)

### JS-5
**Primary cilia-mediated extracellular vesicles**

Koji Ikegami[1,2] ([1]*Dept Anat & Dev Biol, Grad Sch Biomed & Health Sci, Hiroshima Univ*, [2]*PRESTO, JST*)

Extracellular particles including extracellular vesicles (EVs) are highly heterogeneous in size, composition, contents, origin, and mechanisms of release. Thus, heterogeneous extracellular particles have different names; exosome, microvesicle, ectosome, apoptotic body, exomere, etc. The presenter and colleagues reported that the tip of primary cilia was excised and released into extracellular milieu as an extracellular particle (Phua *et al.* Cell 168: 264-279, 2017). Further analyses identified primary cilia-derived extracellular particles as extracellular vesicles and clarified characteristics of the primary cilia-derived EVs. Biological effects of primary cilia-derived EVs were also investigated *in vitro*. The presenter also found that primary cilia were involved in the release of a subset of extracellular vesicles. Characteristics of the primary cilia-involved extracellular vesicles were investigated in detail. Furthermore, behaviors of the primary cilia-involved EVs were analyzed with time-lapse imaging. In this talk, EVs and their release mediated by primary cilia are presented and discussed. (COI:No)

### Meeting Symposia

### Meeting Symposium1

### Mechanics of cells and tissues

(March 28, Sun. 9：00～11：00, Room2)

### MS1-1

**Mechanical forces acting on renal cells and their sensing mechanisms**

Miki Nagase[1] ([1]*Dept. Anatomy, Sch. Med., Kyorin Univ.*)

Kidney cells are constantly exposed to various mechanical forces caused by blood flow, urinary flow, and pressure. These cells sense the changes of forces, and maintain body fluid homeostasis by flexibly adjusting urine composition. For example, the juxtaglomerular apparatus senses changes in extracellular fluid volume and regulates glomerular filtration rate by modulating afferent arteriolar tonus and renin secretion from granular cells. Mechanobiology is also involved in the pathogenesis of acute kidney injury, chronic kidney disease, and hereditary kidney disease. Podocytes, located in the outermost aspect of the glomerular capillary loop, have abundant cellular processes and actin cytoskeleton. Podocytes are exposed to tensile force due to circumferential wall stress, and fluid shear stress in the filtration slit and in the Bowman's space. Glomerular hyperfiltration and hypertension contribute to podocyte injury and glomerulosclerosis in diabetic nephropathy and hypertensive nephrosclerosis. In this talk, I will review the mechanobiology of the kidney, focusing on the forces acting on renal cells, candidate mechanosensors, and downstream signal cascades. (COI:No)

### MS1-2

**High temperature region heats up TRPV2-mechanosensor function and axonal outgrowth**

Koji Shibasaki[1] ([1]*Lab Neurochem, University of Nagasaki Grad Sch Human Sci*)

We previously reported that TRPV2 is a mechanosensor channel which contributes to axonal outgrowth in membrane stretch dependent manner (J. Neurosci. 2010, JPS 2016, FASEB J. 2017), although TRPV2 was originally cloned as a noxious heat sensor (>52°C). These results indicate that TRPV2 is an important component for the responses against the stretch. In this study, we examined the intracellular temperature distribution during axonal outgrowth by a temperature-imaging method. We found that specific high temperature regions (2~4°C higher) were occasionally localized in the growth cones. Surprisingly, physiological temperature (37°C) was insufficient to sensitize the TRPV2 activation. Unexpectedly, over 39°C condition dramatically accelerates the TRPV2 sensitivity for mechanical stimuli. These results suggest that the hot regions in growth cones contribute to accelerate axonal outgrowth through the TRPV2 sensitization. TRPV2 function is necessary to form long peripheral axons in embryonic stages. (COI:No)

### MS1-3

**Mechanically activated ion channel PIEZO1 is required for lymphatic valve formation**

Keiko Nonomura[1,2], Toshihiko Fujimori[1,2], Ardem Patapoutian[3] ([1]*Div. Embryology, NIBB,* [2]*SOKENDAI,* [3]*TSRI*)

Lymphatic vessels collect extracellular fluid from the interstitial space and are equipped with many valves ensuring unidirectional flow of lymph. In 2015, loss of function mutations of *PIEZO1 w*ere found among patients of familial lymphedema. PIEZO1 is a cation channel that is activated by mechanical forces applied on cell membrane. In order to elucidate how PIEZO1 is involved in the lymphatic system, we have analyzed mouse lines lacking PIEZO1 in endothelial cells, the main cell type of the lymphatic vessels. Those mice died postnatally and exhibited pleural effusion. In addition, the number of lymphatic valves was dramatically reduced. Among steps of valve formation, the protrusion process, in which cells involved in valve formation migrate toward lumen was impaired in Piezo1 cKO mice. This process is associated with actin polymerization and remodeling of cell-cell junctions. We confirmed that activation of PIEZO1 can induce active remodeling of actomyosin and VE-cadherin$^+$ cell-cell adhesion by using cultured lymphatic endothelial cells and PIEZO1 agonist Yoda1. Our analysis show that mechanosensor protein PIEZO1 is a key regulator of lymphatic valve formation. (COI:No)

### MS1-4

**ERK-mediated mechanochemical feedbacks in multicellular tissues and epithelial tissue morphogenesis**

Tsuyoshi Hirashima[1] ([1]*The Hakubi Center, Kyoto University/JST PRESTO*)

How does a group of cells form complex patterns and architectures in a self-organized manner? To address this question, I have studied biophysical aspects of both the mechanical and biochemical regulations underlying multicellular tissue morphogenesis, using murine embryonic organs, live imaging, and mathematical modeling. In the talk, I will focus on an interplay between active cellular forces and the mechano-sensitive MAP kinase ERK signaling, and provide several related topics including collective cell migration and branching morphogenesis. First, we will show a biophysical origin of collective cell migration and ERK activity waves in epithelial cells, and then discuss a close link to the murine cochlear duct development. Next, I will introduce a curvature-driven mechano-chemical feedback control exemplified in lung branching morphogenesis. Finally, I will talk a bit about the future extension, which hopefully enables us to integrate mechano-chemical feedbacks, multicellular pattern formation, and physiological function. (COI:No)

### MS1-5

**Intrauterine mechanical environment produced by smooth muscle contractions for early mouse morphogenesis**

Isao Matsuo[1], Chiharu Kimura-Yoshida[1], Yoko Ueda[1] ([1]*Osaka Women's and Children's Hospital* )

Mammalian embryogenesis proceeds *in utero* with the support of maternal tissues. Since the development of post-implanted embryos in *ex utero* culture is challenging, intrauterine mechanical environment is supposed to be involved in embryogenesis. Notably, mouse embryos morphologically change from the spherical blastocysts to the elongated egg-cylinder shape of post-implanted embryos, which is closely linked to primary axis polarization. However, how mechanical environment can contribute to such shape change remains unaddressed. Here, we found that intrauterine pressures were produced by uterine smooth muscle contractions, showing the highest periodic peaks just after implantation and were necessary for egg-cylinder elongation and axis polarization of embryos. In addition, Reichert's membrane, a basement membrane that wraps around the embryo, played a crucial role as a shock absorber to protect embryos from the excess intrauterine pressures. Mechanistically, the pressures were buffered by the sealed space created with Reichert's membrane. Thus, we propose the buffer space generated by Reichert's membrane cushions the intrauterine pressures for early mouse morphogenesis. (COI:No)

### Meeting Symposium2

### Possibility of reseaches in autonomic nervous system

(March 28, Sun. 9：00～11：00, Room4)

### MS2-1

### Arterial pressure control via the vestibular system

Chikara Abe[1] ([1]*Department of Physiology, Gifu University Graduate School of Medicine*)

Daily activity-induced changing of the gravitational vector is one of the major disturbances that affects cardiovascular system. A postural change from a recumbent to an upright position reduces arterial pressure (AP). This reduction in AP is buffered by the arterial baroreflex, which is an important negative feedback system. On the other hand, postural changes stimulate the peripheral vestibular organs. Stimulation of the peripheral vestibular organs by head movements or changes in gravitational forces is known to induce sympathoexcitation (vestibulo-sympathetic reflex). We have reported that the combination of both the baroreflex and the vestibulo-sympathetic reflex is important for maintaining AP during postural change. Daily vestibular stimulation such as exercise is important for maintaining the vestibular function. Elderly people and astronauts are known to show a hypofunction of the vestibular system because of reduction in vestibular inputs (less daily activity in elderly people and no gravity in astronauts). This might participate in the increase in their risk of orthostatic hypotension. This possibility will be discussed in this symposium. (COI:No)

### MS2-2

### Pathways of hypoxia-induced respiratory and cortical arousal

Shigefumi Yokota[1], Noriyuki Hama[2], Kotaro Takeda[3], Yasumasa Okada[4] ([1]*Dept Anat &Neurosci, Shimane Univ Sch Med*, [2]*Dept Phsiol, Shimane Univ Sch Med*, [3]*Fuc Rehab, Fujita Hlth Univ*, [4]*Clin Res Ctr, Murayama Med Ctr*)

Decrease of arterial oxygen pressure facilitates breathing as well as induces arousal from sleep. Under hypoxia, the information on lowered arterial oxygen pressure inputs to and activates glutamatergic neurons in the caudal solitary nucleus (cNTS). However, the neural circuits on these respiratory and arousal responses are not fully understood. Here, we first examined glutamatergic pathways from the cNTS to the medullary respiratory center (rVRG) and arousal-related area in the hypothalamus (PeF) in mice. By genetically labeling glutamatergic cNTS axons, we found their direct connections to the rVRG neurons as well as indirect connections via the parabrachial nucleus (PB) to the rVRG and PeF. Second, by chemogenetically activating glutamatergic cNTS neurons, we demonstrated increase of tidal volume and minute ventilation with no significant change in respiratory rate, and Fos expression in not only rVRG neurons but also PB neurons projecting to the rVRG and PeF. These results suggest that glutamatergic cNTS neurons induce respiratory facilitation by direct and indirect projections via the PB to the rVRG as well as induce arousal by projection to the PeF via the PB. (COI:No)

### MS2-3

### Gut hormone GLP-1 enhances insulin sensitivity via <vagal afferents-brain> axis

Yusaku Iwasaki[1] ([1]*Anim Sci, Grad Sch Life Env Sci, Kyoto Pref Univ*)

Gut hormone glucagon-like peptide-1 (GLP-1) enhances insulin secretion in a blood glucose (BG)-dependent manner. Therefore, GLP-1 receptor agonists have the excellent therapeutic effect for diabetes through the direct action on pancreatic β-cells. While GLP-1 receptor agonists are a stable compound in the circulation, endogenous GLP-1 is an extremely unstable substance. We identified the rare sugar allulose (Allu) as a selective GLP-1 releaser and found that intestinal GLP-1 improved glucose tolerance without significant insulin secretion via vagal afferents (Iwasaki Y, Yada T et al, Nat Com 2018). Therefore, endogenous intestinal GLP-1 might improve glucose tolerance through a novel mechanism of action mediated by vagal afferents. We compared Allu and GLP-1R agonist, exentine-4 (Ex4), in their action to correct hyperglycemia and to case adverse hypoglycemia in normal and diabetic mice. Intestinal GLP-1 exhibited greater BG-lowering effect at higher insulin levels, and no effect at normal glucose levels without causing adverse hypoglycemia. GLP-1 releaser may provide a novel category of GLP-1-based substance/medicine with high efficacy and safety to correct hyperglycemia. (COI:No)

### MS2-4

### Anti-inflammatory mechanism through autonomic-immune system

Tsuyoshi Inoue[1] ([1]*Dept Physiol, Grad Sch Med, Nagasaki Univ*)

In recent years, the anti-inflammatory effect of the autonomic nervous system has attracted attention, and research on the cholinergic anti-inflammatory pathway through the vagus nerve is particularly active. Indeed, we have so far showed the renal protective effect of vagus nerve stimulation and the importance of α7 nicotinic acetylcholine receptor in macrophages, and we identified novel factors existing downstream of α7 nicotinic acetylcholine receptor. In addition, we found that sympathetic nerves are important for the renal protective and anti-inflammatory effects of C1 neurons in the medulla oblongata, and that macrophages exert anti-inflammatory and renal protective effects through β2 adrenergic receptors. In this session, I will briefly show anti-inflammatory mechanism mediated by the autonomic-immune system including the latest findings. (COI:No)

### MS2-5

### Identifying neural circuits important for kidney protection induced by vagus nerve stimulation

Shinji Tanaka[1] ([1]*Division of Nephrology, University of Virginia*)

Acute kidney injury (AKI) is highly prevalent and associated with high morbidity and mortality, and there are no approved drugs for the prevention and treatment of AKI. Vagus nerve stimulation (VNS) alleviates inflammatory diseases including kidney disease; however, neural circuits involved in the tissue protection by VNS remain poorly understood. The vagus nerve is a heterogenous group of neural fibers innervating a number of organs. VNS broadly stimulates these fibers without specificity. We used optogenetics to selectively stimulate vagus efferent and afferent fibers. We demonstrated that anterograde efferent fiber stimulation or anterograde sensory afferent fiber stimulation both confer kidney protection from ischemia-reperfusion injury. We also identified the C1 neurons?sympathetic nervous system?splenic nerve?spleen?kidney axis as the downstream pathway of anterograde vagus afferent fiber stimulation. These findings provide a novel map of the neural circuits important for kidney protection induced by VNS, which is critical for the safe and effective clinical application of VNS for protection from AKI. (COI:No)

### Meeting Symposium3

### Cutting-edge researches on the organogenesis of hypothalamus-pituirary systems

(March 28, Sun. 9：00～11：00, Room5)

### MS3-1

**Regulation mechanism of vascular structure of the anterior pituitary**

Takashi Nakakura [1] ([1]*Dept Anat, Sch Med, Teikyo Univ, Tokyo, Japan*)

The anterior pituitary (AP) is vascularized by a hypophyseal portal system (HPS), which is a fenestrated capillary that connects the AP with the median eminence of the hypothalamus. Capillary fenestrae are the transcellular pores and act as the passage holes for peptide hormones. Each pore is about 70 nm in diameter and divided by a diaphragm made of PLVAP. We previously showed that VEGF-A, a major secreted angiogenic factor, is involved in the formation of rat HPS (Nakakura et al. *Cell Tissue Res* 2006). We also showed by using the transgenic *Xenopus* that vascularization by overexpresses of VEGF-A in intermediate pituitary, which is originally non-vascular tissue, affects the differentiation of the endocrine cells (Tanaka, Nakakura et al. *Gen Comp Endocrinol* 2013). Recently, we recruited the primary culture system of endothelial cells isolated from rat AP and found that fibronectin, a component of the vascular basement membrane, is required to maintain the fenestration of the cells (Nakakura et al. *Cell Tissue Res* 2020). We also found that the endomembrane system and microtubule cytoskeletons are essential for the maintenance of PLVAP at the fenestrae and morphology of pores. (COI:No)

### MS3-2

**The role of folliculo-stellate cells as a supporting pituitary stem cells in the adulthood**

Ken Fujiwara[1] ([1]*Department of Biological Sciences, Faculty of Science, Kanagawa University*)

Anterior pituitary gland is composed of five types of hormone-producing cells and folliculo-stellate cells (FS cells). FS cells interconnect homotypically to build 3-dimensional meshwork, which assist in retaining mechanical structure in the anterior pituitary. In addition to structural function, the FS cells are also believed to be involved in cell renewal and supply for hormone-producing cells. Several recent studies demonstrated that a part of FS cells express SOX2, and that hormone-producing cells could be induced from FS cells under culture condition. However, it remains unclear how these stem cells are maintained in the adult anterior pituitary gland. In order to identifying factors involved in the maintenance of stem cells, we performed transcriptome analysis in rat FS cells. We isolated FS cells from adult S100b-GFP transgenic rats, and then compared the genes expressions between FS cell and other pituitary cells by a DNA microarray. Through this analysis, we found that FS cell produces several humoral factors that may support adult stem cells. In this talk, I would like to discuss the involvements of FS cells in the stem cell maintenance in the adult pituitary gland. (COI:No)

### MS3-3

**Cluster of differentiation (CD) 9-positive pituitary cells are adult stem/progenitor cells.**

Kotaro Horiguchi[1] ([1]*Dept. Health Sci. Kyorin Univ.*)

A supply of hormone-producing cells from stem/progenitor cells is critical to sustain the endocrine activity of the pituitary gland. In the adenohypophysis composing the anterior and intermediate lobe, sex-determining region Y-box 2 (SOX2)-positive cells are stem/progenitor cells that supply hormone-producing cells. However, they are likely composed of several subpopulations. In rodents, a SOX2-positive cell population can be distinguished by the presence of S100β. We identified the novel markers cluster of differentiation (CD) 9 and CD81, which are members of the tetraspanin superfamily, for the identification of S100β/SOX2-positive cells. Although CD9/CD81 double-knockout mice grew normally until 3 weeks after birth, they exhibited atrophy of the pituitary gland. These findings suggested that CD9/CD81/S100β/SOX2-positive cells in the pituitary are adult stem/progenitor cells. We succeeded in isolating CD9-positive cells from the anterior lobes of adult rodents and showed the capacity to form pituispheres and differentiate into hormone-producing cells. These findings indicate that CD9-positive cells may play role as adult stem/progenitor cells in SOX2-positive subpopulations. (COI:No)

### MS3-4

***In vitro* recapitulation of hypothalamic tanycyte development using embryonic stem cell culture**

Yu Kodani[1], Miho Kawata[1], Yoko Kaneko[1,2], Akira Nakashima[3], Hiroshi Nagasaki[1] ([1]*Dept Physiol, Fujita Health Univ Sch Med*, [2]*Fac Pharm, Gifu Univ Med Sci*, [3]*Dept Physiol Chem, Fujita Health Univ Sch Med*)

Tanycytes are specialized ependymal cells lining the third ventricle and act as adult neural stem cells (NSCs) that modulate hypothalamic function through neurogenesis. During embryonic development, the earliest hypothalamic NSCs occur as neuroepithelial cells (NECs) of the neural tube and then convert to the second form of NSCs called radial glial cells (RGCs). Tanycytes are considered to be remnants of RGCs with a limited NSC activity, but their maturation process remains enigmatic. We recently established a 3D culture model of hypothalamic development using mouse embryonic stem cells (mESCs). To examine tanycyte generation in this model, we traced phenotypic changes of $Rax^+$ cells because Rax is a transcription factor common to hypothalamic NECs, RGCs, and tanycytes. As expected, mESC-derived $Rax^+$ cells showed a stepwise transition from NEC-like to tanycyte-like state in terms of morphology, gene expression, and proliferative capacity. By analyzing this transition process, we newly identified a cell surface marker upregulated in tanycytes. Our findings highlight the utility of mESC differentiation system for studying tanycyte development and function. (COI:No)

### MS3-5

**Generation of hypothalamic-pituitary unit from ES/iPS cells**

Hidetaka Suga[1], Hiroshi Arima[1] ([1]*Department of Endocrinology and Diabetes, Nagoya University Graduate School of Medicine*)

The hypothalamic-pituitary system is essential for maintaining homeostasis. We have established techniques that allow the generation of functional adenohypophysis and hypothalamus from pluripotent stem cells.

Recent results have shown that strict removal of exogenous patterning factors during the early differentiation period induces rostral hypothalamic-like progenitors, which means the hypothalamus position in the cerebral nervous systems is characterized by the most rostral. Those progenitors generated hypothalamic neurons, in particular magnocellular vasopressinergic neurons, which release hormones upon stimulation.

We have subsequently succeeded in inducing both hypothalamic and oral ectodermal tissues simultaneously. Self-organization of Rathke's pouch, pituitary primordium, occurred at the interface of the two epithelia. Corticotrophs and somatotrophs were produced from the Rathke's pouch-like structures. The induced corticotrophs efficiently secreted adrenocorticotropic hormone (ACTH) in response to corticotropin-releasing hormone (CRH). Thus, we have generated a useful methodology for the production of functional hypothalamic-pituitary tissue. (COI:No)

### Meeting Symposium4

### Neural mechanisms of motivated and goal-directed behaviors

(March 28, Sun. 9：00～11：00, Room6)

### MS4-1

**Neural mechanisms of motivational control based on cost-benefit comparison in monkeys**

Takafumi Minamimoto[1] ([1]*Dept. Funct. Brain Imaging, NIRS, QST, Chiba, Japan*)

The motivation to engage in goal-directed behavior arises from expected reward value (nature and size of the reward) and the cost associated with its obtention, such that the net [motivational] value decreases with the delay, risk, or effort. We assessed monkeys' motivation to perform an instrumental lever-release to obtain a reward, which reflects the expected reward amount and additional cost (i.e., delay or workload) to obtain it. We showed that the incentive task performance was markedly distorted when the orbitofrontal cortex (OFC), rostromedial caudate nucleus (rmCD) or the ventral pallidum (VP) was chemogenetically or pharmacologically inactivated bilaterally. Neuronal activity of rmCD and VP encode incentive value during cue periods, together suggesting that the serial circuit connected through OFC-rmCD-VP have an essential role in incentive motivation. Similarly, we demonstrated that the activity of the dorsal part of the caudate head (dCDh) causally related to the temporally discounted value, suggesting an essential contribution of dCDh to integrate cost and benefit for motivation. (COI:No)

### MS4-2

**Effects of repeated social defeat stress on effort-based decision-making behavior in mice**

Mayumi Nishi[1], Nozomi Endo[1], Nami Somayama[1] ([1]*Department of Anatomy & Cell Biology*)

Repeated social defeat stress (RSDS) is commonly employed as an ethologically relevant stressor in rodents. Recently, many studies have shown that the RSDS affects depressive--like behaviors that modify motivation. We examined motivation related to reward acquisition in RSDS mice by using our original effort-based decision-making test. In this test, chocolate as a high-reward and food pellet as a low-reward were placed in each side of the experimental box. A high wall was set upped as a high-cost in front of the chocolate, while a low wall was set upped as a low-cost in front of the food pellet. Mice were allowed to select either high-reward/high-cost or low-reward/low-cost option. Interestingly, RSDS mice were divided into two groups; RSDS mice that chose the comparable percent of high-reward/high-cost option as with control mice and RSDS mice that did not absolutely choose high-reward/high-cost option. These findings suggest that RSDS shows abnormal reward-cost ratios in effort-based decision-making which causes individual differences in susceptibility to the same stress. We are now analyzing the neural basis of the effects of RSDS on effort-based decision-making behavior. (COI:No)

### MS4-3

**Opposing medial vs. lateral accumbal projection neuron activities are shaped by striatal interneurons during goal-directed behaviors**

Kenji Tanaka[1] ([1]*Keio Univ Sch Med*)

Medium spiny neurons (MSNs) of mice show opposing activities upon initiation of a food-seeking lever press task: ventromedial striatal (VMS)-MSNs are inhibited but ventrolatelal striatal (VLS)-MSNs are activated. These activities mediate action selection and action initiation, respectively. However, what input shapes the opposing MSN activities is largely unknown. Here we monitored cortical input activities at the cell-population level and artificially reversed them to understand whether the input shares the role of the respective MSNs. The ventral hippocampus (vHP) and insular cortex (IC) are major inputs to VMS and VLS, and both projections showed silencing at the trial start time. We demonstrated that the vHP-VMS and IC-VLS pathways formed functionally coupled input-output units. The former input-output activity relation was parallel but the latter was opposing, indicating the absence and existence of feedforward disinhibition, respectively. We demonstrated that a biased localization of striatal parvalbumin-interneurons generated the opposing MSN activity during the task. (COI:No)

### MS4-4

**The role of monkey orbitofrontal cortex in reward value computation**

Tsuyoshi Setogawa[1], Takashi Mizuhiki[2], Narihisa Matsumoto[3], Barry Richmond[4], Munetaka Shidara[5] ([1]*System Emotional Science, Faculty of Medicine, Univ. of Toyama*, [2]*Kurita Hospital*, [3]*Human Informat. Res. Inst., AIST*, [4]*NIMH, NIH*, [5]*Faculty of Medicine, Univ. of Tsukuba*)

When faced with having to choose one from some alternatives, animals, including humans, will normally choose more valuable options than less valuable ones. Previous studies have reported that neuronal activities in orbitofrontal cortex (OFC) are related to the subjective values of offered options. However, it is still unclear whether 1) neurons in OFC encode the difference in value between offered options, and 2) there is a causal link between OFC neuronal activity and choice. Here we developed a decision-making task in which two choice targets were sequentially presented before choice, and recorded 256 single neurons from monkey OFC. For 56/256 (21.9%) of the recorded neurons, their activities were significantly correlated with the subtraction between the values of the presented choice targets, suggesting that these neuronal responses encode the difference in value between offered options. Inactivating OFC by muscimol led the monkeys more likely to choose the less valuable option, when the difference in value was small. These results suggest that OFC neurons code for value information that could be used to guide choices, and these signals have a direct influence on the choice. (COI:No)

### MS4-5

**Neuronal encoding of locomotion during goal-directed behavior in the mouse dorsomedial prefrontal cortex**

Hiroshi Nishimaru[1], Sachu Riga[1], Yusaku Takamura[1], Junpei Matsumoto[1], Mariana Ferreira Pereira de Araújo[2], Taketoshi Ono[1], Hisao Nishijo[1] ([1]*System Emotional Science, Faculty of Medicine, University of Toyama*, [2]*Department of Physiological Sciences, Health Sciences Center, Federal University of Espirito Santo, Brazil*)

It has been shown that the prefrontal cortex (PFC) plays a crucial role in goal-directed behaviors. In particular, the dorsomedial PFC including the anterior cingulate cortex (ACC) has been implied in a wide range of relevant functions in these behaviors, such as motor control, sensory processing, motivational control and the encoding value of the resulting outcome. In many mammals, including rodents, goal-directed behavior involves overground locomotion such as walking and running, which allows the animal to move from one location to another. However, how the dorsomedial PFC neurons dynamically encode such information while the animal repeatedly locomotes for the reward remains unclear. In this talk, we would like to present our recent data on how the action and its values are encoded in ACC neurons in mice during a locomotor task that requires the animal to spontaneously and repeatedly initiate and terminate running to obtain rewards. (COI:No)

### Meeting Symposium5

### Neonatal and adult neurogenesis: novel regulatory mechanisms, functional implications, and contribution to disease pathology

(March 28, Sun. 9：00～11：00, Room7)

### MS5-1

**Roles of acid-sensing ion channel-1a in adult hippocampal neurogenesis**

Natsuko Kumamoto[1], Yasuhiro Shibata[1], Takashi Ueda[1], Shinya Ugawa[1] ([1]*Dept Anat & Neurosci, Grad Sch Med Sci, Nagoya City Univ, Aichi, Japan*)

It is well known that adult hippocampal neurogenesis is enhanced after ischemic brain injury. Local tissue acidosis occurs in lesion area, which may be one of the key factors affecting neurogenesis. ASIC1a (acid-sensing ion channel-1a) is a neuronal acid-activated cation channel located in the postsynaptic membrane. The channel receives synaptic protons, contributing to synaptic plasticity, learning and memory. To explore the potential roles of ASIC1a in adult hippocampal neurogenesis, we used an onco-retrovirus-mediated approach to genetically label and manipulate newborn dentate granule cells (DGCs) *in vivo*. Three-dimensional reconstruction of confocal Z-stacks was applied to morphologically characterize the dendritic arborization and spine formation of ASIC1a-deficient newborn DGCs labeled with EGFP. We found that ASIC1a in newborn DGCs mediates dendrite growth, complexity and spine development in the normal brain. Further understanding of the involvement of ASIC1a in hippocampal neurogenesis may lead to new therapeutic strategies to actively repair the damaged brain. (COI:No)

### MS5-2

**Novel mode of antidepressant action based on exercise-induced beneficial effects**

Makoto Kondo[1] ([1]*Dept Neurosci & Cell Biol, Grad Sch Med, Osaka Univ*)

Major depression is a highly prevalent mental disorder. Although selective serotonin reuptake inhibitors (SSRIs) are the most widely used antidepressants, a significant proportion of depressed patients do not achieve remission. Physical exercise provides neurogenic and antidepressant effects, and we recently demonstrated that the serotonin type 3 receptor (5HT3R) is essential for exercise-induced hippocampal neurogenesis and antidepressant effects. Therefore, we examined the 5HT3R-mediated mechanism in detail. In this study, we showed that treatment with a 5HT3R agonist induces antidepressant effects and increases hippocampal neurogenesis, independent of fluoxetine (a commonly used SSRI). In addition, histological analyses revealed that 5HT3R and IGF1 are expressed in the same neurons in the hippocampal dentate gyrus. Furthermore, in vivo microdialysis analysis showed that 5HT3R agonist increases extracellular IGF1 levels in the hippocampus, and that IGF1 signaling is required for the 5HT3R-dependent hippocampal neurogenesis. Our findings suggest a novel 5HT3R-IGF1 mechanism distinct from fluoxetine-induced responses, which could provide a new therapeutic target for depression. (COI:No)

### MS5-3

**Adult neurogenesis in the human hippocampus**

Tatsunori Seki[1] ([1]*Dep Hist Neuroanat, Tokyo Medical Univ.*)

The concept of adult hippocampal neurogenesis (AHN) has been widely accepted, and a large number of studies have been performed in rodents using modern experimental techniques, which have clarified the nature and developmental processes of adult neural stem/progenitor cells, the functions of AHN, such as memory and learning, and its association with neural diseases. However, a fundamental problem is that it remains unclear as to what extent AHN actually occurs in humans. The answer to this is indispensable when physiological and pathological functions of human AHN are deduced from studies of rodent AHN, but there are controversial data on the extent of human AHN. Recent our study suggests that immature neuronal marker-expressing neurons are abundant in the adult human hippocampus, but the level of neuronal production is low. In this talk, studies on AHN performed in rodents and humans will be briefly reviewed, followed by studies in nonhuman primates that fill the gap between the 2 species. Then, how data of rodent and nonhuman primate AHN should be applied to understanding human AHN will be discussed. (COI:No)

### MS5-4

**Mechanisms for maintenance and termination of neuronal migration in the postnatal brain**

Masato Sawada[1,2], Kazunobu Sawamoto[1,2] ([1]*Dept Dev Regen Neurobiol, Inst Brain Sci, Nagoya City Univ Grad Sch Med Sci, Aichi, Japan,* [2]*Div Neural Dev Regen, NIPS, Aichi, Japan*)

In the postnatal brain, neural stem cells in the ventricular-subventricular zone (V-SVZ) constantly generate new neurons. These neurons form chain-like aggregates and migrate along each other toward the olfactory bulb (OB). After arriving the OB, new neurons migrate radially toward the OB layers, where they differentiate into mature interneurons. Altering the migration of new neurons affects their positioning and functions in the OB, suggesting that regulation of neuronal migration is critical for the plasticity of postnatal OB circuits.
We have been studying the mechanisms for maintenance and termination of migration of new neurons in the OB. We found that new neurons transiently extend a novel cellular protrusion called "filopodium-like lateral protrusion (FLP)" during migration termination in the OB. The FLP formation coincided with microtubule reorganization, primary cilium extension, and pausing of somal translocation. The timing of migration termination, controlled by PlexinD1 signaling, affects the final positioning and functions of new neurons in the OB. Our results highlight the importance of the mechanisms for the proper positioning of cells in postnatal OB circuits. (COI:No)

### MS5-5

**Synaptic incorporation of new neurons in the olfactory bulb by olfactory learning**

Masahiro Yamaguchi[1] ([1]*Dept Physiol, Kochi Med Sch, Kochi Univ*)

In the mammalian olfactory bulb (OB), newly generated interneurons, granule cells (GCs) and periglomerular cells, are continuously incorporated into adult neuronal circuit. Among new GCs, nearly half are incorporated while the others are eliminated, and this selection is important to fine-tune OB circuit. While peripheral odor inputs are important in GC incorporation, combination with odor learning, but not simple odor exposure, efficiently promotes GC incorporation. However, the synaptic mechanisms of the GC incorporation are not well understood. Here we stimulated peripheral odor inputs by using optogenetics, and classified new GC synapses into those received peripheral inputs and those did not. While simple delivery of peripheral inputs showed no apparent difference in the size and distribution between the two groups of GC synapses, combination with olfactory learning showed preferred preservation of GC synapses that received peripheral inputs. The results indicate the importance of olfactory learning in the effective utilization of new GCs at synaptic level. Underlying synaptic mechanisms will be discussed. (COI:No)

### MS5-6

**Function of adult-born neurons for memory consolidation in sleep**

Masanori Sakaguchi[1] ([1]*WPI-IIIS, U Tsukuba*)

The occurrence of dreaming during rapid eye movement (REM) sleep prompts interest in the role of REM sleep in hippocampal-dependent episodic memory. Within the mammalian hippocampus, the dentate gyrus (DG) has the unique characteristic of exhibiting neurogenesis persisting into adulthood. Despite their small numbers and sparse activity, adult-born neurons (ABNs) in the DG play critical roles in memory; however, their memory function during sleep is unknown. Here, we investigate whether young ABN activity contributes to memory consolidation during sleep using $Ca^{2+}$ imaging in freely moving mice. We found that contextual fear learning recruits a population of young ABNs that are reactivated during subsequent REM sleep against a backdrop of overall reduced ABN activity. Optogenetic silencing of this sparse ABN activity during REM sleep alters the structural remodeling of spines on ABN dendrites and impairs memory consolidation. These findings provide a causal link between ABN activity during REM sleep and memory consolidation. (COI:No)

### Meeting Symposium6

### Physiology of energy homeostasis and pathophysiology of obesity

(March 28, Sun. 14：20～16：20, Room5)

### MS6-1

**Nutrient and hormone signals in appetite dysregulation - From animal studies to clinical care of obesity**

Daisuke Aotani[1], Tomohiro Tanaka[1] ([1]*Department of Gastroenterology and Metabolism Nagoya City University Graduate School of Medical Science*)

In clinical medicine, the effectiveness of currently-approved non-surgical treatments of obesity such as lifestyle intervention or drugs is limited. More importantly, they are not fully based on our understanding of the basic physiology of body weight regulation. Leptin, a satiety signal from adipose tissue regulates energy homeostasis by acting on receptors in the hypothalamus. However, leptin functions in the hypothalamus are reduced in obesity, termed "leptin resistance". We demonstrated that leptin exerts its effect through the suppression of the hedonic feeding pathway of the reward system in both human and rodent. Furthermore, our data suggests that the effect to leptin on the reward system is also attenuated under conditions of leptin resistance. We then tried to elucidate the potential mechanism of diet-induced leptin resistance. As a result, diet-derived lipid moieties actively changed the hypothalamic lipid composition and may play a critical role in the development of leptin resistance. In our institution, the Center for Obesity Research and Therapeutics (CORT) was established in 2019, aimed at the creation of new mechanism-based methods for the combat against obesity. (COI:No)

### MS6-2

**Roles of endogenous NPGL/NPGM system in energy metabolism**

Kenshiro Shikano[1], Reiko Hanada[1] ([1]*Department of Neurophysiology, Faculty of Medicine, Oita University, Japan*)

Hypothalamus is the center of feeding behavior and energy metabolism, and several hypothalamic neural substrates regulate energy metabolism. We found novel secretory proteins and named them neurosecretory protein GL (NPGL) and neurosecretory protein GM (NPGM) in 2014. Our previous data showed that NPGL or NPGM administration promotes feeding behavior and fat accumulation. However, the mechanisms of energy metabolism mediated by NPGL/NPGM system has not yet totally elucidated.

To investigate the loss of function of NPGL/NPGM system, we have established NPGL and NPGM double knockout mice (NPGL/NPGM dKO). NPGL/NPGM dKO had a lean phenotype under a high fat diet condition because of decreasing food intake and increasing energy expenditure compared with wild type mice. Furthermore, NPGL/NPGM dKO showed not only decreasing fat deposition but also augmenting thermogenic uncoupling protein 1 (UCP1) expression in brown adipose tissue. These results imply that endogenous NPGL/NPGM system has important roles in feeding behavior and energy expenditure. The findings of NPGL/NPGM system will provide us a novel insight into the mechanism of energy metabolism. (COI:No)

### MS6-3

**The effects of exercise training on differential ability of adipose-derived stem cells**

Hisashi Kato[1], Tetsuya Izawa[2] ([1]*Org for Res Initiatives and Dev, Doshisha Univ.*, [2]*Grad Sch of Health & Sports Sci, Doshisha Univ.*)

There is a growing interest to understand mechanisms of adiposederived stem cell (ADSC) differentiation of which dysregulations might be a key factor determining the obesity-related metabolic diseases. Habitual physical exercise, a representative useful tool for prevention obesity, is reported to alter differentiation potential of ADSC into several cell types. However, the underlying molecular mechanisms are not fully understood. To clarify the issue, we focused on the impact of exercise training on the role of microRNA (miRNA) and extracellular vesicles derived from ADSC (ADSC-EVs) in controlling the differentiation potential of ADSC into adipocyte in rats. After a 9-week intervention, ADSC was isolated from inguinal and epididymal adipose tissue of either sedentary or exercise-trained group. Thereafter, microarray analysis of either miRNA or ADSC-EVs was performed. The data obtained indicate that exercise training blunted the ability of ADSC to differentiate into adipocyte with altered profiles of miRNAs compared with sedentary group. Possible alterations in miRNA of ADSC may be involved in the mechanisms underlying the beneficial effects of exercise on the prevention of obesity. (COI:No)

### MS6-4

**Roles of oncostatin M, a member of IL-6 family of cytokine, in obesity and its related disorders**

Tadasuke Komori[1], Yoshihiro Morikawa[1] ([1]*Dept Anat & Neurobiol, Wakayama Med Univ*)

Obesity induces chronic low-grade inflammation that potentiates the development of metabolic diseases. A member of IL-6 family of cytokine, oncostatin M (OSM), is related to various inflammatory diseases. Now, we focused on uncovering the roles of OSM in obesity-related metabolic diseases. The expressions of OSM and OSM receptor β (OSMR β) were increased in the adipose tissue of obese mice. High-fat diet-induced metabolic disorders, including obesity, adipose tissue inflammation, insulin resistance, and hepatic steatosis, were aggravated in mice lacking OSMRβ compared to those in wild-type littermates, suggesting that defects of OSM signaling is important to the development of obesity-related metabolic diseases. In addition, the treatment of diet-induced obese mice with OSM dramatically improved such metabolic disorders. We recently observed that the expression of OSM was up-regulated after exercise in the skeletal muscle. Exercise is one of the most effective non-pharmacological approaches for metabolic diseases. Our results suggest that OSM contribute to the anti-metabolic effects of exercise and is a promising therapeutic target for metabolic diseases. (COI:No)

### MS6-5

**How do emotions impact thermogenic and cardiovascular functions?**

Kazuhiro Nakamura[1], Naoya Kataoka[1,2], Akihiro Fukushima[1] ([1]*Dep Integrative Physiol, Nagoya Univ Grad Sch Med*, [2]*Nagoya Univ Inst Adv Res*)

Emotions strongly impact thermogenic and cardiovascular functions by affecting the central sympathetic regulatory system. We have discovered a central neural pathway that mediates psychological stress signaling by connecting the corticolimbic emotion circuit to the hypothalamomedullary autonomic regulatory system. This is a master psychosomatic pathway that drives a variety of sympathetic and behavioral stress responses, such as brown adipose tissue (BAT) thermogenesis, tachycardia, and pressor response. Recently, we have also identified a central circuit mechanism by which the oxytocin nervous system, which is stimulated by emotional cues, impacts metabolic and cardiac functions. *In vivo* optogenetic and physiological studies revealed that oxytocinergic inputs from the hypothalamus to medullary sympathetic premotor neurons stimulate BAT thermogenic and cardiac responses by potentiating glutamatergic sympathoexcitatory efferent transmission. Based on these findings, we will discuss central psychosomatic circuit mechanisms that underlie a variety of phenomena due to "mind-body" relationship in health and disease. (COI:No)

### Meeting Symposium7

### Visceral pain and sensaton: basic mechanisms and pathophysiological aspects

(March 28, Sun. 16：30～18：30, Room4)

### MS7-1

**Mechanisms of abnormal gastrointestinal motility and hyperalgesia in functional gastrointestinal disorders**

Hiroaki Okuda[1], Yu Kozakai[1], Aye-Mon Mon[1], Kiyomi Hori[1], Kwankaew Nichakarn[1], Tatsuya Ishikawa[1], Noriyuki Ozaki[1] (*[1]Dept Functional Anat, Grad Med Sci, Kanazawa Univ*)

Pain can be classified as "somatic pain" and "visceral pain" in their origin. Compare to somatic pain, sensory mechanisms and factors that contribute to the pathogenesis of visceral pain are poorly understood.
In recent years, awareness of functional gastrointestinal disorders (FGIDs) has increased due to their high prevalence. FGIDs are defined by gastrointestinal symptoms occur in any parts of gastrointestinal tracts from mouth to anus, including irritable bowel syndrome, functional dyspepsia (FD), and non-diffuse gastroesophageal reflux disease. Visceral hypersensitivity and abnormal gastrointestinal motility are thought to be involved in various symptoms such as visceral pain, early fullness, nausea, bloating, vomiting, diarrhea, and constipation observed in patients of FGIDs. To develop effective treatments for FGIDs, it is important to elucidate the underlying mechanisms.
We will present study focuses on FD, analyzing the mechanism of visceral hypersensitivity and abnormal gastrointestinal motility, and noted therapeutic drug candidates. In addition, we will present our study focuses on visceral hypersensitivity associated with diabetic gastropathy. (COI:No)

### MS7-2

**Role of macrophage-derived HMGB1, a possible therapeutic target, in visceral pain**

Atsufumi Kawabata[1] (*[1]Laboratory of Pharmacology and Pathophysiology, Faculty of Pharmacy, Kindai University*)

High mobility group box 1 (HMGB1), a nuclear protein, once released from necrotic cells or secreted by immune cells, etc., aggravates inflammatory responses and tissue injury, hence a typical protein of damage-associated molecular patterns (DAMPs). We have shown that peripheral HMGB1 is pro-nociceptive, and that macrophage (Mϕ)-derived HMGB1 participates in the neuropathic pain induced by spinal nerve injury or cancer chemotherapeutics. Interestingly, Mϕ-derived HMGB1 is also involved in the development of pancreatic, bladder and colonic pain. Therefore, pharmacological deletion of HMGB1 by anti-HMGB1-neutralizing antibodies or by thrombomodulin alfa, capable of causing thrombin-dependent degradation of HMGB1, is considered beneficial for treatment of visceral pain. Membrane receptors, such as the receptor for advanced glycation end-products (RAGE) and Toll-like receptor 4 (TLR4), activated by HMGB1 also appear to serve as therapeutic targets for treatment of visceral pain. Thus, I will show our recent findings concerning the role of HMGB1 and its upstream/downstream signaling molecules in the development and/or persistence of visceral pain. (COI:Properly Declared)

### MS7-3

**Spinal micturition control by sensory synaptic responses evoked in parasympathetic neurons**

Hidemasa Furue[1], Atsushi Hakozaki[2] (*[1]Dept Neurophysiol, Hyogo Col Med, [2]Dept Inf Physiol, NIPS* )

We have developed *in vivo* patch and extracellular recording techniques to detect excitation of the parasympathetic preganglionic neurons in the lumbosacral spinal cord in combination with urinary bladder contraction monitoring, and examined synaptic responses in the preganglionic neurons evoked by afferent fibers. Spinal preganglionic neurons evoked excitatory postsynaptic currents in response to lumbosacral afferent dorsal root stimulation. *In vivo* extracellular recordings from the lumbosacral parasympathetic nucleus showed that spontaneous firing was detected *in vivo* with characteristic bursts of firing coinciding with the increases in intravesical pressure during micturition. *In vivo* whole-cell recordings from spinal parasympathetic preganglionic neurons also showed similar bursts of action potentials associated with the increased intravesical pressure and showed the characteristic morphological features of parasympathetic preganglionic neurons. The *in vivo* analyses also revealed that synaptic inputs from the primary afferent fibers play an important role on setting the threshold for normal micturition reflex. (COI:No)

### MS7-4

**Visceral sensory information activates parabrachio-amygdaloid system**

Yukari Takahashi[1,2], Takayuki Matsushita[1,2,3], Saaya Kojima[1,2,4], Takao Okuda[1,2], Yosuke Oto[1,2,3], Yae Sugimura[1,2], Fusao Kato[1,2] (*[1]Dept Neurosci, Jikei Univ Sch Med, [2]Cent Neurosci Pain, Jikei Univ Sch Med, [3]Dev Rheumatology, Dept Int Med, Jikei Univ Sch Med, [4]Dept Pharmacol, Hoshi Univ*)

Three pathways for the brain to receive visceral sensory information are i) the vagus nerve to the nucleus of the solitary tract (NTS), ii) the spinal nerve to the dorsal horn (DH), and iii) sensory circumventricular organs, such as the area postrema (AP). Interestingly, all of these have rich direct projections to the parabrachial nucleus (PBN), a site for central surveillance of adverse situations, and then to the central amygdala, an essential site for defensive survival functions (Sugimura et al., 2016). Here I introduce examples for visceral information convergence and plasticity in the AP/NTS/PBN/CeA system. 1) Using “FosTRAP” technology, we found that the neuronal population for somatic pain in the PBN and CeA overlaps that for visceral pain but to a limited extent, 2) intraperitoneal injection of lipopolysaccharide increases FOS expression in the PBN and CeA and modify cell excitability, and 3) sustained peripheral inflammation with rheumatoid arthritis activates microglia in the AP and alters spontaneous behaviors (Oto et al, 2019). We propose that this system is the kernel for the emotional/defensive outcomes of the visceral sensation. (COI:No)

### MS7-5

**Involvement of TRP channels in intraoral pain**

Masamichi Shinoda[1] (*[1]Department of Physiology, Nihon University School of Dentistry*)

The oral cavity is the boundary region between the body surface and gastrointestinal tract, intraoral pain also has different characteristics from somatic pain and visceral pain. The intraoral and face skin incisions caused pain hypersensitivity. The number of TRPV1-immunoreactive (IR) and TRPA1-IR trigeminal ganglion (TG) neurons innervating the intraoral mucosa and facial skin were significantly increased, and the number of TRPV1/TRPA1-IR TG neurons innervating facial skin, but not the intraoral mucosa, was significantly increased. Altered expressions of TRPV1 and TRPA1 in TG neurons may contribute to the discrepancy between intraoral and facial skin incisional pain.
Burning mouth syndrome (BMS) which is an intractable intraoral sensory disorder is characterized by altered sensory qualities, particularly pain hypersensitivity without any apparent histological changes, and its sensory deficits resemble those of irritable bowel syndrome. We have established in BMS model rodents and found that tongue mucosal Artn overexpression in BMS potentiates the tongue TG neuron excitability due to TRPV1 overexpression attributable to the enhancement of GFRα3 signaling, resulting tongue pain. (COI:No)

### Meeting Symposium8

### Glial research to understand brain formation, maturation and plasticity

(March 29, Mon. 9：00～11：00, Room3)

### MS8-1

**In vivo imaging of the microglial brain surveillance that dictates synapse formation and elimination in learning and disease**

Ako Ikegami[1], Andrew J Moorhouse[2], Daisuke Kato[1], Zhongtian Guo[1,3], Junichi Nabekura[4], Hiroaki Wake[1] (*[1]Department of Anatomy and Molecular Cell Biology, Nagoya University Graduate School of Medicine, Nagoya, Japan, [2]School of Medical Sciences, UNSW Sydney, Sydney, Australia., [3]Department of System Neuroscience, Kobe University Graduate School of Medicine, Kobe, Japan, [4]Division of Homeostatic Development, National Institute for Physiological Sciences, National Institutes of Natural Sciences, Okazaki, Japan.*)

Microglia are vital to neuronal circuit regulation, contributing to morphological and functional synaptic plasticity. Yet how they actively affect synaptic plasticity remains unknown.
Here, we identified microglia-synapse interactions and their effect on synaptic remodeling in physiology and pathology.
*In vivo* imaging of mice during motor learning revealed that microglial contacting of dendrites induces rapid spine formation in the early phase of learning. In the later phase, the processes repetitively made contact with more specific spines that resulted in their elimination. This regulation was lost in schizophrenia model mice. We further identified epigenetic/transcriptome changes in microglia of model mice that showed a common tendency to downregulate synapse-related and microglial signature genes. These microglial movement/molecular alterations were accompanied by spine loss and psychotic behavior in adolescence.
Our findings show microglial processes sense and induce crucial synaptic plasticity at contact sites. Microglia malfunction in schizophrenia model mice, possibly due to a vulnerability to epigenetic changes triggered in fetal microglia exposed to immune activation. (COI:No)

### MS8-2

**Astrocytes rehabilitate noxious circuits in chronic pain**

Ikuko Takeda[1,2], Kohei Yoshihara[3], Dennis Cheung[2], Makoto Tsuda[3], Kei Eto[4], Andrew Moorhouse[5], Junichi Nabekura[2] (*[1]Department of Anatomy and Molecular Cell Biology Graduate School of Medicine, Nagoya University, [2]Div. of Homeostatic Development National Institute for Physiological Sciences, [3]Department of Molecular and System Pharmacology, Graduate School of Pharmaceutical Sciences, Kyushu University, [4]Kitasato University School of Allied Health Sciences, [5]Department of Physiology, School of Medical Sciences, The University of New South Wales*)

Astrocytes proliferate throughout the central nervous system and are essential for environmental homeostasis. More recently, additional crucial roles in controlling synaptogenesis and synaptic maintenance have been reported. Given this growing appreciation for the importance of astrocytes in facilitating neural circuit rewiring, we developed a novel approach for modulating astrocyte activation in order to stimulate noxious to innocuous circuit reorganization in chronic pain. Through transient astrocyte activation in the somatosensory cortex (S1), established allodynia via prior partial sciatic nerve ligation was alleviated. This therapy engineered S1 spine elimination, presumably corresponding to the dismantling of inappropriate neural connections. Thus, activated astrocytes, by facilitating S1 circuit reorganization, have the potential to cure chronic pain. (COI:No)

### MS8-3

**Microglial dynamics and its contribution to neurogenesis during the embryonic cerebral cortex**

Yuki Hattori[1] (*[1]Dept Anatomy and Cell Biology, Grad Sch Med, Nagoya Univ, Aichi, Japan*)

Microglia, the resident immune cells of the central nervous system, are known to extensively survey the cerebral wall in the embryonic stage. These cells have been reported to provide the particular functions, e.g., phagocytotically regulating the number of neural progenitors and inducing neural stem-like cells to differentiate into intermediate progenitors. We previously reported that, in mouse embryonic brain, microglia transiently disappear from the cortical plate (CP) in the midembryonic stage by their bidirectional migration via the attraction by C-X-C motif chemokine ligand 12 (CXCL12) released from the meninges and subventricular zone, and this microglial temporary absence from the CP is required for proper differentiation of postmigratory neurons that accumulate in the CP. Although such actively migrating microglia are critical for neuronal differentiation, about half of microglia show non-migrating patterns. We found that these stationary microglia contact pericytes surrounding vascular endothelial cells. In this talk, I will discuss how pericytes might be involved in microglial behavior and development in the embryonic brain. (COI:No)

### MS8-4

**Astrocyte-neuron interaction through Gq-protein coupled receptor signaling**

Eiji Shigetomi[1], Shuichi Koizumi[1] (*[1]University of Yamanashi, Japan*)

Emerging evidence suggests that astrocytes control brain functions and animal behavior. Astrocytes receive neuronal information at synapses and display $Ca^{2+}$ signals. $Ca^{2+}$-dependent gliotransmitter (e.g. glutamate, ATP) release is one of well-studied mechanisms of synaptic regulation through astrocytes. However, its dynamic feature and its relevance to brain functions are still not fully understood. To this end, we have developed a method to visualize astrocyte-neuron interaction using $Ca^{2+}$ sensors and neuro-/glio-transmitter sensors and manipulate expression of P2Y1 receptor, one of main Gq-protein coupled receptors expressed in astrocytes. We found that overexpression of P2Y1 receptors in astrocytes enhance neuron-to-astrocytes transmission, leading to robust P2Y1 receptor-mediated $Ca^{2+}$ signals in astrocytes, and augment glutamatergic excitatory synaptic transmission. However, the augmentation in excitatory synaptic transmission is unlikely to be mediated by $Ca^{2+}$-dependent glutamate release. Instead, our data suggest a novel mechanism through increased expression of a signaling molecule which could promote glutamatergic synaptic transmission. (COI:No)

### MS8-5

**Manipulation of astrocytic cAMP modulates memory**

Ryuta Koyama[1] (*[1]Grad Sch Pharmaceut Sci, Univ Tokyo*)

Astrocytes play a key role in brain homeostasis and functions including memory. Specifically, astrocytes express multiple receptors that transduce signals via the second messenger cAMP. However, the involvement of astrocytic cAMP in animal behavior and the underlying glial-neuronal interactions remains largely unknown. Here, we show that an increase in astrocytic cAMP is sufficient to induce synaptic plasticity and modulate memory. We developed a method to increase astrocytic cAMP levels *in vivo* using photoactivated adenylyl cyclase (PAC) and found that increased cAMP in hippocampal astrocytes at different time points facilitated memory formation but interrupted memory retention via NMDA receptor-dependent plasticity. Furthermore, we found that the cAMP-induced modulation of memory was mediated by the astrocyte-neuron lactate shuttle (ANLS). Thus, our study unveils a role of astrocytic cAMP in brain function by providing a novel tool to modulate astrocytic cAMP *in vivo*. (COI:No)

### Meeting Symposium9

### From Reception to Perception, and Disorder: Current Topics in Chemoreception Research

(March 29, Mon. 9：00～11：00, Room4)

### MS9-1

**Cyclic AMP-buffering by Olfactory Marker Protein ensures resilient olfactory neuronal activity in odor-source searching**

Noriyuki Nakashima[1], Akiko Nakashima[1], Kie Nakashima[2], Akiko Taura[3], Harunori Omori[4], Makoto Takano[1] ([1]*Dept Physiol, Kurume Univ Sch Med, Fukuoka, Japan,* [2]*Lab Dev Neurobiol, Grad Sch Biostudies, Kyoto Univ, Kyoto, Japan,* [3]*Dept Med Engineer, Facult Health Sci, Aino Univ, Osaka Japan,* [4]*Dept Physiol, Sch Med, Kanazawa Med Univ, Ishikawa, Japan*)

Olfactory sensation occurs in the cilia of olfactory receptor neurons (ORNs) by converting the odorant-induced cAMP surge into $Na^+/Ca^{2+}$ influx via CNG channels. Under lasting stimulation, olfaction undergoes strong desensitization. However, ORNs must maintain resilient firing even during odor-source searching. We have discovered a novel mechanism that prevents neural desensitization under sensory stimuli. We identified a genetic signature of mature ORNs, the olfactory marker protein (OMP) as a cAMP-binding protein. OMP directly captures the odor-induced surplus cAMP and swiftly reduces the freely available cAMP. This cAMP-buffering process quickly terminates CNG activity, prevents excessive depolarization under repetitive stimulation and maintains the resilient firing responses. Behaviorally, *OMP*$^{-/-}$ mice made frequent errors in odor-source searching due to the cAMP-overload by continual sniffing. The cAMP-buffering by OMP proposes a novel cellular strategy for spatiotemporal regulation of cAMP-associated signaling in ORNs and other OMP-expressing cells throughout the body. (COI:No)

### MS9-2

**Cellular and molecular mechanisms underlying sodium taste in taste buds**

Akiyuki Taruno[1,2] ([1]*Department of Molecular Cell Physiology, Kyoto Prefectural University of Medicine, Japan,* [2]*JST PRESTO, Kawaguchi, Saitama*)

Sodium taste mediates sodium intake. Despite knowledge regarding its sensor molecule amiloride-sensitive epithelial sodium channel (ENaC), our understanding of the cellular and molecular basis of the perception of sodium taste in taste buds remains incomplete. Here, we have identified and characterized sodium taste cells and elucidated the mechanism of peripheral sodium taste. A subset of taste bud cells with ENaC function fire action potentials in response to ENaC-mediated $Na^+$ influx without altering the intracellular $Ca^{2+}$ concentration, and form a channel synapse with afferent neurons involving the voltage-gated neurotransmitter-release channel CALHM1/3. Conditional knockout of ENaC in CALHM1-expressing cells as well as global *Calhm3* knockout in mice negate their ability to perceive sodium taste. Together, cells expressing ENaC and CALHM1/3 constitute sodium taste cells, whereby the entry of $Na^+$ elicits depolarization for action potentials driving voltage-dependent neurotransmission via the channel synapse. Interestingly, this study revealed that, unlike other forms of sensory transduction, all steps in sodium taste signaling are voltage-driven and independent of $Ca^{2+}$ signals. (COI:No)

### MS9-3

**Linkages of Taste and Olfaction: The importance of olfactory system in the taste-guided ingestive motivation to polysaccharides**

Chizuko Inui-Yamamoto[1] ([1]*Dept of Oral Anat & Dev Biol, Osaka Univ Grad Sch of Dent, Osaka, Japan*)

We tested whether motivated behavioral responsiveness to taste compounds is independent of the olfactory system in mice. The bulbotomy severely blunted concentration-dependent licking for the disaccharide sucrose, the maltodextrin Maltrin, the fat emulsion Intralipid relative to their sham surgery in C57BL/6 (BULBx B6) mice. The bulbotomy also decreased licking for the noncaloric sweetener saccharin. In the mice lacking a "sweet" receptor (T1R2+T1R3 knockout), the responsiveness to Maltrin and sucrose was exceptionally curtailed by bulbotomy. Nor did it temper intake of and preference for high concentrations of affectively positive stimuli in the long-term (23-hr) 2-bottle tests demonstrating that the bulbotomy does not lead to a generalized motivational deficit. These results show that specific aspects of taste-guided ingestive motivation are profoundly disturbed by eliminating the anatomical connections between the main/accessory olfactory bulbs and the rest of the brain. It suggests the importance of understanding gustatory function in a broader context that involves a significant contribution from the olfactory system to maintain certain taste-motivated behaviors. (COI:No)

### MS9-4

**Genetic polymorphisms of TRPV1 association with Oral Capsaicin perception and burning mouth syndrome**

Mizuho Kido[1], Junko Yoshizumi[2,5], Reiko Yoshimoto[1], Masahiro Oike[3], Yutaka Takaoka[6], Tomoka Takao[1], Yukiko Oyama[5], Reona Aijima[4], Yoshihide Mori[2], Akira Toyofuku[7], Shunichi Kajioka[8] ([1]*Dept. Anatomy and Physiol, Med Sch, Saga Univ.,* [2]*Dept.Oral & Maxillofacial Surgery,* [3]*Dept. Pharmacology, Grad. Sch.Med. Sci, Kyushu Univ.,* [4]*Dept.Oral and Maxillofacial Surgery, Faculty of Medicine, Saga University,* [5]*Oral and Maxillofacial Surgery, Division of Maxillofacial Diagnostic and Surgical Science, Faculty of Dental Science, Kyushu University,* [6]*Medical Informatics and Bioinformatics, Kobe University Hospital,* [7]*Depart.Psychosomatic Dentistry Grad.Sch.Tokyo Med.Dent. Univ.,* [8]*Department of Pharmaceutical Sciences, School of Pharmacy at Fukuoka, International University of Health and Welfare*)

Capsaicin, an ingredient of hot chili pepper, causes a burning or spicy sensation in our oral cavity and the capsaicin-induced perception varies individuals. Capsaicin is well-known activator of transient receptor potential vanilloid-1 (TRPV1) channel, however, the association of TRPV1 with oral perception of capsaicin and painful disease still remained unclear. We explored the frequencies of two single nucleotide polymorphisms of TRPV1 and the impact of these polymorphisms on the sensory properties of the oral cavity. Furthermore, to know whether TRPV1 polymorphisms affect oral pain, we genotyped 461 healthy people (controls) and 113 patients diagnosed with burning mouth syndrome (BMS) using the International Association for the Study of Pain 2013 criteria. It is because patients with BMS experience persistent burning or tingling pain in the tongue or oral mucosa similar to that evoked by hot chili. Our results suggest that TRPV1genetic polymorphisms affect oral capsaicin perception and oral pain. (COI:No)

### MS9-5

**Smell and taste dysfunctions of COVID-19**

Takaki Miwa[1] ([1]*Kanazawa Medical University, Department of Otorhinolaryngology*)

Smell and taste dysfunctions are characteristic symptoms of the Corona virus disease 2019 (COVID-19). More than half of the patients with COVID-19 complains these symptoms. The clinical characteristics of olfactory dysfunction in COVID-19 are very different from those of other etiologies. Infected patients just present it without other significant complains such as nasal obstruction or discharge. Most of the patients improved their smell function in a few weeks. The pathophysiology of olfactory dysfunction has been clarified by basic research using human and animals. Virus-cell fusion is mediated by angiotensin-converting enzyme 2 (ACE2) and transmembrane serine protease 2 (TMPRSS2) with their organ expression pattern determining affinity of virus to human organs. Both ACE2 and TMPRRS2 are abundant in the nasal cavity and tongue, and are expressed markedly in supporting cells rather than olfactory neurons in the olfactory epithelium. These findings from basic research may be consisted with clinical characteristics of olfactory dysfunction by COVID-19. In this symposium, I would like to present the updated findings of smell and taste dysfunctions by COVID-19. (COI:No)

### Meeting Symposium10

### Regulatory mechanism of cardiovascular development and function

(March 29, Mon. 14：20～16：20, Room6)

### MS10-1

**Regulation of angiogenic events by regulatory protein for heterotrimeric G-proteins**

Motohiko Sato[1], Hisaki Hayashi[1], Aya Yamamura[1] ([1]*Dept of Physiol, Aichi Med Univ*)

Angiogenesis is initiated by various factors including vascular endothelial growth factor (VEGF). Here, we present a role of regulatory protein for heterotrimeric G-proteins in angiogenic events. Activator of G-protein signaling 8 (AGS8) was identified as a receptor-independent accessory protein for the G$\beta\gamma$ subunit from rat heart subjected to repetitive transient ischemia with extensive collateral developments. AGS8 knockdown in human umbilical vein endothelial cells decreased VEGF-mediated proliferation, migration and tube formation. AGS8 knockdown also inhibited VEGF-induced phosphorylation of VEGF receptor 2 and downstream molecules, that was associated with a decrease of cell surface VEGF receptor. Role of AGS8 was further analyzed neovascularization model *in vitro*, which was induced by laser irradiation to mouse choroid. In this model, AGS8 mRNA was upregulated in in the newly formed vessels, and siRNA injection to vitreum significantly inhibited expression of AGS8 and choroidal neovascularization.These data indicate AGS8 regulates VEGF-mediated angiogenic events, and suggest a role of regulatory protein for heterotrimeric G-proteins in tissue development and remodeling. (COI:No)

### MS10-2

**Intercellular communication via extracellular vesicles in tumor angiogenesis**

Nami Yamada[1], Yukihiro Akao[2], Takao Senda[1] ([1]*Dept. Anato. Gifu Univ. Grad. Sch. Med,* [2]*Uni. Grad. Sch. Drug, Med. Info. Sci. Gifu Univ.*)

Extracellular vesicles (EVs) are emerging mediators of intercellular communication. We previously demonstrated that colorectal cancer cells secrete miR-92a-3p via EVs and promote angiogenesis through the downregulation of anti-oncogene Dickkopf-3 (Dkk-3). However, pro-angiogenic function of miR-92a-3p is not only attributable to the downregulation of Dkk-3. Thus, we further performed comprehensive analysis of gene sets affected by the ectopic expression of miR-92a-3p in endothelial cells. Modular enrichment analysis by using GeneCodis was conducted to interpret the underlying biological processes. As results, the ectopic expression of miR-92a-3p upregulated cell cycle- and mitosis-related genes while it downregulated adhesion-related genes in endothelial cells. We also identified *claudin-11* (*CLDN11*), an integral component of tight junction (TJ), as a novel target gene of miR-92a-3p. Our results suggest that the EVs rich in miR-92a-3p induce a pro-angiogenic process called as partial EndoMT in endothelial cells. We would like to present the pro-angiogenic mechanism in detail and discuss potential therapies targeting tumor-derived EVs and microRNAs to prevent cancer progression. (COI:No)

### MS10-3

**Physiological role and three-dimensional localization of cardiac progenitor cells ACMs in the mouse heart**

Mariko Omatsu-Kanbe[1], Ryo Fukunaga[1], Kakeru Shimoda[2], Masakazu Agetsuma[3], Junichi Nabekura[3], Motohiro Nishida[2], Hiroshi Matsuura[1] ([1]*Department of Physiology, Shiga University of Medical Science,* [2]*Cardiocirculatory Signal, NIPS,* [3]*Homeostatic Dev,NIPS*)

The adult mammalian heart comprises several cardiac stem or progenitor cells, though cardiomyocytes do not actually multiply to substitute new cells for damaged ones. Atypically-shaped cardiomyocytes (ACMs) were identified as spontaneously beating cells in the cultures of interstitial cell fractions from adult mouse hearts. ACMs express proteins specific for atrial and ventricular myocytes, SA nodal cells and fetal heart cells, thus suggesting that these cells are likely to be cardiac progenitors rather than cardiac stem cells. These cells displayed peculiar morphology with many protrusions and contain multiple nuclei. ACMs in culture were observed to be fused with neighbored cells resulting in the larger cells with more complicated shapes. The nuclei in ACMs divided irregularly without cytokinesis, suggesting that multinuclear cells are caused by not only cell fusion but also nuclear division. To visualize and trace ACMs in vivo, we prepared GFP-expressing ACMs in mice. 3D-analyses confirmed the localization of ACMs in the interstitial space among ventricular myocytes. The results suggest the possibility that ACMs fuse with existing cells to regenerate injured heart. (COI:No)

### MS10-4

**Role of MAGUK polarity protein DLG1 in the cardiac neural crest and the second heart field development.**

Akiko Kogo[1], Takao Senda[2], Hiroshi Kougo[1], Maiko Ikezawa[1], Toshiyuki Matsuzaki[1] ([1]*Dept Anat Cell Biol, Grad Sch Med, Gunma Univ, Maebashi, Japan,* [2]*Dept Anat, Grad Sch Med, Gifu Univ, Gifu, Japan*)

DLG1 is a MAGUK family protein and involved in many physiological processes including epithelial cell polarization as a scaffolding protein. We have analyzed Dlg1 null mutant mice and found that the mice die shortly after birth with many congenital anomalies of renal and urogenital tract, digestive tract, cochlea, bone, face, heart, and great vessels. In these defects, craniofacial hypoplasia shows complete penetrance and cardiovascular defects including coarctation of aorta and aortic septal defect show incomplete but considerable penetrance, indicating that DLG1 regulates the development of neural crest cells. In addition, other congenital heart defects in Dlg1 KO mice such as ventricular septal defect and double outlet right ventricle suggest developmental defects of the second heart field (SHF). As the expression of DLG1 is almost ubiquitous, it remains unclear whether the developmental defects in SHF-derived cells arise by intrinsic DLG1 deficiency in the SHF or are induced indirectly through the primary defect in neural crest cell development. Future analysis of tissue specific loss-of-function mouse models is expected to give us further information. (COI:No)

### MS10-5

**Quantitative analysis of tissue and cell dynamics during early heart development**

Yoshihiro Morishita[1], Naofumi Kawahira[1], Naoki Kida[1], Daisuke Otsuka[1] ([1]*RIKEN BDR*)

Vertebrate heart development begins with the formation and looping of a heart tube, and the looping is the first event that shows a clear morphological left-right asymmetry. Although several models of the heart looping have been proposed so far, its morphogenetic mechanisms remain controversial due to a lack of information concerning precise tissue-level deformation and its connection to cellular dynamics, which causes difficulties in evaluating previously proposed models. Against this background, we performed 4-D high-resolution imaging to reconstruct a tissue deformation map, which revealed that, at the tissue-scale, initial heart looping is achieved by left-right asymmetry in the direction of deformation within the myocardial tube. We further identified F-actin-dependent directional cell rearrangement in the right myocardium as a major contributor to left-right asymmetric tissue deformation. Our findings demonstrate that heart looping involves dynamic and intrinsic cellular behaviors within the tubular tissue itself, and provide a significantly different viewpoint from current models that are based on left- right asymmetry of growth/stress at the tube boundaries. (COI:No)

### MS10-6

**Mechanistic logic underlying cellular behaviors that lead to the formation of vascular structure**

Hiroki Kurihara[1] ([1]*Dept Physiol Chem Metab, Grad Sch Med, Univ Tokyo*)

Angiogenesis is a morphogenetic process whereby vascular endothelial cells (ECs) sprout from preexisting vessels to form new branching network. It has been supposed that this process is driven by tip cells activated by angiogenic stimuli such as VEGF, which subsequent stalk cells follow. In contrast to this dichotomous view, we have identified more complex cellular behaviors, in which ECs move with different speeds, forwards and backwards, and change their relative position, resulting in frequent exchange of tip cells. This phenomenon was well simulated by a mathematical model based on the assumption of two-body interactions. Validating it, analysis of individual ECs revealed coordinative linear and rotating movements potentiated upon cell-cell contact, which appeared to enable "cell mixing" during angiogenic morphogenesis. Furthermore, the coordinative linear movement was shown to depend on VE-cadherin. Refined mathematical model well recapitulated morphogenetic processes in the presence and absence of VE-cadherin. These experimental and theoretical approaches are expected to unveil mechanistic logic of cellular behaviors leading to branching morphogenesis of blood vessels. (COI:No)

### Meeting Symposium11

### Neural circuits controling organ functions

(March 29, Mon. 16：30～18：30, Room3)

### MS11-1

#### Immunohistochemical study of molecular components required for ATP-mediated hypoxic signal transduction in the carotid body

Takuya Yokoyama[1], Yoshio Yamamoto[2], Masato Hirakawa[1], Tomoyuki Saino[1] ([1]*Department of Anatomy [Cell Biology], Iwate Medical University, Yahaba, Japan,* [2]*Laboratory of Veterinary Anatomy and Cell Biology, Faculty of Agriculture, Iwate University, Morioka, Japan*)

ATP has shown to be released by exocytosis from carotid body (CB) type I cells when these cells respond to hypoxia, and excites sensory nerve endings via P2X2/P2X3 purinoceptors for signal transduction. We examined the morphology of P2X2-/P2X3-immunoreactive nerve endings and immunoreactivity for vesicular nucleotide transporter (VNUT) in the rat CB. P2X2-/P2X3-immunoreactive nerve endings were associated with clustered type I cells, whereas some endings were sparsely distributed in a few clusters. Nerve endings were hederiform in shape and extended flattened axon terminals. Some nerve endings formed sac- or goblet-like terminal structures, and were attached to tyrosine hydroxylase (TH)-immunoreactive type I cells, whereas few endings were attached to dopamine beta-hydroxylase (DBH)-immunoreactive type I cells. VNUT immunoreactivity was localized in the subpopulation of TH-immunoreactive type I cells associated with P2X3-immunoreactive nerve endings, but not in DBH-immunoreactive cells. The selective localization of VNUT in the subpopulation of CB type I cells attached to sensory nerve endings suggests that these cells release ATP by exocytosis for the hypoxic signal transduction. (COI:No)

### MS11-2

#### Physiological and morphological study of the neuronal mechanism underlying respiratory control by inhibitory neurons in the solitary nucleus

Noriyuki Hama[1], Kotaro Takeda[2], Yasumasa Okada[3], Naohiro Koshiya[4], Hidehiko Koizumi[4], Shigefumi Yokota[5] ([1]*Department of Neural and Muscular Physiology, Shimane University School of Medicine,* [2]*Faculty of Rehabilitation, School of Healthcare, Fujita Health University,* [3]*Clinical Research Center, Murayama Medical Center,* [4]*Cellular and Systems Neurobiology Section, NINDS, NIH,* [5]*Department of Anatomy and Neuroscience, Shimane University School of Medicine*)

Inhibitory neurons in the ventrolateral subnucleus of the solitary tract (vlNST) receive the slowly adapting pulmonary mechanoreceptor afferent and are thought to mediate the Hering-Breuer reflex. Exact function of inhibitory vlNST neurons in control of respiration however is unknown. Here we show that inhibitory vlNST neurons projected to the B?tzinger complex (B?tC) and pre-B?tzinger complex (preB?tC), which generate respiratory rhythm, and rostral part of ventral respiratory group (rVRG), which relays inspiratory drive to the spinal cord. Impairment of vesicular GABA transporter of inhibitory vlNST neurons increased tidal volume (TV) and decreased respiratory rate (RR) by shortening the duration of inspiratory (Ti) and expiratory (Te) phases. In contrast, pharmacogenetic activation of inhibitory vlNST neurons increased RR and decreased TV. Optogenetic activation of inhibitory vlNST neurons during inspiratory phase decreased Ti and Te, thus decreasing RR, but the activation during expiratory phase failed to induce any change in the respiration. These results suggest that inhibitory vlNST neurons inhibit rhythmogenic inspiratory neurons within the preB?tC to regulate RR. (COI:Properly Declared)

### MS11-3

#### Oxytocinergic pathway stimulating brown adipose tissue thermogenesis

Akihiro Fukushima[1], Kazuhiro Nakamura[1] ([1]*Department of Integrative Physiology, Nagoya University Graduate School of Medicine, Japan*)

Endothermic animals maintain their core body temperature by controlling skin vasomotion and brown adipose tissue (BAT) thermogenesis. These thermoregulatory organs are regulated by sympathetic inputs from the hypothalamo-medullary-spinal central pathway.
Oxytocin (OXT), a hypothalamic neuropeptide, contributes to a variety of behaviors and stress responses. Impairment of the oxytocinergic (OXTergic) neural system leads to metabolic hypofunction. However, the neuronal pathway underpinning the link between OXT and metabolism has not been determined. To elucidate the mechanism, we performed neural tract tracing and *in vivo* BAT sympathetic recording in combination with optogenetic stimulation of a specific OXTergic neural pathway, and found that (i) OXT neurons in a dorsocaudal part of the paraventricular hypothalamic nucleus innervate sympathetic premotor neurons in the rostral medullary raphe region (rMR); (ii) the OXTergic input to the rMR stimulates BAT thermogenesis by potentiating glutamatergic transmission onto sympathetic premotor neurons. This OXTergic pathway may underlie etiologies of metabolic disorders, such as Prader-Willi syndrome, and be relevant to emotional responses. (COI:No)

### MS11-4

#### Neural pathway regulating colorectal motility

Yasutake Shimizu[1], Takahiko Shiina[1] ([1]*Department of Basic Veterinary Science, Laboratory of Physiology, The United Graduate School of Veterinary Sciences, Gifu University, Japan*)

Gastrointestinal motility is controlled by the enteric nervous system located in the gastrointestinal wall as the submucosal plexus and myenteric plexus. Although the neural network functions without the central nervous system, the central nervous system also regulates gastrointestinal functions. The central regulating mechanisms of defecation would be important to obtain a pathophysiological understanding of some diseases such as irritable bowel syndrome.
We previously demonstrated that noxious stimuli in the colorectum enhance propulsive colorectal motility through reflex pathways involving the spinal and supraspinal defecation centers. Our findings provide evidence that descending monoaminergic neurons are activated by noxious stimulation to the colorectum, leading to facilitation of colorectal motility. Furthermore, we demonstrated that intracolonic noxious stimulation activates GABAergic and serotoninergic descending neurons in female rats, whereas serotoninergic and dopaminergic neurons are dominantly activated in male rats. In this symposium, we summarize recent findings on neural pathway regulating colorectal motility. (COI:No)

### Meeting Symposium12

### Clocks in Anatomy and Physiology

(March 29, Mon. 16：30～18：30, Room5)

### MS12-1
**Light affects behavioral despair involving the clock gene Period 1**

Urs Albrecht (*Department of Biology, University of Fribourg, Switzerland*)

Light at night has strong effects on physiology and behavior of mammals. It affects mood in humans, which is exploited as light therapy, and has been shown to reset the circadian clock in the suprachiasmatic nuclei (SCN). This resetting is paramount to align physiological and biochemical timing to the environmental light-dark cycle. Here we provide evidence that light affects mood-related behaviors also in mice by activating the clock gene Period1 (Per1) in the lateral habenula (LHb), a brain region known to modulate mood-related behaviors. A light pulse given at ZT22 to wild type mice caused profound changes of gene expression in the mesolimbic dopaminergic system including the nucleus accumbens (NAc). Sensory perception of smell and G-protein coupled receptor signaling was affected the most in this brain region. Interestingly, most of these genes were not affected in Per1 knock-out animals, indicating that induction of Per1 by light serves as a filter for light-mediated gene induction in the brain. Taken together we show that light affects mood-related behavior in mice at least in part via induction of Per1 in the LHb with consequences on signaling mechanisms in the mesolimbic dopaminergic system.

### MS12-2
**Regulation of behavioral rhythm and locomotor activity by light-inducible components of the circadian clock in zebrafish**

Jun Hirayama[1] ([1]*Department of Clinical Engineering, Faculty of Health Sciences, Komatsu University*)

The zebrafish (*Danio rerio*) is an attractive diurnal animal model for the study of vertebrate circadian clocks. In particular, the formation of behavioral rhythms in zebrafish is a useful experimental system to analyze light-dependent regulation of the circadian clock *in vivo*. Previous studies have identified acute light-inducible clock genes, namely zebrafish *Period2* (*zPer2*) and *Cryptochrome1a* (*zCry1a*). To address the physiological roles of zCRY1a and zPER2, *zCry1a* and *zPer2* knockout (KO) zebrafish have been engineered. It was found that zPER2 and zCRY1a synchronize cellular clocks in a light-dependent manner to form the behavioral rhythms of zebrafish, and identified zCRY2a as a third light-induced cellular clock synchronizer that contributes to behavioral rhythm formation in zebrafish. In addition, unforeseen roles for zPER2 and zCRY1a in regulating the total level of locomotor activity was uncovered. Components of the circadian clock regulate both behavioral rhythms and locomotor activity, which likely functions to maximize physiological efficiency. (COI:No)

### MS12-3
**Regulatory mechanism underlying circadian rhythm of intraocular pressure**

Keisuke Ikegami[1], Yasufumi Shigeyoshi[2], Satoru Masubuchi[1] ([1]*Dept Physiol, Grad Sch Med, Aichi Med Univ, Aichi, Japan,* [2]*Dept Anato Neurobiol, Kindai Univ Facul Med, Osaka, Japan*)

Elevated intraocular pressure (IOP) can cause glaucoma. The circadian rhythm of IOP depends on the dynamics of the aqueous humor and is synchronized with the circadian-rhythm pacemaker, the suprachiasmatic nucleus (SCN). Normal-tension glaucoma, ~70% of glaucoma in Japan, shows abnormal IOP rhythm. The SCN resets peripheral clocks mainly via sympathetic nerves or adrenal glucocorticoids (GC). However, the detailed mechanisms underlying IOP rhythmicity remain unclear. We have investigated this regulatory pathway in mice. Adrenalectomy and superior cervical ganglionectomy disrupted rhythms of IOP and circadian clock in the iris/ciliary body culture. Instillation of GC and norepinephrine rescued IOP rhythm, indicating the dual rhythm transmission pathway. Further, the $\beta$ 2-adrenergic receptor, GC receptor, and clock proteins (Bmal1 and Per1) were strongly expressed at the non-pigmented epithelia of the ciliary body producing aqueous humor. However, retina- and ciliary epithelium-specific *Bmal1* knock-out mice maintained their IOP rhythm, indicating direct entrainment of the IOP rhythm by the SCN, but not the ciliary clock. This finding may be useful in the chronotherapy for glaucoma. (COI:No)

### MS12-4
**A flexible structure of the biological clock and adjustment to environment**

Ken-ichi Honma[1] ([1]*Hokkaido Uni. Grad Sch Med Professor Emeritus*)

The biological clock consists of the central clock located in the suprachiasmatic nucleus (SCN) and the peripheral clocks in almost all tissues and organs outside the SCN. The central clock entrains to light-dark (LD) cycles and sends the circadian signals to the peripheral. The peripheral clocks drive the circadian rhythms in tissue or organ specific functions and entrain to non-photic time cues as well as the SCN circadian signal. The principal role of biological clock is to establish stable phase relationships with environmental cycles in order to guarantee the best performance of body function at a certain time of day. Except for the equator, the times of dawn and dusk are changing throughout a year, resulting in the seasonal changes in day length. A stable entrainment to ever-changing LD cycles is established by two mutually coupled oscillators (E, M oscillators) in the SCN. The E oscillator drives the activity onset and M oscillator the activity end. Taking advantage of circadian clock gene expression rhythm in mice, we identified the sites of E and M oscillators in the rostral and caudal SCN shell, respectively. They are also involved in re-entrainment of shifted LD cycle. (COI:No)

### MS12-5
**Multiple oscillators in the suprachiasmatic nucleus, the center of the mammalian circadian clock**

Yasufumi Shigeyoshi[1] ([1]*Anat Neurobiol. Med. Kindai*)

In mammals, the central clock is located in the hypothalamic suprachiasmatic nucleus (SCN). The SCN has some functionally distinct regions and the whole arrangement of the functional units has not been fully clarified. First, the SCN is divided into a ventrolateral region (VLSCN) and dorsomedial region (DMSCN). The VLSCN receives a direct projection from the retina while the DMSCN does not. Further, the DMSCN is not a uniform oscillator. The medial region of the DMSCN has a small region on the medial side in which neurons which shows neurons showing short-period accumulate and lateral part where long-period oscillator neurons showing longer periods. The two regions possibly produce a phase wave in the SCN that propagates from medial to lateral region. On the other hand, Yoshikawa et al. (Yoshikawa et al. Sci. Rep. 2017) reported a morning clock localized in the caudal part and an evening clock localized in the rostral region of the DMSCN. We are currently trying to clarify the overall picture of regional oscillators in the SCN. In the symposium, we will present our recent understanding on the multiple oscillators in the SCN. (COI:No)

### Meeting Symposium13

### Spatial distribution of synapse and brain function

(March 30, Tue. 9：00〜11：00, Room2)

### MS13-1

**Dendritic synapse geometry optimizes binaural computation in sound localization circuit**

Hiroshi Kuba[1] ([1]*Dept Cell Physiol, Grad Sch Med, Nagoya Univ*)

Synaptic clustering is a way of neurons to overcome the attenuation of signals at dendrites. Using glutamate uncaging and immunohistochemistry together with simulation, we show in avian binaural integrators that clustering of synapses at distal dendrites promotes the dendritic attenuation via facilitating sublinear integration, but improves the binaural computation at the soma for intense input. The extent of this clustering differed according to dendritic length and frequency tuning of neurons, being prominent for long dendrites and low-frequency tuning, which ensured binaural spatial hearing for wide intensity and frequency ranges. These results indicated that synaptic clustering is a mechanism of expanding the dynamic range of neuronal computation in manners dependent on dendritic morphology and input frequency, thereby strengthening the computational power of the auditory system. (COI:No)

### MS13-2

**Dendritic compartment-specific regulation of spine density during adolescent cortical development**

Takeshi Imai[1] ([1]*Grad Sch Med Sci, Kyushu Univ*)

It is generally believed that the number of dendritic spines increases during childhood, and then declines during adolescence. However, their distribution on a whole-neuron scale has not been fully established. Here we performed comprehensive high-resolution mapping of dendritic spines in cortical pyramidal neurons in mice using volumetric super-resolution imaging and SeeDB2. In this study, we focused on thick-tufted layer 5 neurons. Spine density was relatively uniform in basal dendrites in these neurons. However, the spine density is highly biased along the middle compartment of long apical dendrites (a spine density "hotspot"), demonstrating ~10-fold accumulations. In basal dendrites, spine density moderately declined during the adolescence stage. In apical dendrites, however, the spine density continued to increase specifically at the spine density hotspot. Accumulation of dendritic spines to the hotspot was impaired by genetic manipulation of genes related to neuropsychiatric diseases. Thus, the spine "accumulation" to the hotspot is a hallmark of cortical circuit maturation during adolescence. (COI:No)

### MS13-3

**Quantitative analysis of the synaptic inputs on various neuron subtypes in rat cortex**

Yoshiyuki Kubota[1,2], Yasuo Kawaguchi[1,3] ([1]*Division of Cerebral Circuitry, National Institute for Physiological Sciences,* [2]*Dept Phys Sci, Sch Life Sci, SOKENDAI, Okazaki, Japan,* [3]*Brain Sci Inst, Tamagawa, Univ, Machida, Tokyo, Japan*)

The neocortex has numerous classes of pyramidal and nonpyramidal cells. Those are distinct in firing, morphological, and chemical characteristics. Morphological studies show distinct axonal projection patterns from these different classes of neurons, suggesting selective synapse formation onto postsynaptic targets. However, the synaptic inputs to individual cell subtypes have not been studied thoroughly. In order to reveal rules governing selective synapse formation onto cortical neuron subtypes, crossed-corticostriatal (IT)layer 5 pyramidal cell, four defined nonpyramidal neuron subtypes: fast spiking basket cell, Martinotti cell, double bouquet cell and large basket cell, were investigated. Excitatory and inhibitory synapses onto dendrites and soma were identified by postembedding GABA immunohistochemistry in ultra thin sections in which postsynaptic elements were revealed by immunohistochemical staining. We subsequently reconstructed somata and dendrites with identified GABAergic/non-GABAergic synaptic inputs three-dimensionally and quantified the synaptic input structure on each neuron subtypes. These results help to understand cortical microcircuit functional architecture.

### MS13-4

**Simulation of spatiotemporal signaling dynamics for synaptic plasticity by using technologies for EM connectomics**

Hidetoshi Urakubo[1] ([1]*NIPS, Div Cerebral Circuit*)

Synaptic plasticity in the brain, a basis of learning and memory, occurs as a result of dynamics of intracellular signaling in a particular spatial domain, i.e., a postsynaptic spine. The characteristic shape of the spine functions as a separator of synaptic signaling from other synapses, which realizes the site-specific occurrence of synaptic plasticity (synaptic specificity). On the other hand, this spatial separation is imperfect, synaptic plasticity also occurs in cooperation with neighboring synapses (synaptic cooperativity). We are conducting computer simulation on the working hypothesis that the imperfect spatial separation depends on the morphological characteristics of spines, and the brain region-dependent synaptic cooperativity may come from region-dependent spine shapes. We target the spines in the hippocampal CA1, striatum, and neocortex, and are examining how spine shapes affects intracellular signaling. We would like to report the current status of this project. The shapes of spines are taken from three-dimensional electron microscopic (EM) images, which is based on the technologies for EM connectomics. We would also introduce such technologies. (COI:No)

### MS13-5

**Extra-large spines distort neuronal computation in synaptic disorders**

Akiko Hayashi-Takagi[1] ([1]*RIKEN, CBS*)

The spatiotemporal organization of neuronal firing is crucial for information processing, but how thousands of inputs to the dendritic spines drive the firing remains a central question in neuroscience. Despite the fact that the distribution of spine sizes, an index of synaptic weight, is strongly skewed with a heavy tail, the (patho)physiological significance of large spines remains entirely unknown. Here, we found that extra-large (XL) spines (more than three standard deviations from the average) supralinearly boosted the firing triggered by NMDA spikes within these spines. The resulting synaptic amplification in a few XL spines was sufficient to drive neuronal firing in the absence of the normally required $Ca^{2+}$ spike. Interestingly, mice with knockdown of DISC1, a molecule implicated in psychiatric conditions, exhibited ten-fold more XL spines and markedly increased firing. We experimentally and theoretically observed that XL spines negatively correlated with working memory, which can contribute to psychiatric pathophysiology. (COI:No)

### Meeting Symposium14

### Inter-organ communication networks maintain organismal homeostasis

(March 30, Tue. 9：00～11：00, Room3)

### MS14-1
**Role of Brain-liver cross-talk in glucose homeostasis and its deterioration**

Hiroshi Inoue[1] ([1]*Institute for Frontier Science Initiative, Kanazawa University, Japan*)

Brain, especially hypothalamus, regulates hepatic glucose metabolism via endocrine- and neuronal-organ cross-talk. The vagal nerve plays the central role in both of these cross-talk. The vagal nerve regulates pancreatic endocrine function, which have an enormous influence on hepatic glucose metabolism, through the muscarinic cholinergic action. It also controls hepatic glucose metabolism through the nicotinic action to the Kupffer cells. Indeed, we have reported that hypothalamic nutritional sensing induces acute IL-6 secretion from the Kuppfer cells via alpha 7 nicotinic acetylcholine receptor (A7nAchR), resulting in the suppression of hepatic glucose production by IL-6/STAT3-dependent suppression of gluconeogenic genes.
Obesity and insulin-resistance impairs the vagal-mediated brain-liver cross-talk. In obese mice, the vagal nerve dysregulates the pancreatic endocrine function and fails to suppress hepatic gluconeogenic genes and HGP, in spite of the smoldering activation of Kupffer cells. The impediment of brain-liver cross-talk would be related to impaired glucose homeostasis in obesity and insulin-resistance. (COI:No)

### MS14-2
**Gut-liver axis and obesity-associated hepatocellular carcinoma**

Naoko Ohtani[1] ([1]*Dept Pathophysiology, Grad Sch Med, Osaka City Univ*)

Obesity is known to be a risk factor for several types of cancer including hepatocellular carcinoma (HCC). We previously identified that the enterohepatic circulation of deoxycholic acid (DCA), a gut microbial metabolite, promotes cellular senescence and the senescence-associated secretory phenotype (SASP) of the hepatic stellate cells (HSCs), a phenotype that senescent cells secrete a variety of inflammatory cytokines, chemokines and proteases and so on. The SASP factors are associated with creating a cancer promoting tumor microenvironment, suggesting that DCA is one of the crucial factors to accelerate obesity associated liver cancer. We reported that IL-1beta, a SASP factor and an upstream regulator of the cytokine cascade, was secreted from senescent HSCs to promote HCC development. However, downstream players of IL-1beta remained to be elucidated. Recently, we identified a specific cytokine, which is a down-stream target of IL-1beta, plays an important role in obesity-associated HCC development. Moreover, we uncovered the activation mechanism of this cytokine triggered by gut microbial component, lipoteichoic acid (LTA) that was absorbed and translocated to the liver. (COI:No)

### MS14-3
**The organic networks for hepatic lipid homeostasis.**

Tsutomu Matsubara[1], Kazuo Ikeda[1] ([1]*Department of Anatomy and Regenerative Biology, Osaka City University Graduate School of Medicine*)

The liver is involved in a variety of metabolic processes including decomposition of fatty acids. In Japan, non-alcoholic steatohepatitis (NASH) is increasing and can lead to liver cirrhosis and liver cancer. Elucidation of the molecular pathology of NASH, which progresses to fat accumulation, inflammation, and fibrosis in the liver, is expected to lead not only to the establishment of NASH therapy, but also to the prevention of liver cirrhosis and liver cancer.
The liver is known to link networks of multiple organs, such as the brain, intestine, kidney, and adipose tissue, to maintain hepatic lipid homeostasis. In this session, I will introduce the mechanism by which the liver, adipose tissue, and intestine regulate the hepatic fat level in the NASH, presenting the molecular mechanism of hepatic fat accumulation via the peroxisome proliferator-activated receptor $\gamma$-fat specific protein 27 (Fsp27, also known as cell death-inducing DFFA-like effector C) cascade, lipid regulation between liver and adipose tissue revealed in adipose tissue-specific Fsp27-deficient mice, and the fluctuation of hepatic fat accumulation mediated by a stress-elevated bile acid, $\beta$-muricholic acid.
(COI:No)

### MS14-4
**Macrophages and organ crosstalk in homeostasis and multimorbidity**

Ichiro Manabe[1] ([1]*Department of Disease Biology and Molecular Medicine, Chiba University Graduate School of Medicine*)

Multimorbidity is a growing medical challenge in the aging of society. Heart failure is one of the key diseases of multimorbidity and causes multiple comorbidities, including chronic kidney disease (CDK), frailty, cachexia, and cancer. For instance, risks of heart failure and CKD are closely associated. We showed that cardiac macrophages are regulated by the heart-brain-kidney organ network and play a vital role in the cardiac adaptive response to pressure overload. Pressure overload in the heart activates renal collecting duct (CD) epithelial cells via sympathetic nerves. Within the kidneys, activated communication between CD cells, tissue macrophages, and endothelial cells leads to the secretion of CSF2, which in turn stimulates cardiac-resident Ly6C$^{lo}$ macrophages essential for the myocardial adaptive response to pressure overload. We also found that cardiac resident macrophages crucially contribute to the multiple physiological processes in the heart. They maintain electrical conduction, energy metabolism, and mechanical stress response. Accordingly, cardiac macrophage dysfunction would lead to cardiac diseases, such as heart failure, which in turn may promote multimorbidity. (COI:No)

### Meeting Symposium15

### Mechanisms in the maintenance of cardiac homeostasis

(March 30, Tue. 9：00～11：00, Room5)

### MS15-1

**Homeostatic maintenance in human iPS cell-derived cardiomyocytes**

Kazuho Sakamoto[1], Junko Kurokawa[1] ([1]*Department of Bio-Informational Pharmacology, School of Pharmaceutical Sciences, University of Shizuoka*)

Human iPS cell-derived cardiomyocytes (hiPSC-CMs) are conceptually promising as an unlimited source of human cardiomyocytes. However, hiPSC-CMs generated using current methods are structurally and physiologically immature, resembling fetal cardiomyocytes. Field electric stimulation is an engineering method to improve the maturity of hiPSC-CMs. Our histological analysis revealed that electrical stimulation of the cells enhanced sarcomere formation. Our motion field imaging revealed that the electrical stimulation reduced spontaneous activity of hiPSC-CMs. Our fluorometric investigation showed that the electrical stimulation enhanced the intracellular sodium concentration. A mathematical model of hiPSC-CM used to assess the relationship between the enhancement of sodium concentration and spontaneous activity. We would like to discuss about molecular mechanisms of hiPSC-CM maturation, cardiac development, and homeostasis. (AMED 19mk0104117, ExCELLs 19-204, JSPS KAKENHI JP19H03380) (COI:No)

### MS15-2

**Developmental changes in the excitation-contraction mechanisms of the myocardium**

Shogo Hamaguchi[1], Iyuki Namekata[1], Hikaru Tanaka[1] ([1]*Department of Pharmacology, Toho University Faculty of Pharmaceutical Sciences*)

The excitation-contraction mechanism of the myocardium varies among different developmental stages, animal species, and pathological conditions. In our laboratory, to obtain a basis for a comprehensive understanding of the excitation-contraction mechanism of the myocardium, I have been studying isolated myocardial tissue and cellular preparations with contractile force and action potential measurements, and fluorescence imaging of cell morphology and intracellular calcium dynamics. In this symposium, I will present a review of the changes during postnatal development focusing three points: ① cellular morphology and calcium handling, ② action potential configuration and response to autonomic stimuli, and ③ energy metabolism. Through such researches, I am aiming to understanding the relationships between the excitation-contraction mechanisms and pharmacological properties of the myocardium. (COI:No)

### MS15-3

**Role of mitochondria quality control in the maintenance of cardiac robustness**

Motohiro Nishida[1,2,3] ([1]*Dept. Physiol., Grad. Sch. Pharm., Sci., Kyushu Univ.*, [2]*Div. Cardiocirc. Signal., NIPS/ExCELLS*, [3]*SOKENDAI*)

Mitochondria are dynamic organelles that continuously undergo fission and fusion, which are necessary for maintaining bioenergetic homeostasis and robustness in heart. Mitochondrial quality is precisely controlled by redox-active Cys-containing GTPase, Drp1. We revealed that redox-active Cys624 on Drp1 is basically polysulfidated. Exposure of cardiomyocytes to oxidative or electrophilic stress causes depolysulfidation of Drp1, which leads to Drp1-dependent mitochondrial hyperfission as well as cardiac vulnerability to hemodynamic load in mouse hearts. Reactive sulfur species such as Cys persulfides that are produced through mitochondria-localized Cys tRNA synthetase (CARS2) preferentially contribute to electrophile metabolism. Supplementation of sulfur by exogenous treatment with NaHS completely abolished electrophile-induced sulfur deprivation of Drp1 protein as well as exacerbation of myocardial injury induced by mechanical stress. These results strongly suggest that formation of Drp1 Cys624 polysulfidation negatively regulates electrophile-mediated mitochondrial hyperfission and cardiac stress resistance against environmental stress. (COI:No)

### MS15-4

**Sex differences in the homeostatic maintenance of cardiomyocytes**

Junko Kurokawa[1], Kazuho Sakamoto[1] ([1]*School of Pharmaceutical Sciences, University of Shizuoka*)

Cardiovascular diseases show significant sex differences. Sex hormonal regulation in cardiac ion channels accounts for functional responses to sympathetic nervous system stimulation, which show sex differences in susceptibility of arrhythmias associated with QT prolongation. Although women have a greater risk than men in long QT syndrome, the sex difference is evident when compared with adult women at follicular phase, suggesting protective effects of androgen and progesterone. Although transcriptional regulation has been reported to prolong QT intervals, we have found that androgen and progesterone ($P_4$) acutely shorten QT/APD via non-genomic pathways of cardiac sex hormone receptors. Both sex hormones produced NO which up-regulates the $I_{Ks}$ channel currents and suppressed L-type $Ca^{2+}$ channel currents ($I_{Ca,L}$). The $I_{Ca,L}$ suppression by $P_4$ is cAMP-dependent, suggesting a $P_4$-specific localized cross-talk of cAMP/NO signaling. These data may explain a sex difference in QT intervals and arrhythmia risk, and can be a clue to understand physiological mechanism to maintain electrical homeostasis of cardiomyocytes. (COI:No)

### MS15-5

**Post-transcriptional regulation of cardiac excitation**

Katsushige Ono[1] ([1]*Oita University School of Medicine, Department of Pathophy siology*)

Excitability is a basic characteristic of cardiomyocytes, which is precisely governed by ion channel and ion transporter activities modulated by various factors. Recent studies have led to discovery of microRNAs (miRNAs) as a new player in the cardiac excitability. Indeed, evidence has emerged indicating the crucial role of miRNAs in controlling cardiac excitability by regulating expression of ion channel genes at the post-transcriptional level. And several key pathways related to miRNAs, such as $Ca^{2+}$-dependent signaling pathways, inflammatory, apoptotic and cycle pathways have been found, which indicates that miRNAs are likely to be the therapeutic target for atrial fibrillation. This review investigation is to explain a particular ionic remodeling process in arrhythmias or atrial fibrillation with the corresponding deregulated miRNAs under that pathological condition. (COI:No)

### Meeting Symposium16

### Mitochondrial disease: update

(March 30, Tue. 9：00～11：00, Room6)

### MS16-1

**Development of mutant mtDNA-targeted TALENs and their application to iPSC-based mitochondrial disease model.**

Naoki Yahata[1] ([1]*Dept Anatomy I, Sch Med, Fujita Health Univ*)

Various mitochondrial diseases, including mitochondrial encephalopathy, lactic acidosis, and stroke-like episodes (MELAS), are associated with heteroplasmic mutations in mitochondrial DNA (mtDNA). We generated iPSCs from a patient with MELAS for disease modeling. The patient's dermal fibroblasts harboring G13513A mutant mtDNA were reprogrammed, and we established iPSC clones with and without mutant mtDNA, respectively. We also developed platinum TALENs, which preferentially cleaved the G13513A mutant mtDNA (G13513A-mpTALEN), to manipulate mtDNA heteroplasmy. MELAS-iPSCs with a high proportion of G13513A mutant mtDNA showed unusual properties of spontaneous, embryoid body-mediated differentiation *in vitro*, which was relieved by decreasing the heteroplasmy level with G13513A-mpTALEN. Additionally, drug-inducible, myogenic differentiation 1(MYOD)-transfected MELAS-iPSCs (MyoD-iPSCs) efficiently differentiated into myosin heavy chain-positive myocytes, with or without mutant mtDNA. Hence, heteroplasmic MyoD-iPSCs controlled by optimized mpTALENs will contribute to a detailed analysis of the relationship between mutation load and cellular phenotypes in disease modeling. (COI:No)

### MS16-2

**The role of tRNA Modifications in mitochondrial disease**

Fanyan Wei[1], Kazuhito Tomizawa[2] ([1]*Tohoku Univ. IDAC,* [2]*Kumamoto Univ. Dept. Mol. Phsyiol*)

A subset of mt-tRNAs contain unique modifications derived from taurine at position 34U. Importantly, mitochondrial disease (MELAS and MERRF) patients exhibit a decrease in taurine modification in mt-tRNA$^{Leu(UUR)}$ and mt-tRNA$^{Lys}$, respectively. To elucidate the molecular function of taurine modification and its relevance with mitochondrial disease, we have established mouse model with deficiency of Mto1, the taurine modification enzyme. Deficiency of Mto1 completely abolished taurine modification in mt-tRNAs and severely impaired mitochondrial translation, leading to a marked OXPHOS dysfunction and abnormal membrane organization. Moreover, the catastrophic mitochondrial dysfunction impaired trafficking of nuclear DNA-encoded mitochondrial proteins into mitochondria. The mistargeted proteins were aggregated and misfolded in the cytosol, which induced cytotoxic unfolded protein response. In summary, our results demonstrate that taurine-modification is essential for mitochondrial functions, and the deficiency of taurine-modification is the primary cause of the development of mitochondrial disease through defective proteostasis. (COI:No)

### MS16-3

**Reverse genetic studies on mitochondrial DNA-based diseases in mice**

Kazuto Nakada[1] ([1]*Faculty of Eviron Sci, Univ of Tsukuba* )

Mammalian mtDNA encodes 13 polypeptides which are essential subunits for electron transport complexes and 22 tRNAs and 2 rRNAs which are necessary for the translation of these 13 polypeptides. The predominant accumulation of pathogenic mutant mtDNAs and the resultant mitochondrial respiration defects are manifest as a variety of mitochondrial diseases. At present, accumulation of pathogenic mutant mtDNAs is extended to patient with diabetes, neurodegenerative diseases, cancers, and further to aged subjects. Generation of model animals carrying mutant mtDNAs is useful for understanding the pathophysiological mechanisms of the multiple mtDNA-based disorders. Using the cell fusion techniques, we have succeeded in generating mice with pathogenic mutant mtDNAs, named "mito-mice", by the introduction of mitochondria carrying pathogenic mutant mtDNAs into mouse zygotes and mouse embryonic stem cells. In this presentation, I would like to show multiple phenotypes in the mito-mice and also discuss regulation of pathogeneses in mtDNA-based diseases. (COI:No)

### MS16-4

**Unraveling the pathophysiology of mitochondrial respiratory chain disorders by comprehensive genome analysis**

Yasushi Okazaki[1] ([1]*Intractable Dis Res Ctr, Grad Sch Med, Juntendo Univ*)

Mitochondrial disorders have the highest incidence among inborn errors of metabolism and are characterized by biochemical respiratory chain complex deficiencies. It occurs at a rate of 1 in 5,000~7,500 births, and has phenotypic and genetic heterogeneity. Mutations in mtDNA and about 1,500 nuclear encoded mitochondrial proteins may cause mitochondrial disorder. Over 350 genes that lead to mitochondrial disorder have been reported to date. Exact genetic diagnosis for patients, however, still remains largely unimplemented. We performed over 700 targeted and 700 WES and 160 WGS for patients with childhood-onset mitochondrial disorders. We have so far identified and reported mitochondria-related genes (*MRPS23, QRSL1, SLC25A26, C1QBP, IARS, ATAD3 del, COQ4, GTPBP3, ECHS1, TOP3A, PTCD3, NDUFA8, ATAD3 dup*, etc) as novel causative genes. Our approaches enhance the ability to identify pathogenic gene mutations in patients with biochemically defined mitochondrial disorders. We will report the molecular pathophysiology and discuss about the significance and limitations of genetic diagnosis of the mitochondrial diseases. (COI:No)

### MS16-5

**Clinical and molecular basis of mitochondrilal disease and mitochondrial medicine**

Kei Murayama[1] ([1]*Department of Metabolism, Chiba Children's Hospital*)

Mitochondrial diseases are inherited metabolic diseases based on disorders of energy production. The expansion of exome analyses has led to the discovery of many pathogenic nuclear genes associated with these diseases, and research into the pathogenesis of metabolic diseases has progressed. Our comprehensive gene analyses have led to the discovery of several novel genes for mitochondrial diseases. Given the varied pathogeneses, treatments for mitochondrial diseases should be specifically tailored to the mutated gene. Treatment and management approaches, including prenatal diagnoses, specifically tailored to the various phenotypes and pathologies of mitochondrial diseases are expected to become increasingly available. (COI:No)

### Meeting Symposium17

### Regulatory factors and functions for developmental neurotoxicity

(March 30, Tue. 14：20～16：20, Room6)

### MS17-1

**Development-progressive neurotoxicity regulated neuroinflammation with prenatal chemical exposure on the rat.**

Sachiko Yoshida[1], Yoko Nomura[2], Yasunari Kanda[3] ([1]*Department of Applied Chemistry and Life Science, Toyohashi University of Technology,* [2]*Queens College, the City University of New York,* [3]*Division of Pharmacology, National Institute of Health Sciences*)

Chemical exposure in utero has potential effects on developmental neurotoxicity (DNT). Lipopolysaccharide (LPS), a well-known pro-inflammatory factor, may cause mental disorders. LPS-treated pups exposed to 100 μg / kg in utero showed increased inflammatory cytokine expression such as TNF alfa and IL-1beta compared to control rats at postnatal day 2 (P2); however, the decrease of the Purkinje cells and an increase of microglia were observed at P14, not at P7. Neurotoxicity of LPS would progress during the second week after birth. Glyphosate (GLY) is the main compound of a broadly applied herbicide that inhibits a shikimate pathway enzyme, not existing in animals; however, GLY is frequently questioned for its neurodevelopmental safety. Exposure of 250 mg/kg- GLY to pregnant rats acutely or chronically led to Purkinje cell death and microglial activation in the offspring at P14. The expression of IL-1 beta was increased at P5 transiently, whereas iNOS expression was increased after P16 in the GLY-exposed cerebellar cortex. We suggest development-progressive neurotoxicity would be induced with chemicals and might be able to recover in the early stage. (COI:No)

### MS17-2

**Multifunctional GABA and neuro-psychiatric diseases**

Chitoshi Takayama[1] ([1]*Dept. Mol. Anat., Sch. Med., U Ryukyus*)

In the mature central nervous system (CNS), γ-aminobutyric acid (GABA) acts as an inhibitory neurotransmitter by mediating depolarization of membrane potential. On the other hand, it mediates depolarization in the immature CNS. This difference in GABAergic action depends on the intracellular chloride ion concentration $[Cl^-]_i$, primarily regulated by sodium potassium chloride co-transporter 2 (KCC2). In the immature CNS, KCC2 expression is low, $[Cl^-]_i$ is high, and GABA induces the influx of $Cl^-$. During development, KCC2 expression markedly increased, $[Cl^-]_i$ declines, and GABAergic action shifts from excitation to inhibition. Previous studies demonstrated that abnormalities of GABAergic transmission are related to various psychiatric diseases, such as schizophrenia, depression, autistic spectrum diseases, sleeplessness, and so on. Furthermore, GABA may prevent inflammation and neuronal cell death, and its dysfunction may be relate to the neurodegenerative diseases in the CNS. In this session, I will feature above multi functions of GABAergic inhibition and excitation under the control of KCC2 function. (COI:No)

### MS17-3

**Evaluation of chemical toxicity by using GluA2 expression in neurons as an index**

Yaichiro Kotake[1] ([1]*Grad Sch Biomed Health Sci, Hiroshima Univ*)

In assessing the toxicity of countless chemicals in the world, it is important to find indices of toxicity. In the process of studying the neurotoxicity of organotin, we found that exposure of primary cultured cortical neurons to 20 nM tributyltin at concentrations close to those prevalent in the brain reduced the expression of the AMPA-type glutamate receptor subunit GluA2 and rendered the neurons vulnerable to GluA2. It was found that reduced GluA2 expression increases the calcium-permeable AMPA receptor, which does not contain GluA2, making neurons more vulnerable and prone to cell death. In addition, in vivo, GluA2-reduced substances, including tributyltin, were found to cause neuronal vulnerability. These results suggest that the reduction of GluA2 expression may explain part of the neurotoxicity of these chemicals, which has yet to be elucidated. In the future, it would be expected to use neurotoxicity markers such as GluA2 reduction for screening of neurotoxic chemicals. (COI:No)

### MS17-4

**Development of neurotoxicity assessment using human iPS cell technology**

Yasunari Kanda[1] ([1]*Division of Pharmacology, National Institute of Health Sciences [NIHS]*)

Animal models have contribute to our understanding of physiology, mechanisms of diseases, and toxicity. However, the predictivity of adverse effects is not enough during drug development and human health from environmental chemicals. Therefore, human-based in vitro models are expected to evaluate safety and efficacy of drugs/chemicals. Human iPS cell technology provide such a platform with the unique advantage of reproducing the human relevant in vitro models. Here we present the framework of iPS cell-based assays regarding neurotoxicity studies and drug safety assessment toward global standardization. Neurotoxicity can be divided into structural and functional aspects and we have collaborated with OECD developmental neurotoxicity group as well as international consortium HESI (NeuTox subteam) to make new in vitro models for safety and efficacy evaluation. In the symposium, I would like to share our research activities and discuss future perspectives toward in vitro neurotoxicity method using iPS cell technology.(COI:No)

### Symposia

### Symposium1

### Pleiotropic functions of polyamines

(March 28, Sun. 9：00～11：00, Room3)

### SY1-1

**Chemical basis of high-affinity polyamine interactions in the inner cavity of Kir channels**

Harley Kurata[1] (*[1]Dept Pharmacol, Alberta Diabetes Institute, University of Alberta, Alberta, Canada*)

Steep voltage-dependence of inwardly-rectifying potassium (Kir) channels arises from blockade by intracellular polyamines. These endogenous blocking particles reach a stable binding site in the channel inner cavity, and high-affinity binding depends on carboxylate side chains (the 'rectification controller') in this region. However, there is uncertainty regarding the pKa/protonation state of carboxylate side chains in the cation-selective Kir channel pore. We have investigated how introduction of positive charges with different hydrogen bonding propensity (by modification of Kir2.1[I176C] influences polyamine interaction with the rectification controller. We observed that strong hydrogen bond donors (eg. MTSEA) strongly disrupted spermine block, while bulkier reagents incapable of hydrogen bonding (eg. MTSET) did not alter spermine affinity, but slowed unbinding kinetics. We have also used a hydrogen-bonding deficient spermine analog (Me10-spermine), and heavy water (D2O) to test the nature of chemical interactions between amines and carboxylates in the Kir inner cavity. Overall, these findings highlight the importance of close amine-carboxylate interactions for high affinity polyamine block. (COI:No)

### SY1-2

**Polyamine that enhances the surface activity of lung surfactant inflates lung collapse of acute respiratory distress syndrome.**

Makiko Ohkido[1], Yasushi Mio[2], Norifumi Tatsumi[3], Naofumi Kimura[4] (*[1]Dept Mol Biol, Jikei Univ Sch of Med, Tokyo, Japan, [2]Dept Anesthesiol, Jikei Univ Sch of Med, Tokyo, Japan, [3]ept Anat, Jikei Univ Sch of Med, Tokyo, Japan, [4]Dept Pharmacol, Jikei Univ Sch of Med, Tokyo, Japan*)

Worldwide, many patients with acute respiratory distress syndrome (ARDS) are now dying because there are currently no effective therapeutic drugs. Surfactant deactivation, caused by edema in alveoli, is a hallmark of ARDS and results in alveolar collapse. Several exogenous surfactant replacement therapies have been attempted for ARDS, but none have been reported to improve life prognosis. We recently found an endogenous polyamine in extracellular space alveoli, and investigated whether polyamine is involved in respiratory function. In vitro experiments, that mimics ARDS, showed that polyamine had a function of enhancing surface activity of bovine lung surfactant. In the experiment of rat model of ARDS, administration of polyamine to alveoli helped increase the lung compliance and partial pressure of arterial oxygen $PaO_2$. It also helped improve the heterogeneity of aeration of ARDS. The endogenous polyamine, as well as surfactant, should be diluted by edema in alveoli, but polyamine alone replacement to alveoli was working to inflate ARDS lung. These results indicate that polyamine is a key component in respiration and will contribute to therapeutic strategies for ARDS. (COI:No)

### SY1-3

**Localization and physiological function of vesicular polyamine transporter (VPAT)**

Miki Hiasa[1] (*[1]Okayama University Graduate School of Medicine, Dentistry and Pharmaceutical Sciences*)

The polyamines, spermine (Spm) and spermidine (Spd), and their precursor, putrescine play essential roles in cell growth and differentiation in practically all living organisms. In the central nervous system, Spm and Spd act as neuromodulators. Although polyamines are synthesized in neurons and astrocytes, and released into the extracellular space, it remains unclear how and where polyamines are released. Under the circumstances, we focused on the mechanism on vesicular secretion through vesicular polyamine transporter (VPAT), the fourth member of the SLC18 transporter family. VPAT protein is predominantly expressed in the hippocampus and is associated with vesicles in astrocytes and neuron, and actively transport Spm and Spd coupled to the exchange of $H^+$. We showed that VPAT is responsible for vesicular storage and secretion of polyamines in central nervous system and immune system. In mast cells, released polyamines through VPAT regulated histamine release. Thus, identification of VPAT localization and function has helped to determine the novel physiological functions of polyamines. (COI:No)

### SY1-4

**Role of polyamine in skeletal muscle**

Toshiko Yamazawa[1] (*[1]Department of Molecular Physiology, The Jikei University School of Medicine*)

Polyamines are considered to be essential for growth factors in all virtually cells. The polyamines putrescine, spermidine and spermine are low molecular weight organic polycations, well known as mediators involved in cell homeostasis. The proposed roles of polyamines are the functioning of ion channels, nucleic acid packaging, signal transduction, cell proliferation, and differentiation, as well as regulation of gene expression. In skeletal muscle, regulation of polyamine levels is associated with muscle hypertrophy and/or atrophy, but the mechanisms are not well-defined. Here, we studied how polyamines may affect the proliferation and/or differentiation of murine myoblast progenitor C2C12 cell line. We further examined the effects of polyamine administration in C57BL6 mice that underwent sciatic nerve amputation of the hindlimb. Therefore, our study demonstrates that polyamines may play an important role in regulating the skeletal muscle homeostasis. (COI:No)

### Symposium2

### Comprehensive understanding of the shape and function of "The Thrifty Phenotype"

(March 28, Sun. 9：00～11：00, Room8)

### SY2-1

**Abnormal negative feedback of glucocorticoids caused by embryonic malnutrition and changes in the body-tendency due to the higher glucocorticoids**

Takahiro Nemoto[1] (*[1]Dept. Physiology, Nippon Medical School*)

Two epidemiological studies of famine in Leningrad and the Netherlands have led to the idea that a mismatch between acquired body-tendency and environment is a risk factor for the development of the disease. Fetal survivors exposed to long-term undernourished Leningrad famine have acquired a "thrifty phenotype" that adapts the postnatal environment. It is considered that the fetal survivors exposed to short-term undernourished Dutch famine has acquired a "disease-prone body-tendency" that easily induces a mismatch with the environment. These hypotheses have come to be called PARs (Predictive adaptive responses) / Mismatch hypotheses and are now evolving into the DOHaD (Developmental Origins of Health and Disease) theory. In fact, subjects with Leningrad famine did not have an increased incidence of non-communicable chronic diseases. We generated a new model of full-term low-carbohydrate calorie restriction during the embryonic period has higher blood corticosterone after a high-fat diet exposure. Using this rat model, we would like to present our data of changes due to trade-offs in elucidating the substance of "thrifty phenotype" and body-tendency. (COI:No)

### SY2-2

**Prenatal undernutrition increases expression of an orphan membrane transporter, SLC22a23 in rat brain**

Yasuhiro Uchimura[1], Kodai Hino[1], Tomoko Kimura[1,2], Masakazu Shinohara[3], Yataro Daigo[4], Jun Udagawa[1] (*[1]Department of Anatomy and Cell Biology, Shiga University of Medical Science, [2]Faculty of Health Sciences, Kyoko Tachibana University, [3]Division of Epidemiology and The Integrated Center for Mass Spectrometry, Kobe University Graduate School of Medicine, [4]Department of Medical Oncology, Cancer Center, and Center for Advanced Medicine against Cancer, Shiga University of Medical Science*)

In order to identify the genes involved in the hyperactivity observed in adult rats subjected to prenatal undernutrition (40% food intake relative to control animals between gestational day 5.5 to 10.5), we performed RNA array analysis using mRNAs isolated from rat embryo (gestational day 10.5). We found that the expression of solute carrier 22 family member 23 (SLC22a23) was statistically significantly higher in the forebrain of embryos subjected to prenatal undernutrition compared to control animals (fc >2, padj < $10^{-13}$). Furthermore, the expression of SLC22a23 gene was statistically significantly higher in adult brain subjected to prenatal undernutrition compared to control animals (fc > 2.2, p < 0.009). These results suggest that the overexpression of SLC22a23 gene is a cause of hyperactivity observed in adult animals subjected to prenatal undernutrition. Although SLC22a23 gene is thought to belong to SLC transporter family, the function of SLC22a23 transporter is unknown. We are trying to identify the substrate, the cells and the sites of expression in rat brain. Our research may reveal the molecular mechanisms underpin the hyperactivity induced by prenatal undernutrition. (COI:No)

### SY2-3

**The role of translation repressor, 4E-BP1, for the development of fatty liver under protein deprivation**

Yuka Toyoshima[1,2], Fumiaki Yoshizawa[3], Reiko Tokita[2], Yusuke Taguchi[2], Shinichiro Takahashi[4], Hisanori Kato[5], Shiro Minami[2] (*[1]Institute for Human Life Innovation, Ochanomizu University, [2]Department of Bioregulation, Institute for Advanced Medical Sciences, Nippon Medical School, [3]Department of Agrobiology and Bioresources, School of Agriculture, Utsunomiya University, [4]Department of Animal Sciences, Graduate School of Agriculture and Life Science, The University of Tokyo, [5]Department of Applied Biological Chemistry, Graduate School of Agricultural and Life Sciences, The University of Tokyo*)

Protein deprivation is known to affect glucose and lipid metabolism, resulting in the development of fatty liver in humans and animals. Impaired triglyceride (TG) secretion from the liver and the enhancement of TG synthesis in the liver have been involved in the increase in hepatic TG level under protein deprivation; however, the underlying molecular mechanisms remain largely unknown. It is also unclear whether other lipid metabolic pathways are involved in the TG accumulation during protein deprivation. We have shown that a low-protein diet increases eukaryotic translation initiation factor 4E-binding protein 1 (4E-BP1) protein and TG levels in the livers of growing rats. 4E-BP1 is a substrate of mechanistic target of rapamycin complex 1 (mTORC1) and regulates the initiation of translation. In addition, there are increasing evidence showing that mTORC1 pathway including 4E-BP1 controls lipid metabolism. Therefore, we hypothesized that the increase in the 4E-BP1 protein level contributes to the TG accumulation in the liver under protein deprivation. Here, we introduce our studies and discuss the role of 4E-BP1 in the development of fatty liver under protein deprivation. (COI:No)

### SY2-4

**Change in Size and Function of Adipocyte in Low-Birth-Weight Infants**

Akio Ebata[1], Yuya Nakano[1], Yoko Mizukoshi[1], Yoshiyuki Hasebe[1], Katsumi Mizuno[1] (*[1]Showa university Dept Pediatrics*)

**Background/Aims:** Low-birth-weight (LBW) infants have a high risk of developing insulin resistance and associated disorders later in life. This study aimed to evaluate differences in adipocyte size during infancy between LBW infants and term appropriate-for-gestational-age (AGA) infants.
**Method**: We assessed 93 infants (15 LBW and 78 term AGA infants) aged 0.5-4.0 years who would undergo surgery during infancy. Anthropometric measurements and blood samples were evaluated and obtained preoperatively. Adipose tissue samples were obtained from the patients intraoperatively. Each sample was immediately fixed by osmic acid, and the adipocyte diameters were evaluated after separating the adipocytes from each other.
**Results**: LBW infants have significantly larger size of adipocyte, compared to term AGA infants ($p < 0.05$), although the body mass index (BMI) was similar between the groups. Significant differences were strengthened after adjusting for variables such as age and BMI and age ($p < 0.001$). However, the adipocyte diameters were not associated with hematologic parameters.
**Conclusions**: The adipocyte size of LBW infants during infancy is larger than that of term AGA infants. (COI:No)

### SY2-5

**Underweight and developmental disorders/psychiatric disorders**

Noriyoshi Usui[1,2,3,4] (*[1]Department of Neuroscience and Cell Biology, Graduate School of Medicine, Osaka University, [2]United Graduate School of Child Development, Osaka University, [3]Global Center for Medical Engineering and Informatics, Osaka University, [4]Addiction Research Unit, Osaka Psychiatric Research Center, Osaka Psychiatric Medical Center*)

It has been pointed out that low birth weight (LBW) infants are at risks for not only developmental delay and developmental disorders (autism spectrum disorder, learning disability, and attention deficit hyperactivity disorder) but also adult health and psychiatric disorders (schizophrenia and depression). Recently, we have identified *ZBTB16* as a gene involved in sociality. Deletion of *Zbtb16* in mice leads to small body size and weight loss. *Zbtb16* KO mice also show both autism spectrum disorder- and schizophrenia-like behaviors such as social impairment, repetitive behaviors, risk-taking behaviors, and cognitive impairment. These data suggest that developmental problems in *Zbtb16* KO mice may be associated with disorders-relevant phenotypes. In this symposium, we will discuss our latest findings with a focus on studies using *Zbtb16* KO mice. (COI:No)

### SY2-6

**Undernutrition in utero and risk of Non-alcoholic fatty liver disease (NAFLD) in later life**

Hiroaki Itoh[1] (*[1]Department of Obstetrics and Gynecology, Hamamatsu University School of Medicine*)

Non-alcoholic fatty liver disease (NAFLD) is a hepatic manifestation of metabolic syndrome. We recently reported that a treatment with tauroursodeoxycholic acid (TUDCA), a secondary bile acid, improved developmentally-deteriorated hepatic steatosis in an undernourishment (UN, 40% caloric restriction) *in utero* mouse model after a postnatal high-fat diet (HFD). We performed a microarray analysis and focused on two genes (Cidea and Cidec) because they are enhancers of lipid droplet (LD) sizes in hepatocytes and showed the greatest up-regulation in expression by UN that were completely recovered by TUDCA, concomitant with parallel changes in LD sizes. TUDCA remodeled developmentally-induced histone modifications (di-methylation of H3K4, H3K27, or H3K36), but not DNA methylation, around the Cidea and Cidec genes in UN pups only. Changes of these histone modifications may contribute to the markedly down-regulated expression of Cidea and Cidec genes in UN pups, which was observed in alleviation of hepatic fat deposition even under HFD. These results provide an insight into the future of precision medicine for developmentally-programmed hepatic steatosis by targeting histone modification. (COI:No)

### Symposium3

### The potential of stem cell and rehabilitation therapies for brain disorders

(March 28, Sun. 14：20～16：20, Room2)

### SY3-1

**Development of novel stem cell therapies for perinatal brain injury**

Yoshiaki Sato[1] ([1]*Division of Neonatology, Center for Maternal-Neonatal Care, Nagoya University Hospital*)

Neonatal hypoxic ischemic encephalopathy (HIE) is an important cause of neonatal death and permanent neurological deficits. Stem cell therapy using various cell sources has been recently identified as a novel therapy for neonatal HI. Muse cells are a novel type of endogenous stem cells that are able to self-renew, display pluripotency. We evaluated the effect of Muse cells for neonatal HIE with model rats. We have shown that Muse cells, but not non-Muse Mesenchymal Stem Cells (MSCs), migrated into injured brain in the model rats, and that Muse cells injected intravenously ameliorated learning deficit and motor impairment induced by HIE. In addition, the non-clinical evaluations performed with clinical grade Muse cell-based product, CL2020 developed by Life Science Institute, Inc. also indicated treatment effects without any adverse effects. Based on the results, we started an exploratory investigator-initiated clinical trial (SHIELD trial) to evaluate the safety and the tolerability of the treatment. The treatment with CL2020 does not require special cell processing facility or devices, leading to develop a novel epoch-making therapy for HIE.

(COI:Properly Declared)

### SY3-2

**Elucidation of damaged brain regeneration mechanism by cell therapy and enriched environment for neonatal white matter injury**

Naoki Tajiri[1], Shino Ogawa[1,2], Atsunori Hattori[1], Ayano Otani[1,2], Shinya Ueno[1], Takeshi Shimizu[1], Hideki Hida[1] ([1]*Dept of Neurophysiol & Brain Sci, Grad Sch Med, Nagoya City Univ*, [2]*Dept Ob-Gyn, Grad Sch Med, Nagoya City Univ*)

Hypoxia-ischemia (H-I) in preterm infants occasionally results in neonatal white matter injury (NWMI) associated with neurodevelopmental disabilities such as paralysis and cognitive dysfunction. Based on selective vulnerability of late oligodendrocyte progenitor cells (OPCs) to H-I, we made a rat NWMI model that showed hindlimb motor dysfunction without loss of cortical neurons, hypomyelination in the sensorimotor cortex and disturbed cortical motor map in the ipsilateral motor cortex. To find out new effective treatment for NWMI, we are challenging cell therapy to NWMI model using OPCs as well as focusing on the enriched environment (EE). In study 1, we investigated whether the grafted OPCs can promote motor function in NWMI model via immunosuppressant. In study 2, we examined whether EE could improve the motor dysfunction caused by NWMI, and change the microenvironment. Our data suggest that OPC transplantation during the period of development has a potency to improve deteriorated motor function and increased cell survival rate of grafted cells in the NWMI model. Also, we clarified that long-term EE rearing in NWMI model, is one of treatments that can promote brain development. (COI:No)

### SY3-3

**Cell therapy, electrical stimulation, and rehabilitation for disorders in the central nervous system**

Takao Yasuhara[1], Isao Date[1] ([1]*Department of Neurological Surgery, Okayama University Graduate School of Medicine*)

INTRODUCTION
Recently, regenerative medicine is developing with subsequent clinical application for various central nervous disorders. In this presentation, our data of basic and clinical research are shown with recent progress in this field, current obstacles to overcome, and precision medicine in cell therapy.
BASIC RESEARCH
1. We continued encapsulated cell transplantation for various disease models of rats. This method enables us to supply neurotrophic factors without immunosuppression.
2. A new device was developed to achieve electrical stimulation like clinical settings for small animals. Continuous stimulation of Vagal nerve and spinal cord exerted neuroprotection in Parkinson's disease model of rats.
3. Exercise for Parkinson's disease model of rats ameliorated motor function with neuroprotective/neurogeneic potentials. Limited exercise with hindlimb suspension exacerbated motor function with reduced neurogenesis.

CLINICAL RESEARCH
We participated in STEMTRA trial, in which the efficacy of allogeneic modified bone marrow-derived mesenchymal stem cell implantation was tested. Motor function of the transplanted patients was improved at 6 months after treatment.

(COI:Properly Declared)

### SY3-4

**Reparative medicine; challenge to medical innovation by non-tumorigenic pluripotent Muse cells**

Mari Dezawa[1] ([1]*Department of Stem Cell Biology and Histology, Tohoku University Graduate School of Medicine, Sendai, Japan*)

Muse cells are naturally existing, non-tumorigenic reparative endogenous pluripotent stem cells identified by SSEA-3(+). They are able to selectively home to damaged site after intravenous injection by sphingosine-1-phosphate (S1P)-S1PR2 axis and spontaneously differentiate into tissue-constituent cells to repair tissue. Intravenous drip is the main method of administration, making surgical operation unnecessary. Because Muse cells have an immunomodulatory system similar to the placenta, donor-derived Muse cells can be directly administered to patients without HLA-matching or long term-immunosuppression. Allogeneic Muse cells remain in the host tissue as differentiated functional cells for more than half a year. Clinical trials for the treatment of myocardial infarction, stroke, epidermolysis bullosa, spinal cord injury and cerebral palsy with intravenous drip of donor-derived Muse cells are currently being conducted by the Life Science Institute Inc.
Muse cells may safely provide clinically relevant effects compatible with the 'body's natural repair systems' by a simple cost-effective strategy; collection, expansion and intravenous drip, thus may deliver medical care innovation.

(COI:Properly Declared)

### Symposium4

### Physiome of the Circulation

(March 28, Sun. 14：20～16：20, Room3)

### SY4-1

**How elevated blood pressure influences the vascular smooth muscle cells in the aortic walls? -more stretch due to the shear deformation between the elastic laminas and not so stretched stress fibers-**

Shukei Sugita[1] ([1]*Nagoya Institute of Technology*)

The talk describes what changes in the mechanical environment intraluminal pressurization brings to smooth muscle cells in the aortic wall. Our observation demonstrates that intraluminal pressurization causes shear deformation at the cell scale, where neighboring elastic laminas move in the circumferentially opposite direction to each other. A combination of the shear deformation with circumferential stretch caused by the pressurization maximizes the tissue stretch in the direction angled by 30° counterclockwise from the circumferential axis in the radial-circumferential plane. In contrast, stress fibers in aortic smooth muscle cells are aligned at a clockwise angle of 15° to the circumferential axis. This means that the stress fibers that are responsible for force transmission to the cell nucleus do not receive the stretch as much as the cells. We concluded that the effects of intraluminal pressurization of the aorta on smooth muscle cells are not simple: cells are stretched more but transmitted stretch through stress fibers is less than ones in our previous knowledge. (COI:No)

### SY4-2

**The role of ErbB signaling in the heart**

Maretoshi Hirai[1] ([1]*Kansai Medical Univ, Dept of Pharm*)

During heart development, trabeculae play an important role for maintaining cardiac contractility in developing immature heart. Trabecular myocytes have more differentiated phenotype with mature sarcomeric structure, thus assisting cardiac contractile function. For proper development of trabeculae, both ErbB2 and ErbB4 receptors are known to be indispensable, however, it remains to be known how trabecular cardiomyocytes exit from cell cycle to acquire more differentiated phenotype. In this study, we showed that endocytic protein, 'Numb' leads to the inactivation of ErbB2 receptor in trabecular myocytes through the regulation of vesicular trafficking, resulting in the cell cycle exit. We also found that specifically ErbB2, not ErbB4, is responsible for this exit. This is unexpected because it is widely known that ErbB2/ErbB4 heterodimer function as a receptor for their ligand, Nrg1, activating the same signaling pathway. Recently, we found another evidence showing that ErbB2 and ErbB4 have a distinct function through the analyses of gene-targeted mice. In this talk, we will show the unknown function of ErbB receptors in heart. (COI:No)

### SY4-3

**Drugs, heart, interactions and their effects**

Kazuharu Furutani[1,2] ([1]*Dept Pharmacol, Fac Pharma Sci, Tokushima Bunri Univ,* [2]*Dept Physiol & Mem Biol, Univ California Davis*)

The electrical activity of the heart is crucial for maintaining life. Drugs targeting cardiac ion channels and receptors profoundly impact cardiac physiology. Drugs that have similar molecular effects on their target proteins can exhibit unexpectedly different actions at a cellular level, and qualitatively different pro- or anti-arrhythmic outcomes. To understand how subtle differences in drug mechanism will impact higher level physiology, it is critical to know how physiological context will impact a drug's effect. My research focuses on identifying fundamental physiological and pharmacological mechanisms that make human ether-a-go-go-related gene (hERG) potassium channel blockers safe or arrhythmogenic and make M2 muscarinic receptor agonists more or less effective. For both targets, drug effects are coupled to the target protein's response to membrane potentials. In my presentation, I will highlight our recent studies to understand how membrane potential regulates drug effects on cardiac ion channel and receptors, which in turn affect electrophysiology in the heart. (COI:No)

### SY4-4

**A 2D-simulation analysis of conduction failure caused by a gain-of-function mutation of TRPM4 channel**

Ryuji Inoue[1], Hu Yaopeng[2] ([1]*Division for the Promotion of Science, Fukuoka University,* [2]*Department of Physiology, Fukuoka University School of Medicine*)

TRPM4, a $Ca^{2+}$-activated monovalent cation-selective channel, is most abundantly expressed in the Purkinje fiber system in the heart. Recently, a single mutation on its distal N-terminus E7K was reported to be causative for a familial conduction block that accompanies enhanced channel protein expression and activity (i.e. gain-of-function: GOF). To address the question of why this conduction failure occurs by the GOF mutation, we investigated the gating kinetics of E7K-TRPM4 channel and performed 2D-simulation. TRPM4 channels expressed in HEK293 cells showed a greatly accelerated close-to-open transition by E7K mutation. A 2D-simulation with Pan-Rudy model showed that, while in a homogenous cardiomyocyte sheet the E7K mutation slightly enhances the conduction velocity of action potentials, it slows their propagation eventually resulting in complete conduction failure when the cell-membrane density of TRPM4 channel and/or the fraction of fibroblasts in a heterogeneously arranged cardiomyocyte/fibroblast sheet are increased. These results may suggest the pleiotropic impacts of the E7K mutation in cardiac arrhythmogenesis which depends critically on the tissue-level complexity. (COI:No)

### SY4-5

**Analysis of metabolic diseases using a multiple organ model connected by the blood circulation: taking type II diabetes as an example**

Hiroshi Suzuki[1], Naoto Nishimura[1], Keita Tokuda[1], Yoshiaki Kariya[1], Masashi Honma[1] ([1]*Department of Pharmacy, The University of Tokyo Hospital*)

Lifestyle-related diseases may be developed when the changes in the metabolic pathways in multiple organs exceed the limits of maintaining the homeostasis. We aimed at developing a novel method to identify the metabolic pathways which are changed under type 2 diabetic conditions. We constructed metabolic pathway models in the liver, adipose and skeletal muscle by using the Recon 3D model. By considering the expression of metabolic enzymes, we connected these organ models to construct the network model by considering the substrate fluxes between the organs through blood circulation. Then, we analyzed the changes in the mRNA levels of metabolic enzymes in patients. Analysis indicated that the metabolic pathways such as the hepatic synthesis of fatty acids and beta-oxidation and other pathways such as adipocyte beta-oxidation and TCA cycle were suggested to be changed, which is consistent with the previous report. We could also identify novel pathways which are changed in the type 2 diabetes. Our method may be useful in identifying the novel drug targets and/or proposing the novel biomarkers for the disease progression. (COI:Properly Declared)

### Symposium5

### Anatomy and physiology of the respiratory center: cutting-edge research on the mechanisms of respiratory rhythm generation and hypoxic responsiveness

(March 28, Sun. 14：20～16：20, Room4)

### SY5-1

**Cytoarchitecture of neurons in the parafacial respiratory group (pFRG) in newborn rat medulla**

Keiko Ikeda[1], Hiroshi Onimaru[2] (*[1]Dept Clin Res, Murayama Med Cente, [2]Dept Physiol, Showa Univ, Sch. of Med., Tokyo* )

The respiratory rhythm is generated by neurons in two independent groups, parafacial respiratory group (pFRG)/retrotrapezoid nucleus (RTN) and the pre-Bötzinger complex (preBötC). Both reside in the medulla oblongata to form coupled oscillator. The neurons in the pFRG/RTN preferentially innervate caudal ventral respiratory group (VRG) to drive expiratory premotor neuron activities and trigger inspiratory burst generation, whereas those in the preBötC project to rostral VRG to drive inspiratory premotor activities. pFRG/RTN of neonatal rats predominantly consists of neurons that burst prior to inspiration, named pre-inspiratory (Pre-I) neurons. Some of Pre-I neurons express a paired-like homeobox 2b gene (*Phox2b*) and that they are intrinsically $CO_2$-sensitive.We generated transgenic rat line harboring a mouse BAC carrying a *Phox2b* that was modified to drive enhanced yellow fluorescent protein (EYFP). The EYFP fluorescent labeling of Pre-I neurons made it possible to visualize pFRG/RTN in the medulla in three dimensional way. In addition, gene expression profiles of Pre-I neurons has become clear using in situ hybridization technique on the EYFP fluorescent positive cells. (COI:No)

### SY5-2

**Network mechanism for inspiratory rhythm generation in the preBötzinger complex of the mice medullary slice**

Yoshihiko Oke[1], Fumikazu Miwakeichi[2,3], Yoshitaka Oku[1], Johannes Hirrlinger[4,5], Swen Hülsmann[6] (*[1]Division of Physiome, Department of Physiology, Hyogo College of Medicine, Hyogo, Japan, [2]Department of Statistical Modeling, The Institute of Statistical Mathematics, Tokyo, Japan, [3]Department of Statistical Science, School of Multidisciplinary Sciences, The Graduate University for Advanced Studies, Tokyo, Japan, [4]Carl-Ludwig-Institute for Physiology, University of Leipzig, Leipzig, Germany, [5]Department of Neurogenetics, Max Planck Institute of Experimental Medicine, G?ttingen, Germany, [6]Clinic for Anesthesiology, G?ttingen University, G?ttingen, Germany*)

Spontaneous inspiratory rhythm is generated in the pre-Bötzinger complex (preBötC). The neuronal network structure in the preBötC remains unknown despite of its importance to understand inspiratory rhythmogenesis. Applying $Ca^{2+}$ imaging to the medullary transverse slices in TG mice expressing EGFP in GlyT2+ neurons and tdTomato in GAD65+ neurons, we revealed types of inspiratory neurons in the preBötC. Based on the investigation of activation sequence of inspiratory neuron types during rhythmic bursts, we proposed inspiratory neuronal network model in the preBötC. At the initial phase of rhythmic bursts, both excitatory (GlyT2-/ GAD65-) and glycinergic (GlyT2+/ GAD65-) neurons exhibiting a small $Ca^{2+}$ rise with short duration were stochastically activated. These activation might contribute to generation of earlier-phased burstlet. Then, coordinated inputs from these neuron types might activate and shape both excitatory and glycinergic neurons showing a large $Ca^{2+}$ signal to generate high-amplitude inspiratory burst. Lastly, we introduce our latest results about early postnatal development of inspiratory neuron types in the preBötC. (COI:No)

### SY5-3

**Cellular mechanisms of hypoxia/hypercapnia reception in the medullary cardio-respiratory center**

Hiroshi Onimaru[1], Itaru Yazawa[2], Keiko Ikeda[3], Yasumasa Okada[3] (*[1]Department of Physiology, Showa University School of Medicine, [2]Global Res. Ctr. for Innovative Life Sci., Hoshi Univ. Sch. of Pharm. & Pharmaceut. Sc, Tokyo, Japan, [3]Clinical Research Center, Murayama Medical Center, Musashimurayama, Tokyo Japan*)

We focused on 1) the rostral ventrolateral medulla where respiratory and cardio-vascular centers are located, and 2) the nucleus of the solitary tract (NTS) in the dorsal medulla that is a first relay station of sensory inputs involving autonomic functions. Although these regions include cells that are sensitive to hypercapnia and/or hypoxia, the cell types and detailed structures are not well understood. Our electrophysiological study in the ventral medulla revealed that at least some of Phox2b-positive/TH-positive neurons were intrinsically sensitive to hypoxia but less sensitive to hypercapnia, whereas Phox2b-positive/TH-negative neurons were intrinsically $CO_2$ sensitive. Responses of Phox2b-negative/TH-negative respiratory-related neurons to hypoxia were varying. In the NTS, we analyzed cellular responses to hypercapnia and hypoxia by using calcium imaging. We found that the NTS contained cells that were sensitive to hypercapnia, hypoxia and both stimulations. It was noticeable that more than 30% of putative astrocytes in the NTS were sensitive to hypercapnia, hypoxia or both. We will discuss cellular mechanisms of hypoxia/hypercapnia sensitivity in the medulla. (COI:No)

### SY5-4

**Astrocytes mediate post-hypoxic persistent respiratory augmentation**

Isato Fukushi[1,2], Kotaro Takeda[2,3], Mieczyslaw Pokorski[4], Shigefumi Yokota[5], Yasumasa Okada[2] (*[1]Fac Hlth Sci, Uekusa Gakuen Univ, Japan, [2]Clin Res Ctr, Murayama Med Ctr, Japan, [3]Sch Hlthcare, Fujita Hlth Univ, Japan, [4]Faculty of Health Sciences, The Jan Dlugosz University in Czestochowa, Poland, [5]Dept Anat & Neurosci, Shimane Univ Sch Med, Japan*)

Hypoxia increases respiration and the increase persists into the post-hypoxia recovery phase. The mechanism of post-hypoxia persisting respiratory augmentation (PHPRA) has not been well elucidated. Based on our anatomical observation that astrocytes and neurons are coupled in major respiratory regions in the brainstem, we hypothesized that astrocytes are involved in PHPRA. We investigated this issue by conducting in vitro and in vivo experiments in rodents. In vitro, we found that neural respiratory outputs during hypoxia loading and post-hypoxia recovery phases were recordable in the medulla-spinal cord preparation isolated from newborn rats. We compared respiratory frequencies in the post-hypoxia recovery phase in preparations without and with administration of arundic acid, a blocker of astrocytic activation. In vivo, we assessed the persisting post-hypoxia ventilatory augmentation in adult mice by whole body plethysmography. Likewise, we compared the levels of ventilation before and after administration of arundic acid. In both experimental conditions, arundic acid blocked the PHPRA. We conclude that astrocytes are conducive to the upholding of PHPRA. (COI:No)

### SY5-5

**Molecular mechanism underlying hypoxia sensing and adaptation**

Akito Nakao[1] (*[1]Department of Synthetic Chemistry and Biological Chemistry, Graduate School of Engineering, Kyoto University, Japan*)

Hypoxia sensors are essential for regulating local oxygen homeostasis within the body. This is especially important for the CNS, which is particularly vulnerable to oxygen deprivation due to high energetic demand. Recently, our group reported that transient receptor potential (TRP) A1 acts as an acute hypoxia sensor in astrocytes of the parafacial respiratory group (pFRG) and retrotrapezoid nucleus (RTN) region through oxygen-dependent protein trafficking. Hypoxia-induced TRPA1 activity accelerates ATP release from pFRG/RTN astrocytes in the medulla to modulate respiratory activity. The oxygen-dependent protein trafficking of TRPA1 channels would be an important clue in understanding the universal molecular mechanism underlying hypoxia sensing and adaptation. (COI:No)

### Symposium6

### Mereological approach for unraveling the structure - function relationship of the central nervous system

(March 28, Sun. 14：20～16：20, Room6)

### SY6-1

**Development of multimodal neural devices integrating multielectrode recording and light manipulation technologies**

Tetsu Tanaka[1] ([1]*Graduate School of Biomedical Engineeriing*)

Multimodal neural devices that integrate multielectrode recording and light manipulation technologies are needed to elucidate the relationship between the "part-whole" in living organisms, especially in the brain. The former technology allows simultaneous recording of bioelectrical activity such as neuronal activity (firing) and local field potential (LFP) from multiple locations to enable spatio-temporal patterning analysis. The latter technology enables biological manipulation by optically stimulating light-sensitive proteins expressed in neurons by genetic engineering. This presentation presents a multimodal neural device having light manipulation functions obtained by upconversion light-emitting materials that convert near-infrared light irradiated from outside the tissue into specific visible light for optical stimulation. This neural device eliminates the need for optical fibers and light-emitting diodes for optical tissue stimulation. The multimodal neural devices can be used to elucidate "part-whole" interactions, such as how local optical stimulation induces LFP oscillations and affects neuronal activity in the cortex and hippocampal layer structures. (COI:No)

### SY6-2

**Representation of fear memory by inter-regional coactivation of local cell assemblies**

Kenji Mizuseki[1] ([1]*Dep Physiol, Grad Sch Med, Osaka City Univ*)

The amygdala, hippocampus, and prefrontal cortex are involved in fear memory. However, how these brain areas co-operate to support fear memory remains elusive. To address this question, we performed simultaneous large-scale electrophysiological recordings in the basolateral amygdala, ventral hippocampus, and prefrontal cortex and examined well-isolated units in fear conditioned rats. Recordings were performed continuously throughout baseline, conditioning, context retention, cue retention and extinction, retention of extinction, and homecage sessions preceding, interleaved, and following the behavioral sessions. The proportion of time spent in freezing behavior indicated that the rats had learned an association between cues and shocks and they retrieved the association during retention sessions. Based on our preliminary results, we hypothesize that elements of a given memory are instantly encoded by local cell assemblies within various brain regions, whereas the inter-regional coactivation of the distributed information develops in an experience-dependent manner during memory consolidation. (COI:No)

### SY6-3

**Orchestration of sub-ensemble activities of engram cells constitute a whole picture of one episodic memory**

Noriaki Ohkawa[1] ([1]*Comprehensive Research Facilities for Advanced Medical Science, Dokkyo Medical University*)

The brain stores memories through a set of neurons, termed engram cells. However, it remains unclear how engram cells are organized to constitute a whole picture of one episodic memory. To extract the characteristics of population activity of engram cells, we established a unique imaging system to identify engram cells with a fluorescent protein, KikGR, and to record the $Ca^{2+}$ signals corresponding to the activity of engram cells and non-engram cells during a novel episodic event. To address components of one memory, we proposed to decompose population activity into coactivated neuronal ensembles and time series of their synchronous activities by non-negative matrix factorization (NMF), an algorithm for factorization of a matrix into two matrices. By this approach, we found out that information of one episodic experience is composed of several hippocampal engram sub-ensembles defined by individual synchronous activities, and ~40% of the engram sub-ensemble activities survive from post-experience sleep through retrieval session. Reappearance of the sub-ensemble activities may be a principle of processing of episodic memory during memory consolidation in sleep and during recall. (COI:No)

### SY6-4

**A viewpoint from whole through non-invasive imaging**

Keigo Hikishima[1] ([1]*Health & Med Res Inst, AIST*)

To understand the brain as a system, it is important to measure its structural and functional network at a whole level. MRI has been widely used not only for clinical imaging but also for preclinical research because of its ability to evaluate the brain non-invasively with the various imaging contrasts. To make it possible to perform brain mapping and network analysis also in experimental small animals (mouse, rat and marmoset), we have developed those brain imaging platforms including the standard brain templates and three-dimensional brain atlases. With these brain resources as a platform, we have been analyzing the neurological disease models using whole level multimodality imaging such as MRI and PET. On the other hand, since the brain system has a complex hierarchical structure, understanding it from parts as well as whole is essential, and non-invasive imaging can play an important role in multiscale measurements such as simultaneous measurements. In this symposium, we will introduce our work from a viewpoint of whole through multimodal non-invasive measurement and our efforts in multiscale measurement to elucidate the part-whole relation. (COI:No)

### SY6-5

**Data-Driven Ontology Discovery and Its Possible Framework Based on Knowledge Representation in the Semantic Web**

Hiroaki Wagatsuma[1,2] ([1]*Graduate School of Life Science and Systems Engineering, Kyushu Institute of Technology,* [2]*RIKEN CBS*)

With respect to classical hypothesis-driven approaches for scientific findings, expectations to data-driven approaches are increasing as ways to find a hidden relationship among data. However, the simple data-driven approach is not effective without appropriate annotation of data, which usually requires human operations according to expert knowledge and abilities. A hybrid, or fusion method of hypothesis- and data-driven approaches is highly important, so called data-driven ontology discovery scheme. In this proposal, as a new computer-based hypothesis-driven approaches will be developed as a linkage system for multi-dimensional data for a meaningful reconstruction of what the whole structure represents as a scientific evidence, which not only contributes to the design of the standard data format to connect multiple datasets with different resolutions in time and spatial domains by effective indexing methods, but also a data searching technology across multiple databases by the establishment of hierarchical metadata descriptions. As an inheritance from outcomes in the INCF Datasharing Program, NIX data model and odML metadata mark-up language will be extended in the purpose. (COI:No)

### Symposium7

### New insight into oligodendrocyte research

(March 28, Sun. 14：20～16：20, Room7)

### SY7-1

**Investigation of a novel mechanism underlying neuronal subtype-dependent myelination**

Takeshi Shimizu[1], Hideji Nurakoshi[2,3], Hidetoshi Matsumoto[4], Akimasa Ishida[1], Naoki Tajiri[1], Hideki Hida[1] (*[1]Dept of Neurophysiol and Brain Sci, Grad Sch of Med Sci, Nagoya City Univ, [2]Supp Cent for Brain Res, Natl Inst for Physiol Sci, [3]Dept of Physiol Sci, Grad Univ for Advanced Studies, [4]Dept of Mater Sci and Engineer, Tokyo Inst of Technol*)

We previously reported that some populations of oligodendrocytes (OLs) in the corpus callosum predominantly ensheathed axons derived from either motor cortex or sensory cortex, suggesting characteristics of myelination depending on neuronal subtypes. In addition, there are large and small caliber axons in the central nervous system. The ratio of diameter of axon+myelin to axon diameter (g-ratio) is adjusted to optimum values for each axon, which is essential for higher brain functions. This indicates that various diameters of axons induce some myelin-regulatory factors. However, mechanisms underlying neuronal subtype-dependent myelination are not well known.
We assessed physical factors involving axonal subtype-dependent myelination. To visualize OL generating forces during myelination, a tension sensor based on fluorescence resonance energy transfer (FRET) was used. As a result, OLs generated various strengths of mechanical forces and focal adhesions (FAs) depending on axon diameters. These results suggest that intracellular mechanical signaling is initiated from FA, which is dependent on neuronal subtypes, and controls myelin formation involving actin assembly/disassembly. (COI:No)

### SY7-2

**New insights on oligodendrocyte-mediated demyelination of multiple sclerosis and experimental autoimmune encephalomyelitis**

Yoshio Bando[1] (*[1]Dept Anat, Grad Sch Med, Akita University*)

Recent insights into its molecular neuropathology and immunology have provided a comprehensive overview of the pathology of multiple sclerosis (MS), including demyelination and axonal loss. To date, neuropathology in MS as the human autoimmune disease is considered to be mainly mediated by autoreactive leukocytes. By contrast, accumulating evidence has also suggested that the inflammation and tissue damage in MS and its animal model experimental autoimmune encephalomyelitis (EAE) is also critically regulated by glial cells in the CNS. However, molecular mechanism of the glial cells-mediated pathology of MS/EAE has not been fully understood. We have examined the oligodendrocyte-mediated demyelination and axonal injury. In this symposium, we discuss the pathological roles of oligodendrocytes in MS/EAE. We also highlight recent findings of abnormal myelin formation and axonal injury in EAE. (COI:No)

### SY7-3

**Oligodendrocytes induce axonal conduction plasticity in myelinated fibers and facilitate synaptic plasticity at destination synapses**

Yoshihiko Yamazaki[1] (*[1]Department of Physiology, Yamagata University School of Medicine*)

Myelination is not the sole function of oligodendrocytes. We discovered that artificial oligodendrocytic depolarization increases conduction velocity, enhances axonal excitability, and facilitates excitatory synaptic transmission at destination synapses. Recent evidence suggests that the conduction properties of myelinated axons are quite variable and that the longitudinal distribution of myelin is not uniform. In this study, we used mice with oligodendrocytes expressing channelrhodopsin-2 to investigate the complexity of the modulation of axonal conduction by oligodendrocyte. We found that the magnitude of the increase in conduction velocity induced by optogenetic depolarization of oligodendrocytes varies at different positions along axons. We also examined the effects of oligodendrocyte depolarization on the induction of long-term potentiation (LTP) at destination synapses. The LTP induced by theta burst stimulation was enhanced after oligodendrocyte depolarization. These results indicate that oligodendrocyte depolarization contributes to the fine control of neuronal activities and facilitates destination synaptic functions. (COI:No)

### SY7-4

**Large-scale electron microscopic volume imaging of interfascicular oligodendrocytes in the mouse corpus callosum**

Tatsuhide Tanaka[1], Nobuhiko Ono[2], Akio Wanaka[1] (*[1]Department of Anatomy and Neuroscience, Nara Medical University, [2]Department of Anatomy, Division of Histology and Cell Biology, Jichi Medical University*)

Single oligodendrocytes produce myelin sheaths around multiple axons in the central nervous system. Interfascicular oligodendrocytes (IOs) in the white matter are primarily aligned in rows. IOs facilitate nerve conduction, but their detailed morphologies remain largely unknown. We three-dimensionally reconstructed constitutively aligned IOs in the corpus callosum of adult mouse using serial block face-scanning electron microscopy (SBF-SEM). The constitutively aligned IOs were morphologically polarized and extended thick processes from the cytoplasm-rich part of the cell. The multiple branched processes of each IO myelinated multiple axons with biased diameters and myelin thicknesses, indicating that individual IOs have their own myelination profiles even on distinct target axons. We further reconstructed the sheath immediately adjacent to that derived from each of the seven IOs; the thicknesses of the pair of sheaths were significantly correlated despite emanating from different IOs. These results indicate that the heterogeneity of individual oligodendrocytes involves biased structural patterns of myelination and is modified on the basis of interactions with axons. (COI:No)

### Symposium8

### Crosstalk in modifiable and non-modifiable risk factors for cardiovascular disease

(March 28, Sun. 16：30～18：30, Room3)

### SY8-1

**Contraction Rhythm Homeostasis manifested in warmed cardiomyocytes – the mechanism of stable rhythm and high efficiency of heartbeat at body temperature –**

Seine A. Shintani[1], Takumi Washio[2,3], Hideo Higuchi[4] ([1]*Department of Biomedical Sciences, College of Life and Health Sciences, Chubu University,* [2]*UT-Heart Inc.,* [3]*Future Center InitiativeThe University of Tokyo,* [4]*Department of Physics, Graduate School of ScienceThe University of Tokyo*)

In an in vitro study using rat cardiomyocytes, we found that the contraction rhythm of Hyperthermal Sarcomeric Oscillations (HSOs), which becomes apparent when the cardiomyocytes are warmed, keeps homeostasis. We have succeeded in reproducing the contraction rhythm homeostasis with a simulation model that considers the movement of individual myosin molecules. In isolated cardiomyocytes, the high frequency (5-7 Hz) of HSOs was the same as the heart rate, even though the intracellular calcium concentration changed at a low frequency (~1 Hz). HSOs occur at about 5 Hz at 37 ℃, which is the body temperature of rats, and the frequency (7.6 Hz) and amplitude of oscillations increase and oscillation becomes remarkable at 41 ℃. The property of the fast relaxation speed is important for the function of heart in which ventricle is rapidly relaxed and expand to fill the blood. Therefore, we suggest that the result contributes to the improvement of medical technology such as advance prediction of diastolic heart failure. (COI:Properly Declared)

### SY8-2

**Effects of age, sex, and menopause on the cerebrovascular endothelial function**

Erika Iwamoto[1] ([1]*Sapporo Medical University, School of Health Sciences*)

Cerebrovascular endothelial dysfunction is associated with cerebrovascular events such as stroke and vascular dementia. The endothelial function of peripheral conduit arteries is usually assessed by ischemia-induced flow-mediated dilation (FMD). Interestingly, hypercapnia-induced shear-mediated dilation of the internal carotid artery (ICA) has been suggested to be an index of endothelial function in the cerebral vasculature. In improving/reversal endothelial dysfunction, shear stress is a key stimulus in the peripheral vasculature. Along this line, cerebral endothelial dysfunction is also related to attenuation of cerebral blood flow, and therefore, there is a possibility that enhanced shear stress in the cerebral vasculature has favorable effects on cerebrovascular endothelial function. We have recently reported that repeated shear stress elevations induced by the intermittent-hypoxia increase shear-mediated dilation of the ICA. In this symposium, my presentation would like to focus on the effects of aging, sex, and menopause on the shear-mediated dilation of the ICA. In addition, I will discuss our ongoing carotid studies to improve cerebral endothelial function. (COI:No)

### SY8-3

**Cerebrovascular carbon dioxide reactivity and flow mediated dilation in young healthy South Asian and Caucasian European men**

James Fisher[1] ([1]*Univ. Auckland·Medical Science·Physiology*)

South Asians (i.e., people with ethnic roots in the Indian subcontinent) residing in the United Kingdom (UK) have an increased risk of ischemic stroke when compared with Caucasian Europeans. An impaired cerebrovascular carbon dioxide reactivity ($CVR_{CO2}$) is an independent predictor of ischemic stroke and cardiovascular mortality. To test the hypothesis that $CVR_{CO2}$ is reduced in South Asians, middle cerebral artery blood velocity (MCAv) was monitored in young (~20 years), healthy, UK-based South Asian (n=16) and Caucasian European (n=18) men, during stepwise changes in end-tidal partial pressure of carbon dioxide. Brachial artery endothelial function was determined using the flow-mediated dilatation (FMD) technique. Neither MCAv nor $CVR_{CO2}$ were different in South Asians and Caucasian Europeans. Brachial artery FMD was lower in South Asians [5.48 (2.94) %] than Caucasian Europeans [7.41 (2.28) %; $P < 0.05$], but when FMD was corrected for shear rate no between-group differences were evident ($P > 0.05$). FMD and $CVR_{CO2}$ were not correlated. In sum, these data indicate that $CVR_{CO2}$ and FMD (corrected for shear rate) are not blunted in healthy, young, UK-based South Asian men. (COI:No)

### SY8-4

**The negative impact of post-prandial hyperglycemia and hyperlipidemia on cerebrovascular health**

Hayato Tsukamoto[1] ([1]*Faculty of Sport and Health Science, Ritsumeikan University*)

As well as cardiovascular disease, the risk of cerebrovascular disease is increased by regularly eating the high-glycemic and/or high-fat meals. Recently, it has been demonstrated that both post-prandial hyperglycemia and hyperlipidemia acutely impaired cerebrovascular function such as dynamic cerebral autoregulation (dCA), which is an important cerebrovascular mechanism to maintain relatively constant cerebral blood flow against rapid fluctuations in perfusion pressure for brain homeostasis. These observations indicated that a single meal affects cerebrovascular health and may relate to chronic cerebrovascular health. Moreover, the risk of cerebrovascular events is increased in the morning, implying that an efficient dCA may be particularly necessary in the morning. However, while breakfast consumption has an acute beneficial impact on dCA, it is not observed when breakfast is omitted. In addition, it has been also found that a lower breakfast frequency is associated with the risk of stroke, although up to 35% of young people regularly skip breakfast. In this talk, I propose that breakfast frequency and quality may be important considerations for optimizing cerebrovascular health. (COI:No)

### SY8-5

**Cardiopulmonary baroreflex control of sympathetic vasomotor outflow during exercise in younger and older individuals**

Keisho Katayama[1] ([1]*Research Center of Health, Physical Fitness and Sports, Graduate School of Medicine, Nagoya University*)

Muscle sympathetic nerve activity (MSNA) decreases or does not change from rest during leg cycling exercise at low intensity despite an activation of central command that is one mechanism of increase in arterial blood pressure (BP) during exercise. Recently, our findings suggest that a loading of cardiopulmonary baroreceptors via muscle pump-induced increases in central blood volume inhibits exercise-induced increase in MSNA, and demonstrated that activation of muscle metaboreflex attenuates this muscle pump influence on cardiopulmonary baroreflex. It is well known that a BP response to dynamic exercise even at low intensity is elevated larger in older individuals and heart failure patients compared with younger healthy individuals. These findings provide a possibility that this phenomenon is associated with modified cardiopulmonary baroreflex via different metaboreflex (activation) and muscle pump (attenuated) in older individuals and patients with heart failure. In my presentation, I will focus on cardiopulmonary baroreflex control of sympathetic vasomotor outflow regarding a different blood pressure regulation during exercise between younger and older individuals. (COI:No)

### SY8-6

**Effect of aging and exercise on cerebrovascular andcardiovascular functions**

Shigehiko Ogoh[1] ([1]*Department of Biomedical Engineering, Toyo University, Japan*)

Aging increases the risk of cardiovascular and cerebrovascular diseases. It may be associated with age-related alterations in cardiovascular and cerebrovascular functions; however, its physiological mechanism has not been fully understood. For example, previous studies have demonstrated that the sensitivity of arterial baroreflex function, which contributes to a short term blood pressure regulation, is diminished with increasing age. This can be explained by several vascular stress changes associated with aging, such as arterial stiffening. In contrast, aging does not attenuate the function of cerebral blood flow regulation, e.g. cerebral autoregulation, despite arterial stiffening. The cerebral vasculature may have a compensatory adjustment to the alteration in arterial blood pressure regulation. Under this background, exercise is recommended as a useful method to reduce the risk of cardio- and cerebrovascular diseases in older adults. Therefore, in this presentation, the author aims to provide a consideration regarding the effect of exercise on aging-induced cardiovascular and cerebrovascular regulatory systems alteration to decrease the risk of these diseases. (COI:No)

### Symposium9

### Reproductive biology up to date

(March 28, Sun. 16 : 30～18 : 30, Room5)

### SY9-1

**Switching from mitosis to meiosis is regulated by sexually different mechanism in mammalian germ cells**

Kei-ichiro Ishiguro[1] ([1]*Kumamoto Univ. IMEG*)

The regulatory mechanism of meiotic initiation is a long-standing enigma in mammals. It is known that retinoic acid (RA) signalling has a pivotal role for meiotic initiation. STRA8, which is expressed in response to RA, is thought to be a key factor promoting meiotic initiation. Previously, we identified a germ cell-specific factor MEIOSIN which associates with STRA8 (Ishiguro et al. ***Dev Cell*** 2020). The MEIOSIN-STRA8 complex acts as a transcription factor that drives meiotic gene activation and plays an essential role in the switching from mitosis to meiosis both in male and female.

Further, we identified Rb as another STRA8 binding factor that modulates the MEIOSIN-STRA8 complex. To elucidate the biological significance of Rb-STRA8 interaction, we generated mutant mice that lack the Rb-binding site of *Stra8* (*Stra8-del Rb*), in which STRA8 could not interact with Rb but the STRA8-MEIOSIN interaction was intact. *Stra8-del Rb* mice showed severe fertility defect in female but not in male, suggesting that the meiotic initiation was regulated by a sexually different mechanism. We are currently trying to understand how Rb-STRA8 interaction is involved in the initiation of meiosis. (COI:No)

### SY9-2

**Epigenome reprogramming of primordial germ cells**

Kazuki Kurimoto[1] ([1]*Dept Embryol, Nara Med Univ*)

Primordial germ cells (PGCs), which are sexually undifferentiated progenitors of sperm and oocytes, temporarily arise during embryogenesis. In mammals, PGCs are specified in the epiblast, a pluripotent simple epithelium forming the whole embryo, at the onset of gastrulation, and temporarily show a transcriptome profile quite similar to the early mesoderm in response to the signaling that specifies the mesoderm fate of the surrounding somatic cells, but swiftly suppress the mesoderm-like program by a transcriptional repressor BLIMP1, followed by the acquisition of the germ cell program, including re-acquisition of pluripotent markers and repression of *Hox* genes. Epigenome reprogramming of PGCs, including genome-wide erasure of DNA cytosine methylation, drastic switch of repressive histone marks, relocation of active enhancers, commences accompanying this dynamic change of transcriptional program. This reprogramming of genome-wide epigenetic features contributes to the gametogenesis, which starts at the onset of gonadal sex differentiation. I will talk about our recent findings on the molecular dynamics and functions of PGC reprogramming. (COI:No)

### SY9-3

**Elucidation of the mechanisms underlying Leydig cell differentiation**

Yuichi Shima[1], Kentaro Suzuki[2] ([1]*Dept Anat, Kawasaki Med Sch,* [2]*Dept Dev Genet, Inst Adv Med, Wakayama Med Univ*)

Leydig cells are the primary source of androgens, and it is well accepted that two distinct Leydig cell populations, fetal Leydig cells (FLCs) and adult Leydig cells (ALCs), sequentially emerge in the prenatal and postnatal testis, respectively.

An orphan nuclear receptor, NR5A1 (also known as Ad4BP or SF-1), is essential for functional differentiation of both FLCs and ALCs. We previously identified an FLC-specific enhancer (FLE) of the *Nr5a1* gene, and produced the mice in which FLE was deleted. FLE deletion resulted in complete inhibition of *Nr5a1* gene expression in FLCs and these mice demonstrated defective testosterone production and absence of masculinization at fetal stages.

NR5A1 also plays a pivotal role in pituitary gonadotrope differentiation. We previously identified a pituitary-specific enhancer (PE) of the *Nr5a1* gene and deleted this region from the mouse genome. Although PE deletion mice showed normal masculinization at fetal periods, adult male mice had small testes and the prostate and seminal vesicles were disappeared. These results suggested that FLC development is independent of pituitary regulation, while ALC differentiation is solely dependent on pituitary. (COI:No)

### SY9-4

**Effects of Early Life Stress on growth and development of male reproductive system- from the viewpoint of Developmental Origins of Health and Disease(DOHaD)**

Hidenobu Miyaso[1], Kaiya Takano[1], Kenta Nagahori[1], Zhong-Lian Li[1], Takuya Omotehara[1], Shinichi Kawata[1], Miyuki Kuramasu[1], Xi Wu[1], Yuki Ogawa[1], Masahiro Ito[1] ([1]*Department of Anatomy, Tokyo Medical University*)

Developmental origins of health and disease (DOHaD) postulates that environmental factors during early life stages are related to occurrence of various diseases later. Recently we particularly have focused on the early life stress (ELS), i.e., physiological and psychological stresses soon after birth. In male ICR mice, neonatal maternal separation (NMS), a model of ELS, was performed for 0.5, 1, and 2 hours/day, from postnatal day (PND) 1 to 10. At 10 weeks of age, NMS mice exhibited decrease in Sertoli cell number. The termination of Sertoli cell proliferation in pre-puberty can be induced by p27, a cyclin-dependent kinase inhibitor. We observed increase in p27-positive Sertoli cell and decrease in Sertoli cell numbers by NMS at PND 10 and 16, respectively. ELS reportedly induced secretion of cortisol/corticosterone, stress hormones, which result in health injures. In our studies, NMS caused decrease in serum corticosterone level. Administration of corticosterone during PND 1 to 10 induced elevation in p27-positive Sertoli cell and attenuation in Sertoli cell numbers. Our data suggest that corticosterone induced by ELS yields adverse effects on male reproductive system in adults. (COI:No)

### SY9-5

**Development of a novel method to evaluate male reproductive toxicology**

Satoshi Yokota[1] ([1]*Division of Cellular & Molecular Toxicology, Center for Biological Safety & Research, National Institute of Health Sciences*)

At least half of artificial reproductive technology inseminations are performed via intracytoplasmic sperm injection (ICSI). ICSI is mainly applied to infertile patients who suffer from severe spermatogenesis dysfunction. A single sperm is selected under stereomicroscope to inject into the oocyte. However, sperm taken from such patient cannot work well in the infertility therapy. There is limitation to clarify the cause of the failure because intervention trial cannot be easily performed in human. Instead, a mechanism of male reproductive dysfunction is needed to be elucidated using rodent model. In this session, we report the effects of long-term intake of vitamin A excess (VAE) on mouse spermatogenesis. Spermatogenesis is tightly orchestrated, with tubules periodically cycling through 12 epithelial stages in mice. Surprisingly, distribution of stages of seminiferous tubule in VAE mice are significantly changed and subsequently, increased abnormal sperm head morphology is observed in VAE mice compared with wild-type. Recently, we are trying to develop a novel method to detect abnormality of sperm morphology highly sensitive. (COI:No)

### SY9-6

**Regulation of capacitation and fertilization by serotonin and GABA**

Masakatsu Fujinoki[1] ([1]*Res Lab Lab Anim, Res Cent Lab Anim, Comp Res Facil Adv Med Sci, Sch Med, Dokkyo Med Univ*)

Mammalian sperm have to be capacitated before fertilization. Capacitated sperm show hyperactivation at flagellum and acrosome reaction at head. Hyperactivation is a specialized motility to pass through envelopes of the oocyte. Acrosome reaction is an exocytosis to digest envelopes of the oocyte. Serotonin and GABA are released to the oviduct in order to mature the oocyte and capacitate sperm. In hamster sperm, serotonin induced hyperactivation through 5-$HT_2$ and 5-$HT_4$ receptor, and GABA reduced serotonin-induced hyperactivation associated with 5-$HT_2$ receptor. In mouse sperm, serotonin induced hyperactivation through 5-$HT_2$, 5-$HT_3$, 5-$HT_4$ and 5-$HT_7$ receptors. Moreover, GABA induced hyperactivation. In rat sperm, serotonin induced hyperactivation through 5-$HT_4$ receptor, although GABA did not affect hyperactivation. It was investigated that ability of hyperactivation positively correlates to the success of IVF. In mice, serotonin significantly increased the success of IVF, although GABA did not affect it. These results suggest that serotonin regulates sperm capacitation and fertilization, and GABA regulates only sperm capacitation. (COI:No)

### Symposium10

### Nuclear envelope biology - the structure and function of nuclear envelope, aging and disease caused by its abnormalities

(March 28, Sun. 16：30〜18：30, Room6)

### SY10-1
**Molecular mechanisms of lamin mutation-induced cardiomyopathy**

Issei Komuro[1] ([1]*Dept Cardiovasc Med, Grad Sch Med, The Univ of Tokyo*)

Mutations of lamin, a nuclear membrane protein, cause various diseases such as progeroid syndromes, lipodystrophy, muscle dystrophy and cardiomyopathy. We have examined responsible genes of dilated cardiomyopathy and found that the most frequent mutated gene is titin and the second is lamin. There were big differences between patients with two mutations in responses to drug treatment, lethal arrhythmia and death, and the prognosis of patients with lamin mutation was worse. There were remarkable nuclear deformity and DNA damage in cardiomyocytes of patients with lamin mutation, knock-in mice and differentiated from patient-derived iPS cells. Single cell RNA sequence analysis revealed that cardiomyocytes differentiated from patient-derived iPS cells were immature. Screening of binding proteins revealed that there were many nuclear proteins bound to mutated lamin proteins but not to normal lamin proteins. We screened small molecules which reduced DNA damage in cardiomyocytes differentiated from patient-derived iPS cells and found that a small molecule is an activator of the transcription factor which bound to mutated lamin proteins. (COI:No)

### SY10-2
**Regulations of cellular senescence and tumorigenesis through nuclear envelope stress response mediated by ER-resident transcription factor OASIS.**

Atsushi Saito[1], Tetsuro Yoshimaru[2], Yosuke Matsushita[2], Toyomasa Katagiri[2], Kazunori Imaizumi[1] ([1]*Dept. Biochem., Grad. Sch. Biomed. Health. Sci., Hiroshima Univ.*, [2]*Div. Genome. Med., Inst. Advanced. Med. Sci., Tokushima Univ.*)

Nuclear envelope (NE) comprises double lipid bilayer membranes and nuclear lamina. NE damages is often induced by DNA damages and mechanical strength associated with cellular migration, resulting in disruption of lamina and disorder of functional interactions of NE proteins. These dysfunctions are called "NE stress", which is mentioned the involvement in DNA instabilities, cellular senescence and tumorigenesis.

We found endoplasmic reticulum (ER)-resident transcription factor OASIS specifically accumulates at damaged NE regions and can activate in response to NE stress at an early stage. The activated OASIS induced senescence marker p21, consequently promoting cellular senescence, known as a defense system against tumorigenesis by attenuating cell proliferation. The expression of OASIS was attenuated in several cancer cell-lines including human glioblastoma U251MG due to hypermethylation of its promoter. Xenograft experiments using U251MG stably expressing OASIS showed the inhibited tumorigenesis and high senescent status. Taken together, OASIS is an initial responsive factor for NE stress response and acts as a tumor suppressor through regulation of cellular senescence. (COI:Properly Declared)

### SY10-3
**Change of nuclear envelope structure during cellular senescence and its association with age-related diseases**

Akiko Takahashi[1] ([1]*Proj Cell Senes, Can Inst, JFCR*)

Cellular senescence is the state of essentially irreversible cell cycle arrest that can be induced by permanent DNA damage signaling and is therefore considered to act as an important tumor suppression mechanism. Recently, it has been revealed that senescent cells are accumulated during the aging process *in vivo* and secrete many inflammatory proteins and small extracellular vesicles. This phenotype, termed the senescence-associated secretory phenotype (SASP), contributes to numerous age-related pathologies. We have previously reported that the epigenetic de-regulation and cytoplasmic accumulation of nuclear DNA fragments causes the aberrant activation of cGAS-STING cytoplasmic DNA sensors, promoting the onset of SASP. However, the molecular mechanism of chromosomal DNA fragmentation and the origin of nucleotide ligands for DNA sensors in senescent cells remains unclear. We have tried to observe the change of nuclear envelope structure during cellular senescence and analyze the association with SASP induction in age-related diseases such as cancer. Our research goal is to reveal the molecular mechanism and biological roles of SASP, leading to regulate age-related diseases. (COI:No)

### SY10-4
***Lamin* mutations and mechanoresponsive transcription factors**

Atsushi Kubo[1], Toshihiko Ogura[1] ([1]*IDAC, Tohoku Univ*)

Incompleteness of the nuclear envelope has a significant impact on the maintenance of skeletal muscle homeostasis, as mutations in Lamin or Emerin can cause muscular dystrophy. On the other hand, mechanical stress also plays a crucial role in skeletal muscle, as evidenced by the fact that exercise is the only effective method for preventing and treating disuse muscle atrophy. Mechanical stress applied to cells activates various pathways, some of which are transmitted to the cell nucleus. Indeed, a number of transcription factors have been identified that change their subcellular localization from the cytoplasm to the nucleus in response to mechanical stress. Surprisingly, it has been reported that mutations or deletion of Lamin A results in attenuated signal transduction to the nucleus, suggesting that the integrity of the nuclear envelope is closely related to the mechanical response of the cell. In this presentation, I will discuss how the nuclear envelope is involved in tissue homeostasis and differentiation, with a focus on mechanoresponsive genes. I will also introduce a new system for analyzing the molecular mechanisms of exercise in skeletal muscle. (COI:Properly Declared)

### SY10-5
**Skeletal muscle involvement in murine models of nuclear envelopathies**

Eiji Wada[1], Yukiko Hayashi[1] ([1]*Department of Pathophysiology, Tokyo Medical University, Japan*)

Mutations in the genes encoding nuclear envelope proteins, emerin and lamin A/C, cause myopathy called Emery-Dreifuss muscular dystrophy (EDMD). Since there was no suitable model for nuclear envelope myopathy existed, the underlying molecular mechanism of skeletal muscle involvement in EDMD has not been clarified. Recently, we generated double mutant (Emd-/-/Lmna$^{H222P/H222P}$; EH) mice to elucidate roles of interactions between emerin and lamin A/C in skeletal and cardiac muscles. Both Emd and H222P mice had a very mild phenotype in skeletal muscle; however, EH mice showed the progression of muscular dystrophy before appearance of cardiac dysfunction similar to EDMD patients. Both H222P and EH mice had similar cardiac abnormalities at 30 weeks of age which indicates that emerin has been implicated to contribute to skeletal or cardiac muscle in a manner different to that of lamin A/C. The disease progression is different in H222P and EH mice; however, abnormal nuclei were present in both cardiac and skeletal muscles from H222P and EH mice. These results indicate that emerin may have important roles to suppress skeletal muscle involvement other than preservation of nuclear shape. (COI:No)

### Symposium11

### Schwannoma/demyelination and immune cells: Elucidation of pathogenic mechanism and search for therapeutic strategies

(March 28, Sun. 16：30～18：30, Room7)

### SY11-1

**Schwann cell development, maturation and regeneration: a focus on emerging signaling pathways in neuron-glial cross-talk**

Valerio Magnaghi[1], Veronica Bonalume[1] ([1]*Dept. of Biomolecular and Pharmacological Sciences, University of Milan*)

In the PNS, Schwann cells (SC) physio-pathologic states are differently regulated by a plethora of emerging factors, including hormones, growth factors and neurotransmitters (e.g. purine, Ach, GABA), thus activating entire intracellular pathways, such as protein-kinases or Erk/MAPK. In this light, SC are part of a bidirectional neuron-glial cross-talk. Indeed, SC express GABA receptors, synthetize and release GABA, as well as the neurosteroid allopregnanolone (ALLO), which in turn specifically controls the SC proliferation, differentiation and myelination.
Recently, we identified novel mechanisms, involving GABA-A receptor in axons. Endogenous GABA regulates the physiological desensitization of nociceptors during sustained firing. Moreover, ALLO exerts dual actions on the C-fibers excitability. Acutely ALLO sensitizes axonal GABA-A, while at later stages, it activates a paracrine mechanism in DRG neurons, involving BDNF-trk-b and PKC $\varepsilon$ up-regulation, which leads to GABA-A desensitization.
In conclusion, we suggest that SCs are actively involved also in the local regulation of peripheral nociceptive signaling within nerves, via the modulation of axonal GABA-A receptors. (COI:No)

### SY11-2

**Immortalized Schwann cells as useful tools for therapeutic approaches to peripheral nerve injury and intractable neuropathies**

Kazunori Sango[1] ([1]*Diabetic Neuropathy Project, Tokyo Metropolitan Institute of Medical Science*)

As glial cells in the peripheral nervous system, Schwann cells play a key role in functional maintenance of neurons and axonal regeneration after injury. Schwann cell disorders can be causes of various kinds of intractable neuropathies. We have established spontaneously immortalized Schwann cell lines IMS32 and IFRS1 from adult ICR mice and Fischer 344 rats, respectively. IMS32 cells have been employed to investigate the action mechanisms of axonal regeneration-promoting molecules (e.g. ciliary neurotrophic factor and galectin-1) and the polyol pathway hyperactivity and other pathogenic factors of diabetic neuropathy. IFRS1 cells have shown the capability to myelinate neurites in co-culture with adult rat dorsal root ganglion neurons, and the co-culture system has been utilized to study the action mechanisms of myelination-promoting molecules (e.g. soluble neuregulin-1 type III and extending-4) and the pathogenesis of amiodarone-induced demyelinating neuropathy. These Schwann cell lines can be valuable tools for exploring pathobiology of axonal degeneration and regeneration, and novel therapeutic approaches toward neurological disorders in patients with relevant diseases. (COI:No)

### SY11-3

**C-C chemokine receptor $2^+$ macrophages in nerves ameliorate motor neuron disease model mice**

Ryo Yamasaki[1], Wataru Shiraishi[1] ([1]*Dept Neurol, Kyushu Univ, Fukuoka, Japan*)

Macrophages expressing C-C chemokine receptor type 2 (CCR2) infiltrate into the neural tissues of amyotrophic lateral sclerosis (ALS) patients. We reported an association between intrathecal upregulation of C-C chemokine ligand 2 (CCL2) and disease severity in ALS. We investigated the roles of $CCR2^+$ macrophages in ALS using *mutant Cu/Zn superoxide dismutase* $1^{G93A}$ (*mSOD1*)-transgenic (Tg) mice.
In *mSOD1*-Tg mice, mSOD1 accumulated in the sciatic nerve earlier than in the spinal cord. Before disease onset, $CCR2^+$ macrophages harboring mSOD1 infiltrated the sciatic nerve much earlier than in the lumbar cord. CCR2-deficient *mSOD*-Tg mice showed an earlier onset of disease and axonal derangement in the sciatic nerve compared with CCR2-positive *mSOD1*-Tg mice. CCR2-deficient *mSOD1*-Tg mice had a marked increase in deposited mSOD1 in the sciatic nerve compared with CCR2-positive mice together with a decreased infiltration of $CCR2^+$ macrophages. These findings suggest $CCR2^+$ macrophages recruited into the peripheral nerves exert neuroprotective functions in mSOD1 ALS. Clearance of mSOD1 from the peripheral nerve by $CCR2^+$ macrophages is a hitherto-underestimated host protective mechanism. (COI:No)

### SY11-4

**Extracellular proteases in myelination and demyelination**

Shigetaka Yoshida[1], Yoshio Bando[2] ([1]*Department of Functional Anatomy and Neuroscience, Asahikawa Med University,* [2]*Department of Anatomy, Akita University Graduate School of Medicine, Akita, Japan*)

KLK6 and KLK8 are extracellular serine proteases mainly expressed in the central nervous system (CNS). KLK8 is constitutively expressed in the neurons of some of the limbic system regions. CNS lesions including demyelination in experimental allergic encephalitis (EAE) induce KLK8 in oligodendrocytes. In EAE, oligodendrocytes around the demyelination with infiltrating cells express KLK8. EAE was induce in KLK8-knockout and wild-type mice and knockout mice showed milder symptoms and less demyelination in the spinal cord. KLK6 is constitutively expressed in mature oligodendrocytes. CNS impairment such as injury and demyelination induce stronger expression of KLK6 in oligodendrocytes. KLK6-knockout mice did not show major dysfunction of oligodendrocytes and myelin. However, in EAE, KLK6-knockout mice showed milder behavioral symptom and less infiltration of inflammatory cells in the spinal cord. Metalloproteinase 9 can be activated by KLK6 and may be a key molecule of demyelination. KLK6 and KLK8 can share substrates so further analysis using double knockout mice will elucidate importance of proteases in demyelination. (COI:No)

### SY11-5

**Dysfunction hippocampal oligodendrocytes in the mouse model for posttraumatic stress disorder**

Shozo Jinno[1] ([1]*Grad. Sch. Med. Sci. Kyushu Univ.*)

Posttraumatic stress disorder (PTSD) is a devastating mental illness that develops after a traumatic event. Although recent imaging studies have reported white matter impairments in the brains of PTSD individuals, the details remain elucidated. In this study, we examined the potential involvement of oligodendrocyte dysfunction in the pathophysiology of PTSD, using contextual fear conditioning in mice. The lower myelin coverages were found in the axons of parvalbumin-immunoreactive (PV+) neurons but not in excitatory neurons in the hippocampus of PTSD models compared to controls. The spatial densities of oligodendrocyte precursor cells (OPCs) and oligodendrocytes (OLs) in the hippocampus were lower in PTSD models than in controls. Administration of benztropine, which was shown to enhance OPC differentiation, increased the myelin coverage of axons of PV+ neurons in the hippocampus and reduced the PTSD-related behaviors. Finally, chemogenetic activation of PV+ neurons in the hippocampus also ameliorated the PTSD-related behaviors. These results indicate that dysfunction of OLs related to PV+ neurons in the hippocampus may underlie the pathophysiology of PTSD. (COI:No)

### SY11-6

**Protective effect of molecular hydrogen against ischemia-reperfusion injury in the optic nerve**

Mami Noda[1] ([1]*Kyushu University, Graduate School of Pharmaceutical Sciences, Japan*)

The central nervous system (CNS) white matter ischemia is an important clinical problem and may produce injury, in part, by reactive oxygen species (ROS)-induced mitochondrial dysfunction.. Using the mouse optic nerve as a white matter model, the effect of molecular hydrogen ($H_2$) was tested by quantitatively monitoring the area of compound action potential (CAP) in vitro. A 60 min period of oxygen and glucose deprivation (OGD) caused prompt loss of the CAP followed by an average 20% recovery. After 10-14 days of oral administration of $H_2$, the isolated optic nerves showed that the CAP area did not disappear during ischemia and recovered to a significantly great extent during reperfusion. On the other hand, $H_2$ supplementation in the perfusing solution during the CAP measurement did not show significant protection. Immunostaining of axonal neurofilament also showed significant protection by previous intake of $H_2$. Accumulation of nuclear 8-oxoguanine (8-oxoG), a marker of oxidative DNA damage, was observed mainly in oligodendrocytes after OGD, which was significantly attenuated by previous intake of $H_2$. Perspectives on the action of $H_2$ how to prevent oxidative stress will be discussed. (COI:No)

### Symposium12

### Cellular system for responses against environmental changes in epidermis

(March 29, Mon. 9：00～11：00, Room5)

### SY12-1

**Environmental changes regulate human epidermal stem cell dynamics**

Daisuke Nanba[1] ([1]*Medical Research Institute*)

Adult stem cells are responsible for tissue homeostasis and repair in vivo, and can also maintain their remarkable potentials ex vivo. Hence, controlling and manipulating stem cell behavior promises the advances in stem cell-based regenerative medicine. Adult stem cells reside in the specific microenvironment to maintain their properties. This environment, called "niche", composes of nursing cells, extracellular matrix and growth factors, and affects the behavior of adult stem cells. In addition, physical parameters of the niche, including matrix stiffness, oxygen concentration and temperature, can be also considered as regulating factors of stem cell behavior. The epidermis, the outermost layer of the skin, is localized at the interface between the body and the external environment. The epidermal keratinocyte stem cells play crucial roles in epidermal homeostasis, and adapt to external temperature changes due to their location. In this study, we demonstrate the alteration of human epidermal keratinocyte stem cell dynamics in response to temperature changes. (COI:No)

### SY12-2

**Pathophysiological role of primary cilia in skin immune disease**

Manami Toriyama[1,2,3], Defri Rizaldy[1,2,4], Motoki Nakamura[5], Fumitaka Fujita[1,2,6], Fumihiro Okada[1,6], Akimichi Morita[5], Ken Ishii[2,7,8] ([1]*Dep. Pharm. Sci., Osaka Uni.*, [2]*CVAR., NIBIOHN*, [3]*Dep. Biosci., NAIST*, [4]*Dep. Pharm., Institute Tek. Bandung*, [5]*Grad. Sch. Med. Sci., Nagoya city Uni.ni.*, [6]*Mandom Corp.*, [7]*iFRec., Osaka Uni.*, [8]*Ins. Med. Sci. The Uni. of Tokyo.*)

Atopic dermatitis (AD) is common allergic eczema caused by unknown factors. Immune cells in epidermis, including Langerhans cells (LCs) contribute to the pathogenesis of AD, however, their regulation mechanism in disease have yet to be explored. Here, we show that human dendritic cells (DCs) and LCs have primary cilia-like structure. The primary cilia assembling during DC proliferation by Th2 cytokine, GM-CSF was shut off by DC maturation agents, suggesting the role of primary cilia to transduce proliferation signaling. Platelet-derived growth factor receptor A (PDGFR $\alpha$) pathway, one of proliferation signal transduced in primary cilia, promoted DC proliferation in a dependent manner of intra-flagellar transport (IFT) system. In epidermis from AD patients, aberrant ciliated LCs and keratinocytes (KCs) with showing immature and proliferating state were observed. Our results identify the potential role of primary cilia in allergic inflammation and skin barrier disorder, and suggest primary cilia regulation as therapeutic targets in AD. (COI:Properly Declared)

### SY12-3

**Adaptive evolution of stratum corneum function in mammalian skin epidermis**

Takeshi Matsui[1] ([1]*RIKEN-IMS, Japan*)

About 360 million years ago, first terrestrial vertebrates, amphibians emerged from water and adapted to life on land. These animals evolved their skin epidermis and acquired stratum corneum (SC), the uppermost dead cell layer which provides protective barrier function. Later on, ancestral mammals changed the SC to be soft and moisturized. Previously, we reported that evolutionary acquired mammalian skin-specific retroviral-like aspartic protease SASPase was the key regulator of SC moisturization. It cleaves SC-barrier-related protein, profilaggrin to produce filaggrin monomer in the lower SC. As the optimum pH of SASPase protease activity was pH5.77, we hypothesized that lower SC has an acidic environment. Intravital imaging of mouse SC-pH revealed that SG1 cells undergo intracellular acidification at the initial stage of cornification. This finding collectively indicates that the lower SC has an environment to activate SASPase protease. Thus, co-option (exaptation) of retroviral element-derived SASPase gene function is suggested to be achieved under the acidic lower SC at the emergence of ancient mammals. (COI:No)

### SY12-4

**Innate immune response against SARS-CoV-2**

Hiroyuki Oshiumi[1] ([1]*Dep Immunol, Grad Sch Med Sci, Kumamoto Univ*)

The innate immune system is a first line of defense against viral infection. Viral RNAs are recognizes by pattern recognition receptors. RIG-I and MDA5 are pattern recognition receptors and sense cytoplasmic viral RNAs, such as influenza A, Sendai virus, and hepatitis C virus. SARS-CoV-2 causes COVID-19 pandemic; however, the innate immune response against SARS-CoV-2 is still unclear. We found that both RIG-I and MDA5 recognized cytoplasmic viral RNAs of SARS-CoV-2 and that the two viral RNA regions close to its 3' UTR were preferentially recognized by RIG-I and MDA5, resulting in the type I IFN expression. Interestingly, several viral non-structural proteins suppressed RIG-I- and MDA5-mediated signaling. Moreover, the Riplet ubiquitin ligase suppressed SARS-CoV-2 replication. Riplet is an E3 ubiquitin ligase and mediates K63-linked polyubiquitination of RIG-I, thereby inducing type I IFN expression. Collectively, our data indicate that Riplet-mediated RIG-I activation is crucial for the innate immune response against SARS-CoV-2 although the virus has the ability to suppress the response. (COI:No)

### SY12-5

**Influences of environmental changes on nociceptors on skin surface**

Fumitaka Fujita[1] ([1]*Mandom corp, Depr Grad Pharm, Osaka Univ*)

Twenty years ago, we never imagined that one molecule could sense temperature and sensory irritations on the skin surface. In 1997, it was discovered that an ion channel called TRPV1 responded to not only high temperature but also capsaicin, which dramatically changed understanding of sensory perception on skin. We have clarified that TRPA1, which was found in 2003, is related to sensory irritation by various chemicals and environmental changes.

Aluminum compounds have several effect to human including anti-inflammation. For clarification of aluminum effect, we focused on thermo-sensitive TRP channels because we already found alumminum ion activates hTRPM4. TRP channels expressed in sensory neuron were evaluated whether aluminum ion affect their activity. In calcium imaging experiments showed aluminum ion blocked hTRPV1 and hTRPA1 activities. Futhermore, even at the neutral pH 7.4 aluminum potassium sulfate inhibited hTRPV1 and hTRPA1. These results suggested that aluminum play a role as analgesic agent at ion state, suggesting aluminum compound used as vaccine adjuvant might have the analgesic role at vaccination. (COI:No)

### Symposium13

### Physiological and pathophysiological importance of ion channel complex in muscle cells

(March 29, Mon. 9：00～11：00, Room6)

### SY13-1

**Mechanism and treatment of RyR1-related skeletal muscle diseases**

Takashi Murayama[1] ([1]*Dept Pharmacol, Juntendo Univ Sch Med*)

Type 1 ryanodine receptor (RyR1) is a $Ca^{2+}$ release channel in the sarcoplasmic reticulum and plays an important role in excitation-contraction coupling. Genetic mutations in RyR1 cause various skeletal muscle diseases including malignant hyperthermia (MH) and central core disease. Since the main underlying mechanism of MH is overactive $Ca^{2+}$-induced $Ca^{2+}$ release (CICR) by gain-of-function of the RyR1 channel, inhibition of CICR is expected to be a promising treatment for these diseases. We have developed a novel high-throughput screening platform using time-lapse fluorescence measurement of $Ca^{2+}$ in the endoplasmic reticulum to identify RyR1 inhibitors. By screening of a chemical compound library and subsequent structure expansion, we successfully developed Compound 1 as a novel RyR1-selective inhibitor. Compound 1 effectively prevented or reversed the fulminant MH crisis by isoflurane anesthesia and heat stroke in mice carrying MH mutations. Thus, Compound 1 has the potential to be a promising new candidate for effective treatment of patients carrying RyR1 mutations. (COI:No)

### SY13-2

**Junctophilins regulate formation of functional coupling between sarcolemmal and sarcoplasmic reticulum $Ca^{2+}$channels in striated muscles.**

Tsutomu Nakada[1], Hiroyuki Kawagishi[2], Takuro Tomita[2], Mitsuhiko Yamada[2] ([1]*Dept Instr Anal, Res Ctr Sup Adv Sci, Shinshu Univ.*, [2]*Dept Mol Pharmacol, Shinshu Univ Sch of Med.*)

In striated muscles, L-type calcium channels (LTCCs) form a functional complex with ryanodine receptors (RyRs) at junctional membrane (JM) where the sarcolemma is closely apposed to sarcoplasmic reticulum (SR) membranes. Junctophilins (JPs) stabilize the JM by bridging the sarcolemmal and SR membranes. We reported that expression of JP1 mutant lacking its C-terminal transmembrane domain in mouse skeletal muscles significantly reduced their contractile force without disrupting JM, revealing a novel role of JP1 to directly support the LTCC-RyR1 interaction. Thus, we next examined by using an analogous JP2 mutant (JP2Δ427), a role of JP2 in cardiac myocytes where LTCCs regulate RyR2 not directly but indirectly through $Ca^{2+}$-induced $Ca^{2+}$ release (CICR). Nonetheless, JP2Δ427 also significantly reduced the fractional shortening of the left ventricle without causing overt heart failure. Interestingly, it recruited LTCCs to the surface sarcolemma from T-tubules. Therefore, JP2 supports the cardiac muscle contraction by aligning LTCC and RyR2 within an adequate distance for CICR. In this symposium, we will discuss how JPs regulates excitation-contraction coupling of striated muscles. (COI:No)

### SY13-3

**Physiological role of TRPC channel complexes in cardiac dynamics**

Yohei Yamaguchi[1,2], Keiji Naruse[2], Gentaro Iribe[1,2] ([1]*Dept Physiol, Asahikawa Med Univ, Hokkaido, Japan*, [2]*Dept Cardio Physiol, Okayama Univ, Okayama, Japan*)

When a cardiomyocyte is held in a stretched position, the stretch induces a biphasic twitch force enhancement. The short-term stretch rapidly augments its twitch force, accelerating an increase in myofilament $Ca^{2+}$ sensitivity, called as the Frank-Starling mechanism (FSM). A further long-term stretch over several minutes causes an increase in $[Ca^{2+}]_i$, leading to a slow force response to stretch (SFR), a further increase in the twitch force. The activation of angiotensin II type 1 receptor (AT1R) has been implicated in the biphasic response; however, the downstream pathway is not clearly understood. Here, we investigated the detailed pathway, focusing on TRPC3 and TRPC6, receptor-operated non-selective cation channels, using our developed force-length control device. We found that TRPC3 and TRPC6, regulated by AT1R via diacylglycerol produced by phospholipase C, play a pivotal role in the SFR. In addition, our data interestingly show that TRPC6 stabilizes the twitch force enhancement in FSM. These findings suggest that AT1R-TRPC channel complexes could operate the stretch-induced biphasic twitch force response. (COI:No)

### SY13-4

**Regulation of myocardial atrophy by TRPC3-Nox2 complex formation**

Yuri Kato[1], Kazuhiro Nishiyama[1], Tomohiro Tanaka[2], Akiyuki Nishimura[2], Motohiro Nishida[1,2] ([1]*Dept. Physiol., Grad. Sch. Pharm., Kyushu Univ.*, [2]*Natl. Inst. Physiol. Sci., Explo. Res. Ctr. Life Living Syst., Div. Card.*)

Cardiac plasticity, caused by mechanical loading and unloading, is a major clinical outcome of heart failure. Especially, myocardial atrophy, characterized by the decreases in size and contractility of cardiomyocytes, is reportedly caused by severe malnutrition and/or mechanical unloading. We investigated the mechanism underlying induction of myocardial atrophy by anti-cancer drug treatment, and found that formation of protein signaling complex between transient receptor potential canonical (TRPC) 3 and NADPH oxidase 2 (Nox2) contributed to ROS-mediated myocardial atrophy in mice. We revealed that extracellular ATP mediated nutrient deficiency-induced cardiomyocyte atrophy through TRPC3-Nox2 complex formation. We screened an already approved drug that was able to inhibit TRPC3-Nox2 complex formation, and found that ibudilast, a bronchodilator, potently disrupted the TRPC3-Nox2 complex formation and myocardial and skeletal muscle atrophy in doxorubicin-treated mice. These results suggest that the formation of TRPC3-Nox2 protein complex will become a new therapeutic target of amyotrophic intractable diseases. (COI:No)

### SY13-5

**Role of a mechanosensitive ion channel PIEZO1 in skeletal muscle regeneration**

Yuji Hara[1], Kotaro Hirano[1], Yasuo Mori[1], Masato Umeda[1,2] ([1]*Graduate School of Engineering, Kyoto University, Japan*, [2]*HOLO BIO Co., Ltd*)

Skeletal muscle has the capacity to regenerate myofibers after muscle injury. Upon a variety of stimuli including mechanical stretch, muscle-resident stem cells called muscle satellite cells (MuSCs) are committed to become myoblasts that can fuse with each other to generated multinucleated myotubes. We previously reported that transbilayer relocation of phosphatidylserine is essential for morphogenesis of myotubes through the function of PIEZO1, a $Ca^{2+}$ mechanosensitive cation channel that is activated by membrane tension. However, the molecular entity that determines the cell fate of MuSCs remains to be elucidated. In this session, we will present our recent data showing that PIEZO1 plays crucial roles in activation of MuSCs through activation of an intracellular signaling pathway. Our results will provide insights into cell fate decision of MuSCs in a manner dependent on mechanosensation through mechanosensitive ion channels. (COI:No)

### Symposium14

### Reconsider the nerve furcation patterns based on the studies of macroscopic anatomy, embryology, and physiology

(March 29, Mon. 9：00～11：00, Room7)

### SY14-1

**The comparative anatomy of the branching pattern of the medial branches of the dorsal rami of the spinal nerves**

Yuko Fuse[1,2], Konosuke Tokita[1], Ryuhei Kojima[1], Yukio Aizawa[3], Katsuji Kumaki[3], Ikuo Kageyama[3], Eishi Hirasaki[4] (*[1]Dept. of physical therapy, Fac. of Health and Medical Care, Saitama Med. Univ., [2]Amakusa Rehabilitation Hosp., [3]Dept. of Anatomy, Sch. of Life Dent Niigata, The Nippon Dental Univ., [4]Primate Research Institute, Kyoto Univ.*)

The purpose of this study is to elucidate the branching pattern of the medial branches of the dorsal rami of the spinal nerves and the structures on the spinalis and transversospinalis muscles (Tr) in mammals. Two human cadavers, one Japanese macaque, one fetal pig and one rat were studied. Five to ten Tr which were attached to the same spinal process of the vertebrae were observed. The medial branches were divided into the medial cutaneous and muscular branches. The muscular branches distributed into the spinalis and the semispinalis, then divided into the multifidus and the rotator. The Tr bifurcated into the multifidus and the rotator. The intertransverse muscles developed in the segment which had no medial cutaneous branches. Regarding the semispinalis; there were two muscular branches divided into the multifidus and the intertransverse. The Tr of the rat had two bundles at each spinal process. All the thoracolumbar segment in the rat had no medial cutaneous branches. The branching pattern of the muscular branch was similar to those of the fetal pig. According to these results, the number of the medial cutaneous branches were increased corresponding to the muscle augmentation. (COI:No)

### SY14-2

**The morphological relationship between the branching pattern of the cervical nerves and the cervical muscles**

Saori Anetai[1,2], Ikuo Kageyama[3], Yukio Aizawa[3], Ryuhei Kojima[2], Katsuji Kumaki[3], Eiji Hirasaki[4], Hideki Endo[1] (*[1]Grad Sch Agri and Life Sci, Univ Tokyo, Tokyo, Japan, [2]Dept Phys Ther, Fac Health and Med Care, Saitama Med Univ, Saitama, Japan, [3]Dept Anat, Sch Life Dent at Niigata, The Nippon Dent Univ, Niigata, Japan, [4]Primate Res Inst, Kyoto Univ, Aichi, Japan*)

The dorsal shoulder girdle muscles(DSG) and dorsal scalenus muscles(DS) are innervated by nerves arising from the most dorsal layer of the ventral rami of the cervical nerves. In the present study, the morphology of these muscles and their innervations were macroscopically investigated in several primates (1 humans, 1 chimpanzee, 2 common squirrel monkey and 2 red-handed tamarin). Interspecies discrepancy in running of the innervating branch of the DSG(nDSG) were observed. In humans and chimpanzee, the nDSG ran on the ventral surface of the DS or passed through the DS. In common squirrel monkey and red-handed tamarin, C4-C5 nDSG ran on the dorsal surface of the DS. In addition, each ventral ramus of C4-C6 branched out from the nDSG and an innervating branch of the ventral layer of the DS from their roots. The ventral ramus arising from C7 branched out with three rami innervating dorsal layer of the DS, DSG and ventral layer of the DS from its root. On the basis of these findings, morphogenesis of the DSG and DS was considered. This work was supported by the Cooperative Research Program of the Primate Research Institute of Kyoto University. (COI:No)

### SY14-3

**A novel view of the spinal nerve branching pattern from embryonic mesodermal lineages.**

Shunsaku Homma[1], Takako Shimada[1], Katsuji Kumaki[2], Katsuki Mukaigasa[1], Noboru Sato[2], Hiroyuki Yaginuma[1] (*[1]Dept. Neuroanat. Embryol., Fukushima Med. Univ., Fukushima, JAPAN, [2]Div. Gross Anat. Morphogenesis, Niigata Univ. Grad. Sch. Med. Dent. Sci., Niigata, JAPAN*)

The musculoskeletal system of the vertebrate body develops exclusively either in the sclerotome-derived connective tissue environment (primaxial domain) or in the lateral plate (abaxial domain), into which the muscle progenitor cells invade from the myotome. However, the traditional epaxial-hypaxial muscle classification, based on the adult innervation pattern of the dorsal-ventral rami of the spinal nerve, does not correspond to the embryonic primaxial-abaxial distinction; a single intercostal nerve (ventral ramus) innervates the muscles in both primaxial and abaxial domains. For this reason, we took a deductive approach, by developing a model of the spinal nerve branching pattern in harmony with the innervation pattern, which is compatible with mesodermal lineages. Implementing our model into human gross anatomy provides reasoning for the innervation pattern of the spinal nerve. The embryonic branching pattern of the intercostal nerve in mice is consistent with our model but is later modified into the adult form. We believe that our model provides a novel framework and evolutionary insight for understanding the spinal nerve innervation pattern and body wall organization. (COI:No)

### SY14-4

**Actin reorganization and plasma membrane trafficking in three-dimensional space of nerve growth cones**

Motohiro Nozumi[1], Michihiro Igarashi[1] (*[1]Department of Neurochemistry & Molecular Cell Biology, School of Medicine, and Graduate School of Medical and Dental Sciences Niigata University, Niigata, JAPAN*)

We are missing various interactions between intracellular structures in the dynamic living circumstance from the optical limit of the observation. Neuronal growth cones are the F-actin-enriched highly motile structures at the tip of extending axons in the developing or the regenerating neurons. We recently revealed, using super-resolution microscopy (SIM), that local endocytosis is associated with the F-actin-bundling at the leading edge of growth cones. The endocytosis most likely contributes to the membrane retrieval, containing the lipid rafts from the nonadherent surface in the growth cone (Cell Rep 2017). Using 3D-SIM, we also found that F-actin bundles were extended toward not only the leading edge but also the nonadherent surface. An axon guidance receptor neuropilin-1 was transiently concentrated in the filopodia. Endocytic components were also accumulated in the filopodia when they retract. These results suggest that the formation of nonadherent filopodia promotes the neuropilin-1-associated local endocytosis effectively. We will discuss new findings over the optical limit using super-resolution microscopy. (COI:No)

### Symposium15

### Brain development and environment - The involvement of endocrine system

(March 29, Mon. 14：20〜16：20, Room3)

### SY15-1

**The effects of nuclear corepressors in thyroid hormone action on brain development**

Izuki Amano[1], Ayane Ninomiya[1], Megan Ritter[2], Kristen R. Vella[2], Anthony N. Hollenberg[2], Noriyuki Koibuchi[1] ([1]*Department of Integrative Physiology Gunma University Graduate School of Medicine, Gunma, Japan,* [2]*Division of Endocrinology, Weill Cornell Medicine, New York, NY, USA*)

The nuclear corepressor 1 (NCoR1) and the silencing mediator of retinoid and thyroid hormone receptors (SMRT) are essential coregulators of the thyroid hormone receptor (TR), mediating transcriptional repression via histone deacetylation. Thyroid hormone (TH) plays an essential role in many physiological processes via the TR. How the corepressors regulate TR signaling is not fully understood, especially in the brain. To understand the role of NCoR1 and SMRT in the brain, we used mice with conditional NCoR1 ($NCoR1^{lox/lox}$) and SMRT ($SMRT^{lox/lox}$) alleles in combination with mice that express *Cre* recombinase in a neuronal specific fashion (Snap25-Cre). Global deletion of NCoR1 or SMRT during embryogenesis results in lethality. We also showed that NCoR1/SMRT double knock-out mice die within two weeks after induction of Cre activity in adult mice. Now, we found that neuronal specific NCoR1 or SMRT KO mice survive without obvious impairment of neuronal development. However, NCoR1/SMRT double knock-out mice die within postnatal 1-2 weeks and have impaired body growth. Thus, both NCoR1 and SMRT have important roles in maintaining normal neuronal function. (COI:No)

### SY15-2

**The effect of nighttime lighting on brain development and its molecular mechanism**

Shogo Haraguchi[1] ([1]*Dept Biochem, Showa Univ Sch Med*)

Nighttime lighting affects brain development in vertebrates. In brain development, a natural light-dark cycle promotes better brain development than constant conditions, such as constant light. However, little is known about the molecular mechanisms through which environmental light conditions affect brain development.

We recently demonstrated that the pineal gland, a photosensitive gland in the brain, synthetizes various neurosteroids in birds, which are diurnal animals like humans. Pineal allopregnanolone, a metabolite of progesterone, prevents neuronal apoptosis in the developing cerebellum. We found that pineal allopregnanolone synthesis during nighttime is higher in the pineal glands of birds that are housed in natural light-dark cycle conditions compared to birds that are housed in light-at-night conditions. Furthermore, the number of cerebellar Purkinje cells was decreased in birds housed at light-at-night conditions. The decrease in Purkinje cell number was rescued by allopregnanolone injection. We, therefore, suggest that pineal allopregnanolone is a critical metabolite that affects cerebellar development in vertebrates, depending on the environmental light conditions. (COI:No)

### SY15-3

**Neonatal exposure to estrogen causes irreversible infertility via specific suppressive action on hypothalamic *Kiss1* neurons in male and female rodents**

Shiori Minabe[1,2], Naoko Inoue[2], Yoshihisa Uenoyama[2], Hitoshi Ozawa[1], Hiroko Tsukamura[2] ([1]*Dept. Anat. Neurobiol., Grad. Sch. Med., Nippon Med. Sch., Tokyo, Japan,* [2]*Grad. Sch. Bioagr. Sci., Nagoya Univ., Nagoya, Aichi, Japan*)

Aberrant exposure to environmental estrogen during the critical developmental period may cause improper hypothalamic programming, thus resulting in reproductive dysfunction in adulthood. Kisspeptin (encoded by *Kiss1* gene) is a key player in reproduction via direct stimulation of GnRH and then gonadotropin release in mammalian species. Neonatal estradiol-benzoate (EB) administration caused abnormal spermatogenesis and a decrease in plasma testosterone in adulthood in male rats. The EB-treated female rats showed persistent vaginal diestrus in adulthood. The rats showed profound suppression of pulsatile luteinizing hormone (LH) release and *Kiss1*/kisspeptin expression in the arcuate nucleus (ARC) even after gonadectomy in adulthood. Furthermore, neonatal estrogen may act through both estrogen receptor (ER) α and ERβ, because EB exposure significantly reduced the number of ARC *Kiss1*-expressing cells in wild-type mice but not in ERα or ERβ knockout mice. Taken together, long-term exposure to estrogen in the developing brain causes defects in ARC kisspeptin neurons via ERα and ERβ, resulting in inhibition of LH pulses and consequent infertility in both male and female rodents. (COI:No)

### SY15-4

**Effects of the developmental environment on mice behaviors under group-housing conditions**

Nozomi Endo[1], Mayumi Nishi[1] ([1]*Department of Anatomy and Cell Biology*)

Conventional behavioural examinations using rodent models to investigate the effect of developmental environment were conducted under simplified artificial conditions for a short observational period. However, social context plays a critical role in both the aetiology and expression of psychopathology in humans. Analysis of animal behaviours in an environment with a social context is essential. In order to address this point, we developed a behavioural analysis system which can identify multiple mice in a group-housing environment and continuously localize each mouse for several days. Then, we applied our newly system to analysis for mice subjected to social isolation after weaning or repeated maternal separation. We found that social isolation mice exhibited longer social distance and needed more time until huddle with cagemates than control mice. In addition, maternal separation mice showed impairment of social proximity and alteration of approach preferences to others. Focussing on behavioural abnormalities in an environment with a social context will be important insights to understand the neuronal circuitry underlying neurodevelopmental and neuropsychiatric disorders. (COI:No)

### Symposium16

### The mouth is the gate of happiness! - Common factors between Oral Senses and Whole body -

(March 29, Mon. 14：20～16：20, Room4)

### SY16-1

**A variety of physiological roles of acid-sensing ion channels, generators of acidosis-related pain**

Shinya Ugawa[1], Yasuhiro Shibata[1], Natsuko Kumamoto[1], Takashi Ueda[1] (*[1]Department of Anatomy and Neuroscience, Graduate School of Medical Sciences, Nagoya City University*)

The acid-sensing ion channel (ASIC) family is a major branch of the mechanosensory degenerin/epithelial sodium channel superfamily of ion channels. Four ASIC genes (from ASIC1 to ASIC4) have been identified in mammalian organisms so far. Individual ASIC proteins (ASICs) are subunits that associate as homo- or hetero-trimers to form amiloride-blockable proton-gated cation channels in the central and peripheral nervous systems. ASICs are known to be involved in a wide range of neuronal functions, such as synaptic plasticity, mechanotransduction, nociception linked to local tissue acidosis, and sour-taste sensation. We previously reported that ASIC1b is expressed in inner hair cells (IHCs) and outer hair cells (OHCs) of the mouse cochlea. To explore the *in vivo* functional roles of ASIC1b in the auditory system, we generated conventional ASIC1b knockout mice, measured their auditory brainstem responses and distortion product otoacoustic emissions, and recorded proton- and voltage-gated currents in both IHCs and OHCs. In this presentation, we will show you our data from the experiments after a brief introduction of ASICs expressed in human trigeminal ganglion neurons. (COI:No)

### SY16-2

**Why do we feel a "toothache" with nerve-free dentin: Sensory transduction mechanism in dentinal sensitivity based on the special architecture of dentin-pulp complex**

Yoshiyuki Shibukawa[1] (*[1]Dept. Physiol., Tokyo Dent. Coll.*)

When enamel is removed to expose dentin following enamel loss, dentin is extremely sensitive to various stimuli applied on dentin surface; cold (thermal stimulation), scrapping/drilling dentin (mechanical stimulation), and sour/sweet tastants (low-pH/hypertonic stimuli). This produces unendurable pain on tooth as "dentinal sensitivity", often resulting in "dentin hypersensitivity". Dentin is permeated by dentinal tubules which contain dentinal fluid. Volume changes in the fluid by multiple stimuli applied on dentin surface induce changing in hydrodynamic force inside tubules which results in outward movement of dentinal fluid. This movements provide mechanical effects of tubules at dentin-pulp border where odontoblasts are located. In this symposium, we would like to discuss new model to describe dentinal sensitivity, as referred to "odontoblast hydrodynamic receptor theory". Mechano-sensory transduction in odontoblasts mediates intercellular odontoblast-neuron signal transduction, underlying sensory generation mechanism of dentinal pain. We also discuss results in drug discoveries for dentin regeneration/formation, which provide next strategy for clinical application in dentistry. (COI:Properly Declared)

### SY16-3

**The role and modulation mechanism of WASABI receptor TRPA1 in the sensation of pain**

Yi Dai[1,2], Koichi Noguchi[2] (*[1]Dept Pharm, Sch Pharm, Hyogo Univ Health Sci, [2]Dept Anat & Neurosci, Hyogo Col Med*)

TRPA1 is a member of the transient receptor potential (TRP) channel family, which can be activated by multiple stimuli such as chemical, thermal ( ≦ 18 ℃), and mechanical stimuli. Allyl isothiocyanate, the main pungent component in WASABI can activate TRPA1 to generate acrid sensation in the mouth, as well as pain sensation in the skin. Besides WASABI, the TRPA1 can detect a broad range of chemicals, including garlic, wintergreen oil, clove oil, ginger, and cinnamon oil, all of which induce acute painful burning or pricking sensation and spicy taste.

TRPA1 is expressed by nociceptors and is critically involved in nociception. We have demonstrated that inflammatory mediators include bradykinin and trypsin/tryptase can sensitize TRPA1 channels through posttranslational regulation; this regulation involved intracellular signals such as PKA and PLC-mediated PIP2 hydrolysis. Recently, we discovered an intracellular energy sensor, AMPK, can negatively regulate TRPA1, which may serve as a potential mechanism of painful diabetic neuropathy.

This presentation will review the role and modulation mechanism of TRPA1 in the sensation of pain, which has been demonstrated by our lab and others. (COI:No)

### SY16-4

**Mechanism of sensory transmission from oro-facial tissues; morphology and function of trigeminal nociceptors**

Hiroyuki Ichikawa[1], Takehiro Yajima[1], Tadasu Sato[1] (*[1]Div Oral Craniofac, Grad Sch Dent, Tohoku Univ*)

Primary nociceptors have small to medium-sized cell bodies in the trigeminal ganglion (TG). In this study, immunohistochemistry for calcitonin gene-related peptide (CGRP) and other substances was performed to investigate the morphology and nature of TG nociceptors. CGRP-positive TG neurons innervating the facial skin and tooth pulp were mainly small and medium-sized, respectively. Brain-derived neurotrophic factor (BDNF) was a transmitter candidate for many TG neurons innervating the facial skin. However, BDNF-containing TG neurons were rare in the tooth pulp. By analysis about ion channels, TG neurons responding to heat (> 43℃), capsaicin and proton were abundant in the facial skin whereas those responding to heat (> 52℃) and mechanical stimulus were common in the tooth pulp. In addition, noxious stimulation to the facial skin induced expression of c-Fos in the trigeminal subnucleus caudalis. After tooth pulp stimulation, c-Fos expression was common in the subnuclei caudalis and oralis. The present study indicates that the cell size of TG nociceptors is associated with their projection sites, and contents of neurotransmitters and sensor proteins. (COI:No)

### Symposium17

### Expanding Sleep Research - The Next Decade -

(March 29, Mon. 14：20～16：20, Room5)

### SY17-1

**Cellular basis for redistributing synaptic strengths amongst neighbors sharing the dendrites**

Yukiko Goda[1] ([1]*RIKEN Center for Brain Science*)

Synaptic plasticity is crucial for how the brain perceives the environment, learns and stores memories, and rebalances during sleep. Yet, despite the wealth of knowledge on synaptic plasticity mechanisms in general, how synaptic strength changes at a given synapse influence the strengths of neighboring synapses, which in turn shape dendritic integration properties, are not well understood. In particular, how presynaptic strengths of neighboring synapses are altered by local activity have received little attention. We sought to gain insights into the basic cellular rules underlying local synaptic cross-talk in hippocampal pyramidal neurons, by examining both pre and postsynaptic strength changes at neighboring synapses upon eliciting plasticity at target synapses. We find that long-term potentiation spreads along the dendrite to neighboring spines whose polarity is dependent on the distance from target synapses. At nearby pre-synapses, plasticity is limited to depression of neurotransmitter release. Notably, the polarity of heterosynaptic plasticity is not necessarily matched between the pre and the postsynaptic sides at individual synapses. (COI:No)

### SY17-2

**A neuronal circuit induces a hibernation-like state in mice**

Tohru Takahashi[1] ([1]*WPI-IIIS, Grad Sch Med, Tsukuba Univ, Tsukuba, Japan*)

Hibernation is considered to be one of the survival strategies against lack of energy resource, owing to much reduced energy expenditure. Hibernating animals show robust inactive, seemingly similar to sleep, however, they do reduce body temperature and metabolic rate, which never seen in homeothermic animals even during sleep. Although numerous studies suggest that the brain plays a key role in regulation of hibernation, we are still far from a complete understanding of neuronal pathways involved.

To quest the neuronal mechanism, we utilized laboratory mice (*Mus Musculus*), non-hibernating mammals, since they have the rich genetic resources. We identified a unique subset of a neuronal population, which can induce a long-lasting hypothermic and hypometabolic state. These neurons-'Q neurons'-are along the most anterior part of the third ventricle of the hypothalamus-a part of the preoptic area.

We designated this state to be QIH (Q neurons-induced hypometabolism) and concluded that it is a hibernation-like hypometabolic state, because QIH shares several key features with hibernation. Our finding could make advance in understanding of the neuronal circuit relevant to hibernation.

(COI:No)

### SY17-3

**Tackling the mystery of sleep slow waves from the claustrum**

Kimiya Narikiyo[1] ([1]*Department of Anatomy, Faculty of Medicine, Toho University*)

The appearance of slow waves in electroencephalogram is a physiological hallmark of sleep state. Slow waves derive mainly from the silencing of many neurons in the cerebral cortex for about 200 milliseconds at a time, but underlying neuronal mechanisms and its functions remain largely unclear. Recently, we found that the brain region called the claustrum is involved in the generation of slow waves. The claustrum is a thin sheet-like neural structure that locates beneath the insular cortex and has reciprocal projection with a wide area of the cerebral cortex. We investigated the relationship between the claustrum and cortical slow waves using neuroanatomical, electrophysiological, and optogenetic methods. It was found that the firing activity of the claustrum neurons correlated with cortical slow waves, that the claustrum activation selectively drove firing activity of inhibitory interneurons in the cerebral cortex, and that the activity of inhibitory interneurons silenced many cortical neurons for about 200 milliseconds at a time and induced a slow wave. Based on the findings, various hypotheses regarding mechanisms and functions of the sleep slow waves will be discussed. (COI:No)

### SY17-4

**Neural regulatory mechanism of sleep/wakefulness by the hypothalamic neurons**

Akihiro Yamanaka[1,2], Hiroto Ito[1,2] ([1]*Department of Neuroscience II, Research Institute of Environmental Medicine, Nagoya University,* [2]*JST CREST*)

Recent development of new optical tools to manipulate activity of neurons or to measure activity of neurons revealed neural regulatory mechanism of sleep/wakefulness by hypothalamic neurons. We focused on peptide-producing neurons such as orexin/hypocretin-producing neurons and melanin concentrating hormone (MCH)-producing neurons. Optogenetical activation of MCH neurons converted non-rapid eye movement (NREM) sleep to REM sleep. And in vivo activity recording at single cell resolution using micro endoscope revealed that MCH neurons are classified to three types, that is wake-active, REM-active and wake and REM-active type MCH neurons. State dependent inhibition of MCH neurons revealed that REM-active MCH neurons are involved in erasure of memory during REM sleep. We applied these techniques to orexin neurons and confirmed that orexin neurons are active in wakefulness. However, these neurons also showed weak activity in NREM sleep. This activity might have a role in the regulation of NREM sleep since narcolepsy model mice showed fragmentation of sleep and wakefulness. We will discuss about these recent results in this symposium. (COI:No)

### SY17-5

**Intracellular signals in the regulation of sleep/wakefulness**

Hiromasa Funato[1,2] ([1]*Dept Anat, Sch Med, Toho Univ,* [2]*IIIS, Univ Tsukuba*)

Sleep is regulated in a homeostatic manner. Sleep deprivation increases sleep need, which is compensated mainly by increased non-rapid eye movement (NREM) sleep delta (0.5-4 Hz) power and by increased sleep time. However, how the molecular and cellular mechanism executes the homeostatic regulation of sleep remains unknown. We found that a protein kinase SIK3 plays a crucial role in determining sleep need. The loss of a well-conserved PKA site and alanine-substitution of the serine residue increased NREM sleep time and NREM sleep delta power. Other members of SIK family, SIK1 and SIK2, also have the S551-equivalent sites. The mice that have the alanine-substitution of the S551-equivalent sites showed increased sleep need but to a lesser extent than SIK3 (S551A). In vitro analysis showed that the PKA signaling altered kinase activity and downstream transcription. Thus, SIK signaling may constitute intracellular signaling regulating sleep need. (COI:No)

### Symposium18

### New Approach for Electron Beam-based Organelle-imaging

(March 29, Mon. 14：20～16：20, Room7)

### SY18-1

**Large-scale electron microscopy and imaging big data**

Yosky Kataoka[1,2], Yasuhisa Tamura[1,2], Masanori Yamato[1,2], Asami Eguchi[1,2], Kumi Takata[1,2], Toshiyuki Goto[1], Mitsuyo Maeda[1,2] (*[1]RIKEN BDR, [2]RIKEN-JEOL Collaboration Center*)

We have developed an automatic acquisition system for large-scale electron microscopic images that can provide microstructure information including organelles in wide areas (>1 mm$^2$) of biological tissues at high spatial resolutions. The system consists of automatic and sequential acquisition of a huge number of images in scanning electron microscopy (SEM) followed by automatic tiling of acquired images. The technology brings about imaging big data on cells and organelles. Furthermore, we have made an imaging viewer that provides a straightforward graphical user interface to visualize such large-scale images at the gigabyte level. This imaging research platform has been used by many researchers. Recently, we are challenging automatic segmentation of microstructures in large-scale images using machine learning technologies. The study will realize comprehensive morphological analysis called "Micro-morphomics", one of omics analyses of imaging. Automatic or semiautomatic analysis of such imaging big data will allow us to discover novel biological events that occur under aging or pathophysiological conditions.
COI: Authors belong to RIKEN-JEOL Collaboration Center (COI:Properly Declared)

### SY18-2

**The analysis for the interaction between the influx of mitochondria and microglial activation in the axon initial segment of injured motor neuron**

Hiromi Tamada[1], Sumiko Kiryu[1], Sohgo Sawada[1], Hiroshi Kiyama[1] (*[1]Grad. Sch. Med., Nagoya Univ.*)

The axon initial segment (AIS) is structurally and functionally characteristic compared with other axonal regions. In this study, the alteration of mitochondrial localization in AIS and the association of activated microglia with the AIS of motor neurons in response to nerve injury were analyzed with the Focused Ion Beam / Scanning Electron Microscopy (FIB/SEM). The huge number of mitochondrial accumulations were observed in AIS in response to axon transection, whereas very few, if any, mitochondria were seen in normal AIS. FIB/SEM analysis also demonstrated that axon-injured AIS in which numerous mitochondria existed was surrounded by microglia. When the microglial activation was suppressed with an inhibitor such as minocycline, the mitochondrial accumulation was not observed even in axon-injured AIS. Taken these results into consideration, it is likely that nerve-injury activated microglia adhering to AIS affect structural changes of AIS in response to nerve injury, and thereby mitochondrial influx into AIS is allowed. (COI:No)

### SY18-3

**Ultrastructural analysis on the process of isolation membrane formation during piecemeal mitophagy**

Ritsuko Arai[1], Shunichi Yamashita[2], Tatsuya Sugisaki[1], Wu Huajui[1], Tomotake Kanki[2], Satoshi Waguri[1] (*[1]Department of Anatomy and Histology, Fukushima Medical University, School of Medicine, [2]Department of Cellular Physiology, Graduate School of Medical and Dental Sciences, Niigata University*)

In a type of mitophagy that is induced by an uncoupler CCCP, mitochondrial fragmentation precedes enwrapment by the isolation membrane (IM). On the other hand, a piecemeal mitophagy induced by iron chelator DFP, involves pinching off a small portion of the mitochondrial network by IM (Yamashita et al., J. Cell Biol., 2016). However, detailed processes at electron microscopic levels remain elusive. By correlative light and electron microscopy (CLEM), we found that LC3-positive IMs tightly attached along a surface of 'bud-like' projection of mitochondria in DFP-treated HeLa cells. Moreover, a mitophagy receptor BNIP3 was associated with such mitophagy profiles. Electron tomography in samples prepared with a fixative of aldehyde-osmium mixture revealed the presence of IM-associated tubules (IMATs), elements of the ER, and vesicles near the rim of IMs. These findings suggest that in DFP-induced mitophagy the extension of IM occurs on a target surface labeled with mitophagy receptors, and that this mechanism contributes to the direct segregation of a portion of the mitochondrial network. (COI:No)

### SY18-4

**Electron microscopic analyses of neuronal synapses and mitochondria modulated by postnatal environment**

Yoshiaki Shinohara[1], Nobuhiko Ohno[1] (*[1]Department of Anatomy, Division of Histology and Cell Biology Jichi Medical University*)

Since neuronal organelles play critical roles for brain functions, quantitative analyses of the organelle greatly promote understanding of neuronal physiology. We reared rats in enriched environment for 4 weeks and found that gamma oscillations of the right hippocampus are greater in magnitude than the left side. Histological exanimations also showed that synapse densities of the CA1 pyramidal neurons are more numerous on the right side. Electron microscopic detection of the synapses indicated more greater right/left ratios than light microscopy of VGAT immunostaining followed by fluorescent puncta counting, probably due to the limitation of the spatial resolution of the optical microscope. Though organelle detection under electron microscopy (EM) is powerful methodology, this method has several drawbacks as follow:
1) Narrow areas measured under EM are prone to produce sampling bias.
2) Researchers need training and expertise in EM.
3) Compared to fluorescent microscopy, EM is time consuming.
In this section, we discuss possible technical approaches to efficiently quantify neuronal organelles, such as synapses and mitochondria, from large volumes of EM samples. (COI:No)

### SY18-5

**Electron microscopy for the analysis of membrane lipids**

Takuma Tsuji[1], Toyoshi Fujimoto[1] (*[1]Laboratory of Molecular Cell Biology, Research Institute for Diseases of Old Age, Juntendo University Graduate School of Medicine*)

During autophagy, isolation membranes (IM) expand by incorporating phospholipids from the ER, but how those phospholipids reach both leaflets of IM has not been clear. Atg9 can translocate phospholipids across liposomal membranes *in vitro*, and thus, was proposed to function as a scramblase in IM. Consistently, we previously showed that PtdIns3P is present in both leaflets of yeast autophagosomal membranes, but whether major phospholipids, e.g., PtdCho and PtdSer, distribute similarly, and if so, how quickly such distribution is achieved have not been known. In the present study, we examined distribution of PtdCho, PtdSer, and PtdIns4P by quick-freezing and freeze-fracture labeling electron microscopy, which can define phospholipids distribution in individual leaflets, and found that all those phospholipids distribute in both leaflets of autophagosomes. Moreover, by metabolic labeling of *de novo* synthesized PtdCho, symmetrical distribution in autophagosomes was found to be attained within 30 min after synthesis, without dispersing to the other membranes. The result indicates that transbilayer phospholipid transport, most likely mediated by Atg9, occurs in autophagosomal membranes. (COI:No)

### SY18-6

**Novel procedure for visualizing specific neural circuit information by using large area correlative light and electron microscopy imaging with multi-beam scanning electron microscopy**

Shinsuke Shibata[1,2,3], Ryo Ihara[2,3], Taro Iseda[2,3], Tomoko Shindo[2], Nobuko Moritoki[2], Toshihiro Nagai[2], Hideyuki Okano[3] (*[1]Division of Microscopic Anatomy, Graduate School of Medical and Dental Sciences, Niigata University, Niigata, Japan, [2]Electron Microscope Laboratory, Keio University School of Medicine, Tokyo, Japan, [3]Department of Physiology, Keio University School of Medicine, Tokyo, Japan*)

To visualize the localization of the specific molecules in the specific neural circuitry, fluorescence labeling with the antibody or with the genetic modification was usually used. Recently we developed a large-area correlative light and electron microscopy (designated as LA-CLEM) imaging procedure with multi-beam scanning electron microscopy for biological specimens. In this symposium, we are going to demonstrate some application of our LA-CLEM imaging for detecting neural circuit information in detail, which enabled us to carry out the large area observation at the resolution of electron microscopy level with extraordinary high speed. The millimeter order neural tissue, labeled with several kinds of antibodies, were clearly imaged both with fluorescence microscopy and with multi-beam scanning electron microscopy in the same brain section. We hope our novel technologies would help to visualize the abnormality of the impaired neural tissue suffering from the various neuropsychiatric diseases in near future. (COI:No)

### SY18-7

**Recent advances of in-resin CLEM of Epon-embedded samples**

Isei Tanida[1] (*[1]Dept Cell Mol Neuropahol, Junetndo Grad Sch Med*)

CLEM (Correlative light-electron microscopy) is a method to correlate fluorescent images with electron microscopic images. Post-fixation with osmium tetroxide staining and the embedding of Epon are one of robust and essential treatments to preserve and visualize intracellular membranous structures during electron microscopic analyses. However these treatments can significantly diminish the fluorescent intensity of most fluorescent proteins in cells. We found a far-red fluorescent protein retains fluorescence after osmium staining, and achieved Epon embedding to perform an in-resin CLEM of both the Golgi apparatus and the endoplasmic reticulum as well as mitochondria in Epon-embedded samples using this fluorescent protein. In this symposium, recent advances of in-resin CLEM of Epon-embedded samples will be reported. We will show new probes including other alternative fluorescent proteins and achievement of multicolor and 3D in-resin CLEM of Epon-embedded samples. (COI:No)

### Symposium19

### Advances in secretion science - Challenges of morphology and physiology

(March 29, Mon. 16：30～18：30, Room4)

### SY19-1

**History and progress of secretory science**

Takahiro Sato[1], Masayasu Kojima[1] ([1]*Division of Molecular Genetics, Institute of Life Science, Kurume University*)

In the process of evolution, organisms have been able to transmit information between cells and tissues by acquiring various secretions and developing various secretion modes. Therefore, the discovery of secretion products and the proposal of new secretion modes broaden the scope of application of secretory science and greatly improve the understanding of biological functions. Secretory phenomena can be broadly classified into endocrine and exocrine, but recent discoveries of secretion products and secretory modes are emerging new concepts of secretory science. For example, the discovery of ghrelin revealed the existence of biosynthesis by fatty acid transferases. In addition, elucidation of secretory patterns, that is, secretory mode that transmit information bidirectionally between epithelial cells and secretory mode that transmit information between endocrine and exocrine systems, has led to the discovery of new biological functions. This lecture summarizes the history of secretory science and recent advances in secretory science, and outlines this symposium. (COI:No)

### SY19-2

**Search for novel bioactive peptides and study of secretion patterns**

Takanori Ida[1] ([1]*Frontier, University of Miyazaki*)

G-protein-coupled receptors (GPCRs) constitute a large protein superfamily that shares a 7-transmembrane motif as a common structure. Human genome sequencing has identified several hundred orphan GPCRs for which ligands have not yet been identified. GPCRs play crucial roles in cell-to-cell communication involved in a variety of secretion phenomena and are the most common target of pharmaceutical drugs. Therefore, the identification of endogenous ligands for orphan GPCRs will lead to clarification of novel physiological regulatory mechanisms and potentially facilitate the development of new GPCR-targeted therapeutics. But in the last 10 years there has been little discovery of novel bioactive peptides for orphan receptors. Recently, we have discovered novel bioactive peptides for orphan GPCRs in model organisms such as Drosophila and C. elegans. New physiological functions have been revealed from the secretory patterns of these peptides. The goal is to apply it to mammals in the future. (COI:No)

### SY19-3

**Three-dimensional imaging techniques to elucidate the morphological organization of secretory cells**

Daisuke Koga[1], Satoshi Kusumi[2], Tsuyoshi Watanabe[1] ([1]*Department of Microscopic Anatomy and Cell Biology, Asahikawa Medical University,* [2]*Division of Morphological Sciences, Kagoshima University Graduate School of Medical and Dental Sciences*)

The osmium maceration method enables direct observation of the 3D ultrastructure of membranous organelles by scanning electron microscopy (SEM) without reconstruction. Using the maceration method, we have revealed the morphological diversity of secretory cells (endocrine and exocrine cells), which have well-developed organelles involved in secretion such as the Golgi apparatus and endoplasmic reticulum. However, it is difficult to obtain the entire 3D shape of these organelles because freeze-cracked surfaces of tissues are observed in the maceration method. To overcome this problem, we have developed a novel 3D imaging technique termed "serial section SEM". This novel technique reveals the full 3D shape of organelles in secretory cells by 3D reconstruction of backscattered electron images of serial ultrathin tissue sections embedded in resin. In this symposium, the 3D shape of the Golgi apparatus in both endocrine and exocrine cells is introduced (endocrine cells: pituitary endocrine cells; exocrine cells: pancreatic acinar cells and gastric chief cells), and the relationship between the spatial architecture of the Golgi and secretory polarity is also presented. (COI:No)

### SY19-4

**Tight junction organizes exocrine systems**

Hiroo Tanaka[1,2,3], Atsushi Tamura[1,2,3], Sachiko Tsukita[2,3] ([1]*Department of Pharmacology, School of Medicine, Teikyo University, Tokyo, Japan.,* [2]*Strategic Innovation and Research Center, Teikyo University, Tokyo, Japan.,* [3]*Laboratory of Barriology and Cell Biology, Graduate School of Frontier Biosciences, Osaka University, Osaka, Japan.*)

Exocrine systems for sweat, gastric acid, bile and intestinal juice control metabolism and protect our body from infections to maintain homeostasis. Secretion of exocrine substances depends on epithelial cells which interact with each other to form exocrine glands. Accumulating evidences indicate that the tight junction (TJ), a specific cell-cell adhesion apparatus of epithelial cells, functions as a paracellular barrier to prevent back-flow of the exocrine substances from apical to basal sides of exocrine glands. As a typical example, we reveal that reflux of gastric acid due to defects in the paracellular barrier induces gastritis and gastric tumorigenesis.
On the other hand, it has become clear that paracellular channels formed in TJs permeate water, ions and/or small solutes to organize various biological systems. We show that in liver, paracellular channel functions of TJs provide a system for water and $Na^+$ to generate directional bile flow.
We will discuss recent reports that paracellular barrier and channel functions of TJs are spatiotemporally regulated to organize exocrine systems. (COI:No)

### SY19-5

**Secretion plays an important role for the communication between the blood and brain in the circumventricular organs, called "windows on the brain"**

Shoko Takemura [1], Ayami Isonishi[1], Tatsuhide Tanaka[1], Yoko Tatsumi[1], Akio Wanaka[1] ([1]*Department of Anatomy and Neuroscience, Nara Medical University*)

Circumventricular organs (CVOs) are "windows on the brain" that lack the typical blood-brain barrier. In some of the CVOs, median eminence and neurophypophysis, neurohormones are secreted from axonal terminals into the circulation. We demonstrated that continuous angiogenesis occurred in the CVOs. Structural reorganization of neuron, glia, and vasculature occurs in response to increased demands for neurosecretion. Another CVOs, the subfornical organ (SFO), rapidly secretes proinflammatory cytokines including interleukin-1β (IL-1β) in response to peripherally injected lipopolysaccharides (LPS), and repeated LPS injection attenuates IL-1β production. We found that perivascular macrophages secretes IL-1β after systemic LPS administration. When tolerance developed to LPS-induced sickness behavior in mice, the SFO perivascular macrophages ceased producing IL-1β, although peripherally injected LPS reached the SFO perivascular space. In summary, "secretion" enables the CVOs appropriate communication between the blood and brain. (COI:No)

### SY19-6

**Discovery of a novel neurosecretory factor and its biological functions**

Kazuyoshi Ukena[1] ([1]*Laboratory of Neurometabolism, Graduate School of Integrated Sciences for Life, Hiroshima University*)

We have recently identified a novel cDNA encoding a previously unknown small protein of 80 amino acid residues. Because the predicted C-terminal amino acids of the small protein were Gly-Leu-$NH_2$, the small protein was named neurosecretory protein GL (NPGL).
Subcutaneous and intracerebroventricular infusions of NPGL increased body mass gain in chicks, suggesting a central role for this protein in regulating growth and energy homeostasis. A database search revealed that the *Npgl* gene is conserved in vertebrates, including rats and mice. Using protein administration, neutralizing antibody administration, and gene overexpression in rats, we established that NPGL increased lipid accumulation in white adipose tissue (WAT). This adiposity was associated with an induction of *de novo* lipogenesis in WAT but not in liver. *Npgl* mRNA expression was upregulated by fasting and low insulin levels. Additionally, NPGL-producing cells were responsive to insulin. These results point to NPGL as a novel neurosecretory regulator that drives fat deposition through *de novo* lipogenesis in WAT and acts to maintain steady-state fat deposition in concert with insulin. (COI:No)

### SY19-7

**The possibility of novel secretory science**

Tomoya Nakamachi[1] ([1]*Faculty of Science, Academic Assembly, University of Toyama*)

Endocrine and exocrine systems have been studied as important mechanisms for maintaining homeostasis, as a concept of secretory science based on the direction of bioactive substances and fluid secreted from cells. Many endocrine and exocrine factors have been identified, and the regulatory mechanisms of endocrine and exocrine have been elucidated. The author's group revealed that one of the neurotransmitters, pituitary adenylate cyclase-activated polypeptide (PACAP) act as an important regulator of exocrine secretion in the lacrimal glands, sweat glands, and salivary glands. On the other hand, we recently found that PACAP is present in tears and is involved in the corneal repair. These data means that PACAP may have both endocrine factor and exocrine factor properties. Therefore, by summarizing these findings and the contents of other presentations in this symposium, I would like to discuss the possibility of novel secretory science that transcends the boundaries of endocrine and exocrine fields. (COI:No)

### Symposium20

### New development of Lymphology based on Anatomy and Physiology

(March 29, Mon. 16：30～18：30, Room6)

### SY20-1

**Construction of blood and lymphatic networks in 3D engineered human tissues and their functional morphogenesis**

Daisuke Okano[1], Seiji Watanabe[2], Yoshiya Asano[1], Hirokazu Narita[2], Tomohiro Chiba[2], Kazuko Tadahira[2], Hiroshi Shimoda[1,2] ([1]*Dept. of Neuroanatomy, Cell biology and Histology, Hirosaki Univ. Graduate School of Medicine,* [2]*Dept. of Anatomical Science, Hirosaki Univ. Graduate School of Medicine*)

Production of three-dimensional (3D) artificial tissues comprising human cells are required for progressive development of regenerative medicine. The 3D vital human tissues are promising not only as transplantation materials for regenerative medicine but also as a new medical research tool for development of substitute model for laboratory animals and for drug discovery. It is most important issue for development and application of 3D human tissues recreating precise anatomical structure and functional expression to introduce blood and lymphatic vasculature into the 3D tissues.
We performed a cell accumulation method using layer-by-layer (LbL) technique to construct 3D human tissues incorporating blood and/or lymphatic vascular network. The blood and/or lymphatic endothelial cells inserted into laminated fibroblasts were autonomously integrated to form distinct tubular structures, and they showed similar anatomical characteristics to blood and lymphatic capillaries *in vivo*.
Here we demonstrate the dynamics of blood and/or lymphatic vascular network formation with the underlying molecular mechanisms in the vascularized 3D human tissues and their availability for medical research. (COI:No)

### SY20-2

**Evaluation of physiological function of lymphatic vessels using animal model**

Tomomi Asaka[1], Moyuru Hayashi[1], Satoshi Uemura[2], Jun Takai[2], Takashi Moriguchi[2], Yoshiko Kawai[1] ([1]*Dept Physiol, Sch Med, Tohoku Medical and Pharmaceutical Univ,* [2]*Dept Physiol, Sch Med, Tohoku Medical and Pharmaceutical Univ*)

Lymphatic vessels are important for substance exchange as the maintenance of fluid homeostasis. Lymphatic dysfunction after lymphadenectomy in cancer therapy often causes lymphedema. The transcription factor Gata2, a zinc finger transcription factor and binds to GATA domain, is important for the lymphatic development during embryogenesis, and the responsible gene for Emberger syndrome, which exhibits lymphedema. Gata3, another GATA family, is required for LTi cell differentiation in embryogenesis and is essential for lymph node formation. Aiming at understanding the pathophysiology of lymphedema and the physiological function of GATA2 and GATA3 in the adult lymphatics, we constructed an experimental model of lymphatic recanalization after popliteal lymph node extirpation in hetero-deficient mice of Gata2 and 3. Lymphatic recanalization was impaired in Gata2-heterodeficient mice, while there were no abnormalities in Gata3 hetero-deficient mice. A certain number of Gata2-Gata3 double hetero-deficient mice showed complete lymphatic recanalization, suggesting that Gata2 disorders are partially canceled additional Gata3 deficiency. (COI:No)

### SY20-3

**Three-dimensional structure of human dermal lymphatic vessels and age-related changes**

Nao Itai[1], Haruyo Yamanishi[1], Kazuki Takagaki[1], Yasuhiro Yoshimatsu[2,3], Tetsuro Watabe[3], Kentaro Kajiya[1] ([1]*Shiseido Global Innovation Center,* [2]*Division of Pharmacology, Graduate School of Medical and Dental Sciences, Niigata University,* [3]*Department of Biochemistry, Graduate School of Medical and Dental Sciences, Tokyo Medical and Dental University [TMDU]*)

In the skin, the lymphatic system plays an important role in the maintenance of fluid homeostasis and the afferent phase of immune responses. Previously, we have reported a dramatic reduction of lymphatic vessels with age. However, the precise structure of the dermal lymphatic vessels and their molecular changes with age remained unknown. Herein, we present a three-dimensional (3D), macroscopic view of the structure of human lymphatic capillaries. The 3D images were captured by light-sheet microscopy showed that the lymphatic capillaries were widespread across the dermis, especially just below the epidermis, suggesting their potential role in epidermal homeostasis. Additionally, we revealed that human dermal lymphatics undergo endothelial-to-mesenchymal transition (EndMT) and found the dramatic increase of EndMT with age. Furthermore, our technology performs precise and continuous tomography of skin tissues, enabling us to observe fine lymphatic structures at the electron microscope level and suggesting that how waste is transported into the lymphatic vessels. Taken together, our data could provide the methods to improve cutaneous lymphatic function that deteriorates with age. (COI:Properly Declared)

### SY20-4

**Glymphatic and lymphatic pathways in human central nervous system**

Sakura Suzuki[1], Kota Tanaka[1], Reina Matsumaru[1], Tomohiro Chiba[1], Hirokazu Narita[1], Seiji Watanabe[1], Hiroshi Shimoda[1] ([1]*Dept. Anat. Sci., Grad. Sch. Med. Hirosaki Univ.*)

Recent studies have described glymphatic system as a tissue fluid clearance apparatus and meningeal lymphatic vessels in central nervous system. Both the systems have been shown in mice, but little information regarding those in humans is available. Thus, we demonstrate microanatomy of the drainage pathways in human central nervous system.
The small glial cells with expression of aquaporin4 (AQP4), a key molecule in glymphatic system, formed superficial glial limiting membrane, whereas the deep cortex exhibited AQP4-positive astrocytes extending their processes around the blood vessels. They were often contacted with each other to intermediate between both areas, and thus this might form a glymphatic pathway.
The elderly human dura mater displayed only a few lymphatics around superior sagittal sinus. However, the arachnoid tissue with expression of podoplanin, a lymphatic endothelial marker, expanded into the dura mater, and a dye injected into the subarachnoid space flew into the intradural arachnoid tissue and initial lymphatics around the spinal nerve roots. The human central nervous system, therefore, is likely to prepare a lymphatic pathway different from in mice. (COI:No)

### SY20-5

**Advances in lymphatic imaging and interventions**

Masasyoshi Yamamoto[1], Hiroshi Kondo[1], Hiroshi Oba[1] ([1]*Teikyo University school of medicine, Department of Radiology*)

The lymphatic system has been known and studied since the era of Hippocrates, but it was thought to be difficult to apply these findings to catheter procedures because of the small size of the lymphatic vessels. In 2013, Rajebi et al. reported on thoracic duct embolization using intranodal lymphangiography. Since then, various embolization techniques have been developed for lymphorrhea.
The wide variety of lymphatic diseases that we have successfully treated include chylothorax, chylous ascites, cancerous ascites, chylopericardium, chyluria, chylocorporrhea, inguinal lymphorrhea, protein loosing enteropathy, and plastic bronchitis.
There are various etiologies such as (i) traumatic injury to the lymphatic vessels, (ii) regurgitation due to lymphatic insufficiency, (iii) lymphatic obstruction due to cancer or other causes, and (iv) congestion of the entire lymphatic system due to obstruction or stenosis of the outlet. So, we try to fit our treatment to the pathological conditions based on the complex lymphatic anatomy.
In this presentation, we will present advances in lymphatic embolization based on our own experience. (COI:No)

### Symposium21

### Divergent roles of $Ca^{2+}$ signaling from organelle, cells, tissues to whole body

(March 29, Mon. 16：30～18：30, Room7)

### SY21-1

**Impact of mitochondria on local calcium release in murine sinoatrial nodal cells**

Yukari Takeda[1], Satoshi Matsuoka[1] ([1]*Department of Integrative and Systems Physiology, Faculty of Medical Sciences, University of Fukui*)

Local calcium release (LCR) from the sarcoplasmic reticulum (SR) modulates the automaticity of sinoatrial nodal cells (SNCs). We have been studying contribution of mitochondrial $Na^{+}$-$Ca^{2+}$ exchanger (NCXm) to LCR in murine SNCs. Although our previous observations indicated that NCXm-mediated mitochondrial $Ca^{2+}$ efflux contributes to $Ca^{2+}$ handling of nearby SR, spatial interrelation between LCR and mitochondria has never been clarified. In this study, two dimensional and confocal line-scan imaging of murine SNCs revealed close association between LCR and mitochondria. LCRs in the early phase of $Ca^{2+}$ transient cycle length (CL) appeared with a higher probability in close proximity to mitochondria. Occurrence of the late LCR nearby mitochondria was not significantly higher than those outside of mitochondria. Attenuation of mitochondrial $Ca^{2+}$ efflux by a NCXm inhibitor, CGP-37157, reduced the LCR amplitude and the occurrence of early LCRs, simultaneously prolonged LCR period and CL. Together, results suggested that mitochondria are involved in LCR generation by modulating SR $Ca^{2+}$ content through NCXm-mediated $Ca^{2+}$ efflux in murine SNCs. (COI:No)

### SY21-2

**Regulatory mechanism of the polarized distribution of Calcium transporter in enamel formation**

Keishi Otsu[1], Akira Inaba[1,2], Shojiro Ikezaki[1], Kazumasa Morikawa[2], Hidemitsu Harada[1] ([1]*Dept Anat, Div Dev Biol Regen Med,* [2]*Dept Oral Health Sci, Div Pediatr Spec Care Dent*)

Enamel is the most highly calcified tissue in vertebrates and the only epithelial-derived tissue that mineralized in nonpathological situations. During amelogenesis, ameloblasts show highly polarized distribution of molecules involved in protein secretion, cell adhesion and ionic transport to exert their proper function. However, the molecular mechanism remains poorly known. Our recent studies have shown that the ameloblasts function is strictly regulated by RhoA signal. Semaphorin4D-RhoA-Akt signal regulates the directional enamel matrix secretion in coordination with cell polarization. LPA6-RhoA signal also contributes to the cell polarity by the establishment of cell adhesion actin filament network. Further, LPA6 knockout mouse and dominant negative-RhoA expressing mice showed enamel hypomineralization with the loss of polarized distribution of $Ca^{2+}$ transporters, indicating that LAP6-RhoA signal regulates the localization of $Ca^{2+}$transporters. In this presentation, we will share our recent research data and discuss the regulatory mechanism of polarized distribution of $Ca^{2+}$ transporter in enamel formation, and the possibility of enamel hypomineralization caused by its disruption. (COI:No)

### SY21-3

**Role of a warm-activated cation channel TRPV4 in oral epithelial wound healing**

Reiko Yoshimoto[1], Reona Aijima[2], Yasuyoshi Osaki[1], Cao Ai-Lin[1], Gao Wei-Qi[1], Megumi Nishiyama[1], Yuko Honda[1], Kaho Uchino[1], Takeshi Sawada[1], Mizuho Kido[1] ([1]*Dept Anat & Physiol, Fac Med, Saga Univ,* [2]*Dept Oral Maxillofac Surg, Fac Med, Saga Univ*)

Wound healing is a crucial process to restore tissues, and improving wound healing resolution is a major medical priority due to the increased incidence of traumatic injury, chronic wounds, and scarring. Oral mucosal wound healing has long been regarded as an ideal wound resolution because of more rapid healing with fewer complications than cutaneous wounds. We found the faster wound closure in the oral mucosa of calcium-permeable ion channel transient receptor potential vanilloid 4 gene-deficient (TRPV4KO) mice using the palatal wound model. We observed warm temperature sensitivity of the palatal epithelial cells from WT mice, and the reaction was suppressed in those from TRPV4KO mice. The regenerating epithelia of TRPV4KO mice had wider intercellular space between the cells, and the epithelia showed intense phosphorylated myosin at the cell periphery, indicating elevated myosin contractile activity. Furthermore, the oral epithelial cells from TRPV4KO mice showed faster migration, immature cell-cell contact, and more phosphorylated myosin than those from WT mice. We can conclude that TRPV4 activity affects epithelial wound healing via regulation of migration and cell-cell contact. (COI:No)

### SY21-4

**A novel $Ca^{2+}$-dependent signaling underlying energy metabolism in mice**

Shu Nakao[1,2,4], Sakiko Matsumura[3], Shigeo Wakabayashi[1,5], Tomoe Nakamaura-Nishitani[1,3] ([1]*Department of Molecular Physiology, National Cerebral and Cardiovascular Center Research Institute, Osaka, Japan,* [2]*Department of Biomedical Sciences, College of Life Sciences, Ritsumeikan University, Shiga, Japan,* [3]*Department of Pharmacology, School of Medicine, Wakayama Medical University, Wakayama, Japan,* [4]*Ritsumeikan Global Innovation Research Organization, Ritsumeikan University, Shiga Japan,* [5]*School of Nursing, Faculty of Health Science, Osaka Aoyama University*)

Obesity is a major risk factor of life-threatening diseases, and several $Ca^{2+}$-dependent pathways are suggested to underlie obesity. Neuronal $Ca^{2+}$ sensor-1 (NCS-1) is a $Ca^{2+}$ binding protein playing important roles in excitable cell functions. We here investigated whether NCS-1-mediated pathways are crucial for obese regulation. We found that NCS-1-deficient (KO) mice showed obese with fat accumulation compared to wild-type (WT) mice. Metabolic cage analysis revealed that energy metabolism was decreased in KO mice, in agreement with the fact that mitochondrial respiration rate was decreased in KO cells. Moreover, proteins/mRNAs involved in mitochondrial thermogenesis and biosynthesis, and their $Ca^{2+}$-dependent upstream regulators were all decreased in the KO brown adipocytes, suggesting that mitochondrial respiration failure results in decreased energy expenditure in KO mice. Metabolome analysis in brown and white adipose tissues further specified altered pathways potentially causing obesity in KO mice. Taken together, these results demonstrate a mechanism of energy metabolism mediated by a $Ca^{2+}$ regulatory protein NCS-1, which could be a new therapeutic target for obesity. (COI:No)

### Symposium22

### Cutaneous sensation: Processing mechanisms and their applications

(March 30, Tue. 9：00～11：00, Room4)

### SY22-1

**Defense and survival through dynamic regulation of tactile sensitivity by the nociceptive amygdala**

Fusao Kato[1], Yukari Takahashi[1], Yae Sugimura[1] ([1]*Department of Neuroscience, Jikei University, Japan*)

Tactile allodynia, characterized by an aberrantly lowered threshold for the touch-induced withdrawal, is a hallmark symptom in various acute/chronic pain models in rodents. It has been conceived that re-organization in the dorsal horn network after injury or inflammation underlies this symptom. Evidence indicates the largest portion of the projecting neurons receiving C fiber afferents in the dorsal horn superficial layer target the parabrachial nucleus (PBN). We have demonstrated that neonatal capsaicin injection abolishes excitatory synaptic potentiation between the PBN and the central amygdala (CeA) in the spinal nerve ligation models (Ikeda et al., 2007) as well as capsaicin-induced responses in the cornea while keeping the tactile allodynia intact (Nakao et al., 2012). Surprisingly, chemogenetic excitation of central amygdala neurons alone is sufficient to induce robust tactile allodynia (Sugimoto et al., in revision). It is interpreted that the central amygdala, of which the activity is under control of various alert information such as that from the PBN, regulates the tactile sensitivity to avoid the additional risk of injury and tissue damage.

(COI:No)

### SY22-2

**Approach from morphology of molecular and functional evolution of itch**

Keiko Takanami[1] ([1]*Mouse Genomics Resource Laboratory, National Institute of Genetics*)

Itch is defined as an unpleasant sensation that elicits the desire to scratch. Vicious itch-scratch cycle causes worsen skin lesions in chronic itch. The difficulty of itch research is peripheral mediators and neural circuits have much in common with pain. About 10 years ago, gastrin-releasing peptide (GRP) and GRP receptor were discovered as an itch-specific mediator in the rodent's spinal sensory system. Then, we focused on GRP/GRP receptor as an itch neural marker in the trigeminal sensory system. We found that GRP was expressed in the small-sized primary afferents and projected to the spinal trigeminal nucleus caudalis where GRP receptor was localized in rodents. Next, we explored the molecular evolution of GRP and neural circuits through vertebrates. Bioinformatics analysis and cloning have succeeded in the isolation of orthologs of GRP in fish and Xenopus. GRP genes showed high homology with other vertebrates. Also GRP neural circuits appeared to be essentially conserved among mammals (eulipotyphla and non-human primate as well as rodents). Finally, I would like to discuss behavioral characteristics to understand the origin and significance of itch.

(COI:No)

### SY22-3

**Physiological changes in parents and infants induced by physical contact**

Sachine Yoshida[1], Hiromasa Funato[1,2] ([1]*Dept Anatomy, Faculty Med, Toho Univ,* [2]*WPI-IIIS, Univ Tsukuba*)

Caregivers not only provide food and warmth to infants but also provide safety, proximity, and emotional bonding to them through physical interactions such as hugging. We examined heart rate responses evaluated from R-R interval (RRI) in the first-year infants during a hug. We found that infants older than four months showed an increased RRI during a hug, indicating reduced heart rates and pronounced parasympathetic activity, while infants younger than four months did not. Older infants who showed few head movements immediately before hugging showed an increased RRI compared with those who showed many head movements. Younger infants showed the RRI decrease by pressurization derived from a hug, but older infants did not. Decision tree classification suggested that the RRI increase ratio can be predicted if both the infant's age and the head movement type are known. Such a context-dependent RRI change was apparent both in maternal and paternal hugs, but absent in younger infants and older infants hugged by female strangers. Parents' heart rates were reduced by hugging their infants. The parent-infant hug may begin to function as a form of communication for around four months.

(COI:No)

### SY22-4

**Atypical touch perception in individuals with autism spectrum disorder**

Makoto Wada[1,2] ([1]*Dev Disorders Sect, Dept Brain Rehab, Res Inst of NRCD,* [2]*Dept Informatics, Shizuoka Univ*)

Sensory atypicalities are known in individuals with autism spectrum disorder (ASD). In addition to hypersensitivity, atypicality in localizations of tactile stimuli also is becoming evident. In typically developing individuals, crossing arms causes reversals in the tactile temporal order judgment (Yamamoto & Kitazawa, 2001), suggesting that the tactile stimulus comes to consciousness after it is processed in spatial coordinates. In contrast, the reversal was small in ASD children (Wada et al, 2014). Meanwhile, in the cutaneous rabbit illusion paradigm, illusory perception occurs even on a stick (Miyazaki et al, 2010). We found that more than one-third of individuals with ASD avoided reporting the illusions on the stick, though they felt cutaneous rabbit illusion (Wada et al, 2020). As to touch perception outside the body, rubber hand illusion is atypical in individuals with ASD (Paton et al, 2012), and deficit in body ownership illusion was also found in mouse models of ASD (Wada et al, 2019). From these results, we hypothesize that tactile stimulus comes to consciousness at the skin coordinates in individuals with ASD, and it would be related to their sensorimotor atypicality.

(COI:No)

### SY22-5

**Tactile Sharing and Feedback Technology**

Yoshihiro Tanaka[1] ([1]*Nagoya Institute of Technology*)

Tactile sensation is subjective, depending on our bodies. First, the tactile sensation is derived from skin deformation and temperature change generated by a mechanical interaction between the skin and an object. Even for the same object, mechanical stimulation on the skin might differ among individuals due to different mechanical properties of the skin and exploratory movements. Secondly, the relationship between tactile sensations and exploratory movements is bidirectional. Tactile sensation is generated by exploratory movement, but tactile sensation also influences exploratory movement. Based on the above two aspects, we have developed a wearable skin vibration sensor that detects skin-propagated vibrations elicited by the mechanical interaction between the fingertip and an object. This sensor can collect subjective tactile sensations and has various potential applications because the tactile sensation is useful not only to recognize the contact object but also to manipulate the object and recognize the body. In this talk, I will introduce this sensor and its applications to communications, rehabilitation, and human-human/robot collaboration with shared tactile perception.

(COI:No)

### Symposium23

### Up-to date of continence medicine

(March 30, Tue. 9：00～11：00, Room8)

### SY23-1

**Physiology and pathophysiology of lower urinary tract function: overview and insights from animal models**

Minoru Miyazato[1] ([1]*Dept Syst Physiol, Grad Sch Med, Univ of Ryukyu*)

The lower urinary tract has two main functions, storage and elimination, which is under voluntary control of the spinobulbospinal reflex pathway, whereas many other visceral organs are regulated involuntary. Due to these complicated systems, lower urinary tract function is easily damaged by injuries or numerous medical diseases, resulting in overactive bladder, underactive bladder, and stress urinary incontinence. Animal models of neurogenic lower urinary tract dysfunction are available for cerebral infarction, Parkinson's disease, multiple sclerosis, spinal cord injury, diabetes, bladder outlet obstruction, and interstitial cystitis. Studies in animals for these diseases involved in the central or peripheral nervous system indicate that the lower urinary tract dysfunction is dependent on the damage of nervous system innervating lower urinary tract simply or additionally, such as cross sensitization, or plasticity of the neural pathways controlling the lower urinary tract. These animal models may help to investigate the mechanism involved in the genesis of pathological conditions as well as the plasticity in reflex pathways to the lower urinary tract after neurogenic lesions. (COI:No)

### SY23-2

**Pathophysiology of Underactive bladder (Overview)**

Naoki Aizawa[1] ([1]*Dept Pharmacol and Toxicol, Sch Med, Dokkyo Med Univ*)

Underactive bladder (UAB), which has been described as a symptom complex suggestive of detrusor underactivity (DU), is usually characterized by prolonged urination time with or without a sensation of incomplete bladder emptying, usually with hesitancy, reduced sensation on filling, and slow stream often with storage symptoms. Several causes such as aging, bladder outlet obstruction, and diabetes mellitus have been assumed to be responsible for the development of UAB. In this symposium, I will introduce several contributing factors including myogenic failure, efferent and/or afferent dysfunctions, and central nervous system dysfunction in the pathophysiology of UAB. (COI:No)

### SY23-3

**Classification and etiology of interstitial cystitis/bladder pain syndrome**

Akira Furuta[1] ([1]*Department of Urology, Jikei University School of Medicine*)

The East Asian clinical guideline for interstitial cystitis/bladder pain syndrome (IC/BPS) published in 2020 has showed that IC/BPS is divided into two groups with or without Hunner lesions such as Hunner-type IC (HIC) and BPS because histopathology and comprehensive gene expression analysis totally differ between HIC and BPS; HIC is associated with severe chronic inflammation of the bladder accompanied by lymphoplasmacytic infiltration and urothelial denudation whereas BPS shows little pathological changes in the bladder. The sever HIC has been classified as a designated intractable disease by the Japanese government in 2015.

The etiology of IC/BPS remains unknown, but it has been suggested that the causes of HIC are in the bladder and those of BPS are out of the bladder. The hypothesis causing HIC is that bladder epithelium is invaded by bacteria or viruses and suburothelial bladder afferent nerves are then activated by urine substances due to urothelial barrier failure, developing to chronic bladder pain and inflammation. On the other hand, the causes of BPS seem to be associated with pelvic organ cross-talk or central sensitization. (COI:No)

### SY23-4

**The new strategy for the treatment of refractory overactive bladder**

Tomonori Yamanishi[1], Toshihiko Kamasako[1], Kanya Kaga[1], Mayuko Kaga[1], Miki Fuse[1]
([1]*Dept Urology, Continence Center, Dokkyo Medical UNiversity*)

Overactive bladder (OAB) who are not successful to these conservative therapies more than three months are determined as refractory OAB.The new strategy for the treatment of refractory OAB includes neuromodulation and intra-detrusor injection of botulinum toxin (BOTOX). Neuromodulation includes electrical stimulation(ES), magnetic stimulation (MS), and sacral nerve neuromodulation (SNM). Sacral neuromodulation (SNM) is an implant type of neuromodulation, that uses mild electrical pulses to continuously stimulate the sacral nerves which innervate the lower urinary tract. A percutaneous nerve evaluation (PNE) of the S3 roots is recommended as a temporary screening test to determine the response to neuromodulation, and satisfactory responders are implanted with a permanent (chronic) system. Intra-detrusor injection of botulinum toxin therapy (BOTOX) has been covered by health insurance from April 2020. It has been found that botulinum toxin inhibits the release of acetylcholine from cholinergic nerves and acts on afferent nerves via chemical denervation. (COI:No)

### SY23-5

**Dynamic coordination mechanisms of the pelvic floor support by the skeletal and smooth muscle tissues**

Keiichi Akita[1], Satoru Murou[1] ([1]*Tokyo Medical and Dental University [TMDU]*)

We have examined the male and female pelvic floors macroscopically and histologically. In the male, bundles of the levator ani muscle extend to connect to the perineal muscles. The levator ani muscle and the external anal sphincter form the anterior and posterior muscular slings of the anal canal. The rectourethralis muscle laterally extends smooth muscle fibers both on the levator ani's superoposterior and inferoanterior surfaces. In the female, the anterior perineal skeletal muscles, the levator ani, and the external anal sphincter muscles connect to each other. The anterior skeletal muscular wall of the anal canal is composed of the anterior muscle bundle of the levator ani, superficial transverse perineal, and proper external anal sphincter muscles. Smooth muscle tissues of the vaginal wall laterally extend toward superior and inferior to the levator ani. According to such various connections among the skeletal muscles and the smooth muscles in the pelvic floor, these continuous muscle sheets have roles in the dynamic coordination mechanisms of the pelvic floor support in males and females. (COI:No)

### SY23-6

**Pathophysiology of urinary incontinence after radical prostatectomy and significance of pelvic floor muscle training**

Mikako Yoshida[1] ([1]*Department of Women's Health Nursing & Midwifery, Tohoku University Graduate School of Medicine*)

The main cause of urinary incontinence after radical prostatectomy is insufficient urethral closure due to urinary sphincter deficiency. Autonomic afferent denervation of the membranous urethral mucosa was seen in most patients. To close the urethra enough to prevent urinary leakage, it is necessary to increase activation of muscles surrounding the urethra, especially the pelvic floor muscle (PFM).

It is a well-known fact that PFM Training (PFMT) improves postoperative incontinence. Although PFMT depends on various factors related with the increase of PFM strength, the most important point is motor learning of correct contraction of PFM. To facilitate motor learning of correct contraction, clinicians recently started providing PFMT before radical prostatectomy when patients have normal sensation around the urethra. Additionally, biofeedback technique is also important. Transperineal ultrasound has an advantage in term of visualizing the urethral closure. Preoperative transperineal ultrasound-guided PFMT facilitates motor learning of PFM contractions and ameliorate urethral sphincter dysfunction after radical prostatectomy, thereby leading to early recovery of continence. (COI:No)

### Symposium24

### Shedding new lights on cell polarity

(March 30, Tue. 14：20～16：20, Room2)

### SY24-1

**The roles of cortical actin rings in the establishment of epithelial cell polarity**

Shusaku Kurisu[1], Shigenobu Yonemura[1,2] ([1]*Dept Cell Biol, Grad Sch Med Sci, Univ Tokushima*, [2]*Lab Ultrastruct Res, RIKEN BDR*)

In polarized epithelial cells, the boundary between apical and basolatreral membranes is traditionally thought to be defined by the cell-cell adhesion complex at the apex of the lateral membrane. However, recent evidence suggests that the cell-cell adhesion may be dispensable for separating apical and basal compartments, raising the question of what is the actual border between the two. Here, we exploit an alpha-catenin-deficient epithelial cells, namely R2/7 cells, which are defective in forming cell-cell junctions but still exhibit apicobasal polarity, to reveal critical components of the boundary. We systematically mapped the localization of cortical proteins and lipids in these cells and found that some actin regulators and phosphoinositdes are accumulated at the boundary. Interestingly, the boundary is ring-shaped and delineated by actomyosin cables, and most of the proteins and lipids identified were required for the boundary and resultant apicobasal polarity establishment. In the symposium, we will discuss how an interplay between actin dynamics and phosphoinositide metabolism leads to actin ring formation and epithelial polarization. (COI:No)

### SY24-2

**Functional mechanism of polarized phosphoinositides distribution generated by voltage-sensing phosphatase in sperm flagellum**

Takafumi Kawai[1], Yasushi Okamura[1] ([1]*Grad Sch of Med, Osaka univ*)

Voltage-sensing phosphatase (VSP) shows phosphoinositides phosphatase activity that is coupled to membrane potential. Previously, we reported that VSP-deficient sperm show severe defect in their motility after capacitation, resulting in significant reduction in success rate of fertilization in *in vitro* fertilization experiment. Electrophysiological analysis indicated that $K^+$ current that would be derived from Slo3, sperm specific $K^+$ channel is enhanced in $VSP^{-/-}$ sperm, and the polarized PtdIns(4,5)P2 distribution was important for regulating the Slo3 activity. Our results indicate that VSP appears to optimize PtdIns(4,5)$P_2$ distribution of the principal piece, contributing the normal sperm motility during capacitation. In spite of the important function of VSP in sperm physiology, we still do not know how and when such specialized PtdIns(4,5)$P_2$ distribution is formed by VSP activity. In the present study, we report the maturation-dependent VSP activity by examining sperm at different maturation stages. We also discuss the mechanism how membrane potential is important for regulating VSP activity during sperm maturation. (COI:No)

### SY24-3

**Functional analysis of a SNARE protein SNAP23 in the development of the cerebral cortex and cerebellum.**

Masataka Kunii[1], Akihiro Harada[1] ([1]*Dept Cell Biol, Grad Sch Med, Osaka Univ.*)

In the developing brain, the polarity of neural progenitor cells (NPCs) is necessary for neurogenesis. However, the mechanism for the NPC polarization remains unclear. Here, we show that SNAP23, which is a soluble *N*-ethylmaleimide-sensitive factor attachment protein receptor (SNARE) protein, has a crucial role in NPC polarization.

SNAP23 is involved in membrane fusion between transport vesicles and the plasma membranes; however, the *in vivo* function of SNAP23 is largely unknown. To elucidate the function of SNAP23, we generated central nervous system (CNS)-specific SNAP23 knockout (NcKO) mice.

NcKO mice showed severe hypoplasia of the neocortex and no hippocampus or cerebellum. In the developing NcKO brain, NPCs lose their polarity following the disruption of apical junctional complexes (AJCs) and exhibit reduced proliferation, increased differentiation, and increased apoptosis. We found that SNAP23 and its partner SNAREs, VAMP8 and Syntaxin1B, are important for the localization of an AJC protein, N-cadherin, to the apical plasma membrane of NPCs. Altogether, SNARE-mediated localization of N-cadherin is essential for AJC formation and NPC polarization during brain development. (COI:No)

### SY24-4

**Class II ARF can be a novel Nav1.6 localizing factor at the axon initial segment of cerebellar Purkinje cells**

Nobutake Hosoi[1], Koji Shibasaki[2], Teiichi Furuichi[3], Hirokazu Hirai[1], Tetsushi Sadakata[4] ([1]*Dept Neurophysiol and Neural Repair Gunma Univ Grad Sch Med*, [2]*Neurochem Lab Dept Nut Sci Univ Nagasaki*, [3]*Dept App Biol Sci, Tokyo Univ Sci*, [4]*Edu Res Support Center Gunma Univ Grad Sch Med*)

ADP-ribosylation factors (ARFs) are a family of small monomeric GTPases and six ARFs are categorized into class I (ARF1, 2, and 3), class II (ARF4 and 5), and class III (ARF6). Although class I and III ARFs are involved in vesicular membrane trafficking, the role of class II ARFs remains unclear. In this study, we generated class II ARF-deficient mice and found that $ARF4^{+/-}/ARF5^{-/-}$ double KO mice (class II ARF KO mice) exhibited essential tremor (ET)-like behaviors. Slice patch-clamp experiments demonstrated the reduced excitability of the cerebellar Purkinje cells (PCs) in class II ARF-KO mice. The class II ARF-KO PCs exhibited a reduction in the resurgent Na current which is related to Nav1.6 pore-forming Na channel alpha subunit and contributes to repetitive firing in PCs. Immunohistochemistry of class II ARF-KO mice revealed a severe and selective decrease of Nav1.6 proteins, but not ankyrin-G, in the axon initial segment (AIS) which is the action potential initiation site. These results suggest that class II ARFs are involved in the targeting of Nav1.6 proteins to the AIS of PCs, and that class II ARF deficiency may lead to ET-like movement disorder. (COI:No)

### Symposium25

### Metabolic regulation in the liver and its pathophysiological consequences

(March 30, Tue. 14：20～16：20, Room3)

### SY25-1

### Role of glucagon-inducible lncRNA in the regulation of hepatic metabolism

Takao Naganuma[1], Toshiya Matsukawa[1], Masaru Mitsushima[1], Masato Kasuga[2], Michihiro Matsumoto[1] ([1]*Department of Molecular Metabolic Regulation, Diabetes Research Center, Research Institute, National Center for Global Health and Medicine,* [2]*Institute for Adult Diseases, Asahi Life Foundation*)

Blood glucose homeostasis is primarily maintained by the coordinated action of two pancreatic hormones: glucagon and insulin. In diabetes, excessive glucagon action enhances gluconeogenesis in the liver, leading to hyperglycemia. Enhanced expression of gluconeogenic genes is mediated by glucagon, and its suppression ameliorates hyperglycemia in diabetes. Therefore, elucidating the molecular mechanism of gluconeogenic gene expression is an important challenge to control this process and thereby treat diabetes. We have previously shown that a nuclear signaling module, including acetyltransferase GCN5, transcriptional co-regulator CITED2, and PKA, is essential for the transcriptional induction of hepatic gluconeogenic genes by glucagon and that inhibition of this module in the diabetic liver improves hyperglycemia. To fully unveil the metabolic function of this module in the liver, we have comprehensively searched for molecules whose expression is induced by glucagon via this module and identified Lnc-GI, the glucagon-inducible lncRNA. In this presentation, we report the role of Lnc-GI in the regulation of hepatic metabolism. (COI:No)

### SY25-2

### Role of type2 innate lymphoid cells (ILC2) in liver on glucose metabolism regulation

Masanori Fujimoto[1] ([1]*Department of Molecular Diagnosis, Chiba University Graduate School of Medicine*)

Type 2 innate lymphoid cells (ILC2s) were recently identified as essential components of innate immunity. Besides, beneficial roles of ILC2s in metabolic diseases are attracting attentions. Therefore, we focused on unknown role of liver ILC2 in hepatic gluconeogenesis. Firstly, ILC2 activation by IL-33 injection decreased fasting blood glucose (FBG) levels in wt mice and T cell-deficient nude mouse. But IL-33 did not decrease FBG levels in NOD/SCID *Il2rg* null (NSG) mice lacking ILC2s. Pyruvate tolerance test showed IL-33 treatment also suppressed hepatic gluconeogenesis in wt mice, but not in NSG. IL-33 treatment drastically increased IL-13+ ILC2, and IL-33 did not suppress blood glucose in *Il13-/-* mice. Additionally, the transfer of *Il13-/-* ILC2 failed rescue the hypoglycemic effect of IL-33, suggesting that the effect of IL-33 depends on ILC2-derived IL-13. In consistent with these results, IL-13 suppressed expressions of gluconeogenic enzyme in primary hepatocyte in mouse and also in human. Furthermore, we investigated precise mechanisms of ILC2 activation and IL-13 indcution using Omics analysis (RNA-seq/ATAC-seq/GATA3-ChIP-seq/GATA3-IP-MASS). (COI:No)

### SY25-3

### Regulation of fatty acid biosynthesis in liver through GPR52 signaling

Mitsuo Wada[1,2], Kayo Yukawa[2], Hiroyuki Ogasawara[2], Koichi Suzawa[3], Tatsuya Maekawa[3], Yoshihisa Yamamoto[2], Takeshi Ota[4], Eunyoung Lee[1], Takashi Miki[1] ([1]*Department of Medical Physiology, Chiba University, Graduate School of Medicine,* [2]*Pharmaceutical Frontier Research Laboratories, Central Pharmaceutical Research Institute, Japan Tobacco Inc.,* [3]*Central Pharmaceutical Research Institute, Japan Tobacco Inc.,* [4]*Laboratory of Animal Physiology and Functional Anatomy, Graduate School of Agriculture, Kyoto University*)

GPR52 is a constitutively active, orphan G protein-coupled receptor, while its physiological function remains mostly unknown. We found that Gpr52 deficient (*Gpr52*$^{-/-}$) mice exhibit leanness with reduced liver weight and enhanced insulin sensitivity. *de novo* synthesis of fatty acid and cholesterol in the organ culture of liver was decreased in *Gpr52*$^{-/-}$ mice. We knocked-down (KD) GPR52 in a human hepatic cell line HepG2 cell and stimulated the cells with a GPR52 agonist c11. c11 treatment increased fatty acid synthesis in control cells (cont-HepG2) but not in KD-HepG2. In contrast, c11 treatment did not affect cholesterol synthesis in cont-HepG2, but KD of GPR52 significantly reduced its basal rate. In accord with this, mRNA expressions of lipogenic enzymes (*SCD1*, *ELOVL6*, and *ACC1*) were increased by c11 in cont-HepG2, but not in KD-HepG2. While mRNA expressions of *Scd1* and *Elovl6* were increased by high fat diet (HFD) feeding in wild-type mice, such induction was nullified in *Gpr52*$^{-/-}$ mice. Our study showed that GPR52 plays an essential role in promoting fatty acid biosynthesis under HFD feeding via transactivating lipogenic enzymes to facilitate fatty acid accumulation in liver. (COI:No)

### SY25-4

### The steatosis severity grade and cell death in liver regeneration

Yuka Inaba[1], Emi Hashiuchi[2], Hiroshi Inoue[1,2] ([1]*Insutitute for Frontier Science Initiative, Kanazawa University,* [2]*Graduate school of Medical Sciences, Kanazawa University*)

The liver has robust regenerative potential in response to damage, but hepatic steatosis weakens this potential. Impaired liver regeneration after cellular damage in the fatty liver is associated with an increased risk of postoperative complications and a progression of non-alcoholic fatty liver disease. We investigated this mechanisms using partial hepatectomy models of mice with the different severity grade of steatosis. Even mild steatosis showed impaired liver regeneration due to reduced cell proliferation and increased cell death by increased cellular stress with alpha subunit of eukaryotic initiation factor 2 (eIF2$\alpha$) phosphorylation. Growth arrest and DNA damage-inducible 34 (Gadd34) reduces the cellular stress by dephosphorylating eIF2$\alpha$. Therefore, we investigated the role of the cellular stress in fatty liver regeneration by knockdown or overexpression of Gadd34. We found that enhanced cellular stress impaired fatty liver regeneration, and that suppressed cellular stress ameliorates fatty liver regeneration failure by preventing cell death. In this symposium, I would like to discuss the molecular mechanism of the impaired liver regeneration induced by the cellular stress. (COI:No)

### SY25-5

### Role of cholesterol metabolism in macrophages in the pathogenesis of non-alcoholic steatohepatitis

Michiko Itoh[1,2], Takayoshi Suganami[2] ([1]*Kanagawa Institute of Industrial Science and Technology,* [2]*Department of Molecular Medicine and Metabolism, Research Institute of Environmental Medicine, Nagoya University*)

Increasing attention has been paid to nonalcoholic steatohepatitis (NASH) as a leading cause of hepatocellular carcinoma. In contrast to benign simple steatosis with triglyceride accumulation, NASH is characterized by accumulation of free cholesterol and cholesterol crystals, which induces hepatocyte cell death. Although Kupffer cells play important roles in engulfment of cell debris and remnant lipids, it still remains unclear how clearance processes of dead hepatocytes affect the pathogenesis of NASH. We previously reported that Melanocortin 4 receptor-deficient mice on Western diet exhibit NASH, and identified a unique histological structure termed "crown-like structure (CLS)", where dead or dying hepatocytes are surrounded by macrophages and fibroblasts, thereby accelerating liver fibrosis. We found cholesterol crystals in CLS, and increased cholesterol content in macrophages. Intriguingly, intervention to reduce free cholesterol in macrophages effectively ameliorated liver fibrosis. These findings provide the evidence that impaired cholesterol metabolism in macrophages contribute to the development of NASH, which would be a target of novel therapeutic strategies. (COI:No)

### SY25-6

### Treatment strategy for liver fibrosis using a novel deactivation factor of fibrogenic hepatic stellate cells

Yasuhiro Nakano[1,2], Yutaka Inagaki[1] ([1]*Center for Matrix Biology and Medicine, Graduate School of Medicine, Tokai University,* [2]*Department of Developmental and Regenerative Biology, Medical Research Institute, Tokyo Medical and Dental University [TMDU]*)

The activation of hepatic stellate cells (HSCs) is a key event accelerating liver fibrosis. Recently, HSC deactivation has been reported as a mechanism underlying the reversibility of experimental liver fibrosis. Here, we have identified Tcf21 as a novel deactivation factor of fibrogenic HSCs by screening transcription factors whose expression is up-regulated in parallel to the differentiation of embryonic HSCs. Tcf21 expression in HSCs was remarkably decreased in murine and human fibrotic liver tissue. Tcf21 was also examined for its effects by adeno-associated virus 6 (AAV6)-mediated *Tcf21* gene transfer into cultured activated HSCs and mice with carbon tetrachloride- or methionine-choline deficient diet-induced liver fibrosis. Overexpression of Tcf21 in activated HSCs not only suppressed fibrogenic gene expression, but also restored cells, at least in part, to a quiescent phenotype, both *in vitro* and *in vivo*. These phenotypic changes of HSCs were accompanied by the regression of steatohepatitis and fibrosis, and improved hepatic architecture and function. Collectively, these results provide insight into a treatment strategy for the otherwise intractable liver fibrosis. (COI:No)

### Symposium26

### New horizons in crosstalk between nociceptive and self-defense system

(March 30, Tue. 14：20～16：20, Room4)

### SY26-1

**Molecular mechanisms of action potential conduction and generation on mammalian myelinated nerves.**

Hirosato Kanda[1,2], Tsuyoshi Dai[1,2], Koichi Noguchi[2], Gu Jianguo[3] (*[1]Dept. Pharmacol., Hyogo Univ. Health Sci., [2]Dept. Anat. and Neurosci., Hyogo col. of Med., [3]Dept. Anesthesiology and Perioperative Med., Univ. of Alabama at Birmingham*)

Rapid conduction of sensory nerve impulses are critical in life and rely on the leap of action potentials (APs) via the nodes of Ranvier (NRs) along myelinated afferent nerves. While NRs are the only places where APs are regenerated to permit salutatory conduction, ion channel mechanisms underlying the regeneration and conduction of APs at mammalian NRs remain incompletely understood. Here we have developed a pressure-clamped patch-clamp recording method applying to the NRs of rat myelinated nerves. Using this key technique, we show that TREK-1 and TRAAK, the two-pore domain potassium channels, are clustered at NRs of A$\beta$-afferent nerves. We demonstrate that these TREK-1/TRAAK channels, but not voltage-gated $K^+$ channels are required for rapid AP repolarization at NRs. Furthermore, we show that these channels permit high-speed and high-frequency AP conduction, and loss of function of these channels retards nerve conduction and impairs sensory behavioral responses in rats. Collectively, our findings provide a novel mechanism responsible for rapid regeneration and conduction of APs in mammalian myelinated afferent nerves. (COI:No)

### SY26-2

**Neutrophil-neuron crosstalk induces mechanical allodynia in experimental autoimmune encephalomyelitis**

Yoshinori Hayashi[1] (*[1]Department of Physiology, Nihon University School of Dentistry*)

In multiple sclerosis (MS) patients, pain is a frequent and disabling symptom. However, the underlying mechanisms of pain in MS patients is not fully understood. In the present study, we found that neutrophil accumulation was observed in the dorsal root ganglion (DRG) in experimental autoimmune encephalomyelitis (EAE) mice caused by $MOG_{35\text{-}55}$ immunization, an animal model of MS. Accumulated neutrophils in the DRG released neutrophil elastase in a cathepsin E (CatE)-dependent manner. Adoptive transfer of $MOG_{35\text{-}55}$-stimulated wild-type but not *CatE*$^{-/-}$ neutrophils elicited mechanical allodynia in na?ve C57BL/6 mice. Neutrophil elastase activated protease-activated receptor 2 in DRG neurons. Activation of neutrophils by $MOG_{35\text{-}55}$ was mediated through toll-like receptor 4 (TLR4). $MOG_{35\text{-}55}$ also increased expression of chemokine (C-X-C motif) ligand 1 (CXCL1) in DRG neurons. Knockdown of CXCL1 or TLR4 in DRG neurons abrogated neutrophil accumulation in the DRG and mechanical allodynia after $MOG_{35\text{-}55}$ immunization. Thus, neutrophil-neuron crosstalk in the DRG is required for mechanical allodynia in EAE. These findings may provide a new strategy for preventing pain in MS patients.S patients. (COI:No)

### SY26-3

**The role of primary somatosensory cortex in nociception**

Tatsuya Ishikawa[1], Kei Eto[2], Hitoshi Ishibashi[2], Noriyuki Ozaki[1], Junichi Nabekura[3,4] (*[1]Department of Functional Anatomy, Graduate School of Medical Sciences, Kanazawa University, [2]Department of Physiology, Kitasato University School of Allied Health Sciences, [3]Division of Homeostatic Development, National Institute for Physiological Science, [4]Department of Physiological Sciences, Sokendai*)

It is well known that primary somatosensory cortex (S1) respond to noxious stimulation and has an important role in cognition of pain. Several groups including us revealed that neural circuit remodeling and synaptic plasticity in contralateral S1 (cont-S1) contributed to development of chronic pain state. In addition to activation of cont-S1, ipsilateral S1 (ipsi-S1) was also activated in chronic pain patients demonstrated by fMRI studies. Mechanisms and role of activation in ipsi-S1 was still unclear in chronic pain patients. To understand the role of ipsi-S1, we investigated the cellular activities of ipsi-S1 using *in vivo* 2-photon imaging in mice model of chronic pain.
Our data suggested that activation of inhibitory neurons and astrocytes in layer I in ipsi-S1 increased in chronic pain state. Enhanced inhibitory neuronal activity might avoid to cause abnormal pain sensation appears in the limb contralateral to the injury, called mirror image pain.
Thus, the role of activation of ipsi-S1 in chronic pain patients might be a defense system that avoids spreading of the pain symptom to contralateral uninjured regions. (COI:No)

### SY26-4

**Molecular mechanism of chronic post-surgical pain**

Yutaro Kumagai[1] (*[1]Cellular and Molecular Biotechnology Research Institute, National Institute of Advanced Industrial Science and Technology*)

Chronic post-surgical pain (CPSP) is a cause of pain suffering even after recovery from surgical insult with incidence rate of 5-50 %. Prior studies suggest that excessive activation of peripheral neurons as well as remodeling of neural circuit by microglia and/or astrocytes are cause of CPSP. However, its molecular mechanism is still elusive. To elucidate the mechanism of CPSP, we devised our original CPSP animal model and investigated the mice with deficiency in various genes involved in inflammatory response and migration of immune cells. We have found that mice deficient in genes involved in inflammatory response were resistant to CPSP. These results indicate that strong inflammation and migration of monocytes into the brain caused by surgical insult are essential molecular basis for CPSP. Moreover, to clarify molecular mechanism under inflammatory remodeling of the brain, we performed transcriptome analysis of whole brain of CPSP mice, and have found that expression levels of genes specific for myelinated oligodendrocytes was reduced. This indicated that CPSP accompanies brain functional network remodeling with demyelination-like reduction of myelinated oligodendrocytes. (COI:No)

### SY26-5

**Functional analyses of ion channels affected by environmental factors**

Yasunori Takayama[1] (*[1]Dep Physiol, Showa Univ Sch Med*)

Many natural factors directly activate or inhibit ion channels expressed in primary sensory neurons and epithelial cells. Importantly, one molecule can have the opposite effects on some ion channels. For instance, menthol activates TRPM8, a cold sensor in primary sensory neuron, while inhibits TMEM16A, a calcium-activated chloride channel involving in burning pain sensation. TMEM16A is inhibited by other plant-derived compounds other than menthol. Here, we show that a flavonoid generated in enterobacteria flora, liquiritigenin, inhibits TMEM16A. This finding suggests that relationship between food and bacteria can be involved in some physiological and pathological mechanisms dependent on TMEM16A, for instance gastrointestinal peristalsis and liver disease. On the other hands, we identified that RNA extracted from feces of mouse activated Piezo1, a mechanosensitive cation channel. Additionally, the duration of Piezo1 current induced by mechanical stimulation was expanded by RNA treatment. Thus, we indicate the novel relationship between nucleic acid and Piezo1 in intestinal epithelium. (COI:No)

### SY26-6

**Mechanisms of microbial bioerosion of bone**

Kenta Maruyama[1] (*[1]National Institute for Physiological Science*)

Understanding of the molecular mechanisms of the nociception has grown impressively in recent years. In nociception, microbial components evoke unpleasant feelings to alert host to the dangers of tissue damage. Such "infective" noxious stimuli trigger a variety of escape reactions and usually result in an altered homeostasis of osteo-immunity. Notably, nociceptors have the capacity to recognize pathogens or cytokines via sensory molecules or innate immune receptors and participate in osteo-immune responses. Accumulating evidence suggested that activated nociceptor produces various humoral factors and acts like an endocrine organ. Thus, understanding the interplay between the nociceptive and osteo-immune systems may open new avenues for the development of interdisciplinary research filed, which can be called "Sensoimmunology". In this talk, we will discuss the physiological importance of sensoimmune system in the context of bone biology, and how researching this system could produce novel treatments to cure osteo-immune diseases. (COI:Properly Declared)

### Symposium27

### Cardiovascular physiological response and pathologic analysis

(March 30, Tue. 14：20～16：20, Room5)

### SY27-1

**Physiological response to external stimuli (hydrostatic pressure and hyperthermia) in cardiac fibroblast**

Masanari Umemura[1], Masatoshi Narikawa[2], Ryo Tanaka[3], Rina Nakakaji[1], Akane Nagasako[1], Hiroko Nemoto[1], Yoshihiro Ishikawa[1] ([1]*Cardiovascular Research Institute, Yokohama University Graduate School of Medicine,* [2]*Department of Medical Science and Cardiorenal Medicine, Yokohama City University Graduate School of Medicine,* [3]*Yokosuka City Hospital*)

Mechanical stimulus and humoral factors may contribute to not only enlargement of cardiac myocytes, but also to activation and proliferation of cardiac fibroblasts, and differentiation of cardiac fibroblasts into myofibroblasts. However, the cellular signaling pathway and function of physical stimulus such as compressive force, i.e. hydrostatic pressure (HP) or hyperthermia in cardiac fibroblast remain elusive. We evaluated the effects of HP using a pressure-loading apparatus in human cardiac fibroblasts (HCFs) culture cells. Our finding showed that HP under a certain condition suppressed cardiac fibrosis via Akt/GSK-3 signaling compared to atmospheric pressure in HCFs. In contrast, hyperthermia (42 ℃) inhibited the TGF-β-induced collagen I, IL-6 production and α-SMA protein expression. Hyperthermia treatment also prevented cardiac fibrosis in Ang II infusion mice model. Our results demonstrated that hyperthermia directly inhibited the TGF-β induced differentiation from fibroblast to the myofibroblast phenotype in HCFs and cardiac fibrosis in mice model. We concluded that regulation of physical stimulus may be applicable to the prevention of cardiac fibrosis. (COI:No)

### SY27-2

**Effects of occlusal disharmony-induced stress on heart function in mice**

Kenji Suita[1], Yoshiki Onuki[1], Satoshi Okumura[1] ([1]*Dept Physiol, Sch Dent Med, Tsurumi Univ, Yokohama, Japan*)

Occlusal disharmony (OD) is perceived as chronic stress that can cause imbalances of the autonomic nervous system. Chronic sympathetic activation is associated with an increased incidence of cardiovascular diseases such as arrhythmias and heart failure. In this study, we hypothesized that OD may be a risk factor for cardiovascular diseases. We induced OD in mice by introducing bite-opening via placing an appliance on the lower incisors and examined the effects of OD on heart function and susceptibility to atrial fibrillation (AF). The low frequency/high frequency ratio, an index of sympathetic activity, was consistently increased after OD induction. The left ventricular ejection fraction and fractional shortening were lower, and the duration of AF induced by transesophageal burst pacing was longer in OD mice than control. Consistent with animal studies, OD aggravated myocyte apoptosis, fibrosis and oxidative DNA damage in cardiac tissue, but co-treatment with propranolol, a β-blocker, mitigated these OD-dependent changes. Our data suggests that disharmonious occlusal condition induces cardiac remodeling by chronic sympathetic activation, leading to deterioration of heart function. (COI:No)

### SY27-3

**Transcription Factor EB Regulates Hyperphosphatemia-induced Vascular Calcification.**

Ryo Ishiwata[1], Yuji Morimoto[1] ([1]*Dept Physiol National Def Med Col*)

**Introduction:** Vascular calcification (VC) is associated with cardiovascular-related mortality. Hyperphosphatemia deters the autophagy-lysosomal pathway in vascular smooth muscle cells (VSMCs), leading to VC. However, the reason why this pathway fails is elusive. Transcription factor EB (TFEB) is a master regulator of lysosome biogenesis. **Hypothesis:** Dysfunction of TFEB causes VC. **Results:** In an *ex vivo* culture of mouse aorta, the addition of inorganic phosphate (Pi) at a 1.7 mmol/L decreased TFEB protein expression (0.23 ± 0.10-fold, $n$ = 5). A decrease in TFEB was correlated with the VC formation. In rat VSMCs, the addition of Pi dose-dependently decreased TFEB protein expression both in whole cell lysate and in the nuclear fraction (0.07± 0.03-fold, $n$ = 5; 0.01 ± 0.003-fold, $n$ = 4) while it instead increased mRNA expression of *Tfeb* (4.48 ± 0.95-fold, $n$ = 7). The addition of Pi caused the accumulation of TFEB in the SDS-insoluble fraction, suggesting the formation of protein aggregates. The Knockdown of TFEB in VSMCs by siRNA exacerbated Pi-induced calcium deposition (2.93 ± 0.85-fold, $n$ = 4). **Conclusion:** Hyperphosphatemia causes diminution of TFEB, predisposing to VC. (COI:No)

### SY27-4

**Role of cardiac connexins in mice and humans**

Kiyomasa Nishii[1], Akiko Seki[2], Taisuke Ishikawa[3], Naomasa Makita[3], Yosaburo Shibata[4], Yasushi Kobayashi[1] ([1]*Dept. Anat. Neurobiol., Natl. Def. Med. Coll., Tokorozawa, Japan,* [2]*Dept. Prev. Med., Tokyo Women's Med. Univ., Tokyo, Japan,* [3]*Omics Res. Cent., Natl. Cereb. Cardiovasc. Cent., Suita, Japan,* [4]*Fukuoka Pref. Univ., Tagawa, Japan*)

Gap juncitons (GJs) in the heart consist of three major connexin (Cx) isoforms, and provide direct electrotonic connectivity between the cytoplasm of neighboring cardiomyocytes. Cx45 is the one with sparse permeability and predominates within the tubular heart. Mice lacking Cx45 completely or partially in the heart died in utero. We developed a computer-aided analysis of video microscopy and found that in a mixture of Cx45-positive and negative cardiomyocytes, unusual impulse propagation frequently caused retrograde conduction. In a recent study, we found a novel bradyarrhythmia syndrome associated with bone malformation in humans. The responsible gene encoded a Cx45 mutation, R75H, which impeded GJ communication in a dominant-negative manner. Lucifer yellow dye transfer and gap junction conductance were severely impaired between cell pairs expressing mutant Cx45. Patient-derived induced pluripotent stem cells will offer new insights on how Cx45 functions in human cardiogenesis and treatment of the patients. (COI:No)

### Symposium28

### Cortical neural microcircuit and its remodeling during learning

(March 30, Tue. 16：30～18：30, Room1)

### SY28-1

**Remodeling of Input/Output of the Primary Motor Cortex during Motor Learning.**

Yasuhiro Tanaka[1], Yoshito Masamizu[2,4], Yasuyo Tanaka[2], Takanori Shinotsuka[2], Masanori Matsuzaki[2,3,4] (*[1]Brain Sci. Inst., Tamagawa Univ., Tokyo, Japan, [2]Dept. Physiol., Grad. Sch. Medicine, Univ. Tokyo, Tokyo, Japan, [3]IRCN, UTIAS, Univ. Tokyo, Tokyo, Japan, [4]CBS, RIKEN, Saitama, Japan*)

Through motor learning, animals achieve the skilled movements required to accomplish their goals in everyday life effectively. The learning experiences should remodel how the primary motor cortex (M1) relates to subcortical structures. We have recently conducted two-photon imaging of M1 neurons and thalamocortical axons of mice acquiring motor skills. We found with neuronal calcium imaging that ensemble motor representations by layer 5a neurons, including neurons projecting the striatum, evolved. Axonal calcium imaging showed that thalamocortical inputs also altered during motor learning. Layer 1 (L1) axons, transmitting mainly basal ganglia signals, came to exhibit activity both at lever-pull initiation and termination. By contrast, layer 3 (L3) axons, conveying primarily cerebellar outputs, did so at lever-pull initiation. We also found that the neuronal sequence length in late sessions was longer in L1 than in L3. These findings suggest that during motor learning, M1 was remodeled to communicate effectively with subcortical structures.
(COI:No)

### SY28-2

**Low-contrast preference of activity in rat primary visual cortex after learning an orientation discrimination task.**

Rie Kimura[1,2], Yumiko Yoshimura[1,2] (*[1]Division of Visual Information Processing, National Institute for Physiological Sciences, National Institutes of Natural Sciences, [2]Department of Physiological Sciences, School of Life Science, SOKENDAI [The Graduate University for Advanced Studies]*)

Animals can often perceive familiar vague visual stimuli. To explore the neural bases, we performed multiple single-unit recordings in rat primary visual cortex (V1) and analyzed visual responses to high- or low-contrast stimuli after learning an orientation discrimination task. We found that the firing rates in a subset of neurons increased with a reduction of the contrast. These low contrast-preferring neurons were hardly observed during passive-viewing without training or anesthesia after training. The firing rates in the neurons were higher in correct-choice trials than incorrect trials. At single-neuron and population levels, the neurons accurately represented low-contrast orientations. After training, excitation was enhanced irrespective of stimulus contrast, and the phase synchronization of spikes to the *beta* oscillations, generated selectively at high contrast, was stronger in putative inhibitory than excitatory neurons. The change in excitation and inhibition balance might contribute to low-contrast preference. Taken together, low contrast-preferring activities, which are established in V1 after learning, are suggested to contribute to low-contrast visual discrimination. (COI:No)

### SY28-3

**Reconfiguration of corticocortical and thalamocortical synapses during motor learning**

Jaerin Sohn[1], Yasuo Kawaguchi[1,2], Yoshiyuki Kubota[1] (*[1]Division of Cerebral Circuitry, National Institute for Physiological Sciences [NIPS], [2]Brain Science Institute, Tamagawa University*)

Repetitive practice affords a novel motor skill to animals, while it rearranges the circuit in the motor cortex. This network rewiring is indicated by spinogenesis on pyramidal cell dendrites. Spine formation and maintenance are then considered to be important for motor-skill acquisition and retention. The description of postsynaptic spine plasticity, however, lacks information of presynaptic side in axo-spinous interaction, and the origin of input to those newly-formed spines remains unknown. In our current work, we determined the presynaptic axon terminals innervating new spines formed during motor learning. Post hoc characterization of the presynaptic axon terminals on motor learning-related new spines revealed that the new spines formed during the initial 4 days of training were frequently innervated by corticocortical axon fibers. By contrast, new thalamocortical synapses appeared less frequently, but survived longer and increased spine size more than new corticocortical synapses. The input type-dependent dynamics during motor learning suggests that acquisition and maintenance of motor skills involve different cortical and subcortical network remodeling. (COI:No)

### SY28-4

**Optical and computational dissection of prefrontal neural circuit for fear memory**

Masakazu Agetsuma[1,2,3], Issei Sato[4], Yasuhiro Tanaka[5], Atsushi Kasai[6], Yoshiyuki Arai[3], Miki Yoshimoto[1], Hitoshi Hashimoto[6], Junichi Nabekura[1], Takeharu Nagai[3] (*[1]National Institute for Physiological Sciences, [2]Japan Science and Technology Agency, PRESTO, Kawaguchi, Japan, [3]The Institute of Scientific and Industrial Research, Osaka University, Ibaraki, Japan, [4]Graduate School of Frontier Sciences, The University of Tokyo, To kyo, Japan, [5]Brain Science Institute, Tamagawa University, Machida, Japan, [6]Graduate School of Pharmaceutical Sciences, Osaka University, Sui ta, Japan*)

For efficient and accurate information processing in cerebral cortex, neural population dynamics must be spatially and temporally regulated with great precision. Medial prefrontal cortex (mPFC) of rodents has been shown important for various types of learning and memory, including fear memory. However, it has been challenging to understand the computational architecture in the mPFC, of which major problems are the complexity and heterogeneity of the prefrontal computation. We investigate this by chronic two-photon Ca2+ imaging from populations of neurons in mouse mPFC in vivo, which allows us to record activities simultaneously from large number of neurons at the single cell resolution, and investigate changes of neuronal responses over the learning process. We investigated the change in responses of mPFC neurons during Pavlovian fear conditioning (tone + aversive stimulus) and memory retrieval using a new device to perform them with a head fixed mouse. In the presentation, I will discuss how individual neurons and the neural population encode the fear memory. (COI:No)

### Symposium29

### Neurobiology of single axon: structure and function

(March 30, Tue. 16：30～18：30, Room2)

### SY29-1

**Membrane potential imaging of dynamic axonal information processing in the cerebellar neurons**

Shin-ya Kawaguchi[1] (*[1]Society-Academia Collaboration for Innovation, Kyoto University, Japan*)

Electrical signals are processed in one-way direction in central neurons: synaptic inputs are integrated in dendrites and soma, and the signal is then changed to action potentials (APs) which faithfully propagate to axon terminals, where the information is transmitted to target neurons. However, small size of an axon and terminals of central neurons has hindered complete demonstration of the one-way signaling. Here, I show an activity-dependent long-term plasticity of axonal information flow, revealed by high spatio-temporal analysis of neuronal activity using genetically-encoded fluorescent voltage imagings coupled with axonal patch-clamp recordings. Cerebellar Purkinje cells basically show firing initiation around the soma, but the site drastically shifts toward the distal axonal regions for days after the chemical induction of learning-related synaptic plasticity. The dynamic change of AP firing site was accompanied with augmentation of axonal $GABA_A$ receptors and higher local membrane excitability. Thus, dendritic postsynaptic plasticity induces sustained axonal plasticity, resulting in the dynamic changes of axonal firing sites.

(COI:No)

### SY29-2

**Roles of the ER-mitochondria contact site in the axon of neocortical excitatory neurons**

Yusuke Hirabayashi[1] (*[1]Dept. ChemBio., Sch. Eng., Univ. Tokyo*)

Endoplasmic Reticulum (ER)-mitochondria contact are important for maintaining cellular homeostasis, the regulation and physiological roles of these contacts are still largely unknown in metazoans. This is because the molecular mechanisms underlying ER-mitochondria tethering remain elusive, Recently, we identified PDZD8 as an ER-resident protein present at ER-mitochondria contacts (Hirabayashi et al. ***Science*** 2017). Functionally, we discovered that PDZD8 is a critical ER-mitochondria tethering protein in the dendrite, where it is essential for rapid calcium transfer from the ER to the mitochondria in response to synaptic inputs. In the axon, we have recently found that there is a unique form of ER-mitochondria contact sites at the presynaptic bouton. Here, I would like to discuss the roles of ER-mitochondria contact sites in the axonal growth and functions.

(COI:No)

### SY29-3

**Axonal collateralization of single neurons in the rat parahippocampal regions**

Yoshiko Honda[1] (*[1]Dept.Anat and Neurobiol. Sch.Med. Tokyo Women's Med Univ.*)

The axonal branching patterns of single neurons in the rat parahippocampal regions including the presubiculum (PreS) and entorhinal cortex (EC) were investigated by using in vivo injection of a viral vector expressing membrane-targeted palmitoylation site-attached green fluorescent protein. The vector is a highly sensitive anterograde tracer, and we can clarify the components of known, major projections at the single-cell level. We indicated that single layer III neurons of PreS formed elaborate terminal arborsin EC and that layer V neurons could be classified into at least six types according to the axonal branching patterns. Furthermore, this approach can lead to identification of novel projections, which have never been detected. We could find that single layer V neurons of EC had axon collaterals that innervated to the dentate gyrus with complex terminal arborization. To further understand the functional roles played by PreS and EC, it is essential to elucidate how output signals from each neuron of these areas distribute to the target regions. Knowledge about single cell-level projections provide new insight into the functional organization of these cortical areas.

(COI:No)

### SY29-4

**Elucidation of axon conduction properties with high-density microelectrode array**

Kenta Shimba[1], Kiyoshi Kotani[2], Yasuhiko Jinbo[1] (*[1]Dept Precision Engineering, School of Engineering, The University of Tokyo, [2]Research Center for Advanced Science and Technology, The University of Tokyo*)

The neural axons are considered to play a role in the computational functions, but not just cables. Although electrophysiological methods have been used to characterize conduction properties, spatial resolution has been limited to evaluate spatial heterogeneity along axon. The present study aimed to clarify the relationship between axonal structure and conduction properties using a high-density microelectrode array (HD-MEA), a measurement device with more than 20,000 electrodes on the culture substrate. Rat dorsal root ganglion neurons were cultured on the HD-MEAs. Neural activity was induced by blocking potassium ion channels, and the activity was recorded. Since axonal signal is smaller than soma, the activity was averaged by triggering the cell body's activity times. We detected the shape of the axons of individual neurons. The results show that there is a difference in conduction velocity depending on the shape of the axon. These results show that HD-MEA is suitable for evaluating the spatial variation of conduction properties of axons.

(COI:No)

### SY29-5

**Single axon morphology to classify neuronal populations and understand brain organization**

Izumi Sugihara[1], Luo Yuanjun[1] (*[1]Department of Systems Neurophysiology, Tokyo Medical and Dental University*)

An axon is a conducting unit of intrinsic neuronal activity to all the terminals of its branches, formed by intrinsic/genetic and activity-dependent mechanisms to compose neural circuitries in the CNS.

Therefore, single axon morphology (pathway, branching and termination) represents neuronal characteristics and nervous system organization directly.

Single axons of the cerebellar mossy fiber systems from somatosensory, vestibular and pontine nuclei show significant differences in branching patterns besides pathway differences. Some have no collaterals outside of the cerebellum (precerebellar type) while others show many collaterals outside of the cerebellum (non-precerebellar type), suggesting some phylogenetic change in axonal morphology. In the cerebellar cortex, mossy fibers originating from a single axon of these systems often terminate in a combination of multiple lobules, which is an indicative functional link among them. Furthermore, such target differences indicate subpopulations in each group of the mossy fiber axons and their original neurons.

In summary, single axon morphology is essential in understanding the organization of cerebellar mossy fiber systems.

(COI:No)

### SY29-6

**Subcellular analysis of propagation and oscillation of single axon**

Haruyuki Kamiya[1] (*[1]Department of Neurobiology, Hokkaido University Graduate School of Medicine, Japan*)

Reliable and high-speed computation in the brain is enabled due to the binary nature of spike generation as well as ultrafast propagation along the axon. To explore the dynamics of the axonal spikes and the impacts on the synaptic strength and plasticity, we developed direct recordings from the single axon terminals of the mossy fibers in mouse hippocampal slice preparation. Spike propagation along the mossy fibers was found to be highly reliable in the physiological frequency range and accompanies minimal conduction failure. Contrary to the all-or-none principle, however, the height of the axonal spike is subjected to a small depression in response to repetitive stimuli. Short-term depression of axonal spikes results in the modulation of the presynaptic $Ca^{2+}$ entry and therefore is revealed as a determinant of fine-tuning of short-term synaptic plasticity. Subcellular recording from the axons also provides a valuable opportunity for studying the ectopic spike generation from the distal axon, a different mode from the physiological spike initiation from the axon initial segment. The mechanisms underlying ectopic bursts as well as the functional significance will be discussed.

(COI:No)

### Symposium30

### Front in progress on cancer stem cell research - molecular basis and novel therapeutic strategies

(March 30, Tue. 16：30〜18：30, Room3)

### SY30-1
**Functional analysis of non-coding RNAs in glioma stem cells**

Souichi Oe[1], Rio Kakizaki[1], Sumika Sakamoto[1], Masaaki Kitada[1] ([1]*Dept. Anat., Kansai Med. Univ.,*)

Gliomas are the most frequently occurring primary brain tumors. In recent years, glioma stem cells (GSCs), which are capable of tumorigenesis, self-renewal, and multilineage differentiation, has attracted much attention as a cause of GBM. In addition, there are several subtypes of GSCs; proneural (PN) GSCs expressing genes involved in neurogenesis, and mesenchymal (MES) GSCs with high tumorigenic potential. Proneural-to-Mesenchymal Transition (PMT) has been proposed as a key process that contributes to malignancy. We aim to elucidate the molecular mechanisms of PMT using PN and MES GSCs established from human glioma specimens. Furthermore, we focus on non-coding RNAs, especially microRNAs and long non-coding RNAs (lncRNAs). We have identified several microRNAs and lncRNAs that are expressed in a subtype-specific manner by microarray analysis and real-time PCR. Subsequent functional analysis revealed that non-coding RNAs are involved in the regulation of PMT, proliferation, invasion, drug resistance, and apoptosis. In this presentation, we will discuss the mode of actions of non-coding RNAs in GSCs. (COI:No)

### SY30-2
**Plasticity of power-law coded heterogeneous glioma stem cell populations**

Michiya Sugimori[1], Yumiko Hayakawa[2], Ryohei Tamura[1], Satoshi Kuroda[2] ([1]*Univ Toyama, Faculty Med, Dept Integrative Neuroscience,* [2]*Univ Toyama, Faculty Med, Dept Neurosurgery*)

The cell populations responsible for the recurrence and progressive tumor expansion in glioblastoma are glioma stem cell (GSC) populations. For developing an efficient chemoradio-therapy, the issues of chemoradio-resistance and tumor size expansion should quantitatively be detailed. We employ a clonal tumor neurosphere culture system, where glioma stem cells are enriched as a population. The culture system allows glioma cell-derived clones to survive and clonally to expand to form glioma spheres (GS). The GS clones differentially grow to reconstitute the GS populations, where the GS clone sizes exhibit diversity and strictly follow a power-law. The power-law coded heterogeneous GS populations reconstitute the power-law coded heterogeneity over generations, while the population sizes gradually increase. Subsets of both small- and large-sized GS clones completely reconstitute power-law coded GS populations, suggesting the GS populations are plastically self-renewable heterogeneous GSC populations. The culture system benefits us to reveal chemo-drugs' efficacies in the survival, the growth, and the heterogeneity of GSC clones; how plastically GSC populations resist the chemo-drugs. (COI:Properly Declared)

### SY30-3
**Development of anticancer drug for brain tumor targeting mucolipin**

Mikio Hayashi[1] ([1]*Department of Cell Physiology, Institute of Biomedical Science, Kansai Medical University*)

Glioblastoma multiforme (GBM) is the most fatal malignant primary brain tumor. GBM contains functional subsets of cells called glioblastoma stem-like cells (GSCs), which are radio- and chemo-resistant and eventually lead to tumor recurrence. However, the molecular target for treatment of GSCs has not been extensively investigated. Thus, the present study aimed to develop anticancer drug targeting ion channels in GSCs. We established the stem-like cells from human GBM using three-dimensional cell culture. We found a potent blocker of non-selective cation currents in the GSCs using patch-clamp techniques. The treatment of the blocker inhibited cell growth of GSCs. Immunohistochemical analysis revealed that mucolipin, a non-selective cation channel, was expressed in the plasma membrane of GSCs. These results indicate that mucolipin contributes to cell growth of GSCs and has the potential to be therapeutic targets of GBM. (COI:No)

### SY30-4
**Intercellular communication at glioblastoma stem cell niches as a therapeutic target**

Takuichiro Hide[1], Yuki Shirakawa[2], Hirofumi Jouno[2], Ichiyo Shibahara[1], Akitake Mukasa[3], Hideyuki Saito[2], Toshihiro Kumabe[1] ([1]*Department of Neurosurgery, Kitasato University School of Medicine,* [2]*Department of Clinical Pharmaceutical Sciences, Graduate School of Pharmaceutical Sciences, Kumamoto University,* [3]*Department of Neurosurgery, Graduate school of Medical Sciences, Kumamoto University*)

Glioblastoma (GBM) is a lethal brain tumor and commonly develops in adult. GBM comprises a small population of GBM stem cells (GSCs), which potentially cause therapeutic resistance and recurrence. GSCs harbored in special microenvironments (GSC niches). However, the mechanisms underlying the pathogenesis and maintenance of GSCs remain largely unknown.
Stemness and chemo-radioresistance was promoted not only by additional mutation, but also GSC niches. We reported that growth factors and cytokines secreted by oligodendrocyte and macrophages/microglia at border niche induce stemness into GBM cells. Moreover, we found that ribosomal protein S6 (RPS6) promotes stemness into GBM cells, and other ribosomal proteins were also upregulated in GSC niches. Ribosomal proteins play crucial roles in the development and maintenance of GSCs, and are clinically associated with therapeutic resistance and recurrence.
Thus, intercellular communications through growth factors, cytokines, and ribosomes are regarded as new therapeutic targets of GBM. (COI:No)

### SY30-5
**Spatiotemporal Regulation of Long non-coding RNA to propagate Cancer Cells**

Yutaka Kondo[1] ([1]*Div Cancer Biol, Grad Med Sch, Nagoya Univ*)

As the term glioblastoma "multiforme" suggests, the histopathology of this tumor is extremely variable. Given dynamic regulation of gene function (i.e. epigenetic mechanism) is not only important for tumor formation but also plays a pivotal role in the plasticity of tumor cells, it might contribute to the establishment of intratumor heterogeneity. We found that polycomb repressive complex 2 (PRC2) and its catalytic component protein, EZH2, is required for the differentiation and dedifferentiation of glioma stem cells. Furthermore, we identified long non-coding RNAs (lncRNAs) to regulate gene expression through providing a scaffold for chromatin modifying proteins, such as PRC2, and recruiting these proteins to target loci. Recently, we found a lncRNA has a novel spatiotemporal role in cancer cells as a compulsory molecule to regulate oncogene-induced DNA replication stress (RS). In this session, I focus on the recent advancements in epigenetic research with respect to gliomas, consider how epigenetic mechanisms dynamically regulate tumor cells, including the cancer stem cell population, and discuss perspectives and challenges for glioma treatment in the near future. (COI:Properly Declared)

### Symposium31

### Emerging technologies for live-cell imaging and analysis

(March 30, Tue. 16：30～18：30, Room4)

### SY31-1

**Visualizing phagosomal membrane potential and investigating the roles in macrophages**

Yoshifumi Okochi[1], Hidekazu Tsutsui[2], Yasushi Okamura[1] (*[1]Integrative Physiology, Graduate School of Medicine, Osaka University, [2]Bioscience and biotechnology, JAIST*)

Phagosome is a specific vesicle, which is formed upon phagocytosis of pathogens and dead cells. While phagosomal pH is essential for killing and digesting pathogens, roles of the membrane potential in phagosomes is unclear. Because, several tools measuring phagosomal pH have been developed, whereas it lacks the method measuring membrane potential change. To overcome this problem, we applied fluorescent imaging technique to phagosome and succeeded visualization of phagosomal membrane potential in RAW264.7 macrophage cell line by using FRET-based voltage probe Merm2. We previously reported that phagosomal membrane potential hyperpolarizes with maturation of phagosome compared to plasma membrane. We next addressed the question what ion channels contribute to hyperpolarization in phagosomes. For the purpose, we isolated phagosomal membrane fraction from RAW cells and identified molecules on phagosomes by using mass spectrometry. Those fractions included chloride channels and a potassium channel, those of which possibly contribute to hyperpolarization. We are now trying to identify which channels are responsible for hyperpolarized membrane potential pharmacologically and genetically. (COI:No)

### SY31-2

**Toward molecule-specific formation of neuron-microelectrode junctions**

Hidekazu Tsutsui[1], Sam Young Kim[1], Sm Ahasanul Hamid[1], Mieko Imayasu[1], Tomoyuki Yoshida[2], Kosuke Sekine[1], Wataru Haga[1] (*[1]Material Sci., JAIST, [2]Grad. Sch. Medicine, Toyama Univ.*)

The microelectrode techniques have long enabled us to investigate the physiological properties of the excitable cells and their networks. Although these techniques generally allow high temporal resolution recordings, they intrinsically lack cell-type specificity. Target cells must be identified by means of other indirect criteria such as marker gene expression and cellular morphology, which is not well-compatible with parallel recordings from highly heterogeneous cellular networks. By combining the robust bioactivities of synapse inducing molecules with microfabrication techniques, we have set out to establish a means enabling the molecule-specific formation of neuron-microelectrode junctions. Following contact of neurons with a microelectrode immobilized with natural synapse organizing molecule, presynaptic terminals were successfully induced onto the electrode. Also, we have generated some sets of prototype engineered synapse organizing molecules that do not cross-react with each other. It is expected that such molecular tools are exploited to develop the micro- or nano-electrode technique that permit selective recording from genetically specified cells. (COI:No)

### SY31-3

**Visualization of retinoic acid gradients**

Satoshi Shimozono[1], Atsushi Miyawaki[1] (*[1]The Institute of Physical and Chemical Research*)

Retinoic acid (RA) is a biologically active metabolite of vitamin A. In vertebrates, RA functions as a morphogen, a substance whose concentration gradients control the morphogenesis of embryonic organs.

So far the spatiotemporal pattern of the RA signaling has been visualized by reporter gene assays in which a reporter such as LacZ is put downstream of an a retinoic acid response element sequence. The responses are, however, relatively slow compared to the dynamic processes of embryogenesis.

To detect RA more directly, we have developed FRET (Fluorescence Resonance Energy Transfer)-based probes with ligand binding domains of RA receptors being flanked by CFP and YFP. The probes were named GEPRAs (Genetically Encoded Probes for Retinoic Acid).

We first applied GEPRA probes to visualize RA in zebrafish. Live imaging by GEPRAs revealed linear concentration gradients of RA declining from the trunk to the head and tail, where the hindbrain and somites are formed, respectively. We found a clear correlation between the slope of the RA gradients and the morphology of the hindbrain. Now we are analyzing RA distributions within the mouse embryo expressing the GEPRA probe. (COI:No)

### SY31-4

**Development of POLArIS, a versatile and genetically encoded fluorescent probe for molecular orientation, and its application in live-cell imaging**

Sumio Terada[1] (*[1]Dept. Neuroanat./Cell. Neurobiol., Grad. Sch. Med. Dent. Sci., Tokyo Med. Dent. Univ.*)

Monitoring molecular orientation is the key approach to study the spatial organization of molecular alignments within supramolecular complexes in living cells, but the opportunity is missing even with the latest super-resolution approaches. Fluorescence polarization microscopy is a promising solution, but it is challenging to label target proteins with fluorescent probes in a sterically constrained manner, which is prerequisite for fluorescent polarization imaging. We have developed POLArIS, a versatile and genetically encoded probe for fluorescent polarization imaging. POLArIS is a recombinant binder with a rotationally constrained fluorescent protein and can target arbitrary biomolecules by combining with phage-display screening. Application of POLArIS that specifically binds to F-actin to live-imaging of starfish embryos revealed radially extended actin filaments in mitosis that had been escaping from previous observations. By taking advantage of the genetically encoded nature, POLArIS has a potential of supporting broader applications of fluorescence polarization techniques to a variety of living specimens. (COI:No)

### Symposium32

### Frontiers in heart research: morphogenesis, structure and function

(March 30, Tue. 16：30～18：30, Room5)

### SY32-1

**Novel Findings of Myocardial Differentiation via Single Cell Analysis**

Kenta Yashiro[1] ([1]*Anatomy & Developmental Biology, Kyoto Prefectural University of Medicine*)

Better understanding of cardiac development is vital to uncover the pathophysiology underlying congenital heart diseases as well as to develop regeneration therapy for the heart failure. However, our knowledge is still incomplete to translate in the clinical arena.
During embryogenesis, the heart originates from cardiac progenitor cells (CPCs). CPCs populate "the Heart Field" within the anterior part of the embryos at embryonic day 7.5 in mice, after presumptive heart mesoderm cells migrate anteriorly from the primitive streak. Thus far, the molecular mechanism for the commitment to CPCs and for the cell fate determination of cardiac cells still remains unclear.
To deepen our knowledge further, we have elucidated CPCs via single-cell expression profiling and lineage tracing. Based on our data, we will discuss the behavior of the earliest CPCs and their differentiation. (COI:Properly Declared)

### SY32-2

**Homeostatic regulation of calcium signal in cardiac myocytes and its failure in diastolic dysfunction**

Satomi Adachi-Akahane[1] ([1]*Dept Ohysio, Fac Med, Toho Univ, Tokyo, Japan*)

In ventricular myocytes, in response to the action potential, $Ca^{2+}$ influx through the L-type $Ca^{2+}$ channel (LTCC) triggers $Ca^{2+}$-induced $Ca^{2+}$ release from the type 2 ryanodine receptor, situated in proximity to the LTCC, to cause an increase in the intracellular $Ca^{2+}$ concentration ($[Ca^{2+}]_i$) and contraction. Subsequently, $[Ca^{2+}]_i$ rapidly drops to the resting level by extrusion of $Ca^{2+}$ via $Na^+$-$Ca^{2+}$ exchanger and $Ca^{2+}$ uptake into the sarcoplasmic reticulum (SR) by SERCA2. These critical steps for homeostatic regulation of $Ca^{2+}$ signal are based on the geometrical localization of $Ca^{2+}$ signaling machinery and regulators at the junctional membrane structure consisting of the T-tubular and the junctional SR membranes. On the other hand, it has recently been shown that the spatial localization of $Ca^{2+}$ signaling proteins and their modulatory mechanisms start to deteriorate early in the onset of ventricular diastolic dysfunction. In this symposium, I would like to introduce the latest research on the molecular mechanism in regulating the spatial localization and modulation of $Ca^{2+}$ signaling proteins and its role as the causal mechanism for diastolic dysfunction and the therapeutic target. (COI:No)

### SY32-3

**Search for RyR2 inhibitors and verification of their effects on arrhythmogenic myocardium**

Nagomi Kurebayashi[1] ([1]*Department of Pharmacology, Faculty of Medicine, Juntendo University, Tokyo, Japan*)

Ryanodine receptor 2 (RyR2) is the $Ca^{2+}$ release channel on the sarcoplasmic reticulum (SR) and plays an essential role in EC-coupling in the heart. Abnormal activation of RyR2 has been linked to arrhythmogenesis, where spontaneous $Ca^{2+}$ release via RyR2 triggers ectopic activity by enhanced Na-Ca exchange reaction. For example, chronic phosphorylation of RyR2 in heart failure and gain-of-function mutations in RyR2 linked to catecholaminergic polymorphic ventricular tachycardia (CPVT) have been reported to cause malignant ventricular arrhythmias. In these cases, drugs that suppress RyR2 activity are expected to have anti-arrhythmic effects, but specific inhibitors of RyR2 have not been reported yet. We have recently established a high-throughput screening method for detection of isoform specific RyR inhibitors (Murayama et al, Mol Pharmacol, 2018) and found several RyR2 inhibitors. Furthermore, we successfully synthesized structural analogs from one of hit compounds with higher affinity. These newly found RyR2 inhibitors on myocardium ameliorated abnormal $Ca^{2+}$ and membrane potential signals. Our results suggest that RyR2 inhibitors are promising novel antiarrhythmic drug candidates. (COI:No)

### SY32-4

**Activation of ryanodine receptor type 2 by TRIC-A**

Hiroshi Takeshima[1] ([1]*Kyoto Univ, Grad Sch Pharm Sci*)

In the endo/sarcoplasmic reticulum (ER/SR), ryanodine receptor (RyRs) and inositol trisphosphate receptors ($IP_3Rs$) function as $Ca^{2+}$ release channels, and TRIC-A and TRIC-B are major $K^+$ channels. When $Ca^{2+}$ is released, a negative potential is generated on the ER/SR lumen and may inhibit subsequent $Ca^{2+}$ release. Counter-ions balancing SR/ER membrane potential have been thus proposed. TRIC subtypes likely contribute to the counter-ion movements, because *Tric*-deficient cell types commonly exhibited impaired $Ca^{2+}$ release and store overloading. The cardiac SR abundantly contains RyR type 2 (RyR2) and TRIC-A, and *Tric-a*-knockout cardiomyocytes exhibited impaired RyR2-mediated $Ca^{2+}$ release and SR overloading. When expressed in HEK cells, RyR2 generated $Ca^{2+}$ oscillations, presumably triggered by SOICR (store-overload-induced $Ca^{2+}$ release) gating and following ambient CICR ($Ca^{2+}$-induced $Ca^{2+}$ release) gating. Coexpressed TRIC-A highly activated RyR2 to abolish $Ca^{2+}$ oscillations, likely by increasing the $Ca^{2+}$ sensitivity for SOICR and/or CICR gating. Thus, in addition to the counterion-conducting function, TRIC-A may directly modulate RyR2 gating. (COI:No)

### SY32-5

**Channel opening mechnism of Ryanodine receptor by $Ca^{2+}$**

Haruo Ogawa[1] ([1]*IQB, The University of Tokyo*)

Ryanodine receptor (RyR) is a large (~2.2 MDa) $Ca^{2+}$ release channel in the sarcoplasmic reticulum of skeletal and cardiac muscles and plays a key role in excitation-contraction coupling. A cardiac specific isoform, RyR2, is activated by $Ca^{2+}$ and its mutations have been implicated in severe arrhythmogenic heart diseases, such as catecholaminergic polymorphic ventricular tachycardia (CPVT). Yet, the structural basis underlying channel opening and how mutations affect the channel remain largely obscured. Here, we combined high-resolution structures determined by cryo-electron microscopy with quantitative functional analysis of channels carrying various mutations in specific residues. Our result revealed the underlying mechanisms in the channel opening upon $Ca^{2+}$ biding and alterations in channel activity by disease-associated mutations of RyR2 at the atomic level. (COI:No)

### Symposium33

### Functional relationships among the sensation, the emotion and the motion from anatomical and physiological view

(March 30, Tue. 16：30～18：30, Room6)

### SY33-1
**The roles of the Parabrachial neurons concerning the nociception-respiration coordination**

Akiko Arata[1] ([1]*Depy Physiol, Hyogo Coll Med*)

The sensation of nociceptive signals projects to the lateral parabrachial nucleus (LPB) of the pons; and LPB has also known as the system of inspiratory-expiratory (I-E) phase switching that contributes to the control of respiratory rate. In this study, we investigated the nociceptive-respiratory system using the pons-medulla-spinal cord preparation intact forelimb isolated from neonatal rats. Respiratory activity was increased significantly when capsaicin was injected into forelimb. We examined the neurons recorded from the nociceptive responded area in LPB using whole-cell patch-clamp. Some I-E neurons and the half of non-respiratory neurons were excited by capsaicin injection. Some Inspiratory neurons showed EPSPs in I-E phase so that be changed from inspiratory to I-E neuron that activated by non-respiratory inputs. These results suggested that the I-E neurons were very likely the core mechanism of nociceptive-respiratory coordination since I-E neurons could be changed their firing pattern by receiving noxious information; the non-respiratory LPB neurons might be participating in the onset of the nociceptive-respiratory relay network.
(COI:No)

### SY33-2
**The role of the central nucleus of the amygdala in inflammation-induced pain-network plasticity**

Yae Sugimura[1], Yukari Takahashi[1], Takao Okuda[1], Ryota Tokunaga[1], Fusao Kato[1]
([1]*Department of Neuroscience, The Jikei University School of Medicine*)

The central nucleus of the amygdala (CeA) receives aversive signals and send outputs to optimize physiological and behavioral responses for survival. One of the sources of these inputs is the lateral parabrachial nucleus (LPB), which receives nociceptive and inflammatory signals from the periphery. Unlike the well-described plasticity at the synapses from the LPB to the capsular part of the CeA (CeC) in various pain models, little is known how output neurons in the lateral and the medial part of the CeA (CeL and CeM, respectively) are affected. To shed light on these CeA output neurons, we made whole-cell patch-clamp recordings from cells projecting to the periaqueductal grey (PAG), a primary nucleus for descending pain modulation system. Electrophysiological and morphological properties, together with synaptic responses to optogenetically triggered inputs of LPB origin revealed a subset of CeA output neurons with distinct electroresponsive properties. Moreover, systemic inflammation altered synaptic responses of LPB origin in CeA output neurons. These findings would support the role of the CeA as a modifiable "hub" in the network processing pain/inflammation.
(COI:No)

### SY33-3
**Projection dependent circuits in layer 5 of the rat frontal cortex.**

Mieko Morishima[1] ([1]*Inst Clin Med Res, Jikei Univ Sch Med*)

To understand the function of the frontal cortex, it is important to investigate how the information are controlled by various types of excitatory and inhibitory cells. Pyramidal cells in layer 5 (L5) are final output neurons to the subcortical and other cortical areas. There are two major subtypes of pyramidal cells in L5 of the rat frontal cortex: one is crossed corticostriatal (CCS) cells projecting to the ipsi- and contralateral striatum, and the other is corticopontine (CPn) cells projecting to the ipsilateral pons. We have revealed that these two subtypes were different in their physiological and morphological properties as well as synaptic connection patterns.
The outputs of these L5 pyramidal cells can be regulated by neighboring inhibitory neurons: fast-spiking (FS) and low threshold spike (LTS) cells. To reveal how these inhibitory neurons inhibit the two types of pyramidal cells, we investigated their inhibitory connections. FS cells similarly innervated with both CCS and CPn cells, whereas LTS cells interact mostly with CPn cells. From these results, we demonstrated that the intracortical excitation-inhibition system is based on the pyramidal projection patterns.
(COI:No)

### SY33-4
**Morphological Re-evaluation of the Network in Basal Ganglia**

Fumino Fujiyama[1], Fuyuki Karube[1] ([1]*Lab Hisol Cytol, Grad Med Sch, Hokkaido Univ*)

The basal ganglia may be involved in motor execution, regulation, and reward-based learning processes. This regulatory system is widely accepted as a direct and indirect pathway scheme, in which there is an independent and antagonistic pathway in the basal ganglia. In recent years, however, there have been quite a few reports urging revalidation of this scheme among studies in which cell type specific neural pathways have been manipulated in optogenetics and other fields. Also, with the help of methodological advances, pathways that are not compatible with conventional direct and indirect pathway schemes have been discovered, such as neurons that project only from the external segment of the globus pallidus (GP) to the striatum (Arkypallidal Neuron), collaterals of striatal direct pathway neurons to the GP, and innervation from the GP to dopamine neurons. In addition, there is a chemical compartment in the striatum called the striosome/matrix, and the relationship between this structure and the basal ganglia network is being elucidated. In this session, we will discuss the possibility of a new basal ganglia scheme while introducing our new findings.
(COI:No)

### SY33-5
**Brain mechanisms how physical exercise can affect pain sensation and emotion**

Emiko Senba[1,2], Katsuya Kami[2,3] ([1]*Dept Phys Ther, Osaka Yukioka Coll Health Sci,* [2]*Dept Rehab Med, Wakayama Med Univ.,* [3]*Dept Rehab, Wkayama Faculty, Takarazuka Univ Med & Health Care*)

Voluntary exercise (VE) in neuropathic pain (NP) model mice reduced pain behavior (exercise-induced hypoalgesia: EIH), which is due in part to the activation of DA neurons in the ventral tegmental area (VTA) projecting to the nucleus accumbens (NAc). Glutamate neurons in the basolateral amygdala (Amy) (BLA), medial prefrontal cortex (mPFC) and ventral hippocampus (vHPC) also project to the NAc. These brain regions are called *mesocortico-limbic system* and play a key role in emotional/motivational processes in the brain.
We show that this system is deactivated in mice suffering NP by partial sciatic nerve ligation (PSL), and VE activates this system to lead to EIH. In mice allowed to run for 2 weeks after PSL, BLA-Glu neurons projecting to the NAc were activated to produce EIH and positive defense response, while central Amy-GABA neurons were deactivated by VE. PSL activated GABA neurons and deactivated pyramidalneurons in the mPFC, while the latter neurons were activated by VE. Moreover, PSL activated vHPC-Glu neurons projecting to the Amy and VE prevented this activation to promote extinction of fear memory.
Thus, VE/active lifestyle can prevent pain chronification and improve QOL.
(COI:No)

### International Symposium1

### Organelle quality control and its pathophysiological significance

(March 29, Mon. 9：00～11：00, Room1)

### IS1

### Organelle quality control and its pathophysiological significances

Nobuhiko Ohno[1], Motohiro Nishida[2,3], ([1]*Jichi Medical University, School of Medicine,* [2]*Kyushu University, Graduate School of Pharmaceutical Sciences,* [3]*Exploratory Research Center on Life and Living Systems [National Institute for Physiological Sciences]*)

Quality control of intracellular organelles is important for maintaining functional homeostasis of cells and tissues. Morphological and structural abnormalities of organelles lead to systemic dysfunctions and the onset of diseases. In this symposium, we will focus on the quality control of mitochondria, the master organelle linked to systemic respiration and energy metabolism, and the latest anatomy on the relationship between structural changes and functional abnormalities of mitochondria. By integrating anatomy and physiology, we aim to create innovative medical strategies targeting mitochondrial quality control.

### IS1-1

### Mitochondrial quality control in cardiac homeostasis and disease

Akiyuki Nishimura[1], Kakeru Shimada[1], Tomohiro Tanaka[1], Kazuhiro Nishiyama[2] Motohiro Nishida[1,2] ([1]*Div. Cardiocirculatory Signal., NIPS,* [2]*Dept. Physiol., Grad. Sch. Pharm. Sci., Kyushu Univ.*)

Proper mitochondrial quality control is indispensable for cardiac homeostasis and its defects are implicated in the development of cardiac diseases. We found that defective mitochondrial dynamics through aberrant interactions between mitochondria and actin cytoskeleton are a key determinant of cardiac remodeling and fragility. Dynamin-related protein 1 (Drp1), a mitochondrial fission-accelerating protein, was activated in myocardium after myocardial infarction, which induced mitochondrial fission-associated myocardial early senescence. Hypoxic stress induced the interaction of Drp1 with the actin-binding protein filamin A, leading Drp1 activation and mitochondrial fission-associated myocardial senescence in an actin binding-dependent manner in cardiomyocytes. Drp1-filamin A interaction was regulated by polysulfidation-depolysulfidation cycle of Drp1 at Cys624, a redox-sensitive cysteine residue. Electrophile-mediated depolysulfidation of Drp1 promoted the interaction with filamin A and induced mitochondrial hyperfission. Our results suggest therapeutic potential targeting pathology-dependent Drp1-filamin A interaction for the treatment of chronic heart failure. (COI:No)

### IS1-2

### BH4 activates CaMKK2 and rescues the cardiomyopathic phenotype in rodent models of diabetes

Hyoung Kyu Kim[1], Ippei Shimizu[2], Tohru Minamino[2], Bernd Nilius[3], Jin Han[1], ([1]*Department of Physiology, BK21 Plus Project Team, College of Medicine, Smart Marine Therapeutics Center, Cardiovascular and Metabolic Disease Center, Inje University, Busan, Korea,* [2]*Department of Cardiovascular Biology and Medicine, Niigata University Graduate School of Medical and Dental Sciences, Niigata, Japan.* [3]*Katholieke Universiteit Leuven, Department of Cellular and Molecular Medicine, Leuven, Belgium.*)

Diabetic cardiomyopathy (DCM) is a major cause of mortality/morbidity in diabetes mellitus patients. Although tetrahydrobiopterin (BH4) shows therapeutic potential as an endogenous cardiovascular target, its effect on myocardial cells and mitochondria in DCM and the underlying mechanisms remain unknown. Here, we determined the involvement of BH4 deficiency in DCM and the therapeutic potential of BH4 supplementation in a rodent DCM model. We observed a decreased BH4:total biopterin ratio in heart and mitochondria accompanied by cardiac remodeling, lower cardiac contractility, and mitochondrial dysfunction. Prolonged BH4 supplementation improved cardiac function, corrected morphological abnormalities in cardiac muscle, and increased mitochondrial activity. Proteomics analysis revealed oxidative phosphorylation (OXPHOS) as the BH4-targeted biological pathway in diabetic hearts as well as BH4-mediated rescue of down-regulated peroxisome proliferator-activated receptor-$\gamma$ coactivator 1-$\alpha$ (PGC-1$\alpha$) signaling as a key modulator of OXPHOS and mitochondrial biogenesis. Mechanistically, BH4 bound to calcium/calmodulin-dependent protein kinase kinase 2 (CaMKK2) and activated downstream AMP-activated protein kinase/cAMP response element binding protein/PGC-1$\alpha$ signaling to rescue mitochondrial and cardiac dysfunction in DCM. These results suggest BH4 as a novel endogenous activator of CaMKK2.

### IS1-3

### Prevention of mitochondrial impairment by inhibition of protein phosphatase 1-Drp1 cascade in amyotrophic lateral sclerosis

So Yoen Choi[1], Ah-Young Chung[2], Hae-Chul Park[2], Hyun Kim[1], Ju-Hyun Lee[1], Jae Ryun Ryu[1], Woong Sun[1] ([1]*Department of Anatomy;* [2]*Graduate School of Medicine, Korea University College of Medicine, Republic of Korea*)

Amyotrophic lateral sclerosis (ALS) is a fatal neurodegenerative disease caused by progressive loss of motor neurons (MNs) and subsequent muscle weakness. These pathological features are associated with numerous cellular changes, including alteration in mitochondrial morphology and function. However, the molecular mechanisms associating mitochondrial structure with ALS pathology are poorly understood. In this study, we found that Dynamin-related protein 1 (Drp1) was dephosphorylated in several ALS models, including those with SOD1 and TDP-43 mutations, and the dephosphorylation was mediated by the pathological induction of protein phosphatase 1 (PP1) activity in these models. Suppression of the PP1-Drp1 cascade effectively prevented ALS-related symptoms, including mitochondrial fragmentation, mitochondrial complex I impairment, axonal degeneration, and cell death, in primary neuronal culture models, iPSC-derived human MNs, and zebrafish models in vivo. These results suggest that modulation of PP1-Drp1 activity may be a therapeutic target for multiple pathological features of ALS.

### IS1-4

### Volume electron microscopy of mitochondria-ER interactions in axons

Nobuhiko Ohno[1,2] ([1]*Dept Anat, Div Histol Cell Biol, Jichi Med Univ,* [2]*Div Ultrastruct Res, Nat Inst Physiol Sci*)

Mitochondria play essential roles in axons and support axonal metabolism and neurotransmission. While the physical interaction between mitochondria and endoplasmic reticulum (ER) is critical for mitochondrial dynamics and functions, their regulatory mechanisms and roles in axons are still elusive. In our previous studies, volume electron microscopy was used for 3 dimensional analyses of the mitochondria-ER association in myelin diseases. The physical association was diminished prior to axonal degeneration in myelin mutant mice, while the association was enhanced under chronic progressive demyelination along with mitochondrial enlargement and increased expression of Mfn2. Subsequent analyses showed that neuronal ablation of MITOL, which facilitates oligomerization of Mfn2, diminished the mitochondria-ER association in axons and increased oxidative stress and mitochondria with abnormal morphology. These results suggest that the mitochondria-ER interaction is modulated through molecular tethering between mitochondria and ER, and contributes to mitochondrial integrity and survival of axons in myelin diseases. (COI:No)

### International Symposium2

### In search of new concepts of the basal ganglia by new techniques

(March 30, Tue. 9：00～11：00, Room1)

### IS2

**In search for new concepts of the basal ganglia by new techniques**

Fumino Fujiyama[1], Atsushi Nambu[2] ([1]*Laboratory of Histology and Cytology, Faculty of Medicine, Hakkaido University,* [2]*Division of System Neurophysiology, National Institute for Physiological Sciences*)

Basal ganglia are a group of nuclei in the cerebrum, and play important roles in motor control, action selection and motor learning. Their malfunctions cause severe movement disorders as observed in Parkinson's disease and dystonia. Recent development of new techniques, such as optogenetic and chemogenetic manipulations, fine tracing of neuronal projections visualized by viral vectors, calcium imaging and single-cell sequencing, have shone light on their intricate circuitries, functions and dysfunctions, and revolutionized our concepts on the basal ganglia. In this symposium, four speakers will present their new data and discuss future directions of basal ganglia research.

### IS2-1

**Exploring novel neural circuits involved in the external segment of globus pallidus**

Fuyuki Karube[1], Fumino Fujiyama[1] ([1]*Grad. Sch. Med., Hokkaido Univ.*)

Recent advances in neuroscience has been unveiling a number of cell type dependent rules of neural connections. We now focus on neural circuitries of the external segment of globus pallidus (GP in rodents). GP is composed of two types of projection neurons. Prototypic neurons mainly project to downstream nuclei of BG, as well as to the striatum (Str). In contrast, arkypallidal neurons exclusively project to Str.

Using PV-Cre rats and neural tracing with adeno-associated virus (AAV), we found PV-expressing prototypic neurons project to SN pars compacta. We confirmed that PV-expressing axon terminals of prototypic neurons indeed formed functional synapses on dopaminergic neurons.

Recently, we also discovered strong selective projection from GP to Str. Both arkypallidal and prototypic neurons highly preferred the matrix compartment of Str to the striosome one. In addition, selective innervation was also found to the matrix from the primary motor cortex and thalamus, and to arkypallidal neurons from the motor cortices. Taken together, these cell type and compartment dependent circuitries can contribute to appropriate generation and control of motor and learning processes related to BG. (COI:No)

### IS2-2

**Novel midbrain pathways modulating basal ganglia function**

Juan Mena-Segovia[1] ([1]*Center for Molecular and Behavioral Neuroscience Aidekman Research Center, Rutgers University, Newark NJ, USA*)

Cholinergic neurons of the midbrain, located in the pedunculopontine (PPN) and laterodorsal tegmental nuclei, are interconnected with all the basal ganglia structures, as well as with motor centers in the brainstem and medulla. Recent studies suggest that PPN cholinergic neurons exert a modulatory role in adaptive behavior involving both motor and cognitive functions. Along with cholinergic neurons, GABAergic and glutamatergic neurons constitute the other two large neuronal populations of the PPN, but little is known about their function. We have used anatomical, electrophysiological and behavioral methods to dissect the contribution of each neuronal population. Despite the difference in connectivity and neurochemical composition of all three populations, our recent functional studies suggest that all neurons have converging roles on the inhibition of ongoing behavior and the reinforcement of new actions mediated mainly by their projections to the basal ganglia. I will present our recent findings based on anatomical and functional studies obtained from the dissection of these midbrain circuits and will propose a unified theory that explains their contribution to behavior.

### IS2-3

**Action Specific Neuronal Activity of the Striatal Direct and Indirect Pathways for Goal Directed Behavior**

Satoshi Nonomura[1] ([1]*Primate Research Institute Kyoto University*)

The basal ganglia play key roles in goal-directed behavior guided by action-outcome history. However, few studies have tested how action and outcome signals are processed for goal-directed behavior through the striatal direct and indirect pathway (dSPN and iSPN). In previous study (Nonomura et al., 2018), we found that dSPNs encoded reward outcomes, whereas iSPNs encoded no-reward outcome and next-action selection. In recent study, we examined signals of iSPNs in the dorsolateral striatum (DLS) and compared with those in the DMS and found that iSPNs in the DLS did not discriminate the reward from no-reward outcomes, which was in sharp contrast to iSPNs in the DMS. Furthermore, iSPNs in the DLS increased their activity just after movement onset with dynamically-change according to the action-specific reward expectation and velocity, while those in the DMS did not show such responses. These results suggest the outcome-dependent nature of iSPNs in the DLS for motivated action and the DMS for outcome-based updating the action selection. (COI:No)

### IS2-4

**Non-canonical codes for behavioral sequences in basal ganglia outputs**

Jeongjin Kim[1,2] ([1]*Brain Science Institute, Korea institute of science and technology [KIST], Seoul, South Korea,* [2]*Division of Bio-Medical Science & Technology, University of science and technology [UST], Daejeon, South Korea*)

The start or end of an action sequence is an essential brain function. To do this, each behavioral program must be properly linked to the cognitive process and turned on or off depending on the situation. When they collapse, it causes many severe neurological disorders. Although basal gangalia output regions including thalamus have massive convergence inputs from the various motor system including cortex, cerebellum and basal ganglia, the underlying mechanism with behavioral sequences are largely unknown. Our goal is to unravel the neural circuits and specific cell types that are important to turn a series of actions on and off. Here, we tried to identify the role of basal ganglia output structures in the generation of action using optogenetic tools, deep brain calcium imaging, and whole-brain activity mapping. Combining these results, we found that the link between the basal ganglia and output structures can control the sequence of action. With a mathematical classification algorithm, we observed the specific neuronal ensemble for action in output structures. Also, we are going to introduce novel cell types of output structures that are important to control neuronal ensemble related to behavioral sequences. These suggest that this novel circuitry and neuronal ensemble in those regions might be a new therapeutic target for neurological disorders that show impaired action generation. Furthermore, this can lead to a new neural coding method for behavioral sequences hidden in basal ganglia output structures.

### Collaborative Session1

### Bottleneck and solution in order to carry out cadaver surgical training (CST) - 3

(March 28, Sun. 16：30～18：30, Room1)

### CS1-1

**Reconsideration of issues in CST implementation based on questionnaire results**

Yasunori Sakakura[1] ([1]*Dept Clin Lab Sci, Sch Med Tech, Health Sci Univ Hokkaido*)

Based on questionary survey concerning CST and technical staffs that Japanese Association of Anatomists carried out in 2018, we present several issues in CST. First, lack or undevelopment of facilities, deterioration of equipment, and security of training space including infection prevention are found out in the survey. Secondarily, the number of teaching and technical staffs is insufficient. The number of teaching staffs was reduced by reduction of administration grant or unification of the departments. The institution that there was not an exclusive staff occupied 70%, and increased workload of technical staffs was more than 80%. These issues will cause a difficult situation in securing of successor. Thirdly, there is shortage of the donated body. The reasons were the limited number accepted in the facilities and inability to anticipate increase of the donated body. These issues are compositely associated with the management task of the donated body used in CST, undesirable overwork on a holiday and on the weekend, and financing and management of the associated expense. We will discuss the bottleneck and solutions in order to perform CST. (COI:No)

### CS1-2

**Challenges for clinical physicians to continue CST**

Yoshio Araki[1], Kuniaki Tanahashi[1], Kinya Yokoyama[1], Kenji Uda[1], Ryota Saito[1], ([1]*Department of Neurosurgery, Nagoya University Graduate School of Medicine*)

In the recent years, with the development of new techniques and devices for surgery, it is required to acquire advanced knowledge and techniques to safely perform surgery by using them appropriately. Since the establishment of the Clinical Anatomy Laboratory Nagoya (CALNA) at Nagoya University, CST seminars have been held more than 30 times. More than 300 doctors participated until today. Based on that achievement, the "Tokai National University Hospital Organization CST Network Project" was adopted. In this project, a network of CST distance lecture / practice systems was established with four national universities in the Tokai area. On the other hand, it is necessary to avoid an excessive burden on the department of anatomy by conducting CST seminars. Surgeons who are in charge of CST management need to assist the management of cadaveric sources, secure operating funds, and plan seminars in addition to daily clinical practice. In this presentation, we would like to introduce the history and outline of CALNA at Nagoya University, and discuss the recent issues and future vision for continuation of CST from the viewpoint of clinicians.

### CS1-3

**Present status and prospects for cadaver surgical training and clinical research**

Toshiaki Shichinohe[1,2], Satoshi Hirano[1], Norihiro Sato[2], Masahiko Watanabe[3] ([1]*Department of Gastroenterological Surgery II, Hokkaido University Faculty of Medicine* , [2]*Center for Medical Device Development, Hokkaido University Hospital* , [3]*Department of Anatomy and Embryology, Hokkaido University Faculty of Medicine*)

Since the publication of "Guidelines for Cadaver Dissection in Education and Research for Clinical Medicine" in 2012, surgical training and clinical research using donated cadavers have been officially available in Japan.
The MHLW's budget for CST increased in fiscal 2018, and the number of universities which organize CST has increased from 16 to 33.
In fiscal 2019, MEXT started an "Advanced Medical Human Resource Training Program" of "Surgical Anatomy and Surgery", which aims to develop human resources in the field of surgical education, clinical anatomy, and R&D utilizing cadavers. A joint proposal for this educational program by Hokkaido, Kyoto and Chiba Universities was selected for the grant, and the program started in 2020.
To promote R&D using cadavers in Hokkaido University, we set up the "Center for Medical Device Development" thanks to financial support from the AMED's grant from fiscal 2019.
We also established a comprehensive education and research platform using cadavers called "CAST Project". We believe this project will provide surgical innovations that will lead to advanced and safe surgery and innovative medical devices in the future. (COI:No)

### CS1-4

**Current status and issues of cadaver surgical training facing the Japanese Society of Oral and Maxillofacial Surgeons**

Yasuyuki Shibuya[1], Hiroshi Kurita[1], Masahiro Umeda[1], Akira Katakura[1], Yoshimasa Kitagawa[1], Tadaharu Kobayashi[1], Akira Sasaki[1], Daichi Chikazu[1], Akihiro Miyazaki[1], Tetsuya Yoda[1], Tadaaki Kirita[1] ([1]*the Japanese Society of Oral and Maxillofacial Surgeons*)

The Japanese Society of Oral and Maxillofacial Surgeons established an ad hoc committee on cadaver surgical training (CST) in 2015, which was promoted to a standing committee as the CST committee in 2017. The CST committee consists of one chairman, one vice chairman, and eight members. CST is conducted about two or three times a year in collaboration between our academic society and the university that hosts the CST. The academic society has provided jaw bone cutting tools and surgical instruments, and the CST committee has created a system to lend these tools to the host university free of charge. Furthermore, the committee has revised the rules for training session so that participants can receive training credits for the specialist qualification system. However, we are facing some issues as the rules concerning CST differ among the various host universities to some degree and therefore require some adjustment. In this session, we will discuss the current status of and issues facing the CST that the Japanese Society of Oral and Maxillofacial Surgeons is working to address. (COI:No)

### Collaborative Session2

### Rehabilitation approach involved in emotional science and related physiological and anatomical evidence

(March 28, Sun. 16：30～18：30, Room2)

### CS2-1

**Therapeutic intervention and neural basis of pain**

Yusuke Ohmichi[1], Mika Ohmichi[1] ([1]*Dept Anato II, Kanazawa Med Univ*)

The appearance of negative emotions such as pain-induced expressions, shudder, and a sense of respiration urgency is accompanied by increased muscle tone. Conversely, pain causes kinesiophobia, suppressing physical activity and eventually resulting in muscle disuse. If overuse and disuse continue for a certain period, muscles can be damaged and become a major cause of pain. Here, we have focused on muscle disuse and analyzed the mechanism of pathological pain caused by the disuse. We clarified that the activation of the pain emotional pathway caused by nociceptive input starting from the oxidative injury of disuse muscle is involved in the pathogenesis of pain. It seems that excessive release of dopamine in the amygdala, which is the center of negative emotions, contributes to the spread of pain beyond the injured area. Accordingly, it is suggested that not only the prevention of nociceptive input but also the care of negative emotions is necessary to prevent chronic pain. In this symposium, I will focus on the disuse pain, and discuss the possibility of physical therapy for the emotional component of pain based on the author's clinical experience and basic science. (COI:No)

### CS2-2

**Changes in stress response circuit and anxiety disorders after brain injury**

Takashi Tanaka[1] ([1]*Department of Anatomy II, Kanazawa Medical University*)

Abnormalities in limbic neural circuits due to brain injury have been implicated in the onset of anxiety disorders. However, the molecular pathogenesis underlying anxiety disorders remains poorly elucidated. Herein, we showed that myristoylated alanine-rich C-kinase substrate like 1 (MARCKSL1) regulates the amygdala circuitry to control the activity of the hypothalamic-pituitary-adrenal (HPA) axis as well as induces anxiety-like behaviors in mice. MARCKSL1 expression was predominantly localized to the prefrontal cortex (PFC), hypothalamus, hippocampus, and amygdala of the adult mouse brain. MARCKSL1 expression was increased in the PFC and amygdala in a brain injury model associated with anxiety-like behaviors. Furthermore, MARCKSL1 transgenic (Tg) mice exhibited anxiety-like behaviors mediated by corticotropin-releasing hormone. MARCKSL1 increased spine formation in the central amygdala, and downregulation of MARCKSL1 in the amygdala normalized both increased HPA axis activity and elevated anxiety-like behaviors. Our findings suggest that MARCKSL1 expression in the amygdala plays an important role in inducing anxiety-like behaviors. (COI:No)

### CS2-3

**The rehabilitative effects involved in the emotion: implication in animal and human study**

Susumu Urakawa[1,2], Trung Duc Le[1], Kazuki Watanabe[1], Hiroyuki Ogawa[1], Taketoshi Ono[2], Hisao Nishijo[2], Naoto Fujita[1] ([1]*Department of Musculoskeletal Functional Research and Regeneration, Hiroshima University, Japan,* [2]*System Emotional Sci, Grad Sch Med Pharmaceutical Sci, Univ of Toyama, Japan*)

The rehabilitative interventions have been applied to patients who suffered cerebral stroke, bone and joint diseases, neuromuscular diseases, etc. These patients had encountered their disease and suddenly faced to the disabilities of daily living. The emotion is one of the notions known as an important role in decision or self-orientation in front of biological crisis. However, little report is available on rehabilitative effects involved in the emotion. We previously have reported that external stimuli or pathological changes of diabetes induced neuronal plasticity including the amygdala, prefrontal cortex, and alterations of emotional behavior in the animal studies. Moreover, we have shown the important role of rostromedial prefrontal cortex on eye-contact communication of infants, rehabilitative precision-task and robot-human interactions in human studies. These data suggest that the emotion-related brain areas including the amygdala, rostromedial prefrontal cortex play an important role in order to restore the patient's disabilities. (COI:No)

### CS2-4

**The modulation of sensorimotor cortical activity by the VTA stimulation**

Nobuo Kunori[1], Taichi Goto[1,3], Kei Ishii[2] ([1]*AIST, Neurorehabilitation RG,* [2]*AIST, MPFMRG,* [3]*Univ. of Tsukuba, Grad. Sch. of Comp. Hum. Sci.* )

In the field of motor rehabilitation, the patient's motivation is thought to be an important factor to improve the motor performance and to facilitate the motor recovery. However, the neuronal mechanisms linking motivational processes with sensorimotor system remain unclear. In this talk, we first introduce the neuronal activity of the sensorimotor cortex of rat evoked by single-pulse stimulation to the ventral tegmental area (VTA); the ventral midbrain region related to both motivation and motor functions. We also show the activity pattern of the VTA neurons obtained by calcium imaging (fiberphotometry) in awake rat during the reaching behavior. The results suggest that the salient sensory stimuli could activate VTA and modulate the motor command. As the second part, we introduce the involvement of VTA on the level-of consciousness. The burst electrical stimulation of VTA during the anesthesia-induced comatose state increased the level-of consciousness indicated by local field potentials pattern in the sensorimotor cortex of rat. The VTA neurons might influence the sensorimotor cortical activity by modulating both baseline activity level and individual task-dependent activity. (COI:No)

### Collaborative Session3

### Nerve - cancer/immunity crosstalk

(March 29, Mon. 14：20～16：20, Room2)

### CS3-1

**Cancer-nerve interplay**

Atsunori Kamiya[1] ([1]*Cellular Physiol, Med Sch, Okayama Univ*)

In the present symposium, I will talk about nerve-cancer interplay based on recent publications (Nature Neuroscience 2019, https://www.nature.com/articles/s41593-019-0430-3). There is growing evidence that these nerves link pathophysiological state more than expected. I revealed that sympathetic and parasympathetic nerves innervated tumor microenvironment of patients with breast cancer. Increased sympathetic and decreased parasympathetic nerve density in tumors were associated with poor clinical outcomes. Since existing methods (drugs, surgical resection or electrical stimulation of nerves) are limited to the selective manipulation of local nerves, we developed a series of genetic techniques to manipulate autonomic innervation in a tumor- and fiber-type-specific manner in mice and rats cancer models. Genetic sympathetic nerves stimulation in tumors accelerated breast cancer growth and progression, whereas genetic sympathetic denervation suppressed them. In contrast, genetic parasympathetic nerves stimulation decreased cancer progression, whereas parasympathetic denervation increased it. These results suggest that autonomic innervation of tumors regulates breast cancer progression. (COI:No)

### CS3-2

**Influence of pain on the immune control system**

Minoru Narita[1,2], Yusuke Hamada[1,2], Yukari Suda[1,2], Michiko Narita[2], Naoko Kuzumaki[1] ([1]*Department of Pharmacology, Hoshi University School of Pharmacy and Pharmaceutical Sciences,* [2]*Division of Cancer Pathophysiology, National Cancer Center Research Institute*)

The interrelationships between neurons and cancer cells have recently attracted attention in the field of cancer research. The formation of the tumor microenvironment is based on the accumulation of heterogeneous populations of cells, including blood vessels and immune cells, as well as cancer cells. Interestingly, it has been documented that sensory nerves also influence the tumor microenvironment. It has been speculated that cancer cells acquire their own proliferative and metastatic capacities by skillfully exploiting the protective function and regenerative process of living tissue. However, for further remodeling of the tumor microenvironment, it is presumed that the sensory neuron network surrounding the cancer cells is as important as blood vessels. A growing body of evidence suggests that axon reflexes in the terminal of sensory nerves are used not only to mediate inflammatory and immune responses, but also to interact with immune cells and cancer cells in the surrounding microenvironment. Here, we outline the significance of a deeper understanding of heterologous cell-cell interactions, such as "neuron-immune cells" or "neuron-cancer cells" and "analgesia". (COI:No)

### CS3-3

**Role of spinal glial cells in the chronicity of pain**

Makoto Tsuda[1] ([1]*Department of Molecular and System Pharmacology, Graduate School of Pharmaceutical Sciences, Kyushu University, Japan*)

While acute pain is a key defense system for detecting danger signals, chronic pain (like neuropathic pain occurring after nerve injury) is not simply a temporal continuum of acute pain but rather due to pathologically altered nervous system function. Our previous findings have indicated that these alterations occur not only in neurons but also in non-neuronal cells, especially spinal cord microglia, a type of resident immune cells of the central nervous system. In response to peripheral nerve injury (a model of neuropathic pain), microglia in the spinal dorsal horn respond rapidly, with alterations in morphology, cell number, and gene expression levels associated with an activated state. Accumulating evidence from studies enabling manipulation of these activation processes indicates that spinal cord microglia are necessary for the development of neuropathic pain. In this talk, I will highlight recent advances in our understanding of the role of spinal cord microglia in the development of neuropathic pain. (COI:No)

### CS3-4

**Crosstalk between sensory neurons and immune system through TRP channels**

Makoto Tominaga[1,2], Kenta Maruyama[1] ([1]*Div Cell Signal, Natl Inst Physiol Sci,* [2]*ExCELLS*)

Certain subtypes of nociceptors secret several peptides such as CGRP (calcitonin gene-related peptide) and substance P upon nociceptive stimulation. Increase in intracellular $Ca^{2+}$ concentrations is required for fusion with the plasma membrane of vesicles containing such peptides. TRP channels expressed in nociceptors especially TRPV1 and TRPA1 show high $Ca^{2+}$ permeability which contribute to the release of CGRP and substance P through exocytosis. The release of CGRP and substance P causes vasodilation and local inflammatory responses, a phenomenon called neurogenic inflammation. While it is well known that such neurogenic inflammation helps the recovery of damaged tissues, peptide release is also involved in many immune responses. Upon infection of *Canduda albicans*, b-glucan activated its receptor Dectin-1 in Nav1.8-positive nociceptors, followed by activation of TRPV1 and TRPA1. Then, released CGRP suppressed osteoinflammation and osteoclast multinucleation via direct suppression of NF-*k*B by the transcriptional repressor Jdp2 and inhibition of actin polymerization, respectively. These results suggest a critical role for Dectin-1-mediated sensocrine pathways in osteoinflammation. (COI:No)

### CS3-5

**Development of the drug that targets neurons, immune cells, and blood vessels to repair neuronal network for the central nervous system diseases**

Toshihide Yamashita[1] ([1]*Dept. Mol. Neuroscience., Grad. Sch. Med., Osaka Univ.*)

When the function of the CNS is damaged, for example, following spinal cord injury, cerebrovascular disorder, or brain trauma, it does not fully recover and there is still no effective treatment. Neural circuit reconstruction is essential to prevent such severe damage. However, the CNS has a mechanism that inhibits axonal regeneration. Several factors, such as proteins that inhibit axonal regeneration, play a role in the mechanism that maintains the stability of a neural circuit developed through a series of stages. We have identified multiple factors that negatively regulate the regeneration of damaged neural circuits and elucidated their molecular mechanisms. Especially, we found that repulsive guidance molecule (RGM) was a potent inhibitor of axon regeneration. Further, RGM regulates immune reaction and blood vessel integrity. Based on the basic research results obtained, we have developed a drug that controls "the biological system" and improves neurological symptoms. We are currently conducting international clinical trials of this drug. (COI:Properly Declared)

### Collaborative Session4

### Scientific basis of the oriental medicine: basic and clinical approaches

(March 29, Mon. 16：30～18：30, Room2)

### CS4-1

**Modulation of nociceptive sensitivity after electroacupuncture stimulation**

Yohji Fukazawa[1], Norikazu Kiguchi[2], Shiro Kishioka[2,3] (*[1]Dept Anatomy, Kansai Univ of Health Sciences, [2]Dept Pharmacol, Wakayama Med Univ, [3]Faculty of Wakayama Health Care Sciences, Takarazuka Univ of Med and Health Care*)

In order to maintain the proper sensitivity of sensory systems, our bodies are equipped with ambivalent mechanisms to adjust the subtle sensitivity. Antinociception induced by endogenous opioid peptides has essential protective effects against a stressful environment. On the other hand, prolonged antinociception leads to possible delay in fight-or-flight reactions resulting loss of chance for the survival. Thus, it is speculated that there are anti-opioid systems that normalize pain thresholds to permit adaptive response to a sense of danger. Indeed, paradoxical clinical feature associated with opioid treatment including analgesic tolerance and opioid-induced hyperalgesia has been observed and it has been suggested that anti-opioid system may involve in the paradoxical attenuation of antinociceptive effect of opioids.
We explored the effect of antinociception induced by electroacupuncture (EA) on the analgesic effect of morphine administrated after EA and found the analgesia induced by morphine after EA significantly attenuated. In this presentation, series of our studies demonstrate that possible underlying mechanisms of this paradoxical attenuation of morphine analgesia after EA. (COI:No)

### CS4-2

**Effects of the plantar acupressure-like stimulation on circulation and gastric pressure.**

Tomomi Narushima[1], Eitaro Noguchi[2] (*[1]Tsukuba University of Technology Center for Integrative Medicine, Department of Health, Faculty of Health Sciences, Tsukuba University of Technology, [2]Course of Acupuncture and Moxibustion, Department of Health, Faculty of Health Sciences, Tsukuba University of Technology*)

Acupressure treats the whole body, including the planta pedis. Treatments targeting the planta pedis are called reflexology, which are performed by treating the reflex zone of the body in the sole. Therefore, we conducted an experiment to confirm the response to pressure stimulation on a localized part of the planta pedis in terms of blood pressure (BP), heart rate (HR), and gastric pressure (GP), and its neural mechanism. Two sites between the 4th/5th and the 1st/2nd metatarsals of the left planta pedis of a rat, which correspond to the heart and stomach of the reflex zone of the human body, were used as stimulation sites. GP and BP tended to rise. However, only the stimulation between the 4th/5th metatarsals was statistically significant. HR tended to rise, but this response was not significant. Each of the responses observed was a reflex response whose afferent pathways consisted of cutaneous and muscle afferent nerves and whose efferent pathways consisted of the autonomic efferent nerves. Although the results failed to clarify the existence of the reflex zone, the effect of plantar acupressure-like stimulation on GP and BP was substantiated. (COI:No)

### CS4-3

**Stress-relieving effect of Kampo medicine**

Masataka Sunagawa[1] (*[1]Department of physiology, School of medicine, Showa university, Japan*)

Several Kampo medications are used to relieve stress, depending on the patients' symptoms. In this symposium, I will describe how Kampo medicine works in patients with stress using an example from basic study on Kamikihito (KKT).
KKT is clinically administered to patients with psychological symptoms, such as anxiety, depression and sleeplessness. Rats were exposed to a 90-min acute restraint stress procedure. Microdialysis and liquid chromatography tandem-mass spectrometry were used to monitor the secretion of oxytocin (OT), which has an anti-stress effect. The OT levels in rats treated with KKT were increased during and after stress loading. Furthermore, the anxiety-like behavior immediately after the acute stress loading was examined using an open field test. As a result, the total moved distance significantly decreased following the stress loading; however, the decrease was significantly inhibited by the administration of KKT. Moreover, the effect of KKT was obstructed by the pre-administration of an oxytocin receptor antagonist.
These results suggest that KKT has anti-stress activities and that increased OT secretion may be a mechanism underlying this phenomenon. (COI:Properly Declared)

### CS4-4

**Effect of acupuncture stimulation on excitability of motor nerve in humans**

Akira Nihonmatsu[1] (*[1]Hokkaido College of Oriental Medicine*)

It has been reported that acupuncture is effective in alleviating abnormalities of muscle tone caused by abnormal motor neuron excitability. Thus, we examined the effect of acupuncture stimulation on electromyogram F wave to determine the effect of acupuncture stimulation of various areas on excitability of spinal motor neuron.
Acupuncture stimulation to the LI4 or ST36 was significantly increased Amplitude ratio of F/M , decreased in ST4 . No effect of acupuncture stimulation to the CV12. We found that acupuncture to the extremity region increases the excitability of spinal motor neuron, acupuncture to the facial region decreases the excitability of spinal motor neuron.
Furthermore, we examined effects of acupuncture stimulation on short latency reflex (SLR) and long latency reflex (LLR) to determine the site of action of acupuncture stimulation in modulating motor reflexes. The amplitude ratio of SLR/M and LLR/M were reduced by the acupuncture stimulation of LI4 respectively. These findings suggest that acupuncture stimulation inhibits motor nerve reflexes via both spinal and supraspinal modulation systems. (COI:No)

### CS4-5

**Effects of cutaneous stimulation on serotonin release in the central nucleus of the amygdala**

Ryota Tokunaga[1], Rie Shimoju[2], Mieko Kurosawa[2,3] (*[1]Dept Neurosci, Jikei Univ Sch Med, Japan, [2]Center Med Sci, Intl Univ Health & Welfare, Japan., [3]Dept Pharm Sci, Intl Univ Health & Welfare, Japan*)

Somatosensory stimulations including acupuncture and moxibustion alter various functions. At the same time, they also evoke emotions such as anxiety and pleasure, and these emotions in turn affect the functions. In the present symposium we will introduce our studies on serotonin release in the central nucleus of the amygdala (CeA), which is known to be associated with anxiety and fear. We have investigated the effects of somatosensory stimulations, i.e., noxious (pinching) and innocuous (stroking) mechanical cutaneous stimulations, on the serotonin release in the CeA in anesthetized rats. Furthermore, its mechanisms were examined with special references to corticotropin releasing factor (CRF). Pinching of the skin increased serotonin release in the CeA, and the increase was mediated via type 2 CRF receptors in the dorsal raphe nucleus (dRN) where serotonergic neurons to the CeA originate. On the other hand, stroking decreased serotonin release in the CeA, and the decrease was mediated via type 1 CRF receptors in the dRN. Present results show that serotonin release in the CeA changes in response to cutaneous stimulations in stimulus modality-dependent way via CRF in the dRN. (COI:No)

### CS4-6

**Effect of acupuncture stimulation on facial blood flow**

Fumiko Yasuno[1] (*[1]Department of Acupuncture and Moxibustion, Faculty of Health Sciences Graduate School of Health Sciences, Tokyo Ariake University of Medical and Health Sciences*)

[Purpose] The effects of manual acupuncture (MA) on facial blood flow was examined by the Laser Speckle Method (PeriCam PSI by PERIMED).
[Method] The design is a crossover study. The participants were 10 healthy adult volunteers, and they were divided into three groups: the F Group (MA only on the face), FL Group (MA on the face + limbs), and C Group (no intervention). After resting with closed eyes for 20 minutes, acupuncture was performed for 15 minutes, and then the needle was removed and measurement was continued for 15 minutes. Intervention was carried out for 15 minutes. The acupuncture points were set to GB14, BL2, ST7, LI20, ST5 (face), LI10, LI4 (upper limb), ST36, ST37, and SP6 (lower limb) on the left side. Blood flow measurement sites were set to forehead, cheek, chin, space between the eyebrows, and nose tip.
[Results] In the F Group, blood flow increased markedly. Blood flow changes were observed bilaterally but there was no statistically significant difference between the left and right sides.
[Discussion and Conclusion] Increase in blood flow from acupuncture stimulation is considered to be due to the axonal reflex and the somatic autonomic nerve reflex. (COI:No)

### Collaborative Session5

### CLEM: the forefront of the method filling the gaps between light and electron microscopy

(March 30, Tue. 14：20～16：20, Room1)

### CS5-1

**Development of CLEM system and application for various biological samples**

Kiminori Toyooka[1] ([1]*RIKEN CSRS*)

Correlative light and electron microscopy (CLEM) is an analysis method that involves observing the same position of the same sample using light and electron microscopes (LM, and EM, respectively). We developed a CLEM system that can quickly and accurately acquire EM images with a field emission scanning electron microscope (FE-SEM) or a transmission electron microscope (TEM), in the same region observed with an LM. To observe the ultrastructure of fluorescently-labeled organelles, it is important to improve sample preparation processes such as fixation, embedding, and sectioning. For example, tissues/cells labeled with fluorescent proteins were embedded in a resin, and the signals of the fluorescent proteins were detected with a high-sensitivity confocal microscope; only then, CLEM images were obtained using an FE-SEM or TEM. In this symposium, I will introduce the development of CLEM systems, collaborate with companies, and discuss the CLEM sample preparation methods suitable for observation purposes in the context of various types of samples such as animal/plant tissues. Moreover, I will show CLEM data combining the latest light imaging with the newest EM technologies. (COI:Properly Declared)

### CS5-2

**The NanoSuit method: a novel histological approach for examining paraffin sections in a nondestructive manner by correlative light and electron microscopy**

Hideya Kawasaki[1] ([1]*Institute for NanoSuit Research, Preeminent Medical Photonics Education & Research Center, Hamamatsu University School of Medicine*)

Histological examination using the light microscopy is currently the gold standard for life science research and diagnostics. However, magnified observations are limited because of the limitations intrinsic to light microscopy. Thus, a dual approach, known as correlative light and electron microscopy (CLEM), has emerged. Previously, we developed the NanoSuit method, which enabled us to keep multicellular organisms alive/wet in the high vacuum of a scanning electron microscope by encasing the sample in a thin, vacuum-proof membrane.

Here, we apply the NanoSuit method to CLEM analysis of paraffin sections with the following features:

(i) the integrity of the glass slide is maintained,
(ii) three-dimensional microstructures of tissue and pathogens are visualized,
(iii) nuclei and 3,3′-diaminobenzidine stained areas are distinct because of gold chloride usage,
(iv) immunohistochemical staining is quantitative, and
(v) contained elements can be analyzed.

Removal of the SSE solution after observation is a further advantage, as this allows slides to be restained and stored. Thus, the NanoSuit method represents a novel approach that will advance the field of histology. (COI:No)

### CS5-3

**Correlative approach to understand neural circuits in the central nervous system**

Hirohide Iwasaki[1] ([1]*Dept Anatomy, Gunma Univ Grad Sch Med*)

The brain is composed of a vast number of neurons, which are connected via synapses to form neural circuits and realize a variety of brain functions. Because synapse is an ultramicroscopic structure, it is not possible to observe synapses in detail using optical microscopy due to the lack of resolution and the use of electron microscopy is necessary. On the other hand, because neurons form synapses with relatively distant neurons with long protrusions, optical microscopy is required to unravel the complex connections between neurons. Therefore, it is important to take advantages of both electron microscopy and optical microscopy for the comprehensive analysis of neural circuits and the development of CLEM approach is essential for understanding neural circuits. In recent years, remarkable progress has been made in the development of such application-oriented techniques, especially for automatic image acquisition and 3D reconstruction using electron microscopy. In this symposium, I will introduce several CLEM approaches, such as Array tomography, aiming to reveal the wiring diagram of the neurons in central nervous system. (COI:No)

### CS5-4

**Two-color in-resin CLEM of Epon-embedded cells**

Isei Tanida[1] ([1]*Dept Cell Mol Neuropahol, Junetndo Grad Sch Med*)

In-resin CLEM of Epon embedded samples can greatly simplify the correlation of fluorescent images with electron micrographs. For the achievement of two-color in-resin CLEM of Epon embedded cells, we screened for monomeric green and red fluorescent proteins that resist CLEM processing, and identified mWasabi, CoGFP variant 0, and mCherry2 (two green and one red fluorescent proteins) as alternatives suitable for in-resin CLEM. Cells expressing mitochondria-localized mCherry2 and histone H2B tagged with CoGFP variant 0 were fixed, and embedded in the Epon812 resins. Green and red fluorescence was detected in thin sections of the Epon-embedded cells. In the same thin sections, we correlated the fluorescent signals to mitochondria and the nucleus using a scanning electron microscope. Similar results were obtained when endoplasmic reticulum-localized mCherry2 and histone H2B tagged with CoGFP variant 0 were expressed in the cells. Two-color in-resin CLEM of two cytoplasmic organelles, mitochondria and endoplasmic reticulum, was also achieved. In conclusion, three fluorescent proteins are suitable for in-resin CLEM of Epon-embedded samples. (COI:No)

### Young Investigator Symposium1

### Development of human resources in anatomy: Initiatives and Proposals

(March 28, Sun. 14：20～16：20, Room8)

### YI1-1

**The Past and Future Role of the Association of Young Researchers**

Hiroaki Nabeka[1] ([1]*Department of Anatomy and Embryology, Ehime University Graduate School of Medicine*)

The preparatory committee "the Gathering of Young Researchers" was established in November 2018. After some months of preparation including questionnaires for young researchers, "the Association of Young Researchers (AYR)" was officially launched at the 124th Annual Meeting of The Japanese Association of Anatomists held in Niigata in March 2019.
The purpose of AYR is to contribute to the continuous development of anatomy through exchange between young researchers.
AYR is currently organized by three groups: the Exchange and Symposium Group, the Education and Research Career Group, and the Annual Meeting and Banquet Group.
As a project of the Exchange and Symposium Group, "Summer School" was held on August 24 and 25, 2019, at the Howa Seminar Plaza in Nagoya. On the first day, we held a panel discussion with young researchers from other societies and discussed future policies. On the second day, we discussed the terms and the future activities of AYR in a workshop.
The questionnaires and the summer school highlighted many concerns of young researchers. We want to make anatomy more exciting from the perspective of young researchers. (COI:No)

### YI1-2

**The important points to recruit clinical doctors to anatomic world, considering current clinical training system.**

Yoshinori Haizuka[1] ([1]*Department of Anatomy, Kyorin University Faculty of Medicine*)

How can we recruit new persons to anatomic field? This time, I will focus on the clinical training system. The history of clinical training system began as the on-the-job training system called the internship system established in 1946. This system had provided on-the-job training for one year before graduation, which was finished in 1968. Then another clinical training system was introduced. Although this clinical training system provided clinical training for two years after obtaining a medical license, but it was not compulsory. Therefore the system was revised in 2004, and all new graduates were obliged to undergo to the clinical training. The feature of this system is to give practice to the medical interns in the clinical departments of internal medicine, surgery, emergency, pediatrics, obstetrics and gynecology, psychiatry and community medicine. While introducing this current clinical training system, I will discuss the career path from a medical doctor to an anatomist based on my own experience. (COI:No)

### YI1-3

**The journey to anatomy**

Yuki Fujita[1,2] ([1]*Dept Mol Neurosci, Grad Sch Med, Osaka Univ*, [2]*IFReC, Osaka Univ*)

I studied pharmaceutical science in my undergraduate program. I never learned anatomy until I started my graduate studies. It had been over a decade now since I started learning anatomy. In this symposium, I will be sharing my journey on how I learned the basic and practical anatomical science and how I finally landed my first anatomy tutor role. (COI:No)

### YI1-4

**Desire to see, desire to understand**

Masaharu Yoshihara[1] ([1]*Ph.D. Program in Humanics, SIGMA, Univ. Tsukuba*)

The structure of the human body is a fine art of the nature in that its basic parts repeatedly emerge among individuals albeit very complicated. The gross anatomy class in School of Medicine, University of Tsukuba cultivated my desire to know the developmental principles behind this commonness of the human body. In addition, the research at Department of Cell Biology and Anatomy, Graduate School of Medicine, University of Tokyo has realized me how meaningful seeing is in our understanding. When anatomy is defined as a field of biology regarding shape, size and position, it deals with principles in the physiology and development of the body as well as description of structures. Importantly, these principles are exemplified in the cadaver body in the gross anatomy class, which might be either an invitation to anatomical research or a great challenge to many students. One of struggles in the recruitment might be how to invite the students in the gross anatomy class to the anatomical research. In this symposium, I would like to introduce some details of my experiences that have directed me to anatomical research. The keys are good research and excellent mentors. (COI:No)

### YI1-5

**Recruiting next-generation candidates of anatomists through the anatomy education**

Koji Ikegami[1] ([1]*Grad Sch Biomed and Health Sci, Hiroshima Univ,*)

A big problem of anatomy in Japan is the decrease in candidates for future anatomists. The main reason of the decrease is that almost all students in medical school become clinicians and that few students become basic researchers. However, several percent of medical students actually are interested in basic research including anatomy or morphology. Alternatively, some medical students do not know that medical schools perform research and provide students with opportunity for basic research until entering medical school. The presenter gives a talk about what are done to make students getting interested in basic research and recruit the "future anatomists" in Hamamatsu University School of Medicine, which is former workplace of the presenter, and Hiroshima University School of Medicine. Though being very simple, "soliciting" in and through the anatomy education that includes lectures and practical courses is important, the presenter thinks. (COI:No)

### YI1-6

**Promoting basic medical research in the department of anatomy: exploring my stance for**

Ryo Nitta[1] ([1]*Division of Structural Medicine and Anatomy, Kobe University Graduate School of Medicine*)

Anatomical education in the medical school is the essential training for medical students, although it is one of the hardest duties for teaching stuffs, especially for the non-MD stuffs. Also for the medical students, the anatomical training is one of the most heavy practices. Therefore, anatomy labs may not be popular for medical students or researchers, thus recruiting students or stuffs to the anatomy lab is a major problem to overcome. In this symposium, I will introduce my effort to promoting basic medical research in our lab, based on my experiences to date. I started my carrier as a medical cardiologist. Three years later, I decided to start basic research as a graduate student of the University of Tokyo, to seek to cure the diseases, especially the cardiomyopathies. I was also involved in the medical scientist training program in the University of Tokyo, giving me a good chance to realize high capability of medical students for medical research. I then moved to RIKEN in which I recognized a difference of properties between MD and non-MD scientists. I finally came to Kobe university in 2017 and am now exploring my stance to develop basic medical research in the anatomy lab. (COI:No)

### Young Investigator Symposium2

### Size control systems in biology: Approach from anatomy and physiology.

(March 28, Sun. 16：30～18：30, Room8)

### YI2-1

**Is organ size essential for its function? Utilizing heart size plasticity as a model**

Tatsuyuki Sato[1], Issei Komuro[1], Norihiko Takeda[2] ([1]*Department of cardiovascular medicine, University of Tokyo, Japatn,* [2]*Division of Cardiology and Metabolism, Center for Molecular Medicine Jichi Medical University*)

All mammalian organs have a specific size. However, it is still unclear how the size of an organ influences its physiological function. The heart is an organ that can change its size not only during growth but even in adulthood. When the adult heart is exposed to mechanical stress such as hypertension, the size of the heart increases (cardiac hypertrophy), and when the stress is removed, the size of the heart decreases back (hypertrophy regression). Our lab has been utilizing this size plasticity of the heart to uncover the relationship between anatomical organ size and physiological function. We have recently developed a new genetically engineered mouse strain which has defects in cardiac hypertrophy. In this presentation, we would like to introduce our recent findings using this mouse strain and discuss the role of size in organ function. (COI:Properly Declared)

### YI2-2

**Temperature dependency of Notch signaling in amniote brain development and evolution**

Tadashi Nomura[1] ([1]*Dev Neurobiol. Kyoto Pref Univ Med*)

Ambient temperature significantly affects developmental timing in animals. The temperature sensitivity of embryogenesis is generally believed to be a consequence of the thermal dependency of cellular metabolism. However, the adaptive molecular mechanisms that respond to variation in temperatures remain unclear. Here, we report species-specific thermal sensitivity of Notch signaling in the developing amniote brain. Transient hypothermic conditions enhanced canonical Notch activity and reduced neurogenesis in chick neural progenitors. Increased biosynthesis of phosphatidylethanolamine, a major glycerophospholipid that constitutes the plasma membrane, mediated hypothermia-induced Notch activation. Furthermore, the species-specific thermal dependency of Notch signaling was associated with developmental robustness to altered Notch signaling. Our results reveal unique regulatory mechanisms for temperature-dependent neurogenic potentials, which confer developmental and evolutionary adaptations to a range of ambient temperatures in amniotes. (COI:No)

### YI2-3

**Molecular basis of periodicity within macromolecular complexes**

Toshiyuki Oda[1] ([1]*Grad. Sch. Med. Univ. Yamanashi*)

Many of the macromolecular complexes within the cell have ordered architecture with discrete periodicities. The molecular mechanisms underlying those periodicities are considered to be related to the physiological function of the complexes. This talk features structural studies of three complexes: the ciliary axoneme, the thin filament and the Z-disc of cardiac muscle. The axoneme has a molecular ruler that determines the 96-nm repeat of the dynein motors. Along the thin filament, tropomyosin coiled-coils bind to one another in an end-to-end fashion, creating a 38.4-nm repeat. Within the Z-disc, the helical arrangement of the alpha-actinin dimers along the F-actin constitutes an 18×18 nm square lattice. Reconstruction of the 3D structures of these complexes using cryo-electron tomography and single particle analysis suggest a correlation between the ordered molecular arrangements and the cellular motility. (COI:No)

### YI2-4

**Analysis of YAP-mechanohomeostasis essential for the 3D organ formation**

Yoichi Asaoka[1], Makoto Seiki[1] ([1]*Department of Systems Biochemistry in Pathology and Regeneration, Yamaguchi University Graduate School of Medicine*)

Living organisms on the earth have evolved to develop homeostasis withstanding external mechanical forces including gravity. Deterioration of the homeostasis often leads to organ atrophy and reduction in regenerative capability, but its mechanism remains to be elucidated. To develop therapies, we have to understand the fundamental questions; how cells in an organ decide to proliferate and determine their sizes by sensing the whole 3D organ size. Through the analysis of the flat-bodied medaka fish mutant, *hirame* in which YAP transcriptional co-activator is mutated, we showed that YAP governs the mechanical properties and the organ physiology by the reciprocal physical interactions between the cells and their extracellular milieu including extracellular matrix and cell-cell contact, which we named YAP-mechanohomeostasis (Porazinski *et al. Nature* 2015, Asaoka and Furutani-Seiki. *Curr Opin Cell Biol* 2017). In this talk, we will present our study of YAP mutant fish and discuss the molecular mechanism of YAP-mechanohomeostasis in the 3D morphogenesis of vertebrates. (COI:No)

### YI2-5

**Epithelial cell-turnover ensures robust coordination of tissue growth in *Drosophila***

Shizue Ohsawa[1], Nanami Akai[1], Yukari Sando[2], Atsushi Igaki[2] ([1]*Div. Biol. Sci., Grad. Sch. Sci., Nagoy Univ.,* [2]*Grad. Sch. Biostudies, Kyoto Univ*)

Highly reproducible animal development is achieved by robust, time-dependent coordination of tissue growth. To study the mechanism that ensure robust coordination of tissue growth, we analyzed developmental processes of a *Drosophila* mutant called *Minute*, a heterozygous ribosomal protein mutant that shows significant developmental delay in their larval period but finally builds up normal flies without any significant defects in tissue/organ patterning. We found that both cell death and cell proliferation ("cell-turnover") were significantly increased in *Minute* wing imaginal discs. Blocking the cell-turnover by inhibiting cell death resulted in morphological defects, indicating the essential role of cell-turnover in *Minute*/+ wing morphogenesis. Interestingly, forced developmental delay in larval period by other means also triggered cell-turnover in wing imaginal discs. Our genetic analyses suggested the possibility that the cell turnover could be induced by Wingless (a Wnt homolog)-dependent cell competition. The mechanism of cell-turnover that ensures robust coordination of tissue growth in *Drosophila* will be discussed. (COI:No)

### Educational Programs

### Sponsored Seminars

### Joint Program on Education1

### Horizontal Integration

(March 28, Sun. 14：20～16：20, Room9)

**EP1-1** Horizontal integration among basic medicines: examples and challenges

Naohiko Anzai
Chiba University

**EP1-2** Curriculum Development for Horizontal Integration

Shuichi Watanabe
Saitama Medical University

**EP1-3** Integrated course of anatomy and physiology at Asahikawa Medical University

Shigetaka Yoshida
Asahikawa Medical University

**EP1-4** Strategies for Horizontal Integration in Medical University Education in Japan

Yo-ichi Sato
Iwate Medical University

### Joint Program on Education2

### Vertical Integration

(March 28, Sun. 16：30～18：30, Room9)

**EP2-1** Medical Education should be with the patient - Challenges at Showa University -

Miki Izumi
Showa University Faculty of Medicine

**EP2-2** Start with what we can do: Integrated life science education by clinical cases

Noriyuki Koibuchi
Gunma University

**EP2-3** From First-Year-Experience Education to Basic Medical Science Curriculum - A Trial at Ehime University -

Naoto Kobayashi
Ehime University School of Medicine

**EP2-4** A stream from natural sciences to basic sciences and clinical sciences - Science education in basic medical education-

Osamu Fukushima
The Jikei University School of Medicine

### Joint Program on Education3

### Model Lecture : Anatomy, Physiology, and Clinical Medicion on Kidney

(March 30, Tue. 9：00～11：00, Room7)

**EP3**

Koichiro Ichimura
Juntendo University

Yoshinori Marunaka
Kyoto Industrial Health Association

Toshiaki Monkawa
Keio University

### Joint Program on Education4

### Educational Workshop

(March 30, Tue. 16：30～18：30, Room7)

**EP4**

Michio Shiibashi
Saitama Medical University

Noriyuki Koibuchi
Gunma University

Susumu Minamisawa
School of Medicine

### Sponsored Seminar1

[ Supported by Bio Research Center Co.,Ltd. ]

### Multimodal imaging platform for Inscopix miniaturized fluorescence microscope and non-human primate application

(March 28・29・30, 12：10～13：10 , Room2)

**SS1** Alice Stamatakis, Anil Bollimunta
Inscopix

### Sponsored Seminar2

[ Supported by Intermedical Co.,Ltd. ]

### Innovative Cardiac Mapping Technologies for Pre-clinical Studies

(March 28・29・30, 12：10～13：10, Room3)

**SS2** Jay Lu
MappingLab

### Sponsored Seminar3

[ Supported by Leica Microsystems K.K. ]

### Advanced fluorescence lifetime imaging and next generation 3D screening

(March 28・29・30, 12：10～13：10, Room4)

**SS3-1** THUNDER creates the new normal of fluorescence microscopy
Nobuhide Tsurumaki
Leica Microsystems K.K.

**SS3-2** So easy! CLSM for everyone with fluorescence lifetime.
Suguru Osari
Leica Microsystems K.K.

### Sponsored Seminar4

[ Supported by Nikon Solutions Co., Ltd. ]

### Having said that, NIS. ai is difficult, isn’t it?

(March 28・29・30, 12：10～13：10, Room5)

**SS4** Takafumi Miyamoto
University of Tsukuba

### Sponsored Seminar5

[ Supported by FUJIFILM Wako Pure Chemical Corporation ]

### Application of CLEM fluorescent proteins to multi-color in-resin CLEM

(March 28・29・30, 12：10～13：10, Room6)

**SS5** Isei Tanida
Junetndo University

### Sponsored Seminar6

[ Supported by Thermo Fisher Scientific ]

### Cryo 3D imaging of cells and tissues by Cryo FIB-SEM

(March 28・29・30, 12：10～13：10, Room7)

**SS6** Tsubasa kai
Thermo Fisher Scientific

### Sponsored Seminar7

[ Supported by JEOL Ltd. ]

### RIKEN-JEOL Collaboration Center Research Introduction : Recent progress in structural biology by cryoTEM technology and application of large-scale SEM imaging for microstructural analysis of biological tissue.

(March 28・29・30, 12：10～13：10, Room8)

**SS7** Yosky Kataoka
RIKEN-JEOL Collaboration Center

### Sponsored Seminar8

[ Supported by Panasonic System Solutions Japan Co., Ltd. ]

(March 28・29・30, 12：10～13：10, Room9)

**SS8-1** Active learning outside the dissection lab with MeAV anatomy: Not just a fancy 3D viewer

Ryusuke Momota
Okayama University

**SS8-2** Improvement of Clinical Clerkship by the use of F.CESS

Toyohiko Sakai
University of Fukui

### Award Presentations

| | |
|---|---|
| **AP-1～AP-2** | $22^{nd}$ Promotion Award of the Physiological Society of Japan for Young Scientists |
| **AP-3～AP-5** | $11^{th}$ Hiroshi and Aya Irisawa Memorial Promotion Award for Young Physiologists |
| **AP-6** | $11^{th}$ Aya Irisawa Memorial Promotion Award for Excellence by Women Physiologists |
| **AP-7** | $11^{th}$ Hiroshi and Aya Irisawa Memorial Award for Excellent Papers in The Journal of Physiological Sciences |
| **AP-8～AP-9** | $11^{th}$ Hiroshi and Aya Irisawa Memorial Promotion Award for Excellent Papers on Research in Circulation in The Journal of Physiological Sciences |

### AP-1 (PP-66)

### Actin-dependent rapid tethering of synaptic vesicles accompanying exocytosis at a fast central synapse

Takafumi Miki[1], Mitsuharu Midorikawa[2], Takeshi Sakaba[1] ([1]*Grad Sch Brain Science, Doshisha Univ,* [2]*Dept Physiol, Sch Med, Tokyo Women's Med Univ* )

A high rate of synaptic vesicle (SV) release is required at cerebellar mossy fiber terminals for rapid information processing. As the number of release sites is limited, fast SV reloading is necessary for achieving sustained release. However, rapid reloading has not been observed directly. Here, we visualize SV movements near presynaptic membrane using total internal reflection fluorescence (TIRF) microscopy. Upon stimulation, SVs appeared in the TIRF-field and became tethered to the presynaptic membrane with unexpectedly rapid time-course, almost as fast as SVs disappearing due to release. However, such stimulus-induced tethering was abolished by inhibiting exocytosis or by actin disruption, suggesting that actin-dependent tethering is tightly coupled to preceding exocytosis. The newly-tethered vesicles became fusion-competent not immediately but only 300-400 ms after tethering. Together with model simulations, we propose that rapid tethering leads to an immediate filling of vacated spaces and release sites within <100 nm of the active zone by SVs, which serve as precursors of readily-releasable vesicles, thereby shortening delays during sustained activity. (COI:No)

### AP-2 (OP3-1)

### A psychosomatic pathway from the prefrontal cortex to the hypothalamus that drives physiological responses to psychological stress

Kataoka Naoya[1], Yuta Shima[1], Kazuhiro Nakamura[1] ([1]*Dep Integrative Physiol, Grad Sch Med, Nagoya Univ Japan,* [2]*Nagoya Univ Institute for Adv Res.*)

The brain network that evokes autonomic and behavioral responses for stress coping has been a major focus of physiological research. We have previously reported that psychological stress induces thermogenesis in brown adipose tissue, hyperthermia and tachycardia by activating a monosynaptic neural pathway from the dorsomedial hypothalamus (DMH) to the rostral medullary raphe. Recently, we discovered that the DMH receives glutamatergic excitatory stress inputs from the dorsal peduncular cortex and dorsal tenia tecta (DP/DTT), located at the ventral limit of the medial prefrontal cortex. Here, it was determined how important the DP/DTT–DMH neural pathway is for the drive of physiological stress responses. Optogenetic inhibition or selective lesions of DP/DTT–DMH projection neurons diminished thermogenic, skin vasoconstrictor, hyperthermic, tachycardic and pressor responses to social defeat stress. Optogenetic inhibition of the DP/DTT–DMH pathway also eliminated avoidance behavior from stressors. These results demonstrate that the DP/DTT–DMH pathway transmits a master psychosomatic signal that drives a variety of sympathetic and behavioral stress responses. (COI:No)

### AP-3 (MS9-1)

### Cyclic AMP-buffering by Olfactory Marker Protein ensures resilient olfactory neuronal activity in odor-source searching

Noriyuki Nakashima[1], Akiko Nakashima[1], Kie Nakashima[2], Akiko Taura[3], Harunori Omori[4], Makoto Takano[1] ([1]*Dept Physiol, Kurume Univ Sch Med, Fukuoka, Japan,* [2]*Lab Dev Neurobiol, Grad Sch Biostudies, Kyoto Univ, Kyoto, Japan,* [3]*Dept Med Engineer, Facult Health Sci, Aino Univ, Osaka Japan,* [4]*Dept Physiol, Sch Med, Kanazawa Med Univ, Ishikawa, Japan*)

Olfactory sensation occurs in the cilia of olfactory receptor neurons (ORNs) by converting the odorant-induced cAMP surge into $Na^+/Ca^{2+}$ influx via CNG channels. Under lasting stimulation, olfaction undergoes strong desensitization. However, ORNs must maintain resilient firing even during odor-source searching. We have discovered a novel mechanism that prevents neural desensitization under sensory stimuli. We identified a genetic signature of mature ORNs, the olfactory marker protein (OMP) as a cAMP-binding protein. OMP directly captures the odor-induced surplus cAMP and swiftly reduces the freely available cAMP. This cAMP-buffering process quickly terminates CNG activity, prevents excessive depolarization under repetitive stimulation and maintains the resilient firing responses. Behaviorally, $OMP^{-/-}$ mice made frequent errors in odor-source searching due to the cAMP-overload by continual sniffing. The cAMP-buffering by OMP proposes a novel cellular strategy for spatiotemporal regulation of cAMP-associated signaling in ORNs and other OMP-expressing cells throughout the body. (COI:No)

### AP-4 (SY24-2)

### Functional mechanism of polarized phosphoinositides distribution generated by voltage-sensing phosphatase in sperm flagellum

Takafumi Kawai[1], Yasushi Okamura[1] ([1]*Grad Sch of Med, Osaka univ*)

Voltage-sensing phosphatase (VSP) shows phosphoinositides phosphatase activity that is coupled to membrane potential. Previously, we reported that VSP-deficient sperm show severe defect in their motility after capacitation, resulting in significant reduction in success rate of fertilization in *in vitro* fertilization experiment. Electrophysiological analysis indicated that $K^+$ current that would be derived from Slo3, sperm specific $K^+$ channel is enhanced in $VSP^{-/-}$ sperm, and the polarized PtdIns(4,5)P2 distribution was important for regulating the Slo3 activity. Our results indicate that VSP appears to optimize PtdIns(4,5)$P_2$ distribution of the principal piece, contributing the normal sperm motility during capacitation. In spite of the important function of VSP in sperm physiology, we still do not know how and when such specialized PtdIns(4,5)$P_2$ distribution is formed by VSP activity. In the present study, we report the maturation-dependent VSP activity by examining sperm at different maturation stages. We also discuss the mechanism how membrane potential is important for regulating VSP activity during sperm maturation. (COI:No)

### AP-5 (OP32-5)

### Troponin T amino acid mutation (ΔK210) knock-in mice is good animal model for neonatal dilated cardiomyopathy.

Jun Tanihata[1], Teruyuki Fujii[1], Shunsuke Baba[1], Yoshitaka Fujimoto[1], Sachio Morimoto[2], Susumu Minamisawa[1] ([1]*Department of Cell Physiology, The Jikei University School of Medicine, Tokyo, Japan,* [2]*Department of Health Science Fukuoka, International University of Health and Welfare, Okawa, Japan*)

**Introduction:** Dilated cardiomyopathy (DCM) in children is exhibits distinct pathological entities from those of adult DCM. Due to the limited number of patients and the lack of a good animal model, the molecular mechanisms underlying pediatric DCM remain poorly understood. The purpose of this study is to establish an animal model of neonatal DCM and identify early progression factors.

**Methods:** Cardiac phenotypes and gene expression profiles in homozygous ΔK210 knock-in ($TNNT2^{\Delta K210/\Delta K210}$) mice were analyzed compared to $TNNT2^{+/\Delta K210}$ and wild-type mice at 0days and 1week of age.

**Results:** Immediately after birth, the cardiac weight in $TNNT2^{\Delta K210/\Delta K210}$ mice was already increased that in $TNNT2^{+/\Delta K210}$ and wild-type mice. Echocardiographic examination of 1-week-old $TNNT2^{\Delta K210/\Delta K210}$ mice revealed similar phenotypes of pediatric DCM. In addition, the KEGG PATHWAY analysis revealed several important pathways such as cancer and focal adhesion that might be associated with the pathogenesis and development of DCM.

**Conclusion:** $TNNT2^{\Delta K210/\Delta K210}$ mice have already developed DCM at birth, indicating that they should be an excellent animal model to identify early progression factors of DCM. (COI:No)

### AP-6 (PP-384)

### Postnatal white-to-brown conversion of adipose tissue in Syrian hamsters

Yuko Okamatsu-Ogura[1], Kazuki Nagaya[1], Junosuke Mae[1], Ayumi Tsubota[1], Junko Kobayashi[2], Kazuhiro Kimura[1] ([1]*Laboratory of Biochemistry, Faculty of Veterinary Medicine, Hokkaido University,* [2]*Laboratory of Histology and Cytology, Faculty of Medicine, Hokkaido University*)

Brown and white adipose tissues (BAT and WAT) are quite different in morphology and function; however, the boundary between these tissues is obscure. In this study, we evaluated the process of BAT formation in Syrian hamsters, which shows postnatal conversion of WAT to BAT. Histological analysis revealed that interscapular fat is occupied with white adipocytes at birth, and progenitors appear and proliferate to fill the whole tissue, and then differentiate into brown adipocytes. Immunostaining for uncoupling protein 1 (UCP1), a responsible protein for BAT thermogenesis, indicated tissue maturation as BAT by postnatal day 14. Consistently, pups before 14-day-old were unable to maintain body temperature at 23°C. Environmental temperature seems not to be critical for BAT formation because it was similar between the pups raised at 23°C and 30°C. Progenitors spontaneously differentiated into brown adipocytes *in vitro*, which was suppressed by co-culture with white adipocytes, indicating that inhibitory factors of progenitor differentiation are secreted from white adipocytes. These results suggest that white adipocyte disappearance is essential for the BAT formation in hamsters. (COI:No)

### JPS-1(AP-7)

### Class II phosphatidylinositol 3-kinase α and β isoforms are required for vascular smooth muscle Rho activation, contraction and blood pressure regulation in mice

Shahidul Islam[1], Kazuaki Yoshioka[1], Sho Aki[1], Kazuhiro Ishimaru[1], Hiroki Yamada[1], Noriko Takuwa[1,2], Yoh Takuwa[1] ([1]*Department of Physiology, Kanazawa University Graduate School of Medical Sciences,* [2]*Department of Health Science, Ishikawa Prefectural University*)

Class II phosphatidylinositol 3-kinases (PI3K), PI3K-C2α and PI3K-C2β, are involved in cellular processes including endocytosis, cilia formation and autophagy. However, the role of PI3K-C2α and PI3K-C2β at the organismal level is not well understood. We found that double knockout (KO) mice with both smooth muscle-specific KO of PI3K-C2α and global PI3K-C2β KO, but not single KO mice of either PI3K-C2α or PI3K-C2β, exhibited reductions in arterial blood pressure and substantial attenuation of contractile responses of isolated aortic rings. In wild-type vascular smooth muscle cells, double knockdown of PI3K-C2α and PI3K-C2β but not single knockdown of either PI3K markedly inhibited contraction with reduced phosphorylation of 20-kDa myosin light chain and MYPT1 and Rho activation, but without inhibition of the intracellular Ca2+ mobilization. These data indicate that PI3K-C2α and PI3K-C2β play the redundant but essential role for vascular smooth muscle contraction and blood pressure regulation mainly through their involvement in Rho activation.

### JPS-2(AP-8)

### Membrane current evoked by mitochondrial Na+–Ca2+ exchange in mouse heart

Mohammed M. Islam[1], Ayako Takeuchi[1,2], Satoshi Matsuoka[1,2] ([1]*Department of Integrative and Systems Physiology, Faculty of Medical Sciences, University of Fukui,* [2]*Life Science Innovation Center, University of Fukui*)

The electrogenicity of mitochondrial Na+–Ca2+ exchange (NCXm) had been controversial and no membrane current through it had been reported. We succeeded for the first time in recording NCXm-mediated currents using mitoplasts derived from mouse ventricle. Under conditions that K+, Cl−, and Ca2+ uniporter currents were inhibited, extra-mitochondrial Na+ induced inward currents with 1 μM Ca2+ in the pipette. The half-maximum concentration of Na+ was 35.6 mM. The inward current was diminished without Ca2+ in the pipette, and was augmented with 10 μM Ca2+. The Na+-induced inward currents were largely inhibited by CGP-37157, an NCXm blocker. However, the reverse mode of NCXm, which should be detected as an outward current, was hardly induced by extra-mitochondrial application of Ca2+ with Na+ in the pipette. It was concluded that NCXm is electrogenic. This property may be advantageous for facilitating Ca2+ extrusion from mitochondria, which has large negative membrane potential.

### JPS-3(AP-9)

### Ionic mechanisms of ST segment elevation in electrocardiogram during acute myocardial infarction

Jun-ichi Okada[1,2], Katsuhiko Fujiu[3,4], Kazunori Yoneda[5], Takashi Iwamura[5], Takumi Washio[1,2], Issei Komuro[3], Toshiaki Hisada[1], Seiryo Sugiura[1] ([1]*UT-Heart Inc.,* [2]*Future Center Initiative, The University of Tokyo,* [3]*Department of Cardiovascular Medicine, Graduate School of Medicine, The University of Tokyo,* [4]*Department of Advanced Cardiology, Graduate School of Medicine, The University of Tokyo,* [5]*Healthcare Solutions Unit, Fujitsu Limited*)

ST elevation on an electrocardiogram is a hallmark of acute transmural ischemia. However, the underlying mechanism remains unclear. We hypothesized that high ischemic sensitivities of epicardial adenosine triphosphate-sensitive potassium (IKATP) and sodium (INa) currents play key roles in the genesis of ST elevation. Using a multi-scale heart simulation under moderately ischemic conditions, transmural heterogeneities of IKATP and INa created a transmural gradient, opposite to that observed in subendocardial injury, leading to ST elevation. These heterogeneities also contributed to the genesis of hyper-acute T waves under mildly ischemic conditions. By contrast, under severely ischemic conditions, although action potentials were suppressed transmurally, the potential gradient at the boundary between the ischemic and normal regions caused ST elevation without a contribution from transmural heterogeneity. Thus, transmural heterogeneities of ion channel properties may contribute to the genesis of ST–T changes during mild or moderate transmural ischemia, while ST elevation may be induced without the contribution of heterogeneity under severe ischemic conditions.

### Oral Presentations

### Oral Presentation1

### Urinary organ, Renal function, Urination, etc.

(March 28, Sun. 9：00～10：12, Room9)

### OP1-1

#### Transcription Factor c-Maf is Critical to Direct Regulation of Glucose Transporters, Sglt2, and Attenuates Diabetic Kidney Condition and Injury Caused by Streptozotocin in Mice.

Mitsunori Fujino[1], Satoru Takahashi[1] ([1]*Department of Anatomy and Embryology, Faculty of Medicine, University of Tsukuba*)

The transcription factor c-Maf expresses in various tissues; in addition, it is known to be important for adult stage. However, due to the problem that c-Maf deficient mice in whole body are embryonic lethal, detailed function of *c-Maf* in adult has not been elucidated. To solve the problem, *c-Maf* conditional knockout mice were generated; analysis of *c-Maf* cko mice by tamoxifen showed high urine glucose levels without diabetic condition. The subsequent analysis suggested that c-Maf regulates *Sglt2*, which is a transporter and expresses in the proximal tubules of kidney and reabsorbs filtered glucose; therefore, the glycosuria is caused by reduction of *Sglt2* by *c-Maf* deletion. Moreover, *c-Maf* cko mice treated with streptozotocin improved not only blood glucose level but also diabetic kidney injury. In fact, it has reported that treatment of SGLT2 inhibitor in diabetic patients attenuates diabetic states and kidney injury; however, *Sglt2* knockout mice did not show improvement of kidney injury. Therefore, in the present research, I hypothesize that loss of *c-Maf* improves the progression of diabetic states and renal damage through not only regulation of *Sglt2* but also another regulation. (COI:No)

### OP1-2

#### Downstream projection of Barrington's nucleus to the spinal cord in mice

Masahiro Kawatani[1], William Chet de Groat[4], Keiichi Itoi[5], Katsuya Uchida[5], Kenji Sakimura[6], Akihiro Yamanaka[2], Takayuki Yamashita[2,3], Masahito Kawatani[1] ([1]*Dept of Neurophysiology, Grad School of Medicine, University of Akita* , [2]*Dept of Neuroscience Ⅱ, RIEM, University of Nagoya* , [3]*Dept of Physiology, School of Medicine, Fujita Health University,* [4]*Dept of Pharmacology and Chemical Biology, University of Pittsburgh,* [5]*Dept of Neuroendocrinology, Graduate School of Medicine, University of Tohoku,* [6]*Dept of Animal Model Development, Brain Research Institute, University of NIigata*)

Barrington's nucleus controls micturition behavior through the downstream projection to the spinal cord. In the Barrington's nucleus, two types of projection neurons, which were classified by the expression of *Crh* ($Bar^{CRH}$) and *Esr1* ($Bar^{ESR1}$), have suggested playing distinct roles on micturition behavior by manipulations of neural activity. The difference of their functional roles was explained by their projection targets, i.e, $Bar^{CRH}$ preferentially projected to the L6-S1 intermediolateral (IML) region in the spinal cord, and $Bar^{ESR1}$ projected to L6-S1 IML and dorsal commissure nucleus (DCN) region in the spinal cord. To understand the detailed mechanisms of synaptic integration in the spinal cord, we conducted *in vitro* slice patch-clamp recordings from spinal neurons, which are involved in the regulation of pelvic organ function. We examined the synaptic connectivity from Bar populations by using ChR2 assisted circuit mapping (CRACM) techniques. As a result, we provide the principles of synaptic connectivity from Bar to the spinal cord and suggest one part of the mechanisms of synaptic integration in the spinal cord. (COI:No)

### OP1-3

#### Inhibition of PDGFRα signal decreased aggressiveness of androgen-independent prostate cancer cell

Nayeem Md Junayed[1], Aya Yamamura[1], Rie Takahashi[1], Hisaki Hayashi[1], Hiroyuki Muramatsu[2], Kogenta Nakamura[2], Naoto Sassa[2], Motohiko Sato[1] ([1]*Dept. Physiol., Aichi Medical Univ.,* [2]*Dept. Urology., Aichi Medical Univ.*)

Androgen receptors (ARs) play important role in prostate cancer (PCa) progression, however growth of a metastatic prostate cancer cell line, PC-3 is independent of ARs. The platelet-derived growth factors receptors (PDGFRs) exhibit multiple functions in cancer progression, whereas a role of PDGFRs in PCa is not well established yet. In this study, we examined a role of PDGFRs in PC-3 cells. We also evaluated the effect of tyrosine kinase inhibitor, imatinib on viability, proliferation, migration and PDGFR signaling. PC-3 cells expressed higher level of PDGFRα than normal prostate epithelial cells. PDGFBB, ligand for PDGFR signaling induced phosphorylation of PDGFRα and Akt in a dose dependent manner. Knock down of PDGFRs by siRNA effectively reduced viability and migration of PC-3 cells. Imatinib, significantly inhibited viability, proliferation, migration and phosphorylation of PDGFRα/Akt in PC-3 cells. These observations suggest that PDGFRα signal is important for the growth of androgen-independent PC-3 cells. An inhibition of PDGFRα may be a novel therapeutic target to control hormone- refractory prostate cancer. (COI:No)

### OP1-4

#### Anatomical study of striated and smooth muscles surrounding the Cowper's gland

Satoru Muro[1], Keiichi Akita[1] ([1]*TMDU·ClinAnat*)

The Cowper's gland (CG) is responsible for lubricating urethral lumen and neutralizing acidic urine. Although it is generally believed that smooth muscle is involved in glandular secretion, a recent physiological study using rats reported that the striated muscle surrounding the Cowper's gland was involved in its secretion. The present anatomical study aimed to clarify the structure of muscle around the CG in humans, in order to contribute to elucidate the secretory mechanism. Six pelvises of male cadavers were used for macroscopic and immunohistological examination. CG was observed dorsal to the membranous part of the urethra. The superficial transverse perineal muscle was located dorsolateral to CG and extended its muscle bundle around CG. On the cranial side of CG, a plate-like structure with transverse fibers was observed. Imminohistological analysis revealed that CG was surrounded laterally by striated muscle and cranio-dorso-medially by smooth muscle. The striated muscle extended from the dorsolateral side of CG to ventral side of the urethra. These findings suggest that the secretion of CG in human is carried out by the cooperation of striated muscle and smooth muscle. (COI:No)

### OP1-5

#### Topographical analysis of inter-chromosomal interaction between *Pgk-1* and *-2* in mouse male germ cells by in situ PCR

Yasuaki Shibata[1], Takehiko Koji[1] ([1]*Department of Histology and Cell Biology, Nagasaki University Graduate School of Biomedical Sciences*)

Phosphoglycerate kinase (PGK) is an indispensable enzyme for glycolysis. In mouse testes, X-linked *Pgk-1* expressed in spermatogonia is ceased in spermatocytes due to meiotic sex chromosome inactivation, whereas chromosome 17-linked *Pgk-2* exclusively expressed in spermatocytes and spermatids. We have hypothesized that the mechanisms involve any inter-chromosomal interaction of both gene and here we tried to establish the double in situ PCR (ISPCR) for detection of both *Pgk* loci. ICR mouse (8W) testes were fixed with 4% paraformaldehyde/PBS, embedded in paraffin and sectioned. Digoxigenin- or biotin-dUTP were incorporated by ISPCR with mouse *Pgk-1*, *-2* or *18S rDNA* primers and detected by immunofluorescent histochemistly. The signals were analyzed with super-resolution microscope. As a result, the signals of *18S rDNA* were detected in the nucleoli of Sertoli cells and spermatogenic cells. Double ISPCR using *Pgks* primer sets and their upstream primers sets (5000 base pairs to 5' direction) successfully revealed co-localization of each signal. Double ISPCR is quite useful for the detection of two gene loci. (COI:No)

### OP1-6

#### Rebuilding Wolffian duct and mesonephric tubules during the development of mouse efferent tubules

Takuya Omotehara[1], Hiroki Nakata[2], Masahiro Itoh[1] ([1]*Department of Anatomy, Tokyo Medical University,* [2]*Faculty of Medicine, Kanazawa University*)

**Introduction:** Spermatozoa are transported to the epididymal duct through two-five efferent tubules in a mouse. Although the origin of the efferent tubules is thought to be mesonephric tubules, their developmental process, i.e. where the rete testis and efferent tubules are connected, is unclear.
**Materials and Methods:** Serial paraffin sections of the developing mouse gonad-mesonephros complex were cut, and some marker proteins were detected by sequential immunohistochemistry. Three-dimensional reconstruction was performed using Amira software.
**Results:** Four-six mesonephric tubules were connected with the Wolffian duct, and rete cells contacted with the three-five mesonephric tubules at their tip. The length of the tubules became shorter at first but longer in later development. Tortuosity, index of twisting, also got smaller as the development proceeded but bigger in the later development.
**Discussion:** The connection between the rete testis and efferent tubules is possibly induced at the tip of the mesonephric tubules. The mesonephric tubules are "S shape" at first like nephric tubules in the kidney and then rebuilt as a similar structure to the adult efferent tubules by birth. (COI:No)

### Oral Presentation2

### Muscle

(March 28, Sun. 9：00～10：12, Room10)

### OP2-1

**Switching of Sox9 expression during musculoskeletal system development.**

Masahito Yamamoto[1], Tetsu Naito[1], Takahiro Takagi[1], Chiemi Kanehira[1], Satoru Mastunaga[1], Kei Kitamura[2], Hitoshi Yamamoto[2], Shinichi Abe[1] (*[1]Anat., Tokyo Dent. Coll, [2]Hist., and Embryo, Tokyo Dent. Coll*)

The musculoskeletal system, which comprises muscles, tendons, and bones, is an efficient tissue complex. The process of its construction into a single functional and complex organization is unclear. SRY-box containing gene 9 (Sox9) is expressed initially in pluripotent cells. This study investigated how Sox9 controls the development of each component of the musculoskeletal system. Sox9 was expressed in MTJ, tendon, and bone progenitor cells at E13 and in bone at E16. We detected Sox9 expression in muscle progenitor cells. However, we found no Sox9 expression in developed muscle. A decrease in Sox9 expression in muscle-associated connective tissues, tendons, and bones led to hypoplasia of the cartilage and its attachment to tendons and muscle. These results showed that switching on Sox9 expression in each component (muscle, tendon, and bone) is essential for the development of the musculoskeletal system. Sox9 is expressed in not only tendon and bone progenitor cells but also muscle progenitor cells, and it controls musculoskeletal system development. (COI:No)

### OP2-2

**Paxillin is a novel signaling molecule which regulates abnormal vascular contraction mediated by an SPC/Fyn/Rho-kinase pathway**

Ying Zhang[1], Tomoka Morita[1], Hakuchou Ro[1], Dan Cui[2], Hiroko Kishi[1], Min Zhang[1], Sen Ro[1], Nan Li[1], Eiji Ikeda[2], Sei Kobayashi[1] (*[1]Dept Mol Cell Physiol, Grad Sch Med, Yamaguchi Univ, Ube, Japan, [2]Dept Pathol, Grad Sch Med, Yamaguchi Univ, Ube, Japan*)

Rho-kinase (ROK)-induced abnormal contraction of vascular smooth muscle (VSM) is a major cause of cardiovascular and cerebrovascular vasospasm. We previously demonstrated that sphingosylphosphorylcholine (SPC) induced this abnormal contraction via an SPC/Fyn/ROK pathway. The combination of pulldown assay and MALDI-TOF mass spectrometry made us identify paxillin as the possible downstream molecule of Fyn. Until now, no direct evidence is provided to prove paxillin involved in the SPC-induced contraction. In the present study, we examined the role of paxillin in the SPC-induced abnormal contraction of VSM. First, paxillin knockdown inhibited the SPC-induced contraction in human coronary artery smooth muscle cells. Then we used Cre-loxP system to generate tamoxifen-inducible and smooth muscle-specific paxillin knockout mouse and showed that targeted deletion of paxillin in VSM inhibited the SPC-induced abnormal contraction of VSM. Paxillin knockout also inhibited the SPC-induced ROK activation and myosin light chain phosphorylation. These results indicate that paxillin plays an important role in the SPC-induced abnormal contraction of VSM. (COI:No)

### OP2-3

**Reactive oxygen species induce $Mg^{2+}$-extrusion from $Mg^{2+}$-loaded rat ventricular myocytes**

Michiko Tashiro[1], Masato Konishi[1], Utako Yokoyama[1] (*[1]Dept Physiol, Tokyo Med Univ*)

To extend our previous study which $H_2O_2$ reduced the basal level of intracellular free $Mg^{2+}$ concentration ($[Mg^{2+}]_i$) of rat ventricular myocytes, we studied whether reactive oxygen species (ROS) induce $Mg^{2+}$- extrusion from $Mg^{2+}$-loaded cells by measurements of $[Mg^{2+}]_i$ with a fluorescent indicator, furaptra. For $Mg^{2+}$ loading, the cells were soaked in low-$Na^+$ and high-$Mg^{2+}$ solution for 2 h to raise $[Mg^{2+}]_i$ (from 1.14 ± 0.028 to 1.72 ± 0.057 mM), then extracellular free $Mg^{2+}$ concentration ($[Mg^{2+}]_o$) was set at 1 mM. Five minutes application of 500 μM $H_2O_2$ (at 1 mM $[Mg^{2+}]_o$ in the absence of extracellular $Na^+$) rapidly decreased $[Mg^{2+}]_i$ to 0.64 ± 0.15 mM within 15 min and this lower $[Mg^{2+}]_i$ level was maintained thereafter up to 90 min. The rate of decrease in $[Mg^{2+}]_i$ during the initial 9 min was 2.12 ± 0.25 μM/s, which was significantly higher than that obtained in the presence of extracellular $Na^+$. The $Na^+$-independent $Mg^{2+}$ extrusion was also induced by 100 μM pyocyanin, a ROS generator. The effect of pyocyanin was inhibited by pre-treatment of *N*-acetyl-l-cysteine, a ROS scavenger. These results suggest that ROS disrupt $Mg^{2+}$ homeostasis in the heart via a decrease in $[Mg^{2+}]_i$. (COI:No)

### OP2-4

**The role of calpain activation and vimentin cleavage in $Ca^{2+}$ -sensitization of vascular smooth muscle contraction**

Hiroko Kishi[1], Sen Ro[1], Tomoka Morita[1], Ying Zhang[1], Hakuchou Ro[1], Min Zhang[1], Nan Li[1], Minhui Xu[1], Sei Kobayashi[1] (*[1]Department of Molecular and Cellular Physiology, Yamaguchi University Graduate School of Medicine*)

$Ca^{2+}$-sensitization of vascular smooth muscle (VSM) plays a critical role in abnormal VSM contractions such as vasospasm. Previously we identified sphingosylphosphorylcholine (SPC)/Fyn/ROK pathway which mediates $Ca^{2+}$-sensitization of VSM contraction. As possible downstream targets of Fyn tyrosine kinase, we identified vimentin by focused proteomics. Interestingly, SPC induced limited proteolysis of vimentin in human coronary artery smooth muscle cells (CASMCs) and VSM strips of the porcine coronary artery (PCA). Since vimentin is reported as the target of calpain, we examined the involvement of calpain. In CASMCs, SPC increased calpain activity, which was blocked by PD150606, a calpain inhibitor. Furthermore, PD150606 inhibited the SPC- and U46619-induced VSM contractions but not high $K^+$ depolarization-induced $Ca^{2+}$-dependent contraction in PCA. PD150606 also inhibited the SPC-induced VSM contraction in mouse basilar artery. Mass spectrometric analysis showed that calpain cleaved vimentin at its N-terminus. These findings suggest the possible involvement of calpain in the signal transduction of $Ca^{2+}$ -sensitization of VSM contraction induced by SPC and U46619. (COI:No)

### OP2-5

**Relaxation process in beta escin skinned taenia cecum in the presence of nucleotides other than ATP**

Masaru Watanabe[1], Sachiko Otsuka[1] (*[1]Laboratory of Physiology, Graduate School of Human Health Sciences, Tokyo Metropolitan University, Japan*)

Slow relaxation process in skinned smooth muscle is known to reflect "latch bridge" formation, and we have hypothesized that a number of detached fast cross-bridges reattached forming "latch like" slow cycling-bridge then slowly detached during the relaxation process (Mihashi et al.., 2020). To characterize the fast cross-bridge detachment and "latch like bridge" cycling during relaxation in detail, the effects of nucleotides other than adenosine ATP on the relaxation process were kinetically analyzed using beta escin skinned taenia cecum, since these nucleotides are poor substrates for myosin light chain (MLC) kinase, but activate cross-bridge cycling under irreversible MLC phosphorylation. Compared with ATP, CTP, ITP and UTP significantly slowed the relaxation process after removal of Ca ion from the maximal Ca ion activated preparations both in the presence or absence of ML-7, an inhibitor of MLC kinase. Regression analysis of the relaxation process suggests that the slowing effect of CTP, ITP and UTP on the relaxation was mainly attributed to increase in the number of the slow cycling-bridge and to reduce detachment rate of the slow cycling-bridge. (COI:No)

### OP2-6

**The inhibitory effect and mechanism exploration of compound H on sphingosylphosphorylcholine(SPC)-induced contraction in vascular smooth muscle**

Sen Ro[1], Hakuchou Ro[1], Ying Zhang[1], Tomoka Morita[1], Hiroko Kishi[1], Min Zhang[1], Nan Li[1], Minhui Xu[1], Sei Kobayashi[1] (*[1]Department of Molecular and Cellular Physiology, Yamaguchi University Graduate School of Medicine, Ube, Japan*)

Cardiocerebrovascular diseases have the highest mortality and disability rates all over the world. We previously found that SPC is the key molecule leading to vasospasm. To date, we have identified the SPC/Fyn/Rho-kinase (ROK) pathway as a novel signaling pathway for $Ca^{2+}$-sensitization of vascular smooth muscle (VSM) contraction. We aimed to investigate whether compound H could inhibit the SPC-induced contraction with little effect on 40 mM $K^+$-induced $Ca^{2+}$ -dependent contraction and to elucidate the underlying mechanisms of it. Compound H (30 μM) significantly inhibited the SPC-induced contraction of porcine coronary artery smooth muscle strips with little effect on 40 mM $K^+$-induced contraction. Compound H blocked the SPC-induced translocations of Fyn and ROK from the cytosol to the membrane of human coronary artery smooth muscle cells (HCASMCs). SPC decreased phosphorylation level of Fyn at Tyr527 in HCASMCs and increased phosphorylation level of myosin phosphatase target subunit 1(MYPT-1) at Thr 850 and myosin light chain (MLC) in both VSM and HCASMCs, which were significantly reduced by compound H. (COI:No)

### Oral Presentation3

### Stress

(March 28, Sun. 10：17～10：53, Room9)

### OP3-1

**A psychosomatic pathway from the prefrontal cortex to the hypothalamus that drives physiological responses to psychological stress**

Kataoka Naoya[1], Yuta Shima[1], Kazuhiro Nakamura[1] ([1]*Dep Integrative Physiol, Grad Sch Med, Nagoya Univ Japan,* [2]*Nagoya Univ Institute for Adv Res.*)

The brain network that evokes autonomic and behavioral responses for stress coping has been a major focus of physiological research. We have previously reported that psychological stress induces thermogenesis in brown adipose tissue, hyperthermia and tachycardia by activating a monosynaptic neural pathway from the dorsomedial hypothalamus (DMH) to the rostral medullary raphe. Recently, we discovered that the DMH receives glutamatergic excitatory stress inputs from the dorsal peduncular cortex and dorsal tenia tecta (DP/DTT), located at the ventral limit of the medial prefrontal cortex. Here, it was determined how important the DP/DTT–DMH neural pathway is for the drive of physiological stress responses. Optogenetic inhibition or selective lesions of DP/DTT–DMH projection neurons diminished thermogenic, skin vasoconstrictor, hyperthermic, tachycardic and pressor responses to social defeat stress. Optogenetic inhibition of the DP/DTT–DMH pathway also eliminated avoidance behavior from stressors. These results demonstrate that the DP/DTT–DMH pathway transmits a master psychosomatic signal that drives a variety of sympathetic and behavioral stress responses. (COI:No)

### OP3-2

**Neural projection from the midbrain to the medulla involved in the pressor response during social defeat stress in rats**

Mio Matsuyama[1], Ena Yamamoto[1], Joji Horiuchi[1] ([1]*Department of Biomedical Engineering, Toyo University, Japan*)

It has been shown that the sympathetic vasomotor pathway of psychological stress is mediated via neurons in the rostroventral medulla (RVM) indirectly from the hypothalamic stress center. In this study, direct projections to the RVM and distribution of neuroexcitatory marker, c-Fos expressed neurons were investigated during the social defeat stress (SDS) in conscious rats. To verify neural projections to the RVM, we injected a neural tracer, FluoroGold (FG) into the RVM. One week later, the FG injected rat was exposed to the SDS and then double-labeled neurons were observed in the midbrain. The double labeled (c-Fos and FG) neurons were locally distributed in the lateral/ventrolateral periaqueductal grey matter (l/vlPAG) in the midbrain. Therefore, we microinjected DL-Homocystic acid (DLH) to stimulate neurons in the l/vlPAG in anesthetized rats. The DLH injection into the l/vlPAG caused decent increases in blood pressure and renal sympathetic activity without obvious changing in heart rate. Taken together, these results suggested that neurons in the l/vlPAG participate in the pressor response during acute psychological stress, like the SDS, as a sympathetic vasomotor relay point. (COI:No)

### OP3-3

**Distribution of c-Fos expressed neurons and the cardiovascular reaction evoked by social defeat stress in the serotonin-deficientrat**

Yuka Ichinotsuka[1], Mio Matsuyama[1], Akari Hori[1], Joji Horiuchi[1] ([1]*Department of Biomedical Engineering, Toyo University*)

It is suggested that central serotonin plays an important role on the stress-induced cardiovascular response. In this study, to clarify the role of central serotonin in the cardiovascular response during acute psychological stress, central expression of a neural excitatory marker, c-Fos, induced by the stress was investigated by using serotonin-deficient model rats (FH/H rats). Social defeat stress (SDS) was used as acute psychological stress. The SDS challenge elicited pressor and tachycardic responses in FH/H rats, but both responses were suppressed or unchanged in magnitude and did not persist in reaction times compared to Wistar rats. The number of c-Fos neurons in the dorsomedial hypothalamic area significantly increased in FH/H rats. In contrast, the numbers of c-Fos neurons in the medullary raphe and in the rostroventral medulla, which are possible cardiovascular centers of the stress responses, was inhibited or unchanged. These results suggest that the hypothalamic serotonin suppressively participates in the SDS induced cardiovascular reaction and that serotonin in the rostral medulla plays the opposite action of that in the hypothalamus. (COI:No)

### Oral Presentation4

### Blood, Lymph, Immunity

(March 28, Sun. 10：17～11：05, Room10)

### OP4-1

**Long-term in vivo Imaging of labeled cells using near-infrared fluorescent organosilica nanoparticles**

Michihiro Nakamura[1], Junna Nakamura[1], Chihiro Mochizuki[1] ([1]*Department of Organ Anatomy & NANOMEDICINE Yamaguchi University Graduate School of Medicine*)

Fluorescence imaging using near infrared (NIR) region has become widely used recently. NIR fluorescence *in vivo* imaging can detect cancer cells *in vivo* since NIR light easily pass through the living body. We are developing a technique to create various multifunctional nanoparticles from organosilicate. In this study, we produced organosilica nanoparticles with NIR fluorescence. NIR fluorescent organosilica nanoparticles demonstrated high biocompatibility and safety even when intravenously administered into mouse. *In vivo* NIR fluorescence imaging enabled long-term observation of particle accumulating organs and cancer tissues. Furthermore, cells (macrophages) were *in situ* labeled by intravenously administration of the particles into mouse body, and the dynamics of labeled cells in the body could be observed. We conducted *in vivo* imaging of *in situ* labeled cells against subcutaneous xenograft cells. The *in vivo* imaging showed a migration and an accumulation of the *in situ* labeled cells to the site of xenograft cells. NIR fluorescence *in vivo* imaging using NIR fluorescent organosilica nanoparticles is useful for long-term observation.

(COI:No)

### OP4-2

**Hematopoietic tissue is one of the organs that suffer lifethreatening damage in hemophagocytic lymphohistiocytosis**

Harada Tomonori[1], Isao Tsuboi[1], Hirotsugu Hino[1], Michiko Naito[1], Hiroyuki Hara[1], Shin Aizawa[1] ([1]*Anat. Sci. Sch. Med. Nihon Univ.*)

Hemophagocytic lymphohistiocytosis (HLH) is a life-threatening systemic hyperinflammatory disorder. Repeated LPS treatment induces HLH-like features in senescence-accelerated mice (SAMP1/TA-1) but not in senescence-resistant control mice (SAMR1). We analyzed the impairment of hematopoiesis in this mouse model. The numbers of myeloid progenitor cells (CFU-GM) and B-lymphoid progenitor cells (CFU-preB) in the bone marrow (BM) rapidly decreased after each LPS treatment in both strains. The number of CFU-GM in SAMP1/TA-1 and SAMR1, and of CFU-preB in SAMR1, returned to pretreatment levels after each treatment. However, the recovery in the number of CFU-preB in SAMP1/TA-1 was limited. Gene expression of B lymphopoietic positive-regulators was persistently decreased in SAMP1/TA-1 but not in SAMR1. Gene expression of p16 and the proportion of $\beta$-galactosidase-positive cells were increased in cultured stromal cells obtained from LPS-treated SAMP1/TA-1 but not in those from LPS-treated SAMR1. LPS treatment induced qualitative changes in stromal cells, which comprise the microenvironment supporting appropriate hematopoiesis, in SAMP1/TA-1. (COI:No)

### OP4-3

**The Function of Histamine in Hepatic Hematopoiesis in Postnatal Mice.**

Hirotawda Otsuka[1], Harumi Kasuya[1], Yasuo Endo[3], Hiroshi Ohtsu[4,5], Masanori Nakamura[2], Satoshi Soeta[1] ([1]*Laboratory of Veterinary Anatomy, NVLU,* [2]*Department of Oral Anatomy and Developmental Biology, Showa University,* [3]*Graduate School of Dentistry, Tohoku University,* [4]*Tekiju Rehabilitation Hospital,* [5]*Tohoku University*)

Histidine decarboxylase (HDC), histamine synthase, is expressed in hematopoietic stem cells. However, the role of histamine in hematopoiesis is not well described. To evaluate the role of histamine in hematopoiesis, we analyzed the morphological analyses of the hematopoietic sites using wild-type and HDC-deficient (HDC-KO) mice. Histological analysis showed no significant change in the adult bone marrow and spleen of HDC-KO mice compared to wild-type mice. Morphological analysis of the liver revealed more numerous hematopoietic colonies and megakaryocytes in HDC-KO mice compared to wild-type mice at 2 and 3 weeks of age, whereas no changes were observed in adults. Most of these hematopoietic colonies consisted of B-lymphocytes and erythroblasts and were positive for the cell proliferation marker PCNA. Notably, these hematopoietic colonies declined in HDC-KO mice upon N-acetyl histamine treatment. A significant increase in the expression of hematopoiesis-related cytokines was observed in the liver of 3-week old HDC-KO mice compared to wild-type mice. These results suggest that histamine-deficiency may maintain an environment suitable for hematopoiesis in the liver of postnatal mice. (COI:No)

### OP4-4

**Regulatory role of thymic epithelial primary cilia on the T cell development**

Osamu Kutomi[1], Shigenori Nonaka[2], Sen Takeda[1] ([1]*Grad. Sch. Med., Univ., Japan,* [2]*NIBB, Japan*)

Primary cilia are ubiquitous hair-like organelle protruding from the cells, and essential for the organogenesis and homeostasis of various physiological functions. However, few reports have reported the roles of primary cilia in immune systems. Thymus is a primary lymphoid tissue that nurtures immature T cells to be a full-fledged state, where a meshwork of thymic epithelial cells (TECs) build the thymic stroma. While we have discovered the primary cilia in TEC, their precise roles in thymic organogenesis and T cell development remain unclear. To clarify this, we established a line of conditional knockout mice (cKO mice) lacking primary cilia specifically in TECs. The cKO mice showed slightly increased development of thymic regulatory T cell (Treg) that suppresses immune responses in the peripheral tissue. Furthermore, we found a cellular interface between T cells and TECs in medulla, and named it "thymic synapse". Considering the fact that thymic medulla serves as a site for differentiation of Treg and acquisition of self-tolerance in T cells, these results suggest that primary cilia of TECs play a crucial role in the maturation of T cells in thymic medulla. (COI:No)

### Oral Presentation5

### Neural development, etc.

(March 28, Sun. 14：20～15：20, Room10)

### OP5-1

**Developmental system drift in the spinal cord development**

Katsuki Mukaigasa[1], Chie Sakuma[1], Hiroyuki Yaginuma[1] ([1]*Fukushima Med. Univ.*)

In the first step of the neural tube development, Shh is induced in the notochord and floor plate, leading to the graded Shh signaling activity from ventral to dorsal. The strength of the Shh signal is translated into the transcriptional activity of the Gli transcription factors. As the result of gene regulatory network (GRN), neuronal progenitor domains are established along the dorsoventral axis of the neural tube, which is largely conserved among vertebrate. However, previous studies have demonstrated that *Gli* mutant phenotypes in the neural tube are different between mice and zebrafish, suggesting that the action of Shh signaling have diverged during vertebrate evolution. Here, we found that several *cis*-regulatory modules (CRMs) located in the neural tube patterning genes are conserved in tetrapod, but not in teleost. These CRMs harbor multiple transcription factor binding sites. Despite being lost in the teleost lineage, reporter assay confirmed that these CRMs are functional in the chick neural tube. Thus, we propose that GRN preceding the establishment of the neural tube has been rewired during vertebrate evolution with the progenitor domain organization being conserved. (COI:No)

### OP5-2

**Single- cell transcriptomic analysis of the conditional Vesicular GABA Transporter deficient mouse**

Shigeyuki Esumi[1], Kento Morooka[2], Yuchio Yanagawa[3], Kenji Sakimura[4], Tatsunori Seki[5], Takaichi Fukuda[1] ([1]*Grad.Sch.Med.,Kumamoto-Univ,* [2]*Kumamoto-Univ Hospital,* [3]*Gunnma-Univ,* [4]*Niigata-Univ,* [5]*Tokyo Medical-Univ*)

Dysfunction of cortical GABAergic neuron is associated with neurological and psychiatric in human and rodent model. Recently, it has been reported that GABA has multiple function during development for example synaptogenesis, migration and control of proliferation. However, effect of GABA during cortical GABAergic neuron development are not yet fully understood. Here, to elucidate function of GABA during cortical development, we performed a droplet based single-cell RNA-seq analysis using Nkx2-1-cre; VGAT cKO homo/ hetero mouse at postnatal day 0. We obtained more than 10,000 individual cell expression data. Further, we separated neuronal and non-neuronal clusters based on their expression profile and various markers. As results of comparing homo/hetero expression profiling data, the distributions of GABAergic and glutamatergic neuron progenitor clusters were shifted, respectively. Our results suggest that GABA has essential role for the development of GABAergic neurons and glutamatergic neurons. (COI:No)

### OP5-3

**An activity-dependent local transport regulation via degradation and synthesis of KIF17 underlying cognitive flexibility**

Suguru Iwata[1], Momo Morikawa[2], Yosuke Takei[3], Nobutaka Hirokawa[1] ([1]*Department of Cell Biology and Anatomy, Graduate School of Medicine, The University of Tokyo,* [2]*Laboratory for Molecular Psychiatry, RIKEN Center for Brain Science,* [3]*Department of Anatomy and Neuroscience, Faculty of Medicine, University of Tsukuba*)

Synaptic weight changes among postsynaptic densities within a single dendrite are regulated by the balance between localized protein degradation and synthesis. However, the molecular mechanism via these opposing regulatory processes is still elusive. Here, we showed that the molecular motor KIF17 was locally degraded and synthesized in an *N*-methyl-D-aspartate receptor (NMDAR)-mediated activity-dependent manner. Accompanied by the degradation of KIF17, its transport was temporarily dampened in dendrites. We also observed that activity-dependent local KIF17 synthesis driven by its 3' untranslated region (3'UTR) occurred at dendritic shafts, and the newly synthesized KIF17 moved along the dendrites. Furthermore, hippocampus-specific deletion of *Kif17* 3'UTR disrupted KIF17 synthesis induced by fear memory retrieval, leading to impairment in extinction of fear memory. These results indicate that the regulation of the KIF17 transport is driven by the single dendrite-restricted cycle of degradation and synthesis that underlies cognitive flexibility. (COI:Properly Declared)

### OP5-4

**Anti Alzheimer gene BRI2 binds MHC Class I**

Shuji Matsuda[1], Takao Senda[1] ([1]*Gifu Univ Med School*)

lzheimer Disease (AD) is characterized by large neuronal loss, neurofibrillary tangles, and beta amyloid deposits. Beta amyloid is cleaved from its precursor, Amyloid Precursor Protein (APP) through successive proteolysis. Since (1) genetic mutations of APP causes familial AD, (2) all known causative genes of AD are involved in metabolism of APP, the processing of APP is the integral component of AD.We have identified type II transmembrane BRI2 binds APP and inhibit all three metabolic pathways of APP. In fact mice over expressing BRI2 suppresses the amyloid deposits of AD model mice. Concurrently, BRI2 knockout mice show memory deficits.In this study, we searched for endogenous proteins that binds BRI2, and found MHC Class I. MHC Class I is expressed in nearly all human cells, with three alleles HLAA, HLAB, and HLAC. These alleles complex with beta 2 microglobulin, and used for antigen representation. At the same time, HLAA, B, C are known to be present at brain synapses. BRI2 binds all HLAA, B, C and the region necessary for the binding is shared in these alleles. This time we discuss how MHC Class I influence the metabolic regulation of APP through BRI2. (COI:No)

### OP5-5

**Distribution of mRNAs for Na,K-ATPase α and β subunit isoforms in the mouse brain**

Koshi Murata[1], Tomoki Kinoshita[1], Tatsuya Ishikawa[1,3], Kazuki Kuroda[1,2], Minako Hoshi[4], Yugo Fukazawa[1,2,5] ([1]*Div. Brain Structure Function, Univ. Fukui, Fukui, Japan.,* [2]*Life Science Innovation Center, Univ. Fukui, Fukui, Japan.,* [3]*Dept. Functional Anatomy, Kanazawa Univ., Ishikawa, Japan,* [4]*Dept. Brain Neurodegenerative Disease Research, Foundation for Biomedical Research and Innovation at Kobe, Kobe, Japan.,* [5]*Research Center for Child Mental Development, Univ. Fukui, Fukui, Japan.*)

Na,K-ATPase contributes to the asymmetric distribution of Na and K ions and the membrane potential maintenance. Na,K-ATPase is composed of three subunits. In mice, the α subunit has four isoforms, three of which are expressed in the brain. However, the functional and biological significance of the isoforms has not yet been fully elucidated. Recent studies have revealed that Atp1a3, a gene encoding the α3 subunit, is associated with various neurological diseases. In this study, we evaluated the mRNA expression of Atp1a1, Atp1a2, and Atp1a3 in mouse brain by in situ hybridization to identify brain regions and cell types that express the α subunit isoforms. Atp1a2 was expressed in glial cells. Most of the neurons coexpressed Atp1a1 and Atp1a3, and the expression level varied greatly by brain region and neuron type. Parvalbumin neurons in the hippocampus expressed little Atp1a1 and high Atp1a3, as well as high Atp1b1 and little Atp1b2/ Atp1b3. Our data showed that the expression levels of Na,K-ATPase α and β subunit isoforms vary among brain regions and neuronal subtypes, and suggest that the identified neuron types with high Atp1a3 may be involved in various neurological diseases. (COI:No)

### Oral Presentation6

### Neuronal projection, Neural network

(March 28, Sun. 15：25～16：25, Room10)

### OP6-1

**Stress-Related Neuronal Clusters in Sublenticular Extended Amygdala of Basal Forebrain Show Individual Differences of Positions**

Hiroyuki Ichijo[1], Munenori Kanemoto[3], Masakiyo Sasahara[2], Tomoya Nakamura[1] ([1]*Univ Toyama, Dept AnatNeuorsci,* [2]*Univ Toyama, Dept Pathol,* [3]*National Center for Geriatrics and Gerontology*)

To understand neuronal circuits for emotion, neuronal activation was examined in mice by expression of Zif268/Egr1 and c-Fos. In all mice, neuronal clusters expressing Zif268 were found in the sublenticular extended amygdala, consisting of mainly GABAergic neurons. Expressions of neuronal markers indicated that these were novel neuronal clusters; thus, we named them the sublenticular extended amygdalar Zif268-expressing neuronal clusters (SLEA-zNCs). SLEA-zNC participated in stress processing because increasing numbers of cells were observed after exposure to restraint stress (RS), the induction of which was suppressed by diazepam treatment. Mapping SLEA-zNCs showed that their positions varied individually. Because time courses of activation differed between the Zif268 and c-Fos, the sequential dual treatment of RSs enabled us to differentiate SLEA-zNCs activated by the first and second RS. The results supported that the same SLEA-zNCs responded to both the first and second RS, and this also applied for all SLEA-zNCs; their positions were invariable in each mouse but were distributed differently between individual mice. SLEA-zNCs showed individual differences in their positions. (COI:Properly Declared)

### OP6-2

**Functional connectivity of neural circuitry necessary for pain behavior elucidated using a holographic system**

Daisuke kato[1], Takuya Okada[2], Hiroaki Wake[1] ([1]*Nagoya University Graduate School of Medicine Department of Anatomy and Molecular Cell Biology,* [2]*Kobe University Graduate School of Medicine Division of Anesthesiology*)

Digital holography molds the laser to reconstruct a 3D image of the object. Recently, we have developed a new microscope for biological application of optogenetics with a two-photon laser using the digital holographic technique. This microscope can stimulate cells in various patterns with higher spatiotemporal resolution, exceeding limits of previous optogenetic system. Here, using this microscope, we first found that increases in synchronized activities within S1 neurons during pain formation. Artificially increasing in neural activities and synchrony using chemogenetics (DREADD) reduced pain thresholds due to high functional connectivity, representing new neural circuitry associated with pain induction. Conversely, decreasing these neural activities with DREADD increased pain thresholds. In addition, increases in expression of N-type voltage dependent $Ca^{2+}$ channel subunits in S1 was seen, and local application of a selective this channel blocker reduced both neural activities and the allodynia. These findings indicated that neural circuitry in S1 play a crucial role in sustained pain, and modulation of their activities may provide a new therapeutic target for neuropathic pain. (COI:No)

### OP6-3

**Projection-identified large-scale recording reveals pathway-specific information outflow from the subiculum**

Takuma Kitanishi[1], Ryoko Umaba[3], Kenji Mizuseki[1] ([1]*Dept Physiol, Osaka City Univ Grad Sch Med,* [2]*PRESTO, JST,* [3]*Dept Neurosurg, Osaka City Univ Grad Sch Med*)

The hippocampus processes multimodal information associated with spatial navigation, including place, trajectory, and speed. However, how such information is distributed to multiple downstream areas remains poorly understood. We investigated this issue by identifying axonal projections using multisite optogenetics during large-scale extracellular recordings from the rat subiculum, the major hippocampal output structure. Subicular neurons demonstrated a noise-resistant representation of place, speed, and trajectory, which was as accurate as or even more accurate than that of hippocampal CA1 neurons. Speed and trajectory information was most prominently sent to the retrosplenial cortex and nucleus accumbens, respectively. Place information was distributed uniformly to the retrosplenial cortex, nucleus accumbens, anteroventral thalamus, and medial mammillary body. Information transmission by projection neurons was tightly controlled by theta oscillations and sharp-wave/ripples in a target region-specific manner. In conclusion, the dorsal subiculum robustly routes diverse navigation-associated information to downstream areas. (COI:No)

### OP6-4

**Multi-regional large-scale electrophysiology revealed that inter-regional coactivations of cell ensembles support fear memory**

Hiroyuki Miyawaki[1], Kenji Mizuseki[1] ([1]*Department of Physiology, Osaka City University Graduate School of Medicine*)

Recent studies indicate that memories are represented as combination of active neurons, known as cell ensembles. Although fear memory related ensemble activities have been reported in various brain regions, it is still elusive how inter-regional interactions of cell ensembles evolves through memory processes. To clarify this point, we performed large-scale electrophysiological recording in the basolateral amygdala (BLA), ventral hippocampus CA1 region (vCA1), and prelimbic region of medial prefrontal cortex (PrL) of fear conditioned rats. We revealed that coactivations of BLA-PrL ensemble pairs emerged during fear conditioning, whereas those of vCA1-PrL developed in the subsequent sleep periods. These coactivations were hosted by fast (100-200 Hz) network oscillations and reappeared during memory retrieval. Furthermore, in BLA and vCA1, but not in PrL, cells contributing to inter-regional coactivations were more active than other cells even in sleep prior to fear conditioning. These findings suggest that elements of memories captured immediately by pre-configured local networks and *de novo* inter-regional network develops to bind distributed information together. (COI:No)

### OP6-5

**Neural pathway contributing to suppression of conditioned taste aversion by gravity disturbance.**

Takenori Miyamoto[1], Mari Takeda[1], Eri Sasakawa[1], Midori Yamguchi[1], Maki Tanaka[1], Sae Fujimoto[1], Ritsuko Nomiyama[1], Aiko Watanabe[1], Hiroko Fujiwara[2], Ryohei Sato[3] ([1]*Lab Behav Neurosci, Fat Sci, Japan Women's Univ,* [2]*Univ Human Art Sci, Grad Sci,* [3]*Dept Physiol Sch Med Kitasato Univ*)

The conditioned taste aversion (CTA) paradigm was employed in order to examine the effect of modification of gravitational force on acquisition and retention of emotional learning and memory. We used 3D clinostat for C57BL/6 male mice to cancel the gravitational force. The gravity disturbance (GD) was applied during 20 min between 0.1% sodium saccharin as a conditioned stimulus and an i.p. injection of 0.15 M LiCl as an unconditioned stimulus in conditioning period. Under GD, both acquisition and retention were suppressed in intact mice, but not in mice with lesions of somatosensory area for the limbs (S1FL/HL). The expression of a neuronal activity marker c-Fos significantly decreased both in S1FL/HL and amygdala (AMY) of mice with GD. Some SIFL/HL neurons have been reported to project to the ventral posterolateral nucleus of thalamus (VPL). In the preliminary experiments, the number of c-Fos immunopositive S1FL/HL cells double-stained by the retrograde tracing with BDA 3000 injected into VPL significantly decreased after GD compared with before GD. These results suggest that the sensory inactivation of the S1FL/HL by GD reduces the CTA-activation of AMY via at least VPL. (COI:No)

### Oral Presentation7

### Neurons, Synapses①

(March 28, Sun. 16：30～17：30, Room10)

### OP7-1

**Effect of voluntary exercise on functional recovery and histological change in the ipsilateral cortex after cerebral ischemia**

Natsumi Yamaguchi[1], Kae Fukumoto[1], Toshinori Sawano[1], Jin Nakatani[1], Daijiro Yanagisawa[2], Ikuo Tooyama[2], Hidekazu Tanaka[1] (*[1]Lab. Pharmacol., Grad. Sch. Life Sci., Ritsumeikan Univ., [2]Dept. Diag. and Therap. for Brain Dis., Mol. Neurosci. Res. Ctr., Shiga Univ. Med. Sci.*)

It has been reported that rehabilitation after cerebral ischemia affects neural plasticity and improves functional recovery. However, the mechanisms are not clear. We investigated the histological change in the ipsilateral cortex to reveal more detailed mechanisms of functional recovery. We adopted highly reproducible focal cerebral ischemia model using C.B-17/Icr-$^{+/+}$Jcl mice. Mice were divided into 4 groups: Sham, Sham + exercise, Middle cerebral artery occlusion (MCAO), MCAO + exercise. Mice in the exercise group were given free access to the running wheel for 24 hours. In grid walking test and wire hang test, functional recovery was improved in MCAO + exercise group on postoperative day 14. The volume of survived ipsilateral cortex and the number of mature neurons in the peri-infarct cortex were not affected by exercise. On the other hand, the ischemia-induced basal dendritic spine loss was suppressed by exercise. In this region, voluntary exercise also increased the number of c-Fos-positive cells. From these results, voluntary exercise after cerebral ischemia may maintain the basal dendritic spine density, which can be related to modified neuronal activity. (COI:No)

### OP7-2

**Carbonic anhydrase related protein Car8 is essential for the establishment of cerebellar neuronal circuit, regulating precise matching of pre and postsynapse in Purkinje cell**

Taisuke Miyazaki[1], Miwako Yamasaki[2], Kenji Sakimura[3], Masahiko Watanabe[2] (*[1]Dept. Health Sciences, Sch. Med. Hokkaido Univ.. Sapporo, Japan, [2]Dept. Anatomy, Grad. Sch. Med. Hokkaido Univ.. Sapporo, Japan, [3]Dept. Cell. Neurobiol., Brain Res., Inst.. Niigata Univ.. Niigata, Japan*)

A carbonic anhydrase related protein Car8 is exclusively expressed in cerebellar Purkinje cells (PC). Previous reports showed that mutation of Car8 caused defects in motor coordination and cerebellar excitatory circuit formation. However, the functions of Car8 has not been fully understood. In the present study, we produced Car8 knockout mouse and assessed the function of Car8. In the mutant cerebellum, some climbing fibers showed aberrant PC wiring to neighboring PCs. Immunoelectron microscopy revealed that inhibitory terminals frequently formed asymmetrical synapses with PC spines, which were normally innervated by excitatory terminals. Postembedding immunogold revealed that the axo-spinous inhibitory synapses moderately expressed excitatory synaptic components such as AMPA receptor and also expressed inhibitory synaptic components such as GABAA receptor a1 subunit. At dendritic inhibitory synapse, the expression of inhibitory synaptic molecules was significantly decreased. Intriguingly, the axo-spinous inhibitory synapse was suppressed by GluD2 knockdown. These results suggest that Car8 is essential for cerebellar circuit formation and neurochemical matching in cerebellar PCs. (COI:No)

### OP7-3

**Specific Neuroligin3-αNeurexin1 Trans-synaptic Interaction Regulates GABAergic Synaptic Function in an Input Cell Type-Dependent Manner**

Motokazu Uchigashima[1], Kohtarou Konno[3], Emily Demchak[5], Amy Cheung[2], Takuya Watanabe[4], David Keener[2], Manabu Abe[6], Timmy Le[2], Kenji Sakimura[6], Toshikuni Sasaoka[7], Takeshi Uemura[8,9] (*[1]Dept Cel Neuropathol, BRI, Niigata Univ, [2]Dept Neurobiol, Univ Massachusetts Med Sch, [3]Dept Anat, Hokkaido Univ Grad Med Sch, [4]Dept Neuropharm, Fac Pharm Sci, Fukuoka Univ, [5]Dept Biochem & Mol Biol & Inst Perso Med, Penn State Univ Col Med, [6]Dept Animal Model Dev, BRI, Niigata Univ, [7]Dept Comp & Exp Med, BRI, Niigata Univ, [8]Div Gene Res, Res Center Supp Adv Sci, Shinshu Univ, [9]Inst Biomed Sci, Interdis Cluster Cut Edge Res, Shinshu Univ, [10]Dept Pharm Penn State Univ Col Med*)

Synapse formation and regulation require trans-synaptic interactions between pre- and postsynaptic proteins, in particular cell adhesion molecules (CAMs). It has been proposed that the functions of neuroligins (Nlgns), postsynaptic CAMs, rely on the formation of trans-synaptic complexes with neurexins (Nrxns), presynaptic CAMs. Nlgn3 is a unique Nlgn isoform that localizes at both excitatory and inhibitory synapses. However, Nlgn3 function mediated via Nrxn interactions is unknown. Here, we demonstrate that Nlgn3 localizes at postsynaptic sites apposing vesicular glutamate transporter 3-expressing (VGT3+) inhibitory terminals and regulates VGT3+ inhibitory interneuron-mediated synaptic transmission in mouse organotypic slice cultures. Gene expression analysis of interneurons revealed that the αNrxn1+AS4 splice isoform is highly expressed in VGT3+ interneurons as compared with other interneurons. Most importantly, postsynaptic Nlgn3 requires presynaptic αNrxn1+AS4 expressed in VGT3+ interneurons to regulate inhibitory synaptic transmission. Our results indicate that specific Nlgn-Nrxn trans-synaptic interaction provide distinct functional properties at different synapses. (COI:No)

### OP7-4

**Direction-dependent modulation of excitatory synaptic inputs in dendrites of hippocampal neurons revealed by fluorescent imaging of membrane potential**

Masato Morita[1], Shinya Kawaguchi[1] (*[1]Dept Biophys, Grad Sch Sci, Kyoto Univ, Japan*)

In hippocampal pyramidal neurons, many studies have demonstrated the cable property how synaptic potentials propagate from dendrites to the soma. However, their spatio-temporal property remains obscure because of the difficulty in simultaneous measuring membrane potentials of several subcelluer compartments. To study this issue, using a genetically encoded voltage indicator together with a spot glutamate uncaging, we have examined dendritic propagation of EPSPs. An improved version of ASAP1, showing 1 % fluorescence changes as 2 mV membrane potential changes, was expressed in cultured hippocampal neurons. Membrane potential imaging and/or whole-cell patch clamp method detected EPSPs caused by glutamate uncaging at by spot 405 nm laser illumination at a spine. The EPSP tended to substantially attenuate during the propagation toward the soma. In contrast, EPSP surprisingly tended to be augmented during the propagation toward the distal region of dendritic branch, depending on voltage-gated $Ca^{++}$ channels. Thus, our results suggest an interesting dendritic computation of synaptic inputs: excitatory inputs are oppositely modulated depending on the direction of propagation. (COI:No)

### OP7-5

**Modulation of glutamatergic synaptic transmission and neuronal excitability in the medial prefrontal cortex via δ-opioid receptors**

Daisuke Yamada[1], Keita Iio[2], Hiroshi Nagase[2], Akiyoshi Saito[1] (*[1]Lab Pharmacol, Fac Pharm Sci, Tokyo Univ Sci, [2]IIIS, Univ of Tsukuba*)

We previously reported that local perfusion of a selective agonist to δ-opioid receptor (DOP), KNT-127, attenuated the veratrine-induced elevation of extracellular glutamate in the prelimbic medial prefrontal cortex (PL-PFC) and anxiety-like behavior in mice. These results suggested the possibility that KNT-127 suppresses glutamate release from presynaptic site in the PL-PFC. To confirm this, we performed patch-clamp recording from pyramidal neurons in the PL-PFC, and examined the spontaneous and electrically-evoked excitatory postsynaptic currents (EPSC)s. We found that bath application of KNT-127 significantly suppressed frequency, but not amplitude of miniature EPSCs in a DOP-dependent manner. Also, KNT-127 increased paired-pulse ratios in the PL-PFC pyramidal neurons tested. Further, we analysed the firing properties of pyramidal neurons in the PL-PFC, and found that KNT-127 treatment significantly reduced the number of action potentials and rheobase. These results suggested that KNT-127 not only suppresses glutamatergic synaptic transmission by inhibiting glutamate release from presynaptic site, but also reduces cell excitability via DOP in the mouse PL-PFC. (COI:Properly Declared)

### Oral Presentation8

### Neurons, Synapses②

(March 28, Sun. 17：35～18：35, Room10)

### OP8-1

**A neurological analysis of GAD67 knockout rats**

Dongyu Liu[1], Tomokazu Ohshiro[1], Kazuki Fujihara[2], Yuchio Yanagawa[2], Hajime Mushiake[1] ([1]*Dept Physiol, Grad Sch Med, Tohoku Univ, Sendai, Japan,* [2]*Dept Gen Behav Neuro, Grad Sch Med, Gunma Univ, Gunma, Japan*)

GABA is synthesized by two isozymes, glutamic acid decarboxylase (GAD) 65 and 67. GAD65 deficiency causes epilepsy seizures in mice and rats, but it's still unclear that GAD67 deficiency causes any epilepsy in these models.
Here, we found that GAD67 knockout(-/-)rats show the spike and wave discharges(SWDs), an epileptic form of the brain activity, from their infancy, although heterozygous GAD67(+/-) and even wild type rats develop similar SWDs after their adulthood. The SWD could be suppressed by Ethosuximide, a T-type Ca2+channel blocker, but not Phenytoin, a Na+ channel blocker, suggesting the involvement of aberrant activation of the Ca2+ channels. The SWDs are observed in humans during the absence of epilepsy with a brief loss of consciousness. The patients would not respond to external disturbances such as calls during the absence of epilepsy. We tested whether the rats respond to acoustic noises when they show SWD while monitoring the EEG and behavior. The GAD67(+/-)rats responded to the stimulus and SWDs were interrupted. Therefore, SWDs seem not to be related to the absence of epilepsy in these rats. We just have started analyzing the homozygous GAD67 (-/-) rats. (COI:No)

### OP8-2

**Glutamate imaging at the ribbon-type synapses in the goldfish retinal bipolar cell terminal**

Tomoko Oshima[1], Hirokazu Sakamoto[1], Yukihiro Nakamura[1,3], Shigeyuki Namiki[1], Kenzo Hirose[1], Masao Tachibana[1,2,4], Hideki Takago[1,2] ([1]*Dept. of Pharmacol, Grad. Sch. of Med., The Univ. of Tokyo, Tokyo, Japan.,* [2]*Dept. of Rehabilitation for Sensory Functions, Research Inst., Nat'l Rehabilitation Ctr. for Persons with Disabilities, Saitama, Japan,* [3]*Dept. of Pharmacol, Jikei Univ. Sch. of Med. Tokyo, Japan,* [4]*Ctr. for Systems Vision Science, Organization of Science and Technology, Ritsumeikan Univ., Shiga, Japan*)

At the ribbon-type synapses in the sensory organs, presynaptic $Ca^{2+}$ signals that trigger glutamate release show heterogeneity in their size. However, it is yet to be investigated whether glutamate release from individual ribbon-type active zones (AZs) demonstrate this heterogeneity. Further, these cells exhibit kinetically separate (i.e. fast and slow) components of release. Besides, the ribbon-associated and ribbon-free AZs in the retinal bipolar cell terminal might underlie fast and slow components of release, respectively. This hypothesis should be further tested by visualizing the dynamics of glutamate release from multiple AZs. Using a retinal bipolar cell specific type of enhanced glutamate optical sensor (BC-eEOS), we found that BC-eEOS fluorescence intensities for the fast component were variable across individual ribbons, and that sites for the slow component appeared to be spatially confined, i.e. at both ribbon-associated and ribbon-free sites. Thus, the novel imaging method using BC-eEOS enables us to analyze the fast and slow components of the release at multiple ribbon-associated and ribbon-free sites in the retinal bipolar cell terminal. The authors declare no COIs. (COI:No)

### OP8-3

**Decoding of experience: dynamic time warping method for hippocampal ripples**

Dai Mitsushima[1], Koshi Seo[1], Junko Ishikawa[1] ([1]*Department of Physiology, Yamaguchi University Graduate School of Medicine, Japan*)

The hippocampal CA1 is necessary to maintain experienced episodes. Multiple-unit firings of CA1 neurons were recorded from male rats experienced one of four episodes for 10 min: restraint stress, contact with a female, a male, or a novel object. Before an experience, the neurons exhibited sporadic firings with synchronized ripple-like firings (ripples) in habituated home cage. Experience diversified individual ripples in an episode-specific manner in their home cage (Ishikawa et al. BioRxiv 2019).
By using dynamic time warping algorithm, we analyzed the similarity in thousands of ripple-pairs. We used Sakoe-Chiba band to constrain the warping path. Euclidean distance between any ripple-pairs was adjusted by the length of warping path for 2-way ANOVA.
We found significant differences among 4 episodes, in time and interaction. *Post-hoc* analysis further showed difference between any pair of experiences, suggesting episode-specific dynamics in the ripple similarity. Here we successfully specified experienced episode by analyzing Euclidean distances among thousands of ripple-pairs. It is possible to decipher encrypted experience through the deep learning of orchestrated ripples. (COI:No)

### OP8-4

**Molecular mechanisms underlying the coordinated construction of pre- and post-synaptic compartments revealed by FIB-SEM imaging**

Yugo Fukazawa[1], Ruwaida Elhanbaly[1,4], Tatsuya Ishikawa[5], Koshi Murata[1,2], Kazuki Kuroda[1,2] ([1]*Div Brain Struct Funct, Univ Fukui, Japan,* [2]*Life Sci Inov Cent, Univ Fukui, Japan,* [3]*Res Cent Child Men Dev, Univ Fukui, Japan,* [4]*Dept Anat, Histol Embryol, Facult Vet Med, Assiut Univ, Egypt,* [5]*Dept Funct Anat, Kanazawa Univ Sch Med, Japan*)

The synaptic contact is composed of pre- and post-synaptic neuronal compartments and the property of synaptic transmission is regulated by changes at both compartments. Thus, in order to understand the mechanisms underlying synaptic plasticity, it is necessary to study the properties of pre- and post-synaptic compartments of a given synapse simultaneously. Here we introduce a morphological approach that can examine the fine structures of pre-and post-synaptic compartments of individual synapses by FIB-SEM. Quantitative investigation of sub-synaptic components of both sides of a synapse revealed a significant positive correlation among individual components across the two compartments, such as the number of synaptic vesicles and the surface area of postsynaptic membrane specializations, in several distinct synaptic contacts of the mouse. This result indicates that the coordinated changes of pre- and post-synaptic compartments are a common construction rule of the central synapses. We will also present results obtained from mutant mice and discuss genes and molecular mechanisms underlying the coordinated changes of pre- and post-synaptic compartments. (COI:No)

### OP8-5

***In vivo* deep imaging with a vast field of view in the mouse brain utilizing PEO-CYTOP fluoropolymer nanosheets as a novel open-skull window**

Taiga Takahashi[1], Hong Zhang[6,7], Ryosuke Kawakami[4,5,8], Kenji Yarinome[9], Masakazu Agetsuma[10], Junichi Nabekura[3,10], Kohei Otomo[1,2,3,4,5], Yosuke Okamura[6,7,9], Tomomitsu Nemoto[1,2,3,4,5] ([1]*Division of Biophotonics, National Institute for Physiological Sciences, National Institutes of Natural Sciences, Aichi, Japan,* [2]*Biophotonics Research Group, Exploratory Research Center on Life and Living Systems [ExCELLS], National Institutes of Natural Sciences, Aichi, Japan,* [3]*School of Life Science, The Graduate University for Advanced Studies [SOKENDAI], Kanagawa, Japan,* [4]*Research Institute for Electronic Science, Hokkaido University, Hokkaido, Japan,* [5]*Graduate School of Information Science and Technology Hokkaido University, Hokkaido, Japan,* [6]*Department of Applied Chemistry, School of Engineering, Tokai University, Kanagawa, Japan,* [7]*Micro/Nano Technology Center, Tokai University, Kanagawa, Japan,* [8] *Department of Molecular Medicine for Pathogenesis, Graduate School of Medicine Ehime University, Ehime, Japan,* [9]*Course of Applied Science, Graduate School of Engineering, Tokai University, Kanagawa, Japan,* [10]*Division of Homeostatic Development, National Institute for Physiological Sciences, National Institutes of Natural Sciences, Aichi, Japan*)

*in vivo* two-photon imaging in animal brains with a vast field of view has revealed functional connectivity between brain regions. Most researchers employ the open skull method for deep imaging of a living mouse and usually seal the cranial with a glass coverslip. This study developed a novel open skull window by utilizing a newly developed nanomaterial, a polyethylene-oxide-coated CYTOP (PEO-CYTOP) nanosheet. Its thickness was ~130 nm and exhibited a water retention effect and a hydrophilized adhesive surface [Takahashi *et al.iScience*, 2020]. PEO-CYTOP nanosheets firmly adhered to the brain surface of the living mouse, which suppressed bleeding on the surface from a living mouse brain. Moreover, we were able to prepare large cranial windows using PEO-CYTOP nanosheet as a sealing material instead of a glass coverslip, achieving *in vivo* deep imaging of neural structure in a vast field of view at high resolution. This novel technique improves the surgical procedure and expands the optically observable regions, promoting the understanding of the functional connectivity between multiple cortical regions of living animals. (COI:No)

### Oral Presentation9

### Circulation①

(March 29, Mon. 9：00～10：00, Room8)

### OP9-1

**Pathogenetic relevance of calcification to osteogenesis in atherosclerosis of *Apoe*-KO mice**

Masa-Aki Shibata[1], Ryo Takahashi[1], Asuka Takei[2], Chinatsu Shiraoka[1], Azumi Hirata[1], Yoichi Kondo[1] (*[1]Dept. Anatomy and Cell Biol., Osaka Med. Coll., [2]Dept. Plastic Reconstr. Surg., Osaka Med. Coll.*)

Understanding the relationship between calcified lesions and unstable plaques is clinically important.
**Methods:** Calcified plaques in atherosclerosis of *Apoe*-KO mice were analyzed for osteogenic markers.
**Results:** BMP-2 was expressed in normal aortic wall. Expression of BMP-2/4 was elevated in both atherosclerotic plaques and neighboring smooth muscle cells but was markedly decreased in the calcified areas. In real-time RT-PCR analysis, *Runx2* was expressed in normal aorta, and relative levels of *Runx2* were significantly elevated in atherosclerotic lesions compared with the normal aorta. Multiplex immunofluorescence staining for RUNX2-related molecules showed osteoblasts expressing RUNX2 in early and progressive lesions, but the calcified lesions demonstrated many osteoblasts expressing Osterix and RUNX2. Although expressions of MSX2 (a *Runx2* transcriptional repressor) and DLX5 (the transcriptional activator) varied in non-calcified lesions but the expressions were particularly strong in calcified lesions.
**Conclusions:** Pathogenesis of the calcification in atherosclerosis of *Apoe*-KO mice may be closely related to osteogenesis. (COI:No)

### OP9-2

**Mitochondria ultrastructural changes in pulmonary artery ligated right ventricular cardiac muscle.**

Nur Khatijah Mohd Zin[1], Hiroki Bochimoto[1], Naritomo Nishioka[1], Ping Yu Xiong[2,3], Shunsuke Baba[1], Takahiro Inoue[4], Yoichiro Kusakari[1], Jun Tanihata[1], Susumu Minamisawa[1] (*[1]Dept Cell Physiol, Tokyo Jikei Univ. Sch Med, Tokyo, Japan, [2]Dept Med, Queen's Univ. Kingston, ON, CA, [3]Dept Biomed and Mol Sci, Queen's Univ. Kingston, ON, CA, [4]Dept Cardiac Surg, Tokyo Jikei Univ. Sch Med, Tokyo, Japan*)

**Introduction:** Pressure overload in the right ventricle (RV) especially in pulmonary arterial hypertension (PAH) is a major cause of RV dysfunction. The destruction of RV mitochondria plays a role in the mechanism of long-term PAH-induced death.
**Purpose:** Exploring the functional and ultrastructural damages in hyperacute phase of pulmonary artery (PA) ligated cardiac muscle.
**Methods:** PA of male SD-rats (BW>350g) were ligated for 30 seconds, then it was released for 30 minutes to make a RV expansion. The cardiac function was monitored by transthoracic echocardiogram. Intercellular ultrastructure of the RV free wall was then observed by TEM. Using ImageJ, the ultrastructural character of the mitochondria was measured.
**Results:** Despite the fact that the PA ligated sample has minimal to no significant difference in RV ejection fraction and volume, and TAPSE between before and 30 min after ligation, the mitochondrial matrix significantly changes to electron-lucent visually as well as statistically.
**Conclusion:** Even hyperacute PAH, in which cardiac function can be rapidly restored, produce damage in mitochondria, could incur significant injury to the heart muscle in long-term. (COI:No)

### OP9-3

**$PGE_2$-EP4 signaling pathway increases fibulin-1 expression to promote intimal thickening after vascular injury**

Yuko Kato[1], Shota Tanifuji[1], Yuki Okumura[2], Kiku Shimizu[2], Utako Yokoyama[1] (*[1]Depertment of Physiology, Tokyo Medical University, [2]School of Medicine, Tokyo Medical University*)

Remodeling of extracellular matrix favoring vascular smooth muscle cell (VSMC) migration is a crucial in intimal thickening (IT) after vascular injury. We previously reported that the prostaglandin $E_2$ ($PGE_2$) receptor EP4 increased the glycoprotein fibulin-1, regulates VSMC migration. We, therefore, examined roles of $PGE_2$-EP4 signaling in vascular injury-induced IT.
Vascular injury mouse models were made by large wire insertion into femoral arteries in $EP4^{+/+}$ and $EP4^{+/-}$ mice. Expression of fibulin-1 in injured femoral arteries was evaluated by immunohistochemistry. Migration of primary cultured mouse VSMCs was examined by a wound-healing assay.
The area of IT in $EP4^{+/-}$ mice was significantly smaller than that in $EP4^{+/+}$ mice (0.3-fold, $p$<0.01). Fibulin-1 expression was decreased in injured femoral arteries of $EP4^{+/-}$ mice compared to $EP4^{+/+}$ mice. $PGE_2$-induced fibulin-1 mRNA expression in VSMCs was inhibited by an EP4 antagonist (0.5-fold, $p$<0.001). Silencing of fibulin-1 using siRNAs significantly decreased VSMC migration than control RNAs (0.5-fold, $p$<0.001).These data suggest that $PGE_2$-EP4-induced fibulin-1 expression promotes IT after vascular injury via promoting VSMC migration. (COI:No)

### OP9-4

**Development of a novel zebrafish model for aortic aneurysm**

Shota Tanifuji[1], Genri Kawahara[2], Saki Iida[1], Yukiko Hayashi[2], Utako Yokoyama[1] (*[1]Dept Physiol, Tokyo Med Univ, [2]Dept PathoPhysiol, Tokyo Med Univ*)

Aortic aneurysm (AA) is a progressive lethal disease, but no effective pharmacological therapy to inhibit its progression is currently available. Although several rodent models have been developed to simulate the natural history of AA, high-throughput drug screening is not feasible. We, therefore, aimed to develop a novel zebrafish model for investigating AA pathogenesis. To induce AA, we microinjected angiotensin II (AngII) into *Tg(kdrl:EGFP)* zebrafish embryos in which vascular endothelial cells are labelled with EGFP. At 5 days post-fertilization, five parts of the diameter of the dorsal aorta was measured by confocal microscopy. Average aortic diameter was significantly increased in zebrafish injected with AngII (80 or 160 ng) compared to controls (controls, 19.1±0.8 mm; AngII 80 ng, 22.0±0.5 mm; AngII 160 ng, 23.2±0.7 mm; n=13-18). Treatment of AngII combined with the lysyl oxidase inhibitor beta-aminopropionitrile did not enhance the expansion of the dorsal aorta. These results suggest that AngII-injected zebrafish may be a novel model for investigating molecular mechanisms of AA progression and accelerating in-vivo drug screening. (COI:No)

### OP9-5

**A comparison of human and monkey cerebrovascular systems**

Keiichi Sawano[1], Shuichi Tanoue[2], Takeshi Tanaka[3], Yuzuru Hamada[4], Toshi Abe[2], Masato Nakatsukasa[5], Takaakira Yokoyama[8], Hiroaki Hagiwara[9], Syojiro Kato[3], Yoshihiro Yamada[6] (*[1]Yokohama Institute of Angiological Science, [2]Department of Radiology Kurume University School of Medicine, [3]Edogawa Hospital, [4]Primate Research Institute Kyoto University, [5]Dept. Physical Anthropology, Kyoto Univ., [6]Kanagawa Dental Univ., [7]Dept. Bio. Univ. of Tokyo, [8]Dept. Neurosurgery, Hiratsuka City Hospital, [9]Dept. Radiology, Yokohama Minami Kyousai Hospital*)

We performed angiography CT scans with humans and *Macaca fuscata fuscata* (Mff), anatomical research that included several other macaques. This time, we will mainly report on the cranial base and the vicinity of the tentorium cerebelli. The characteristic presence in Mff is that veins originate from the center area of the cranial cavity and go laterally posteriorly at an angle of about 40 degrees, and then merge to part of the Sinus transversus or Sinus sigmoides. This first half (rostral) section is considered to correspond to the basilar vein of Rosenthal in humans. However, in the case of Mff, it is very different in that it descends in the middle, joins the vein one step lower in the horizontal plane toward the lateral rear at almost the same angle, and then faces the horizontal plane toward the lateral rear again. The shape of the section where the sigmoid sinus passes through the Foramen jugulare and transitions to the internal jugular vein is also very different between humans and Mff. Humans show a large flexion of 270 degrees or more, but in Mff there is a gentle bend as in other macaques and apes. The shape of the front superior sagittal sinus is also different. (COI:No)

### Oral Presentation10

### Autonomic nervous system

(March 29, Mon. 9：00～10：12, Room9)

### OP10-1

### Blood flow response in the olfactory bulb to olfactory nerve stimulation and its nicotinic cholinergic regulation

Sae Uchida[1], Fusako kagitani[1] ([1]*Department of Autonomic Neuroscience, Tokyo Metropolitan Institute of Gerontology, Japan*)

Our previous study demonstrated that odor stimulation produces an increase in blood flow in the olfactory bulb, and the blood flow response is influenced by nicotinic acetylcholine receptor activation in the brain. In this study, we investigated the involvement of olfactory nerve for those responses by direct electrical stimulation of olfactory nerve instead of odor stimulation.
In urethane-anesthetized rats, unilateral olfactory nerve stimulation for 5 s produced current (≥100 μA) and frequency-dependent (≥5 Hz) increases in blood flow in the olfactory bulb ipsilateral to the stimulus, without changes in frontal cortical blood flow or mean arterial pressure. The increased olfactory bulb blood flow peaked at 30 ± 7% using stimulus parameters of 300 μA and 20 Hz. The intravenous injection of nicotine (30 μg/kg), a nicotinic receptor agonist, augmented the olfactory bulb blood flow response to nerve stimulation (20 Hz, 300 μA) by approximately 1.5-fold. These results indicate that olfactory nerve stimulation increases olfactory bulb blood flow, and the response is potentiated by the activation of nicotinic cholinergic transmission. (COI:No)

### OP10-2

### Sympathoexcitation and locomotion by dorsal hypothalamic neurons

Emi Narai[1], Tatsuo Watanabe[2], Satoshi Koba[2] ([1]*Div Integrative Physiol, Grad Sch Med Sci, Tottori Univ, Japan,* [2]*Div Integrative Physiol, Fac med, Tottori Univ, Japan*)

We aimed to determine the role of dorsal hypothalamus (DH) neurons in sympatho-motor activation via optogenetics in rats. Rats received DH microinjection of an adeno-associated virus vector that was encoded channelrhodopsin-2 (ChR2) and eYFP. In freely-moving conscious rats (n = 6), laser illumination of DH to activate ChR2 rapidly induced not only pressor and tachycardiac responses but also locomotion. In urethane-anesthetized rats, optogenetic excitation of DH neurons elicited renal sympathoexcitation that was synchronized with the intermittent manner of laser illumination. Of note, it was *post hoc* confirmed that orexinergic neurons which were abundantly distributed in DH were eYFP-negative. These results demonstrate that excitation of DH nonorexinergic neurons is sufficient to elicit sympatho-motor activation, hypothetically being part of the central circuitry that coordinates sympathetic and somatomotor nervous system activation during locomotion.
(COI:No)

### OP10-3

### Analysis of the role of β3-adrenergic receptor in heart rate regulation

Kazuki Yanagisawa[1], Aiko Baba[1], Tomoe Ueyama[2], Takahiro Sogo[2], Shu Nakao[1,2], Teruhisa Kawamura[1,2] ([1]*Department of Biomedical Sciences, College of Life Sciences, Ritsumeikan University,* [2]*Ritsumeikan Global Innovation Research Organization, Ritsumeikan University*)

β1-adrenergic receptor (AR) signaling has a positive chronotropic effect in the heart, but the role of β3-AR, a minor cardiac β-AR isoform, in heart rate regulation remains unknown. β3-ARs are highly expressed in adipose tissue that promote energy expenditure. We here investigated whether β3-ARs are expressed in the sinoatrial node (SAN), the primary pacemaking site, and regulates heart rate in mice. β3-AR transcripts were detected at a modest level in the SAN. Immunolabeling revealed that β3-ARs were expressed at low levels in SAN myocytes and at high levels in adipocytes and nerve fibers in the SAN region. In electrocardiogram recordings *in vivo*, the heart rate was decreased by a β1-AR inhibitor. A subsequent injection of a specific β3-AR inhibitor further reduced the heart rate and prolonged PR intervals. In electrophysiological experiment *in vitro*, SAN-driving intrinsic heart rate was significantly increased by a specific β3-AR agonist, suggesting a β3-AR-mediated regulation of impulse generation and propagation. This mechanism possibly presents in SAN myocytes as well as the adjacent adipose tissue which may provide energy for action potential firing. (COI:No)

### OP10-4

### The somato-lumbar sympathetic nerve reflexes induced by tetanic contractions of the fast-contracting hindlimb muscles help to maintain its contractile force

Harumi Hotta[1], Kaori Iimura[1], Nobuhiro Watanabe[1], Kazuhiro Shigemoto[2] ([1]*Dept Auton Neurosci, Tokyo Metropol Inst Gerontol,* [2]*Dept Gerontol Motor Syst, Tokyo Metropol Inst Gerontol*)

This study aimed to clarify whether reflex excitation of muscle sympathetic nerve induced by tetanic contractions of the fast-contracting muscles modulates its own contractility. In anesthetized male rats, isometric tetanic contractions of the triceps surae muscles were induced by electrical stimulation of the intact tibial nerve (0.2 ms, two times of motor threshold, 100 Hz, for 0.1 s), before and after transection of the lumbar sympathetic trunk (LST), spinal cord, or the lumbar dorsal roots. The amplitude of the tetanic force was reduced, by approximately 10%, at 20 min after transection of either LST, spinal cord, or dorsal roots. Averaging postganglionic sympathetic nerve activity recorded from lumbar gray ramus revealed both spinal and supraspinal reflexes were induced in response to the tetanic contractions. Electrical stimulation of cut peripheral end of the LST (0.5 ms, supramaximal intensity, 5-20 Hz) increased tetanic force amplitude. These results suggest that spinal and supraspinal somato-lumbar sympathetic nerve reflexes induced by tetanic contractions of the fast-contracting hindlimb muscles contribute to the maintenance of its own contractile force.
(COI:No)

### OP10-5

### Cervical disc herniation may cause unilateral or segmental anhidrosis – estimation of the cervical intramedullary sudomotor pathways from a sweating disorder distribution pattern

Yoko Inukai[1], Satoshi Iwase[1], Motohiko Sato[1] ([1]*Department of Physiology, Aichi Medical University School of Medicine, Japan*)

Some patients with unilateral or segmental anhidrosis have cervical disc herniation. Cervical myelopathies may be associated with these pathemas. We analyzed 14 patients aged 37–74 years with those anhidrosis which may be caused by cervical disc herniation. Cases were exposed to 40℃ ambient temperature and examined the sweat distribution (Minor's method), and performed MRI. MRI revealed maximal protrusion of the intervertebral disc to almost anhidrotic side **near the midline** in 89% of the patients with <u>unilateral anhidrosis</u>, and about 3 mm **lateral to the midline** to segmental anhidrotic side in 100% of the patients with <u>segmental hemianhidrosis</u>. In 80% of the latter patients, disc protrusion was corresponded to the segment of anhidrosis. In the median type, the protruded disc may compress the sulcal artery and cause insufficient peripheral perfusion of the sudomotor pathway. In the lateral type, the disc may compress the sympathetic premotor neuron in the dorsolateral funiculus and spare the upper segments synapsing with spinal segmental interneurons and propriospinal neurons. Analysis of the sweat distribution and the lesion can help clarify intramedullary sudomotor pathways. (COI:No)

### OP10-6

### Vasopressin suppresses food intake via V1a receptor and vagal afferent nerves

Tenkou Shimizu[1], Mayu Toyooka[2], Takaaki Koshimizu[3], Toshihiko Yada[4], Yusaku Iwasaki[1,2] ([1]*Anim Sci, Grad Sch Life Env Sci, Kyoto Pref Univ, Kyoto, Japan,* [2]*Anim Sci, Fac Life Env Sci, Kyoto Pref Univ, Kyoto, Japan,* [3]*Div Mol Pharm, Dep Pharm, Jichi Med Univ. Shimotsuke, Japan,* [4]*Div Integr Physiol, Kansai Electric Power Med Res Ins, Kobe, Japan.*)

Arginine-Vasopressin (AVP), a posterior pituitary hormone, reportedly reduces food intake, however its mechanism and physiological significance are unclear. Oxytocin, another neurohypophysial peptide with similar structure to AVP, suppresses feeding via direct interaction with vagal afferents (Y. Iwasaki et al., 2015). In present study, we examined whether AVP directly acts on vagal afferent neurons through AVP receptor, and whether this action on vagal afferents suppress food intake.
AVP increased cytosolic $Ca^{2+}$ concentration ($[Ca^{2+}]_i$) in single nodose ganglion neurons (NGN) isolated from C57BL/6J mice. The incidence of $[Ca^{2+}]_i$ responses to AVP showed a concentration-dependency, with a maximal value around 15% at $10^{-8}$ and $10^{-7}$ M. AVP failed to increase $[Ca^{2+}]_i$ in NGN from V1a receptor knockout (V1aR KO) mice. IP administration of AVP decreased food intake in wild-type mice, but not V1aR KO mice. AVP-induced anorexigenic effect was blunted by subdiaphragmatic vagotomy. In contrast, when ICV injected V1aR antagonist did not affect the AVP-induced anorexigenic effects. In conclusion, AVP directly activates vagal afferents via V1aR, thereby suppressing food intake. (COI:No)

### Oral Presentation11

### Nutritional and metabolic physiology, Thermoregulation

(March 29, Mon. 9：00～10：12, Room10)

### OP11-1

#### Roles of olfactomedin 1 in the alterations of muscle and bone induced by hypergravity in mice

Naoyuki Kawao[1], Takeshi Shimoide[1], Hironobu Morita[2], Yuya Mizukami[1], Yoshimasa Takafuji[1], Masayoshi Ishida[1], Hiroshi Kaji[1] (*[1]Dept Physiol & Regen Med, Kindai Univ Fac Med, Osaka, Japan, [2]Dept Physiol, Gifu Univ Grad Sch Med, Gifu, Japan*)

The interactions between muscle and bone have been recently noted. We previously reported that hypergravity affects muscle and bone through vestibular system in mice. However, its precise mechanisms have not been elucidated. Here, we examined roles of olfactomedin 1 (OLFM1), whose expression was enhanced in the soleus muscles of mice exposed to 3 *g* for 4 weeks compared to 1 *g* mice. Vestibular lesion significantly suppressed OLFM1 expression in the soleus muscle and serum OLFM1 levels enhanced by hypergravity in mice. A phosphatidylinositol 3-kinase inhibitor blunted shear stress-enhanced OLFM1 expression in mouse C2C12 myotubes. As for bone metabolism, OLFM1 antagonized osteoclast formation in mouse bone marrow cells and monocytic RAW264.7 cells. Moreover, OLFM1 suppressed receptor activator of nuclear factor-κB ligand expression and nuclear factor-κB signaling in mouse osteoblasts. Serum OLFM1 levels were positively related to OLFM1 mRNA levels in the soleus muscle and trabecular bone mineral density of mice. In conclusion, the present study showed that OLFM1 contributes to the alterations of muscle and bone induced by hypergravity through vestibular system in mice. (COI:No)

### OP11-2

#### Physiological function of bombesin like peptides in energy metabolism

Ryoko Higa[1], Ikuko Morisaki[2], Kenshiro Shikano[1], Toshikatsu Hanada[2], Reiko Hanada[1] (*[1]Department of Neurophysiology, Faculty of Medicine, Oita University, [2]Department of Cell Biology, Faculty of Medicine, Oita University*)

Neuromedin B (NMB) and gastrin releasing peptide (GRP) is known as bombesin like peptides. These peptides are reported to regulate energy homeostasis, however, the detailed function of endogenous NMB and GRP is not fully understood. To investigate the physiological roles of NMB and GRP in energy homeostasis, we have generated NMB and GRP double gene-deficient mice (dKO). Then, we have examined the body weight in wild-type mice (WT) and dKO mice fed normal diet (ND) or high fat diet (HFD). In HFD, dKO mice had decreased body weight compared with WT, even though both of them had showed similar body weight gain in ND. Next, we were analyzed food intake, oxygen consumption and locomotor activity in order to identify the mechanisms of anti-obese phenotype in dKO mice. There was no significant change between dKO mice and WT mice in food intake and locomotor activity under HFD condition. On the other hand, dKO mice had significantly increased oxygen consumption compared with WT mice, and UCP1 protein level in brown adipose tissue of dKO was clearly elevated than WT. These data indicate that dKO were resistance to diet-induced obesity because of increasing their thermogenesis in BAT. (COI:No)

### OP11-3

#### Intestinal GLP-1 improves hyperglycemia by enhancing insulin sensitivity in a plasma insulin-dependent manner.

Kento Ohbayashi[1], Masaaki Tokuda[2], Toshihiko Yada[3], Yusaku Iwasaki[1] (*[1]Anim Sci, Grad Sch Life Env Sci, Kyoto Pref Univ, Kyoto, Japan, [2]Fac Med, Kagawa Univ, Kagawa, Japan, [3]Div Integr Physiol, Kansai Electric Power Med Res Ins, Kobe, Japan.*)

Glucagon-like peptide-1 (GLP-1) receptor agonist promotes insulin release from pancreatic islets to improve hyperglycemia. We have demonstrated that intestinal GLP-1 secretion after peroral injection of rare sugar D-allulose (Allu) improves glucose tolerance via vagal afferents. However, the mechanisms underlying these effects of Allu, particularly in diabetes, remain to be elucidated. In this study, we examined effect and mechanism of Allu on blood glucose (BG) using lean and diabetes model mice.

Single po injection of Allu did not change BG, although it increased plasma GLP-1 levels in lean mice. In type 2 diabetic model mice presenting with hyperglycemia and hyperinsulinemia, Allu markedly lowered BG without significant insulin secretion, suggesting enhanced insulin action. The Allu-induced BG-lowering was blunted in GLP-1 receptor knockout mice fed high-fat diet. In contrast, type 1 diabetic model mice showing hyperglycemia due to impaired insulin secretion, Allu failed to improve hyperglycemia. In conclusion, intestinal GLP-1 secretion after peroral Allu do not evoke hypoglycemia, and improves hyperglycemia by enhancing insulin sensitivity in the presence of plasma insulin. (COI:No)

### OP11-4

#### Transcriptional coactivator LRPGC1 translocates to the nucleus in response to lactate and promotes lactate metabolism

Takashi Tanida[1], Ken Ichi Matsuda[1], Masaki Tanaka[1] (*[1]Department of Anatomy and Neurobiology, Graduate School of Medical Science, Kyoto Prefectural University of Medicine*)

Lactic acid (LA) is a byproduct of glycolysis resulting from intense exercise or a metabolic defect in aerobic processes. LA metabolism is essential to prevent lactic acidosis, but the mechanism through which LA regulates its own metabolism is largely unknown. Here, we report a LA-responsive protein, named LRPGC1, that particularly mediates LA response. Following LA stimulation, LRPGC1, but not PGC1α, translocates from the cytoplasm to the nucleus through deactivation of nuclear export signals, and thereby interacts with the nuclear receptor ERRγ and upregulates *TFAM*, which ensures mitochondrial biogenesis. Knockout of PGC1 gene in HepG2 hepatocarcinoma cells decreased LA consumption and *TFAM* expression, which were rescued by LRPGC1, but not by PGC1α. These LRPGC1-induced effects were mediated by ERRγ, concomitantly with mitochondrial activation. Notably, liver-targeted silencing of *Lrpgc1* reduced the survival of a mouse model of lactic acidosis, whereas pharmacological activation of ERRγ significantly ameliorated the survival of those mice. These findings demonstrate LA-responsive transactivation via LRPGC1 that highlights an intrinsic molecular mechanism for LA homeostasis. (COI:No)

### OP11-5

#### The effect of microgravity in space flight on histological character of the stomach tissue of mouse.

Hiroki Bochimoto[1], Nur Khatijah Mohd Zin[2], Daisuke Kondo[3], Susumu Minamisawa[1,2] (*[1]Div Aerosp Med, Dept Cell Physiol, Jikei Univ Sch Med, [2]Dept Cell Physiol, Jikei Univ Sch Med, [3]Lab Vet Anat, Obihiro Univ Agricultur Vet Med*)

**Background:** The understanding of nutritional physiology in space is important to living in space. In this research, we analyzed the histological changes occurred in the fundus gland in the tissues of stomach of mice on board the international space station (ISS) for 35 days.

**Methods:** Six of C57BL/6 J male mice were housed under μg in ISS. In contrast, other 6 mice were kept on artificial earth-gravity. In addition, 6 mice were provided as the ground control. All mice were euthanized after experimental period, the stomach tissues were procured and fixed by fixative of 4 % paraformaldehyde. After the fixation, the tissue samples were morphologically analyzed by microscopy.

**Results:** The size of the nucleus and cytoplasmic area of parietal cells were reduced after spaceflight. Interestingly, exposure of mice to 1g using centrifugation cages in the ISS mitigated the area reduction of the parietal cells.

**Conclusion:** Our data suggest that spaceflight leads to promote the proliferation of parietal cells, thereby reducing the relative size of the parietals, and exposure to 1 × g might alleviate the changes of parietal cells homeostasis induced by spaceflight. (COI:No)

### OP11-6

#### Fundamental Research on Behavioral Thermoregulation and Thermal Perception-Using a novel cross-shaped behavioral thermoregulatory system-

Yuta Masuda[1], Riho Sakai[1,3], Kei Nagashima[2,3] (*[1]Graduate school of human sciences, [2]Faculty school of human sciences, [3]Body temperature & fluid laboratory, Waseda University*)

**Introduction** It has been reported that the expression of the thermoreceptor channel (i.e. TRPV1) is downregulated by continuous heat exposure. The change of thermal perception may affect behavioral thermoregulation, but the details of this effect have not been clarified. The present study was designed to develop a new behavioral device and to examine the changes in behavioral thermoregulation when peripheral sensory nerves are desensitized.

**Methods** Ten male C57BL/6J3 mice were used. The behavioral apparatus consisted of five square peltier plates arranged in a cruciform pattern. During the behavioral test, an area set to 32℃, the others set at 38℃ (heat stress). The test conducted for 90min and 32℃ area changed every 5 min. Abdominal temperature ($T_{abd}$) and the %time in the 32℃ area were measured during the experiment. After capsaicin desensitization, the behavioral test was conducted again (post-trial).

**Results and Discussion** Percent time in the 32℃ area decreased by desensitization (78 ± 11% vs 23 ± 8%). $T_{abd}$ was higher in the post-trial (37.1 ± 0.1℃ vs 39.4 ± 0.4℃). The change of peripheral sensation can affect thermal behavior directly. (COI:No)

### Oral Presentation12

### Circulation②

(March 29, Mon. 10：05～11：05, Room8)

### OP12-1

**Expression of prostaglandin receptor EP4 in the developing mouse ductus arteriosus.**

Sayuki Oka[1], Hiroyuki Kuroda[1,2], Utako Yokoyama[1] ([1]*Dept Physiology, Tokyo Med Univ, Tokyo Japan*, [2]*Dept Pediatrics, Yokohama City Univ, Yokohama, Japan*)

The ductus arteriosus (DA) is necessary to maintain fetal life and closes after birth. The $PGE_2$-EP4 signaling plays a critical role in DA closure. However, regulation of EP4 expression in the cardiovascular system is not fully understood. We generated the reporter mice with EP4-IRES-nlsLacZ. X-gal staining was performed to examine EP4 expression. The DA and the aortic arch are derived from neural crest cells. At embryonic day 14 (E14), weak EP4 expression was detected only in the DA, but not in the aortic arch. EP4 was highly expressed in the DA at E18.5, which was gradually decreased after postnatal day 1 (P1) and completely disappeared at P14. EP4 was mainly expressed in the smooth muscle cells, but not in endothelial cells. EP4 was evenly distributed in pulmonary arterial and aortic sides in the mouse DA. EP4 expression became visible in the coronary arteries after birth. These findings suggest that EP4 is specifically expressed in smooth muscle cells of the DA among the cardiovascular system during the fetal period and EP4 expression disappears rapidly after birth. In addition, our study revealed that EP4 is expressed in the neonatal coronary arteries. (COI:No)

### OP12-2

**Effects of abdominal vagal activation on hemodynamics in rats with acute myocardial infarction**

Meihua Li[1], Can Zheng[1], Toru Kawada[1], Masaru Sugimachi[1] ([1]*NCVC·Dep. Cardiovas. Dyna.*)

Vagal nerve stimulation (VNS) is well known to prevent lethal arrhythmias and maintain hemodynamics during acute myocardial infarction (AMI). A novel mechanism for beneficial effects of VNS on cardiovascular diseases has been suggested, the anti-inflammatory effects such as suppression of hepatic production and release of cytokines. We examined whether this anti-inflammatory mechanism contributed to the beneficial effects of VNS on the hemodynamics after AMI in rats. Two minutes after AMI by ligating the left coronary artery in anesthetized rats, we stimulated the cervical right vagal efferent nerve with the intact (IVNS) or denervated (DVNS) abdominal vagal nerve branches for one hour. Rats underwent hemodynamic measurement and liver tissue sampling for tumor necrosis factor (TNF)-a and interleukin (IL)-1b assays. Cardiac index, mean blood pressure, and left ventricular $dp/dt_{max}$ were significantly higher, and liver TNF-a and IL-1b levels were significantly lower in IVNS than DVNS. The electrical stimulation of the cervical vagal efferent fibers improves the hemodynamics after AMI through the anti-inflammatory effects of abdominal vagal nerve activation. (COI:No)

### OP12-3

**S-Nitroso-N-Pivaloyl-D-Penicillamine, a novel inducer of cardiac non-neuronal ACh system, modulates cardiac diastolic function to increase cardiac performance**

Yoshihiko Kakinuma[1], Shino Oikawa[1], Yoko Kai[1], Asuka Mano[1], Shigeo Nakamura[2] ([1]*Department of Bioregulatory Science [Physiology]*, [2]*Department of Chemistry*)

In a previous study, we reported that the non-neuronal cardiac cholinergic system (NNCCS) was equipped with cardiomyocytes to synthesize acetylcholine (ACh), indispensable for cardiac homeostasis. To identify an inducer of NNCCS, we screened chemical compounds with structures similar to that of S-nitroso-N-acetyl-DL-penicillamine (SNAP) with weak induction potency, and identified a novel compound S-nitroso-N-pivaloyl-D-penicillamine (SNPiP). This slowly elevated the intracellular cGMP levels and nitric oxide metabolite, and gradually translated ChAT gene. SNPiP elevated ACh levels 72 h following injection. The SNPiP-treated mice (72 h later) significantly upregulated its cardiac function with enhanced diastolic function leading to enlarged cardiac output, contrasting with 24 h or 48 h after injection. SNPiP improved impaired cardiac function of the db/db heart specifically diastolic function as well as systolic function. In conclusion, SNPiP, a novel NNCCS inducer, could be applied as a therapeutic modality for upregulation of NNCCS and a unique tool for modulating cardiac function through a focus on diastolic function. (COI:No)

### OP12-4

**The therapeutic effects of EPA on pulmonary hypertension via inhibition of tyrosine kinase FYN**

Lin Kurahara[1], Keizo Hiraishi[1], Ying Zhang[2], Hiroko Kishi[2], Sei Kobayashi[2], Aya Yamamura[3], Katsuya Hirano[1] ([1]*Dept Cardiovasc Physiol, Kagawa University, Japan*, [2]*Dept Mol Cell Physiol, Yamaguchi Univ, Grad Sch Med*, [3]*Dept of Physiol, Aichi Medical Univ, Nagakute, Aichi, Japan*)

**Background and Purpose:** Pulmonary hypertension (PH) is a multifactorial disease characterized by PA vasoconstriction and remodeling. The Src family non-receptor tyrosine kinases including Fyn play critical roles in vascular function via the inhibition of STAT3 signaling. Eicosapentaenoic acid (EPA) is known to inhibit Fyn kinase activity. In this study, we investigated the therapeutic potential of EPA and its metabolite RvE1 for PH.
**Results:** EPA treatment ameliorated right ventricular remodeling and dysfunction, and PA medial wall thickening and prolonged survival in PH rats. EPA and RvE1 suppressed vasocontraction of rat and human PA. EPA or RvE1 treatment decelerated the enhanced proliferation of HPASMCs derived from the PH patients. Fyn siRNA prevented TGF-β2-induced EndoMT in HPAECs and IL-6-induced STAT3 phosphorylation. EPA and RvE1 suppressed Src family activity by modulating its autophosphorylation level.
**Summary:** EPA showed therapeutic effect on pulmonary arterial vasoconstriction and remodeling. Our results suggest that the therapeutic effects are likely mediated at least in part via Fyn inhibition. Fyn is suggested to be a potential target for the treatment of PH. (COI:No)

### OP12-5

**Sphingosylphosphorylcholine induced contraction in basilar arteries of Fyn knockout mice.**

Tomoka Morita[1], Ying Zhang[1], Hiroko Kishi[1], Sei Kobayashi[1] ([1]*Department of Molecular and Cellular Physiology, Yamaguchi University Graduate School of Medicine*)

Cerebral vasospasm after subarachnoid hemorrhage can develop severe and sometimes lethal cerebral infarction and is mediated by the $Ca^{2+}$-sensitization of vascular smooth muscle (VSM) contraction. We previously identified a sphingosylphosphorylcholine (SPC)/Fyn/Rho-kinase (ROK) pathway as which induces an abnormal VSM hypercontraction. Although genetically modified mice would be required to study the importance of this pathway *in vivo*, the $Ca^{2+}$-sensitization of abnormal cerebral VSM contraction has been unpublished in mice. The aim of this study was to clarify if SPC could induce abnormal VSM contractions of Fyn knockout (KO) mice basilar arteries. High $K^+$- and the SPC-induced contractions of basilar arteries of both wild type and Fyn KO mice were observed in the presence of L-NAME, a nitric oxide synthase inhibitor. Surprisingly, not only in wild type mice but also in Fyn KO mice, 10 μM single-dose SPC significantly induced contractions of basilar arteries compared with the vehicle control, and there was no significant change between these mice. This result suggested possibilities that unknown signaling molecules except Fyn may mediate SPC/ROK pathway. (COI:No)

### Oral Presentation13

### Digestion, Digestive system, etc.

(March 29, Mon. 10：17～11：05, Room9)

### OP13-1

**Impact of bitter taste substances on the rhythmic contraction of the intestinal tract**

Masato Ota[1], Megumi Imaeda[1], Atsuko yamashita[1] ([1]*Anat. Phys.,Grad. Sch. Hum. Dev. Desi., JWU.*)

Taste is an important sensation for animals to detect nutritious foods and foods containing harmful poisons, and the basic tastes confirmed to date are bitterness, sweetness, taste, sourness, saltiness, and fatness. is there. It has been reported that the bitter taste receptors among them are GPCR-type membrane receptors. It has been shown that these single nucleotide polymorphisms (SNPs) affect the function of binding to taste substances as taste receptors. It has been reported that the small intestine, which is classified into the duodenum, jejunum, and ileum, expresses several TAS2R family genes.
However, the details of the function of these bitter taste receptors are unknown.
We prepared small pieces of mouse intestinal specimens, investigated the effects of neurotransmitters and bitter substances on the rhythmic movement of the small intestine, and analyzed the gene expression of TAS2R family genes. It was suggested that Acetylcholine-dependent rhythmic movements of the small intestine were affected by bitter substances such as quinine and thiamine via TAS2R receptor. (COI:No)

### OP13-2

**Effects of aqueous extracts from wasted Ume seeds on glucose absorption in isolated small intestine of mice**

Tomoo Homma[1], Hiroshi Hirose[1], Satoru Ishihara[2] ([1]*Division of Biotechnology, Graduate School of Engineering, Maebashi Institute of Technology,* [2]*Gunma Agricultural Technology Center*)

Authors reported that aqueous extracts from wasted Ume seeds (AEUS) showed whitening effects (inhibitory effect of tyrosinase activity). In order to study further functionalities of AEUS, the present study investigated effects of AEUS on glucose (Glu) absorption by using everted sac specimen of isolated small intestine of mice. Absorbed quantities of Glu were measured after 20 min since Glu application. In the presence of AEUS (5, 10, or 20%), Glu absorption was inhibited AEUS's concentration-dependently. By analyzing organic acids in AEUS, it was clarified that 0.26% citric acid (CA) and 0.50% malic acid (MA) were included. So, instead of AEUS, organic acids' solution (OA) including 0.26% CA and 0.50% MA was prepared and effects of OA on Glu absorption were examined. Glu absorption was inhibited by 5% OA, but inhibitory quantities by 10% and 20% OA were the same as that of 5% OA. This result suggests other unknown components in AEUS are related with inhibition of Glu absorption. (COI:No)

### OP13-3

**Analysis of alveolar bone loss and molar occlusal angle in Japanese macaques**

Akiko Kato[1], Munetaka Naitoh[2], Koji Inagaki[3,4], Eishi Hirasaki[5], Shintaro Kondo[6], Masaki Honda[1] ([1]*Dept Oral Anat, Aichi-Gakuin Univ Sch Dent,* [2]*Dept Oral Maxillofac Radiol, Aichi-Gakuin Univ Sch Dent,* [3]*Dept Dent Hygiene, Aichi-Gakuin Univ Jr Coll,* [4]*Dept Periodontol, Aichi-Gakuin Univ Sch Dent,* [5]*Sec Evol Morph, PRI, Kyoto Univ,* [6]*Dept Anat, Nihon Univ Sch Dent at Matsudo*)

The pathogenesis of periodontal disease has been debated for a long time. The aim of the present study was to investigate the alveolar bone loss and evaluate occlusal angles in the molars of Japanese macaques by using dental cone-beam computed tomography (CBCT) images. Mesiodistal and buccolingual alveolar bone loss was quantified in the maxillary and mandibular first (M1), second (M2) and third (M3) molars from 18 individuals of *M. fuscata* with CBCT images. The angle between the occlusal plane and the lingual slope of the buccal cusp (occlusal angle) was measured in the maxillary molars. The result shows that the entire root apex was exposed in 89% and 61% of the buccal side of the maxillary M1 and M2, respectively. While, bone loss was less than 30% in the other area. The occlusal angle was largest in maxillary M1 (29.6°), followed by M2 (25.9°), M3 (21.9°). The greatest alveolar bone loss and largest occlusal angle observed in the maxillary M1 suggested that they are possibly related. Further study is needed to elucidate the relations between them.
This work was supported by the Cooperative Research Program of Primate Research Institute, Kyoto University. (COI:No)

### OP13-4

**The measurement of fungiform papillae and filiform papillae using "oral mucosa mirror"**

Sayoko Takano[1], Satoko Tsuchida[2], Ken Yoshimura[2], Shinichi Yamagiwa[3], Junko Takahashi[4], Hinako Sawano[4], Shin-ichi Iwasaki[4] ([1]*Kanazawa Institute of Technology,* [2]*The Nippon Dental University College at Niigata,* [3]*University of Tsukuba,* [4]*Hokuriku University*)

There are a large number of protrusions called lingual papillae on the tongue surface. In this study, we measured the shape and position of the filiform papillae and the fungiform papillae from the base to the apex of the tongue based on tongue surface images using "oral mucosa mirror". The subjects were 20 adolescent men and women. Filiform papillae and fungiform papillae were traced manually on the image files, and their position and size were measured.As a result, the filiform papillae of all subjects distributed almost uniformly from the base to the apex of the tongue, and they were three dimensionally conical shape and fine branches and of their base was elliptical shape. The fungiform papillae distributed mainly from the center of the tongue to the tongue apex, and the number increased toward the tongue apex. They were close to a perfect circle. This study revealed the number and the size of the lingual papillae based on the entire lingual mucosa surface of living humans. (COI:No)

### Oral Presentation14

### Behabior, Biological rhythm, Sleep

(March 29, Mon. 10：17～11：05, Room10)

### OP14-1

**Sleep architecture is shaped by the transcription factor AP-2β**

Ayaka Nakai[1], Tomoyuki Fujiyama[1], Nanae Nagata[1], Mitsuaki Kashiwagi[1], Aya Ikkyu[1], Marina Takagi[1], Chika Tatsuzawa[1], Kaeko Tanaka[1], Miyo Kakizaki[1], Mika Kanuka[1], Taizo Kawano[1], Seya Mizuno[3], Fumihiro Sugiyama[3], Satoru Takahashi[3], Hiromasa Funato[1], Takeshi Sakurai[1], Masashi Yanagisawa[1], Yu Hayashi[1,4]
(*[1]International Institute for Integrative Sleep Medicine [WPI-IIIS], University of Tsukuba, Ibaraki, Japan, [2]Doctoral Program in Neuroscience, University of Tsukuba, Ibaraki, Japan, [3]Laboratory Animal Resource Center, University of Tsukuba, Ibaraki, Japan, [4]Department of Human Health Sciences, Graduate School of Medicine, Kyoto University, Kyoto, Japan*)

The molecular mechanism of sleep remains largely unknown. Here, we focused on the transcription factor AP-2β (TFAP2B). Sleep abnormalities such as short-sleep and sleep-walking have been self-reported in families that carry mutations in *TFAP2B*. Moreover, mutations or knockdown of genes encoding AP-2 transcription factors in invertebrate animals result in reduced sleep-like behavior. Thus, AP-2 transcription factors might have a conserved role in sleep regulation across animal phyla. However, direct evidence supporting the involvement of TFAP2B in mammalian sleep was lacking. By using the CRISPR/Cas9 technology, we generated two *Tfap2b* mutant mouse strains mimicking the mutations in human kindreds that self-reported sleep abnormalities. In addition, we analyzed sleep in mice carrying the *Tfap2b* knockout allele. As a result, different mutations in *Tfap2b* led to diverse effects on the sleep architecture including the reduction or fragmentation of non-REM sleep (Nakai *et al.*, *Genetics* 2020). We are currently addressing the effects of selectively knocking out *Tfap2b* in the nervous system. Based on the results, we will discuss the possible roles of TFAP2B in sleep regulation. (COI:No)

### OP14-2

**The effects of gut microbiota depletion by chronic antibiotic treatment on the sleep/wake architecture and sleep electroencephalogram in mice**

Yukino Ogawa[1], Chika Miyoshi[1], Nozomu Obana[4], Kaho Yajima[2], Noriko Hotta[1], Aya Ikkyu[1], Satomi Kanno[1], Tomoyoshi Soga[2], Shinji Fukuda[2,4,5,6], Masashi Yanagisawa[1,7]
(*[1]WPI-IIIS, Univ. Tsukuba, Ibaraki, Japan, [2]Inst. Adv. Biosci., Keio Univ., Yamagata, Japan, [3]Food Res. Inst., NARO, Ibaraki, Japan, [4]TMRC, Univ. Tsukuba, Ibaraki, Japan, [5]KISTEC-KAST, Kanagawa, Japan, [6]Metabologenomics Inc., Yamagata, Japan, [7]Dept. Mol. Genet., U. Texas Southwestern Med. Center, TX, USA*)

Sleep is an essential physiological process regulated in the brain to maintain biological homeostasis; sleep quality is affected by lifestyle including eating habit. Meanwhile, dysbiosis of the gut microbiota is known to affect brain functions. To investigate the relationship between sleep and intestinal environment, we examined the effects of gut microbiota depletion on sleep. Metabolome profiling of cecal contents showed significant alterations in the metabolic pathways related to neurotransmitters between the antibiotic-induced microbiota-depleted (AIMD) and control mice. The changes include depletion of serotonin in the AIMD mice. Total time of non-rapid eye movement sleep (non-REMS) and REMS during the dark phase increased in the AIMD mice, accompanied by more frequent transitions between non-REMS and REMS. In contrast, total time of NREMS and theta power density of REMS during the light phase decreased in the AIMD mice compared with those in the controls, resulting in a dampening of circadian variations in sleep/wake behavior. These results suggested that the gut microbiota has a potential to affect the sleep quality by altering the balance of neurotransmitters. (COI:Properly Declared)

### OP14-3

**REM sleep-related activity of the medullary neurons in rats**

Yoshifumi Arai[1], Hayato Iwata[2], Tatsuya Suzuki[2], Kaname Mochizuki[2], Yoshimasa Koyama[2]
(*[1]Master's Program in Medical Sciences, Grad Sch of Comprehensive Human Sciences, Univ. of Tsukuba, [2]Dept of Symbiotic System Sciences, Fukushima Univ.*)

The medulla plays crucial roles in the regulation of physiological functions such as the cardiovascular system and REM sleep, which is characterized by EEG desynchronization, muscle atonia or fluctuations of the autonomic nervous system. However, little is known about the medullary neuron's activity during sleep/wake cycles. Thus, we performed extracellular single neuronal recording from the medulla in unanesthetized head-fixed rats in sleep/wake cycles. We recorded 180 neurons and 72 of them (40.0%) were PS active neurons which showed higher activity during REM sleep. Seven of the PS active neurons were selectively active during REM sleep and increased their activity before the onset of REM sleep. Such neurons were recorded from the rostroventral part. Fourty-one (22.8%) were W/PS neurons that showed higher activities during wakefulness and REM sleep and were recorded from the gigantocellular reticular nucleus. Seventeen (9.4 %) were SW/PS neurons that showed higher activity during slow-wave sleep and REM sleep, and 9 of them (52.9%) were located in the ventral area. The results suggest that different part of the medulla plays a different role in the regulation of REM sleep. (COI:No)

### OP14-4

**Vasopressin neurons in the paraventricular hypothalamus promote wakefulness via orexin neurons**

Md Tarikul Islam[1], Florian Rump[2], Yusuke Tsuno[1], Takashi Maejima[1], Michihiro Mieda[1]
(*[1]Department of Integrative Neurophysiology, Kanazawa University, [2]Faculty of Medicine, University of Wuerzburg*)

Arginine vasopressin neurons (AVP) of the paraventricular hypothalamus (PVH) play roles in the regulation of stress response, blood osmolarity, and food intake. However, the role of $PVH^{AVP}$ neurons in sleep-wake regulation remains unknown. Here, we found that optogenetic activation of $PVH^{AVP}$ neurons via a stable step function opsin induced a fast transition from sleep to wakefulness in mice. A similar arousal effect was observed when $PVH^{AVP}$ projections in the lateral hypothalamus (LH) were activated optogenetically. Moreover, designer receptor exclusively activated by designer drugs (DREADD)-based chemogenetic inhibition of $PVH^{AVP}$ neurons significantly increased sleep and reduced wakefulness. Immunohistochemistry revealed projections of $PVH^{AVP}$ neurons to orexin neurons. Besides, administration of suvorexant, a dual orexin receptor antagonist, suppressed wakefulness induced by optogenetic activation of $PVH^{AVP}$ neurons. Intriguingly, in vivo $Ca^{2+}$ measurements in $PVH^{AVP}$ neurons using fiber photometry demonstrated a clear daily rhythm of their neuronal activities. Collectively, $PVH^{AVP} \rightarrow LH^{Orexin}$ pathway may play a role in the daily sleep-wake regulation. (COI:Properly Declared)

### Oral Presentation15

### Neurological disorders, Neuropathophysiology

(March 29, Mon. 14：20～15：20, Room9)

### OP15-1

**Role of IL-17A and ependymal cells in tissue repair mechanism after spinal cord injury**

Hisao Miyajima[1], Takahide Itokazu[2], Shogo Tanabe[2], Masashi Fujitani[1], Toshihide Yamashita[2] (*[1]Department of Anatomy and Neuroscience, Faculty of Medicine, Shimane University, [2]Department of Molecular Neurosciences, Graduate School of Medicine, Osaka University*)

Spinal cord injury (SCI) causes impaired signal transduction between nerves, resulting in loss of central nervous system function. Thus, it is necessary to develop a novel treatment for restoring neural function. Recent studies have suggested that ependymal cells, located in the central canal of the spinal cord, act as neural stem cells and contribute to tissue repair after SCI. However, the molecular mechanism underlying the regulation of ependymal cell activity after SCI remains unclear. In this study, we identified Interleukin (IL) -17A as a regulating factor of ependymal cell proliferation in the injured spinal cord. IL-17A neutralizing antibody treatment promoted functional recovery and axonal reorganization, which also promoted ependymal cell proliferation. In addition, ependymal cell-specific genetic ablation of IL-17RA enhances both axonal growth and functional recovery. Moreover, the mRNA expression of neurotrophic factors was upregulated in injured spinal cord. These results suggest that IL-17A signaling disturbs tissue repair by inhibiting the proliferation of ependymal cells after SCI. (COI:No)

### OP15-2

**Closed-loop brain stimulation for epileptic seizures and beyond**

Yuichi Takeuchi[1] (*[1]Department of Physiology, Osaka City University Graduate School of Medicine, [2]Department of Physiology, Faculty of Medicine, University of Szeged*)

Temporal lobe epilepsy with distributed hippocampal seizure foci is often intractable and its secondary generalization might lead to sudden death. Early termination through spatially extensive hippocampal intervention is not feasible directly, due to the large size and irregular shape of the hippocampus. In contrast, the medial septum (MS) is a promising target to govern hippocampal oscillations through its divergent connections to both hippocampi. Combining this 'proxy intervention' concept and precisely timed stimulation, we report here that closed-loop MS electrical stimulation can quickly terminate intrahippocampal seizures and suppress secondary generalization in a rat kindling model. Precise stimulus timing governed by internal seizure rhythms was essential. Cell-type-specific stimulation revealed that the precisely timed activation of MS GABAergic neurons underlaid the effects. Our concept of time-targeted proxy stimulation for intervening pathological oscillations can be extrapolated to other neurological and psychiatric disorders, and has potential for clinical translation. (COI:No)

### OP15-3

**Physiological roles of VRK2 in the zebrafish**

Ryohei Umeda[1], Nobuyuki Shimizu[2], Kazumasa Hada[2], Kenshiro Shikano[1], Ryoko Higa[1], Hirotaro Urushibata[2], Hiroshi Shiraishi[2], Toshikatsu Hanada[2], Reiko Hanada[1] (*[1]Dept. Neurophysiol, Fac Med, Oita Univ, Japan, [2]Dept. Cell Biol, Fac Med, Oita Univ, Japan*)

Vaccinia related kinase 2 (VRK2), a serine/threonine kinase which is a member of Vaccinia-related kinase family, plays important roles in cell survival and stress response. Recently, it have been reported that mutations of VRK2 gene in human is one of the causes of several psychiatric disorders such as schizophrenia. *In vitro* experiment, there are some evidences that VRK2 is involved in signal transduction related to intracellular nerve cell differentiation and proliferation. However, the physiological role of VRK2 *in vivo* models has not been clarified yet. Then, we have established VRK2 gene-deficient zebrafish (VRK2 KO) and examined the physiological function of VRK2. First of all, we have observed general conditions of VRK2 KO and control zebrafish (WT), and examined morphological changes by H&E staining. In addition, we have performed a series of behavioral analysis to investigate the brain function related to schizophrenia such as aggression, social behavior, and anxiety. Based on these experiments, we have found that aggression and locomotor activity were increased in VRK2 female fish. Now, we are investigating the molecular mechanisms of these phenomena of VRK2KO. (COI:No)

### OP15-4

**Acute motor deficit and subsequent remyelination-associated recovery following internal capsule demyelination in mice**

Reiji Yamazaki[1], Huang Jeffrey [2], Nobuhiko Ohno[1] (*[1]Department of Anatomy, Division of Histology and Cell Biology, School of Medicine, Jichi Medical University, [2]Department of Biology, Georgetown University*)

Multiple sclerosis (MS) is a chronic inflammatory demyelinating disease of the CNS characterized by progressive remyelination failure and accumulated clinical disability. However, whether remyelination promotes motor recovery following demyelinating injury remains unclear. Damage to the internal capsule (IC) is known to result in motor impairment in MS and stroke. Here, we induced focal IC demyelination in mice by lysolecithin (LPC) injection, and examined its effect on motor behavior. We also compared the effect of LPC-induced IC damage to that produced by endothelin-1 (ET1)-induced experimental stroke lesions. We found that LPC or ET1 injections induced motor deficit at 7 days postlesion (dpl), and that both lesion types displayed myelin. The motor deficit and lesion pathology remained in ET1-injected mice at 28dpl. In contrast, LPC-injected mice regained motor function by 28dpl, with corresponding recovery of myelin staining in lesions. These results suggest that LPC-induced IC demyelination results in acute motor deficit and subsequent recovery through remyelination, and may be used to complement future drug screens and identify drugs for promoting remyelination. (COI:No)

### Oral Presentation16

### Oraganelle, Membrane transport

(March 29, Mon. 15：25～16：25, Room9)

### OP16-1

**Functional analysis of the ciliary proteins encoded by ciliopathy-associated genes in hTERT-RPE1 cells**

Zhuoma Yinsheng[1], Ko Miyoshi[1], Sarina Han[2], Yuanyuan Qin[1], Genki Amano[1], Takeshi Yoshimura[1], Taiichi Katayama[1] ([1]*Dept of Child Develop and Molecular Bra Sci, United Grad Sch of Child Develop, Osaka Univ, Osaka, Japan,* [2]*Dept of Biofunctional Imaging, Hamamatsu Univ Sch of Med, Shizuoka, Japan*)

Impaired function of primary cilia results in pleiotropic disorders collectively termed ciliopathies. Joubert syndrome (JBTS) is a ciliopathy characterized by retinal degeneration and brain malformation, while mechanism by which mutations of causative genes such as ARL13B, INPP5E, RPGRIP1L and CEP290 result in JBTS is poorly understood. The ARL13B and INPP5E proteins localize to the ciliary membrane, while the RPGRIP1L and CEP290 proteins localize to the proximal region of the cilium, termed the transition zone, and form a complex that functions as a ciliary gatekeeper. In this study, RPGRIP1L-knock out (KO) and CEP290-KO hTERT-RPE1 cells were established by genome editing. Primary cilia of these KO cells were longer than that of control cells. Further, the localization of the ARL13B and INPP5E proteins at the ciliary membrane was profoundly reduced in the KO cells. The exogenous expression of RPGRIP1L in RPGRIP1L-KO cells rescued the defect. Our finding suggests that the RPGRIP1L and CEP290 proteins contribute to the maintenance of primary cilia at the proper length and the localization of ARL13B and INPP5E at the ciliary membrane. I have no COI with regard to our presentation. (COI:No)

### OP16-2

**TGFβ receptor endocytosis and Smad signaling require synaptojanin1, PI3K–C2α-, and INPP4B-mediated phosphoinositide conversions**

Sho Aki[1], Kazuaki Yoshioka[1], Noriko Takuwa[2], Yo Takuwa[1] ([1]*Department of Physiology Kanazawa University School of Medicine,* [2]*Department of Health and Medical Sciences, Ishikawa Prefectural Nursing Univ*)

Phosphoinositide (PPI) conversion regulates a diverse array of dynamic membrane events including endocytosis. However, it is not well understood which enzymes are involved in phosphoinositide conversions for receptor endocytosis. We found by siRNA mediated knockdown (KD) that class II PI3K α-isoform (C2α), the 5′ phosphatase synaptojanin1 (Synj1), and the 4′-phosphatase INPP4B were required for TGFβ induced receptor endocytosis. TGFβ induced rapid decreases in PI(4,5)$P_2$ at the plasma membrane (PM) with increases in PI(4)P, followed by increases in PI(3,4)$P_2$, in a TGFβ receptor kinase-dependent manner. TGFβ induced the recruitment of both Synj1 and C2α to the PM with their substantial colocalization. KD of Synj1 abolished TGFβ-induced PI(4,5)$P_2$ decreases and PI(4)P increases. Interestingly, C2α KD abolished not only TGFβ-induced PI(3,4)$P_2$ increases but also TGFβ-induced Synj1 recruitment to the PM, PI(4,5)$P_2$ decreases. Finally, the PPI conversions were necessary for TGFβ-induced activation of Smad2/3. These observations demonstrate that the sequential PPI conversions mediated by Synj1, C2α, and INPP4B are essential for TGFβ receptor endocytosis and its signaling. (COI:No)

### OP16-3

**Ultrastructural evidence for an unusual mode of ciliogenesis in mouse multiciliated epithelia**

Keishi Narita[1], Sen Takeda[1] ([1]*Dept Anat Cell Biol, Sch Med, Univ Yamanashi*)

Multiciliogenesis is a cascading process for generating hundreds of motile cilia in single cells. Although the early steps to amplify centrioles have been characterized in molecular detail, subsequent steps to establish multicilia have been relatively overlooked. Here, we focused on unusual cilia-related structures previously observed in wild type mouse ependyma using transmission electron microscopy (TEM) and analyzed their ultrastructural features and the frequency of their occurrence. In the ependyma, approximately 5% of cilia existed as bundles; while the majority of the bundles were paired, bundles of more than three cilia were also found. Furthermore, apical protrusions harboring multiple sets of axonemes were occasionally observed, suggesting an unusual mode of ciliogenesis. In trachea and oviduct epithelia, ciliary bundles were absent, but protrusions containing multiple axonemes were observed. These data suggested that the late steps of multiciliogenesis might include a unique process underlying the development of cilia, which is distinct from the ciliogenesis of primary cilia. (COI:No)

### OP16-4

**Role of Snx9 in myogenesis**

Takumi Adachi[1], Hideshi Sugiura[2], Satoshi Kametaka[2] ([1]*Grad. Sch. Med., Nagoya, Univ,* [2]*Biofunct. Sci., Grad. Sch. Med., Nagoya, Univ*)

Skeletal muscle is an organ with various physiological functions such as maintenance of body temperature, storage of glucose, endocrine, and supporting of immune system, as well as a source of power for physical exercise. In addition, skeletal muscle is also known as a highly plastic tissue. Skeletal muscle repair is thought to follow the similar process of muscle differentiation during development. Therefore, elucidating the molecular mechanisms of the muscle differentiation process in detail will provide important insights into muscle repair. Recently, we found that sorting nexin 9 (Snx9) may play a role in this muscle differentiation process. Snx9 was first discovered as an endocytic accessory protein involved in clathrin mediated endocytosis. In addition, recent studies suggested that Snx9 is a multifunctional scaffold that coordinates membrane trafficking and remodeling with changes in actin dynamics to affect diverse cellular processes. The molecular function of Snx9 in myogenic differentiation, however, remains unknown. This study describes the function of Snx9 and its potential role in the process of muscle differentiation. (COI:No)

### OP16-5

**Novel $Ca^{2+}$ signaling mechanism induced by cancer cell detachment**

Takuto Fujii[1], Takahiro Shimizu[1], Hiroshi Takeshima[2], Hideki Sakai[1] ([1]*Dept. Pharm. Physiol., Fac. Pharm. Sci., Univ. Toyama.,* [2]*Dept. Biol. Chem., Grad. Sch. Pharm. Sci., Kyoto Univ.*)

During the metastatic process, the detached cancer cells from the primary tumor tissue spread to different sites through blood vessels. Cancer cells can survive even under the loss of anchorage, however, the detachment-elicited mechanisms have remained unknown. In this study, we found that intracellular $Ca^{2+}$ concentration ($[Ca^{2+}]_i$) was immediately increased by detachment of human colon cancer HT-29 cells attached on the dish. The $[Ca^{2+}]_I$ increase was blocked by inhibitors of nicotinic acid adenine dinucleotide phosphate (NAADP) and focal adhesion kinase (FAK) but not by inhibitors of phospholipase C and $IP_3$ receptor. Knockdown of SERCA3 $Ca^{2+}$-ATPase significantly attenuated the detachment-induced $[Ca^{2+}]_I$ increase. In addition, measurement of membrane capacitance by patch clamp technique suggested that exocytosis was elicited by cancer cell detachment. The exocytosis was inhibited by NAADP and FAK inhibitors. Interestingly, SERCA3 was translocated to plasma membrane upon cell detachment. These results indicate that cell detachment induces the NAADP- and FAK-dependent $Ca^{2+}$ mobilization from a SERCA3-expressing $Ca^{2+}$ store, which moves to the plasma membrane of cancer cells. (COI:No)

### Oral Presentation17

### Circulation③

(March 29, Mon. 16：30～17：30, Room8)

### OP17-1

**Risk prediction method for drug induced arrhythmia based on early afterdepolarization simulated in mathematical models of human ventricular myocytes**

Akira Kimura[1], Shingo Murakami[2] ([1]*Chuo Univ. Grad. Ele.*, [2]*Chuo Univ. Sci & Eng.*)

The drug-induced arrhythmia is initiated by the occurrence of early afterdepolarization (EAD) under prolonged action potential (AP) due to $I_{Kr}$ block. However, the occurrence of EAD differs among $I_{Kr}$ blockers even under the same prolonged action potential duration (APD). For example, amiodarone causes the prolongation of APD, but does not induce EAD and suppresses arrhythmia in human. On the other hand, terfenadine and bepridil cause the prolongation of APD, but increase the occurrence of EAD. In the present study, we studied how different block kinetics of non-selective $I_{Kr}$ blockers on L-type $Ca^{2+}$ current ($I_{CaL}$) affects the occurrence of EAD. By examining how different blockade kinetics of $I_{CaL}$ affects the frequency of EAD with mathematical models of human ventricular myocytes, we showed that the different drug block kinetics on $I_{CaL}$ can account for the different occurrence of EAD. The $I_{CaL}$ block model of amiodarone suppressed EAD effectively. In contrast, the $I_{CaL}$ block models of terfenadine and bepridil increased the occurrence of EAD. These results suggest that the different risks of non-selective $I_{Kr}$ blockers can be predicted by the voltage dependence in $I_{CaL}$ blockade. (COI:Properly Declared)

### OP17-2

**Cardioprotective effects of eicosapentaenoic acid against saturated fatty acids-caused electrical remodeling**

Masaki Morishima[1], Misato Matsuda[1], Hanako Murakami[1], Katsushige Ono[2] ([1]*Dept Food Sci and Nutr, Fac Agri, Kindai Univ*, [2]*Dept Pthophysiol, Sch Med, Oita University*)

Eicosapentaenoic acid (EPA), mostly contained in fish oil, is known to reduce risks of cardiovascular diseases. Prospective cohort studies have demonstrated an inverse association of arrhythmia with intake of fish oil. However, underlying molecular mechanisms are poorly understood. Here we investigated the effect of EPA on saturated fatty acid-induced electrical remodeling in the heart. Neonatal mouse cardiomyoyctes were cultured with 500 μM palmitate/oleic acid (OAPA: 2:1) in the presence or absence of EPA (10 nM) for 24 h. Increased lipid droplet, ROS accumulation, attenuated mitochondrial membrane potential, and upregulation of cyclooxygenase 2 (COX2) mRNA were demonstrated caused by OAPA, which were all reversed by EPA. EPA also retrieved regular spontaneous beating of cardiomyocytes in association with remodeling of ion channels (*KCNJ3, CACNA1C, CACNA1D, HCN4*), where OAPA caused beating irregularity with changes of these ion channels expression. Our data suggest that EPA prevents abnormal automaticity caused by lipotoxicity in cardiomyocytes. It is also implicated that cardiac electrical excitation could be representing dietary habits and the quality of fatty acid intake. (COI:No)

### OP17-3

**Effects of hyperglycemia and hyperosmolarity on cardiac electrophysiology and hemodynamics in *in vivo* canine model**

Hiroko Izumi-Nakaseko[1], Ai Goto[1], Ryuichi Kanbayashi[1], Yoshiki Hirokawa[1], Mihoko Nagasawa[1], Yoshio Nunoi[1], Yoshinori Takei[2], Akio Matsumoto[3], Shinichi Kawai[4], Jörg Täubel[5], Atsushi Sugiyama[1,2,3,4] ([1]*Dept. Pharmacol., Faculty Med., Toho Univ.*, [2]*Dept. Translational Research & Cellular Therapeutics, Faculty Med., Toho Univ.*, [3]*Dept. Aging Pharmacol., Faculty Med., Toho Univ.*, [4]*Dept. Inflammation & Pain Control Research, Faculty Med., Toho Univ.*, [5]*Richmond Res. Inst., St George's Hosp., Univ. London*)

To explore how extreme increase of plasma glucose level would elicit cardiovascular responses, glucose or mannitol in a dose of 3 g/kg was intravenously infused over 30 min into isoflurane-anesthetized intact dogs (n=4 for each). Mannitol was employed to isolate the effects of hyperosmolarity from those of hyperglycemia. Plasma glucose level and osmolality at the end of glucose infusion were 1,124±57 mg/dL and 335±4 mOsm/kg·$H_2O$, whereas those at the end of mannitol infusion were 89±4 mg/dL and 339±2 mOsm/kg·$H_2O$, respectively. Increases in cardiac output and left ventricular end-diastolic pressure, a decrease in total peripheral vascular resistance and prolongation of atrial effective refractory period were observed by each monosaccharide. Glucose increased heart rate and $LVdP/dt_{max}$ and delayed intraventricular conduction, which were less great for mannitol. Glucose-induced QT/QTc prolongation was earlier in onset and larger in increment than those by mannitol, in which hyperosmolarity-induced increase of $K^+$ gradient across plasma membrane of cardiomyocytes would be enhanced by insulin-induced upregulation of $Na^+$-$K^+$-ATPase along with uptake of glucose *in vivo*. (COI:No)

### OP17-4

**Effects of supplementation of docosahexaenoic and arachidonic acids on fatty acid composition and contractility in cultured rat cardiomyocytes**

Mizuna Yano[1], Yuta Umehara[1], Daisuke Sato[1], Masataka Kusunoki[2], Zhonggang Feng[1] ([1]*Graduate School of Science and Engineering, Yamagata University*, [2]*Research Center of Health, Physical Fitness and Sports, Nagoya University*)

The twitch stress of cardiac tissue equivalent reconstructed in vitro is markedly lower than that in vivo. Previously, we reported that contents of docosahexaenoic (DHA) and arachidonic (AA) acids in cultured rat cardiomyocytes were lower than those in the neonatal myocardium and that contractile fraction of the cells was maximized under 20 μM DHA or 50 μM AA supplementation. In the present study, we evaluated fatty acid composition and contractility in cultured rat cardiomyocytes under simultaneous supplementation of DHA and AA for 11 days (n=4-10). Compared to non-supplemented cells, DHA content was significantly higher ($p<0.05$) under 20 μM DHA and 10 μM AA supplementation, and AA content was significantly higher ($p<0.05$) under 5 or 10 μM DHA and 40 μM AA supplementation. Four days after the onset of 10 μM each for DHA and AA supplementation, the contractile fraction was significantly higher ($p<0.05$) than that in the non-supplemented cells (n=8). In conclusion, we found that simultaneous supplementation of DHA and AA elevated their contents in cultured cardiomyocytes but the fatty acid elevation did not always improve the contractile function. (COI:No)

### OP17-5

**Atrium-specific Pitx2c Overexpression Impaired Sinus Node Function and Increased Atrial Arrythmias**

Shunsuke Baba[1], Toru Akaike[1], Satoko Shinjo[3], Hiroki Bochimoto[2], Susumu Minamisawa[1] ([1]*Department of cell physiology, Jikei medical university, Japan*, [2]*Division of Aerospace Medicine, Jikei university school of medicine*, [3]*Department of Biology, University of Padova*)

**Introduction:** Sinus node (SN) dysfunction is related to atrial fibrillation (AF). The transcription factor Pitx2c which expresses in the left-sided heart may suppress differentiation of SN cells because Pitx2c knockout mice had aberrant pacemaker cells. We hypothesized Pitx2c overexpression in the right atrium (RA) might impair SN function and induce atrial arrhythmias.
**Methods:** We generated the atrial-specific overexpression of Pitx2c by cre-loxP system. We examined the cardiac phenotypes of $Pitx2c^{flox/cre+}$ (OE), $Pitx2c^{flox/cre-}$ (CON) and $Pitx2c^{flox-/cre-}$ (WT) mice.
**Results, Conclusions:** The expression of Pitx2c protein was higher in the RA of OE than CON and WT mice. Electrophysiology study uncovered corrected sinus node recovery time and AF inducibility increased significantly in OE. Telemetry electrocardiography showed the standard deviation of heart rate was significantly larger in OE than other mice because of frequent atrial arrhythmias. Immunostaining revealed the ectopic HCN4 positive cells were expressed in the RA of OE mice. These data indicate Pitx2c may suppress the differentiation of SN cells, and ectopic HCN4 expression in the RA may cause atrial arrhythmias. (COI:No)

### Oral Presentation18

### Molecular anatomy, Molecular physiology①

(March 29, Mon. 16：30～17：30, Room9)

### OP18-1

**Fatty Acid Binding Protein 7 (FABP7)/oleic acid mediated epigenetic regulation drives glioma cell proliferation**

Banlanjo Umaru[1], Yoshiteru Kagawa[1], Yuji Owada[1] ([1]*Grad. Sch. Med., Tohoku Univ., Sendai, Japan*)

Altered lipid metabolism is an important feature in glioblastomas. Although polyunsaturated fatty acids (PUFAs) are associated with decreased tumor growth, recent studies link oleic acid (OA), n-9 PUFA, to enhanced tumor proliferation. Fatty acid-binding protein 7 (FABP7), an intracellular lipid chaperon for especially n-3 and n-9 PUFAs, is highly expressed in glioblastoma and associated with decreased survival. FABP7 is at the center of glioma proliferation, but the mechanisms are still lacking. Here, we examined the effects of FABP7/OA interaction on glioma proliferation. FABP7 overexpression showed an increase in cell proliferation. OA addition boosted FABP7-mediated proliferation. Interestingly, a lipid binding domain mutation in FABP7 abrogated this effect. Also, OA treatment increased FABP7 nuclear translocation, and in turn, increased acetylation of histone H3K27. Moreover, microarray analysis showed that OA enhanced the level of several proliferation related genes. Our findings suggest that FABP7/OA intracellular interaction may modulate the epigenetic status of proliferation related genes and thereby drive tumor cell proliferation. (COI:No)

### OP18-2

**The role of O-GlcNAcylated and phosphorylated beta-actin in the nucleus**

Yoshihiro Akimoto[1], Yuri Miura[2], Akihiko Kudo[1], Gerald W Hart[3], Tamao Endo[2] ([1]*Dept. Anat., Kyorin Univ. Sch. Med.* , [2]*Res. Team Mech. Aging, Tokyo Metro. Inst. Gerontol.*, [3] *Biochem Mol Biol, Comp Carbohyd Res Ctr, Univ. Georgia*)

Although beta-actin is known as the cytoskeletal protein in the cytoplasm, in recent years it has been shown that actin is also involved in the regulation of transcription in the nucleus. Actin is phosphorylated and glycosylated (O-GlcNAc modified), in which single N-acetylglucosamine (GlcNAc) attaches to serine or threonine residue. By mass spectrometry, it has been revealed that there are six O-GlcNAc modified amino acid residues of in the actin, and that three sites (Ser 52, 199, 323) of those are also phosphorylation sites. However, it is unknown whether the glycosylation at these sites is involved in the function of the actin. On the other hand, there are 35 phosphorylation sites. And it was revealed that the phosphorylation of Tyr53,Thr201, 202, 203 are involved in the elongation of the actin filament. In the previous study we found that O-GlcNAcylated and phosphorylated Ser199 actin exists in not only the cytoplasm but also the nucleus. In the present study to examine the role of glycosylated actin and the phosphorylated actin in the nucleus we introduced the anti-glycosylated and phosphorylated-actin antibodies into the cells and examined the morphological changes. (COI:No)

### OP18-3

**Development and evaluation of POLArIS$^{act}$, a molecular orientation probe for F-actin**

Keisuke Sato[1], Ayana Sugizaki[1], Kenta Saito[1], Shalin B. Mehta[2], Mikako Shirouzu[3], Tomomi Tani[4], Sumio Terada[1] ([1]*Department of Neuroanatomy and Cellular Neurobiology, Graduate School of Medical and Dental Sciences, Tokyo Medical and Dental University,* [2]*Chan Zuckerberg Biohub, US,* [3]*RIKEN, Center for Biosystems Dynamics Research, Laboratory for Protein Functional and Structural Biology,* [4]*AIST, Biomedical Research Institute, Functional Biomolecular Research Group*)

Fluorescence polarization microscopy (FPM) can monitor orientation of fluorescent molecules by analyzing the polarization state of the fluorescence. Labeling biomolecules with fluorescent proteins in a rotationally constrained manner is prerequisite for FPM, but universal methods have been lacking. To address this problem, we recently developed POLArIS (Probe for Orientation and L ocalization for Arbitrary Intracellular Structures). POLArIS is a recombinant binder rigidly connected to a fluorescent protein and can target arbitrary biomolecules by combining with phage-display screening.
As an initial test case of POLArIS, we developed POLArIS$^{act}$, which specifically binds to F-actin in living cells. Localization analysis using fluorescently labeled phalloidin in fixed cells and co-expression with other genetically encoded F-actin probes indicated that POLArIS$^{act}$ is an excellent F-actin probe. We confirmed that the orientation of F-actin can be monitored by observing cells expressing POLArIS$^{act}$ with FPM. POLArIS$^{act}$ has been successfully applied to various species and cell types, including mammalian cell lines, murine primary neurons, starfish eggs/early embryos, and fission yeast. (COI:Properly Declared)

### OP18-4

**Mechanism of L-glutamine-induced glucagon-like peptide-1 secretion via taste receptor and cAMP signaling**

Kazuki Harada[1], Takumi Nakamura[2], Taichi Kamiya[1], Mai Takizawa[1], Kazuo Nakajima[2], Tetsuya Kitaguchi[3], Kunihiro Ota[1], Tadafumi Kato[2], Takashi Tsuboi[1] ([1]*Dept. Life Sci., Grad. Sch. Arts Sci., Univ. Tokyo, Tokyo, Japan,* [2]*Lab. Mol. Dynam. Mental Disord., RIKEN CBS,* [3]*CLS, IIR, Tokyo Tech.*)

Glucagon-like peptide-1 (GLP-1), which is secreted from enteroendocrine L cells in the small intestine, promotes insulin secretion from pancreatic β cells and reduces appetite. L-glutamine most potently stimulates GLP-1 secretion among amino acids, and it is reported that L-glutamine induces an increase in intracellular $Ca^{2+}$ levels ($[Ca^{2+}]_i$) and cAMP levels ($[cAMP]_i$). However, the precise mechanism of $[Ca^{2+}]_i$ and $[cAMP]_i$ elevation remains unclear. Here we used mouse enteroendocrine L cell line GLUTag cells and CRISPR/Cas9 technology to address this issue. Inhibition of sodium-coupled amino-acid transporters suppressed the increase of $[Ca^{2+}]_i$, and inhibition of taste receptor type 1 member 3 (TAS1R3) suppressed the increase of $[cAMP]_i$. We then established homozygous TAS1R1- or TAS1R3- C terminal deletion (ΔC) GLUTag cell lines using CRISPR/Cas9. Some TAS1R3-ΔC GLUTag cells did not show the increase of $[cAMP]_i$ or GLP-1 secretion by L-glutamine, while TAS1R1-ΔC cells showed an increase in $[cAMP]_i$ comparable to original cells. These results suggest that TAS1R3 is involved in the induction of L-glutamine-induced GLP-1 secretion via $[cAMP]_i$ elevation. (COI:No)

### OP18-5

**a7nAChR-stimulated ciliary beating in mouse airway epithelia: an increase in $[Ca^{2+}]_i$ mediated via L-type $Ca^{2+}$ channels.**

Daichi Saito[1], Kotoku Kawaguchi[2], Shinji Asano[2,3], Yoshinori Marunaka[3,4,5], Takashi Nakahari[3] ([1]*Department of Molecular Physiology, Graduate School of Pharmaceutical Sciences, BKC, Ritsumeikan University.,* [2]*Department of Molecular Physiology, Faculty of Pharmaceutical Sciences, BKC, Ritsumeikan University.,* [3]*Research Unit for Epithelial Physiology, Research Organization of Science and Technology, BKC, Ritsumeikan University,* [4]*Department of Molecular Cell Physiology, Graduate School of Medical Science, Kyoto Prefectural University of Medicine,* [5]*Research Institute for Clinical Physiology, Kyoto Industrial Health Association*)

Airway ciliary cells isolated from mouse lungs were stimulated by PUN282987 (1 μM, an agonist of a7 nicotinic acetylcholine receptor (a7nAChR)). Stimulation with PUN282987 increased ciliary beat frequency (CBF) and ciliary beat distance (CBD, an index of ciliary beat amplitude) by 10 % and their increases were inhibited by nifedipine (20 μM, an inhibitor of L-type $Ca^{2+}$ channels). PUN282987 slightly increased intracellular $Ca^{2+}$ concentration ($[Ca^{2+}]_i$), which was inhibited by nifedipine. An increase in extracellular $K^+$ concentration (150 mM), which induces depolarization, increased CBF, CBD and $[Ca^{2+}]_i$, which were inhibited by nifedipine and a $Ca^{2+}$-free solution. Mice airway ciliary cells express L-type $Ca^{2+}$ channels (Cav 1.2 ) and a7nAChR. ACh (1 μM) also increased CBF and CBD by 25%, which partially inhibited by nifedipine or MLA (50 nM, an inhibitor of a7nAChR). These results suggest that ACh activates muscarinic AChR (mAChR) and a7nAChR in airway ciliary cells, and a compartment where a7nAChR exists may be different from that where mAChR exists. The role of a7nAChR is still unclear, but it plays an important role in activation of airway ciliary beating. (COI:No)

### Oral Presentation19

### Medical education, New experimental methods

(March 29, Mon. 16：30～17：30, Room10)

### OP19-1

**The effect of simulation-based education before a cadaver dissection course**

Munekazu Naito[1], Tomiko Yakura[1], Naoyuki Hatayama[1], Shuichi Hirai[1], Takashi Nakano[1]
(*[1]Aichi Medical University Department of Anatomy Aichi Medical University*)

Simulation-based education (SBE) has been widely shown to be effective as an educational method that enhances practical skills by integrating learners' knowledge and skills. This study aimed to confirm the effect of SBE as preparatory education before the cadaver dissection course on the dissection skills and anatomical knowledge of medical students. All teachers had the history of using knives, carving knives and chisel, but all students not. Also, to examine the effectiveness of SBE, self-contentment scores (SCS), self-efficacy scores (SES), and written examination scores (WES), students' questionnaire results were statistically analyzed. Two years after the introduction of SBE, SES and WES significantly increased, but SCS scores did not. Furthermore, incidence of injuries were analyzed. Findings showed that the introduction of SBE improves Incidence of injuries decreased, students' SES and improves their acquisition of knowledge evident on WES. Conclusions are that SBE as preparatory education may effectively promote the fusion of dissection skills and anatomical knowledge for greater student success in the cadaver dissection course. (COI:No)

### OP19-2

**Gross anatomy practical course with active defense against COVID 19 resulted in success of education without omission of contents**

Takaichi Fukuda[1], Shigeyuki Esumi[1], Naoki Shigematsu[1], Yuta Miyamoto[1], Yoshihiro Kumagai[1], Yoshikazu Koba[1] (*[1]Grad. Sch. Med. Anat, Kumamoto Univ*)

Gross anatomy practical course needs serious countermeasures to prevent COVID 19 infection among participants, because the course is inevitably accompanied with risky situations. There will be two directions in dealing with the risk. One is restriction in many aspects: decreasing the number of participants, shortening the schedule and reduction of educational contents. But all these measures lead to insufficient education. Thus we decided to take another direction to secure educational contents by active defense. Sufficient ventilation was maintained at each table, which was confirmed by authorized experts. Participants were obliged to wear both surgical masks and face shields. We did not have students refrain from discussion, because discussion is essentially important; students teach each other, solve the problem by themselves through discussion, and learn to execute missions by group work. We strictly restricted the number of students in the changing room and at sinks. These countermeasures permitted us to enforce the standard practical course without reduction of educational contents lasting 14 weeks. Spread of infection including COVID 19 did not occur during the course. (COI:No)

### OP19-3

**Sparse functional connectivity of cortical neurons during sleep revealed by calcium imaging and graphical lasso**

Takeshi Kanda[1], Takehiro Miyazaki[1], Hideitsu Hino[2], Masashi Yanagisawa[1]
(*[1]University of Tsukuba, [2]ISM*)

Sleep refines dynamic properties of the brain through its modulatory effects on the cerebral cortex. The exact nature of modulation, however, is poorly understood. Here we investigated changes in cortical network dynamics during spontaneous sleep/wake states using two-photon calcium imaging in the motor cortex. Functional connectivity, that is, covarying activity between two neurons, was estimated with a statistical learning approach graphical lasso. Individual neural activity decreased in non-rapid eye movement sleep (NREM) sleep and increased in REM sleep. Functional connectivity was sparse in NREM sleep and dense in REM sleep. Sleep deprivation is known to disrupt brain functions. We examined if sleep deprivation and its recovery sleep affect local cortical connectivity. Sleep deprivation induced strong connectivity. Subsequent NREM sleep after sleep deprivation exhibited weak connectivity. Functional connectivity is a cause of synaptic plasticity. Taken together, these findings indicate that local cortical connectivity becomes quite dense without NREM sleeps, which could cause saturation of learning ability such as synaptic potentiation (COI:No)

### OP19-4

**Nonwoven nanofibers as artificial corneal materials**

Davood Kharaghani[1] (*[1]Davood Kharaghani, [2]Yuji Yoshiko, [3]Ick Soo Kim*)

This study aimed to produce composite nanofibers scaffolds with high potential bioactivity to encourage epithelization for artificial cornea application and the possibility of using nanohydroxyapatite as the bioactive material in the artificial cornea. An electrospinning method was employed to prepare composite nanofibers from polyvinyl alcohol - hydroxyethyl cellulose - graphite that was cross-linked by glutaraldehyde. The prepared scaffolds were subjected to dipping in calcium and phosphate solutions to load different amounts of nHA on the surface of composite nanofibers. *In-vitro* and *in-vivo* cell attachment results indicated that nHA had a significant effect on epithelial cell growth and immigration into the scaffolds. The results showed scaffolds with large amounts of nHA were not suitable for soft tissue regeneration although scaffolds without or with a small amount of nHA were promising candidates for artificial cornea implantation. (COI:No)

### OP19-5

**A new system of a Water-in-oil Droplet discharge electroporation for cell transfection performance.**

Rika Numano[1], Rika Numano[1], Minako Matsuo[1], Ryuto Shinozaki[1], Hirofumi Kurita[1], Kojiro Matsumoto[2] (*[1]Toyohashi University of Technology, Department of Applied Chemistry and Life Science, [2]Nepa Gene Co., Ltd.*)

The water-in-oil(W/O) droplet electroporation system which we have developed in 2015, is cell transfection methods using dielectric oil and an aqueous several micro liter droplet containing mammalian cells and transgene DNA. An aqueous droplet performed bouncing motion so frequently between a pair of electrodes by Coulomb force in dielectric oil and short cutting during applying a DC electric field. Then, the instantaneous short circuit via the droplet facilitates gene transfection by this W/O droplet electroporatio. However, there were some problems of improving the operation and the reproducibility of gene transfection. In this study these points have been improved with stability of performance according to holding the oil-free droplet between a pair of electrodes and introducing a pulsed electric field due to electric discharge into droplet. The prototype electroporator has been created in this format consisted of a power supply and a power limiting capacitor for limiting electrical power to reduce damage to cells. We performed to create the useful cells in the medical field by Crispr/Cas9 genome editing using this W/O droplet electroporation. (COI:No)

### Oral Presentation20

### Glia

(March 29, Mon. 17：35～18：47, Room8)

### OP20-1

**Astrocytes perform phagocytosis under microglial dysfunction**

Hiroyuki Konishi[1], Takayuki Okamoto[1], Katsuaki Sato[2], Hiroshi Kiyama[1] ([1]*Dept. Funct. Anat. & Neurosci., Nagoya Univ. Grad. Sch. Med.*, [2]*Div. Immunol., Dept. Infect. Dis., Fac. Med., Univ. Miyazaki*)

Microglia are the principal phagocytes in the central nervous system (CNS). In *Siglech*$^{dtr}$ mice, which enable highly specific ablation of microglia, microglial debris were rapidly cleared even under the absence of functional microglia, raising a question which cells removed the debris. The microglial ablation did not cause infiltration of non-microglial mononuclear phagocytes, such as perivascular macrophages and circulating monocytes, in CNS parenchyma, suggesting that non-professional phagocytes cleared microglial debris. We found by RNA-seq that astrocytes became pro-inflammatory activated upon microglial ablation. The activated astrocytes extended their processes to phagocytose microglial debris. Besides this ablation model, phagocytic astrocytes were also observed in Irf8-deficient mice, in which microglia were present but dysfunctional. TAM phagocytic receptors, which play central roles in the clearance, were expressed by astrocytes even in the physiological condition, suggesting that astrocytes possess phagocytic property and stand by in case of microglial impairment. This compensatory function of astrocytes may contribute to the maintenance or prolongation of a healthy CNS. (COI:No)

### OP20-2

**Visualization of corticospinal tract and microglia in spinal cord injury mice using *in vivo* two photon microscope**

Ryotaro Oishi[1], Hiroaki Wake[2], Daisuke Kato[2] ([1]*Department of Orthopedic Surgery, Nagoya University Graduate School of Medicine*, [2]*Department of Anatomy and Molecular Cell Biology, Nagoya University Graduate School of Medicine*)

Spinal cord injury (SCI) is a severe condition that leads to impairment in motor or sensory function. Glial scar is formed after SCI and it inhibit axonal regeneration. Recent studies have revealed the molecular and cellular features of microglia in glial scar formation. However, many of these studies have only focused on clarifying the relations between microglia and corticospinal tract *in vitro* or posterior funiculus *in vivo*, and the causal relation is still unclear. Thus, to study the effect of glial scar on axonal regeneration, we developed the system to visualize the microglia and corticospinal tract *in vivo* over time.

We injected an adeno-associated virus (AAV) carrying the tdTomato gene into the layer 5 of left primary motor cortex in CX3CR1-EGFP mice. Four weeks after AAV injection, spinal cord was exposed at the apex vertebra with a laminectomy and chamber was firmly attached with dental cement in SCI and sham operation mice. We successfully observed the interactions between axon and microglia using *in vivo* two photon microscope. Now we are trying to assess whether microglial motility affects the axonal behavior and whether it defines the recovery of motor function. (COI:No)

### OP20-3

**Hypothermic culture attenuates neuronal apoptosis via prolonged erythropoietin secretion from astrocyte**

Kohki Toriuchi[1], Hiroki Kakita[1,2], Hiromasa Aoki[1], Tetsuya Tamura[3], Satoru Takeshita[1,2], Yasumasa Yamada[2], Mineyoshi Aoyama[1] ([1]*Department of Pathobiology, Nagoya City University School of Pharmaceutical Sciences*, [2]*Department of Perinatal and Neonatal Medicine, Aichi Medical University*, [3]*Department of Anesthesiology and Intensive Care Medicine, Nagoya City University Medical School*)

Hypoxic-ischemic encephalopathy (HIE) involves severe neurologic deficits. Therapeutic hypothermia (TH) has been shown to provide neuroprotection in infants with HIE. Although the cellular mechanism of the neuroprotective effect of TH remains unclear, astrocytic erythropoietin (EPO) is known to be a key mediator of neuroprotection under hypoxic conditions. In the present study, we investigated whether hypothermia attenuates neuronal damage via astrocytic EPO expression. Rat cortical astrocytes were cultured under oxygen/glucose deprivation (OGD). After OGD, astrocytes were cultured under normothermic (37℃) or hypothermic (33.5℃) conditions. OGD induced EPO expression, although at lower levels than hypoxia alone. EPO expression after OGD was significantly higher under hypothermia. Moreover, expression of HIF-1$\alpha$ and HIF-2$\alpha$ protein was enhanced under hypothermia. In the presence of astrocyte conditioned medium (ACM) derived from hypothermic astrocytes following OGD, neuronal apoptosis was suppressed. Hypothermia after OGD stabilized HIF-EPO signaling in astrocytes, and upregulated EPO expression could suppress neuronal apoptosis. (COI:No)

### OP20-4

**Astrocytes regulate synapse elimination in the developing cerebellum through type 2 inositol 1,4,5-trisphosphate receptor**

Naofumi Uesaka[1], Tsubasa Akamatsu[2], Honoka Suzuki[2], Masanobu Kato[2] ([1]*Dept. Neurobio, TMDU*, [2]*Dept.Neurophy, Grad Medicine, Univ Tokyo*)

Elimination of unnecessary synapses in the developing nervous system is a fundamental process to organize functional neural circuits. In the neonatal cerebellum, Purkinje cells (PCs) receive multiple climbing fiber (CF) inputs. A single CF is then selectively strengthened and translocates to PC dendrites while the other CFs around PC somata are eliminated. Bergmann glia, specialized unipolar astrocytes in the cerebellar cortex, are closely associated with PCs and shown to be involved in CF synapses elimination. However how Bergmann glia contribute to CF synapse elimination remains unclear. Here we tested the hypothesis that $Ca^{2+}$ activity through IP3R2 in Bergmann glia contributes to CF synapse elimination. Electrophysiological analyses demonstrated that IP3R2 was necessary for CF synapse elimination. Morphological analyses revealed that elimination of CF terminals from PC somata was impaired in IP3R2 knockout (KO) mice. Neither glutamatergic parallel fiber (PF)-PC synapses nor inhibitory synapses on PCs were affected in IP3R2-KO mice. These results suggest that astroglial IP3R2-mediated signals are transmitted to PC somata or CF terminals to promote their elimination. (COI:No)

### OP20-5

**The anti-inflammatory property of human bone marrow-derived mesenchymal stem/stromal cells (hMSCs) is preserved in late-passage cultures**

Hirokazu Ohtaki[1], Dandan Song[1], Atsuko Ishii[1,2], Kazumichi Yagura[1,3], Akira Yoshikawa[4], Yutaka Hiraizumi[5], Kazuho Honda[1] ([1]*Dept. Anat., Showa Univ. Sch. Med., Tokyo, Japan*, [2]*Div. Toxicol., Dept. Pharmacol., Toxicol. and Therapeutics, Showa Univ. Sch. Pharmacy, Tokyo, Japan*, [3]*Dept. Orthopedic Surg., Showa Univ. Fujigaoka Hosp., Yokohama, Kanagawa, Japan*, [4]*Dept. Physiol., Showa Univ. Sch. Med., Tokyo, Japan*, [5]*Dept. Orthopedic Surg., Showa Univ. Sch. Med., Tokyo, Japan*)

hMSCs were approved recently to treat for spinal cord injury, and is a prospective candidate for brain damages. We have reported that hMSC transplant decreased hippocampal cell death after ischemia in mice, and suggested to suppress inflammatory responses mediated by modulation of microglia/macrophages. On the other hands, hMSCs in the late-passaging lose stem cell property such as proliferation, colony formation and differentiation, and are considered not to suitable for the therapy. However, it is still unclear for the anti-inflammation. We examined anti-inflammatory property of hMSCs using mixed culture with mouse microglial cell line, BV-2. Although BV-2 increased NO by INF$\gamma$, it was suppressed mixed with passage 3 hMSCs in hMSCs number-dependent fashion. Mixed with inviable hMSCs or human fibroblasts was no or low suppression, and the effect was suggested to require a cell-cell communication. Late-passage hMSCs, which decrease stem cell properties, or hMSCs interfered PPAR$\gamma$, a key factor for adipocyte differentiation, also suppressed NO production. These results suggested that the anti-inflammatory property of hMSCs is preserved more than the stem cell property. (COI:No)

### OP20-6

**Thyroid hormones (TH) modulate the membrane receptors and affect the migration of astrocytes**

Winda Ariyani[1], Wataru Miyazaki[1,3], Koibuchi Noriyuki[1] ([1]*Department of Integrative Physiology, Gunma University, Japan*, [2]*Research Fellow of Japan Society for the Promotion of Science, Japan*, [3]*Department of Bioscience and Laboratory Medicine Hirosaki University Graduate School of Health Sciences*)

Activation or modulation of the membrane receptors plays a vital role in the migration of astrocytes. TH can modulate membrane and nuclear receptors in astrocytes. However, the effects of TH in astrocyte are poorly understood. Thus, we examined TH effects on astrocyte by wound healing, cell migration assay, and western blot analysis. We found that TH increased cell migration and adhesion in astrocytes with increased actin remodeling, focal adhesion proteins, and phosphorylation levels of FAK, ERK1/2, and Akt. Both overexpressed and knockdown of TR$\alpha$ and TR$\beta$ did not affect TH-induced cell migration. Besides, the knockdown of integrin $\alpha v\beta 3$ also did not reduce the effects. However, knockdown of Grb2 as a direct target of RTK significantly reduced TH-induced cell migration, focal adhesion protein, and phosphorylation levels. Also, only the activation of FAK and Akt by TH will increase Rac1/Cdc42 phosphorylation to increased actin remodeling and astrocyte migration. These findings indicate that THs exposure exerts their action through the TR-independent pathway by modulating RTK, which may play an essential role in the migration of astrocytes during brain development or injury. (COI:No)

### Oral Presentation21

### Molecular anatomy, Molecular physiology②

(March 29, Mon. 17：35～18：35, Room9)

### OP21-1

**Optogenetic control of small GTPases reveals RhoA-mediated intracellular calcium signaling**

Hironori Inaba[1], Qianqian Miao[1], Takao Nakata[1] (*[1]Department of Cell Biology, Graduate School of Medical and Dental Sciences, Tokyo Medical and Dental University*)

Rho/Ras family small GTPases are known to regulate numerous cellular processes, including cytoskeletal reorganization, cell proliferation, and cell differentiation. These processes are also controlled by $Ca^{2+}$, and consequently, crosstalk between these signals is considered likely. However, systematic quantitative evaluation is not yet reported. Thus, we developed optogenetic tools to control the activity of small GTPases (RhoA, Rac1, Cdc42, Ras, Rap, and Ral) using an improved light-inducible dimer system. Using these optogenetic tools, we investigated $Ca^{2+}$ mobilization immediately after small GTPase activation. Unexpectedly, we found that only RhoA activation induced a transient intracellular calcium elevation in RPE1 and HeLa cells. Transients were also observed in MDCK and HEK293T cells by RhoA activation, but interestingly, molecular mechanisms were identified to be different among cell types. In RPE1 and HeLa cells, RhoA activated phospholipase C (PLC) ε at the plasma membrane, which in turn induced $Ca^{2+}$ release from the endoplasmic reticulum. The RhoA-PLC ε axis induced $Ca^{2+}$-dependent NFAT nuclear translocation, suggesting it does activate intracellular calcium signaling. (COI:No)

### OP21-2

**The mechanism of microtubule nucleating center formation by CAMSAP2**

Tsuyoshi Imasaki[1], Satoshi Kikkawa[1], Shinsuke Niwa[6], Yumiko Saijo-Hamano[1], Hideki Shigematsu[2], Kazuhiro Aoyama[4,5], Kaoru Mitsuoka[5], Mikako Shirouzu[3], Masatoshi Takeichi[3], Ryo Nitta[1] (*[1]Division of Structural Medicine and Anatomy, Kobe University Graduate School of Medicine, [2]RIKEN SPring-8 Center, [3]RIKEN Center for Biosystems Dynamics Research, [4]Materials and Structural Analysis [ex FEI], Thermo Fisher Scientific, [5]Research Center for Ultra-High-Voltage Electron Microscopy, Osaka University, [6]Frontier Research Institute for Interdisciplinary Sciences, Tohoku University*)

Microtubules are dynamic protein polymer consisting of tubulin heterodimer, essential for fundamental cellular functions. In the centrosome, the initiation of polymerization of tubulins to form microtubules called nucleation is regulated by gamma-tubulin ring complex. On the other hand, the nucleation mechanism of non-centrosomal microtubules, which grow independently from centrosomal microtubules, is still under investigation. Here we demonstrate that minus-end-binding calmodulin-regulated spectrin-associated protein 2 (CAMSAP2) serves as a strong nucleator for microtubule from soluble tubulins independent from gamma-tubulin. CAMSAP2 stabilizes longitudinal contact of tubulins to form filamentous structure to reduce nucleation barrier, which is close to the critical concentration of microtubule in the cells. Electron microscopy study showed CAMSAP2 and tubulins clusters together to form nucleation intermediates from which numerous microtubules radiate outward, resembling the centrosomal aster,. Our findings suggest that CAMSAP2 supports microtubule growth by organizing a nucleation center as well as by stabilizing microtubule nucleation intermediates. (COI:No)

### OP21-3

**A molecular mechanism of cisplatin sensitivity regulated by actin filaments in cancer cells**

Takahiro Shimizu[1], Takuto Fujii[1], Hironao Otake[1], Hideki Sakai[1] (*[1]Dept Pharm Physiol, Fac Pharm Sci, Univ Toyama*)

An anticancer drug, cisplatin, triggers apoptosis in various cancer cells. However, cancer cells gradually acquire tolerance to cisplatin. We previously reported that by protein comparison between the cisplatin-sensitive human epidermoid carcinoma KB cells and its cisplatin-resistant KCP-4 cells, the actin filaments are essential for the activation of volume-sensitive outwardly rectifying anion channels (VSOR). Since VSOR activities are known to be a prerequisite for the induction of apoptosis, we here investigated whether the formation of actin filaments contributes to cisplatin-triggered apoptosis. Cisplatin significantly decreased cell viability in KB cells, which was inhibited by co-treatment with cytochalasin D, an inhibitor of actin polymerization. Besides, cytochalasin D suppressed an increase in caspase-3/7 activities and apoptotic volume decrease after cisplatin application. Intriguingly, the treatment of KCP-4 cells with trichostatin A, a histone deacetylase inhibitor, recovered the actin filament formation and the cisplatin sensitivity. These results suggest that the actin filament-dependent activation of VSOR regulates the cisplatin sensitivity of KB cells. (COI:No)

### OP21-4

**Osteoprotegerin-dependent M-cell self-regulation balances gut infection and immunity**

Shunsuke Kimura[1], Yutaka Nakamura[1], Nobuhide Kobayashi[1,2], Koji Hase[1] (*[1]Div. Biochem., Fac. Pharma., Keio Univ., [2]Dep. Bacterio., Grad., Med., Kanazawa Univ., [3]Lab. Histo., Fac. Med., Hokkaido Univ.*)

Microfold cells (M cells) are responsible for antigen uptake to initiate immune responses in the gut-associated lymphoid tissue (GALT). Receptor activator of nuclear factor-κB ligand (RANKL) is essential for M cell differentiation. Follicle-associated epithelium (FAE) covers the GALT and is continuously exposed to RANKL from stromal cells underneath the FAE, yet only a subset of FAE cells undergoes differentiation into M cells. Here, we show that M cells express osteoprotegerin (OPG), a soluble inhibitor of RANKL, which suppresses the differentiation of adjacent FAE cells into M cells. Notably, OPG deficiency increases M-cell number in the GALT and enhances commensal bacterium-specific immunoglobulin production, resulting in the amelioration of disease symptoms in mice with experimental colitis. By contrast, OPG-deficient mice are highly susceptible to infection with *Salmonella*. Thus, OPG-dependent self-regulation of M-cell differentiation is essential for the balance between the infectious risk and the ability to perform immunosurveillance at the mucosal surface. (COI:No)

### OP21-5

**Generation of Cre-inducible gene expression system without non-specific "leaky" gene expression**

Yasuyuki Osanai[1], Tobias Merson[2], Nobuhiko Ohno[1,3] (*[1]Division of Histology and Cell Biology, Jichi Medical University, [2]Australian Regenerative Medicine Institute, Monash University, [3]Division of Ultrastructural Research, National Institute for Physiological Sciences*)

Cre-inducible gene expression systems such as lox-STOP-lox cassette are widely used for spatiotemporal regulation of transgene expression. Using lox-STOP-lox and P2A peptide, we generated a Cre-responsive bicistronic vector, pCAG-lox-GFP-STOP-lox-rtTA-P2A-mCherry. Unexpectedly, upon transfection into cells, > 7% of transfected cells misexpressed mCherry without Cre recombination, whereas rtTA was expressed in a strictly Cre-dependent manner. When the order of mCherry and rtTA coding sequences was swapped, mCherry expression was tightly regulated but rtTA expression became leaky, suggesting that expression of the second gene is not efficiently blocked by the upstream STOP cassette. Surprisingly, bicistronic constructs lacking promoters also expressed the second genes. This study indicates that coding sequences positioned 3' of the P2A sequence in an inducible bicistronic expression vector is leaky because CDS located at the 5' region behave as a promoter. Applying this finding, we generated vectors that express target genes strictly in a Cre dependent manner. The new vectors will be versatile tools for analyzing morphologies/functions in specific target cell types. (COI:No)

### Oral Presentation22

### Gross anatomy①

(March 30, Tue. 9：00～10：00, Room9)

### OP22-1

**An anatomical study of tunnel structures for the ulnar nerve at the medial elbow: Relationship to the ulnar collateral ligament**

Rintaro Yamamoto[1], Mizuki Izumida[1], Tohma Sakuraya[1], Kenji Emura[2], Takamitsu Arakawa[1] (*[1]Grad. Sch. Health Sci., Kobe Univ., [2]Fac. Health Care Sci., Himeji Dokkyo Univ.*)

Ulnar nerve passes through the osseofibrous tunnel called cubital tunnel. Although previous researches reported that ulnar nerve disorder in cubital tunnel could be accompanied by ulnar collateral ligament (UCL) injury, however, the detailed anatomical relationship between cubital tunnel and UCL is still unclear despite their close location. Thus, we investigated tendinous structures including UCL through which ulnar nerve passes at the medial elbow. 23 elbows of 15 cadavers were analyzed after muscle bundles removed. At the proximal elbow, ulnar nerve passed through the tendinous tunnel formed connectively by triceps brachii, medial intermuscular septum and posterior bundle of UCL and then entered cubital tunnel. Distally, ulnar nerve ran posterior to the common aponeurosis of flexor digitorum superficialis and flexor carpi ulnaris (FCU) arose from anterior bundle of UCL, and ran anterior to FCU tendon originating from olecranon and oblique bundle of UCL. These findings indicate that tendinous structures connecting with UCL form each wall of cubital tunnel, suggesting that UCL injury may also damage walls of cubital tunnel and could bring the risk of ulnar nerve injury. (COI:No)

### OP22-2

**Composition of lower part of the thoracolumbar fascia**

Hirotaka Ishikawa[1], Mizuki Izumida[1], Rintaro Yamamoto[1], Tohma Sakuraya[1], Kenji Emura [3], Takamitsu Arakawa[1] (*[1]Grad. Sch. Health Sci., Kobe Univ., [2]Iwamoto Pain Clinic, [3]Fac. Health Care Sci., Himeji Dokkyo Univ.*)

The lower part of the thoracolumbar fascia, in which myofascial lumbar pain syndrome often occur is still poorly understood. 16 sides of 11 cadavers were macroscopically examined in detail to reveal the composition. In 12 cases, the aponeurosis for the origin of the transversus abdominis fused with the insertion of the internal oblique and the origin of the latissimus dorsi. This fused aponeurosis blended to the thoracolumbar fascia. In 4 cases, no participation of the latissimus dorsi to the fused aponeurosis was observed. At the iliac crest, the aponeurosis by which covered the gluteus medius ran medially along the iliac crest and merged to the thoracolumbar fascia in all cases. These results indicate that the composition of the lower part of the thoracolumbar fascia consistently consists of the aponeurosis of the transversus abdominis, the internal oblique, and the gluteus medius, suggesting that these participants may be involved with myofascial lumbar pain syndrome. (COI:No)

### OP22-3

**Gross anatomical and image analytic approaches to layered facial fasciae and muscles**

Hisako Takami[1], Takafumi Hayashi[2], Noboru Sato[1], Hayato Ohshima[3] (*[1]Div Gross Anat, Niigata Univ Grad Sch Med & Dent Sch, [2]Div Oral Maxillofac Radiol, Niigata Univ Grad Sch Med & Dent Sch, [3]Div Anat Cell Biol Hard Tissue, Niigata Univ Grad Sch Med & Dent Sch*)

**Background and purpose:** We have demonstrated layered facial fasciae and muscles by gross anatomical and image analytical approaches. However, the relationship between the findings in the dissection and the structures recognizable in the images remains to be fully elucidated. The present study aimed to correlate the morphological evidence in the cadaver with the images obtained from computed tomography (CT) and ultrasonography.
**Materials and methods:** We dissected 17 cadavers at the gross anatomical course of Niigata University (2019-2020), and compared the gross anatomical findings with the images in medical CT and ultrasonography. Furthermore, we prepared face-off samples.
**Results and discussion:** The three-layered fasciae in dissection coincided with the images in CT and ultrasonography in the temporal-malar region. The middle fascia covering the parotid gland elongated toward the neck region, and connected with the superficial layer of deep cervical fascia ensheathing the trapezius and sternocleidomastoid muscles. Thus, elucidation of the facial fascia contributes to understanding the expansion of inflammation in the facial region. (COI:No)

### OP22-4

**Morphological analysis of the posterior part of the joint capsule of the temporomandibular joint**

Keiko Fukino[1], Masahiro Tsutsumi[2], Takashi Ono[1], Keiichi Akita[2] (*[1]Department of Orthodontic sciences, TMDU, [2]Department of Clinical anatomy, TMDU*)

Anterior displacement of the articular disc is one of the main symptoms of temporomandibular joint disorders. However, the supporting mechanisms of the articular disc remain unclear. This study aimed to examine the morphological features of the posterior part of the joint capsule including its anatomical relation to the surrounding masticatory muscles. We evaluated three halves three heads from Japanese cadavers. The masseter and temporalis partly originated from the masseteric and temporal fasciae, respectively. Masseteric fascia extended posteriorly, and widely covered with the posterior surface of the joint capsule. Besides, a part of temporalis originated from the temporalis fascia. The posterior part of the temporal fascia descended at the base of the zygomatic process and also widely covered with the posterior surface of the joint capsule. Both temporal and masseteric fasciae adjoined to the joint capsule. These findings suggested that the contraction of the masseter and temporalis can prevent the excessive anterior movement of the articular disc via the joint capsule complex including the temporal and masseteric fasciae. (COI:No)

### OP22-5

**Positional relationship between the lateral border of the Denonvilliers' fascia and pelvic plexus**

Meienn Ka[1], Satoru Muro[1], Keiichi Akita[1] (*[1]Department of Clinical Anatomy, TMDU*)

Denonvilliers' fascia (DVF) is important as a landmark of dissection layer during prostate or rectal surgeries. However, there has few reports about how DVF laterally extends. In this study, we investigated the lateral extent and position of the lateral border of the DVF in macroscopic examination using 12 pelvic halves from 8 cadavers. The lateral margin of DVF attached to the pelvic plexus (PP). The origins of nerve branches from PP to pelvic organs except the rectum were basically located anterior or anterosuperior to the lateral border of DVF. The origins of nerve branches to the prostate were mainly anterior to the lateral border of DVF, but in 3/12 pelvic halves, the nerves origins were also observed posteroinferiorly to the lateral border of DVF. The attachment point of DVF to prostate was more superior in these 3 pelvic halves (the distance from the top point of posterior surface of prostate to the attachment point: 5.6±1.9mm) than that in other 9 pelvic halves (10.1±3.6mm). The lateral border of DVF is closely related with PP, and can be also used as a landmark to identify nerve branches from PP to pelvic organs. (COI:No)

### Oral Presentation23

### Cartilage, Bone, Connective tissue①

(March 30, Tue. 9：00~9：48, Room10)

### OP23-1

**Mouse *Fgf9*$^{N143T}$ leads to cartilage widening, resulting in widened long bones**

Masayo Harada[1], Keiichi Akita[1] ([1]*Department of Clinical Anatomy, Graduate School of Medical and Dental Sciences, Tokyo Medical and Dental University*)

Long bones of the appendicular skeleton are formed through endochondral ossification. Endochondral bone formation initiates with mesenchymal condensation, followed by the formation of a cartilage template which is replaced by bone. Fibroblast growth factor 9 (FGF9) regulates bone development. *Fgf9* $^{-/-}$ mice exhibit disproportionate shortening of proximal skeletal elements. *Fgf9* missense mutations in mice and humans induce joint synostosis. Thus, FGF9 is critical for regulating bone length and joint formation. Conversely, mechanisms regulating bone width remain unclear. Here, we showed that the homozygous elbow knee synostosis (Eks) mutant mice harboring N143T mutation in *Fgf9* have wide long bones at birth. We investigated the cellular and molecular mechanisms underlying the widened prospective humerus in *Fgf9* $^{Eks/Eks}$ embryos. Increased and expanded FGF signaling during chondrogenic condensation of the humerus led to increased density of chondrocytes and widened cartilage, generating wider prospective humeri in neonatal *Fgf9* $^{Eks/Eks}$ mice. Our study suggests that FGF9 regulates the width of prospective long bones by controlling the width of chondrogenic condensation. (COI:No)

### OP23-2

**Reduced calcification in femoral epiphyses of mice over-expressing tissue non-specific alkaline phosphatase driven by type II collagen promoter**

Tomoka Hasegawa[1], Tomomaya Yamamoto[1,2], Hiromi Hongo[1], Toshihisa Komori[3], Norio Amizuka[1] ([1]*Developmental Biology of Hard Tissue, Faculty of Dental Medicine, Hokkaido University,* [2]*Northern Army Medical Unit, Camp Makomanai, Japan Ground Self-Defense Forces,* [3]*Basic and Translational Research Center for Hard Tissue Disease, Graduate school Biomedical Sciences, Nagasaki University*)

During endochondral ossification, tissue non-specific alkaline phosphatase (TNAP) plays a pivotal role for the formation and growth of calcium phosphate crystals. In order to examine if over-expression of TNAP could give rise to premature calcification in cartilage, we have generated type II collagen-driven TNAP transgenic (Tg) mice, and examined the epiphyseal cartilage. TNAP Tg mice at E18 showed slightly shortened femoral epiphyses compared to wild-type littermates. The resting and proliferative chondrocytes of Tg mice revealed an intense TNAP positivity. Matrix vesicles were localized around the chondrocytes, however, the mineral crystals were not present in the matrix vesicles. Hypertrophic zone of the Tg epiphyseal cartilage revealed less numbers of calcified nodules, which were composed of fine needle-like crystals showing faint electron density, compared with wild-type littermates. In addition, the hypertrophic chondrocytes showed an intense immunoreactivity of ENPP1, which inhibits calcification by producing pyrophosphates. Summarizing, it is likely that TNAP over-expression would facilitate ENPP1 synthesis, resulting in inhibition of calcification in cartilage. (COI:No)

### OP23-3

**The p53 deficiency promotes bone defect healing by enhancing the function of mesenchymal cells.**

Tadashi Ninomiya[1], Toshimichi Nagashima[3], Yoshiki Nakamura[4], Shirabe Nishimura[4], Tomihisa Takahashi[1,2] ([1]*Department of Anatomy, Nihon University School of Dentistry,* [2]*Dental Research Center, Nihon University School of Dentistry,* [3]*Department of Oral and Maxillofacial Surgery, Nihon University School of Dentistry,* [4]*Department of Orthodontics, Nihon University School of Dentistry*)

Following a bone fracture, mesenchymal stem cells are recruited to the injured area, proliferate to increase cell number, and then, differentiated into osteoblasts that repair bone tissue. The transcription factor p53, which plays a pivotal role in cell cycle and cell death, suppresses osteoblast differentiation through inhibition of osterix expression, however, the role of p53 in fracture healing has not been fully elucidated.

In this study, we investigated the effects of p53 in bone repair process. Bone marrow-derived mesenchymal stromal cells (MSCs) and calvaria-derived osteoblasts were collected from p53-deficiency (KO) mice and examined for abilities of cell motility, cell proliferation, and wound healing. KO cells showed higher potency for all functions compared with cells from wild type (WT) mice. In osteoblast differentiation by addition of BMP-2, KO-MSCs showed higher ability than WT-MSCs. Moreover, a bone defect was created in the femur of mice and its repair was observed. KO mice had higher defect healing ability than WT mice. This study suggests that p53 deficiency promotes the healing of bone defect by enhancing the function of mesenchymal cells. (COI:No)

### OP23-4

**The effect of Transient receptor potential-vanilloid 4 (TRPV4) deletion on endochondral ossification**

Kaho Uchino[1], Reiko Yoshimoto[1], Megumi Nishiyama[1], Takeshi Sawada[1], Weiqi Gao[1], Ailin Cao[1], Yuko Honda[1], Mizuho Kido[1] ([1]*Div. Histol. Neuroanat., Fac. Med., Saga Univ.,* [2]*JSPS Research Fellow*)

Transient receptor potential-vanilloid 4 (TRPV4) is known as a calcium permeable cation channel activated by hypo-osmolarity, temperatures, or mechanical stimulation. Human TRPV4 gene mutations have been identified in skeletal dysplasia. However, the mechanisms of TRPV4 involvement in ossification remain unclear. In this study, we compared the process of endochondral ossification of wild-type (WT) mice with TRPV4-deficient (TRPV4KO) mice at 3- and 8-week-old. Under micro-CT observations, the length of metatarsal bone was significantly shorter in TRPV4KO mice than that in WT mice. The length of tibial growth plate was significantly shorter in TRPV4KO mice than that in WT mice. Immunoreactivities for beta-catenin and osterix were stronger in the nucleus of hypertrophic chondrocytes in TRPV4KO mice than that in WT mice. The expressions of type I and type II collagen were significantly less in TRPV4KO mice than those in WT mice. These results suggested that TRPV4 regulated chondrocyte differentiation process and matrix generation then affected endochondral ossification. (COI:No)

### Oral Presentation24

### Gross anatomy②

(March 30, Tue. 10：05～11：05, Room9)

### OP24-1

**Morphological study of lumbar vertebra using X-ray in vivo (with age, gender and level)**

Yoshihisa Otsuka[1], Akiyo Otsuka[1,3], Nobuyuki Suzuki[2], Seiji Otsuka[5], Masahito Yoshida[2], Hiroaki Fukushima[2], Yoshihiko Otsuka[1] (*[1]Otsuka Orthopaedic Clinic, [2]Nagoya City University, Department of Orthopaedic Surgery, [3]Fujita Health University, Department of Orthopaedic Surgery, [4]Tokai College of Medical Sciences, Occupational Therapy and Physical Therapy, [5]Toyokawa City Hospital, Department of Orthopaedic Surgery Spine Center*)

(PURPOSE/METHOD)
Spinal vertebrae have role of weight bearing through vertical direction. Humans suffer various vertebral originated disorders.
206 patients (107 males and 99 females, age 20~69) underwent X-ray photos. Angle of vertebrae were calculated and analyzed by sex and ages.
(RESULT)
The vertebra-angles in their 20's 30's 40's 50's and 60's were -0.66°± 0.3,0.17°±0.2, -0.25°± 0.3,-0.24°± 0.3, -0.27° ± 0.4 respectively. ($p > 0.05$) The vertebra-angles were 0.4° ± 0.2 (male), and -1.1° ± 0.2 (female).($p < 0.01$). The vertebra-angles at L1, L2, L3, L4 and L5 were 3.8°± 0.2, 1.9°± 0.2, 0.3°± 0.2, -1.7°± 0.2, -5.9°± 0.2, respectively. Angles decreased with caudal level up to L5 ($p < 0.01$). Gender analyses were divided by vertebra level and age respectively.
(DISCUSSION)
Vertebra angles decreased with caudal level up to L5. L1 and L2 has anterior tilted angle, L3 and L4 has flat shape and otherwise L5 has posterior tilted angle. Murakami H. et al. analyzed biomechanical pressure to vertebrae due to alignment. This time anatomical shape difference of vertebra itself was detected and suggested to have relationship add to biomechanical stress and fracture type. (COI:No)

### OP24-2

**How to convert skulls and brains into 3D data inexpensively, easily, safely and with high accuracy**

Hiroshi Kikuchi[1], Atsushi Yamaguchi[1], Tatsuya Jitsuishi[1], Takashi Hozumi[1], Osamu Sawai[1] (*[1]Chiba Univ*)

Detailed structures of the body can be obtained by using Computed Topography and Magnetic Resonance images. However, such devices are expensive. If there is a device that can acquire the structure with the accuracy that can be acquired in the latest medical field and at a low budget, it will bring useful information in anatomical field. We propose a method to convert a replica skull and brain (replica) into 3D data with a 3D scanner. A commercially available replica was used. SOL software manufactured by Scan Dimension and a computer with windows 10 (The PC) were used. Specimen was scanned by The Scanner. We were able to convert replicas into 3D data. The cost of The Scanner was € 699. The scan was performed by placing the sample on the table of The Scanner, and all other work was done automatically. In the 3D data, the color tone was expressed as if it were real, and the suture lines of the skull were also depicted in detail, so the accuracy of at least 1 mm or more was maintained. By using "SOL 3D SCANNER", replicas can be converted into 3D data inexpensively, easily, safely and with high accuracy. It seems that this method can be applied to the actual skull and brain (COI:Properly Declared)

### OP24-3

**An unusual case of innervation of the anterior belly of the digastric muscle by the hypoglossal nerve**

Ikuo Kageyama[1], Ryuhei Kojima[2], Kojiro Takezawa[1], Katsuji Kumaki[1] (*[1]Dept. of Anatomy, Sch. of Life Dent Niigata, The Nippon Dental Univ., [2]Dept. of physical therapy, Fac. of Health and Medical Care, Saitama Med. Univ.*)

We observed the anterior belly of the digastric muscle innervated by the hypoglossal nerve at the right side of the neck. This was found on the right side of the cadaver of a 79- year-old male in the 10th macroscopic seminar. It was held at the Nippon Dental University at Niigata from August 8th to 20th, 2016. The hypoglossal nerve generally originates from the occipital somite and innervates the tongue muscles except for the palatoglossal muscle. In most cases, the anterior belly of the digastric muscle is innervated by the mylohyoid nerve which has a first branchial arch origin. In rare cases, the hypoglossal nerve innervates the sternocleidomastoid muscle (Koizumi *et al.* 1993). However, in this unusual case, the branch of the hypoglossal nerve was distributed into the anterior belly of the digastric muscle. It was exhibited completely different from that of previous observed. According to Quain's Anatomy (1960), the hypoglossal nerve innervates to the mylohyoid, the digastric, and the stylohyoid muscles in very rare cases. The correlation between the muscle and nerve development in the occipital somite and branchial arches should be elucidated for further study. (COI:No)

### OP24-4

**Rare case report of the excessive stylohyoid muscle**

Ming Zhou[1], Akimitsu Ishizawa[2], Hideo Akashi[1], Yoshio Bando[1] (*[1]Akita Univ. Grad. Sch. Med., [2]Sci. Edu. Sup. Sivis. Kashiwa City*)

An excessive stylohyoid muscle and stylohyoid ligament was observed on the left side of a Japanese cadaver. The excessive styloid muscle is located on the lower medial aspect of the styloid process and distal to the styloid muscle, and is generated as two origins by the tip of the styloid process. It descended to the area between the stylohyoid muscle and the external carotid artery inside the stylohyoid muscle and stopped at the lesser horn of the hyoid bone. On the other hand, a small branch of this muscle descended along the outside of the stylopharyngeus, passed through the lateral edge of the middle pharyngeal constrictor muscle, and stopped at the lower part of the greater horn of the hyoid bone. The stylohyoid ligament originates from the tip of the styloid process and divided into two parts. One was going backwards ended up spreading near the large angle of the hyoid bone. The anterior one spread along the lower edge of the mylohyoid.
The excessive stylohyoid muscle was considered to have the action as the medial muscle when it was sandwiched between the stylohyoid and the digastric tendon. In addition, it was thought to influence the actions of the hyoid pharyngeal muscles. (COI:No)

### OP24-5

**Morphological changes in infrapatellar fat pad associated with anterior cruciate ligament injury: Ultrasonographic and quantitative dynamic assessment on Thiel embalmed cadaver.**

Yoshiyuki Tokuda[1], Tsuneo Nakamura[1], Yoshitake Shiraishi[1], Tatsuya Ishikawa[1], Kiyomi Hori[1], Hiroaki Okuda[1], Noriyuki Ozaki[1] (*[1]Kanazawa Univ. Grad. Sch. Department of Functional Anatomy*)

Methods: Thiel embalmed cadavers donated to Kanazawa University for research and educational purposes were used in this study (8 knees of 5 cadavers; 5 females; average age: 93.4 years). This study was approved by ethics committee of Kanazawa University Graduate School of Medicine (No. 2882).
Gem-clips were inserted into the infrapatellar fat pad (IPFP) of Thiel embalmed cadaver under ultrasonography (US). Location of the clips were investigated with knees flexion at 0, 30, 60, and 90-degrees, and displacement of the clips between each angle were measured. IPFP of control group, sham group, and ACL-tear (ACL-T) group, were investigated, and US images were analyzed by ImageJ and SPSS statistic software.
Results: On the anterior-posterior direction, displacement of IPFP between knee flexion at 60 and 90 degrees in ACL-T group significantly decreased compared to control and sham groups ($p < 0.001$).
Conclusion: Dynamic ultrasonographic assessment of IPFP is an auxiliary diagnostic method for ACL-T. IPFP might be a functional indicator of knee joint.
The authors declare no conflicts of interest associated with this manuscript. (COI:Properly Declared)

### Oral Presentation25

### Cartilage, Bone, Connective tissue②

(March 30, Tue. 9：53～10：29, Room10)

### OP25-1

#### The role of O-GlcNAc transferase in osteoblast differentiation

Yao Weng[1], Airi Tanai[1], Yoko Fukuhara[1], Mika Ikegame[1], Hirohiko Okamura[1] ([1] *Grad. Sch. Med, Dent, Pahrma, Okayama Univ.*)

**Background:** Protein glycosylation plays various roles in regulating numerous cellular events. Among them, O-GlcNAcylation adds and removes N-acetylglucosamine (GlcNAc) to serine and threonine residues of proteins by two enzymes: O-GlcNAc transferase (OGT) and O-GlcNAcase (OGA). In this study, we investigated the role of OGT in osteoblast differentiation.
**Methods:** Subcellular localization of OGT was examined by immunostaining in mouse preosteoblastic MC3T3-E1 cells and in mouse calvarial sections. The proliferation rate was investigated in OGT-knockdown cells by cell counting. The effect of OGT knockdown on osteoblast differentiation and the expression of bone markers were examined by ALP and Alizarin red staining, qPCR, and Western blotting.
**Results:** OGT was mainly localized in nucleus of osteoblasts. OGT knockdown decreased the proliferation of MC3T3-E1 cells. Knockdown of OGT also resulted in the decreased intensity of ALP and Alizarin red staining. The expression of bone markers were lower in OGT-knockdown cells than those of control cells.
**Conclusion:** These results suggest that OGT regulates the ability to differentiate and mineralize in osteoblasts. (COI:No)

### OP25-2

#### Study of age-related change in femur left-right differences in rats

Uetake Shunichi[1], Toru Negishi[3], Zhang Ming-Shou[1], Shuang-Qin Yi[1] ([1] *Dept. Frontier Health Sciences, Grad. Sch. Human Health Sciences, Tokyo Metropolitan Univ.*, [2] *Dept. Physical Therapy, Touto Rehabilitation College*, [3] *Dept. Radiological Sciences, Grad. Sch. Human Health Sciences, Tokyo Metropolitan Univ.*)

**Background:** Body left-right differences is determined in the early stage of embryonic development. In this study, it was analyzed whether there are left-right differences in the femur with growth in rats.
**Methods:** Eight week-old male Sprague-Dawley rats (n=6) were employed to measure the growth of the femur at aging. Photograph the femurs with mammography was taken every 8 weeks until 48$^{th}$ weeks. Both hind legs of rats were photographed in the posture of 90°hip flection, maximum abduction and internal-rotation under anesthesia.
**Results:** The total length of the femur and the length of the lesser trochanter to tip of condyle were significantly longer on the left side than on the right side at all ages ($p$<0.05). The width of femoral head was significantly longer on the left side than on the right side at 24$^{th}$, 32$^{th}$ and 48$^{th}$ week ages ($p$<0.05). Medial-lateral condyle width was not significantly different at all week ages.
**Conclusions:** These results suggested that there are already left-right differences in major axis direction of femur at the initial stage. It is considered that effect for cartilaginous ossification is involved in long axis direction of the femur. (COI:No)

### OP25-3

#### Inducible nitric oxide synthase pathways promoted osteoclast differentiation under hypoxia.

Takao Kondo[1], Yuto Ostuka[1], Narumi Masukawa[1], Hiromasa Aoki[1], Yo Goto[2], Ken Miyazawa[2], Shigemi Goto[2], Mineyoshi Aoyama[1] ([1] *Depertment of Pathobiology,Graduate Scool of Pharmaceutical Sciences, Nagoya Ctiy University*, [2] *Department of Orthodontics, School of Dentistry, Aichi-Gakuin University*)

Purpose: Our previous studies showed that osteoclast formation was promoted under hypoxia. However, the precise mechanism of osteoclast formation under hypoxia has not been elucidated. In the present study, we investigated the role of inducible nitric oxide synthase (iNOS) on osteoclast differentiation under hypoxia.
Method: Bone marrow cells obtained from mice were stimulated with receptor activator of NF-kappa B ligand (RANKL) and macrophage colony stimulating factor (M-CSF) to induce osteoclast differentiation. Hypoxia (oxygen concentration 5%), iNOS inhibitor, or NO (nitric oxide) donor was used as stimuli during the culture.
Results and Discussion: The number of osteoclasts was increased in the culture under hypoxia compared with that in the culture under normoxia (oxygen concentration 20%). The gene and protein expression of iNOS increased in the culture under hypoxia. The addition of iNOS inhibitor in hypoxic culture reduced the number of osteoclasts. When NO donors were added in normoxic culture, the number of osteoclasts were increased. These results suggested that hypoxic condition could promoted osteoclast differentiation due to iNOS pathway. (COI:No)

### Oral Presentation26

### Higher brain function, etc.

(March 30, Tue. 14：20～15：32, Room8)

### OP26-1

**Enhancement of contextual memory by cross-modal distraction of tones**

Nozomu Nakamura[1], Lukas Beichert[1], Yoshitaka Oku[1] (*[1]Dept Physiol, Hyogo Col Med*)

Animals have a tremendous ability to discriminate contextual information (e.g., Morris water maze and hippocampal place cell firing). However, it remains unclear about the refinement of contextual processing under multiple sensory modalities. To understand cross-modal refinement of visual information regarding cognitive performance, we employed a fear conditioning paradigm with a pair of conditioned stimulus (CS+) and conditioned inhibition of fear (CS-) in mice competing between visual and auditory cues. Animals were deliberately tested to discriminate between spatial contexts (black and white walls) and tones (high and low tones) for a cross-modal paradigm. We found that contextual safety CS was superior to tone fear CS when those were exposed at the same time, indicating that contextual memory overrode tone memory. Importantly, a mixture of the contextual memory with tone distraction further superseded object memory (i.e., the acrylic box). Meanwhile, when the tones disappeared, object memory kept being more predominant than contextual memory. We suggest that contextual target information was enhanced by congruent tone distraction that can alter cognitive performance. (COI:No)

### OP26-2

**Contrasting roles of spinal and cortical premotor neurons for control of grasping in primates**

Tomohiko Takei[1], Otomichi Oya[2,3], Kazuhiko Seki[2,3,4] (*[1]Dept. of Neurobiol/ Hakubi Cent., Kyoto Univ., [2]Dept. of Neurophysiol., Natl. Inst. of Neurosci., [3]Dept. of Developmental Physiol., Natl. Inst. for Physiological Sci., [4]PRESTO, JST*)

Corticomotoneuronal (CM) cells and spinal premotor interneurons (PreM-INs) are two major input sources to primate hand motoneurons, but their functional difference in the control of dexterous hand movement remains unclear. Here we explored their functional difference of two groups by examining their post-spike effects (PSEs) on hand muscle activity. Single-unit activities were recorded in the spinal cord (C6 – T1) or primary motor cortex (M1) simultaneously with 20 electromyographyic signals (EMGs) from hand and arm muscles, while monkeys performed a precision grip task. Spike-triggered averaging of rectified EMGs identified 20 PreM-INs and 21 CM cells. The majority of PreM-INs (14/20) produced PSEs on more than one muscle, and the number of muscles with PSEs (muscle field size) was 3.0 ± 2.2. In contrast, a smaller proportion of CM cells produced PSEs on more than one muscle (9/21) and their muscle field size was significantly smaller than those of the PreM-IN (1.8 ± 1.0). These results highlight the relative functions of the two neural systems: PreM-INs coordinate a larger number of muscles, whereas CM cells provide more selective control to enable dexterous hand movements. (COI:No)

### OP26-3

**Event-related desynchronization during swallowing in healthy adults**

Satoko Koganemaru[1], Fumiya Mizuno[5], Toshimitsu Takahashi[1], Yu Takemura[2], Hiroshi Irisawa[2], Takashi Mizushima[2], Masao Matsuhashi[3], Tatsuya Mima[4], Kenji Kansaku[1] (*[1]Department of Physiology, Dokkyo Medical University, [2]Department of Rehabilitation Medicine, Dokkyo Medical University, [3]Department of Epilepsy, Movement Disorders and Physiology Graduate School of Medicine, Kyoto University, [4]The Graduate School of Core Ethics and Frontier Sciences, Ritsumeikan University, [5]Department of Rehabilitation Medicine, Dokkyo Medical University Hospital*)

Background: Swallowing-related brain activity has been observed by brain imaging methods. Magnetoencephalography (MEG) has reported swallowing-related desynchronization (ERD) on the oropharyngeal region of the sensorimotor cortex. This indicates that the cerebral cortex is involved in swallowing movement. In EEG, an event-related potential has been reported for brain activity, but ERD has not yet been reported. If ERD can be measured by EEG, it will be possible to more easily evaluate the central nervous system related to swallowing.
Object: We investigated whether ERD was observed during swallowing in healthy subjects using EEG.
Methods: Brain activity of eighteen healthy adults without dysphagia were recorded during spontaneous swallowing of water by using a 30-ch electroencephalograph (Sampling rate: 1000 Hz). The power spectra at rest and during swallowing were calculated, and the ERD was measured.
Results: ERD was observed on the sensorimotor area and motor-related area during swallowing.
Discussion: ERD could be observed in EEG, which is similar to previous MEG studies. In the future, we plan to investigate the swallowing-related brain activity by EEG in patients with dysphagia. (COI:No)

### OP26-4

**Cortical contributions to the anticipatory postural adjustments in dart throwing**

Amiri Matsumoto[1], Nan Liang[1], Hajime Ueda[1], Keisuke Irie[1] (*[1]Cognitive Motor Neuroscience, Department of Human Health Sciences, Graduate School of Medicine, Kyoto University*)

It remains unclear whether the changes in the corticospinal excitability contribute to the anticipatory postural adjustment (APA) in the lower leg when performing a ballistic movement of upper limb. In this study, we examined the excitability of the primary motor cortex (M1) during the APA by using transcranial magnetic stimulation (TMS). Healthy volunteers were asked to perform dart throwing with the dominant hand with a visual cue. Electromyography (EMG) was recorded from the ipsilateral anterior deltoid, triceps brachii (TB), tibialis anterior (TA), and soleus muscles. Because the EMG of TA muscle was markedly observed prior to the EMG of TB muscle (agonist), we applied TMS over the hotspot of TA muscle in the contralateral M1 at 0 to 100 ms prior to the EMG onset of TA muscle. Compared with control, the motor evoked potential (MEP) amplitude in the TA gradually increased prior to the movement, especially at the time point immediately before the onset of TA muscle. MEP/B.EMG results suggested a cortical contribution to the APA. The results of kinematic parameters from the three-dimensional analysis and the displacement of center of pressure will also be discussed. (COI:No)

### OP26-5

**Effects of stress on corticosterone in urine and adaptive motor learning in the VOR in mice**

Akira Katoh[1], Aiko Yamagiwa[2] (*[1]Dept Physiol, Tokai Univ Sch Med, [2]Grad Sch Biol Sci, Nara Inst Sci and Tech*)

Under stressful conditions, the endocrine system is activated, resulting in changes in the physiological status and behavior. However, mechanisms how stress affects motor performance are not clear. Here we examined the influence of stresses to adaptive changes in the vestibulo-ocular reflex (VOR) in mice. First, using C57BL/6 male mice, we found the concentration of the corticosterone increased in urine after restrained or co-housing with another non-littermate male mouse. We also found impaired motor learning in the VOR after restrained or co-housing stress. Using mice expressing the archaerhodopsin T selectively in the corticotropine releasing factor (CRF)-positive cells, we illuminated around the paraventricular nucleus (PVN) with 525 nm LED to inactivate CRF-positive neurons in the PVN during restrained stress and whole training period for the VOR motor learning, and found adaptive changes in the VOR recovered. Taken together with our previous report that CRF local administration to the cerebellum enhanced motor learning in the rotarod, our results suggest differential roles of CRF in the PVN and the cerebellum for motor learning under stress exposure. (COI:No)

### OP26-6

**Histamine receptor *Hrh1* independent action of the anti-histamines on the cerebral microcirculation.**

Tomokazu Ohshiro[1], Takeo Yoshikawa[2], Hajime Mushiake[1] (*[1]Department of Physiology, School of Medicine, Tohoku university, Japan, [2]Department of Pharmacology, School of Medicine, Tohoku university, Japan*)

Rhythmic oscillations of the brain activity at very slow ranges (0.1Hz or less) are known as infra-slow oscillation, whose origin has not been well understood. We have found that the slow oscillation reflects the cerebral vasomotion, a slow rhythmical oscillation of the cerebral arteries in their vessel wall diameter. To understand the mechanism underlying the rhythm generation, we screened biologically active molecules in mice and rats, and identified Histamine, which strongly dilated the arteries and halted the on-going vasomotion *in vivo*. Classical anti-histamines against histamine receptor *Hrh1* such as Ketotifen, oppositely, constricted the arteries. Intravenous infusion of Mepyramine which crosses the Brain-Blood-Barrier (BBB), halted the cerebral vasomotion, whereas, Cetirizine which cannot, showed a negligible effect, suggesting a central action of these anti-histamines. Surprisingly, *Hrh1* knockout mice exhibit seemingly normal cerebral vasomotion. However, intravenous infusion of Mepyramine strongly attenuated the arterial vasomotion in *Hrh1* knockout mice, suggesting an unidentified receptor sensitive to antihistamines responsible for the cerebral vasomotion generation. (COI:No)

### Oral Presentation27

### Sensory function, Sensory organ

(March 30, Tue. 14：20～15：32, Room9)

### OP27-1

**Generation of a medaka model for inherited retinal disease by Crispr/Cas9 mediated genome editing**

Keita Sato[1], Yang Liu[1], Takahiro Yamashita[2], Hideyo Ohuchi[1] (*[1]Grad. Sch. of Med., Dent. and Pharm. Sci., Okayama Univ., [2]Grad. Sch. of Sci., Kyoto Univ.*)

Inherited retinal dystrophies (IRDs) such as retinitis pigmentosa (RP) cause progressive photoreceptor degeneration in the retina, leading to vision loss or eventual blindness. Although substantial efforts have been made to establish therapeutics for IRDs, effective therapies for IRDs have been quite limited. One reason for this would be the considerable genetic and phenotypic heterogeneity. Understanding detailed molecular pathology of each IRD is crucial to develop treatments. *Eyes shut homolog* (*EYS*) is one of the most prevalent causal genes for autosomal recessive RP in Japanese, Asian and European populations. Despite the need for elucidating molecular functions of EYS, the information remains insufficient as the genomes of mouse and other mammals lack *Eys*. In this study, we have chosen medaka fish (*Oryzias latipes*) as a model animal to investigate physiological and pathological roles of *eys*. We have generated an *eys*-knockout medaka by CRISPR/Cas9 technology. *eys*$^{-/-}$ medaka showed mislocalization and decreased expression of visual phototransduction components. We discuss possible functions of *eys* in the retina based on the histological and molecular analysis of the medaka mutant. (COI:No)

### OP27-2

**Noradrenergic modulation of visual detectability via adrenergic β receptor in V1**

Keisuke Tsunoda[1], Akinori Sato[2], Satoshi Shimegi[1] (*[1]CELAS, Osaka Univ, Osaka, [2]Dept. Basic Med. Sci., Nagoya Univ., Aichi*)

Noradrenaline (NA) is secreted from locus coleuses depending on behavioral context, and modulates various brain functions, including vision. Previous electrophysiological studies in primary visual cortex (V1) revealed that iontophoretically-administered NA facilitates or inhibits the visual responses in a dose-dependent manner, and this bi-directional modulatory effect is achieved by the activation of specific adrenoreceptor subtype. However, it has been unclear whether and how noradrenergic response modulation of V1 neurons affects perceptual visual detectability. In order to investigate this point, we performed multi-unit extracellular recordings from V1 of the rats performing the visual detection task, and examined the relationship between task performance and neural activity, and then, tested the effect of adrenergic beta-receptor antagonist, propranolol (PRP). PRP administration significantly improved perceptual visual detectability for low contrast stimuli, and also improved the neuronal signal-to-noise ratio in V1. Therefore, NA might reduce animal's visual ability to detect stimuli at low contrast conditions via beta-receptor in V1. (COI:No)

### OP27-3

**Nicotinic regulation of spatiotemporal and intensity changes of tone-evoked flavin autofluorescence responses in mouse auditory cortex**

Makoto Nakanishi[1], Hideki Kawai[1] (*[1]Department of Biosciences, Graduate School of Science and Engineering, Soka University*)

Nicotine is known to enhance sound evoked cortical responses in humans and rodents. How nicotine exposure regulates sound-evoked cortical processing in subfields of auditory cortex (ACx) remains to be investigated. Here, we examined spatiotemporal and intensity changes of flavin autofluorescence (FA) in ACx of anesthetized mice. FA responses were recorded in response to AM tones. Nicotine (2 mg/kg, free base) was injected intraperitoneally. To block nicotinic receptors containing α4 and β2 subunits (α4β2*-nAChRs), dihydro-β-erythroidine (DHβE, 10 μM) was injected locally dorsal to tone activated area. AM tones increased FA in a wide area of ACx, including primary ACx (AI), anterior auditory field (AAF), and secondary ACx (AII). Nicotine exposure enhanced FA intensity within 500 ms of tone onset in the 3 cortical subfields without affecting activated area size. DHβE injection blocked these enhancement. These results indicate that α4β2*-nAChR activation enhances tone-evoked neuronal activities in not only AI but also AAF and AII. (COI:No)

### OP27-4

**Electrochemical properties in epithelial-like tissue stria vascularis of mammalian cochlea are sensitive to sounds**

Qi Zhang[1], Takeru Ota[1], Takamasa Yoshida[3], Mitsuo Sato P.[4], Katsumi Doi[4], Arata Horii[2], Fumiaki Nin[1], Hiroshi Hibino[1,5] (*[1]Department of Molecular Physiology, Niigata University School of Medicine, [2]Department of Otolaryngology, Niigata University School of Medicine, [3]Department of Otorhinolaryngology, Graduate School of Medical Sciences, Kyushu University, [4]Department of Otolaryngology, Kindai University Faculty of Medicine, [5]AMED-CREST, AMED*)

The cochlea in inner ear transduces acoustic pressure to electrical signals. [$K^+$]-enriched cochlear endolymph exhibits +80 mV. This endocochlear potential (EP), which accelerates vibration-induced $K^+$-entry to sensory hair cells, is maintained by $K^+$ transport mechanisms in stria vascularis. An extracellular compartment inside the stria, the intrastrial space (IS), showed a low [$K^+$] and a positive potential similar to the EP. This IS potential underlies the EP and is accounted for by $E_K$ in the stria. Whether and how the stria responds to acoustic stimuli remains obscure. We targeted the portion sensitive to 1 kHz in live guinea pigs and examined the IS with microelectrodes that measures [$K^+$] and potential. Imposition of 1-kHz sound at 90 dB upon the animals caused IS potential to decrease and [$K^+$] to increase; the change of the potential match that of $E_K$. Moreover, the amplitudes of the measured responses depended on the stimulus intensities and frequencies. These observations indicate that the electrochemical properties of the stria vascularis are sensitive to acoustic stimuli. Theoretically, this phenomenon likely stems from coupling of the stria to hair cells by a loop current. (COI:No)

### OP27-5

**Neuropeptide Manserin Localized in the Dorsal Root Ganglion in Pain Function**

Michiru Eto[1], Takeshi Ohkawara[1], Masaaki Narita[1] (*[1]Grad. Sch. Med., Mie Univ.*)

Manserin is a neuropeptide discovered from rat brain and is localized in the neuroendocrine system. We previously reported manserin distribution in the spiral ganglion, suggesting that it is also localized in other ganglia. Here, we investigated the manserin immunoreactivity in the dorsal root ganglion (DRG) and spinal cord of the adult Wistar rat. DRG consists of large cells and 2 types of small cells (CGRP-positive peptide neurons and CGRP-negative non-peptide neurons). Manserin immunoreactivity was observed as dots in the cytoplasm of the small cells. Double immunostaining showed that the manserin-positive cells corresponded to some of the CGRP-positive small cells. The DRG is projects to each layer of the dorsal horn of the spinal cord. Manserin immunoreactivity was observed in the CGRP-positive lamina I and outer lamina II, but not to the isolectin IB4-positive inner lamina II of the dorsal horn. These results suggest that manserin is localized in CGRP-positive cells in the DRG and projects to the dorsal horn of the spinal cord, and is secreted together with other neuropeptides to participate in the nociceptive function. (COI:No)

### OP27-6

**Effect of light isoflurane anesthesia on direction- and orientation-selective functional structures in mouse superior colliculus.**

Masatoshi Kasai[1], Tadashi Isa[1,2] (*[1]Dept Neurosci, Grad Sch Med, Kyoto Univ, [2]ASHBi, Kyoto Univ*)

Superior colliculus (SC) has been gathering greater interest for its role in transformation of bottom-up sensory information to various behavioral outputs, such as orientation, approach, and escape. The SC receives direct visual inputs from retina, which construct the well-ordered retinotopic map in its superficial layer (sSC). Recent imaging studies revealed other types of functional architectures such as direction or orientation selective (DS/OS) cells in line with its retinotopy. But it remains unclear what kind of rules regulate the relationships of these multiple functional architectures. In this study, we found that the light isoflurane increases the number of OS cells in the sSC. The local similarity of their preferred orientation is higher in light isoflurane conditions that the awake state. We next examined the properties of inhibitory sSC cell by expressing GCaMP in a Cre-dependent manner. At the population level, OS pattern of the inhibitory cells did not change by the isoflurane, while the number of DS cells decreased at the population level. From these results, we will discuss the mechanisms of how DS and OS properties are regulated in the sSC. (COI:No)

### Oral Presentation28

### Embryology, Regenerative Medicine, Development, Growth, Aging

(March 30, Tue. 15：37～16：25, Room8)

### OP28-1

### The nephric mesenchyme lineage of intermediate mesoderm is derived from Tbx6-expressing derivatives of neuro-mesodermal progenitors via BMP-dependent Osr1 function

Shinichi Hayashi[1], Masaaki Kitada[1], Tatsuya Takemoto[2] ([1]*Dep. Anat., Facu. Med, Kansai Medical Univ.*, [2]*Development., IAMS., Tokushima Univ.*)

In mouse gastrulation, epiblast cells invaginates through the primitive stream and forms mesoderm and endoderm, whereas a population staying the superficial layer forms ectoderm. In the classical view, this germ layer formation is considered to restrict their own fates. Recently, we have proposed that axial stem cells generates both paraxial mesoderm cells and neural plate cells during body elongation, where these two cell populations are induced by Tbx6 and Sox2, respectively. We found mesodermal cells double-positive for Tbx6 and Osr1, one of metanephric markers of the metanephric mesoderm, suggesting the generation of the intermediate mesoderm, a primordium of nephrons, from axial stem cells. In Tbx6 knockout mice, the metanephric mesoderm was not formed, and the posterior elongation of Wolffian duct (nephric duct) was not observed. Furthermore, we identified BMP4 as an inducer of the intermediate mesoderm from a Tbx6-positive cell population, implying that BMP4 demarcates the border of the intermediate mesoderm from the paraxial mesoderm. These results demonstrate that axial stem cells also generate the intermediate mesoderm in addition to the paraxial mesoderm and neural tube. (COI:No)

### OP28-2

### Maturation of complex synaptic connections of layer 5 cortical axons in the posterior thalamic nucleus requires SNAP25

Shuichi Hayashi[1], Anna Hoerder-Suabedissen[2], Emi Kiyokage[3], Catherine Maclachlan[4], Kazunori Toida[1], Graham Knott[4], Zoltan Molnar[2] ([1]*Department of Anatomy, Kawasaki Medical School*, [2]*Department of Physiology, Anatomy and Genetics, University of Oxford*, [3]*Department of Medical Technology, Kawasaki University of Medical Welfare*, [4]*BioEM Facility, School of Life Sciences, EPFL*)

Synapses are able to form in the absence of neuronal activity, but how is their subsequent maturation affected in the absence of regulated vesicular release? We explored this question using 3D electron microscopy and immuno electron microscopy analyses in the large, complex synapses formed between cortical sensory efferent axons and dendrites in the posterior thalamic nucleus (Po). Using a *Synaptosome Associated Protein 25* conditional knockout (*Snap25* cKO) we found that during the first two postnatal weeks the axonal boutons emerge and increase in the size similar to the control animals. However, by P18, when an adult-like architecture should normally be established, axons were significantly smaller with 3D reconstructions showing that each *Snap25* cKO bouton only forms a single synapse with the connecting dendritic shaft. No excrescences from the dendrites were formed. These results show that activity mediated through regulated vesicular release from the presynaptic terminal is not necessary for the formation of synapses, but it is required for the maturation of the specialised synaptic structures between layer 5 corticothalamic projections in Po. (COI:Properly Declared)

### OP28-3

### Injured motor neurons lose polarity in a proteasome–dependent manner to increase the mitochondrial transport into regenerative axons

Sumiko Kiryu-Seo[1], Reika Matsushita[1], Yoshitaka Tashiro[3], Ryosuke Takahashi[3], Takeshi Yoshimura[4], Yohei Iguchi[2], Masahisa Katsuno[2], Hiroshi Kiyama[1] ([1]*Nagoya Univ, Grad Sch Med, Dept Functional Anat and Neurosci*, [2]*Nagoya Univ, Grad Sch Med, Dept Neurol*, [3]*Kyoto Univ, Grad Sch Med, Dept Neurol*, [4]*Osaka Univ, United Grad Sch Child Dev*)

Injured motor neurons regenerate with a robust induction of stress-responsive transcription factor, ATF3. They share multiple stress responses with pathological ALS motor neurons, caused by proteasomal dysfunction. To examine the proteasome-sensitive stress responses in damaged motor neurons, we have established injury-induced Rpt3 CKO mice in which a subunit of proteasome, Rpt3, is ablated and mitochondria are simultaneously labeled by GFP upon injury by using Atf3 gene regulatory element. Rpt3-deficient injured motor neurons decreased the number of axonal mitochondria, prior to ALS-relevant degeneration. Intriguingly, motor neurons of Rpt3 CKO mice failed to disassemble the axon initial segment (AIS) in response to injury, which localized in the proximal axon and maintained neuronal polarity. The failure of the AIS disassembly restricted mitochondrial entry to the injured axons. Likewise, the ATF3-expressing vulnerable motor neurons of ALS model mice maintained the AIS with reduced axonal mitochondria. Therefore, injury-induced proteasome-dependent AIS remodeling would be beneficial to allow damaged motor neurons to lose polarity and satisfy energy demand for regenerative axons. (COI:No)

### OP28-4

### Analysis of cell behaviors in the pancreatic islets during pancreatic β cells in zebrafish

Hiroki Matsuda[1] ([1]*College pf Life Sicences, Ritsumeikan University*)

Pancreatic β cells, which produce Insulin, play a central role for glucose homeostasis. Regenerative capacity of mammalian β cells is limited, so that loss of β cells causes diabetes. On the other hand, zebrafish have high regenerative capacity of pancreatic islets, including β cell, throughout their entire life. Thus, zebrafish is an attractive model for the study of β cell regeneration. However, it has not yet solved basic questions including when β cell regeneration is completed, and what cells regenerating β cell arise from, and so on. Using several zebrafish transgenic lines, I revealed that zebrafish completed pancreatic β cell regeneration at 14 days after β cell ablation, and that zebrafish pancreas undergoes two-step regeneration (functional regeneration and morphological regeneration) to complete functional β cell regeneration. Furthermore, I found that pancreatic β cell arose from *neurod1* expressing cells, which contacted with pancreatic α cells directly, during β cell regeneration. Altogether, my cell behavioral analyses shed light on novel and basic cellular mechanisms underlying β cell regeneration. (COI:No)

### Oral Presentation29

### Molecular anatomy, Molecular physiology, Cell biology, Histology (Others)

(March 30, Tue. 15：37〜16：13, Room9)

### OP29-1

**β-arrestin-2 conformational BRET biosensor monitoring tail and core conformation via GPCR-β-arrestin-2 interaction**

Atsuro Oishi[1], Julie Dam [2], Miki Nagase[1], Ralf Jockers[2] ([1]*Kyorin University, Faculty of Medicine, Department of Anatomy,* [2]*Institut Cochin, Univ. Paris/INSERM/CNRS*)

β-arrestins are critical regulators of G proteincoupled receptors (GPCRs) that desensitize G protein signaling, promote receptor internalization, and initiate signaling on their own. Recent structural findings indicate that β-arrestins adopt different conformations upon interaction with agonist-activated GPCRs. Here, we established a β-arrestin-2 conformational bioluminescence resonance energy transfer (BRET) sensor composed of the Nanoluc BRET donor and the red-shifted CyOFP1 BRET acceptor. The sensor monitors early intramolecular conformational changes of β-arrestin-2 in complex with a wide panel of different class A and B GPCRs upon agonist activation. The deletion of the β-arrestin-2 finger-loop region detected the "tail-conformation" corresponding to the interaction of β-arrestin with the C-terminal domain of GPCRs. The new sensors combine the advantages of the BRET technique in terms of sensitivity, robustness, and suitability for real-time measurements with a high responsiveness toward early conformational changes to help to elucidate the different conformational states of β-arrestins associated with GPCR activation in living cells. (COI:No)

### OP29-2

**Roles of Ezrin in regulation of ciliary beating in lung multiciliated cell**

Kotoku Kawaguchi[1], Daichi Saito[1], Haruka Kogiso[2], Kasane Yasuoka[1], Yoshinori Marunaka[2], Takashi Nakahari[2], Shinji Asano[1] ([1]*Dept. Mol. Physiol., Col. Pharm. Sci., Ritsumeikan Univ., Japan.,* [2]*Res. Unit Epithelial Physiol., Res. Org. Sci. and Tech., Ritsumeikan Univ., Japan.*)

The mucociliary clearance is the first line of defense in the lung. The beating cilia plays removing inhaled particles and pathogens. Ezrin, which is a crosslinker between membrane proteins and actin cytoskeleton, may play the important role of apical localization of $\beta_2$-adrenergic receptor ($\beta_2$AR) in multiciliated cells. Here, we examined roles of ezrin in the regulation of ciliary beating in lung multiciliated cells by using ezrin-knockdown ($Vil2^{kd/kd}$) mice. We analyzed ciliary beat frequency (CBF) and ciliary bend distance (CBD) in lung multiciliated cells of mice. In the results, stimulation with 1 nM procaterol, which is selective $\beta_2$AR agonist, increased the ratios of CBF and CBD by about 80% in WT multiciliated cells. However, in $Vil2^{kd/kd}$, stimulation with 1 nM procaterol increased the ratios of CBF and CBD by only 40%. While it is no significant difference that the increase of CBF and CBD by stimulation with 100 μM IBMX in WT and $Vil2^{kd/kd}$. In addition, the cell surface localization of $\beta_2$AR is disturbed in $Vil2^{kd/kd}$ multiciliated cells. These results suggest that ezrin regulates ciliary beating in lung multiciliated cells by promoting the apical localization of $\beta_2$AR. (COI:No)

### OP29-3

**A customizable RNA-binding protein for the visualization and manipulation of endogenous RNAs in living cells**

Akira Takai[1], Yasushi Okada[1,2] ([1]*Lab. for Cell Pol. Reg., BDR, RIKEN,* [2]*Dept. of Phys., Grad. Sch. of Sci., Univ. of Tokyo*)

RNAs do not only serve as the blue print for the protein assembly, but also play wide variety of essential functions in cells.Thus, a method to visualize and manipulate endogenous RNAs in living cells would be beneficial for both basic and applied sciences. Here, we report the development of a designer RNA-binding protein (dRBP), which is customizable to bind to the RNA sequence of interest. We first established an ELISA-like assay using our bright bioluminescent protein, Nano-lantern (Takai et al., PNAS 2015), and showed our dRBPs have high affinity and high specificity to the target RNAs. We also showed our dRBPs can be used for the visualization of the authentic RNAs including ACTB mRNA or lncRNA Neat1_2 in living cells. Immunoprecipitation of the dRBPs followed by quantitative PCR analysis demonstrated that the target, endogenous ACTB mRNA is specifically recognized. Furthermore, manipulation of the localization of the ACTB mRNA using the dRBP fused to constitutively active kinesin resulted in the elongation of cellular processes. These data collectively suggests our dRBP would serve as a powerful tool for the imaging and manipulation of endogenous RNAs in living cells. (COI:No)

### Oral Presentation30

### Ion channels, Receptors

(March 30, Tue. 16：30～17：42, Room8)

### OP30-1

**Bicarbonate transport by airway surface epithelia in luminally-perfused mice bronchioles**

Libin Liu[1], Akiko Yamamoto[1], Makoto Yamaguchi[1], Itsuka Taniguchi[1], Nao Nomura[1], Miyuki Nakakuki[1], Yuka Kozawa[1], Mayuko Higuchi[1], Tsutomu Tamada[2], Hiroshi Ishiguro[1] (*[1]Department of Human Nutrition, Nagoya University Graduate School of Medicine, [2]Department of Respiratory Medicine, Tohoku University Graduate School of Medicine*)

$HCO_3^-$ concentration of airway surface liquid is important for antimicrobial activity and mucociliary clearance in airway. In our study, bronchioles (150-180μm diameter) were dissected from the lungs of C57BL/6J mice and the lumen was microperfused. The epithelium was loaded with BCECF-AM by the lumen and changes in intracellular pH ($pH_i$) were measured by microfluorometry. When both bath and lumen were perfused with the $HCO_3^-$-free Hepes-buffered solution, removal of luminal $Cl^-$ (replaced with gluconate) did not affect $pH_i$. When both bath and lumen were perfused with the $HCO_3^-$-$CO_2$-buffered solution, removal of luminal $Cl^-$ caused reversible elevation of $pH_i$ from 6.94± 0.03 to 7.09±0.03(n=8). And the intracellular alkalization was largely inhibited by luminal application of $H_2$DIDS (200μM) . While luminal application of higher concentration of amiloride (100μM) caused decline of $pH_i$ by 0.06±0.01 (n=8). Luminal application of lower concentration of amiloride (1μM) caused elevation of $pH_i$ by 0.02±0.01 (n=8). In summary, the present data suggest that $Cl^-$-$HCO_3^-$ exchange, $Na^+$-$H^+$ exchange, and ENaC in the apical membrane are involved in $HCO_3^-$ secretion by airway surface epithelia. (COI:No)

### OP30-2

**Role of $Ca^{2+}$-activated $K^+$ channel $K_{Ca}$1.1 in *in vitro* model of tumor microenvironment**

Susumu Ohya[1], Junko Kajikuri[1], Kyoko Endo[1], Hiroaki Kito[1] (*[1]Dept Pharmacol, Grad Sch Med Sci, Nagoya City Univ, Nagoya, Japan*)

Three-dimensional (3D) *in vitro* cell culture system mimics *in vivo* solid tumors resistance to chemotherapy in tumor microenvironment (TME). In the human osteosarcoma MG-63 cells isolated from 3D spheroid models, $K_{Ca}$1.1 activity was largely enhanced, compared with adherent 2D monolayer cells. In MG-63 spheroids, the protein expression level of $K_{Ca}$1.1 was significantly increased, without changing its transcriptional level. The spheroid formation caused down-regulation of the ubiquitin E3 ligase FBXW7, and its inhibition in 2D monolayer cells increased the $K_{Ca}$1.1 protein expression. In the MG-63 spheroids, treatment with the $K_{Ca}$1.1 inhibitor suppressed chemoresistance ability to paclitaxel, doxorubicin, and cisplatin. Of several multidrug resistance ABC transporters, a multidrug resistance-associated protein, MRP1 was up-regulated in the MG-63 spheroids, and chemosensitivity was recovered by the $K_{Ca}$1.1 inhibition. Our studies revealed the pathophysiological significance of $K_{Ca}$1.1 up-regulation in solid-tumor microenvironment *in vitro* using 3D spheroid model. (COI:No)

### OP30-3

**Phylogenetic approach to understanding molecular mechanisms of enzyme actions of voltage-sensing phosphatase**

Ian Costa Paixao[1], Takafumi Kawai[2], Natsuki Mizutani[2], Yoshifumi Okochi[2], Yasushi Okamura[1,2] (*[1]Osaka University, Frontier Bioscience, [2]Osaka University, dept of Medicine*)

Voltage-sensing phosphatase (VSP) shows phosphoinositides (PIPs) phosphatase activity that is coupled to membrane potential. In spite of its interesting property, the biophysical mechanism underlying the enzyme activity remains elusive. We found that some mammals of the Marsupialia infraclass possess an amino acid change on the residue 382, which is located within the PIPs binding site, even though it is highly conserved in most other animals. Here, we analyzed the impact of Lysine-382 on the enzyme activity of Ci-VSP. Interestingly, the mutations, mainly to Leucine or Arginine, seem to impact voltage-sensor movement as analyzed by electrophysiological recording of "sensing" currents that correspond to charge mobilizations of the voltage sensor. Furthermore, the mutation significantly affected the activity toward one of the substrates, PI(3,4)$P_2$, while more moderately affecting the activity toward PI(4,5)$P_2$. Now, other amino acid mutations on this site are being studied, such as Isoleucine, Glutamine and Arginine, in order to understand the mechanism and importance of Lysine-382. We discuss the functional mechanism and importance of PIPs interaction in VSP functioning machinery. (COI:No)

### OP30-4

**Essential role of TRPA1 for fetus survival during anemia**

Jiacheng Sun[1] (*[1]Gradutaes School of Engineering and Faculty of engineering, Kyoto University*)

Anemia in pregnancy is one of the most frequent causes for new-born outcomes. However, the mechanism that protect fetus from anemia is still unknown. Here, our study reveals the essential role of TRPA1 for fetus survival in anemia. *Trpa1* KO mouse embryo exhibits intrauterine lethality during anemia, yet survives in normoxia. Surrogacy experiment indicates that fetal TRPA1 contributes to pregnancy outcomes during anemia. We found that TRPA1 is expressed in trophoblast giant cells (TGCs) which are central to placentation and the regulation of blood supply to the embryo. *Trpa1* disruption abolished anemia-induced vascular remodeling in placenta due to the lack of migration of TGCs into uterine spiral arteries, which lead to insufficient blood flow to fetus. This mechanism may be account for intrauterine lethality of the embryo, but on contrarily can also protect the mother from hypoxia stress during severe anemia. Our findings suggest that hypoxia sensitivity of trophoblast through TRPA1 enables the placenta to protect fetus from anemia and provide a theoretical basis for the treatment of this disease. (COI:No)

### OP30-5

**Voltage-Sensing Phosphatase (VSP) Facilitates Nutrient Absorption in Zebrafish Enterocytes.**

Adisorn Ratanayotha[1], Makoto Matsuda[1], Yukiko Kimura[2], Israil M. Hossain[1], Shinichi Higashijima[2], Takafumi Kawai[1], Yasushi Okamura[1] (*[1]Laboratory of Integrative Physiology, Department of Physiology, Graduate School of Medicine, Osaka University, Japan, [2]Laboratory of Behavioral Neurobiology, Department of Biodesign Research, Okazaki Institute for Integrative Bioscience, Aichi, Japan , [3]Department of Anatomy, Faculty of Medicine Siriraj Hospital, Mahidol University, Bangkok, Thailand*)

Voltage-sensing phosphatase (VSP) is a membrane protein that translates membrane electrical activities into phosphoinositide signals within the cells. VSP orthologs from various species have been studied for their biophysical properties using a heterologous expression system. However, the functional role of VSP in native tissues remains unclear. Here we show that zebrafish VSP (Dr-VSP) is functionally expressed in lysosome-rich enterocytes (LREs) –the specialized intestinal epithelial cells which absorb dietary proteins. We found that Dr-VSP is associated with the intracellular vesicles and facilitates endocytosis in the zebrafish LREs. Dr-VSP-deficient LREs were significantly inefficient in forming endosomal vesicles after initial endocytosis of dextran and mCherry, suggesting the impairment of nutrient absorption. We also found that Dr-VSP-deficiency disrupts the morphological integrity of LREs. Moreover, Dr-VSP-deficient zebrafish showed growth restriction and higher mortality during early development. Our results demonstrate a novel concept of VSP being expressed at intracellular structures and highlight its function in nutrient absorption through specialized epithelial cells. (COI:No)

### OP30-6

**Late Sodium Current Inhibitor Shortens Prolonged Action Potential Durations in iPS Cell-derived Cardiomyocytes of long QT syndrome associated with Cav 1.2 Ion Selectivity Disruption**

Asami Kashiwa[1], Takeru Makiyama[1], Hirohiko Koujitani[1], Yuta Yamamoto[1,3], Jingshan Gao[1], Hai Huang[1], Tomohiko Imamura[1], Takanori Aizawa[1], Taisuke Ishikawa[2], Seiko Ono[3], Futoshi Toyoda[4], Seiichi Sato[5], Kazuhiro Takahashi[6], Minoru Horie[7], Takeshi Kimura[1] (*[1]Department of Cardiovascular Medicine, Kyoto University Graduate School of Medicine, [2]National Cerebral and Cardiovascular Center, Research Institute and Omics Research Center, [3]Department of Bioscience and Genetics, National Cerebral and Cardiovascular Center, [4]Department of Physiology, Shiga University of Medical Science, [5]Division of Pediatric Cardiology & Pediatric Intensive Care Unit, Okinawa Prefectural Nanbu Medical Center & Children's Medical Center, [6]Kizawa Memorial Hospital, [7]Department of Cardiovascular and Respiratory Medicine, Shiga University of Medical Science*)

**Background:** *CACNA1C*-E1115K, a missense mutation located in the crucial site of $Ca^{2+}$ selectivity in L-type Ca channel, was reported to cause arrhythmogenic phenotypes. The physiopathological mechanism of this mutation and targeted drug therapy are unclear.
**Methods and Results:** Using iPSC-CMs derived from a patient carrying heterozygous *CACNA1C*-E1115K, presenting LQT and Brugada syndrome, we performed the electrophysiological analysis. As a control, we created isogenic iPSC lines by the CRISPR-Cas9 system. Patch-clamp recordings showed impaired $Ca^{2+}$ ion selectivity, abnormal permeability to monovalent cation, and longer AP durations (APD) in E1115K-iPSC-CMs (E1115K). Under AP-clamp, $I_{NaL}$ was markedly upregulated when using voltage command of E1115K than that of control.Furthermore, we evaluated the response to $I_{NaL}$ inhibitors. In optical recordings using a voltage-sensitive dye, mexiletine and GS-458967, which inhibit $I_{NaL}$, shortened APDs specifically in E1115K. $I_{NaL}$ might contribute to APD prolongation in E1115K.
**Conclusion:** We demonstrated upregulation of $I_{NaL}$ and efficacy of $I_{NaL}$ inhibitor in E1115K-iPSC-CMs. $I_{NaL}$ blockers might be therapeutic candidates in this mutation. (COI:No)

### Oral Presentation31

### Endocrine

(March 30, Tue. 16：30～17：42, Room9)

### OP31-1

**UCP1-reporter mice reveal UCP1 protein expression in the adrenal medulla**

Hirofumi Fujita[1], Munenori Habuta[1], Takako Hattori [2], Satoshi Kubota [2], Hiromi Kumon[3,4], Hideyo Ohuchi[1] (*[1]Department of Cytology and Histology, Okayama University Graduate School of Medicine, Dentistry and Pharmaceutical Sciences, [2]Department of Biochemistry and Molecular Dentistry, Okayama University Graduate School of Medicine, Dentistry and Pharmaceutical Sciences, [3]Innovation Center Okayama for Nanobio-Targeted Therapy, Okayama University, [4]Niimi University*)

The Uncoupling protein 1 (UCP1) gene is known to be highly expressed in brown adipose tissue (BAT). Although human whole-body FDG-PET analysis which can detect thermogenic tissues shows high FDG uptake at perirenal regions containing the adrenal gland, significance of UCP1 protein expression in the adrenal gland remains unclear. To explore the regulation of *UCP1* expression in the adrenal gland, we generated a reporter knockin mouse in which the *GFP* gene was inserted into the *UCP1* locus using CRISPR-Cas9 system. Western blot analysis verified that *UCP1*-driven GFP protein expression in the interscapular BAT of the knockin mice kept at 4℃. Immunostaining showed that GFP protein was detected in the adrenal gland of the knockin mice. More intense GFP expression was observed in the adrenal medulla than in the cortex of the reporter mice with or without cold exposure. Consistent with this, immunohistochemistry using anti-UCP1 antibody, as well as western blot analysis showed UCP1 protein expression in the wild-type adrenal medulla. These results suggest that the mouse adrenal gland is a novel organ expressing UCP1 protein and its expression is not influenced by cold exposure.

(COI:Properly Declared)

### OP31-2

**Vasopressin neurons exhibit reciprocal volume responses to hyperosmotic stimulation with the secretory volume decrease (SVD) and the regulatory volume increase (RVI)**

Kaori Sato-Numata[1], Tomohiro Numata[2], Yoichi Ueta[3], Yasunobu Okada[4,5,6] (*[1]Japan Society for the Promotion of Science, [2]Department of Physiology, School of Medicine, Fukuoka University, [3]Department of Physiology, School of Medicine, University of Occupational and Environmental Health, [4]National Institute for Physiological Sciences, [5]Department of Physiology, School of Medicine, Aichi Medical University, [6]Department of Physiology, Kyoto Prefectural University of Medicine*)

Most animal cell types exhibit cell volume regulation after osmotic shrinkage, called the regulatory volume increase (RVI). However, arginine vasopressin (AVP) neurons respond to hyperosmotic stimulation with persistent shrinkage without showing volume recovery. We found that the AVP secretion induced by hyperosmotic stimulation was abolished by an exocytosis inhibitor, tetanus toxin (TeTx), and by known blockers, $Ni^{2+}$ and flufenamic acid (FFA), for voltage-gated $Ca^{2+}$ channels (VGCCs) in AVP neurons (Sato-Numata et al. 2020 J Physiol Sci). In the presence of TeTx, $Ni^{2+}$ or FFA, AVP neurons exposed to a hyperosmotic solution exhibited osmotic shrinkage followed by a clear RVI event which was inhibited by blockers for $Na^+/H^+$ and $Cl^-/HCO_3^-$ antiporters. Taken together, it is concluded that AVP neurons respond to hyperosmotic stimulation with osmotic shrinkage followed by reciprocal volume responses, that is, the RVI event and additional shrinkage coupled to AVP secretion, called the secretory volume decrease (SVD), thereby apparently exhibiting persistent shrinkage. (COI:No)

### OP31-3

**Histone deacetylase 3 inhibitor alleviates motor coordination defects in perinatal hypothyroid mice**

Sumiyasu Ishii[1], Alvin Susetyo[1], Izuki Amano[1], Noriyuki Koibuchi[1] (*[1]Department of Integrative Physiology, Gunma University Graduate School of Medicine*)

Background: Histone acetylation status is highly correlated with transcriptional regulation. However, the role of histone acetylation/deacetylation in cerebellar development remains largely unknown. Perinatal hypothyroidism impairs cerebellar organogenesis and results in motor coordination defects. In the absence of ligand, thyroid hormone receptor binds to co-repressor complexes which contain histone deacetylase (HDAC) 3, and acts as a transcriptional repressor. In this study, we aim to study the role of HDAC3 in the cerebellar developmental defects induced by hypothyroidism.

Methods: Perinatal hypothyroidism and cerebellar defects were induced by an anti-thyroid agent propylthiouracil in mice. The mice were further treated with a specific HDAC3 inhibitor, RGFP966. Motor coordination was analyzed by three behavioral tests.

Results: Treatment with HDAC3 inhibitor significantly alleviated motor coordination defects in hypothyroid mice, suggesting that the enzymatic activity of HDAC3 contributes to motor coordination defects in these mice.

Conclusion: HDAC3 plays an important role in cerebellar developmental defects induced by hypothyroidism. (COI:No)

### OP31-4

**Investigation of molecular mechanisms in pancreatic β cell regeneration using β-cell-specific injured mice**

Ryo Hatano[1], Xilin Zhang[1], Gyokketsu Ma[1], Xue Chen[1], Eunyoung Lee[1], Atsushi Kaneda[2], Takashi Miki[1] (*[1]Department of Medical Physiology, Chiba University Graduate School of Medicine, [2]Department of Molecular Oncology, Chiba University Graduate School of Medicine, Japan*)

Reduced β-cell mass is known to be associated with pathogenesis of diabetes mellitus (DM). The regulation of β-cell mass involves a balance of its replication and apoptosis. To maintain the β-cell proliferative ability is essential to prevent progression of DM. To understand the mechanisms, we examined a process of β-cell proliferation after ablating a majority of β-cell using β-cell-specific diphtheria toxin receptor (DTR) expressing mice in this study. DT administration ablated 70% of β-cells, in which Cre-mediated DTR expression was induced. Intact β-cells (~30%) increased by 2.5 folds at day 14, suggesting that proliferation of intact β-cells replenished the reduced β-cell mass. qPCR analysis of isolated islets showed significant decrease of MafA, a mature β-cell marker and significant increase of Neurogenin 3 and MafB, immature β-cell markers at day 6 and 14. Furthermore, Aldh1a3, a marker for β-cell dedifferentiation, was significantly increased, suggesting that β-cell dedifferentiation is associated with compensatory β-cell proliferation. These results suggest that compensation of β-cell mass is mediated through the dedifferentiation of β-cells in DTR mice. (COI:No)

### OP31-5

**Enhancement of insulin secretion by pentadecanoic acid-based triglyceride**

Kazuhito Tomizawa[1], Korin Sakakida[1], Hitomi Kaneko[1], Kunimitsu Kaya[3], Makoto Tsuboi[3], Yasuko Sakata[3], Seiji Sakamoto[3], Fan-Yan Wei[1,2] (*[1]Dept. of Mol. Physiol., Faculty of Life Sci., Kumamoto Univ., [2]Dept. of Modomics Biol. & Med., IDAC, Tohoku Univ., [3]Sea Act Co., Ltd.*)

Even-chain saturated fatty acid concentrations are associated with an increased risk of coronary heart disease, whereas odd-chain saturated fatty acid concentrations such as pentadecanoic acid (15:0) and heptadecanoic acid (17:0) are associated with a decrease of the risk. Similarly, even-chain saturated fatty acids are positively associated with type 2 diabetes, whereas odd-chain saturated fatty acids are thought to be inversely associated with type 2 diabetes (T2D). We established a purification method of pentadecanoic acid -based triglyceride (PABT) from Aurantiochytrium limacinum, a kind of microalgae. Here we demonstrate the effect of the purified PABT on insulin secretion in a mouse pancreatic beta-cell line. PABT increased the cell viability of the cells in a dose-dependent manner. PABT significantly induced high glucose-stimulated insulin secretion but not low glucose-stimulated insulin secretion. In contrast, pentadecanoic acid had no effect on high glucose-stimulated insulin secretion. These results suggest that PABT is more effective on insulin secretion rather than pentadecanoic acid. We are now demonstrating the impact of PABT in an Asian-type diabetes model mouse. (COI:No)

### OP31-6

**The effect of lactational PFOS exposure on cerebellar function and motor coordination**

Ayane Ninomiya[1], Abdallah Mshaty[1], Asahi Haijima[1], Hiroyuki Yajima[1], Michifumi Kokubo[1], Aghnia Miski Khairinisa[1], Winda Ariyani[1], Yuki Fujiwara[1], Sumiyasu Ishii[1], Izuki Amano[1], Noriyuki Koibuchi[1] (*[1]Dept Integrative Physiol, Grad Sch Med, Gunma Univ*)

Perfluorooctane sulfonate (PFOS) is an endocrine disrupter and was widely used in industries and consumer products. PFOS has a possible significant neurotoxicity in humans and rodents. However, the effects of PFOS exposure on the cerebellar development and function remain unclear. Thus, we examined the effect of early lactational PFOS exposures on motor coordination of male mice. PFOS solution was orally administered to dams during the post-partum days 1 to 14. After postnatal weeks 8-10, the rotarod test was performed. We then performed a whole-cell patch-clamp recording to examine the PFOS effects on the cerebellar synaptic plasticity. PFOS-exposed mice showed significant decreases in time-to-fall latency compared to control ones. The PFOS group showed the attenuation of the induction of long-term depression at parallel fiber (PF) – Purkinje cell synapses. Also, the glutamate release probability from PFs was attenuated in the PFOS group. Our study showed that postnatal PFOS exposure has profound long-lasting effect on the cerebellar function.

(COI:No)

### Oral Presentation32

### Pathophysiology

(March 30, Tue. 16：30～17：42, Room10)

### OP32-1

### Investigation of novel Iba1-positive cells in the ischemic core

Toshinori Sawano[1], Natsumi Yamaguchi[1], Jin Nakatani[1], Shinobu Inagaki[2,3], Takayuki Nakagomi[4], Tomohiro Matsuyama[4], Hidekazu Tanaka[1] (*[1]Lab. Pharm., Dept. Biomed Sci., Ritsumeikan Univ., [2]United Grad. Sch. Child Develop., Osaka Univ., [3]Dept. PT., Yukioka Col. Health Sci., [4]Lab. Neurogenesis and CNS Repair., Inst. Adv. Med. Sci., Hyogo Col. Med.*)

Brain ischemia induces massive cell death, and ischemic core is considered as unsalvageable area. However, we previously demonstrated that brain pericytes develop multipotency in the ischemic core, and these ischemia-induced multipotent stem cells (iSCs) can contribute to tissue repair. In this study, we identified novel Iba1 (microglia marker)-positive cells in the ischemic core. They expressed Nestin (neuroectodermal stem cell marker), and parabiotic analysis revealed that these novel Iba1-positive cells were not derived from peripheral blood cells. Microarray analysis showed that vascular development-related genes were abundant in ischemic core residential Iba1-positive cells. Furthermore, depletion of Iba1-positive cells using PLX3397 caused the reduction in the number of iSCs-sphere derived from ischemic core. These results suggest that novel Iba1 cells appearing in the ischemic core contribute to maintenance of pericyte-derived iSCs. (COI:No)

### OP32-2

### Antiallodynic effect of betanin (red beetroot extract) via modulation of microglial activation in a mouse model of neuropathic pain

Nichakarn kwankaew[1], Hiroaki Okuda[1], Kiyomi Hori[1], Tatsuya Ishikawa[1], Tsuneo Nakamura[1], Yoshitake Shiraishi[1], Noriyuki Ozaki[1] (*[1]Dept Functional Anat, Kanazawa Univ, Grad Med Sci*)

Neuropathic pain (NeP) medications, such as opioids, have several side effects that affect NeP patients' quality of life. In this study, we focused on betanin (red beetroot extract) as a potential therapeutic candidate for NeP. In chronic constriction injury (CCI) model mice, repeated betanin treatment, both intraperitoneally and orally, attenuated developing mechanical allodynia in a dose-dependent manner without impairing motor coordination. In addition, betanin treatment attenuated developed mechanical allodynia and prevented the onset of mechanical allodynia in CCI mice. Microglial activation in the spinal cord is known to play a key role in the development of NeP. In our experiment, Betanin treatment reduced CCI-induced microglial activation in the spinal cord. Moreover, in primary microglia cultured cells, the activation of microglia by lipopolysaccharide application was suppressed by betanin treatment. In summary, betanin treatment apparently ameliorates mechanical allodynia associated with CCI-induced NeP in mice by inhibiting microglial activation. These findings suggest that betanin could be a useful potential therapeutic candidate for NeP. (COI:No)

### OP32-3

### Longitudinal MR imaging reveals blood-spinal cord barrier as a predictive biomarker of demyelination in targeted EAE mouse.

Takahide Itokazu[1], Takeshi Hirata[1,4], Atsushi Sasaki[1,4], Fuminori Sugihara[3], Toshihide Yamashita[1,2,3] (*[1]Neuro-Medical Science, Graduate School of Medicine, Osaka University, [2]Molecular Neuroscience, Graduate School of Medicine, Osaka University, [3]Immunology Frontier Research Center, Osaka University, [4]Mitsubishi Tanabe Pharma*)

For the effective development of new therapeutic agents, establishing sensitive biomarkers that reflect disease status with temporal and spatial resolution are of great importance. However, considering the neurological disorders, there are still few fluid diagnostic markers. To test the availability of MRI as a predictive biomarker of multiple sclerosis (MS), we established quantitative evaluation system of mouse spinal cord by using high-field MRI, then analyzed the spinal cord of targeted EAE model mouse. MRI parameters including Gd leakage (blood-spinal cord barrier(BSCB) disruption) and radial diffusivity (RD) map (demyelination) were obtained longitudinally with clinical score at each time point. By using this system, the relationship between vascular failure and the degree of demyelination in the same individual can be analyzed quantitatively over time, and the results indicated that the extent of BSCB disruption is a predictive factor of demyelination. Our results suggest that imaging quantification of CNS vascular disruption could be used as a biomarker for stratification and treatment efficacy in clinical trials for MS patients. (COI:Properly Declared)

### OP32-4

### Real time *in vivo* imaging of brain metastasis visualized dynamic reaction of microglia against cancer cells

Takahiro Tsuji[1], Hiroaki Wake[1], Mariko Shindo[1], Daisuke Kato[1], Hiroaki Ozasa[2], Toyohiro Hirai[2] (*[1]Department of Anatomy and Molecular Cell Biology, Graduate School of Medicine, Nagoya University, [2]Department of Respiratory Medicine, Graduate School of Medicine, Kyoto University*)

Cancer cells (CCs) may escape from host immunity to form distant metastasis. However, the detailed interaction between CCs and immune cells in the brain niche is still unclear. Here, we develop an *in-vivo* imaging method to evaluate pathological immune reaction against CCs in single cell resolution with transcriptome profile.

Lung CCs (CMT167) expressing mCherry was injected via the internal carotid artery of the CX3CR1-GFP mice, and the cerebral cortex were visualized *in vivo* using two-photon microscopy for 14 days. Monitoring of embolized CCs in the brain revealed 49.8% of CCs displaced, 40.4% developed micro-metastasis (MMs), and 6.9% were phagocytosed by microglia. The microglia around MMs were activated, suggesting that CCs can escape from activated microglia. Microglia depletion can suppress MMs that implicates microglia can inversely promote tumor progression. A genetic deletion of *CD47* of CCs, the molecule candidate "don't eat me signal", significantly reduced the formation of MMs.

We are further developing a method for isolating CCs labeled *in vivo* using holographic stimulation and photo-switching protein, for evaluating by single cell transcriptome. (COI:No)

### OP32-5

### Troponin T amino acid mutation (ΔK210) knock-in mice is good animal model for neonatal dilated cardiomyopathy.

Jun Tanihata[1], Teruyuki Fujii[1], Shunsuke Baba[1], Yoshitaka Fujimoto[1], Sachio Morimoto[2], Susumu Minamisawa[1] (*[1]Department of Cell Physiology, The Jikei University School of Medicine, Tokyo, Japan, [2]Department of Health Science Fukuoka, International University of Health and Welfare, Okawa, Japan*)

**Introduction:** Dilated cardiomyopathy (DCM) in children is exhibits distinct pathological entities from those of adult DCM. Due to the limited number of patients and the lack of a good animal model, the molecular mechanisms underlying pediatric DCM remain poorly understood. The purpose of this study is to establish an animal model of neonatal DCM and identify early progression factors.

**Methods:** Cardiac phenotypes and gene expression profiles in homozygous ΔK210 knock-in ($TNNT2^{\Delta K210/\Delta K210}$) mice were analyzed compared to $TNNT2^{+/\Delta K210}$ and wild-type mice at 0days and 1week of age.

**Results:** Immediately after birth, the cardiac weight in $TNNT2^{\Delta K210/\Delta K210}$ mice was already increased that in $TNNT2^{+/\Delta K210}$ and wild-type mice. Echocardiographic examination of 1-week-old $TNNT2^{\Delta K210/\Delta K210}$ mice revealed similar phenotypes of pediatric DCM. In addition, the KEGG PATHWAY analysis revealed several important pathways such as cancer and focal adhesion that might be associated with the pathogenesis and development of DCM.

**Conclusion:** $TNNT2^{\Delta K210/\Delta K210}$ mice have already developed DCM at birth, indicating that they should be an excellent animal model to identify early progression factors of DCM. (COI:No)

### OP32-6

### Allele-specific down-regulation of a mitochondrial gene in familial bipolar disorder iPSC-derived neuronal cells

Gakuya Takamatsu[1], Yoko Manome[4], Kumiko Yanagi[5], Junseok Lee[1], Kae Koganebuchi[6], Dimitar Dimitrov[9], Kotaro Hattori[7,8], Chikako Hara Miyauchi[4], Minami Hasegawa[4], Hiroshi Kunugi[7], Tomoyuki Takahashi[9], Tsuyoshi Kondo[2], Tadashi Kaname[5], James Hirotaka Okano[4], Ryosuke Kimura[3], Masayuki Matsushita[1] (*[1]Department of Molecular & Cellular Physiology, Graduate School of Medicine, University of the Ryukyus, [2]Department of Neuropsychiatry, Graduate School of Medicine, University of the Ryukyus, [3]Department of Human Biology and Anatomy, Graduate School of Medicine, University of the Ryukyus, [4]Division of Regenerative Medicine, Jikei University School of Medicine, [5]Department of Genome Medicine, National Center for Child Health and Development, [6]Department of Biological Sciences, Graduate School of Science, The University of Tokyo, [7]Department of Mental Disorder Research, National Institute of Neuroscience, National Center of Neurology and Psychiatry, [8]Medical Genome Center, National Center of Neurology and Psychiatry, [9]Cellular and Molecular Synaptic Function Unit, Okinawa Institute of Science and Technology Graduate University*)

Bipolar disorder (BP) is a major psychiatric disorder. The pathophysiology is little understood. Genetics is important for the development of BP; however, identifying genomic variants that strongly contribute to BP is still unprecedented. Here, to elucidate the pathogenesis of BP, we focused on rare familial cases with potential high-risk genetic factors and conducted allele-specific expression analysis using patient-derived induced pluripotent stem cells (iPSCs). First, we performed parametric linkage analysis on a three-generation multiplex family with BP. Interestingly, we detected a significant linkage peak in a chromosome 1p region, previously reported links to BP. Then, we determined the haplotype in the region and, to explore putative cis-regulatory variations, we analyzed allelic imbalances of transcripts from the haplotype by RNA sequencing on iPSC-derived neurons of the family. Finally, we found decreased expression in the affected allele compared with the reference allele of a gene encoding a key mitochondrial regulator protein. It might contribute to mitochondrial dysfunction and the development of the disease in the family. (COI:Properly Declared)

### Poster Presentations

### Day1

(March 28, Sun. 18：30～19：30)

| | |
|---|---|
| **PP-1～PP-17** | Neuroanatomy, Neurophysiology, Neuronal cell biology : Neural development |
| **PP-18～PP-27** | Neuroanatomy, Neurophysiology, Neuronal cell biology : Plasticity |
| **PP-28～PP-40** | Neuroanatomy, Neurophysiology, Neuronal cell biology : Neuronal projection, Neural network |
| **PP-41～PP-51** | Neuroanatomy, Neurophysiology, Neuronal cell biology : Neurohistochemistry, Neurochemistry |
| **PP-52～PP-69** | Neuroanatomy, Neurophysiology, Neuronal cell biology : Neurons, Synapses |
| **PP-70～PP-83** | Neuroanatomy, Neurophysiology, Neuronal cell biology : Glia |
| **PP-84～PP-95** | Neuroanatomy, Neurophysiology, Neuronal cell biology : Higher brain function |
| **PP-96～PP-99** | Neuroanatomy, Neurophysiology, Neuronal cell biology : Motor function |
| **PP-100～PP-121** | Neuroanatomy, Neurophysiology, Neuronal cell biology : Sensory function, Sensory organ |
| **PP-122～PP-135** | Neuroanatomy, Neurophysiology, Neuronal cell biology : Neurological disorders, Neuropathophysiology |
| **PP-136～PP-137** | Neuroanatomy, Neurophysiology, Neuronal cell biology : Others |

### PP-1

### Alteration of parvalbumin expression and perineuronal nets formation in the anterior cingulate cortex of Fabp3 KO mice

Yui Yamamoto[1,2], Masaki Ogata[1], Keiju Kamijo[1], Yuji Owada[2] ([1]*Dept. Anatomy, Tohoku Med. and Pharm. Univ.*, [2]*Dept. Organ Anatomy, Tohoku Univ. Grad. Sch. Med*)

Polyunsaturated fatty acids (PUFAs) are essential for brain development and function. Increasing evidence has shown that an imbalance of PUFAs is associated with various human psychiatric disorders, including autism and schizophrenia. Fatty acid-binding proteins (FABPs), cellular chaperones of PUFAs, are involved in their intracellular trafficking, signal transduction, and gene transcription. Previously, we showed that FABP3 is strongly expressed in the parvalbumin-expressing interneurons (PV neurons) of the mouse anterior cingulate cortex (ACC) and regulates GABA synthesis through transcriptional regulation of *Gad67*. In this study, we analyzed the density and the percentage of PV neurons surrounded by perineuronal nets (PNNs) in the ACC of *Fabp3* KO mice. PV density increased in the ACC of *Fabp3* KO mice, whereas the number of PV-neurons remained unchanged. The density of PNN and the number of PNN-positive PV neurons were significantly increased in the ACC of *Fabp3* KO mice. These findings demonstrate that FABP3 is involved in the control of expression of PV and formation of PNNs in the ACC, thus suggesting the importance of PUFA homeostasis in the ACC for maturation of PV neurons. (COI:No)

### PP-2

### Expression patterns of PSA-NCAM in the olfactory placode-derived migratory neurons during early developmental stages

Shizuko Murakami[1], Yasuo Uchiyama[1] ([1]*Dept of Cellul and Mol Neuropathol, Juntendo Univ Grad Sch of Med, Tokyo, Japan*)

The olfactory placode (OP) generates the neuronal cells which migrate from the OP into the mesenchyme. In chick embryos, the early OP-derived migratory cells which express highly polysialilated NCAM (PSA-NCAM), form a cellular cord extending from the OP to the telencephalon (TEL) at the Hamburger and Hamilton stages (HH) 18-20. The physical destruction of the cellular cord induced a lack of olfactory sensory neuron (OSN) axon innervation of the TEL, suggesting that the early OP-derived neurons may guide the axon growth of OSN. To examine the property of early OP-derived neurons, a GFP vector was introduced into the OP of HH13-17 embryos. After 1 day, a few GFP-labeled cells were observed in the cellular cord. Almost all cells expressed PSA-NCAM. After 2-3 days, the proportion of GFP-labeled and PSA-NCAM-positive cells decreased to 30% by these stages. It seems likely that GFP-labeled and PSA-NCAM-negative cells are positioned at their migratory pathway, since PSA-NCAM is thought to play a role in the neuronal migration. These raise the possibility that early OP-derived neurons with PSA-NCAM locate in the presumptive olfactory nerve tract as guidepost cells. (COI:No)

### PP-3

### Two distinctive pathways migrating progenitor cells form granule cell layer in the dentate gyrus

Hiroshi Shinohara[1], Tatsunori Seki [1] ([1]*Histol Neuroanat., Tokyo Med Univ.*)

The dentate gyrus (DG) continues neurogenesis from embryonic to adult stages. Despite the interest for adult neurogenesis, the DG cellular migration are not yet completely understood. In contrast, the cellular migration of cerebral cortex (NCX) is studied well. In the embryonic NCX, neural progenitors migrate from apical to basal region (i.e. cortical plate). To address the cellular migration of DG, we focused the migration of the dentate progenitor cells and performed *in vivo* observation and time-lapse imaging of DG primordium using *Gfap*-GFP mice. In the early stage, interestingly, dorsal and ventral cell migrating population were observed, and both cellular populations migrated toward the invagination point of the hippocampal fissure (HF) and mixed. In the late stage, we found the pia-bound and the HF-bound migratory pathways. It seems that HF- and pia-bound cells derive from dorsal population and ventral population respectively. Moreover, it is observed that HF- and pia-bound cells differentiate into granule cells. These results demonstrate that two distinctive dorsal and ventral pathways form granule cell layer in the developing DG and the difference from the NCX. (COI:No)

### PP-5

### Effects of netrin-1 on the subpopulations of developing layer 5 cortical neurons

Hideko Matsumoto[1], Masabumi Nagashima[1] ([1]*Dept. Anatomy, Saitama Med. Univ*)

A multifunctional axon guidance cue netrin-1 is known to play various important roles in the correct wiring of the nervous system during development. We previously observed two distinct functions of netrin-1 – promotion of axon outgrowth and promotion of axon collateral branching – in dissociated cerebral cortical neurons of embryonic mice depending on developmental stages. As the cortical culture employed in that study was supposed to be highly heterogeneous containing multiple populations of neurons, we started to take an interest in the identification and characterization of the population exerting each of these netrin-1 functions.

The aim of the present study is to find out whether subpopulations of cortical neurons which reside in layer 5 would show netrin-1-induced axon outgrowth and/or axon branching. Using dissociated cortical neurons of embryonic mice, corticospinal motor neurons and layer 5 callosal projection neurons were respectively labeled and then subjected to the analyses of the lengths of primary axon shafts and the numbers of branch points with or without netrin-1 stimulation. (COI:No)

### PP-6

### The functional Morphology of the hippocampus in Wnt10A-deficient mouse

Jiahe Zhang[1], Qian Zhou[1], Xiao Liu[1], Ke-Yong Wang[2], Haruki Hayashi[1], Yasuhiro Adachi[1], Kagaku Azuma[1] ([1]*Dept Anat, Sch Med, UOEH, Japan*, [2]*Shared-use Res, Sch Med, UOEH, Japan*)

Previous studies showed that the deficiency of Wnt10A causes ectodermal dysplasia. The present study investigated the functional morphology of the hippocampus in Wnt10A-/-mice.Experiments were performed in 20- to 25-week-old male Wnt10A-/- mice and wild-type C57BL/6J mice. Brains were removed, fixed in 4% paraformaldehyde and serial sections were prepared. The hippocampus volume was calculated using imaging software. Immunohistochemistry was carried out by incubating with anti-doublecortin and anti-Iba1. Western blot was performed using anti-β-catenin, amyloid-β, BDNF, PSD95 and synaptophysin.As compared with the wild-type mouse, hippocampal volume was significantly lower in Wnt10A-/-mouse. The number of doublecortin-positive cells and Iba1-positive microglia in the hippocampus were significantly decreased. The protein expression of β-catenin, BDNF, PSD95 and synaptophysinwas significantly lower, while the expression of amyloid-β in the hippocampus was significantly higher. These findings suggest that Wnt10A signaling pathway plays a pivotal role in hippocampus-dependent cognitive function. (COI:No)

### PP-7

### Cdk5 regulates phosphorylation of αII-spectrin and axonal periodic cytoskeletal structures.

Takeshi Yoshimura[1], Matthew Rasband[2], Taiichi Katayama[1] ([1]*Dept. of Child Dev. & Mol. Brain Sci., United Grad. Sch. of Child Dev., Osaka Univ., Suita, Japan*, [2]*Dept. of Neurosci., Baylor College of Med., Houston, TX, USA*)

Axons have a specific cytoskeletal structure lining the cytoplasmic face of the axolemma. Super-resolution microscopy has revealed a remarkable periodic lattice in axons. The spectrin/ankyrin-based cytoskeleton forms this periodic structure. αII-spectrin, βIV-spectrin and Ankyrin-G form the periodic lattice in the axon initial segment (AIS), whereas the distal periodic cytoskeleton consists of αII-spectrin, βII-spectrin and Ankyrin-B. Spectrins are widely expressed and exist as tetramers consisting of two α and two β subunits. βI-IV spectrins are found in neurons. However, αII-spectrin is the only α spectrin detected in the nervous system, suggesting that αII-spectrin must play important roles in the entire axon. It was reported that human mutations in αII-spectrin cause West syndrome. While αII-spectrin intracellular localization is fairly well understood, the molecular mechanism by which αII-spectrin is regulated remains unclear. Here, we report that Cdk5 phosphorylates αII-spectrin. Inhibition of Cdk5 impaired AIS formation and neuronal polarity. These results suggest that Cdk5 regulates the axonal periodic cytoskeleton through the phosphorylation of αII-spectrin. (COI:No)

### PP-8

### Foxp2 related to vocalization decreases at the specific regions in the chick midbrain after hatching

Chikafusa Bessho[1], Shunji Yamada[1], Takashi Tanida[1], Masaki Tanaka[1] ([1]*Kyoto Pref. Univ., Grad. Med., Anatomy &Neuro. Bio*)

FOXP2, a transcription factor, regulates human speech and animal vocalization. As an animal model of vocal learning, zebra finch has been well studied, and it has been shown that FoxP2 decreases in the area X of zebra finch brain after singing.
On the other hand, the expression and function of FoxP2 of avian vocal non learner are incompletely investigated, although there are lots of data indicating that the midbrain is important for innate vocalization.
To reveal the relationship between the FoxP2 distribution and the call of avian vocal non learner, we analyzed the Foxp2 protein in the midbrain of chick that doesn't call before hatching but beep after hatching. By western blotting, we found the significant reduction of FoxP2 protein in the midbrain of post hatched chick. Quantitative immunohistochemistry revealed that FoxP2 positive cells significantly decreased at optic tectum layer1 and torus semicircularis in midbrain of post hatched chick. Thus, our findings support our hypothesis that the change of Foxp2 expression at the specific regions in midbrain controls vocalization in avian vocal non learners. (COI:No)

### PP-9

### Analysis of mRNA transport mechanism of a cell cycle regulator Cyclin D2 in radial glial cells: a possible mechanism for enlargement of the cortex in placental mammals

Takako Kikkawa[1], Yoshio Wakamatsu[1], Yukiko U Inoue[2], Kunihiro Suzuki[3], Takayoshi Inoue[2], Noriko Osumi[1] ([1]*Department of Developmental Neuroscience, Tohoku University Graduate School of Medicine*, [2]*Department of Biochemistry and Cellular Biology, National Institute of Neuroscience, National Center of Neurology and Psychiatry*, [3]*Research Institute of Oral Science, Nihon University, School of Dentistry at Matsudo*)

Radial glial (RG) cells, the progenitors in the developing cortex, have long basal processes. We previously showed in mouse that mRNA of *Cyclin D2* (*Ccnd2*), coding a cell cycle regulator, is transported to the basal end-foot of the RG cell, and a short sequence in its 3'UTR (CTE) is sufficient for this transport. In the CTE-deleted mutant mice, generated in this study, *Ccnd2* mRNA was no longer localized in the basal end-feet of the RG cells. The mutant mice had a thinner cortex than wild type, suggesting the importance of *Ccnd2* mRNA transport in corticogenesis. As the CTE is only conserved in placental mammals, *Ccnd2* mRNA was not observed in the basal end-feet of neural progenitors of opossum or chicken. While opossum *Ccnd2* 3'UTR could not direct EGFP reporter mRNA to the basal end-foot in mouse, mouse *Ccnd2* 3'UTR could do so in opossum and chicken neural progenitors, suggesting a conservation of the transport machinery in amniotes. Since basal progenitors are proliferative in placental mammals but not in opossum, CTE-mediated transport of *Ccnd2* mRNA might be critical for producing basal progenitors to make a large cortex of placental mammals. (COI:No)

### PP-10

### TSNARE-1, subcellular localization and function

Harukata Miki[1], Yoshiharu Kido[1], Ruyun Zhou[1], Wataru Nishimura[1], Yasuko Noda[1] ([1]*Dept. Anatomy, Sch Med, Jichi Medical Univ*)

TSNARE-1 (Syntaxin 20) is a Syntaxin, possessing signature SNARE and Syntaxin domains upstream of the C-terminal transmembrane domain. TSNARE-1 also has an unique, 78 amino acid residue TSNARE-1 domain (TS1dom). Although the cellular function has not been reported previously, TSNARE-1 has been statistically implicated in hereditary schizophrenia and bipolar disorder. We have found that mice, rats and rabbits lack the TSNARE-1 locus in their genome and do not express TSNARE-1. However, other mammals such as humans and some other vertebrae have a functional TSNARE-1 locus. Phylogenetically, TSNARE-1 groups with endosomal Stx12 (a.k.a. Stx13 or Stx14) and Stx7. In the cell, we find the TS1dom localized to mitochondria while full length TSNARE-1 and TSNARE-1 lacking the TS1dom (ΔTS1dom) localized to endosomes. We find TSNARE-1 involved in maturing endosomes docking with mitochondria. These results suggest that TSNARE-1 is a Syntaxin localized to endosomes and may function in endosomal fusion with mitochondria. (COI:No)

### PP-11

### Involvement of estrogen receptor beta in the abnormal brain development in fetuses by maternal bisphenol A diglycidyl ether exposure during gestation and lactation

Ikuko Miyazaki[1], Chiharu Nishiyama[1], Ryo Kikuoka[1], Takeru Nagoshi[1], Kyle Quin[1], Nami Isooka[1], Kazumasa Zensho[1], Masato Asanuma[1] ([1]*Dept. of Medical Neurobiology, Okayama Univ. Grad. Sch. of Med., Dent. and Pharmaceut. Sci*)

Bisphenol A diglycidyl ether (BADGE), an epoxy resin, is used for the inner coating of canned food and beverages. BADGE can easily migrate from the containers and become a contaminant. In the previous studies, we reported that maternal BADGE exposure (1.5 mg/kg/day) during gestation and lactation periods could accelerate neuronal differentiation in the fetuses. In this study, we examined involvement of estrogen receptor (ER) beta in the abnormal brain development induced by BADGE exposure. The histological analysis demonstrated the increase in the ER beta-positive signals in the cortex of offspring from BADGE-exposed dams at postnatal day 1. In primary cultured cortical neurons from SD rat embryos at 15-day gestation, a direct BADGE exposure promoted neurite outgrowth and neuronal connection. In addition, the elongation of neurites induced by BADGE treatment was significantly inhibited by ER beta antagonists, ICI182,780 and G15. These data suggest that BADGE exposure can accelerate neuronal differentiation via ER beta-mediated signal pathways. (COI:No)

### PP-12

### The developmental effect of chronic and low concentration of Glyphosate exposure in utero

Shigehisa Satake[1], Ken Futagami[1], Kwong Soon Thomas Tiong[1], Yoko Nomura[2], Yasunari Kanda[3], Sachiko Yoshida[1] ([1]*Toyohashi University of Technology*, [2]*Queens College, the City University of New York*, [3]*National Institute of Health Sciences*)

Environmental chemicals in-utero have potential effects on developmental neurotoxicity. DOHaD hypothesis mentions that harmful chemical exposure during pregnancy might cause a lifelong lasting effect on pregnant mothers and offspring's psychological and physiological health.
Glyphosate (GLY) is the main compound of a broadly applied herbicide that inhibits a shikimate pathway enzyme not existing in animals; however, GLY is frequently questioned for its neurodevelopmental safety.
Previously, we have observed acute Gly-administration of 250 mg/kg gestate day 16 induced the decrease of Purkinje cells, and the increase of microglia at postnatal day 14 (P14). Additionally, chronic Gly-administration of a total of 250 mg/kg (13.8 mg/kg/day) showed similar toxicity of acute exposure. In this study, we observed the effect of lower concentration and chronic exposure of Gly. In acute exposure, 50 mg/kg Gly administration showed little neurotoxicity. Additionally, 1 mg/kg/day (a total of 22 mg/kg) exposure and 0.1 mg/kg/day (a total of 2.2 mg/kg) showed little alteration of cerebellar development. We suggest that these low-level exposures of Gly would have little developmental problems. (COI:No)

### PP-13

### Neurotoxicity of acute exposure of neonicotinoid, Acetamiprid in developing cerebellum

Christine Li Mei Lee[1], Thomas Kwong Soon Tiong[1], Johnny Ademir Lopez[2], Yoko Nomura[2], Yasunari Kanda[3], Sachiko Yoshida[1] ([1]*Toyohashi University of Technology*, [2]*Queens College, the City University of New York*, [3]*National Institute of Health Sciences*)

Environmental chemicals in-utero have potential effects on developmental neurotoxicity. DOHaD hypothesis mentions that harmful chemical exposure during pregnancy might cause a lifelong lasting effect on pregnant mothers and offspring's psychological and physiological health.
Neonicotinoids, a new class of insecticides, mimic the nicotine chemical structure to bind to the nAChR. Due to the difference of nAChR sensitivities between insects and mammals, neonicotinoids have been seen as safer insecticides than organophosphate compounds; however, recently, some reports suggest the developmental neurotoxicity of neonicotinoids. In this study, we exposed Acetamiprid (ACE), a neonicotinoid dissolved in DMSO, orally in different dosages to pregnant rats during gestation day 16, and observed cerebellar development in the offspring. The 40 mg/kg-ACE-exposed pups showed Purkinje Cell (PC) misalignment and excessive folding between lobule V and VI in the cerebellar vermis 14 days after birth (P14), results that were similar to the cerebellum treated with HDAC inhibitors. We suggest that ACE treatment would alter the timing of PC development and circuit formation. (COI:No)

### PP-14

### Myocyte Enhancer Factor 2D (MEF2D) mediates late phase synapse elimination in the developing cerebellum

Honoka Suzuki[1], Myeongjeong Choo[1], Takaki Watanabe[1,3], Kenji Sakimura[2], Naofumi Uesaka[1], Masanobu Kato[1,3] ([1]*Department of Neurophysiology, Faculty of Medicine, The University of Tokyo,* [2]*Department of Animal Model Development, Brain Research Institute, Niigata University ,* [3]*International Research Center for Neurointelligence, The University of Tokyo*)

Synapse elimination during postnatal development is crucial for establishing mature neural circuits. In the cerebellum, each Purkinje cell (PC) is innervated by multiple climbing fibers (CFs) at birth, but all except one are eliminated during the first three postnatal weeks in mice, a phenomenon known as CF synapse elimination. While the activity of PCs plays essential roles in CF synapse elimination, it is unclear how the expression of genes mediating synapse elimination is precisely regulated by the activity of PCs. Here, by combining genetic manipulation in mouse PCs with electrophysiological and morphological analyses in mouse cerebellar slices, we show that a transcription factor, Myocyte Enhancer Factor 2D (MEF2D) is required for CF synapse elimination. PCs lacking MEF2D were innervated by a greater number of CFs from postnatal day 16 to young adulthood compared to control PCs. We also report that MEF2D shares signaling pathway for CF synapse elimination with P/Q-type voltage dependent calcium channel (P/Q-VDCC). These results suggest that proper gene regulation by MEF2D downstream of P/Q-VDCC is essential for CF synapse elimination. (COI:No)

### PP-15

### Effects of prenatal administration of various HDAC inhibitors to rat cerebellar development

Sarasa Matsui[1], Akari Adachi[1], Misaki Iwanaga[1], Yasunari Kanda[2], Sachiko Yoshida[1] ([1]*Toyohashi University of Technology, Applied Chemistry and Life Science Engineering Department,* [2]*National Institute of Health Sciences*)

Prenatal chemical exposure is one of the causes of developmental disorders such as autism spectrum disorder (ASD). Valproate (VPA), a widely used anti-epileptic drug and a well-known ASD inducer, has an HDAC inhibition effect, and we have observed VPA-administrated rat pups showed the alteration of Purkinje cell development and the excess folding in the cerebellum. In this study, we observed the effect of other HDAC inhibitors on cerebellar development. We administrated Vorinostat (SAHA) 50 mg/kg i.p., Entinostat (MS-275) 4.0 mg/kg p.o., or butyric acid (BA) 200 mg/kg p. o. at embryonic day 16 (E16), and observed the cerebellum of pups at postnatal day 7 (P7) or P14. Both SAHA-administrated pups and BA-administrated pups showed the alteration of Purkinje cell development and the excess folding of the cerebellar lobule, whereas MS-275-administrated pups showed a little developmental alteration. BA is a broad effect-HDAC inhibitor like VPA, and SAHA inhibits class I and II HDACs. Because MS-275 inhibits HDAC-1 in class I HDACs selectively, we suggest broad inhibition of HDACs would be required for developmental alteration. (COI:No)

### PP-16

### The possibility of epigenetic alteration due to LPS neurotoxicity

Sharumadhi Veloo[1], Haruko Otsuka[1], Kazunobu Tsunemoto[2], Thomas Kwong Soon Tiong[1], Yasunari Kanda[2], Sachiko Yoshida[1] ([1]*Toyohashi University of Technology, Toyohashi, Japan ,* [2]*National Institute of Health Sciences, Kawasaki, Japan*)

Autism spectrum disorders (ASD) and mental disorders have been on the rise, and cerebellar degeneration is one of the pathological focal points in early ASD. Lipopolysaccharide (LPS), which is present in the outer membrane of Gram-negative bacteria, is a well-known pro-inflammatory factor via binding to Toll-like receptor 4, and may cause mental disorders. We have observed the developmental cerebellar degeneration and behavior of LPS-administrated rat offspring and discussed its pathology.

In this study, we observed the developmental alteration of the LPS-rat cerebellum, which was administrated 100 μg / kg of LPS i.p. in gestation day 16. Some animals were administrated with PBS in the same period as the vehicle. LPS-rat showed high-level expression of inflammatory cytokines, TNFalpha and IL-1 beta at postnatal day 2 (P2), and the decrease of Purkinje cells at P14, which was not observed at P7. Moreover, the LPS-rat cerebellum showed excessive folding of cerebellar V and VI lobes as same as the alteration in HDAC inhibitor-administrated animals. We suggest that some unknown epigenetic alteration with LPS administration would induce developmental neurotoxicity in rat cerebellum. (COI:No)

### PP-17

### neuronal markers-positive but glial cell-markers negative central nervous system cells in culture show cell division

Hiromi Hiruma[1] ([1]*Department of Physiology, Kitasato University School of Medicine*)

This study investigated whether the cultured rodent central nervous system (CNS) neurons can divide. Rat and mouse brain and spinal cord cells were cultured, time-lapse microscopy was used to observe cell division, and immediately after observation, immunocytechemistry was performed. Cell division occurred in neuron-like cells derived from the various CNS regions at all ages from fetus to adult. The mean division interval was 21 h. The divided neuron-like cells were positive for neuronal markers but not for glial cell markers and showed action potentials. The cells identified as neurons by live-cell immunocytochemistry, expressing neuronal cell surface antigen Thy1.1 but not neuronal stem cell surface antigen prominin-1, were dividing. Cell division was also found in neurons of the Thy1-yellow fluorescent protein transgenic mice, which can be identified as differentiated neurons by fluorescence emission. The present study further indicated that some neurons were in cell cycle and showed mitotic figures, DNA precursor incorporation and DNA replication. These results suggest that rodent CNS neurons have the ability of cell division under physiological culture conditions. (COI:No)

### PP-18

### Ndufs4 regulates synaptophysin expression in hippocampus

Subata Shil[1], Takaaki Abe[1], Yoshiteru Kagawa[1], Banlanjo Umaru[1], Yuji Owada[1] ([1]*Grad. Sch. Med., Tohoku Univ., Sendai, Japan*)

Respiratory chain complex I dysfunction in brain cells leads to many neurodegenerative diseases. NADH Dehydrogenase (Ubiquinone) Fe-S protein 4 (Ndufs4) is one of the subunits of complex I and its mutation is associated with Leigh syndrome (LS) in human. Mimicking However, the molecular role of Ndufs4 in neuronal function is still unexplored. In this study, upon confirmation of Ndufs4 in NeuN expressing neurons and GFAP expressing astrocytes, elevated GFAP expression. Was found in *in vivo* hippocampus of Ndufs4-KO mouse, but not changed in *in vitro*. Although there was no change in the number of NeuN expressing neurons in Ndufs4-KO hippocampus, the expression of synaptophysin was decreased. To investigate the detailed mechanism, we silenced Ndufs4 followed by differentiation in Neuro2A cells and found shorter neurite lengths with decreased synaptophysin per length As well as decrease in activity of ERK signalling. These results suggest that Ndufs4 may regulate synaptophysin expression in hippocampus and thus altered synaptoplasticity may lead to astrogliosis. (COI:No)

### PP-19

### Exercise and pharmacological inhibition of histone deacetylase improves cognitive function in normal mice

Ryo Ikegami[1], Mika Kitahara[1], Takahiro Inoue[1,3], Yasunari Kanda[2], Yasuyuki Takamatsu[2], Harukazu Tooyama[2], Hiroshi Maejima[2] ([1]*Grad Health Sci, Hokkaido Univ, Sapporo, Japan,* [2]*Dept Reha Sci, Fac Health Sci, Hokkaido Univ, Sapporo, Japan,* [3]*JSPS*)

Exercise is recognized to prevent cognitive impairment in the elderly and patients with central nervous system disorders. Histone deacetylase inhibitors (HDACis) acetylate histones and enhance gene transcription in epigenetic regulation. Thus, HDACis are expected to be a potent pharmacological treatment for cognitive function. The objective of this study was to examine the interactive effect of pharmacological treatment using HDACis and exercise on cognitive function. ICR mice were divided into four groups based on two factors (HDAC inhibition and exercise). Intraperitoneal administration of an HDACi (1.2 g/kg sodium butyrate, NaB) and moderate exercise (10 m/min, 60 min) were performed 5 days a week for 4 weeks. The novel object recognition test showed that NaB administration improved recognition memory. Notably, the step-through passive avoidance test showed the improvement of learning and memory, specifically in the presence of NaB administration plus exercise. This study showed that repetitive administration of HDACis improves cognitive function and HDACis administration plus exercise has a synergic effect on learning and memory. (COI:No)

### PP-20

### Structural plastic changes of gray matter assessed by voxel-based morphometry and histological analysis of dendritic arborization after internal capsular infarcts in macaque monkeys

Kohei Matsuda[1,2], Kazuaki Nagasaka[3], Junpei Kato[1,2], Noriyuki Higo[1] ([1]*Human Informatics Research Institute, National Institute of Advanced Industrial Science and Technology,* [2]*Graduate School of Comprehensive Human Sciences, University of Tsukuba,* [3]*Institute for Human Movement and Medical Science, Niigata University of Health and Welfare*)

It is not fully understood how secondary neuronal damage in the intact brain regions occurs after a focal stroke. To investigate this, we performed voxel-based morphometry (VBM) using T1-weighted magnetic resonance imaging and immunohistochemical analysis using a macaque model of unilateral internal capsular infarcts we recently developed. The VBM results showed that gray matter volume (GMV) significantly decreased in the primary motor cortex (M1) of the ipsilesional hemisphere, and also changed in several other brain areas at 3 months following infarcts as compared to those before infarcts. We then performed immunohistochemistry using SMI-32 antibody to investigate changes in the extent of dendritic branching in pyramidal neurons in layer V of motor cortical areas where GMV changes were indicated by VBM. The histological analysis indicated a significant decrease in dendritic arborizations in pyramidal neurons in the ipsilesional M1 compared to those in the contralesional and intact M1, suggesting that changes in neuronal structures including dendrites occur in brain regions at a distance from the infarcted area following internal capsular infarcts. (COI:No)

### PP-22

### Role of primary visual cortex and superior colliculus in visual search task in mice

Maki Kimura[1], Kaoru Isa[2], Reona Yamaguchi[3], Tadashi Isa[2,3] ([1]*Department of Medial Science, Faculty of Medicine, Kyoto university,* [2]*Department of Neuroscience, Graduate School of Medicine, Kyoto University,* [3]*Institute for the Advanced Study of Human Biology [WPI-ASHBi], Kyoto University*)

The retino-geniculo-striate pathway to primary visual cortex (V1) is highly important for visual perception. On the other hand, it is known that some patients with damage to the V1 can perform goal directed behavior in spite of denying having a conscious percept. This phenomenon has been called blindsight. Previous anatomical studies suggested that the extrageniculate visual pathway mediated via the superior colliculus (SC) in the midbrain underlies blindsight. However, little is known about to what extent the SC can contribute to visual behavior which requires complex cognitive processes. In this study, we investigated the function of the mouse SC for navigation in the maze task. First, we measured the time to reach the goal in the maze task. After the V1 lesion, the time to reach the goal was slightly prolonged, but recovered in 2-3 days. Then, the SC lesion was added. The time to reach the goal was significantly prolonged in 2-3 days compared with the case of the V1 lesion. These results suggested that the extrageniculate visual pathway via SC can control navigation of the mice in the maze task. (COI:No)

### PP-23

### The regulation of Na/K ATPase activity facilitates dendritic spine formation and motor learning performance in adult mice

Junichi Hashimoto[1], Keisuke Tanimoto[1], Kazumasa Matsumoto-Miyai[1] ([1]*Graduate School of Comprehensive Rehabilitation, Osaka Prefecture University, Japan*)

We have shown that the C-terminal fragment of agrin cleaved by neurotrypsin induced the formation of dendritic spines. Since the C-terminal agrin fragment is reported to regulate the Na/K ATPase activity, we investigated whether intraperitoneal administrations of cardiac glycoside digoxin (1, 4, 65, or 650 μg/kg) influences dendritic spine formation and motor learning in adult mice. The histological analysis using the Golgi-Cox staining revealed that intraperitoneal injection of 65 μg/kg digoxin, which activates Na/K ATPase, significantly increased the density of newly-formed spines in cerebral cortex and hippocampus in comparison to the mock-treated condition. Furthermore, the motor learning performance in the rotarod test were enhanced by the intraperitoneal injection of 4 or 65 μg/kg digoxin into C57BL/6 mice. The neurotrypsin-knockout (NT-KO) mice showed the significant less performance than the wild-type (NT-WT) mice under the mock-treated condition, and low concentration of digoxin improved the performance of NT-KO mice up to the similar degree in NT-WT mice. These results suggest that Na/K ATPase activation could promote motor learning by enhancing spine neogenesis. (COI:No)

### PP-24

### Endotoxin-induced hypoexcitability plasticity of layer 5 pyramidal neurons in the medial prefrontal cortex.

Yuki Yamawaki[1], Michiyo Muramatsu[2], Gen Otsuki[1] ([1]*Department of Drug Discovery Medicine, Kyoto University Graduate School of Medicine,* [2]*Department of Human Health Sciences, Kyoto University Graduate School of Medicine*)

Lipopolysaccharide (LPS), the outer component of Gram-negative bacteria, induces a response of animal innate immunity via microglia. Endotoxin-triggered plasticity of the intrinsic excitability of neurons has been studied in different cell types. However, it is unclear whether the modulation of neurophysiological properties is similar. we recorded firing frequencies of L5 pyramidal neurons under whole-cell patch clamp after exposure to LPS in the medial prefrontal cortex (mPFC) of rats.The frequency of firing for LPS was reduced in L5 pyramidal neurons but increased in cerebellar Purkinje cells.The decrease in excitability of pyramidal neurons by immune activation was abolished under the agonist of small-conductance $Ca^{2+}$-activated $K^+$ channels, SK-channels. This was dependent on intracellular $Ca^{2+}$ and its phosphatase activity from experiments using BAPTA and PP2B inhibitors.An inhibitor of tumor necrosis factor-a (TNF-a), an inflammatory cytokine released from microglia, prevented the excitability decrease by LPS. Therefore, our results suggest that the direction of the excitability changes by microglia are not consistent, depending on the brain region and the cell-type. (COI:No)

### PP-25

### The motor recovery and synaptic plasticity was affected by the types of exercise in the hemorrhage model rat.

Chihiro Sato[1], Kunikazu Tanji[2], Shunichi Kato[1], Kai Hatakenaka[1], Misaki Mikami[1], Shuhei Koeda[1], Junko Yamada[1] ([1]*Dept Comprehenshive rehabilitation, Grad Sch Health Sci, Hirosaki Univ, Aomori, Japan ,* [2]*Dept of Neuropathology, Grad Sch Health Sci, Hirosaki Univ, Aomori, Japan* )

Previously, we reported that the voluntary exercise (V-Ex) has greater recovery than the forced exercise (F-Ex) and the synaptic changes were observed in V-Ex and F-Ex groups than the non-exercise group. However, the factors related the motor recovery were unknown. The purpose of our study is to clarify the mechanisms of recovery with rehabilitation. To determine the factors affected motor recovery, the stress and motivation levels were compared among three groups. The concentration of corticosterone was analyzed to assess the stress level, and was higher in the F-Ex group than the other groups. The expression of ΔFosB in nucleus accumbens was used as a marker of activated neuron in the reward system, and was higher in the V-Ex group than the other groups. In V-Ex group, the running distance increased day by day. To prepare the amount of exercise, the running condition of the F-Ex was adjusted and carried out same as V-Ex group. The adjusted F-Ex. group had less effective than the voluntary exercise.

These data suggested that the synaptic plasticity in motor recovery might be affected by not only the running distance but also the stress or motivation. (COI:No)

### PP-26

### Investigation of the mathematical relationship among components of short-term synaptic plasticity, relationship of augmentation and potentiation with facilitation, at the frog neuromuscular junction

Naoya Suzuki[1] ([1]*Dept Phys, Sch Sci, Nagoya Univ*)

To investigate mechanism of stimulation induced enhancement of transmitter release, we analyzed the short-term synaptic plasticity at the frog neuromuscular junction, specifically, the relationship of augmentation[A] and potentiation[P] with facilitation[F] using four mathematical models having relationships among these components; (1+F)(1+A)(1+P), (1+F+A)(1+P), (1+F)(1+A+P), and (1+F+A+P). Endplate currents (EPCs) were recorded extracellularly with a surface glass microelectrode. Two stimulation patterns, five repeats of 60 stimuli at 20Hz with 2 seconds intervals or five repeats of 100 stimuli at 20Hz with 10 seconds intervals to induce synaptic plasticity. According to the mathematical model described by Zengel and Magleby (J.Gen.Physiol., 1982): constant increments are added to each component instantaneously by each stimulus and each component decays independently with its fixed time constant, both rising (during tetanus) and decaying processes of EPCs were quantitatively analyzed. The multiplicative relationship model, (1+F)(1+A)(1+P), gave the best results to produce the similar calculated (1+F) from each tetanus repeat by deconstruction of A and P components. (COI:No)

### PP-27

### Changes in spine morphology and learning behavior in drebrin knockout mice.

Mai Sawabe[1], Nobuhiko Kojima[1,2] ([1]*Grad Sch of Life Sciences, Toyo Univ, Japan*, [2]*Faculty of Life Sciences, Toyo Univ, Japan*)

Dendritic spine morphology is known to change dynamically with neural activity and related to higher brain functions. It has been suggested that drebrin is a key protein for spine morphogenesis and plasticity. In the previous studies we demonstrated that NMDA receptors (NMDAR) activities triggered transient efflux of drebrin from spines. We also demonstrated that activity-dependent synaptic plasticity was altered in drebrin mutant mice. These results suggest that spine localization of drebrin and glutamate receptor activity interact with each other. We then speculate that changes in spine morphology with synaptic plasticity, and learning behavior, depend on drebrin in dendritic spines. In this study, we investigated morphological and behavioral phenotypes of drebrin knockout (DXKO) mice to delineate the drebrin-dependent interaction between behavior and spine morphology. We found that immature protrusion was significantly increased in the hippocampus in DXKO mice. We also observed elevated anxiety-like behavior and impaired memory task in DXKO mice. We are now investigating whether change in either NMDR or mGluR activity is involved in behavioral phenotypes in DXKO mice. (COI:No)

### PP-28

### Anatomical analysis of neuropeptide Y (NPY) neurons in the nucleus accumbens

Shunji Yamada[1], Takuma Mori[3], Katsutoshi Taguchi[1], Atsushi Tsujimura[2], Masaki Tanaka[1] ([1]*Department of Anatomy and Neurobiology, Graduate School of Medical Science, Kyoto Prefectural University of Medicine*, [2]*Department of Basic Geriatrics, Graduate School of Medical Science, Kyoto Prefectural University of Medicine*, [3]*Department of Molecular and Cellular Physiology Shinshu University, School of Medicine*)

We previously reported that Neuropeptide Y (NPY) neurons in the nucleus accumbens (NAc) are involved in regulation of anxiety behavior. Obtaining more knowledge about NAc NPY neurons, in the present study, we investigated neural output and input of the NAc NPY neurons using NPY-Cre mice. We observed dense mCherry-positive fibers in the lateral hypothalamus (LH) in the AAV-FLEX-mCherry-injected NPY-Cre mice into the NAc. To confirm the projection of NAc NPY neurons to the LH, we injected cholera toxin b subunit (CTb), a retrograde tracer, into the LH in the mice whose NAc NPY neurons labeled by mCherry and found CTb and mCherry double-positive neurons in the NAc. Finally, we investigated neural input to the NAc NPY neurons using a first-infected cell specific rabies virus-monosynaptic retrograde tracing. We found that NAc NPY neurons receive direct synaptic connections from the paraventricular nucleus of the thalamus and amygdala. These results suggest that a neural circuit including NAc NPY neurons projecting to the LH may regulate anxiety behavior. (COI:No)

### PP-29

### Monosynaptic facilitation mediated by group Ia afferents from the extensor carpi radialis to the first dorsal interosseous motoneurons in humans

Mitsuhiro Nito[1], Takuya Yoshimoto[1], Wataru Hashizume[1], Akira Naito[1] ([1]*Department of Anatomy and Structural Science, Yamagata University School of Medicine, Yamagata, Japan*)

This study investigated neural projection from the low threshold afferents of extensor carpi radialis (ECR) to the first dorsal interosseous (FDI) motoneurons in 10 healthy human subjects. Electrical conditioning stimulation to the radial nerve branch innervating ECR with the intensity immediately below the motor threshold was delivered during very weak index finger abduction. In the PSTH study, a total of 41 FDI motor units was examined from 5 subjects. The stimulation produced an early and significant peak (facilitation) of FDI motor unit firings in 15/41 (37%) compared with control situation. Remaining 26 motor units received no effects. Examining the process of central synaptic delay showed that the facilitation is mediated through a monosynaptic path. In the EMG-A study, the stimulation produced an early and significant peak (facilitation) in all the 10 subjects. The facilitation diminished following vibration to the ECR muscle belly and recovered 20-50 minutes after the vibration. These findings suggest that facilitation from ECR to FDI motoneurons exists in humans. Group Ia afferents should mediate the facilitation through a monosynaptic path in the spinal cord. (COI:No)

### PP-30

### Tangential migration contributes to establish tectofugal visual pathway in the developing avian optic tectum

Yuji Watanabe[1], Chie Sakuma[1], Hiroyuki Yaginuma[1] ([1]*Neuroanat.Embryol., Fac. Med., Fukushima Med. Univ*)

Human visual pathway consists of geniculate and extra-geniculate pathway. Extra-geniculate pathway from the retina transfers toward the associated visual cortex via superior colliculus and pulvinar to pursuit eye movement for tracking moving objects. In birds, the latter pathway carries both image recognition and visual movement, which is called tectofugal pathway via tectum and the nucleus rotundus.
We have previously demonstrated that the tangentially migrating cells in the developing chick optic tectum differentiate into SGC neurons in tectal layer 13, which have large dendritic fields and send an axon to the nucleus rotundus. In this study, we report that these SGC neurons comprise tectofugal visual pathway. Using recombinant vesicular stomatitis virus, we trans-synaptically labelled retino-tectal and tecto-rodundal projections constituting chick tectofugal visual pathway. Another labeling of SGC neurons after tangential migration coincided with the labeling of the tectofugal pathway. The result suggests that tangential migration in early optic tectum is the key developmental process for arranging SGC neurons to form tectofugal visual pathway eventually. (COI:No)

### PP-31

### Genetic dissection of the mouse habenular subnuclei by retrograde viral vector

Wanqin Tan[1], Miho Matsumata[1], Tomomi Aida[2], Harumi Ishikubo[2], Takako Usami[3], Kohichi Tanaka[2], Hidenori Aizawa[1] ([1]*Grad Sch Biomed Health Sci, Hiroshima Univ.*, [2]*Lab Mol Neurosci, Med Res Inst, Tokyo Med Dent Univ*, [3]*Lab Recomb Animals, Med Res Inst, Tokyo Med Dent Univ*)

Habenula consists of the medial and lateral habenula and can be further divided into more than 15 subnuclei. Brn3a is a POU-domain transcription factor which is essential for the identity and survival of the habenular neurons. To label a subpopulation of neurons according to the distinct neural connectivity, we developed Brn3a-IRES-Flp mouse line which labeled all the habenular neurons. Indeed, intra-habenular injection of AAV-Syn-fDIO-ChR2-EYFP labeled the projection of the habenular neurons targeting to the multiple brain regions such as interpeduncular nucleus (IPN) and ventral tegmental area. To limit the genetic labeling to a subpopulation of the habenular neurons, we generated mice carrying Brn3a-IRES-Flp; Rosa-loxP-STOP-loxP-FRT-STOP-FRT-EGFP-TeNT which expressed GFP fused with tetanus neurotoxin dependent upon Cre recombinase. Injection of the retrograde vector AAVretro-Syn-mCherry-Cre to the IPN of this mouse led to the specific expression of EGFP-TeNT in the medial habenula projecting to the IPN where the presynaptic Vamp2 was reduced. These results provided a proof-of-concept to the genetic labeling of the multiple pathways embedded in the habenula. (COI:No)

### PP-32

### A new function of hypothalamic neurons-Defensive behavior to potential threat-

Noriko Horii[1], Kensaku Nomoto [2,4], Takefumi Kikusui[2], Akihiro Yamanaka[3], Mayumi Nishi[1] ([1]*Anat. Cell Biol., Nara Med. Univ.*, [2]*Companion Animal Res. Sch. Veterinary med. Azabu Univ.*, [3]*Dept. Neurosci. II, Res. Inst. Env. Med., Nagoya Univ.*, [4]*Dept. Physiol. Dokkyo Med. Univ*)

Defensive behaviors are evolved responses to threat stimuli, and a potential threat elicits risk assessment (RA) behavior. However, neural mechanisms underlying RA behavior are hardly understood. Urocortin-3 (Ucn3) is a member of corticotropin-releasing factor peptide family and here, we report that Ucn3 neurons in the hypothalamic perifornical area (PeFA) are involved in RA of a novel object, a potential threat stimulus, in mice. Histological and *in vivo* fiber photometry studies revealed that the activity of PeFA Ucn3 neurons was associated with novel object investigation involving the stretch-attend posture, a behavioral marker for RA. Chemogenetic activation of these neurons increased RA and burying behaviors toward a novel object without affecting anxiety and corticosterone levels. Ablation of these neurons caused the abnormal behaviors of gnawing and direct contacts with novel objects, especially in a home-cage. These results suggest that PeFA Ucn3 neurons modulate defensive responses to a potential threat stimulus. (COI:No)

### PP-33

### Projection from the oval paracentral nucleus in the intralaminar thalamic nuclei to the cerebral cortex in the rat.

Yumi Tsutsumi[1], Yuka Mizuno[1], Fumihiko Sato[1], Misaki Inoue[1], Yayoi Morita[1], Takahiro Furuta[1], Atsushi Yoshida[1] (*[1]Department of Oral Anatomy and Neurobiology, Graduate School of Dentistry, Osaka University*)

The oval paracentral nucleus (OPC) is isolated from the paracentral nucleus (PC) in the rat intralaminar thalamic nuclei. We have demonstrated that proprioceptive sensation arising from rat jaw-closing muscle spindles is conveyed to the OPC. To reveal the OPC-cortex projections, we performed three experiments using rats. (1) We injected an anterograde neural tracer BDA into the electrophysiologicaly identified OPC. Axons and terminals were labeled in the primary (S1) and secondary (S2) somatosensory cortex and the granular insula cortex (GI). (2) We injected BDA in the caudal PC. BDA labeling appeared in the medial and lateral agranular cortices. (3) We injected a retrograde neural tracer FG in the S1, S2 and GI where the BDA-labeled axons from the OPC were observed in (1). Among the intralaminar and sensory thalamic nuclei, only the OPC sent strong projections to all of the three cortical areas. In conclusion, the cortical projection features were different between the OPC and the other intralaminar and sensory thalamic nuclei. The proprioceptive sensation conveyed by the OPC-cortex pathway may be involved in sensory discrimination and integration. (COI:No)

### PP-34

### Molecular mechanism of axon collateralization regulated by receptor protein tyrosine phosphatase

Misato Yasumura[1], Tokuichi Iguchi[1], Quynh Mai Nguyen[1], Makoto Sato[1,2] (*[1]Dept Anat & Neurosci, Grad Sch Med, Osaka Univ, [2]Div Dev Nueosci, United Grad Sch Child Dev, Osaka Univ*)

Neurons in various brain regions form axon collateral branches, which enable a single neuron to connect to multiple targets, and mediate synchronization of neuronal activity. The corticospinal neurons project to various subcortical targets including the basilar pons via axon collateral branches. Developmental profile of the axon collateral formation to the basilar pons has been intensively investigated, however, the molecular mechanisms regulating the formation and stabilization of axon collaterals are poorly understood. To identify molecules that are essential for axon collateralization of the corticospinal neurons, we screened candidate receptors expressed in the corticospinal neurons by knocking down them one by one. We found that knockdown of one of the receptor protein tyrosine phosphatases (RPTPs) in the corticospinal neurons significantly suppressed the axon collateral formation to the basilar pons. This suppression was rescued by cytoplasmic phosphatase domains-deleted RPTP mutant or substrate-trapping RPTP mutant. These results suggest that RPTP regulates axon collateralization in phosphatase activity-independent manner. (COI:No)

### PP-35

### A prefrontal–paraventricular thalamus circuit requires juvenile social experience to regulate adult sociability

Kazuhiko Yamamuro[1,2], Minobu Ikehara[1], Hirofumi Morishita[2], Yasuhiko Saito[3], Toshifumi Kishimoto[1] (*[1]Department of Psychiatry, Nara Medical University, [2]Department of Psychiatry, Icahn School of Medicine at Mount Sinai, [3]Department of Neurophysiology, Nara Medical University*)

Juvenile social isolation reduces sociability in adulthood, but the underlying neural circuit mechanisms are poorly understood. We found that, in male mice, 2 weeks of social isolation immediately following weaning leads to a failure to activate medial prefrontal cortex neurons projecting to the posterior paraventricular thalamus (mPFC→pPVT) during social exposure in adulthood. Chemogenetic or optogenetic suppression of mPFC→pPVT activity in adulthood was sufficient to induce sociability deficits without affecting anxiety related behaviors or preference toward rewarding food. Juvenile isolation led to both reduced excitability of mPFC → pPVT neurons and increased inhibitory input drive from low threshold spiking somatostatin interneurons in adulthood, suggesting a circuit mechanism underlying sociability deficits. Chemogenetic or optogenetic stimulation of mPFC→pPVT neurons in adulthood could rescue the sociability deficits caused by juvenile isolation. Our study identifies a pair of specific medial prefrontal cortex excitatory and inhibitory neuron populations required for sociability that are profoundly affected by juvenile social experience. (COI:No)

### PP-36

### Massive re-routing of corticospinal projection after functional recovery from spinal cord injury in the macaque monkey

Satoko Ueno[1], Reona Yamaguchi[2], Kaoru Isa[1], Toshinari Kawasaki[1,3], Masahiro Mitsuhashi[1,4], Tadashi Isa[1,2] (*[1]Dept Neuroscience, Grad Sch Med, Kyoto Univ, Kyoto, Japan, [2]Institute for the Advanced Study of Human Biology [WPI-ASHBi], Kyoto Univ., Kyoto, Japan, [3]Dept Neurosurgery, Grad Sch Med, Kyoto Univ, Kyoto, Japan, [4]Dept Neurology, Grad Sch Med, Kyoto Univ, Kyoto, Japan*)

Developed hand movements of higher primates are considered to be controlled primarily by the corticospinal tract (CST). Once the CST is damaged, the motor function is impaired, and rebuilding the same neuronal network as before the injury is difficult. However, it is known that intense rehabilitation has some effect on the recovery after partial spinal cord injury (SCI) with damage to the CST. This indicates that the morphological reorganization might have occurred in the CST to compensate for the impaired motor functions.
In this study, we investigated the plastic change in CST of macaque monkeys after the rehabilitative training after sub-hemisection of the spinal cord, in which coarse power grip recovered, although precision grip did not. Normally, majority of the CST fibers from the motor cortex cross at the pyramidal decussation. However, in this case, we found that a massive number of CST fibers originating from the forelimb area of the primary motor cortex on the contralesional side did not cross at the level of decussation. These uncrossed CST fibers might have been induced during the rehabilitation and contributed to the recovered motor function after sub-hemisection. (COI:No)

### PP-37

### Visualizing of input-output architecture of orexin neurons by retrograde tracing vectors

Yuki Saito[1], Takeshi Sakurai[1] (*[1]WPI-IIIS, University of Tsukuba*)

The lateral posterior part of the hypothalamus contains neuronal populations implicated in maintenance of arousal, including orexin-producing neurons (orexin neurons) in the lateral hypothalamic area (LHA).Orexin neurons send widespread projections to nuclei containing monoaminergic neurons such as locus coeruleus (LC), tuberomamillary nucleus (TMN) and raphe nuclei (Nambu, et al., 1999). We previously identified input neurons that send direct synaptic contact to orexin neurons with modified rabies vector-based retrograde tracing (Saito, et al., 2018). This study showed orexin neurons receive input from many regions of the brain. It is unknow whether different orexin neurons that send projections to particular regions receive differential input.
In this study, we first generated Orexin-iCre Knock-in mice. We used this line to analyze the input-output relationship of orexin neuronal circuits using modified multi-color cTRIO method. This study revealed that orexin neurons projecting to each output region also project to all brain regions we examined. Orexin neurons integrates information from broad regions of the brain and broadcasts to all monoaminergic nuclei and other regions. (COI:No)

### PP-38

### Axonal projection to the lumbar cord from the mouse primary sensorimotor cortex

Hiroshi Kameda[1], Naoyuki Murabe[1], Hiroaki Mizukami[2], Keiya Ozawa[3], Toshihiro Hayashi[1], Masaki Sakurai[1] (*[1]Dept Physiol, Teikyo Univ Sch Med, Tokyo, Japan, [2]Div Genetic Therap, Ctr Molecular Medicine, Jichi Medical Univ, Tochigi, Japan, [3]Div Genetic Therap, Inst Med Sci, Univ of Tokyo, Tokyo, Japan*)

The corticospinal (CS) neurons have been reported to distribute not only in motor-related cortical areas, but also in somatosensory areas in many species of mammals. We have shown that, in mice, axons from the primary motor (M1) and somatosensory (S1) cortices innervating the cervical cord were distributed on its specific division in the cervical cord gray matter: (1) M1 projects mainly to the intermediate and ventral areas, (2) the rostral part of S1 to the mediodorsal area, and (3) the caudal part of S1 to the dorsolateral area. Thus, we next investigated the axonal projection from a cortical region innervating the lumbar cord. The region also contains M1/S1, but is relatively small compared to that innervating the cervical cord. We injected adeno-associated virus vectors expressing fluorescent proteins into cortical sites separately to cover the region, and observed labeled axons in the lumbar cord gray matter. We found that there was a relation between locations of CS neurons and distribution patterns of their axons. The structure of topographic connections was basically similar to those observed in the analysis of cervical-cord-innervating CS neurons. (COI:No)

### PP-39

### Exploring the neuronal projections to the nucleus of the solitary tract activated by high-intensity exercise

Kei Tsukioka[1,2], Ko Yamanaka[1], Hidefumi Waki[1] ([1]*Department of Physiology, Grduate School of Health and Sports Science, Juntendo University, Japan,* [2]*JSPS Research Fellow*)

Proper cardiovascular regulation during high-intensity exercise is important to maintain performance. The nucleus of the solitary tract (NTS) is known to be a cardiovascular center and is connected to various brain regions. Although many brain regions exhibit prominent activation during high-intensity exercise, the neuronal regions which signal the NTS remain unclear. In this study, we attempted to answer this question using double immunostaining methods of retrograde tracing and c-Fos expression during high-intensity exercise. First, we injected a retrograde tracer (Cholera toxin β subunit; CTb) into the NTS of rats. After one week of survival, an incremental exercise test was conducted, and brain tissue was extracted for c-Fos immunostaining. We explored and confirmed the co-localization of cells expressing c-Fos and CTb in several regions, including the central nucleus of the amygdala and paraventricular nucleus of the hypothalamus. These regions may be involved in cardiovascular regulation during high-intensity exercise via NTS.
(COI:No)

### PP-40

### Distinct projection of anterior cingulate cortex layer 2/3 and 5 neurons

Yaolong Li[1], Kotaro Mizuta[1], Yasunori Hayashi[1] ([1]*Graduate School of Medicine, Kyoto University* )

The anterior cingulate cortex (ACC) plays important roles in multiple functions such as emotion controlling, decision making, and spatial navigation. ACC connects with multiple brain regions such as dorsal-medial striatum (dmSTR) and dysgranular retrosplenial cortex (RSCd). All of these regions are involved in different functions. For example, RSCd is important in spatial navigation whereas dmSTR relates to decision making. It raises the question whether the same neuron in ACC process different information and projects to multiple brain regions. To figure out this question, we performed double labeling of the projection neurons experiment in ACC by introducing the retrograde tracers AAV2-retro-Syn-EGFP and AAV2-retro-CAG-tdTomato into dmSTR and RSCd, respectively. We found that ACC layer 2/3 neurons preferentially project to dmSTR, whereas layer 5 neurons preferentially project to RSCd. By immunostaining with antibody anti-CaMKIIa and ani-GAD67, we revealed that most of these projection neurons were excitatory neurons. Therefore, it raises the probability that multiple information processed in ACC and transfer to distinct brain regions. (COI:No)

### PP-41

### Identification of brain regions where the general anesthetics sevoflurane and propofol induce neural activation using c-Fos as a marker of neuronal activity

Nobutaka Kamei[1], Shinpei Higo[2], Hitoshi Ozawa[2] ([1]*Dept. Anesthesiology and Pain Medicine., Grad. Sch. Med., Nippon Med. Sch., Tokyo, Japan,* [2]*Dept. Anat. Neurobiol., Grad. Sch. Med., Nippon Med. Sch., Tokyo, Japan*)

**Background**: General anesthetics are frequently used in surgery, but the basic mechanism of action, especially action site, remains unclear. This study aims to identify the brain regions activated by anesthetics to elicit neural activity, in order to elucidate the mechanisms of action and side effects of anesthetics.
**Methods:** Male Wistar rats (8-10 weeks old) were anesthetized for 1 hour with either sevoflurane or propofol. Then brain sections were prepared for *in situ* hybridization and immunohistochemistry and the number of c-Fos (+) cells were assessed.
**Results:** Sevoflurane significantly increased the number of c-Fos (+) cells in the islands of Calleja, central amygdala, medial habenular nucleus, medial vestibular nucleus and solitary tract nucleus. Propofol showed a significant increase in the number of c-Fos (+) cells in lateral habenular nucleus and medial vestibular nucleus.
**Discussion:** Differences in the brain regions activated by the two general anesthetics may lead to differences in the characteristics of anesthesia, including mechanism of action and incidence of adverse effects. (COI:No)

### PP-42

### Cellular expression and subcellular localization of diacylglycerol kinase γ in rat brain

Yasukazu Hozumi[1], Tomoyuki Nakano[2], Kaoru Goto[2] ([1]*Grad. Sch. Med., Akita Univ., Akita, Japan,* [2]*Sch. Med., Yamagata Univ., Yamagata, Japan*)

Diacylglycerol kinase (DGK) phosphorylates diacylglycerol to produce another second messenger phosphatidic acid. Of the DGK family, DGK is predominantly expressed in the brain at the mRNA level. Recent studies have shown the expression of DGK γ in vascular endothelial cells and adrenal medullary cells at the protein level, although its detailed cellular expression pattern and subcellular localization in the brain remain to be determined. In the present study, we addressed this point using specific DGK γ antibody. DGK γ was expressed in both projection neurons and interneurons in the cerebral cortex, hippocampal formation, and cerebellum. In cerebellar Purkinje cells, DGK γ was distributed to the soma and dendrites. Fractionation study revealed that DGK γ is enriched in the internal membranes containing the endoplasmic reticulum and Golgi complex. In immunoelectron microscopy, DGK γ was localized throughout the smooth endoplasmic reticulum system. These findings suggest that DGK γ shows unique cellular expression pattern in the brain and distinct subcellular localization different from other DGK isozymes.
(COI:No)

### PP-43

### Distribution of neurotransmitter agents in postganglionic neurons of the human middle cervical, superior cervical and stellate ganglion

Tadasu Sato[1], Takehiro Yajima[1], Hiroyuki Ichikawa[1] ([1]*Division of Oral and Craniofacial Anatomy, Graduate School of Dentistry, Tohoku University* )

The middle cervical ganglion (MCG), one of cervical sympathetic ganglia is located between superior cervical (SCG) and stellate ganglia (SG). Distributions of neurotransmitter agents have been demonstrated in the human SCG and SG. However, little is known about them in the human MCG. In this study, immunohistochemistry for neurotransmitters and their related substances was performed on the MCG in human cadavers. In 4 samples of human cadavers, MCG swellings contained numerous sympathetic postganglionic neurons. In another sample, a distinct swelling of the MCG could not be detected. However, neuronal cell bodies were present within the sympathetic nerve trunk between the SCG and SG.
In the MCG, SCG and SG, the majority of postganglionic neurons were immunoreactive for dopamine β-hydroxylase, tyrosine hydroxylase and neuropeptide Y whereas few of them contained vasoactive intestinal polypeptide (VIP). VIP-immunoreactivity was also expressed by nerve fibers surrounding some postganglionic neurons in the MCG, SCG and SG. Like in the human SCG and SG, catecholamines, neuropeptides probably act as neurotransmitter substances in the human MCG. (COI:No)

### PP-44

### Expression of type1 vomeronasal receptors in the olfactory organ of lungfish

Shoko Nakamuta[1,2], Masato Nikaido[3], Yoshio Yamamoto[1], Nobuaki Nakamuta[1] ([1]*Vet Anat., Iwate Univ.,* [2]*Dep. Physiol., Iwate Med Univ.,* [3]*Sch. Life Sci. Tech., Tokyo Tech*)

Lungfish is the closest fish to amphibians, and their olfactory organ consists of two sensory epithelia: the lamellar olfactory epithelium (OE) and the recess epithelium (RecE). Based on the fine structure of olfactory receptor neurons, it has been suggested that the lamellar OE is a fish-type OE, whereas the RecE corresponds to the vomeronasal organ of tetrapod. Previous reports have also demonstrated the expression of molecules involved in the signal transduction of olfactory receptors. However, functional properties of lungfish olfactory organ is still unknown. In the present study, we analyzed the expression of type 1 vomeronasal receptors (V1Rs) to infer the olfactory function of each sensory epithelium in the olfactory organ of lungfish. By in situ hybridization, cells expressing V1Rs were found both in the lamellar OE and RecE. Furthermore, the density of V1R-cells was higher in the lamellar OE than in the RecE. Present results suggest that the two sensory epithelia in the olfactory organ of lungfish have distinct functions from each other. (COI:No)

### PP-45

### Promotion of adult hippocampal neurogenesis by memantine via enhancement of chondroitin sulfate proteoglycan expression in middle-aged mice

Jun Yamada[1], Shoichiro Maeda[1], Kyoko Iinuma[1], Shozo Jinno[1] ([1]*Grad Sch of Med., Kyushu Univ*)

We have recently reported that chondroitin sulfate proteoglycans (CSPGs) may play an essential role in the regulation of adult hippocampal neurogenesis. Memantine, an open-channel blocker of NMDA receptors, is widely used in the treatment of Alzheimer's disease. Because several studies have reported that memantine may promote the hippocampal neurogenesis, we aimed to elucidate the potential effects of memantine on CSPGs and neurogenesis. Behavioral testing showed that memantine improved the long-term memory of middle-aged mice. Double fluorescence for parvalbumin (PV) and *Wisteria floribunda* agglutinin (WFA) showed that memantine did not affect the densities of WFA+ perineuronal nets around PV+ GABAergic neurons in the Ammon's horn and dentate gyrus. However, fluorescence intensities for WFA were increased by memantine in the dentate gyrus, but not in the Ammon's horn. Interestingly, gene expression of matrix metalloproteinases (MMPs), a n enzyme for CSPG degradation, was decreased in the dentate gyrus of memantine-treated mice. These findings indicate that memantine may improve the long-term memory via promotion of CSPG expression and adult hippocampal neurogenesis. (COI:No)

### PP-46

### Immunohistochemical relationships of HOME cells with Calbindin- and GnRH-immunoreactive neurons in the mouse accessory olfactory system during pre- and neonatal stages.

Koh-hei Masumoto[1], Kosei Yonezawa[1], Maho Iida[1], Kenji Nakajima[1], Md Nabiul Islam[1], Kanako Nozaki[1], Akie Yanai[1,2], Koh Shinoda[1] ([1]*Division of Neuroanatomy, Department of Neuroscience, Yamaguchi University Graduate School of Medicine,* [2]*Department of Basic Laboratory Sciences, Faculty of Medicine and Health Sciences, Yamaguchi University Graduate School of Medicine*)

Huntingtin-associated protein 1 (HAP1) is a polyglutamine length-dependent interactor with causal agents in several neurodegenerative diseases and has been regarded as a protective factor against neurodegeneration. In our previous study, we clarified the presence of HAP1-immunoreactive (ir) cells migrating from the accessory olfactory system to the forebrain during the mouse pre- and neonatal stages, and coined them as "HAP1-immunoreactive olfactory migrating embryonic (HOME) cells". GnRH-ir and Calbindin-ir neurons are also known as migrating cells. However, the relationships between HOME cells with Calbindin-ir neurons and GnRH-ir neurons have never been examined. Using immunohistochemistry, in this study, we clarified the relationships among these three cell types. Most of the HOME cells contained Calbindin-immunoreactivity in the mouse accessory olfactory system. In addition, all most all the GnRH-positive neurons contained HAP1-immunoreactivity. Taken together, HAP1 may protect migrating cells from certain stresses during migration process and it is possible that lack of HAP1 may be implicated in some clinical disorders related to the HOME cells, particularly GnRH dysfunction. (COI:No)

### PP-47

### Neuroprotective effects of STB/HAP1 against neuronal degeneration induced by proteasomal inhibition in adult mice.

Kanako Nozaki[1], Fuko Hamasaki[1], takumi Nakashima[1], Hazuki Tanaka[1], Md Nabiul Islam[1], Akie Yanai[1], Koh-hei Masumoto[1], Koh Shinoda[1] ([1]*Division of Neuroanatomy, Yamaguchi University, Yamaguchi, Japan*)

Huntingtin-associated protein 1 (HAP1) is a core molecule of the stigmoid body (STB), a spherical- to oval-shaped, non-membrane-bound inclusion. It has been reported that STB/HAP1 in cultured cells significantly inhibits the apoptosis induced by proteasomal inhibition, where HAP1-immunoreactive STB also changes its morphology from dot like structure to perikaryal reticulogranular clump (PRGC). To answer the question, whether PRGC can also be observed *in vivo*, we performed detailed histological analysis using the brain of WT and HT mice that were intracerebrally injected with proteasomal inhibitor lactacystin. We detected degenerative acidophilic neuronal cells in Jade-c staining analysis, suggesting that the degenerative neuronal cells were substantially increased in the HT mice brain than that in WT mice. In addition, we also observed PRGC-like HAP1-immmunoreactive structures in the injection site of WT mice, but not in HT mice. Taken together, our present results may suggest that STB/HAP1 is an endogenous protective factor *in vivo* that can inhibit the cell death caused by the deterioration of the ubiquitin-proteasome system. (COI:No)

### PP-48

### Elucidation of neurochemical phenotypes of huntingtin-associated protein 1-immunoreactive cells in the submucosal ganglia of the mouse small intestine

Abu Md Mamun Tarif[1], Md Nabiul Islam[1], Mir Rubayet Jahan[1], Akie Yanai[1], Kanako Nozaki[1], Koh-hei Masumoto[1], Koh Shinoda[1] ([1]*Div. Neuroanatomy, Grad. Sch. Med., Yamaguchi Univ., Japan*)

Huntingtin associated protein 1(HAP1) is a neurocytoplasmic component of stigmoid body. Areas with abundant expression of HAP1 in brain and spinal cord remains protected while lack of HAP1 are prone to neurodegeneration. We have recently reported that HAP1 was present in the excitatory/inhibitory motor neurons and interneurons in myenteric ganglion of enteric nervous system (ENS) outside the central nervous system. HAP1 expression and its neurochemical phenotypes in submucosal ganglion of ENS are still unknown. In the present study, we aimed to clarify the expression and neurochemical characterization of HAP1 in the submucosal ganglia of the mouse small intestine. We found that HAP1 was uniformly distributed in the Meissner's plexus and almost all HAP1-immunoreactive cells were co- expressed with cholinergic secretomotor neurons containing ChAT/ CGRP/ somaostatin/calretinin or non-colinergic secretomotor neurons containing VIP/TH/ calretinin and vasodilator neurons containing VIP/calretinin. Our current study is the first to clarify that HAP1 is expressed in all type of cells in submucosal plexus and might be regarded as a novel immunohistochemical marker for submucosal neurons. (COI:No)

### PP-49

### Heat Shock Factor 1 is required for the activation of cellular factor XIII-A as well as the induction of Heat Shock Proteins in the early stages of fish optic nerve regeneration

Kayo Sugitani[1], Ayano Konno[1], Minami Maeda[1], Kazuhiro Ogai[2], Yoshiki Kooriyama[3] ([1]*Div Health Sci, Grad Sch Med Sci, Kanazawa Univ, Kanazawa, Japan ,* [2]*AI Hospital/Macro Signal Dynamics Res. Develop. Center, Kanazawa Univ.,* [3]*Faculty of Pharm, Suzuka Univ. of Med Sci.* )

Fish optic nerve can regenerate their axons and restore their visual functions after nerve injury. Cellular factor XIII A (cFXIII-A) mRNA is rapidly upregulated in the retina 1 day after zebrafish optic nerve injury (ONI). Such a cFXIII-A mRNA showed a short length type which started from the region just after the two putative heat shock factor 1 (HSF-1) binding sites. Chromatin immunoprecipitation provides direct evidence of enrichment of cFXIII-A genomic DNA bound with HSF-1. Generally, HSF-1 contribute to cell homeostasis by synthesizing heat shock proteins (HSPs) to protect cells against various stresses. We focused on the changes of HSF1, HSPs and cFXIII-A in damaged retina within 24 hours after ONI using the zebrafish optic nerve crushed models. The expression of HSF-1 and cFXIII-A mRNA started to increase 0.5 hours after ONI in the retina, with a peak increase at 6 hours. Regarding HSP25, HSP60, HSP70, and HSP90, which are the original targets of HSF-1, the peak expression levels were also observed at 6 hours after ONI.
These results suggest that HSF-1 is involved in the regulation of not only HSPs but also cFXIII-A activation in very early stage of optic nerve regeneration. (COI:No)

### PP-50

### Heat stress-induced cognitive dysfunction is caused by changes in the gut microbiota

Ryota Kato[1], Kenjiro Sato[2], Reo Ishii[3], Nobuhiko Kojima[1,2] ([1]*Grad Sch of Life Sciences, Toyo Univ, Japan ,* [2]*Res Ctr for Biomed Eng, Toyo Univ,* [3]*Sch of Life Sciences, Toyo Univ, Japan*)

Global warming increases the risk to health problems. Heat stroke patients have been reported to cause cognitive impairment, but the details are unknown. It has been reported that gut microbiota (GM) can affect cognitive functions and GM is altered by heat stress. Therefore, we hypothesized that heat stress-induced cognitive decline is caused by changes in the GM. To test this hypothesis, we performed a novel object recognition test in heat-stressed mice. We found that after a 30-min exposure of heat stress (45 ± 2°C) every 12 hours for 4 weeks, mice showed overt memory impairment. We also found that change was seen in the GM of heat-stressed mice as compared to that of non-heat-exposed mice. In order to clarify the causal relationship between memory impairment and changes in GM in heat-stressed mice, we performed transplantation of the fecal lysate prepared from heat-stressed mice into non-heat-stressed mice whose GM were disrupted by antibiotics. We found that mice in which the fecal lysate from heat-stressed mice was transplanted tended to exhibit a memory impairment. These results suggest that heat stress-induced cognitive impairment is in part attributable to changes in GM. (COI:No)

### PP-51

### Improvement of Mn-MRI by Ryanodine receptor antagonist Dantrolene

Akio Inoue[1], Yuriko Inoue[2], Hiromitsu Ezure[2], Naruhito Otsuka[2], Chika Sawa[2], Yoshinobu Manome[3], Koichi Shiraishi[4] (*[1]Human Brain Res Cent, Grad Sch Med, Kyoto Uni, [2]Dep Anat, Syowa Univ, Sch Med, [3]Dev Mol Cell Biol, Res Cent Med Sci,Jikei Uni Med, [4]Div Med Eng,Jikei Univ Med*)

As nerve cells uptake Mn ions through Ca channel depending on nerve activity and Mn ions induce the increase of T1 signal of MRI, Mn-MRI is used to monitor the brain activity in vivo. We studied uptake of Ca and Mn ions by cultured Hippocampal neurons using fluorescent Ca indicator Fluo4. When nerve cells were activated by glutamate, Mn ions entered into nerve cells with Ca ions. After Mn ion uptake, the glutamate activation induced the release of Mn ions from vesicles induced by the Ca induced manner. When Mn ions charged cells were treated several timed with glutamate, Mn ions inside the cells disappeared. Ca and Mn ions are considered to be released from vesicles through Ryanodine receptor, RyR. RyR antagonist, Dantrolene, reduced the increase of cellular Ca ions after glutamate activation. We prevented the release of Mn ions from the vesicles by Dantrolene, and measured the high quality Mn-MRI using Bruker 9.4T MRI machine with cryoprobe. Mn ions were retained by Dantrolene. However, stress-induced or itch and pain induced Mn uptake was not affected by Dantrolene treatment.
I have no COI with regard to the presentation. (COI:No)

### PP-52

### Effects of hypoglossal nerve resection on motoneuron death

Nanae Fukushima[1], Norimi Sumitomo[1], Ayata Nagira[1], Yuko Ichinose[1], Akira Kakegawa[1] (*[1]Dept. Anat., Shinshu Univ. Sch. Med*)

We investigated the total number of hypoglossal (XII) motoneurons that received varying degrees of resection of the XII nerve and evaluated the changes over time after very severe nerve injury by long nerve resection in adult rats. Various lengths of nerve gaps (0.0–13.3 mm) were made of the unilateral XII nerve, and the total number of XII neurons on the injured and uninjured sides was counted after 12 weeks. Moreover, a 9-mm section was resected, and the number of XII neurons were counted at 4, 8, and 12 weeks after the nerve resection. The total number of XII neurons decreased after various lengths of nerve resection, and survival rates ranged from 34.4% to 87.1%. Statistically significant negative correlations were observed between increasing length of the resected nerve and decreasing XII neuron survival. The mean rates of surviving neurons at 4, 8, and 12 weeks after the 9-mm-length resection were 83.5 %, 73.9 %, and 61.1 %, respectively. It was concluded that survival rates of XII neurons after the nerve resection were related to distances between resected nerve stamps and that extensive nerve resection led to slow cell death of the injured neurons. (COI:No)

### PP-53

### Neuronal intake of the albumin-binding dye Evans blue in the rat brain

Sawako Hamasaki[1], Takao Mukuda[1], Shotaro Kume[2], Yuka Koyama[1], Toshiyuki Kaidoh[1] (*[1]Anat., med., Tottori Univ., Yonago, Japan, [2]Life Sci., med., Tottori Univ., Yonago, Japan*)

Recently we found a possibility that neurons in the hippocampus (HIP) can constitutively receive blood-borne proteins although the HIP is thought to be impermeable to blood proteins because of the blood–brain barrier (BBB) integrity. When a fluorescent dye Evans blue (EB), immediately binds to plasma protein albumin, was injected intravenously in the rats, the dye was incorporated into interneurons in the hippocampal dentate gyrus. The present study investigated the effect of various stimuli on neuronal intake of EB in the HIP and other brain areas of physiologically normal rats. When angiotensin II (Ang II), plays as a vasoconstrictor and growth factor, was given into blood to transiently elevate plasma level within the physiological range, the number of EB-positive interneurons tended to increase compared with that in controls. In addition, Ang II evoked neuronal EB-incorporation in the retrosplenial region and amygdala. Spatial learning using the Barnes maze task induced EB-incorporation into not only interneurons but also granule cells in the dentate gyrus. We discuss the mechanisms for neuronal EB-incorporation in these regions from the viewpoint of the BBB vulnerability. (COI:No)

### PP-54

### Expanding the repertoire of the genetically-encoded fluorescent transmitter sensors

Daisuke Ino[1] (*[1]Grad. Sch. Med., Dpt. Histol., Kanazawa Univ*)

In the multicellular system, a variety of signaling molecules are supposed to mediate cell-to-cell communication. However, the information on their spatio-temporal dynamics remains largely elusive. Since fluorescence imaging is a powerful approach to monitor the dynamics of signaling molecules, I decided to develop fluorescent sensors for various signaling molecules. First, I developed a green fluorescent sensor for oxytocin, a neuropeptide that play critical roles in social behaviors, food intake, and stress responses. Through the screening of hundreds of mutants, I finally obtained a sensitive fluorescent sensor that show a large fluorescence change (up to 700% $dF/F_0$). I applied this sensor to in vivo fiber photometry measurement, and found the active oxytocin dynamics in the brain upon a various physiological stimuli. Furthermore, I tried to extend the repertoire of the target signaling molecule of fluorescent sensors by using a similar way. Through the screening, I succeeded in obtaining sensitive green fluorescent sensor for ~20 signaling molecules. These fluorescent sensors will further extend our knowledge on the intercellular signaling dynamics in living systems. (COI:No)

### PP-55

### Characterization of Lrrn4-expressing cells in the hippocampus of adult mice

Tomoko Hisaoka[1], Tadasuke Komori[1], Atsushi Miyajima[2], Yoshihiro Morikawa[1] (*[1]Dept. Anatomy and Neurobiology, Wakayama Med. Univ., [2]Lab. of Cell Growth and Differentiation, Inst. for Quantitative Biosciences, The Univ. of Tokyo*)

Leucine-rich repeat neuronal protein 4 (Lrrn4), a member of leucine-rich repeat superfamily, is expressed in the nervous system. Previously, we have shown that Lrrn4 is highly expressed in the hippocampus and involved in formation of hippocampus-dependent long-term memory. However, little is known about the detailed expression pattern of Lrrn4 in the brain, including the hippocampus. To get insights into the role of Lrrn4 in the hippocampus, we characterized Lrrn4-expressing cells using heterozygous Lrrn4-knockout/lacZ-knockin mice. In both the dorsal and ventral hippocampus, Lrrn4 was expressed in the pyramidal cell layers of CA1-CA3 as well as in the granular and polymorphic layers of dentate gyrus. LacZ staining combined with immunohistochemistry for neuronal or glial markers revealed that Lrrn4 was exclusively expressed in NeuN-positive neurons. In addition, the expression of Lrrn4 was detected in ionotropic glutamate receptor 2/3-positive neurons, but not in $\gamma$-aminobutyric acid-positive neurons. These results suggest that Lrrn4 is expressed in glutamatergic neurons and may be related to their connections within the hippocampus and into other target regions. (COI:No)

### PP-56

### Optimization of SDS-FRL method for high sensitivity detection of the epitope tags on NAK$\alpha$3-containing NAK pumps

Kazuki Kuroda[1,2], Tatty Ishikawa[4], Kochi Murata[1,2], Yugo Fukazawa[1,2,3] (*[1]Div. Brain Structure and Function, Fac. Med. Sci., Univ. Fukui, [2]Life Sci. Inno. Cent,, Fac. Med. Sci., Univ. Fukui, [3]Res. Cent Child Mental Dev., Univ. Fukui, [4]Dept. Functional Anatomy, Grad. Sch. Med. Sci., Kanazawa Univ*)

$Na^+/K^+$-ATPases (NAK pumps), which is responsible for the asymmetrical distribution of $Na^+$ and $K^+$ across the plasma membrane, consists of three subunits, and multiple isoforms for each subunit have been identified. Although the neuronal expression of NAK$\alpha$1 and $\alpha$3 subunits is reported, there are still many unanswered questions, such as their expression ratio, differences among neuronal cell types, the relationship between NAK$\alpha$ and other subunit isoforms, and the relationship between subunit composition and neuronal function. When FLAG-tagged NAK$\alpha$3 mice were subjected to SDS-digested freeze-fracture replica labeling (SDS-FRL), the labeling intensity of FLAG-NAK$\alpha$3 by the M2 FLAG antibody was lower than those of anti-NAK$\alpha$3 polyclonal antibodies. However, by changing some of the experimental conditions; fixation of the brain and washing of the replica membranes, we were able to improve the labeling efficiency. We will discuss further options for the optimization of SDS-FRL toward the high sensitivity detection of membrane molecules on neurons. (COI:No)

### PP-57

### Energizing epilepsy

Kota Furukawa[1], Ko Matsui[1] (*[1]Super-network Brain Physiology Graduate School of Life Sciences Tohoku University*)

Epileptiform activity consumes cellular energy and supply of energy is required just to keep the neuronal firing to continue. Repeated epileptiform activity results in exacerbation of epilepsy through a process called kindling. It can therefore be assumed that energy production and consumption process change during the kindling process. To monitor the change in the energy source, ATP, Thy1-ATeam transgenic mice was used. A train of hippocampal electrical stimulation produced an after-discharge (AD) and the neuronal ATP concentration changes were detected with the fiber photometry system. AD duration increased with repeated hippocampal stimulation over days, but contrary to our expectations, we found that neuronal ATP decrease associated with the stimulation did not change or, in some cases, rather decreased as the AD progressed. The recovery time course of the ATP was also examined but no apparent change occurred. Therefore, it is possible that, upon kindling, the energy usage, for example, for the recovery of cellular ionic balance by Na-K ATPase became extremely efficient upon kindling. A new therapeutic strategy could aim to control the brain metabolism. (COI:No)

### PP-58

### A novel regulatory mechanism of AMPA receptor internalization via interaction between BRAG2 and endophilin 3 at hippocampal excitatory postsynapses

Masahiro Fukaya[1], Hiroyuki Sakagami[1] (*[1]Dept. Anatomy, Kitasato Univ. Sch. Med*)

BRAG2/Iqsec1 is a guanine nucleotide exchange factor for ADP ribosylation factor 6 (Arf6), a small GTPase implicated in the membrane trafficking. BRAG2 regulates Arf6-dependent endocytosis of AMPARs during LTD. However, the mechanism by which the BRAG2-Arf6 pathway links AMPARs to the endocytic machinery remains elusive. Using yeast two-hybrid screening, we identified endophilin 1/3 as novel BRAG2a-binding partners in the brain. In cultured hippocampal neurons, stimulation with DHPG, a group I mGluR agonist, increased the interaction of BRAG2a with endophilin 3 and concomitant Arf6 activation. Knockdown of BRAG2 in cultured hippocampal neurons suppressed the mGluR-dependent decrease in surface AMPAR levels, which was rescued by introducing wild-type BRAG2a, but not wild-type BRAG2b or BRAG2a mutants lacking the ability to activate Arf6 or to interact with endophilin 3. Further immunoelectron microscopy revealed the lateral distribution of BRAG2a, Arf6 and endophilin 3 for efficient endocytosis at the postsynapse. Taken together, the present findings unveiled a novel molecular mechanism by which BRAG2a links AMPARs to the endocytic pathway through its interaction with endophilin 3. (COI:No)

### PP-59

### Body temperature increases TRPM1 channel-mediated glutamate release frequency from retinal rod bipolar cells

Fuminobu Tamalu[1], Koji Shibasaki[2], Michio Shiibashi[1], Shuichi Watanabe[1], Naofumi Miwa[1] (*[1]Dep Physiol, Fac Med, Saitama Med Univ, Saitama, Japan, [2]Grad Sch Human Health Sci, Univ Nagasaki, Nagasaki, Japan*)

Retinal rod bipolar cells (RBCs) express the TRPM1 channel, which is a non-selective cation channel that opens in response to light. We show that RBCs depolarized significantly as the temperature was raised from 22 °C to 34 °C. When using ruthenium red (RR), a TRP channel inhibitor, there was no significant difference in the membrane potentials of RBCs between 22 °C and 34 °C. Similarly, insignificant depolarization by heat was observed in TRPM1 KO mice. Both the frequency and amplitude of EPSCs observed in AII amacrine cells, that are post-synaptic to RBCs, dramatically increased by heat. The electric charge of the EPSCs at 34 °C was 4.3-times greater than that at 22 °C and 3.1-times greater than that at 34 °C with RR, respectively. The EPSC frequency increased significantly as the temperature increased, but decreased by RR. The EPSC amplitude was increased by heat, but partially inhibited by RR. In TRPM1 KO mice, there was no significant difference between 22 °C and 34 °C observed in terms of frequency, but the amplitude increased significantly. Our findings indicate that the TRPM1 plays a role in the glutamate release frequency from RBCs at physiological temperature. (COI:No)

### PP-60

### Prostaglandin $E_2$ induces long-lasting calcium decrease of noradrenergic neurons in the locus coeruleus via $EP_3$ receptor

Yasutaka Mukai[1,2,3,4], Michael Lazarus[5], Takeharu Nagai[6], Kenji Tanaka[7], Akihiro Yamanaka[1,2,4] (*[1]Dept. of Neuroscience II, Research Inst. of Environmental Medicine, Nagoya Univ., Nagoya, Japan, [2]Dept. of Neural Regulation, Nagoya Univ. Grad. Sch. of Medicine, Nagoya, Japan, [3]JSPS Research Fellow DC, Tokyo, Japan, [4]CREST, JST, Saitama, Japan, [5]WPI-IIIS, Tsukuba Univ, Tsukuba, Japan, [6]Dept Biomol Sci and Eng, ISIR, Osaka Univ, Osaka, Japan, [7]Dept of Neuropsychiatry, Sch of Med, Keio Univ, Tokyo, Japan*)

Noradrenergic (NA) neurons in the locus coeruleus (LC-NA neurons) regulates various physiological functions, such as wakefulness. Time duration of the functions ranges from minutes to hours. However, bioactive substances affect the activity of LC-NA neurons in minutes- to hours-time scale are still elusive.

Therefore, we used a method for calcium imaging in acute brain slice to monitor the activity of LC-NA neurons in minutes- to hours-time scale. We generated transgenic mice expressing calcium indicator (YC) in NA neurons. We made acute brain slices and monitored YC signal after candidate substance bath application. Among 57 substances, prostaglandin $E_2$ ($PGE_2$; 1 μM) decreased calcium concentration ($[Ca^{2+}]_i$) for more than an hour.

Among $PGE_2$ receptors, only $EP_3$ receptor ($EP_3R$) is the inhibitory Gi-coupled receptor. Hence, we generated mice in which $EP_3R$ in NA neurons is conditionally knocked out (cKO mice), and monitored $[Ca^{2+}]_i$ after $PGE_2$ application. As a result, $PGE_2$-induced $[Ca^{2+}]_i$ decrease was abolished in cKO mice. This result suggests that $PGE_2$ induces long-lasting $[Ca^{2+}]_i$ decrease via $EP_3R$. We will study the physiological role of the $PGE_2$-$EP_3R$ signal in the LC-NA neurons. (COI:No)

### PP-61

### Calcium mobilization through metabotropic glutamate receptor 1 in the neonatal hippocampus

Megumi Taketo[1] (*[1]Dept Cellular and Functional Biol, Inst Biomed Sci, Facult Med, Kansai Medical Univ*)

Metabotropic glutamate receptors (mGluRs) are G-protein coupled receptors which play important roles in synaptic plasticity and memory formation in central nervous system. Group I mGluRs consisting of mGluR1 and mGluR5, couple to $G_q$ protein and increase intracellular $Ca^{2+}$ concentration ($[Ca^{2+}]_i$). Group I receptors also regulate several channels on cell membranes. Hippocampal marginal zone contains early-developed neurons including Cajal-Retzius (CR) cells, which regulate neuronal migration through production and release of reelin. In addition to instruction of migration, CR cells regulate network activity via excitatory input to other neurons. In the present experiments, fluorescence imaging revealed that group 1 mGluR-specific agonist induced $[Ca^{2+}]_i$ mobilization in CR cells. The $[Ca^{2+}]_i$ elevation was prevented by mGluR1-specific antagonist, and was observed in the absence of extracellular $Ca^{2+}$. The effects of other excitatory neurotransmitters on $[Ca^{2+}]_i$ were also measured and compared with the mGluR1-mediated $Ca^{2+}$ elevation. Possible interaction between mGluR1 and other receptors and channels expressed in CR cells was also investigated. (COI:No)

### PP-62

### Voltage-sensitive $Na^+$ and $K^+$ current components in interneuron precursors in the medial ganglionic eminence of the embryonic cerebrum

Tenpei Akita[1], Atsuo Fukuda[1] (*[1]Dept Neurophysiol, Hamamatsu Univ Sch Med, Japan*)

The expression and roles of voltage-sensitive $Na^+$ and $K^+$ channels in the embryonic cerebrum remain unknown. We explored voltage-sensitive membrane current components in interneuron precursors in the mouse medial ganglionic eminence at embryonic day 14, using the whole-cell patch-clamp technique. The membrane current showed outward rectification with a reversal potential of -30 mV. The tail current generated after the end of a depolarizing voltage step also reversed around -30 mV. The currents were abolished by removal of $Na^+$ and $K^+$ from extra- and intracellular solutions. Removal of $Na^+$ alone selectively abolished inward currents, but it also significantly reduced outward currents. The remaining outward currents showed a clear voltage-gated increase in conductance at around -20 mV, and were suppressed strongly by 4-AP (2 mM) and partially by TEA (1 mM) or Dendrotoxin-I (100 nM). $Gd^{3+}$ (10 μM) application had a similar effect as the $Na^+$ removal, and the currents in the presence of $Gd^{3+}$ were suppressed by 4-AP. Thus, at least 2 different voltage-sensitive current components, presumably mediated by Kv1 channels and non-selective cation channels, are present in the precursors. (COI:No)

### PP-63

### Correlative two-photon and high-resolution fluorescence microscopy of dendritic spines in cortical pyramidal neurons

Yutaro Kashiwagi[1], Hiroshi Terashima[1], Masaaki Endo[1], Shigeo Okabe[1] ([1]*Dept. of Cell. Neurobiol., Med., The Univ. of Tokyo*)

Most of the excitatory neuronal inputs are formed onto spines extending from neuronal dendrites of pyramidal neurons in the neocortex and hippocampus. Spines are transient structures with continual formation and elimination. Spine turnover is thought to underlie as a potential mechanism of functional modulation in the neuronal circuits.
Here, we developed an efficient correlative method of high-resolution fluorescence imaging combined with in vivo two-photon imaging. After the acquisition of spine images in vivo by two-photon imaging, the nano-scale morphological features of the same spines were subsequently analyzed with high-resolution fluorescence microscopy in fixed slices. This technique can obtain images of hundreds of spines in a wide range of dendritic trees extending from a single neuron. Correlative two-photon and high-resolution fluorescence microscopic analysis of spines will be applicable to analyze many spines in a single neuron with the information of the location of individual spines along dendritic arborization. This technique may be useful to understand synaptic weight redistribution in the learning-related neural circuits after memory acquisition. (COI:No)

### PP-64

### Morphological analysis of cortical pyramidal neurons in an autism mouse model

Hiroshi Terashima[1], Yutaro Kashiwagi[1], Takanobu Nakazawa[2], Shigeo Okabe[1] ([1]*Department of Cellular Neurobiology, Graduate School of Medicine and Faculty of Medicine, the University of Tokyo,* [2]*Department of Bioscience, Tokyo University of Agriculture*)

Autism spectrum disorder (ASD) is characterized by deficits in social communication and restricted patterns of behavior and interests. In several different ASD mouse models, dendritic spine turnover in the cortical pyramidal neurons has been shown to be upregulated. This property may indicate the common synaptic deficits in ASD. New tissue-preparation and imaging protocols to analyze the fine structures of spines in a large tissue volume and with high efficiency will help perform a comprehensive analysis of the synaptic pathology in ASD.
Here, we established a method for morphological analysis of spines in tissue slices. We analyzed a newly developed ASD mouse model with *POGZ* mutation. With conventional immunohistochemical techniques, we analyzed the distribution of cortical layer markers and inhibitory interneuron markers. By the exogenous expression of fluorescent proteins, the dendritic arborization of layer 2/3 pyramidal neurons was also compared. We further examined the fine structures of spines using the new imaging technique. Integration of in vivo imaging with this new technique will allow us further to understand the circuit-level abnormalities in ASD mouse models. (COI:No)

### PP-65

### A comparison of dendritic spine turnover in mouse lines with genetic manipulations of lateral olfactory tract usher substance (LOTUS) expression.

Masaaki Endo[1], Shinji Tanaka[1], Kohtaro Takei[2], Shigeo Okabe[1] ([1]*Department of Cellular Neurobiology, Graduate School of Medicine and Faculty of Medicine, the University of Tokyo.,* [2]*Molecular Medical Bioscience Laboratory, Yokohama City University Graduate School of Medical Life Science*)

Lateral olfactory tract usher substance (LOTUS) was identified as a key molecule for lateral olfactory tract formation by antagonizing Nogo receptor-1 (NgR1) signaling. Previous reports show that NgR1 plays essential roles in modulating synaptic function and dynamics. This suggests that LOTUS could regulate synapses by regulating NgR1. However, the function of LOTUS at synapses is not well understood.
Here, we examined this possibility using in vivo spine imaging in the developing postnatal cortex of mouse lines with genetic manipulations of LOTUS expression. We performed in vivo two-photon time-lapse imaging in layer 2/3 pyramidal neurons expressing PSD-95 tagged with green fluorescent protein (GFP) as a synaptic marker, together with red fluorescent protein DsRed2 as a volume marker. We compared the population of newly formed and eliminated spines of wild-type, lotus-deficient (LOTUS-KO), and lotus-overexpressing transgenic (LOTUS-Tg) mice and classified spines into PSD-95-GFP positive or PSD-95-GFP negative spines. This study will give new insights into the function of LOTUS at synapses and elucidate the signaling mechanisms underlying synaptic dynamics. (COI:No)

### PP-66

### Actin-dependent rapid tethering of synaptic vesicles accompanying exocytosis at a fast central synapse

Takafumi Miki[1], Mitsuharu Midorikawa[2], Takeshi Sakaba[1] ([1]*Grad Sch Brain Science, Doshisha Univ,* [2]*Dept Physiol, Sch Med, Tokyo Women's Med Univ* )

A high rate of synaptic vesicle (SV) release is required at cerebellar mossy fiber terminals for rapid information processing. As the number of release sites is limited, fast SV reloading is necessary for achieving sustained release. However, rapid reloading has not been observed directly. Here, we visualize SV movements near presynaptic membrane using total internal reflection fluorescence (TIRF) microscopy. Upon stimulation, SVs appeared in the TIRF-field and became tethered to the presynaptic membrane with unexpectedly rapid time-course, almost as fast as SVs disappearing due to release. However, such stimulus-induced tethering was abolished by inhibiting exocytosis or by actin disruption, suggesting that actin-dependent tethering is tightly coupled to preceding exocytosis. The newly-tethered vesicles became fusion-competent not immediately but only 300-400 ms after tethering. Together with model simulations, we propose that rapid tethering leads to an immediate filling of vacated spaces and release sites within <100 nm of the active zone by SVs, which serve as precursors of readily-releasable vesicles, thereby shortening delays during sustained activity. (COI:No)

### PP-67

### Three modes of spontaneous synaptic vesicle fusion revealed in larval zebrafish neuromuscular junctions

Yoshihiro Egashira[1], Akio Oujida[2], Fumihito Ono[1] ([1]*Dept Physiol, Grad Sch Med, Osaka Medical College, Japan,* [2]*Grad Sch Pharm Sci, Kyushu Univ, Japan*)

The information transfer at synapse is mediated by the release of neurotransmitters stored in the synaptic vesicles (SVs). All synapses show both evoked and spontaneous forms of SV fusion. Although it is now accepted that molecular mechanism underlying spontaneous release is partly different from that of evoked release, it remains controversial whether the SV population giving rise to the two types of release are segregated. To address this issue using the zebrafish neuromuscular junction, we generated a novel transgenic (Tg) fish in which two independent indicators of synapse dynamics, i.e. a pH sensitive fluorescent protein and Halotag, were fused in tandem to the luminal side of a SV protein expressed specifically in the motor neurons. This Tg fish allowed us not only to monitor exo / endocytosis of SVs but also to separately tag SVs mobilized by evoked and spontaneous activities. Using this technique and electrophysiology, we found that there are three distinct modes of spontaneous SV fusion, identified by their difference in sensitivity to temperature, $[Ca^{2+}]_{out}$ and tetanus-toxin. We will discuss the significance and the release mechanism of these distinct SV populations. (COI:No)

### PP-68

### Sensory input dependent and independent development of presynaptic transmitter release mechanisms at lemniscal fiber terminals in the somatosensory thalamus

Mitsuharu Midorikawa[1], Mariko Miyata[1] ([1]*Dept Physiol. Sch Med. TWMU*)

Fast synaptic transmission is necessary for precise information processing at mature central nervous systems. However, how it is acquired through the development is largely unknown, especially on the presynaptic side at most CNS synapses. It also remains unclear how sensory input affect the presynaptic functional maturations.
To address these issues, we focused onto lemniscal fiber terminals, which mediates whisker sensory inputs, at rodent somatosensory thalamus. We have previously shown that the sensory input is crucial for the establishment of the mature neuronal wiring (Takeuchi et al., 2014), but it remains unknow how presynaptic functions such as transmitter release mechanism mature with development, and how sensory input affects the presynaptic functional maturations.
We examined the kinetics of transmitter release from the whisker-mediated lemniscal fiber terminals by membrane capacitance measurements, as well as by preterminal-postsynaptic neuron paired recordings. Our results revealed experience dependent and independent maturation process at the lemniscal fiber terminals. (COI:No)

### PP-69

### Androgen affects the dynamics of intrinsic plasticity of pyramidal neurons in the CA1 hippocampal subfield and inhibitory avoidance memory in adolescent male rats

Mdnabiul Islam[1], Yuya Sakimoto[2], Mir Rubayet Jahan[1], Abu Md Mamun Tarif[1], Kanako Nozaki[1], Koh-hei Masumoto[1], Akie Yanai[1], Dai Mitsushima[2], Koh Shinoda[1] (*[1]Div. Neuroanat., Grad. Sch. Med., Yamaguchi Univ., Japan, [2]Dept. Physiol., Grad. Sch. Med., Yamaguchi Univ., Japan*)

Androgen receptor (AR) is abundantly expressed in the amygdala and hippocampus where androgen plays an important role in emotional behaviors and memory. The effects of androgen on the intrinsic plasticity of hippocampal neurons and inhibitory avoidance (IH) memory have not been elucidated. In this study, the effects of androgen on the expression of AR, dynamics of intrinsic plasticity of CA1 pyramidal neurons and IH memory were examined using in sham-operated, orchiectomized (OCX), OCX + testosterone (T) or OCX + dihydrotestosterone (DHT)-primed adolescent male rats. Orchiectomy significantly decreased AR-immunoreactivity, resting membrane potential, action potential numbers, afterhyperpolarization amplitude, membrane resistance, and latency in IH test whereas it significantly increased action potential threshold and membrane capacitance. These effects were successfully reversed by treatment with either aromatizable androgen T or non-aromatizable androgen DHT. Furthermore, administration of the AR-antagonist flutamide in intact rats showed similar changes to those in OCX rats, suggesting that androgens affect the excitability of CA1 pyramidal neurons possibly by acting on the AR. (COI:No)

### PP-70

### Astroglial CD38 regulates synaptic formation in the cortex and social memory

Tsuyoshi Hattori[1], Jureepon Roboon[1], Hiroshi Ishii[1], Mika Takarada[1], Stanislav Cherepanov[2], Haruhiro Higashida[2], Osamu Hori[1] (*[1]Neuroanat, Grad Sch Med, Kanazawa Univ, [2]Child Ment Dev, Kanazawa Univ*)

Astrocytes have emerged as important players in brain function under both physiological and pathological conditions. CD38 is a multifunctional molecule with ADP-ribosyl cyclase activity and dominantly expressed in astrocytes in developing brain. While critical roles of CD38 in oxytocin neurons for oxytocin release and social behavior have been reported, those of astrocytic CD38 in the developing brain remain largely unknown. By selectively deleting CD38 in postnatal astrocytes, CD38 conditional KO mice exhibited impaired social memory, but did not show other abnormal behavior phenotypes such as sociability, social preference, activity and anxiety. Furthermore, morphological analysis using Golgi staining and immunohistochemistry revealed reduced spine number, mature spines and synaptic numbers in the mPFC of CD38 conditional KO mice. Astrocyte conditioned medium (ACM) collected from CD38 KO astrocytes caused reduced synaptic number in cultured primary neurons. Our data indicate that astroglial CD38 is a positive regulator of synaptic formation and social memory formation. (COI:No)

### PP-71

### Olig2-astrocyte preferentially express astrocytic transporter SLC7A10 (Asc-1) in the central nervous system.

Kouko Tatsumi[1], Kaoru Kinugawa [2,4], Ayami Isonishi[1], Hiroaki Okuda[3], Shoko Takemura[1], Tatsuhide Tanaka[1], Eiichiro Mori[4], Akio Wanaka[1] (*[1]Dept Anat Neurosci, Med, Nara Med Univ, [2]Dept Neurol, Med, Nara Med Univ, [3]Dept Funct Anat, Med, Kanazawa Univ, [4]Dept Future Basic Med, Med, Nara Med Univ*)

We have previously reported that the transcription factor Olig2 labels a subpopulation of astrocytes (Olig2-AS). Olig2-AS were distinct from GFAP-expressing astrocytes (GFAP-AS); the two types occupied mutually exclusive territories in the adult brain regions, even within a single brain nucleus such as the external globus pallidus (LGP), and this unique territory makes us expected that olig2-AS have specific functions. In this study, we compared the gene expression profiles by two methods; one is the data analysis using single-cell RNA-sequence database *in silico*, and another is the mRNA expression analysis using differentially isolated Olig2- and GFAP-AS from the LGP using laser-microdissection. Both analyses showed that Olig2-AS expressed higher neutral amino acid transporter-1, SLC7A10 mRNA than the GFAP-AS. Subsequent immunohistochemical study also showed that Olig2-AS preferentially expressed SLC7A10. Taken together, these results supported the recent study reporting restricted expression of SLC7A10 to a subset of astrocyte and strongly suggested that SLC7A10 was one of the candidate genes characterizing the specific functions of Olig2-AS in the central nervous system. (COI:No)

### PP-72

### Effects of RORγt overexpression on the central nervous system and behaviors

Yosuke Takei[1,2], Satoru Takahashi[3], Tetsuya Sasaki[1,2] (*[1]Dept Anat & Neurosci, Fac Med, Univ of Tsukuba, Ibaraki, Japan, [2]Doctorate Program Neurosci, Grad Sch Comp Human Sci, Univ of Tsukuba, Ibaraki, Japan, [3]Dept Anat & Embryol, Fac Med, Univ of Tsukuba, Ibaraki, Japan*)

Th17 cells are a subset of $CD4^+$ T cells that produce IL-17A. It has been suggested that IL-17A affects brain function. Using transgenic mice overexpressing RORγt, a transcription factor essential for differentiation of Th17 cells (RORγt Tg mice), we examined changes in the brain caused by chronically increased IL-17A resulting from excessive activation of Th17 cells. RORγt Tg mice exhibited elevated *Rorc* and *IL-17A* mRNA expression in the colon, as well as a chronic increase in circulating IL-17A. We found that the immunoreactivity of Iba1 and density of microglia were lower in the dentate gyrus of RORγt Tg mice compared with wild-type mice. However, $GFAP^+$ astrocytes were unchanged in the hippocampi of RORγt Tg mice. Levels of synaptic proteins were not significantly different between RORγt Tg and wild-type mouse brains. In addition, novel object location test results indicated no difference in preference between these mice. Our findings indicate that a continuous increase of IL-17A in response to RORγt overexpression resulted in decreased microglia activity in the dentate gyrus. (COI:No)

### PP-73

### Involvement of hippocampal microglia in vulnerability and resistance to social defeat stress

Risako Fujikawa[1], Shozo Jinno[1] (*[1]Anatomy and Neuroscience, Grad. Med., Kyushu Univ*)

Social stress precipitates psychopathological disorders in susceptible individuals. However, the mechanisms underlying the stress vulnerability are poorly understood. Interestingly, several studies have reported that some but not all mice exposed to social defeat stress (SDS) exhibit stress resistance. In this study, we aim to elucidate the potential role of microglia, resident immune cells of the brain, in vulnerability and resistance to SDS. Male C57BL/6J mice were repeatedly subjected to SDS (1 min/day) by an aggressive male ICR mouse for 5 consecutive days. After that, about half of subject mice showed depression-like behaviors (vulnerable group), while remaining mice showed no such behaviors (resistant group). The spatial densities of microglia in the hippocampus were higher in the vulnerable group than in the resistant group. In addition, the microglial densities showed weak correlations with depression-like behaviors. The three-dimensional reconstruction analysis showed that the complexity of microglia were reduced in the vulnerable group, but not in the resistant group. Distinct activation of microglia may underlie the difference in vulnerability and resistance to SDS. (COI:No)

### PP-74

### Investigation of satellite glial cells around neurite of the dorsal root ganglion neuron with array tomography and CLEM

Taro Koike[1], Susumu Tanaka[1], Masahiko Kase[1], Yukie Hirahara[1], Shinichi Hayashi[1], Souichi Oe[1], Yousuke Nakano[1], Masaaki Kitada[1] (*[1]Dept. Anatomy, Kansai Med. Univ*)

Satellite glial cells (SGCs) surrounding a large dorsal root ganglion neuron are reported to be morphologically classified into two groups: one covers a neuronal soma (regular SGC), and the other covers the initial region of the neurite (specialized SGC). However, there are few papers to describe specialized SGC. In the present study, we investigated morphological features of specialized SGCs in the adult rat. First, specialized SGCs were observed along the neurite by using array tomography. Specialized SGCs in the proximal and middle region of the neurite showed a thick cytoplasm and had filopodia extending toward the neurite, however, the cells distributed in the distal region had thin cytoplasm that surrounded the neurite like a swiss-role. Next, cell markers in these cells were examined by using immunohistochemistry and correlative light and electron microscopy (CLEM). Most specialized SGCs were positive for cell markers for regular SGCs. Interestingly, specialized SGCs in the distal region were positive for a promyelinating Schwann cell marker. These results suggest that most specialized SGCs are regular SGCs but the distal ones are promyelinating Schwann cells. (COI:No)

### PP-75

### Analysis of long chain fatty acid treatment on MG6 microglial cells

Shuhan Yang[1], Hirofumi Miyazaki[1], Yuji Owada[1] ([1]*Dept. of Organ Anatomy,. Grad. Sch. Med., Tohoku Univ*)

Microglia are immune-related cells of the central nervous system and play an important role in brain pathology as well as its normal homeostasis. Recent studies show that the number and morphology of microglia in the brains of obese individuals are altered and that a high-fat diet triggers activation of hypothalamic microglia, suggesting the relationship between systemic metabolism and microglial function. In the present study, we examined the effects of various long-chain fatty acids (LCFAs) on microglial growth and phagocytosis. In microglia cell line MG6, treatment with different LCFAs including palmitic acid, α-linolenic acid and oleic acid increased the number of intracellular lipid droplets. Treatment of MG6 cells with palmitic acid significantly increased latex bead uptake compared to other LCFAs.
The type of LCFAs affects the regulation of microglial function. We will further analyze the responsiveness of brain microglia to a high-fat diet, along with changes in proliferation and phagocytosis-related transcripts after treatment with LCFAs. (COI:No)

### PP-76

### Analysis of pain mechanism in an animal model for the fibromyalgia induced by repeated cold stress.

Koji Wakatsuki[1,3], Masaya Yasui[2], Sumiko Kiryu-Seo[1], Hiroshi Kiyama[1] ([1]*Department of Functional Anatomy and Neuroscience, Graduate School of Medicine, Nagoya University,* [2]*Department of Judo Seifuku and Health Sciences Faculty of Health Promotional Sciences, Tokoha University,* [3]*Tokai college of medical science Judo therapy course*)

Fibromyalgia (FM) is a chronic and incurable disease that causes severe pain and fatigue. The repeated cold stress (RCS) model of rodent is used as an animal model for FM, although detailed mechanisms underlying the pain in FM is unknown. In this model, an accumulation of activated microglia was seen in the restricted region of the dorsal horn; however, the reason why the microglia were activated and localized was not clear. To address this issue from the aspect of the neural circuit, we used hyperactivation-responsive GFP mice (*Atf3*:BAC Tg mice) in which hyperactivated neurons were labeled by GFP. When *Atf3*:BAC Tg mice were exposed to the RCS, the pain threshold of the mice decreased. Immunohistochemical staining demonstrated that both the proprioceptive DRG neurons, which innervated muscle spindle of soleus, and a subset of lumbar spinal motor neurons, which projected to soleus were labeled by GFP, suggesting the reflex arc activation. The microglial accumulation appeared to occur along the arc. These results suggest that chronic hyperactivation of proprioceptor elicits microglial activation along the arc, and these microglia are supposed to lead to the abnormal pain sensation. (COI:No)

### PP-77

### Purinoceptor P2Y$_2$ mediates activation of extracellular signal-regulated kinase 1/2 (ERK) in stellate Schwann-like cells associated with lanceolate sensory endings innervating rat vibrissae.

Hiromi Takahashi-Iwanaga[1] ([1]*Hokkaido Univ. Grad. Sch. Med., Dept. Anat*)

The intracellular ERK signaling reportedly promotes dedifferentiation of Schwann cells to a progenitor-like state in regenerating peripheral nerves. Lanceolate endings — motion-detectors of the rat vibrissae — accompany specific glial elements that retain immature features: terminal Schwann cells with a cytoplasmic process branching into lamellar coverings on receptor axons (1), and stellate Schwann-like cells radiating free processes (2). These two glial cell types were seen to express the metabotropic ATP receptor P2Y$_2$ in our previous experiments with acute tissue preparations isolated from transgenic rats Tg[S100β-EGFP]. To test an involvement of the purinergic signals in ERK activation, the isolated lanceolate specimens were incubated at 37 °C for 60 min in the presence or absence of the P2Y$_2$ agonist UTP at 50 μM, and subsequently immunostained for phosphorylated ERK (pERK). The % cell positivity for pERK immunoreaction was significantly augmented in stellate Schwann-like cells stimulated with UTP as compared with the normal control. The cell reaction was blocked by the P2Y$_2$ antagonist AR-C118925XX, as well as by the phospholipase C inhibitor U73122. (COI:No)

### PP-78

### Phosphatidylserine recognition is required for the cell cycle reentry of Müller glia

Kaori Komoike[1], Hiroki Fujieda[1] ([1]*Tokyo Women's Medical University Anatomy*)

Retinal Müller glia (MG) have a potential to proliferate and regenerate retinal neurons in response to retinal injury. We previously reported that the injury response of MG including proliferation and phagocytosis of dead cells, is mediated by phosphatidylserine (PS), an "eat-me" signal presented by apoptotic cells. However, the precise mechanism by which PS regulates the MG response remains unclear. Here we investigated the temporal window of PS-mediated regulation of MG proliferation. Rat retinas were explant-cultured at two time points (one or two days) after photoreceptor injury by N-methyl-nitrosourea, and the effects of L-SOP, a PS inhibitor, on the different phases of the cell cycle of MG were examined by EdU (S-phase) and phosphorylated pRB immunofluorescence (all phases). When the retina was treated with L-SOP before MG reentered the cell cycle (one day after injury), the G1 phase entry of MG was blocked. When MG in G1 phase were treated with L-SOP (two days after injury), the S phase entry was inhibited. These data indicate that PS-mediated signaling is required both for the G1 phase entry and G1-S phase progression of the injury-induced proliferation of MG. (COI:No)

### PP-79

### Development of the glycine-removal system in astrocytes of the spinal cord

Chigusa Shimizu-Okabe[1], Akihiro Kobashikawa[1], Daisuke Omata[1], Ryuji Tomoyose[1], Kie Okano[1], Chitoshi Takayama[1] ([1]*Dept.of Molecular Anatomy, School of Medicine,University of the Ryukyus*)

Glycine and GABA are inhibitory neurotransmitters in the adult spinal cord. Both are removed from the synaptic cleft into astrocytes by glycine transporter1 (GlyT1) and GABA transporter 3 (GAT-3). To reveal the ontogeny of the glycine-removal system in the astrocytes, we examined immunohistochemistory of GlyT1during development of spinal cord.On E13, GlyT1 was localized in the process of radial glia, whereas GAT-3 was expressed in the radial fibers.These results suggested that glycine and GABA were uptaken in different region of the radial glia in E12-14, but after that, both neurotransmitters were removed by the same astrocytes. After E16, GlyT2 which uptakes glycine in presynaptic terminal was expressed. This results indicated that Before the formation of glycinergic terminal, glycine-removal system in astrocytes might be ready. (COI:No)

### PP-80

### Glial switch of fear memory

Hiroki Yamao[1], Ko Matsui[1] ([1]*Super-network Brain Physiology, Graduate School of Life Sciences, Tohoku University*)

An experience has the potential to become either a long lasting memory, or a faded matter from the past. Glial cells play a significant role in regulating the brain`s environment. We suggest that glial activity modulates memory formation, acting as a switch between different circumstances. To assess the glial contribution to memory, a fear conditioning paradigm was conducted on transgenic mice. Glia specific optogenetic stimulation was applied during conditioning, and effects on fear memory formation was studied. Glia specific ChR2 activation in the amygdala resulted in increased freezing directly after the foot-shock, but contrary to expectations, fear memory formation was suppressed. This may be the result of disrupted neural encoding of the incident. In contrast, glia specific ArchT activation in the amygdala had no effect on freezing directly after the foot-shock, but fear memory formation was augmented. Enhanced memory consolidation was likely triggered by the glial ArchT activation. Activation of ChR2 induces proton influx and ArchT activation results in proton extrusion. Thus, changes in intra-glial pH could be the definitive factor switching the fate of a memorable event. (COI:No)

### PP-81

### Functional oligodendrocyte activity required for the activity dependent myelination

Kenji Yoshida[1], Shota Sugio[1], Daisuke Kato[1], Hiroki Uchida[1], Hiroaki Wake[1] (*[1]Department of Anatomy and Molecular Cell Biology, Nagoya University Graduate School of Medicine, Nagoya, Japan*)

Myelination play a pivotal role in neuronal plasticity by the regulation of conduction velocity and thus the firing timing of neurons. Although, previous study showed the neuronal activity dependent MBP translation *in vitro* (Wake et al., 2011), it is unclear how the activity dependent myelination is regulated *in vivo*. In this research, we visualized functional response of oligodendrocyte (OL) using *in vivo* two photon $Ca^{2+}$ imaging. We found that $Ca^{2+}$ activities of OL increased after the promotion of neural activity using Designer Receptors Exclusively Activated by Designer Drugs. This effect was reduced after application of P2X receptor antagonist (Suramin) on brain surface but not after AMPA receptor antagonist (CNQX), suggesting ATP signals via P2X could affect $Ca^{2+}$ activities of OL but not by the glutamate signals. We further recorded electrical responses from OL by patch clamp recording in acute brain slices. OL electrical response was decreased after Suramin and CNQX application. These results indicate that electrical and $Ca^{2+}$ activities of OL could be differently regulated. We are trying to examine neural activity after deletion of AMPA or P2 receptors from OL. (COI:No)

### PP-82

### Glial contribution to the early phase of the parallel memory formation process

Teppei Kanaya[1], Daichi Sasaki[2], Ryo Ito[3], Hiroki Yamao[2], Kaoru Beppu[4], Ko Matsui[1,2] (*[1]Super-network Brain Physiology, Graduate School of Medicine, Tohoku University, [2]Super-network Brain Physiology, Graduate School of Life Sciences, Tohoku University, [3]Department of Physics, Faculty of Science, Tohoku University, [4]Department of Physiology, Graduate School of Medicine, Tohoku University*)

By releasing gliotransmitter, glia can modulate synaptic transmission and plasticity. We have previously reported that channelrhodopsin-2 photoactivation in the glia can evoked glutamate release, which leads to the acceleration of cerebellar motor learning. However, whether glial contribution is both sufficient and required for the establishment of learning and memory remains to be resolved. As glial glutamate release is triggered by intracellular acidification, we show here, that intracellular alkalization by optogenetic activation of a proton pump, archaerhodopsin-T (ArchT) can suppress the glial glutamate release. Several hours of training can normally increase the amplitude of the eye movement in the horizontal optokinetic response (HOKR) learning paradigm, which is known to be dependent on the flocculus area of the cerebellum. Here, we show that ArchtT photoactivation of the cerebellar glia can impair the HOKR learning process. Surprisingly, however, the HOKR amplitude increased on the next day testing, which shows that long-term memory can be formed without the short-term memory. This result shows the presence of mutually independent parallel process of memory formation. (COI:No)

### PP-83

### Effect of astrocytic $\beta_2$ adrenaline signaling for motor learning

Yuki Aoyama[1,2], Daisuke Kato[1], Ikuko Takeda[1], Hiroaki Wake[1] (*[1]Department of Anatomy and Molecular Cell Biology, Nagoya University Graduate School of Medicine, [2]Division of System Neuroscience, Kobe University Graduate School of Medicine, Kobe, Japan*)

Astrocytes are nonelectrical cells but their $Ca^{2+}$ activities are essential for their physiological functions and induced by neural activity. The noradrenaline signaling (NA) from the locus coeruleus elicits $Ca^{2+}$ responses in astrocytes followed by the induction of synaptic plasticity in the cortex. However, the functional meaning and their change in NA induced astrocytes $Ca^{2+}$ activity during motor learning processes are still unclear. In this research, we characterized astrocytic activities in the secondary motor cortex of mice associated with motor learning with *in vivo* two photon $Ca^{2+}$ imaging of astrocytes. Mice were performed a combination of voluntary forelimb movement task for 14 days with $Ca^{2+}$ imaging of astrocytes. Increased astrocytic activities were observed in the late stage of motor learning. Inhibition of $\beta_2$ NA after the establishment of motor learning showed a change in $Ca^{2+}$activity. Unexpectedly, $Ca^{2+}$ activity increased after administration of $\beta_2$ NA inhibitors, and was higher than in the early, middle, and late stages of motor learning. We inhibiting astrocyte-specific $\beta_2$ NA to determine how astrocytic $\beta_2$ NA involve in motor learning. (COI:No)

### PP-84

### Structural and functional connectivity of precuneus and medial temporal lobe in the default mode network.

Tatsuya Jitsuishi[1], Takashi Hozumi[1,2], Hiroshi Kikuchi[1,5], Keiko Kitajo[1], Setsu Sawai[1,3], Masatoshi Komiyama[4], Atsushi Yamaguchi[1] (*[1]Dept Functional Anatomy, Univ of Chiba, Chiba, Japan, [2]Dept Orthopedic Surgery, Univ of Chiba, Chiba, Japan, [3]Dept Neurology, Univ of Chiba, Chiba, Japan, [4]Dept Nursing Physiology, Univ of Chiba, Chiba, Japan, [5]Dept Neurosurgery, Univ of Chiba, Chiba, Japan*)

The default mode network (DMN) is a representative functional network of the resting brain, which consists of a core and subsystems. The precuneus forms the core of the DMN, while the medial temporal lobe (MTL) is a subsystem of DMN. These two regions are situated close on the medial surface of hemisphere, both of which are involved in memory process and impaired at early stage of dementia. The aim of this study is to investigate the structural (SC) and functional connectivity (FC) of precuneus-MTL connection, using Human Connectome Project dataset. Firstly, we conducted the quantitative tractography analysis of precuneus-MTL connection. The major streamlines originated from the posterior precuneus and projected to the parahippocampal gyurs. Then we conducted the white matter dissection in the post-mortem brain and confirmed the major fiber bundles to connect the posterior precuneus and parahippocampal gyrus. Currently, we are analyzing the SC-FC relation by resting-state functional MRI. Collectively, our results indicate the posterior precuneus is highly connected to the MTL, possibly representing the critical functions in memory process as well as the vulnerability to dementia. (COI:No)

### PP-85

### Encoding of social interaction by neuronal ensembles in the insular cortex

Masaaki Sato[1,2], Isamu Miura[3], Eric Overton[2], Nobuo Kunori[4], Junichi Nakai[5], Takakazu Kawamata[3], Nobuhiro Nakia[2,6], Toru Takumi[2,6] (*[1]Dept Neuropharmacol, Grad Sch Med, Hokkaido Univ, [2]RIKEN CBS, [3]Dept Neurosurg, Tokyo Women's Med Univ, [4]AIST, [5]Div Oral Physiol, Tohoku Univ Grad Sch Dent, [6]Dept Physiol Cell Biol, Kobe Univ Sch Med*)

Recent advances in rodent social neuroscience have uncovered the function of critical centers for social behavioral control, such as hypothalamus, amygdala and medial prefrontal cortex. However, characterization of additional network nodes is necessary for the full elucidation of neural circuit mechanisms underlying complex social behavior. Using microendoscopic calcium imaging of the agranular insular cortex (AI) in mice freely interacting with social targets, we examined how neurons in AI encode information regarding social behavior. We identified two subsets of AI neurons—"Social-ON" cells and "Social-OFF" cells—that alter their activity in opposing directions during social interaction. Social-ON cells involved those that represented social investigation independent of spatial information and consisted of distinct functional subsets, each of which was activated preferentially during interaction with a particular target of physical contact or under a particular behavioral state. These findings reveal a novel role of AI neurons that may act to monitor the ongoing status of social investigation while an animal interacts with unfamiliar conspecifics. (COI:No)

### PP-86

### Salicylate-induced changes of the responses to the upward FM sounds in AI and DC field of guinea pigs observed by optical recording.

Yutaka Hosokawa[1], Shunji Sugimoto[2] (*[1]Dept. of Systems Physiol., Grad. Sch. Univ. of Ryukyus, [2]Dept. of Comp. Sci. and Eng., Grad. Sch. of Eng., Toyohashi Univ. of Technology*)

The influence of salicylate on the responses to the upward FM sounds in the primary auditory cortex (AI) and DC field of the guinea pig were investigated using optical imaging with a voltage-sensitive dye (RH795). Eight guinea pigs were anesthetized with ketamine (80 mg/kg) and xylazine (40 mg/kg). Activity patterns to the upward FM sounds (the linear sweep: the start and end frequency, 0.5 and 16 kHz in 16-64 ms duration) and tones (0.5-16 kHz, 200 ms duration) at 55-85 dB SPL were recorded from the AI and DC field on both sides before (control) and 0.5-2, and 6 hours after the intraperitoneal injection of 300 mg/kg salicylate. When the sound pressure level is high, the active-spots to the upward FM sounds were appeared at the 0.5kHz-frequency band (FB) in the AI and DC field with and without salicylate injection. On the lower sound pressure, the active-spots were narrower appeared in the higher FB of the each field at an hour after salicylate injection. The active-spots positions were separated narrower when the sound pressure was lower. These results show that the responses to the low and high frequency sound were suppressed by the salicylate injection. (COI:No)

### PP-87

### Effects of optimal polarity of transcranial direct current stimulation on motor imagery activity

Shigetoshi Iio[1], Tatsuya Mima[2], Yumie Ono[3] ([1]*Electrical Engineering Program, Graduate School of Science and Technology, Meiji University,* [2]*Graduate School of Core Ethics and Frontier Sciences, Ritsumeikan University,* [3]*Department of Electronics and Bioinformatics, School of Science and Technology, Meiji University*)

We investigated the effect of different polarity (anodal or cathodal) of transcranial direct current stimulation (tDCS) on the motor imagery (MI) responses. Nine young adult participants performed two sessions of hand MI task under EEG measurement. A continuous 1 mA anodal or cathodal tDCS was simultaneously applied to the motor area of the dominant hand in the second session. Changes in the event-related desynchronization (ERD) intensity between two sessions were determined to evaluate the effect of tDCS. Increase in ERD intensity was confirmed in 7 out of 9 participants, among whom 3 and 4 participants showed larger enhancement of ERD intensity with anodal and cathodal stimuli, respectively. When the optimal polarity was selected, tDCS significantly increased the ERD intensity and the number of trials showing positive ERD. There results suggest that the optimal polarity of tDCS should be determined individually and that the optimal stimulation might enhance motor cortical excitability. We will also present the results of neurofeedback training of MI-ERD under optimal tDCS on site. (COI:No)

### PP-88

### Snake and face images-selective neuronal responses in the monkey amygdala

Meng Yang[1], Ha Dinh[1], Hiroshi Nishimaru[1], Jumpei Matsumoto[1], Tsuyoshi Setogawa[1], Taketoshi Ono[1], Hisao Nishijo[1] ([1]*System Emotional Science of Toyama University*)

Snakes and conspecific faces are quickly detected in primates, and the amygdala (AM) has been implicated in detection of biologically relevant stimuli. In this study, we analyzed monkey AM neuronal responses to various visual stimuli including snakes. Here, we show that the monkey AM neuronal responses to snakes and conspecific faces were unique. First, the ratios of the AM neurons that responded strongly to snakes and monkey faces were greater than those of the neurons that responded strongly to the other stimuli. Second, AM neurons responded stronger and faster to snakes and monkey faces than the other categories of stimuli. Third, neuronal responses to snakes were unaffected by low-pass filtering of the images. Finally, activity patterns of responsive AM neurons discriminated snakes and emotional faces from the other stimuli in the second 50 ms period and neutral faces in the third period after stimulus onset. These response features indicate that the AM processes fast and coarse visual information of snakes and faces, and support the hypothesis that snakes and social environments have shaped the primate visual system over evolutionary time. (COI:No)

### PP-89

### Neuronal activity of the monkey medial premotor areas in a duration estimation and production task

Atsushi Chiba[1], Kazunori Morita[2], Sayuki Takara[1], Kenichi Oshio[1], Masahiko Inase[1] ([1]*Dept Physiol, Facult Med, Kindai Univ,* [2]*Dept Physiol, Iwate Med Univ*)

To investigate the neural mechanism for duration perception and generation, neuronal activity in the monkey medial premotor areas was examined during a duration estimation and production task. When a monkey pressed the hold key, a green square (C1) was presented for 0.8, 1.6, or 3.2 sec. Following a 1-sec delay period, a red square (C2) was presented and kept on until the end of the trial. The monkey was trained to keep pressing the hold key until the start of the allowed press interval, and to release it and press a target button during that interval. When the C1 was presented for 0.8 sec, the allowed press interval was between 3.2 and 4.8 sec after the C2 onset. When the C1 duration was 1.6 or 3.2 sec, that interval was 1.6 to 3.2 sec, or 0.8 to 1.6 sec, respectively. A group of neurons showed build-up activity increasing toward the release of the hold key during the retention period after the C2 onset. This activity may be related to duration production for the motor preparation. Another group of neurons exhibited differential activity during the delay period among three C1 durations. This activity may be related to categorical estimation of C1 duration. (COI:No)

### PP-90

### Development of a mouse behavioral task to investigate self-awareness during voluntary action.

Soichiro Fujiki[1], Kensaku Nomoto[1], Kenji Kansaku[1] ([1]*Department of Physiology, School of Medicine, Dokkyo Medical University*)

Understanding the sense of agency (SoA), which is "*the subjective awareness of controlling one's own volitional actions in the world*", is a fundamental issue in neurophysiology. To investigate the SoA; e.g., subjects were asked to control a cursor along target under disturbance environment, and after that, they answered questionnaires about the SoA. Neuroimaging studies in humans have revealed related brain regions; however, the neurophysiological mechanism is not well understood.
To address the issue, we developed a two-step task for mice in this study. Firstly, mice voluntarily manipulated a lever and simultaneously the system made sound, whose frequency had positive correlation to the lever angle, and disturbance sound was added in some trials. Secondly, mice choose one from two spouts to judge if there was disturbance. Correct choice gave mice water reward.
A mouse was trained over 2 weeks and its performance was examined every weekend. The mean success rate was 55.7±4.06% (n=3, ranged from 50.0 to 59.1) and the chi-squared test on the final day showed significant difference from the chance level.
This suggests that the task has a potential for investigating the SoA in mice. (COI:No)

### PP-91

### Effect of mild NMDA receptor blockage on postural control during standing and bipedal walking in Japanese macaque.

Kei Mochizuki[1], Akira Murata[1], Masahiko Inase[1] ([1]*Dept Physiol, Facult Med, Kindai Univ*)

Bipedal locomotion is essentially unstable and requires coordinated regulation of the body, presumably recruiting higher order motor areas in the cerebrum. However, traditional studies on postural control and locomotion have long focused on the role of the brain stem, basal ganglia, and cerebellum, remaining the possible contribution of the cerebral cortex unclear. In the present study, we established a bipedal locomotion task in which a macaque monkey performed sequential standing-up and walking movements on a force platform under a completely unrestrained environment. We used low-dose intramuscular ketamine injection (0.5--1.0 mg/kgBW) to induce mild NMDA receptor blockage, that has been reported to cause psychotic cognitive impairment by modifying cortical glutamatergic transmission without eliciting anesthetic effects such as drowsiness or immobilization. As a result, we found that anticipatory adjustment of center of pressure just before the initiation of bipedal walking was disturbed by low-dose ketamine administration. This suggests a unique role of the cerebral cortex in anticipatory postural control, especially during a highly unstable bipedal movement regulation. (COI:No)

### PP-92

### Autobiographical odor memory associated with an increase in connectivity between the left temporal regions and orbitofrontal cortex in elderly subjects: fMRI study.

Yuri Masaoka[1], Haruko Sugiyama[2], Keiko Watanabe[3], Masaki Yoshida[4], Akira Yoshikawa[1], Nobuyoshi Koiwa[5], Motoyasu Honma[1], Shotaro Kamijo[1], Sawa Kamimura[1], Masahiro Ida[6], Kenjiro Ono[3], Masahiko Izumizaki[1]
([1]*Department of Physiology, Showa University School of Medicine,* [2]*Sensory Science Research, Kao Corporation,* [3]*Department of Neurology, Showa University School of Medicine,* [4]*Department of Ophthalmology, Jikei Medical University,* [5]*Department of Health and Science, University of Human Arts and Sciences,* [6]*National Hospital Organization Mito Medical Center*)

Autobiographical odor memory (AM odor) represents a sense of realism of a specific memory accompanied with increases of arousal level, comfortableness, and pleasantness. Orbitofrontal cortex plays a role for olfactory identification and recognition, however, medial and occipitotemporal regions also involve to associate with memory retrieval. To test this hypothesis, 44 individuals (age range: 31~84) were tested the effect of AM odor on brain activations while undergoing functional magnetic resonance imaging (fMRI). Subjective scales of arousal levels, pleasantness and comfortableness, vividness of the memory for AM odor indicated statistically increased than control odor. All individuals were evident in activations in the left orbitofrontal cortex, left parahippocampus and occipitotemporal regions under the AM-odor stimuli. Functional connectivity analysis revealed that AM odor were associated with an increase in connectivity between left posterior orbitofrontal cortex and the left parahippocampus fusiform gyrus, especially in elderly subjects. (COI:Properly Declared)

### PP-93

### Shallow olfactory sulcus observed prior to the morphological brain changes in olfactory regions in individuals with dysosmia.

Sawa Kamimura[1,2], Yuri Masaoka[1], Nobuyuki Yoshii[3], Kouzo Murakami[4], Akira Yoshikawa[1], Masaki Yoshida[5], Motoyasu Honma [1], Shotaro Kamijo[1], Kei Sakikawa[1,2], Masahiro Ida[6], Hitome Kobayashi[2], Masahiko Izumizaki[1] (*[1]Department of Physiology, Showa University School of Medicine, [2]Department of Otolaryngology, Showa University School of Medicine, [3]Department of Radiological Technology, Showa University Hospital, [4]Department of Radiation Oncology, Showa University School of Medicine, [5]Department of Opthalmology, Jikei Medical University, [6]National Hospital Organization Mito Medical Center*)

Individuals with impaired sensory processing demonstrate morphological changes in brain regions associated with the absent sense. The purpose of the study was to test whether patients with dysosmia demonstrated changes in the thickness of gray matter, and whether these changes were associated with the duration of dysosmia. Cortical thickness in olfactory brain regions that included bilateral rectus, parahippocampus orbitofrontal cortex, and insula was similar between the patient group and the age-matched control group. However the depths of the left olfactory sulcus and circular sulcus of the insula were significantly shallower in the patient group than those of controls. It was reported that the depth of olfactory sulcus is correlated with volumes of the olfactory bulb and rectus. In addition, the posterior tip of the olfactory sulcus reaches the anterior segment of the circular sulcus of the insula. Decreases in the thickness of the left olfactory sulcus and circular sulcus of the insula may be an early sign of brain morphological changes that is identified before an actual volume reductions in the olfactory brain regions occurs. (COI:No)

### PP-94

### The facilitative role of ventral rostromedial prefrontal cortex in the execution with assistive exoskeleton robot: a functional near-infrared spectroscopy study

Trung Duc Le[1], Kazuki Watanabe[1], Hiroki Ogawa[1], Naoki Imada[2], Shingo Taki[2], Takeshi Imura[2], Yuji Iwamoto[2], Hayato Araki[3], Osamu Araki[3], Taketoshi Ono[4], Hisao Nishijo[4], Naoto Fujita[1], Susumu Urakawa[1] (*[1]Dept of Musculoskeletal Functional Res and Regeneration, Grad Sch of Biomedical and Health Sci, Hiroshima Univ, Japan, [2]Dept of Rehabilitation, Araki Neurosurgical Hospital, Japan, [3]Dept of Neurosurgery, Araki Neurosurgical Hospital, Japan, [4]Dept of Sysytem Emotional Sci, Grad Sch of Medicine and Pharmaceutical Sci, Univ of Toyama, Japan*)

Recently, robot-assisted therapy has been applied to restore limb functions in stroke patients. The human-robot interaction provides augmented feedback of aided movements to the brain forming a closed action-perception loop, thereby requiring the attentional focus on the external stimuli. The rostromedial prefrontal cortex (rmPFC) has been shown to executively prioritize external attending. In the present study, we primarily investigated the neural activity of subregions in rmPFC during the execution of an assistive exoskeleton robot. While measured by functional near-infrared spectroscopy, 26 subjects performed goal-specific upper limb tasks in 3 conditions: robot-assisted (ROB), resistive (RES), and free (FREE) torque conditions. Our result revealed that the activity of ventral rmPFC was significantly higher during ROB compared to RES and FREE. Moreover, differences in ventral rmPFC activity between ROB and other conditions (RES, FREE) were positively correlated with the differences in motor performance. These results suggest that during robot-assisted therapy, the ventral rmPFC plays an important role in orienting the attentional resources toward external stimuli. (COI:No)

### PP-95

### Roles of macaque ventrolateral prefrontal cortex in integration of expected value and risk of reward

Yuki Tamaki[1], Rena Nakayama[1], Ryo Sasaki[2], Tadashi Isa[2,3] (*[1]Sch Med, Kyoto Univ, Kyoto, Japan, [2]Dept Neurosci, Grad Sch Med, Kyoto Univ, Kyoto, Japan, [3]Inst for the Adv Study on Hum Biol, Kyoto Univ, Kyoto, Japan*)

Recent studies have shown that neurons in many brain areas represent reward-related parameters. To reveal where and how these parameters are integrated to make a decision, we investigated how macaque monkeys handle high-risk/high-return or low-risk/low-return (HH-LL) choices. We reported the causal role of the ventrolateral prefrontal cortex (vlPFC) in HH-LL decisions (Physiological Society of Japan 2020).

In this study, to understand the functional role of vlPFC to these behavioral choices, we recorded activities of vlPFC neurons while monkeys were performing HH-LL task. To quantify the neural sensitivity to HH-LL and expected value (EV) of reward magnitude, we used discrimination index (DI) to measure the overall strength of tuning relative to response variability. Most vlPFC neurons were sensitive to either HH-LL or EV. Interestingly, we found that some neurons were sensitive to both HH-LL and EV and they were highly correlated with monkey's behavioral choices.

Our findings suggest that the integration of risk/return and EV might be accomplished in vlPFC. We also investigated the functional roles of other brain areas like OFC, ACC and VTA and will report the results. (COI:No)

### PP-96

### In vivo transcranial static magnetic field stimulation over the primary motor cortex causes inhibitory changes of motor function in normal rats

Yasuyuki Takamatsu[1], Takahiro Inoue[2,3], Misato Okamura[2], Ryo Ikagemi[2], Hiroshi Maejima[1] (*[1] Department of Rehabilitation Science, Faculty of Health Sciences, Hokkaido University, Japan, [2]Grad Sch Health Sci, Hokkaido Univ, Sapporo, Japan, [3]JSPS, Tokyo, Japan*)

Transcranial static magnetic field stimulation (tSMS) has inhibitory neuromodulate effects on the human brain. Almost of the studies about static magnetic fields has been performed in vitro. For further understanding the biological mechanisms of tSMS, we investigated the effects of in vivo tSMS on motor behavior in normal rats. Wistar male rats were under deep anesthesia and exposed the skull. Polyethylene tube was attached the skull by dental cement at the center of right primary motor cortex (M1). By attaching a cylindrical NdFeB neodymium magnet into the tube, in vivo tSMS (REAL) was performed. For SHAM, we used a similar size non-magnetic stainless-steel cylinder. After 1-week recovery, all rats randomly received twice each SHAM and REAL stimulation every two days using a crossover design, and motor function was measured during the stimulation. The average value was used for each representative value. Locomotor activity and asymmetry of forelimb use didn't change, but less accurate in ladder test was shown in REAL stimulation. The results show in vivo tSMS over the M1 have inhibitory neuromodulate effects on motor function in rats. The authors have no COI to disclose. (COI:No)

### PP-97

### Cortical oxygenation in the dorsolateral prefrontal cortex during overground walking

Ryota Asahara[1], Keiji Ishii[1], Nan Liang[2], Kanji Matsukawa[3] (*[1]HIIRI, AIST, Tsukuba, Japan, [2]Dept Human Health Sci, Grad Sch Med, Kyoto Univ, Kyoto, Japan, [3]Department of Applied Clinical Research, University of Texas Southwestern Medical Center*)

It has been thought that the dorsolateral prefrontal cortex (DLPFC) is involved in executive control of behavior depending on the level of executive control demand. However, there is no evidence of the involvement of the DLPFC in executive control of walking. The present study compared oxygenated-hemoglobin concentration (Oxy-Hb, as index of cortical activity) in the DLPFC during overground walking among the three levels of walking speed (slow, normal, and fast) in 14 subjects. Furthermore, we compared the Oxy-Hb responses in the DLPFC during slow speed walking in blind condition to those during slow speed walking in normal condition. The Oxy-Hb in the DLPFC decreased during walking at slow speed. The decrease in Oxy-Hb in the DLPFC during walking was counteracted by increasing walking speed. When increasing level of executive control demand by walking in blind condition, the decrease in Oxy-Hb in the DLPFC was counteracted. Taken together, it is likely that the DLPFC may be activated during overground walking depending on the level of executive control demand. (COI:No)

### PP-98

### Single cell activity of primary motor cortex during quadrupedal vs. bipedal locomotion in Japanese monkeys

Katsumi Nakajima[1], Marc A Maier[2], Kazunori Morita[1], Takashi Suzuki[1], Masahiko Inase[3] (*[1]Dept Physiol, Sch Med, Iwate Med Univ, [2]FR3636, NRCS, Univ Paris Descartes, France, [3]Dept Physiol, Sch Med, Kindai Univ*)

To elucidate cortical mechanisms underlying the gait control, we recorded the activity of 162 cells from hindlimb/trunk regions of M1 in 2 monkeys during treadmill locomotion (~1.0 m/s), as well as EMG activity. Each cell was recorded during quadrupedal (QP) and bipedal (BP) gait. Most M1 cells modulated their activity phasically or phasic/tonically for both QP and BP gait. During BP (vs. QP) gait, M1 population activity showed higher mean discharge frequency during the step cycle (21.4±16.9 vs. 18.7±16.2Hz) and higher peak frequency (71.5 ±53.3 vs. 57.7±43.9Hz). The peak occurred predominantly during BP stance in 64% of cells (vs. 50%), particularly during the double stance and around the lift off (49 vs. 33%). Significant proportion of cells tested significantly correlated their step-by-step peak firing rate with near-simultaneous peak activity of at least one hindlimb muscle (n=21/47) or with inverse of the stance- or swing-phase duration (9/25). These results suggest that largely overlapping populations of monkey M1 cells are implicated in BP and QP gait execution and that M1 is significantly involved in generating final outputs in a step-by-step manner, via spinal circuits. (COI:Properly Declared)

### PP-99

### Changes in the corticospinal excitability accompany a short-term motor skill learning of a coordinated upper limb movement in humans

Nan Liang[1], Hajime Ueda[1], Amiri Matsumoto[1], Reina Tanka[1], Keisuke Irie[1] ([1]*Cognitive Motor Neuroscience, Graduate School of Medicine, Kyoto UniversityDepartment of Human Health Sciences*)

Although it is known that motor performance improves through motor skill learning, it is unclear how the corticospinal excitability changes through the learning process. By using transcranial magnetic stimulation (TMS), we examined the changes in the corticospinal excitability of the engaged muscles before and after a short-term learning of a dynamic and coordinated upper limb movement. Healthy volunteers using their dominant hand were asked to hold a pen and dot (black ink) the center of an A4-size paper at which a small red ink dot was printed. The subjects performed the trials at 1 Hz, and were asked to put their hand higher than 100 mm apart from the paper surface trial-by-trial. Fifty trials per session, totally 20 sessions were performed. The error from the target significantly decreased through the learning. The motor evoked potential (MEP)/B.EMG or MEP to TMS in the FDI significantly increased when performing or mental imaging the task, while those in flexor carpi radialis and extensor carpi radialis muscles had no changes. Our results suggest that the motor command to distal, rather than proximal, muscle changes by skill learning of a coordinated upper limb movement. (COI:No)

### PP-100

### Responses of labeled mechanoreceptors to high-frequency vibrations.

Satomi Ebara[1,2], Aya Takenaka[2], Atsuishi Yoshida[2], Takahiro Furuta[2] ([1]*Anatomy, Meiji Univ. Integrative Med.*, [2]*Oral Anat. 2nd, Dent., Osaka Univ*)

Responses of trigeminal primary sensory neurons were investigated using intra-axonal recording and labeling of thetrigeminal tract of the rat brainstem in-vivo. This method displayed superior aspects of low invasiveness coupled with easiness of operation (Furuta et al., 2020, Tonomura et al., 2015). A mechanical stimulator was equipped with 2 speaker units capable of 4 different dimensional vibrations of single mystacial vibrissae. Vibratory stimulation with 10 sets of different vibrations (20-800 Hz, 1.0 second respectively) induced phase-locked responses on each kind of mechanoreceptor neurons of two types of Merkel endings, at the rete ridge collar and inside of the follicle, as well as lanceolate or club-like endings at the level of the follicles. Full observations of the reconstructed tissues showed the relationships between the exact positions of labeled mechanoreceptors in the follicles and responsiveness among a variety of stimulations. In accord with the previous study of Furuta et al. (2020), our observation implies mobility of detailed structures of the exact positions generated by vibratory stimulation of the whiskers. (COI:No)

### PP-101

### Effect of Glucagon-like peptide-1 receptor agonist, liraglutide, against diabetic retinal edema in spontaneously diabetic tori fatty rats.

Kazuho Inoue[1], Shohei Yamada[2], Seiko Hoshino[1], Yugo Shibagaki[2], Atsuko Ikemori[1] ([1]*Department of Anatomy, St. Marianna University School of Medicine*, [2]*Division of Nephrology and Hypertension, Department of Internal Medicine, St. Marianna University School of Medicine*)

**Background:**
The aim of this study is to investigate the effect of glucagon-like peptide-1 (GLP-1) receptor agonists (GLP-1RA), liraglutide, against diabetic retinal edema using an animal model of type 2 diabetes.
**Methods:**
Male spontaneously diabetic torii (SDT) fatty rats at 8 weeks of age were randomly assigned to three groups; the liraglutide group has been subcutaneous injected liraglutide for 8 weeks. Other group was provided both insulin against hyperglycemia and hydralazine against hypertension for matching both levels of blood glucose and blood pressure with the liraglutide group. A control group was injected only a vehicle.
**Results:**
Control group exhibited hyperglycemia and hypertension, whereas two treatment groups similarly reduced both blood glucose and blood pressure levels. While retinal thickening for the retinal edema was found in control group, the degree was significantly prevented in the liraglutide group. Furthermore, the effect was also brought by the treatments of both insulin and hydralazine.
**Conclusions:**
Liraglutide prevented the retinal edema characterized in the early phase of diabetic retinopathy via reduction of both blood glucose and blood pressure levels. (COI:No)

### PP-102

### Synergic interaction of nerve growth factor (NGF) and glial cell-line derived neurotrophic factor (GDNF) in developing muscular hyperalgesia and its anatomical basis in rats

Kazue Mizumura[1], Kimiko Kobayashi[2], Shiori Murase[3] ([1]*Department of Physiology, Nihon University School of Dentistry*, [2]*Department of Anatomy and Neuroscience, Hyogo College of Medicine*, [3]*Nihon Bioresearch*)

Previously we found that NGF and GDNF play pivotal roles in generation of muscular mechanical hyperalgesia after lengthening contraction (DOMS) in rats. We examined in this experiment whether and where collaboration of both neurotrophic factors occurs in SD rats. I.m. injection to gastrocnemius muscle (GC) of a mixture of NGF (0.1 μM) and GDNF (0.008 μM), which by itself alone had no effect, induced a significant mechanical hyperalgesia ($p < 0.001$), demonstrating that NGF and GDNF are synergistic. PERK immunoreactivity in dorsal root ganglion (DRG) neurons induced by muscle compression increased after the mixture injection ($p < 0.05$), thus the interaction of NGF and GDNF could occur at the primary afferent level. *In situ* hybridization study demonstrated that 23.7 % to 29.2% of GC DRG neurons (identified with Fluoro-Gold) coexpress TrkA (NGF receptor) and GFRa1 (GDNF receptor). Cell size of coexpressing neurons distributed widely from small to large size ranges. Some of these coexpressing neurons having thin axons are thought to contribute to muscle mechanical hyperalgesia. These results provide basis of synergism of NGF and GDNF that would occur in natural condition such as DOMS. (COI:No)

### PP-103

### Detection of hemodynamic cortical network derived from electrophysiological activity by simultaneous EEG and fNIRS measurements

Keita Tanaka[1], Swethasri Dravida[2,3], Jack Adam Noah[3], Xian Zhang[3], Joy Hirsch[3], Yumie Ono[4] ([1]*Electrocal Engineering Program, Grad. Sch. of Science and Technology, Meiji Univ.*, [2]*Interdepartmental Neuroscience Program, Yale Sch. of Medicine.*, [3]*Dep. of Psychiatry, Yale Sch. of Medicine.*, [4]*Dep. of Electronics and Bioinformatics, Sch. of Science and Technology, Meiji Univ*)

We investigated visually-evoked metabolic cortical network that corresponds to electrophysiological activities of a specific frequency band. Twenty healthy young adults performed passive picture viewing task under simultaneous recording of 32-channel electroencephalography (EEG) and 134-channel functional near-infrared spectroscopy (fNIRS). The alpha-band EEG activities were used as model functions for the general linear model analysis of fNIRS data to determine the EEG-related metabolic cortical activity. We applied a multivariate Granger causality analysis to determine causal relationship among electrophysiological and metabolic activities. The prefrontal alpha activity was associated with metabolic network involving the extrastriate visual areas (V2 and V3), frontal eye field, superior temporal gyrus, and dorsolateral prefrontal cortices (DLPFC). The occipital alpha activity was associated with metabolic network involving the V2 and DLPFC. The proposed EEG-fNIRS analysis demonstrated reasonable visual network without a priori assumptions, suggesting its detectability of complex information processing in the brain organized by electrical activities of multiple frequency bands. (COI:No)

### PP-104

### Effect of reactive nitrogen species on the tight junctional localization of claudin-1 in human skin keratinocytes

Shokoku Shu[1], Mao Kobayashi[1], Kana Marunaka[1], Toshiyuki Matsunaga[2], Yuta Yoshino[1], Akira Ikari[1] ([1]*Lab. Biochem., Gifu Pharm. Univ.*, [2]*EGPS, Gifu Pharm. Univ*)

The stratum corneum in skin plays an important role in preventing intrusion of the foreign substances and water transpiration from the body. Ultraviolet (UV) reduces the barrier function of the skin, but the mechanism has not been well clarified. We examined the effect of UVB irradiation on the cellular localization of claudin-1 (CLDN1), a component of the tight junction (TJ) strand using human keratinocyte-derived HaCaT cells. The localization of CLDN1 and transepidermal electrical resistance (TER) were decreased by UVB irradiation. The mislocalization of CLDN1, the reduction of TER and the enhancement of lucifer yellow flux by UVB irradiation were inhibited by monodansylcadaverine, a clathrin-dependent endocytosis inhibitor. Furthermore, UVB increased the concentrations of intracellular $Ca^{2+}$, nitric oxide (NO), and peroxynitrite contents, which were inhibited by knockdown of Opsin2 (OPN2), a light sensitive protein. These results suggest that UVB irradiation facilitates the endocytosis of CLDN1, through the activation of OPN2, $Ca^{2+}$ entry, production of NO and peroxynitrite contents. The inhibition of reactive nitrogen species signal may be effective to maintain skin barrier. (COI:No)

### PP-105

### Mechanical hyperalgesia induced by a high-fat-cholesterol diet in SHRSP5/Dmcr rats: possible involvement of fatty acids in serum

Yasuko Kozaki[1], Tomomitsu Miyazaki[1], Daisuke Miyazawa[1], Kazuya Kitamori[2] (*[1]College of Pharmacy, Kinjo Gakuin Univ., [2]College of Human Life and Environment, Kinjo Gakuin Univ*)

As previously reported, the intake of a high-fat-cholesterol diet (HFC), which contains 25% palm oil and 5% cholesterol, induced hyperalgesia in SHRSP5/Dmcr rats (kozaki et al., 2013). In this study, we examined whether fatty acids (FA) content in serum is involved in HFC-induced hyperalgesia. Adult male SHRSP5/Dmcr rats were fed with a control diet (crude fat: 4.8%) for several weeks, then divided into two groups, control group (fed with the control diet throughout the experiment, n=6) and HFC group (fed with HFC for 10 days, n=6). In HFC group, the thresholds for mechanical pain responses were significantly decreased from 3 to 10 days (Day10) after the start (Day0) of HFC ($p<0.01$ vs. Day0). The FA content in serum at Day10 was measured by gas chromatography and compared with that in control group. The total FA content in HFC group was almost unchanged from control group. However, the levels of 20:4n-6 and 22:6n-3 in HFC group were significantly lower than in control group and levels of 16:0 and 18:1 were significantly higher ($p<0.05$). At least some of these FA contents in serum may play key roles in hyperalgesia induced by HFC. [Research funds: KAKENHI 18K07399] (COI:No)

### PP-106

### GABAergic and glycinergic systems regulate ON-OFF electroretinogram by cooperatively modulating cone pathways in the amphibian retina

Hajime Hirasawa[1], Naofumi Miwa[1], Shuichi Watanabe[1] (*[1]Dept Physiol. Saitama Med Univ*)

The network mechanisms underlying how inhibitory circuits regulate ON- and OFF-responses (the b- and d-waves) in the electroretinogram (ERG) remains unclear. To investigate the contribution of inhibitory circuits to the emergence of the b- and d-waves in the full-field ERG, we investigated the effects of several synaptic transmission blockers on the amplitudes of the b- and d-waves in the ERG obtained from newt eyecup preparations. Our results demonstrated that (i) SR95531 (SR) augmented both the b- and the d-wave, indicating that GABAergic lateral inhibitory circuits inhibit both ON- and OFF-BC pathways; (ii) the administration of strychnine in the presence of SR attenuated the d-wave, and this attenuation was prevented by blocking ON-pathways with L-APB, which indicated that the glycinergic inhibition of OFF-BC pathway is downstream of the GABAergic inhibition of the ON-system; and (iii) the glycinergic inhibition from the ON- to the OFF-system widens the response range of OFF-BC pathways, specifically in the absence of GABAergic lateral inhibition. Based on these results, we proposed a tentative explanation of the circuitry mechanisms underlying ON-OFF ERG formation. (COI:No)

### PP-107

### Light stimulation induces biosynthesis of endocannabinoids and N-acyl amide in photoreceptor cells in *Drosophila*

Takaaki Sokabe[1], Heather Bradshaw[2], Craig Montell[3] (*[1]Thermal Biology Group, ExCELLS, Japan, [2]Dept. of Psychological and Brain Sciences, Indiana Univ., USA, [3]Dept. of Molecular, Cellular, and Developmental Biology and the Neuroscience Research Institute, UCSB, USA*)

The phototransduction in *Drosophila* represents a good model for understanding intracellular signaling involving GPCR and TRP channels. Despite numerous efforts, however, the regulation of TRP channel gating downstream of phospholipase C (PLC) activation has not been conclusive. Since PLC activation leads to synthesis/degradation of various lipids, we approached to this conundrum by proving the changes in lipid metabolites in photoreceptor cells. Here we found that light stimulation facilitated the increases of multiple linoleoyl-conjugated lipids including endocannabinoids (2-acylglycerol and anandamide) and N-acyl glycine in fly heads. The increases of those lipids strictly depended on PLC activation, and all the lipids enhanced the activity of TRP channels both in heterologous expression system and dissociated photoreceptor cells. This study shed a light on potential roles of uncharacterized lipid metabolites in *Drosophila* phototransduction and emphasizes an importance of lipids as a regulator in signaling cascades.
(COI:Properly Declared)

### PP-108

### The roles of the intracellular C-terminal domain in mGluR6 cell surface localization

Takumi Akagi[1], Dilip Rai[2], Atsushi Shimohata[1], Toshiyuki Ishii[1], Mie Gangi[1], Takuma Maruyama[1], Yuko Kiyama[1], Ikuo Ogiwara[1], Makoto Kaneda[1] (*[1]Dept Physiol, Nippon Med Sch, [2]Dept of Rehabilitation for Sensory Functions*)

Metabotropic glutamate receptor 6, mGluR6, is specifically expressed in the retinal ON-bipolar cells, and plays a critical role in the processing of retinal visual signals. The intracellular mechanisms of mGluR6 surface localization and receptor function remain elusive. We have recently tested localization of C-terminally truncated mGluR6 in 293T cells and primary hippocampal neuron cultures using immunocytochemistry and electrophysiology (Rai et al. J Neurochem. In press). We have proposed that the CTD may be involved in the regulation of receptor intracellular trafficking and signaling. We here examined another series of mGluR6 deletions, in which amino acids were sequentially removed from the N-terminal side of CTD. To our surprise, mGluR6 surface localization was not markedly affected until the entire CTD was deleted. We also tested a mutant, in which CTD consisted of one alanine, and found that the mutant showed less obscure surface localization compared to that containing one lysine at CTD. These observations suggest that the basic amino acids in CTD are preferable for efficient mGluR6 surface localization. (COI:No)

### PP-109

### The effect of catechin contained in green tea on temporal characteristic of optokinetic responses in mice

Yuko Sugita[1], Takahisa Furukawa[1] (*[1]Laboratory for Molecular and Developmental Biology, Institute for Protein Research, Osaka University, Osaka Japan*)

While it is generally considered that green tea has positive effects on human body and mental health, whether and how green tea affects the visual function is still unclear. In the present study, we examined the effects of green tea on visual function for optokinetic responses (OKRs) in mice after we administrated green tea to mice by feeding the food containing 2% of green tea for one month. From these analyses, we observed the effect of green tea contains the component increasing the optimal temporal frequency in OKR.
Furthermore, we administered epigallocatechin gallate (EGCG), the most abundant catechin contained in green tea, to mice intraperitoneally and measured OKR.We found that the OKR of the mice after EGCG administration became higher in temporal sensitivity than before administration. This was consistent with the large response of OKR when ingesting green tea.
From the above results, the visual function for those moving by ingesting green tea was enhanced, which may be related to the effect of EGCG. (COI:No)

### PP-110

### Gender differences in the development of non-inflammatory masticatory muscle hypersensitivity in rats.

Asako Kubo[1,2], Shiori Sugawara[3], Koichi Iwata[1] (*[1]Dept Physiol, Sch Dent Nihon Univ, [2]Div Cell Signaling, National Institute for Physiological Sciences, [3]Dept Pharmacol, Sch Dent Nihon Univ*)

The animal model of masticatory muscle (MM) hypersensitivity was developed by electric contraction of masseter and injection of nerve growth factor to trapezius muscle in SD rats. We studied the mechanical head-withdrawal threshold (MHWT) in masseter and neuronal activity in brain stem in both male and female *model* rats. Significant reduction of the MHWT to masseter muscle stimulation was observed in female but not male model rats on days 9 to 12 during 10 days repetitive contraction and injection. The distribution of phosphorylated extracellular signal-regulated kinase-immunoreactive (pERK-IR) neurons in the brain stem was examined on day 12. The number of pERK-IR neurons in the trigeminal spinal subnucleus interpolaris/caudalis transition region (Vi/Vc) at the side ipsilateral to the stimulation in the group stimulated both was significantly higher than the groups stimulated only one of them in female but not male rats. The enhancement of neuronal activity in the Vi/Vc could elicit discomfort and masticatory dysfunction associated with MM hypersensitivity in female. (COI:No)

### PP-111

### Two types of Cl transporters contribute to the intracellular Cl concentrations in ON- and OFF-type bipolar cells in the retina

Chengzhu Yin[1], Toshiyuki Ishii[1], Makoto Kaneda[1] (*[1]Dept. Physiol., Nippon Medical School, Tokyo, Japan*)

In the retina, ON- and OFF-type bipolar cells (ON- and OFF-BCs) have concentric center-surround receptive field. Currently, the mechanisms giving rise to surround responses remain unclear. One hypothesis for surround responses is that intracellular Cl concentrations ($[Cl^-]_i$) are set at different levels to achieve opposite polarities for GABA responses in ON- and OFF-BCs. To investigate this hypothesis, we examined the distribution and function of the Cl transporters, NKCC1 and KCC2, in ON- and OFF-BCs using immunohistochemical, *in situ* hybridization, and electrophysiological methods. Although KCC2 and NKCC1 were detected by mRNA and protein analyses in both ON- and OFF-BCs, the number of cells which expressing NKCC1 was more in ON-BCs than in OFF-BCs. In addition, the action of NKCC1 antagonist was larger in ON-BCs than in OFF-BCs, while the action of KCC2 antagonist was larger in OFF-BCs than in ON-BCs. These results mean that $[Cl^-]_i$ of ON-BCs is controlled by NKCC1 and that of OFF-BCs is controlled by KCC2, and $[Cl^-]_i$ is higher in ON-BCs than in OFF-BCs. Thus, the difference of $[Cl^-]_i$ would contribute to the formation the surround responses. (COI:No)

### PP-112

### Neuronal activity in ventral tegmental area during reaching task in rats.

Taichi Goto[1,3], Ichiro Takashima[2,3], Nobuo Kunori[1] (*[1]Neurorehabilitation Research Group, Human Informatics Research Institute, National Institute of Advanced Industrial Science and Technology, [2]Integrative Neuroscience Research Group, Human Informatics Research Institute, National Institute of Advanced Industrial Science and Technology, [3]Master's and Doctoral Programs in Neuroscience, Degree Programs in Comprehensive Human Sciences, Graduate School of Comprehensive Human Sciences, University of Tsukuba*)

Dopaminergic neuron in the ventral tegmental area (VTA) is known to involve sensorimotor functions, but the significance of the VTA in sensorimotor functions is not well known. To investigate this issue, we recorded the neuronal activity of the VTA during reaching task by using *in vivo* calcium imaging.

For measuring the neuronal activity, the adeno-associated virus contained fluorescent calcium indicator was injected into the VTA in rats and recorded fluorescent signal from the VTA through the optical fiber while rats performed reaching task. The dF/F calcium signal was analyzed between the times when the rats protruded and extracted their hand from the slit.

The present data demonstrated that the calcium signal in the VTA was increased when the rats grasped food pellet. The increasing calcium signal was back to the baseline level when the rats dropped food pellet during reaching behavior. In addition, the calcium responses were not observed when the rats reached to the food port without the pellet. These results suggest that increasing the calcium response in the VTA during reaching task can be derived from the sensorimotor aspects generated by the interactions with food pellet. (COI:No)

### PP-113

### Analysis of swallowing patterns during unmatched olfactory-taste stimulation

Saori Maeda[1], Takako Fukuda[2], Shintaro Kusunoki[3], Hiroyuki Kanayama[2], Yuji Miyaji[2], Hiroshi Yoshimura[1] (*[1]Department of Molecular Oral Physiology, Institute of Biomedical Sciences, Tokushima University Graduate School, [2]Graduate School of Oral Sciences, Tokushima University, [3] Graduate School of Sciences and Technology, Tokushima University*)

There are some cases that odors affect subjective taste perception. We previously reported that when we receive odors that do not match with foods being consumed, subjective feelings are disturbed and theta-band brain activity is increased while the unmatched information is cross-checked. In the study, each odor stimulation was delivered while the subject was tasting chocolate, using chocolate paste as the odorant for matched odor stimulation, and garlic paste for unmatched odor stimulation. In the present study, we focused attention on oral movement and swallowing pattern in the same situation with our previous study. We recorded surface electromyogram (EMG) from masseter and suprahyoid muscle around. In addition, sound waveform was recorded simultaneously from the neck to confirm swallowing time. Analysis of the EMG and sound waves revealed that swallowing timing and EMG waveform were different between the cases of matched and unmatched odor stimulation, and that those patterns seemed to be different among individuals. These results suggest that when we receive unmatched odors with the foods being consumed, swallowing central pattern generator might be disturbed. (COI:No)

### PP-114

### Analysis of single unit responses by prosthetic retinal stimulation

Tomomitsu Miyoshi[1], Takeshi Morimoto[2], Takashi Fujikado[3] (*[1]Dept Integrat Physiol, Grad Sch Med, Osaka Univ, [2]Dept Advanced Visual Neurosci, Grad Sch Med, Osaka Univ, [3]Grad Sch Front Biosci, Osaka Univ*)

We have been developed the novel retinal prosthetic system, Suprachoroidal Transretinal Stimulation (STS), for photoreceptor degeneration. Previously we reported that the burstic spike response by STS occurred alternately on ON and OFF cells, and that inhibition after the first burst might form the spiking activity into burstic pattern, by using double pulse stimulation (DPS) (PSJ meeting, 2018). The number of the recorded units, however, was small to analyze quantitatively. Here we analyzed them with additional samples.

An electrode array for STS was implanted into scleral pocket of anesthetized cat, and single units were recorded from lateral geniculate nucleus. The size of single electrode in the array was 0.5 mm in diameter and 0.3 mm in height. The stimulation parameter was 0.5 or 1 mA amplitude, 0.5 ms/phase duration, biphasic. The interval of DPS was changed from 5 to 50 msec.

The preceding stimulation in DPS inhibited the first burst elicited by the next stimulation for all 4 cell types of ON-Y, ON-X, OFF-Y, and OFF-X cells. The inhibition on ON cells was faster than that on OFF cells. These results showed that the burstic response is made by the inhibition after STS. (COI:Properly Declared)

### PP-115

### Relationship between control quality and sense of agency during robot hand illusion of elbow movement

Toshihiro Kawase[1,2,3], Soichiro Fujiki[1], Toshimitsu Takahashi[1], Kenji Kansaku[1,4] (*[1]Dept of Physiol, Dokkyo Med Univ Sch of Med, Mibu, Japan, [2]Inst Biomater & Bioeng, Tokyo Med Dent Univ, Tokyo, Japan, [3]Inst Innov Res, Tokyo Inst of Tech, Yokohama, Japan, [4]Cent Neurosci & Biomed Eng, Univ of Electro-Communications, Chofu, Japan*)

We formerly reported that a sense of ownership (SO) and a sense of agency (SA) were extended to robotic arm controlled by electromyography (Sato et al., 2018). In this study, we investigate the relationship between control quality and sense of agency during robot hand illusion of elbow movement. The robotic arm with one degree of freedom (elbow flexion and extension) was controlled by means of the participant's muscular activity on the elbow flexor and extensor, following the joint position estimated by a feed-forward neural network (NN). The participants took part in the calibration of the NN and the in-phase and out-of-phase movement conditions for 90 sec each. The able-bodied participants (n=4) answered a questionnaire to assess SA immediately after each experiment. The task was carried out two times. The ratings of SA were significantly correlated with the estimation accuracy of the NN in the in-phase movement (Spearman: $r = -0.89$, $p = 0.01$) and in the out-of-phase movement (Spearman: $r = -0.84$, $p = 0.01$). The preliminary results suggest that the robotic arm will contribute to our understanding of our experience on our bodies. (COI:No)

### PP-116

### Characterization of insect repellents targeting TRP channels

Shoma Sato[1], Takaaki Sokabe[1] (*[1]Thermal Biology Group, Exploratory Research Center on Life and Living Systems*)

Transient receptor potential (TRP) channels expressed in sensory neurons are activated by various physical and chemical stimuli and play key roles in nociception and aversive responses in many species. In the fruit fly, *Drosophila melanogaster*, TRPA1 and other TRP channels participate in chemical and thermal nociception. In this study, we examined whether TRP channel activators can modulate behavioral responses of *Drosophila*. An electrophile, *N*-methylmaleimide (NMM) is one of the direct ligands of TRPA1 and induces gustatory aversion in flies. We performed a two-choice assay in which starved-flies choose sucrose containing agarose in the presence or absence of NMM. We observed avoidance responses against NMM in a dose-dependent manner in the wild-type flies, whereas the avoidance was disappeared in TRPA1 mutant flies. Interestingly, we found that an application of a fatty acid in the agarose augmented the effect of NMM. We propose a novel strategy for insect pest management aiming at TRP channels as a target. (COI:No)

### PP-117
### Olfactory marker protein (OMP) contributes to sharpening the juxtagluomerular activities in olfactory bulb during the olfactory investigation.

Akiko Nakashima[1], Taku Nakagawa[1,2], Makoto Takano[1], Noriyuki Nakashima[1] ([1]*Department of Physiology, Kurume University School of Medicine, 67 Asahi-machi, Kurume, Fukuoka, 830-0011, Japan.*, [2]*Department of Anaesthesiology, Graduate School of Medicine, Kyushu University, Fukuoka, 812-8582, Japan*)

Olfaction starts from olfactory receptor neurons (ORNs) that express olfactory marker protein (OMP). The OMP-deficit mice show various olfactory dysfunction due to the impaired responses of ORNs. Recently, OMP has been demonstrated to maintain persistent olfaction by buffering the sensory-evoked cAMP signalling. However, the impact of OMP on olfaction behaviours, which require time to evaluate odour values, remains largely unexplained.
Here, we examined the behaviours of heterozygous $OMP^{+/GFP}$ (HET) mice vs. homologous GFP-knock-in OMP-deficient $OMP^{GFP/GFP}$ (KI) mice during the olfactory investigation of odours with different values. KI mice were capable of sensing the ambient odours. KI mice took longer time to evaluate Reward-related odour vs. Penalty-related odour. Histologically, c-Fos-expressing juxtaglomerular cells were fewer and more broadly distributed around glomeruli in KI mice than HET mice after the task. In conclusion, OMP contributes to the evaluation of odour values by glomerular processing during an olfactory investigation task. (COI:No)

### PP-118
### Suppression of the GABAergic transmission in the mouse accessory olfactory bulb by the vasopression receptor through inhibition of voltage-activated Ca currents

Mutsuo Taniguchi[1], Yoshihiro Murata[1], Masahiro Yamaguchi[1], Hideto Kaba[1] ([1]*Dept. Physiol., Kochi Med. School, Kochi Univ.,*)

Central vasopressin (AVP) facilitates social recognition and modulates many complex social behaviors in mammals. By measuring the reciprocal synaptic currents (IPSCs) from mitral cells (MC) in the accessory olfactory bulb (AOB), the first relay in the vomeronasal system, we have demonstrated that AVP significantly reduced the IPSCs via V1a receptors. The reciprocal transmission, however, contains both glutamatergic transmission from MCs to granule cells (GCs) and GABAergic one from GCs to MCs. Thus, it is unclear whether AVP acts on the excitatory and/or the inhibitory transmissions.
In the present study, to investigate the role of V1a receptors in the GABAergic transmission, AOB slices were prepared from 23- to 35-day-old Balb/c mice. Using the whole-cell voltage clamps, the current response of GCs or MCs was recorded in the presence of antagonists for glutamatergic transmission, CNQX and AP5. While extracellular application of AVP did not affect the magnitude of the response of MCs to GABA, the manipulation slightly suppressed voltage-activated $Ca^{2+}$ currents in the GCs. The results suggest that AVP reduces the GABAergic transmission through the inhibition of $Ca^{2+}$ channels on GCs. (COI:No)

### PP-119
### Effects of estrogen and vagotomy on the excitability of nociceptive neurons in female rats

Akimasa Tashiro[1], Yuji Morimoto[1] ([1]*Department of Physiology, National Defense Medical College, Japan*)

Temporomandibular joint (TMJ) pain is most prevalent in women and estrogen may be risk factor in TMJ disorders (TMD). Autonomic and endocrine dysfunctions are cited among the signs and symptoms of TMD pain. Although estrogen status is a risk factor in painful TMD, it is not known if vagal afferents play a significant role in TMJ nociceptive processing under different estrogen status condition. Under isoflurane anesthesia, TMJ-responsive units were recorded in superficial lamina at the Vc/C1-2 junction from ovariectomized (OvX) female rats treated for 2days with low dose estradiol (LE2, 4μg/day), high dose estradiol (HE2, 40μg/day) naïve rats or bilateral cervical vagotomy (VgX) condition.TMJ units were activated by ATP (0.01-1mM) injected into the TMJ joint space. In LE2 rats, VgX enhanced ATP evoked response of units compared to naïve rats. By contrast, in HE2 rats, VgX had only minor effects on ATP-evoked response. Thus, VgX enhanced TMJ-evoked units responses in LE2 but not HE2. These data support the hypothesis that E2 status modulate vagal nerve evoked antinociception in deep craniofacial pain condition such as TMD. (COI:No)

### PP-120
### Anatomical and electrophysiological analysis of cholinergic inputs from the parabigeminal nucleus to the superficial superior colliculus in mice

Kota Tokuoka[1,2,3,4], Masatoshi Kasai[1,3], Kenta Kobayashi[4,5], Tadashi Isa[1,3,4,5,6,7] ([1]*Dept of Neuroscience, Grad Sch of Med, Kyoto Univ*, [2]*Grad Sch of Biostudies, Kyoto Univ*, [3]*Dept of Developmental Physiology, NIPS*, [4]*Dept of Physiological Sciences, Sch of Life Sciences, Graduate University of Advanced Studies [SOKENDAI]*, [5]*Sect of Viral Vector Development, NIPS*, [6]*WPI-ASHBI*, [7]*Human Brain Research Center, Grad Sch of Med, Kyoto Univ*)

Superior colliculus (SC) is a key structure for visually guided behaviors. Its retinorecipient superficial layers (sSC) receive cholinergic inputs from the parabigeminal nucleus (PBN). However, the details of the cholinergic inputs from the PBN to the sSC are elusive. We visualized and optogenetically manipulated the PBN cholinergic neurons using Cre-dependent gene expression technique in mice. We found that the cholinergic projections terminated densely in the medial sSC, which encode upper visual field. Since upper visual field is critical for survival from predators, the cholinergic inputs may contribute to the detection of visual threat. Then, we recorded looming-evoked visual responses from sSC, and tested the effect of the optogenetic activation and inactivation of PBN cholinergic neurons. We found that optogenetic manipulations in either direction induced response suppression in most neurons, whereas response facilitation was observed in a few neurons after the optogenetic activation. Based on these results we propose a circuit model in which the PBN cholinergic inputs enhance the visual signal processing in the sSC by facilitating the center excitation–surround inhibition. (COI:No)

### PP-121
### Angiotensin II receptors and the epithelial sodium channel in orofacial sensory ganglia of rats

Takeshi Suwabe[1], Toshiaki Yasuo[1], Noritaka Sako[1], Fumihiko Nakamura[1] ([1]*Dept Oral Physiol, Sch Dent, Asahi Univ, Japan*)

Angiotensin II receptors and the epithelial sodium channel (ENaC) have been reported to regulate neuronal excitability in taste cells. To elucidate the role of the Angiotensin II receptors and ENaC in the orofacial sensory system, we investigated gene expression of renin-angiotensin system, angiotensin II receptors and ENaC in the rat trigeminal and geniculate ganglion. Trigeminal and geniculate ganglia were collected from anesthetized rats, total RNA was extracted from these ganglia, and cDNA was synthesized from RNA templates by reverse transcription. Gene expression levels were determined by real-time PCR. Expression of angiotensinogen, renin, ACE, ACE2, angiotensin II type 1a, type 1b and type 2 receptor and the α subunit of ENaC mRNAs was observed in both the trigeminal ganglion and the geniculate ganglion. This result suggests that the neural circuit in the orofacial sensory system may be regulated via activation of angiotensin II receptors and ENaC in the trigeminal and geniculate ganglia. (COI:No)

### PP-122
### Cytokine imbalance affects microglial activity in cerebral cortex

Tetsuya Sasaki[1,2], Yosuke Takei[1,2] ([1]*Dept. Anat&Neurosci, Fac Med, Univ. Tsukuba*, [2]*Ph.D Program of Neurosciences, Graduate School of Comprehensive Human Sciences, University of Tsukuba*)

Viral infection during pregnancy has been suggested to increase probability of autism spectrum disorder in offspring. This phenomenon has been modeled in rodents subjected to maternal immune activation (MIA). Previous studies showed that maternal T helper 17 cells and the effector cytokine interleukin-17A (IL-17A) play a central role in MIA-induced behavioral abnormalities and cortical dysgenesis called cortical patch in offspring. However, it is unclear how IL-17A acts on fetal brain cells to cause ASD pathologies. To assess the effect of IL-17A on cortical development, we performed direct administration of IL-17A into lateral ventricles of fetal mouse brain. We analyzed injected brain focusing on microglia, which express IL-17A receptors. We found that IL-17A activated microglia and altered their localization in the cerebral cortex. Our data suggest that IL-17A activates cortical microglia, which could lead to a series of ASD-related brain pathology, including excessive phagocytosis of neural progenitor cells in the ventricular zone. (COI:No)

### PP-123

### Cellular and temporal pattern of endoplasmic reticulum stress response after brain injury

Qiyan Fan[1], Mika Takarada-Iemata[1], Osamu Hori[1] (*[1]Department of Neuroanatomy, Graduate School of Medical Sciences, Kanazawa University*)

The endoplasmic reticulum (ER) is the major organelle for protein synthesis and maturation, and disturbance of ER protein homeostasis leads to impairment of cellular function and induces unfolded protein response (UPR). Accumulating evidence suggests a crucial role of UPR in neuropathological conditions. However, it is not fully understood when and in which cells the UPR is induced during pathogenesis. In this study, we investigated cellular and temporal pattern of the UPR after brain injury using stab injury model in ER stress-activated indicator (ERAI) mice, which enable to monitor UPR by the fluorescence of spliced XBP-1 fused with Venus. Fluorescent signals increased over time in the ipsilateral cortex after brain injury from 6 hours to 7 days in ERAI mice. They were observed in injured neurons in the early stages after brain injury. However, the major cells positive for fluorescent signals were non-neuronal cells such as vascular cells and astrocytes throughout the period analyzed after brain injury. These results suggest that UPR may play important roles not only in neurons but also in the non-neuronal cells in the course of neurological diseases including brain injury. (COI:No)

### PP-124

### Enhanced quiescence of neural stem cells in the hippocampus by short-term exposure to cuprizone, a mouse model for schizophrenia

Tomohiro Ohgomori[1,2], Shozo Jinno[2] (*[1]Department of Rehabilitation, Osaka Kawasaki Rehabilitation University, [2]Department of Anatomy and Neuroscience, Graduate School of Medical Sciences, Kyushu University*)

Schizophrenia is a severe and chronic mental disorder characterized by cognitive dysfunctions, delusions, and hallucinations. In this study, we analyzed short-term cuprizone (CPZ)-exposed mice, which were shown to exhibit schizophrenia-like behaviors accompanying the abnormal adult hippocampal neurogenesis. We first confirmed that 2-week 0.2% CPZ exposure induced the impairment of prepulse inhibition of the acoustic startle. The densities of microglia in the dentate gyrus were increased by the short-term CPZ exposure, but those of oligodendrocyte precursor cells and mature oligodendrocytes remained unchanged. The expression ratios of phosphorylated signal transducer and activator of transcription 3 (STAT3) in neural stem cells in the dentate gyrus were increased by the short-term CPZ exposure. In addition, short-term CPZ exposure increased the relative proportions of neural stem cells, but decreased the neuronal progenitors and newborn granule cells. These results suggest that short-term CPZ exposure may enhance the quiescence of neural stem cells in the hippocampus via phosphorylation of STAT3, which may be related to the pathophysiology of schizophrenia. (COI:No)

### PP-125

### Translocation of HMGB1 from nucleus to cytoplasm in neurons damaged by blast-induced mild traumatic brain injury (bmTBI)

Takahito Higashi[1], Kiyomasa Nishii[1], Yasushi Satoh[2], Yasushi Kobayashi[1] (*[1]Natl. Def. Med. Co., Dept. Med., Anato. and Neurobiol., [2]Natl. Def. Med. Col., Dept. Med., Biochem*)

A mild traumatic brain injury is one of the most important unsettled issues in explosion accidents and terrorism. Previously we showed that a shock wave exposure by a shock tube induced BBB disruption in several hours. However, detailed mechanisms of the injury remain unclear. We focus on a ubiquitous nuclear protein HMGB1, which belongs to the damage associated molecular patterns (DAMPs). HMGB1 is translocated from nuclei to cytoplasm, released from necrotic and inflammatory cells and induces an inflammatory response. Here we investigated the translocation of HMGB1 after weak shock wave exposure at 25 kPa in mouse brains. We first found that HMGB1 was translocated in neuronal cytoplasm in the entire brain from 6 to 24 hours after the exposure. It was re-localized into the nuclei 3 days later as in the control (non-blast exposed) mice. The time course of this changes is consistent with that of BBB disruption we previously reported. Our findings indicated that HMGB1 translocation occurred even after a weak shock wave exposure. Moreover, because the translocation occurred mainly in neurons, the blast-induced BBB disruption and inflammation can be triggered by neurons. (COI:No)

### PP-126

### Mn-MRI Method's Mn ion analysis of Alzheimer's Disease Model Mouse

Yuriko Inoue[1], Hiromithu Ezure[1], Hiroshi Moriyama[1], Jyunji Ito[2], Chika Sawa[3], Koichi Shiraishi[4], Harumi Hata[5], Masaaki Takayanagi[6], Takashi Takaki[7], Akiko Sasaki[8], Yoshinobu Manome[9], Akio Inoue[10], Naruhito Ohtsuka[1] (*[1]Dept. Anat.[II], Showa Univ. Sch. Med., [2]School of Nursing and Rehabilitation Sci., Dept. Nursing, Showa Univ., [3]Dept. Anat.[I], Showa Univ. Sch. Med. , [4]Medical Engineering Lab., Research Center for Medical Sci., The Jikei Univ. Sch. Med., [5]Center for Research and Development in Pharmacy Education, Nihon Univ., [6]Saitama Prefectural University, [7]Division of Electron microscopy, [8]Pharmacological Reserch Center, Dept. Pharmacology, Division of Medical Pharmacology Showa Univ., [9]Core Research Facilities for Basic Sci., Research Center for Med. Sci., The Jikei Univ. Sch. Med., [10]Human Brain Research Center,Graduate School of Med., Kyoto Univ*)

We studied the cause of Alzheimer's disease using transgenic mouse expressing a large amount of amyloid. The transgenic mouse shows the decrease of memory by the Y-maze test. However, no clear anatomical difference was observed between transgenic and control mice. As nerve cells uptake Mn ions depending on nerve activity, We injected Mn ions into the abdominal cavity of mouse and examined Mn in the brain using Bruker 9.4T MRI machine with cryoprobe. Magnetic resonance imaging (MRI), uses a strong magnetic field and radio waves to generate images of parts of the body. The contrast of image is produced by the difference of stability of activated state produced by radio wave. Nerve cells uptake Ca ions during neuronal excitement. Fluo4 complex is not fluorescent. Activated state of proton in free water is rather stable compared to that in lipid and proteins. As Mn ions destabilize the activated state, T1 intensity is activated by Mn ions. The transgenic mouse shows the decrease in Mn incorporation to the dentate gyrus of hippocampus and also the cerebrum including cingulate cortex, motor and sensory area. (COI:No)

### PP-127

### Neural mechanisms underlying anxiety and social memory impairment in Shank3-KO mice

Myung Chung[1,2], Katsutoshi Imanaka[1], Ziyan Huang[1], Akiyuki Watarai[1], Mu-Yun Wang[1], Kentaro Tao[1], Teruhiro Okuyama[1] (*[1]Laboratory of Behavioral Neuroscience, Institute for Quantitative Biosciences, The University of Tokyo, [2]Molecular Cell Biology, Graduate School of Medicine, The University of Tokyo*)

Autism spectrum disorder (ASD) is a heterogeneous neurodevelopmental disorder diagnosed by deficits in social communication along with highly restricted, repetitive behavior. In addition, patients with ASD often have other neuropsychological comorbid features such as anxiety and social memory impairment. Recent studies revealed a strong evidence of genetic contribution to ASD, particularly the genes that are essential for synapse formation and synaptic function. However, the common neural circuit(s) or region(s) which are responsible for the etiologies of both core and comorbid behavioral features observed in ASD patients remain elusive. Here we show that the elevated levels of observational fear and social memory impairment in *shank3* knockout (Shank3-KO) mice, one of the most promising genetic ASD models. *c-Fos* expression patterns after observational fear task were altered in multiple brain regions including the lateral hypothalamic area (LHA) and the nucleus accumbens (NAc) shell of Shank3-KO mice. (COI:No)

### PP-128

### Detection of a behavioral endpoint related to developmental methylmercury exposure independent of experimental environment in mice

Fumihiko Maekawa[1], Toshihiro Endo[2], Masaki Kakeyama[3] (*[1]Center for Health and Environmental Risk Research, National Institute for Environmental Studies, Japan, [2]Phenovance Research & Technology, LLC, [3]Waseda University School of Human Sciences*)

Epidemiological studies have shown that low concentrations of methylmercury in humans may induce neurophysiological effects. On the other hand, since there have been many empirical reports using animals to clarify the effects of low-level exposure of methylmercury, the reports have not been utilized as a basis for environmental standard values. We believe that the research using unified test method between organizations is the key to derive reliable toxic values. In this study, fetal mice were exposed to methylmercury, and behavioral abnormalities after being adults were analyzed in two organizations using identical analysis apparatus called IntelliCage. We identified that a reliable behavioral endpoint related to perseverative behavior is sensitive to developmental exposure to methymercury. We hope this trial will be the first step to create assessment endpoints useful for conducting toxicity test. (COI:No)

### PP-129

### Association of hippocampal insulin signaling with worsening cognitive impairment in Alzheimer's disease with type 2 diabetes

Wei Wang[1], Daisuke Tanokashira[1], Megumi Maruyama[1], Chiemi Kuroiwa[1], Hideaki Kurachi[1], Takashi Saito[2], Takaomi Saido[3], Akiko Taguchi[1] (*[1]Dept. of Integra. Aging Neurosci., [2]Dept. of Neurocognitive Sci., Nagoya City Univ., Graduate School of Medical Sciences., [3]Lab. for Proteolytic Neurosci*)

Type 2 diabetes mellitus (T2DM) is associated with an increased risk for dementia including Alzheimer's disease (AD). Our recent studies show that modification of insulin receptor substrates1 (IRS1) signaling is associated with cognitive decline in T2DM model mice and middle-aged APP knockin (APP KI) mice for new AD model. However, the effect of T2DM on APP KI mice and the changes of hippocampal IRSs signaling in T2DM-induced APPKI mice remain unknown. We found that T2DM exacerbates cognitive impairment in middle-aged APP KI mice but has no effect on cognitive function in young those mice without alteration in amyloid β (Aβ) accumulation in both young and middle age. Meanwhile, T2DM enhances modification of IRS1 signaling in the hippocampus of middle-aged APPKI mice, whereas unchanged modification of hippocampal IRS1 is observed in young those mice with T2DM. On the other hand, T2DM-induced glucose intolerance is suppressed in middle-aged APP KI mice although it occurs in a young age. These results suggest that the hippocampal IRS1 signaling may be involved in the development of AD with/without T2DM independently of Aβ accumulation and systemic glucose metabolism. (COI:No)

### PP-130

### Orexin And MCH Neuron-Ablated Mice Display Severe Sleep Attacks And Cataplexy

Chijung Hung[1,2,3], Daisuke Ono[1,2,3], Akihiro Yamanaka[1,2,3], Thomas S. Kilduff[4] (*[1]RIEM, Nagoya U, [2]Department of Neural Regulation, Nagoya University Graduate School of Medicine, [3]CREST, JST, Honcho Kawaguchi,, [4]Center for Neuroscience, Biosciences Division, SRI International*)

Orexin/hypocretin-producing and melanin-concentrating hormone-producing (MCH) neurons are co-extensive in the lateral hypothalamic area and project throughout the brain to regulate sleep/wakefulness. Ablation of orexin neurons in mice decreases wakefulness and results in a narcolepsy-like phenotype, whereas ablation of MCH neurons increases wakefulness. Since it is unclear how orexin and MCH neurons interact to regulate sleep/wakefulness, we generated conditional transgenic mice in which both orexin and MCH neurons could be ablated. Double-ablated mice exhibited increased wakefulness and decreased rapid eye movement (REM) and non-REM sleep. The total time in cataplexy and the mean cataplexy bout duration increased significantly in double-ablated mice compared with orexin neuron-ablated mice, suggesting that MCH neurons normally suppress cataplexy and that compromised MCH neurons may exacerbate symptoms in some narcoleptic patients. Double-ablated mice also showed frequent sleep attacks with elevated spectral power in the delta and theta range during wakefulness, a unique state with EEG characteristics indistinguishable from the transition from NREM into REM sleep. (COI:No)

### PP-131

### Visualized neuronal circuitry basis for decipher IL-17a-induced social recovery in ASD model mice

Midori Shibushita[1], Hiroaki Wake[1], Daisuke Kato[1], Ikuko Takeda[1], Ako Ikegami[1] (*[1]Department of Anatomy and Molecular Cell Biology Graduate School of Medicine*)

Autism Spectrum Disorder (ASD) is a neurodevelopmental disorder characterized by impaired social communication, limited interests, and repetitive behaviors. It has been clinically known the recovery of social behavior in ASD patient with fever in childhood, suggesting the contribution of immune reaction for ASD symptoms. A recent study showed the contribution of IL-17a for the recovery. However, the underlying mechanism of the neuronal active pattern change for IL-17a mediate recovery in sociability is unknown. In this study, using *in vivo* imaging we found that ASD model mice exhibited reduced neuronal circuit synchronization which ameliorated though intraventricular IL-17a infusion and neuronal synchronicity was correlated with social behavior. We further investigated the microglial contribution on neuronal synchronicity in ASD mice. Our results suggest that the production of IL-17a during inflammation affects the neuronal synchronicity via microglia in ASD model mice. These findings provide new insights into the mechanisms of neuronal circuit substrates underlying ASD-like behavior. (COI:No)

### PP-132

### The effect of chronic diazepam administration on hippocampal function.

Tomonori Furukawa[1], Yoshikazu Nikaido[2,3], Shuji Shimoyama[1,4], Shinya Ueno[1,4] (*[1]Dept of Neurophysiol, Grad. Sch. of Med, Hirosaki Univ., [2]Dept of Anesthesiology, Grad. Sch. of Med, Hirosaki Univ., [3]Dep of frailty research and prevention, Grad. Sch. of Med, Hirosaki Univ., [4]Research Center for Child Mental Development, Grad. Sch. of Med, Hirosaki Univ*)

Diazepam (DZP), a typical benzodiazepine drug, is widely prescribed for anxiety, epileptic discharge, insomnia, muscle-relaxing, and anti-convulsants. However, recent clinical studies are suggesting that chronic DZP (cDZP) treatment increases the risk of dementing disorder in the elderly. Whereas several studies reported the effect of cDZP administration on neuronal activity and cognitive performance, details of cDZP effect on cognitive function were not understood completely. In this study, to investigate the effect of cDZP administration, we performed Morris water maze test, spine density analysis and LTP assay in hippocampus of both young and aged mice. The memory performance was impaired by cDZP administration in aged but not in young mice. The spine density of hippocampal neuron was decreased by cDZP administration in CA3 of aged mice. LTP was attenuated by cDZP administration in CA3 of both young and aged mice. In addition, spine density and LTP were attenuated by aging. These results suggested that cDZP administration decrease LTP and dendritic spine density. The retrieval performance impairment of aged mice was likely to be attributed to both aging and cDZP administration. (COI:No)

### PP-133

### The synaptic change in early stage of Alzheimer's disease

Zhongtian Guo[1,2], Ako Ikegami[1], Daisuke Kato[1], Hiroaki Wake[1,2] (*[1]Nagoya University Graduate School of Medicine, [2]Kobe University Graduate School of Medicine*)

Alzheimer's disease (AD) is a neurodegenerative disorder that leads to progressive cognitive decline. Synapse loss occurs in AD before the cognitive decline. However, it is unclear when and how this spine loss occurs. To answer this question, we monitor simultaneously plaques with neurons in the AD model mouse by 2 photon microscope for 3 months. Synapse loss occur at 12 weeks old when only a few plaques, no memory loss can be detected.
To elucidate the temporal and spatial features of the synapse loss, we analyzed the spine formation, elimination rate, and distance of plastic synapses. The result shows that the closer spine gets tend to eliminate with the AD progress. We propose that the synapse loss occurs not randomly in early-stage AD but may have eliminate process within some segment. We hypothesis that the synapse loss may be associated with spine activity and their response. We used *in vivo* $Ca^{2+}$ imaging to monitor spine activity that result in synapse loss. We are further trying to manipulate spine activity to generate backpropagation by holographic stimulation to see the generation of action potential may involve in the process of synapse loss. (COI:No)

### PP-134

### Developmentally regulated KCC2 phosphorylation is critical for dynamic GABA-mediated inhibition

Miho Watanabe[1], Kahle Kristopher T[2], Atsuo Fukuda[1] (*[1]Dept Neurophysiol, Hamamatsu Univ. Sch. Med., Hamamatsu, Japan, [2]Departments of Neurosurgery, Pediatrics, and Cellular and Molecular Physiology, Centers for Mendelian Genomics, Yale School of Medicine*)

The $K^+/Cl^-$ cotransporter KCC2 is a molecular switch between excitatory and inhibitory effects of GABAergic inputs into neurons. Despite its importance for GABA inhibition, the regulatory mechanisms of KCC2 during maturation of the CNS are not understood. Here, we applied quantitative phosphoproteomics to systematically map sites of KCC2 phosphorylation during CNS development. KCC2 phosphorylation at Thr906 and Thr1007, which inhibits KCC2 activity, underwent dephosphorylation in parallel with the GABA excitatory-inhibitory sequence *in vivo*. Knockin mice expressing the homozygous phosphomimetic KCC2 mutations T906E/T1007E ($Kcc2^{E/E}$), which prevented the normal developmentally regulated dephosphorylation of these sites, exhibited early postnatal death from respiratory arrest and a marked absence of cervical spinal neuron respiratory discharges. $Kcc2^{E/E}$ mice also displayed disrupted lumbar spinal neuron locomotor rhythmogenesis and touch or pain-evoked status epilepticus associated with impaired KCC2-dependent $Cl^-$ extrusion. These data identify a previously unknown phosphorylation-dependent KCC2 regulatory mechanism that is essential for dynamic GABA-mediated inhibition. (COI:No)

### PP-135

### Adverse effects of amyloid β 1-42 oligomers: an irritant to current-induced spikes in hippocampal CA1 and cortical M1 pyramidal neurons

Hiroyuki Kida[1], Itsuki Kanehisa[2], Thein Oo Paw Min[1], Ryoichi Kimura[3], Dai Mitsushima[1] ([1]*Dept Physiol, Grad Sch Med, Yamaguchi Univ,* [2]*Med, Yamaguchi Univ,* [3]*Center for Liberal Arts and Sciences Sanyo-Onoda City Univ*)

Aggregation of amyloid β (Aβ) peptides is known as a cause of Alzheimer's disease. Especially Aβ42 oligomers are highly toxic, and a major component of senile plaques. In our model, bilateral microinjections of Aβ42 oligomers into the CA1 significantly impaired the performances in IA task. To analyze the effect of Aβ42 oligomers on intrinsic plasticity after training, we unilaterally microinjected the oligomers into the CA1. After one week, we subjected rats to the IA task to prepare acute brain slices for current clamp analysis. Although the training did not change the number of current-induced spikes in saline injected rats, microinjected Aβ42 oligomers significantly increased the number of spikes after the training.
Also, the bilateral M1 microinjections of Aβ42 oligomers significantly impaired the motor performances in rotor rod task. One week after microinjection, although the motor training slightly decreased the number of current-induced spikes in saline injected rats, microinjected Aβ42 oligomers significantly increased the number of spikes after the training. These results suggest novel adverse effects of Aβ42 oligomers in CA1 and M1 pyramidal neurons. (COI:No)

### PP-136

### Optical control and recording of neuronal activity and behaviour in *Caenorhabditis elegans* by yellow-light-activatable caged compound

Hironori Takahashi[1,2], Mako Kamiya[2], Minoru Kawatani[2], Keitaro Umezawa[2], Shinsuke Niwa[3], Toshiyuki Oda[1], Yasuteru Urano[2] ([1]*Dept Anat and Struct Biol, Grad Sch Med, Univ Yamanashi,* [2]*Grad Sch Med, Univ Tokyo,* [3]*FRIS, Tohoku Univ*)

*Caenorhabditis* (*C*) *elegans* is used as a model system to understand the neural basis of behaviour, but application of caged compounds to manipulate and monitor the neural activity is hampered by the innate photophobic response of the nematode to short-wavelength light or by the low temporal resolution of photocontrol. Here, we develop boron dipyrromethene (BODIPY)-derived caged compounds that release bioactive phenol derivatives upon illumination in the yellow wavelength range. We show that activation of the transient receptor potential vanilloid 1 (TRPV1) cation channel by spatially targeted optical uncaging of the TRPV1 agonist *N*-vanillylnonanamide at 580 nm modulates neural activity. Our caged TRPV1 agonist has the sharp absorption spectrum that is orthogonal to that of GCaMP, enabling imaging of GCaMP to be done simultaneously without concomitant undesired photo-uncaging. In this study, we achieved simultaneous optical control and monitoring of activity in sensory and motor neurons, interneurons and muscles using our caged compound and GCaMP6s. Our caged compounds will serve as a useful tool for neuroscientific research. (COI:No)

### PP-137

### FT-GO: a Fluorescent Tyramide Signal Amplification System for Cytochemical and Histochemical Analysis

Kenta Yamauchi[1,2], Shinichiro Okamoto[1,2], Takahiro Furuta[3], Masato Koike[1], Hiroyuki Hioki[1] ([1]*Dept. Cell Biol. and Neurosci., Juntendo Univ. Grad. Sch. Med.,* [2]*Adv. Res. Inst. Health Sci., Juntendo Univ.,* [3]*Department of Oral Anat and Neurobiol., Grad. Sch. Dent., Osaka Univ*)

Tyramide signal amplification (TSA), also called catalyzed reporter deposition (CARD), is a highly sensitive method that enables the detection of low-abundance targets in immunocytochemistry, immunohistochemistry (IHC) and *in situ* hybridization. In a previous study, we reported a simple and cost-effective TSA system, BT-GO, for bright-filed imaging. Here, we developed FT-GO (Fluorescent Tyramide-Glucose Oxidase) as a fluorescent TSA system. FT-GO involves horseradish peroxidase-catalyzed deposition of fluorescent tyramide with hydrogen peroxide produced during the oxidation of glucose by glucose oxidase, as BT-GO. FT-GO markedly enhanced IHC signals while maintaining low background signals. We applied the FT-GO system to IHC for tyrosine hydroxylase and serotonin transporter. FT-GO demonstrated a more widespread distribution of catecholaminergic and serotonergic axons in the mouse brain, compared with conventional indirect detection methods. Given its simplicity, cost-effectiveness and a staining with a high signal-to-noise ratio, our FT-GO system would provide a versatile and scalable platform for cytochemical and histochemical analysis. (COI:No)

### Poster Presentations

### Day2

(March 29, Mon. 18 : 30～19 : 30)

| | |
|---|---|
| **PP-138～PP-145** | Molecular anatomy, Molecular physiology, Cell biology, Histology : Oraganelle, Membrane transport |
| **PP-146～PP-161** | Molecular anatomy, Molecular physiology, Cell biology, Histology : Molecular anatomy, Molecular physiology |
| **PP-162～PP-171** | Molecular anatomy, Molecular physiology, Cell biology, Histology : Histology |
| **PP-172～PP-178** | Molecular anatomy, Molecular physiology, Cell biology, Histology : Others |
| **PP-179～PP-212** | Ion channels, Receptors |
| **PP-213～PP-241** | Embryology, Regenerative Medicine, Development, Growth, Aging |
| **PP-242～PP-259** | Cartilage, Bone, Connective tissue |
| **PP-260～PP-273** | Muscle |
| **PP-274～PP-287** | Digestion, Digestive system, Oral physiology : Digestion, Digestive system |
| **PP-288～PP-296** | Digestion, Digestive system, Oral physiology : Oral physiology and anatomy, Tooth, Salivary gland |

### PP-138

### Oxysterol-binding protein (OSBP)-related protein (ORP) 6 is involved in the turnover of phosphatidylinositol 4-phosphate(PI4P) and phosphatidylserine (PS) in neurons.

Shinya Mochizuki[1], Harukata Miki[1], Ruyun Zhou[1], Yasuko Noda[1] ([1]*Univ. Jichi. Med. Dept. Anatomy*)

Oxysterol-binding protein-related protein (ORP) form a conservative protein family that are present in organisms ranging from yeast to mammalian. ORPs are lipid binding protein that share a conserved OSBP-related ligand binding domain (ORD) in the C-terminal region. Increasing evidence indicates that they are localized at membrane contact site (MCS) of two different membrane organelles. ORP6, are member of ORP family Ⅲ , is one of the least examined molecules because of the lower yield by exogenous expression.
I had previously reported that ORP6 is expressed in the brain and localizes at ER and ER-plasma membrane MCS in cultured cerebellar neuron. I also identified PI4P, PI(4,5)$P_2$, PI(3,4,5)$P_3$ and phosphatidic acid as binding lipid of the PH domain of ORP6. I showed the involvement of ORP6 in the turnover of PI4P by using RNAi.
This time, I have further examined the knockdown effect of ORP6 on the neuron-derived cells and analyzed the mechanism of the lipid transport of ORP6 between organelles. Localization of Phosphatidylserine (PS) marker was changed by knockdown of ORP6 and inhibition of PI4P synthesis in plasma membrane. (COI:Properly Declared)

### PP-139

### Mitochondrial morphological analysis in PARK2 iPSC-derived dopaminergic neurons under oxidative stress

Mutsumi Yokota[1], Soichiro Kakuta[2], Yutaro Yoshino[1], Takahiro Shiga[3], Kei-ichi Ishikawa[3,4], Hideyuki Okano[5], Nobutaka Hattori[4], Wado Akamatsu[3], Masato Koike[1] ([1]*Department of Cell Biology and Neuroscience, Juntendo University Graduate School of Medicine,* [2]*Laboratory of Morphology and Image Analysis, Research Support Center, Juntendo University Graduate School of Medicine,* [3]*Center for Genomic and Regenerative Medicine, Juntendo University Graduate School of Medicine,* [4]*Department of Neurology, Juntendo University School of Medicine,* [5]*Department of Physiology, Keio University School of Medicine*)

Parkinson's disease (PD) with *PARK2* mutations features the loss of dopaminergic neurons in the substantia nigra pars compacta, which has been suggested to result from the accumulation of damaged mitochondria. In recent studies, spherical mitochondria have been observed under oxidative stress and considered as the morphology for the degradation of uncoupled mitochondria. However, it is unclear whether the deficiency in the formation of spherical mitochondria is involved in the pathogenesis of PD. In this study, we used our newly established PARK2 tyrosine hydroxylase reporter iPSC lines and analyzed mitochondrial morphology with correlative light and electron microscopy. We found that the formation of spherical mitochondria, which was induced in control dopaminergic neurons by a mitochondrial uncoupler CCCP, was inhibited in PARK2 dopaminergic neurons. We observed elevated oxidative stress in PARK2 dopaminergic neurons under the CCCP treatment, possibly indicating that the accumulation of damaged mitochondria by the insufficient formation of spherical mitochondria. Our study would provide insights into the processes leading to the cell death of PARK2 dopaminergic neurons. (COI:No)

### PP-140

### Endosome-localized clathrin adaptors AP-1 and GGA2 regulate cell surface expression of EGFR for cell growth

Takefumi Uemura[1], Satoshi Waguri[1] ([1]*Fukushima Med Univ*)

The role of Golgi/endosome-localized clathrin adaptors in the maintenance of steady-state cell surface receptor tyrosine kinases (RTKs) is not well known. Here, we report that both adaptor protein complex 1 (AP-1) and Golgi-localized, γ-adaptin ear-containing, ADP ribosylation factor-binding protein 2 (GGA2) are involved in regulating expression of the epidermal growth factor receptor (EGFR), an RTK. AP-1 depletion reduced the expression of EGFR protein by promoting its lysosomal degradation. Proximity ligation assays demonstrated that the interaction of EGFR with AP-1 or GGA2 occurs in Rab11-positive recycling endosomes. AP-1 depletion suppressed the growth of H1975 cancer cells and normal ARPE-19 cells. AP-1 was expressed in endosomes at high levels in several cancer tissues. Collectively, these results indicate that AP-1 and GGA2 function to retrieve EGFR in recycling endosomes, thereby sustaining its cell surface expression and, consequently, cancer cell growth. (COI:Properly Declared)

### PP-141

### Characterization of Protein N-Glycosylation in Golgi pH regulator deficient cells.

Yu-shin Sou[1], Yusuke Maeda[2], Taroh Kinoshita[2], Masato Koike[1] ([1]*Department of Cell Biology and Neuroscience, Juntendo University Graduate School of Medicine,* [2]*Research Institute for Microbial Diseases, Osaka University*)

The Golgi apparatus plays an indispensable role in the post-translational modification and transport of proteins to their target destinations. The lumen of the Golgi apparatus is regulated to be acidic pH, which is known to be critical for protein glycosylation and transport. The Golgi pH regulator (GPHR) is an anion channel essential for acidification of the Golgi lumen. To better understand why luminal acidic condition of the Golgi apparatus is important for protein N-glycosylation, we have examined here with GPHR knock-out cell lines. We first analyzed the abnormality of protein N-glycosylation in GPHR-deficient cells by MOLDI-TOF mass analysis. In addition, we showed that altered glycosylation of the lysosomal membrane protein Lamp1 and the Golgi membrane protein GPP130 by western blot analysis. By using morphological approach, we also show that abnormal localization of the glycosyltransferase due to impaired retrograde transport pathway from endosome to the Golgi in GPHR knock-out cells. These results suggest that neutralization of the Golgi lumen may change protein N-glycosylation by inducing glycosyltransferase mislocalization. (COI:No)

### PP-142

### lipid droplets dynamics in the nucleus

Yuki Ohsaki[1], Kamil Soltysik[2], Jinglei Cheng[1], Toyoshi Fujimoto[3] ([1]*Anat. Mol. Cell Biol., Grad. Sch. Med., Nagoya Univ.,* [2]*Biochem. Mol. Biol., Grad. Sch. Med., Tokyo Univ.,* [3]*Inst. Dis. Old Age, Juntendo Univ*)

Lipid droplets (LD) are cytoplasmic organelle composed of neutral lipids and a phospholipid monolayer, but also exist in the nucleus in specific types of cells. So far we have identified two biogenesis pathways of nuclear LDs. In hepatic cells, nuclear LDs are derived from VLDL precursor particles, which are first formed in the ER lumen and accumulated in the inward extension the inner nuclear membrane (INM), and become nucleoplasmic LDs. On the other hand in non-hepatic cells, LDs are directly formed in the INM where lipid synthesizing enzymes and lipid precursors are distributed. We also reported nuclear LDs surface can activate phosphatidylcholine synthesis, but other dynamics remains unknown.
In this study we searched molecules which can regulate the size and number of nuclear LDs. By FRAP assay, we found that nuclear LDs increase their size in G1 phase due to lipids transfer either in the INM or between LDs, and the latter can be controlled by Cideb and perilipin-3, which could be a counterpart of cytoplasmic LDs fusion machinery; Cidec and perilipin-1. Now we are investigating other molecules to affect LD dynamics in the nucleus. (COI:No)

### PP-143

### Identification of BICC1-ANKS3-ANKS6 complexes ciliary localization

Yoshiro Nakajima[1], Kazuhiko Matsuo[1] ([1]*Department of Anatomy and Developmental Biology Kyoto Prefectural University of Medicene*)

The primary cilium is an antenna-like structure protruding from most mammalian cells. This organelle is now considered to have important roles in development and tissue homeostasis. Lack of cilia or ciliary proteins results in many cilia-related phenotypes, such as *situs inversus* and renal cystic disease and so on. The base of the primary cilium is the basal body, a modified structure of the mother centriole. The ciliary rootlet, a cytoskeleton-like structure, originates from the centriole.
Ankyrin repeat and SAM domain-containing protein 6 (ANKS6) are unnecessary for ciliogenesis, nonsense mutations or genetic knockouts result in severe multiorgan malformation syndromes including left–right asymmetry perturbations and cystic kidneys. ANKS6accumulates in the Inv compartment, proximal segment of ciliary body. In recent years, ANKS6 has been found to bind to ANKS3 and the RNA-binding protein BICC1. However, it is unclear whether ANKS3 and BICC1 are localized to the primary cilia. In this report, ciliary localization of BICC1-ANKS3-ANKS6 complexes was investigated by super resolution microscopy. (COI:No)

### PP-144

### Contributions of NCLX and NCX to mitochondrial $Na^+$-$Ca^{2+}$ exchange in mouse brain

Ayako Takeuchi[1,2], Satoshi Matsuoka[1,2] ([1]*Integr. Physiol. Fac. Med. Sci. Univ. Fukui,* [2]*Life. Innov. Univ. Fukui*)

We reported that NCLX functions as a mitochondrial $Na^+$-$Ca^{2+}$ exchanger and participates in regulating various cellular functions such as automaticity of HL-1 cardiomyocytes, and $Ca^{2+}$ signaling and chemotaxis in B lymphocytes. Previously, mitochondrial localization of plasma membrane $Na^+$-$Ca^{2+}$ exchangers (NCX) 1-3 were reported in heart and brain. However, functional contributions of NCLX and NCXs to overall mitochondrial $Na^+$-$Ca^{2+}$ exchange activity are still unknown. We studied these issues in mouse isolated brain mitochondria.
Application of Ru360, a mitochondrial $Ca^{2+}$ uniporter blocker, to $Ca^{2+}$ loaded mitochondria resulted in extra-mitochondrial $Ca^{2+}$ increase in the presence of $Na^+$. This $Na^+$-dependent $Ca^{2+}$ efflux was significantly inhibited by a NCLX blocker CGP-37157, but was insensitive to NCX blockers SEA0400 and SN-6. In the presence of Ru360, intra-mitochondrial $Ca^{2+}$ rapidly increased upon $Ca^{2+}$ application when preloaded with $Na^+$. This intra-mitochondrial $Na^+$-dependent $Ca^{2+}$ influx was insensitive to both NCLX and NCX blockers. Taken together, it was revealed that NCLX plays a major part in forward mode of mitochondrial $Na^+$-$Ca^{2+}$ exchange, and contribution of NCX is minor. (COI:No)

### PP-145

### Single molecule analysis of insulin granule movement within pancreatic β-cells

Hiroyasu Hatakeyama[1], Tomomi Oshima[1], Noriko Takahashi[1] ([1]*Department of Physiology, Kitasato University School of Medicine, Japan*)

Insulin secretion from pancreatic β-cells is critical for glucose homeostasis. We have previously suggested importance of insulin granule delivery to the plasma membrane by imaging analysis of insulin exocytosis with two-photon excitation microscopy. However, the behavior of insulin granule occurring deeper within the cells remains unclear due to the technical limitations. To quantify intracellular movement of insulin granule with high positional/temporal precision, we here developed a novel method based on single molecule imaging with Quantum dot (QD) fluorescent nanocrystals. We used HaloTag technology and electroporation to label insulin granule membrane proteins (e.g., phogrin and zinc transporter ZnT8) with QD in rat INS-1 cells. Localization of the exogenously expressed HaloTag-fusion proteins was similar to endogenous insulin. We successfully labeled the HaloTag-fusion proteins with QD and found dynamic movement of the labeled QDs. Both microtubules and F-actin were involved in the dynamic movement, but the regulation was different from each other. Our method is a valuable approach for investigating dynamic cellular processes of insulin granule delivery. (COI:No)

### PP-146

### FABP7 is involved in cell proliferation of wild-type IDH1 glioma.

Yoshiteru Kagawa[1], Banlanjo Abdulaziz Umaru[1], Yuji Owada[1] ([1]*Grad. Sch. Med., Tohoku Univ. Dept. Organ Anatomy*)

Gliomas are tumors that arise from glial or glial progenitor cells in the central nervous system. Currently, the World Health Organization (WHO) classifies them into three malignancy grades based on morphology and cell type. In addition, isocitrate dehydrogenase (IDH) mutations are used as prognostic biomarkers considering survival rate. It has been reported that FABP7, an intracellular fatty acids chaperon, is highly expressed in glioblastoma (previous WHO grade IV) and its expression level is correlated with malignancy and survival in glioma patients. However, FABP7 expression in different glioma grades using current classification and the role of FABP7 in tumor activity are still unknown. In this study, using human samples, we revealed that FABP7 highly expressed in the nucleus of IDH1wt glioblastoma, while IDH1mut expressed a lower level of FABP7 without expression in nucleus. NLS-tagged FABP7 increased nuclear acetyl-CoA and H3K27ac levels, but not observed with NES-tagged FABP7. These findings suggest that IDH1 mutation affects FABP7 expression and nuclear FABP7 is involved in the epigenetic regulation through controlling nuclear acetyl-CoA levels in IDH1wt glioma. (COI:No)

### PP-147

### Effects of electrical stimulation on signal transduction-related proteins in fibroblasts

Kazuo Katoh[1] ([1]*Department of Health Sciences, Tsukuba University of Technology*)

In the field of acupuncture, electrical stimulation of the skin and muscles is known to locally increase blood flow and metabolism, and maintain the body in a sustained healthy state. However, little is known about the changes in cellular morphology and the localization of specific proteins associated with electrical stimulation. In this study, we analyzed the effects of electrical stimulation on the cytoskeletal system in fibroblasts.
When electrical stimulation was applied to cells for about 1 hour, the stress fibers (SFs) in the cells became thicker and the cells showed contraction. When the cells were subjected to periodic electric current for 20 hours, the SFs increased in thickness. In addition, the focal adhesion (FA) increased after 2 hours of continuous stimulation, and both the SFs and the FAs became thicker and larger after 20 hours of continuous stimulation. Staining of the cells after electrical stimulation with anti-phosphotyrosine antibody showed enhanced staining in FAs. The intensities of staining for focal adhesion kinase (FAK) and activated c-Src were also enhanced, indicating that signal transduction-related proteins were affected by electrical stimulation. (COI:No)

### PP-148

### Molecular morphological research of actin assembled structure with arachidonic acid

Hideyuki Tanaka[1], Tahashi Nakakura[1], Kenjiro Arisawa[1], Youko Nekooki[1], Toshio Miyashita[1], Takutoshi Inoue[1], Haruo Hagiwara[1] ([1]*Grad,Sch.Med., Teikyo*)

Podosome is a membrane protrusion, of which intracellular structure is actin-filaments as a core . Vascular smooth muscle cell (VSMC) in the culture dose not little, if any, form the podosome, but it includes the protrusion by adding arachidonic acid. The actin cores are associated with myosin light-chain kinase (MLCK) are known for VSMC, among them, involvement in forming protrusion of myosin Ⅱ, the most important actin binding protein to be clarified. First, we tried the pharmacological approach by the use of the fluorescent microscope. Arachidonic acid ($10^{-6}$M, final concentration ) was applied to the medium culturing A7r5 cells as a typical cell line of VSMC. After incubation for specified periods, the culture plates were subjected to the count how many cells formed podosome. The numbers of VSMC with podosome were increased with the elapse of time. The VSMCs with podosomes were maximal within 30 min, 90 % cells developed podosomes. Actin, myosin and MLCK had been detected triplestaning by anti-actin and anti-myosin, anti-MLCK with arachidonic acid. The observation above regarding the role of myosin and MLCK in podosome suggests that is essential in podosome formation. (COI:No)

### PP-149

### Fatty acid binding protein 5: a potential regulator of macrophage phagocytosis in murine Peyer's patch.

Ryoji Suzuki[1], Yuji Owada[2], Yoshio Bando[1] ([1]*Grad.Sch.Med., Akita Univ., Dept. Anat.,* [2]*Grad.Sch.Med., Tohoku Univ., Dept. Organ Anat*)

Fatty acid binding proteins (FABPs) belong to the group of conserved multigene lipid binding protein family. We previously reported FABP5 localization of germinal center macrophages in Peyer's patches of C57BL/6 mice and FABP5 positive processes of macrophages protruding on surrounding phosphatidylserine (PS) immunopositive cell surface. Since PS on cell surfaces works as eat-me-signal, these findings implied FABP5 association of macrophage phagocytosis. In terms of AnnexinV, known to fix PS on cell surface, our study also revealed positive correlation of FABP5 expression with AnnexinV secretion in vitro. In this study, FABP5 association of macrophage phagocytosis was further investigated.
In vitro study showed FABP5 association to phagocytosis was confirmed by FABP5 enhancement of PS coated microsphere engulfment. PS coated or plain 1 micron microspheres were added in RAW264.7 cell culture. Twelve hours later, FABP5 overexpressed cells contained significantly more PS coated microsphere than control cells, while no difference was observed in plain microsphere added group.
These findings support positive correlation of FABP5 expression on macrophage with phagocytosis. (COI:No)

### PP-150

### Effect of extracellular nucleic acid on vascular endothelial cells

Chika Sawa[1], Mariko Gunji[1], Yuriko Inoue[1], Kazuho Honda[1] ([1]*Dept. of Anatomy Showa Univ. School of Med*)

It has been paid attention to the function of nucleic acids released by necrosis and apoptosis of inflammatory cells and neutrophils recently. We have previously shown that extracellular nucleic acids, especially dATP act on macrophage-like cells (PMA-induced U937) to induce the expression of several factors such as IL-8 and THBS-1 (Thrombospondin-1), thereby activating macrophages in vitro. As reported, IL-8 induces chemotaxis of neutrophils and NK-T cells and plays an important role in the initial immune response, and THBS-1 induces angiogenesis suppression and oxidative stress. Therefore, in this study, we investigated the effect of this phenomenon on the proliferation and angiogenesis and morphology of nearby vascular endothelial cells by using HUVEC. (COI:No)

### PP-151

### Actin filaments radially extend in association with microtubule asters during mitosis of starfish embryos

Ayana Sugizaki[1], Keisuke Sato[1], Kazuyoshi Chiba[2], Masahiko Kawagishi[1], Sumio Terada[1] ([1]*Department of Neuroanatomy and Cellular Neurobiology, Graduate School of Medical and Dental Sciences, Tokyo Medical and Dental University,* [2]*Department of Biological Sciences, Ochanomizu University*)

Fluorescence polarization microscope (FPM) can detect not only the localization but also the orientations of fluorescently labeled molecules thus is useful for studying biomolecular assemblies in live cells, but the lack of general labeling methods has hindered its application. Recently we developed POLArIS (Probe for Orientation and Localization of Arbitrary Intracellular Structures) and applied the method to the observation of actin dynamics in living starfish eggs and embryos. Starfish oocytes were microinjected with mRNA expressing $POLArIS^{act}$, which is the first example of POLArIS probe that specifically binds to F-actin. Through our FPM observation, we found that actin filaments radially extend from centrosomes during mitosis in early embryos. We named this actin-based structure FLARE (FLuffy And Radial actin-aster associated with mitosis in Embryo). Live-cell observation of FLARE with microtubules revealed that both the dynamics and distribution of FLARE are very similar to those of the microtubule aster. Pharmacological studies indicated that the microtubule aster is required for the FLARE formation and its maintenance. (COI:No)

### PP-152

### Mouse transmembrane channel-like protein 1 (mTMC1) can be translated from a splice variant *mTmc1ex1* but the translation from the other variant *mTmc1ex2* is negligible due to its 5' untranslated region

Soichiro Yamaguchi[1], Maho Hamamura[2], Kenichi Otsuguro[2] ([1]*Laboratory of Physiology, Department of Basic Veterinary Sciences, Faculty of Veterinary Medicine, Hokkaido University, Japan,* [2]*Laboratory of Pharmaco, Department of Basic Veterinary Sciences, Faculty of Veterinary Medicine, Hokkaido University, Japan*)

Transmembrane channel-like protein 1 (TMC1) has been revealed to be the pore-forming subunit of the mechanoelectrical transduction (MET) channel in the inner ear. It was reported that the two splice variants for mouse Tmc1 (*mTmc1ex1* and *mTmc1ex2*) were expressed in the cochlea of infant mice. However, we questioned whether mTMC1 was translated from *mTmc1ex2* due to the following two facts. Firstly, there is an upstream open reading frame (uORF) in the 5' untranslated region (UTR) of *mTmc1ex2*. Secondly, *mTmc1ex2* lacks a typical Kozak sequence. Therefore, we examined from which splice variant mTMC1 protein is translated. Firstly, the results of RT-PCR and cDNA cloning of *mTmc1* suggested that more *mTmc1ex1* were expressed than *mTmc1ex2* in the cochlea of five-week-old mice. Secondly, in a heterologous expression system, mTMC1 was translated from mTmc1ex1 but the translation from mTmc1ex2 was negligible. Finally, analyses using site-directed mutagenesis revealed that the uORF and the weak Kozak sequence in *mTmc1ex2* caused the negligible translation of mTMC1 from *mTmc1ex2*. These results suggest that *mTmc1ex1* is the protein-coding mRNA for mTMC1. (COI:No)

### PP-153

### Luminal calcium dynamics in the endoplasmic reticulum during calcium oscillations in mouse eggs analyzed using a fluorescent probe with improved subcellular localization

Takashi Kikuchi[1], Takumi Yokoyama[1], Hideki Shirakawa[1] ([1]*Department of Engineering Science, The University of Electro-Communications*)

Fertilizing sperm activate mammalian eggs by inducing repetitive increases in cytosolic $Ca^{2+}$ concentration ($[Ca^{2+}]_{cyt}$), or $Ca^{2+}$ oscillations, each of which is due to $Ca^{2+}$ release from the endoplasmic reticulum (ER). The regulation of $Ca^{2+}$ concentration in the ER ($[Ca^{2+}]_{ER}$) is important to generate a correct pattern of oscillations required for early embryonic development. We investigated the changes in $[Ca^{2+}]_{ER}$ during $Ca^{2+}$ oscillations in mouse eggs, using a FRET probe with improved performance. Dynamic range of FRET signals was increased by replacing FRET acceptor in standard D1ER probe with circularly permuated Venus (cpV), and intracellular localization of probe proteins became more restricted to the ER by adding the destabilizing domain to D1ERcpV. Simultaneous measurement of $[Ca^{2+}]_{cyt}$ and $[Ca^{2+}]_{ER}$ revealed that the recovery rate of $[Ca^{2+}]_{ER}$ after $Ca^{2+}$ release is dependent on $Ca^{2+}$ entry from outside. Interestingly, the ER was refilled with $Ca^{2+}$, accompanied by no decrease in $[Ca^{2+}]_{cyt}$ even in the absence of extracellular $Ca^{2+}$. The results suggest the involvement of additional intracellular stores to temporally sequester $Ca^{2+}$ in the cycles of $Ca^{2+}$ oscillations. (COI:No)

### PP-154

### Volume-regulated Anion Channels (VRACs) Contribute to Hypo-osmotic Stress Induced ATP Release in Undifferentiated Mammary Cells

Kishio Furuya[1], Yuko Takahashi[1], Hiroaki Hirata[1], Takeshi Kobayashi[2], Masahiro Sokabe[1] ([1]*Mechanobiology Lobo, Nagoya Univ Grad Sch Med,* [2]*Dept Physiol, Nagoya Univ Grad Sch Med*)

The high interstitial ATP concentration in the cancer microenvironment is a major source of adenosine which acts as a strong immune suppressor. However, the source of ATP, which must be continuously released from cancer cells, has not yet been elucidated. Using a real time ATP luminescence imaging system, we found that hypotonic stress induced two patterns of ATP release, a slowly rising diffuse pattern in undifferentiated breast cell lines and a transient sharp pattern in primary cultured differentiated cells. Cholera toxin treatment changed the pattern from slow diffuse to transient sharp in breast cell lines. TGF$\beta$ treatment changed the pattern vice versa in primary cultured cells. DCPIB, an inhibitor of VRACs, only suppressed the diffuse pattern. The inflammatory mediator sphingosine-1-phosphate (S1P) induced diffuse ATP release isovolumetrically. Knockdown of A isoform of leucine-rich repeat-containing protein 8 (LRRC8A), an essential molecular entity of VRACs, using shRNA suppressed the diffuse ATP release induced by both hypotonic stress and S1P application. These results suggest that a subfamily of VRACs is a conduit of ATP in undifferentiated cells including cancer cell. (COI:No)

### PP-155

### Identification of genes regulating sensory functions via modifications of membrane lipids in *Drosophila*.

Takuto Suito[1], Takaaki Sokabe[1], Makoto Tominaga[1] ([1]*Div Cell Signaling, Natl Inst Physiol Sci*)

Sensory function is biologically essential for collecting the information from external environments. An increasing number of sensory receptor proteins such as TRP channels and their roles have been reported, however, other regulatory components in the sensory function remain to be elucidated.

In this study, we focused on the lipid molecules as the regulator of sensory functions. Firstly, we sought the functional genes which are involved in membrane lipid metabolism in peripheral sensory neurons in *Drosophila*. We performed the transcriptome analysis in isolated Class III and Class IV multidendritic neurons which participate in thermo- and mechano-sensations. We observed the enriched expressions of several genes for phospholipid synthesis, fatty acid synthesis, and other lipid metabolisms in those sensory neurons compared with the whole body. Next, to assess the roles of lipid regulatory genes in thermo- and mechano-sensations, we performed the RNAi screening for temperature preference and nociceptive responses to extreme temperature and mechanical stimuli. We will discuss the novel regulatory mechanism of sensory functions via modifications of membrane lipids. (COI:No)

### PP-156

### Analysis of the heat stress response mechanism in HT22 cells

Megumi Kato[1], Yuga Hiraoka[1], Hideo Kawaguchi[1] ([1]*Graduate School of Life Sciences, Toyo University*)

Heat stress can activate molecular chaperones such as HSP70 that refold denatured proteins. Although such stress can cause cerebral dysfunction, the impact of heat stroke on brain function remains poorly understood. The aim of this study is to analyze the heat and endoplasmic reticulum stress responses of HT22 cells derived from mouse hippocampus and to examine the role of high temperature pretreatment in adaptive cytoprotection. After culturing HT22 cells at 43℃ for 24 hours, mRNA expression levels of various genes were analyzed by real time RT-PCR. Results showed that heat treatments increased mRNA levels of both *HSP70* and endoplasmic reticulum localized *eIF2α* and *IRE1α*, indicating that heat stress may cause the endoplasmic reticulum stress. After pretreatment of cells at 41℃ for 6 hours followed by culturing at 43℃ for 24 hours, the viable cell rate measured using flow cytometry increased. This viability increase indicates that adaptive cytoprotection may modulate the endoplasmic reticulum response to create adaptive cytoprotection in the brain. (COI:No)

### PP-157

### Alkaline stimuli increased intracellular cAMP levels in odontoblasts

Eri Kitayama[1,2], Maki Kimura[2], Hiroyuki Mochizuki[2], Kyosuke Kouno[2], Masayuki Ando[2], Sadao Oyama[2], Masahiro Furukawa[1], Yoshiyuki Shibukawa[2] ([1]*Tokyo Dental College Department of Endodontics,* [2]*Tokyo Dental College Department of Physiology*)

Alkali-induced $Ca^{2+}$ signaling in odontoblasts participates in dentinogenesis under high pH conditions. Previously, we clarified cAMP mediated $Ca^{2+}$ signaling in odontoblasts. These reports suggest that cAMP might play critical roles in dentinogenesis following stimuli to dentin surface. However, both the alkali-induced intracellular cAMP signaling pathway and the role of cAMP on the cellular function during dentinogenesis evoked by extracellular high pH environment in odontoblasts are unclear. In this study, we examined the effect of extracellular high pH environment on intracellular cAMP levels in human odontoblast (HOB) cells. We measured intracellular cAMP levels using an mNeon Green-based cAMP sensor. In the presence of extracellular $Ca^{2+}$, application of alkaline solution (pH 8-10) increased intracellular cAMP level in HOB cells. The increases were inhibited by an adenylyl cyclase inhibitor. The increases also showed pH dependence ranging from pH 8 to 10. Repeated application of alkaline solution enhanced increase in alkali-induced intracellular cAMP level. These results suggest that alkaline stimuli increased intracellular cAMP levels in a pH-dependent manner in odontoblasts. (COI:No)

### PP-158

### The role of HSPs in the acquisition of adaptive heat tolerance by cells

Yuga Hiraoka[1] ([1]*Graduate School of Life Sciences, Toyo University*)

Heatstroke requires countermeasures, because heatstroke can damage the brain and cause brain dysfunctions, such as impaired consciousness, in severe cases. We focused on HSP70, which has the function to refold proteins denatured by stresses such as heat. In preliminary experiments, we have shown that HT22 cells, which are derived from the mouse brain hippocampus, can be cultured for long periods of time even at higher-than-usual culture temperatures after the cells were treated with weak heat stress. The present study aimed to confirm if this phenomenon of adaptive cytoprotection was dependent on HSP70. After the addition of the HSP70-specific inhibitor Pifithrin-μ or siRNA of HSP70, HT22 cells were pre-treated with weak heat stress at 41℃ for 6 h. The pre-treated cells were then cultured at 43℃ for 24 h, and the cell viability was measured using flow cytometry. As a result, we found that both the inhibitor and siRNA significantly reduced cell viability. Our findings suggest that the phenomenon of adaptive cytoprotection in HT22 cells depends on HSP70. (COI:No)

### PP-159

### Dysfunction of $Cl^-$ channel promotes epithelial to mesenchymal transition in oral squamous cell carcinoma via activation of Wnt/β-catenin signaling pathway

Akihiro Hazama[2], Kei Kakinouchi[1,2], Shigeyuki Nurono[2], Susumu Yoshie[1] ([1]*Department of Cellular and Integrative Physiology, Fukushima Medical University, Fukushima, Japan,* [2]*Department otolaryngology, Fukushima Medical University, Fukushima, Japan*)

[Background] Oral squamous cell carcinoma (OSCC) is a highly aggressive carcinoma with a high incidence of recurrence and distant metastasis. However, the mechanism of epithelial to mesenchymal transition (EMT) during tumor progression and metastasis in OSCC has not yet been fully elucidated. The aim of the present study is to investigate the role of the $Cl^-$ channel on EMT in the OSCC.
[Methods] OSC-20 cells, which is an OSCC line, were cultured with low serum medium containing the $Cl^-$ channel blocker NPPB. Morphological change, gene expression, and signaling pathway of the NPPB-treated OSC-20 cells were evaluated.
[Results] The morphology of the NPPB-treated OSC-20 cells showed typical morphology of mesenchymal cells. The expression levels of the epithelial marker E-cadherin in the NPPB-treated OSC-20 cells were decreased. Those of mesenchymal markers vimentin and snail were increased. The amount of β-catenin protein was increased in the NPPB-treated OSC-20 cells.
[Conclusions] Dysfunction of $Cl^-$ channel promoted EMT via activation of the Wnt/β-catenin signaling pathway in OSCC. (COI:No)

### PP-160

### Cesium perturbs cytoskeletal actin elongation in NIH/3T3 cells and suppresses cell proliferation

Daisuke Kobayashi[1], Natsumi Nishimura[1], Khatun Ziasmin[1], Akihiro Hazama[1] ([1]*Department of Cellular and Integrative Physiology, Fukushima Medical University, Japan*)

We previously showed cesium inhibited the growth of the human cancer cells by inhibition of the glycolytic pathway. Herein, we investigated the effects of Cs on murine fibroblast cells (NIH/3T3) proliferation and migration. The treatment of Cs inhibited the migration of fibroblast cells compared with the control and the inhibition showed a dose-dependent manner. The number of the cells was decreased a dose-dependent manner, whereas the viability of cells remained almost unchanged. Regarding microscopic observation, a shape of migrating cells seemed to be different between in the absence and in the presence of Cs. We assumed that the morphologic difference was caused by cytoskeleton difference. A structure of cytoskeletal actin fiber was visualized with AlexaFluor568-conjugated phalloidin. The cells treated with Cs tend to round shape, and its actin fibers were condensed at peripheral membrane ruffle compared with control. Moreover, there were structures look like slender fingers of membrane, as well as filopodia in Cs application cells. These results indicated that perturbation of cytoskeletal actin fiber elongation was caused by Cs, and then cell proliferation was inhibited. (COI:No)

### PP-161

### The molecular inhibitory mechanisms of the plant component Ton abnormal vascular contraction and MDA-MB-231cancer cell migration

NAN LI[1], Min Zhang[1], Hakuchou Ro[1], Ying Zhang[1], Hiroko Kishi[1], Tomota Morita[1], Sei Kobayashi[1] ([1]*Dept Mol Cell Physiol, Grad Sch Med, Yamaguchi Univ, Ube, Japan*)

Sphingosylphosphorylcholine(SPC) generated by N-deacylationof sphingomyelin, one of the most abundant lipids in cell membrane, is a phospholipid mediator in blood plasma and has a physiological role in regulation of the heart. Our laboratory discovered SPC/Src(including Fyn)/Rho kinase (ROCK) pathway as a novel pathogenetic pathway for vasospasm, a major cause of sudden death, and further we found the part of this pathway also regulated the cancer cell migration causing lethal metastasis. T is a flavone that is found in tangerine and other citrus peels.It strengthens the cell wall and acts as a plant's defensive mechanism against disease-causing pathogens. With the increasing reports on the role of T the treatment of cardiovascular disease and anticancer, so we aimed to explore whether T have effects on both diseases and the specific the molecular mechanism. (COI:No)

### PP-162

### Crystallographic changes in the decalcified surface of human enamel as seen by Micro-FTIR method

Arata Watanabe[1], Tetsuro Kono[1], Miyuki Toda[2], Ryo Tamamura[1], Toshiro Sakae[1], Hiroyuki Okada[1] ([1]*Department of Histology, Nihon University School of Dentistry at Matsudo,* [2]*Nihon University Graduate School Dentistry at Matsudo*)

This study was carried out to clarify the chemical processes of dental caries in detail, and to clarify whether the caries processes are common through the whole part of various teeth. Human third molars were cut to a thickness of 0.5 mm and immersed in carbonated drinking water for the experimental group and physiological saline for the control group for 1 and 7 days. Among them, one lightly dissolved sample (A) and one heavily dissolved sample (B) were picked up and described. As a result, the micro-FTIR spectroscopy showed the drastic changes in the P-O absorption bands of the outer layer enamel of both samples, while those of the inner layer enamel remained almost unalterably. This result indicated that the erosive processes mainly attacked the phosphate ion environments in the biological apatite crystal structure of tooth enamel. The second order differential curves of the micro-FTIR patterns firstly reported here showed the small but significant P-O band peak shifts among the all analyzing points except for 7d of Sample B, suggesting the individual physicochemical characteristic of tooth enamel apatite. (COI:No)

### PP-163

### Muscle–bone relationships in temporomandibular joint disorders

Hidetomo Hirouchi[1], Satoshi Ishizuka[1], Masahito Yamamoto[1], Mamoru Yotsuya[2], Masaki Sato[3], Sayo Sekiya[1], Yutaro Yamamoto[1], Shinichi Abe[1] ([1]*Department of Anatomy, Tokyo Dental College, Tokyo, Japan,* [2]*Department of Fixed Prosthodontics, Tokyo Dental College, Tokyo, Japan,* [3]*Laboratory of Biology, Tokyo Dental College, Tokyo, Japan*)

Temporomandibular joint osteoarthritis (TMJ-OA) produces degenerative changes in temporomandibular joint tissues, such as the mandibular condyle, that cause bone changes and destruction. However, knowledge gaps remain concerning muscle pathology at the onset of TMJ-OA. Herein, we attempted to document the pathogenesis of bone and muscle at the onset of TMJ-OA using an animal model, mainly using C57BL6J mice. We performed partial resection of the TMJ disc to create a murine model of TMJ-OA mice. After the onset of TMJ-OA, we performed various measurements at 8, 12, and 16 weeks post-surgery in defined groups. Comparing the morphology of the mandibular head between TMJ-OA and a control group of normal mice, the volume of the mandibular head in the TMJ-OA group was significantly increased. Additionally, when observing changes in the temporal muscles, those in the TMJ-OA group were largely deformed, compared with those of the control group. Thus, results showed that the deformity in the temporal muscle increased with hypertrophy of the mandibular head. This study demonstrates that TMJ-OA progresses as organic changes in bone and muscle affect each other. (COI:No)

### PP-164

### Morphological analysis of P2X3-immunoreactive afferent nerve endings in the rat gastric subserosa

Masato Hirakawa[1], Takuya Yokoyama[1], Yoshio Yamamoto[2], Tomoyuki Saino[1] ([1]*Department of Anatomy [Cell Biology]. Iwate Medical University,* [2]*Laboratory of Veterinary Anatomy and Cell Biology. Faculty of Agriculture, Iwate University*)

We investigated the morphology of P2X3 purinoceptor-expressing afferent nerve endings in the rat gastric subserosa by immunohistochemistry of whole-mount preparations. Distribution of P2X3-immunoreactive subserosal endings was restricted in the antral lesser curvature. The immunoreactive endings were morphologically divided into two types: net-like endings and basket-like endings. Net-like endings consisted of web-like terminal structures and peripheral variform axon terminals, and extended two-dimensionally in every direction on the longitudinal smooth muscle layer. Web-like structures and axon terminals surrounded terminal Schwann cells. The morphology of net-like endings may be suitable for receiving the mechanical deformation of gastric wall associated with the antral peristalsis. Basket-like endings consisted of spherical terminal structures surrounding subserosal ganglion, which was characterized by neurons and satellite cells. The close relationships suggested that basket-like endings are involved in the function of subserosal ganglion. A retrograde tracing method using fast blue dye indicated that these endings transmit viscerosensory information via the nodose ganglia. (COI:No)

### PP-165

### Effects of food additives on human colon carcinoma derived cells.

Sakura Onoue[1], Akira Kawata[2], Takeshi Oguchi[3], Yasushi Sasaki[1], Akira Iimura[4], Sho Noguchi[1], Kazuyoshi Higashi[2] ([1]*College of Science and Engineering, Kanto Gakuin University,* [2]*Division of Neuroanatomy, Histology, and Embryology,Department of Oral Science, Graduate School of Dentistry, Kanagawa Dental University,* [3]*Division of Curriculum Development, Kanagawa Dental University,* [4]*Division of Dental Anatomy, Department of Oral Science, Graduate School of Dentistry, Kanagawa Dental University*)

We investigated the effects of four food additives on cell proliferation and morphological changes in the human colon carcinoma cells (Caco-2 cells).
Aspartame, tartrazine, sodium nitrite, and sodium benzoate were added to Caco-2 cells and incubated for 3, 5, 7, and 9 days. The number of viable cells after the culture was determined using the Cell Counting Kit-8. Cytomorphology was observed using a phase-contrast microscope and a scanning electron microscope.
In the control group, polygonal cells of various sizes in monolayers were observed on day 3. The size of the individual cell was reduced and the culture was reached confluence on day 5. Some cells were morphologically changed from squamous cells to columnar epithelial-like cells on day 7. On the other hand, in the food additives-treated group, the cells were polygonal and various cell sizes were observed. The inhibition of cell growth occurred from day 3, and columnar epithelial-like cells were not observed during the experiment period. These results suggested that the cells were not induced to differentiate into intestinal epithelial-like cells when cultured with multiple food additives. (COI:No)

### PP-166

### Three-dimensional distribution of proliferating endocrine cells in parathyroid gland of mice of different ages

Norifumi Tatsumi[1], Hisashi Hashimoto[1], Masataka Okabe[1] ([1]*Department of Anatomy, Jikei University School of Medicine*)

The parathyroid gland is an important organ that releases PTH to maintain normal serum calcium levels. Recently, we reported that the newly proliferating endocrine cells are continuously generated through the division of parathyroid cells, and that the recruitment of these new cells is critical for maintaining the parathyroid function (Yamada et al., 2019). However, the distribution of these proliferating cells in the parathyroid gland is still unclear. In this study, we analyzed the three-dimensional distribution of the new proliferating endocrine cells in parathyroid glands of mice of different ages. Approximately, 50–100 µm thick slices of the tissue of parathyroid gland obtained from mice of different ages were subjected to immunofluorescence staining with anti-Ki67 and anti-Gcm2 antibodies, and their three-dimensional distribution was observed using the confocal laser scanning microscope. The three-dimensional distribution of the proliferating endocrine cells in the parathyroid gland of mice of different ages were revealed upon reconstruction using Imaris. (COI:No)

### PP-167

### The morphological study of the cutaneous glands in the small Japanese mole(*Mogera imaizumii*)

Keiko Sakai[1], Misako Okayasu[1], Minami Okuyama[2], Motonobu Miyazaki[1] ([1]*Saitama City Institute of Health Science and Research,* [2]*Division of Laboratory Animal Science Research Promotion Institute Oita University*)

The small Japanese mole(*Mogera imaizumii*) is a fossorial solitary vertebrate. Because of its subterranean habitat, it is thought to be the odors are important for their behavior. However, there are few studies on the cutaneous glands of moles. In order to clarify the distribution and tissue structure of the secretory glands on the skin of moles, we investigated the skin by anatomical and histological methods with microscope.
Formalin-fixed skin was used for the investigation of the small Japanese moles captured in Saitama City. Paraffin-embedded sections were prepared and these sections were stained with hematoxylin and eosin according to a conventional method to clarify the tissue structure of the glands. In addition, some special stains and immuno-histochemical stains were performed to elucidate the properties of the glands.
From the results of this study, it was found that the small Japanese mole has several secretory glands on its skin. The secretions from these glands may play a protective role by coating the body surface as well as play role of signals detecting each individuals, and further analysis of glandular tissue and its secretions are need to be carried out more.
(COI:No)

### PP-168

### Tight approximation between fibroblasts and intercalated ducts in rat salivary glands

Go Onozawa[1,2], Arata Nagasaka[1], Yudai Ogasawara[1,3], Yasuhiko Bando[1], Koji Sakiyama[1], Osamu Amano[1] (*[1]divijion.anat.Meikai.Univa.schooldent, [2]Divijion.Oral.Surgery1.MeikaiUniva.School.dent, [3]Divijion.Oral.Surgery2.MeikaiUniva.School.dent*)

In salivary glands, acinar and ductal cells are required to link with surrounding connective tissues to exhibit functions such as secretion and reabsorption. The present study investigated localization and morphology of fibroblasts in rat major salivary glands immunohistochemically using 47kDa heart shock protein (Hsp47) as a specific marker of fibroblasts.
Salivary glands of wistar male rats 8-week-old were fixed and cryosected.
In parotid, sublingual, and submandibular glands, Hsp47-immunopositive fibroblasts in the interlobular connective tissue were separated from the large duct and tight approximation between fibroblasts and duct was not recognized. Fibroblasts in the interlobular connective tissue were smaller and lacked tight approximation with acini and striated ducts, however, at the intercalated ducts, many fibroblasts with long processes elongating over the along thin duct were observed. Fibroblastic body and processes were tightly approximated with basal surface of duct cells. This finding was also observed in electron microscopy.
These results suggest that "peri-intercalated duct sheath of fibroblasts" exists in rat major salivary glands. (COI:No)

### PP-169

### Expression of tumor endothelial marker 8 and association with differentiation of tumor cells in canine mammary grand tumors.

Mami Araki[1], Noguchi Syunya[2], Akiko Yasuda[3], Yoshiaki Kubo[3], Miki Koh[1], Hirotada Otsuka[1], Makoto Yokosuka[4], Satoshi Soeta[1] (*[1]Nippon Veterinary and Life Science Univ., Faculty of Veterinary Science, Laboratory of Anatomy, [2]Nippon Medical School, Department of Molecular Medicine and Anatomy, [3]Nippon Veterinary and Life Science Univ., Veterinary Medical Teaching Hospital, [4]Nippon Veterinary and Life Science Univ., Faculty of Veterinary Science, Laboratory of Comparative and Behavioral Medicine*)

Activation of tumor endothelial marker 8 (TEM8) by its ligand endotrophin (ETP) upregulate self-renewal of cancer stem cells and enhance tumor progression in vitro study. In this study, we investigated expression of receptor type TEM8 (TEM8-long) and ETP in tumor cells and analyzed correlation of their expression with histological features and differentiation status of tumor cells in canine mammary gland tumors (CMGTs) by immunohistochemistry. In 214 CMGTs including simple adenoma (n=93), simple carcinoma (n=76) and carcinoma solid (n=45), TEM8-long and ETP was more frequently expressed in simple adenoma and simple carcinoma than in carcinoma solid. Most of TEM8-long+ tumor cells expressed ETP and showed phenotype of CK5+/CK19+/p63-/αSMA- in simple adenoma and simple carcinoma. These results indicate that expression of receptor type TEM8 is associated with luminal progenitor phenotype in tumor cells of CMGTs. Considering TEM8/ETP signaling is involved in cell differentiation of breast cancer cells, our results lead to the possibility that receptor type TEM8 regulates differentiation and maintenance of luminal progenitors in CMGTs through interaction with ETP in autocrine manner. (COI:No)

### PP-170

### Scanning ion conductance microscopy for visualizing the surface topography of rat tissue sections

Yusuke Mizutani[1,2], Manabu Hayatsu[2], Yoshikazu Mikami[2], Tatsuo Ushiki[2] (*[1]Office of Institutional Research, Hokkaido University, [2]Division of Microscopic Anatomy, Graduate School of Medical and Dental Sciences, Niigata University*)

Scanning ion conductance microscopy (SICM) is a type of scanning probe microscopy. The technique uses a glass nanopipette filled with an electrolyte solution as a probing tip that regulates the tip–surface distance by detecting the ion current passing through the aperture of the pipette. Thus, SICM can obtain images of the three-dimensional structures of a sample in liquid condition without mechanical contact.
In our previous studies, we applied SICM for imaging tissue blocks such as the kidney glomerular surface and hair cells of the tracheal lumen. In this study, we used this technique to observe tissue sections.
We first used polyethylene glycol-embedded rat liver sections as samples for observation of the intracellular organelles through transmission electron microscopy (TEM). Thereafter, sections of the liver tissue adjacent to the glycol-embedded sections were observed in a fluid environment, using SICM for imaging of their three-dimensional structures. As a result, we succeeded in obtaining SICM images of the capillary bile ducts, interior of the nucleus, and distribution of mitochondria inside the hepatocytes, with a resolution close to that of TEM. (COI:No)

### PP-171

### Immunohistochemical localization of YAP and TAZ in the mouse molar tooth germ.

Ryo Tamamura[1], Yuichiro Okada[2], Miyuki Toda[2], Arata Watanabe[1], Tetsuro Kono[1], Hiroyuki Okada[1] (*[1]Dept. Histol., Nihon Univ. Sch. Dent. at Matsudo, [2]Nihon Univ. Gra. Sch. Dent. at Matsudo*)

YAP / TAZ is a transcriptional conjugate factor of Hippo signaling pathway, induces various cell responses such as cell proliferation, differentiation and cell death, and is involved in the control of organ size during development. Regarding the expression of Hippo signals in tooth germs, there are some reports on the expression of YAP, but the details are unknown. There are no reports on TAZ. Therefore, in this study, the expression of YAP / TAZ in mouse tooth germ was detected by immunohistochemical staining. The heads of embryos on the 14th (E14) and 16th (E16) days were removed from ICR pregnant mice, fixed with 4 % paraformaldehyde, decalcified with 10 % EDTA, and then embedded in paraffin to make a thin 4 μm section. Immunohistochemical staining was performed using antibodies against YAP / TAZ. Immunostaining results using anti-YAP antibody showed positive findings in the enamel organ of the molar tooth germ. In addition, immunostaining results using anti-TAZ antibody showed localization similar to YAP. From the results of this study, it was suggested that both YAP and TAZ proteins were involved in cell differentiation and proliferation of mouse tooth germ. (COI:No)

### PP-172

### TFAP2E regulates mitotic progression in gingival cancer cells

Kyoko Fujiwara[1,2], Ryo Sakai[3], Tomihisa Takahashi[1,2] (*[1]Dep. Anat., Nihon Univ. Sch. Dent., [2]Div. Funct. Morph., Nihon Univ. Dent. Res. Cent., [3]Nihon Univ. Grad. Dent*)

Mitosis is highly regulated process involving dynamic remodeling of cytoskeletons. Recently, we found that TFAP2E, a member of activator protein-2 transcription factor family, is engaged in the regulation of mitotic progression. ShRNA mediated knockdown of TFAP2E in Ca9-22 cells derived from gingival carcinoma resulted in higher growth rate than those in control cells. In the cell cycle analysis, TFAP2E knockdown cells showed obviously accelerated M-phase exit. Among the control cells synchronized to metaphase by nocodazole treatment, around 20% of the cells entered G1-phase 2 hours after the removal of nocodazole. On the other hand, more than 60% of TFAP2E knockdown cells shifted to G1-phase in the same condition. No obvious difference was observed in the expression and/or activation patterns of cyclins and their regulators. Meanwhile, staining of F-actin with phalloidin revealed that TFAP2E knockdown cells showed shrank morphology with aberrant actin fiber formation in each cell cycle stage. These findings indicate that TFAP2E has certain roles in mitotic exit of cells, probably in the regulation of cytoskeleton dynamics. (COI:No)

### PP-173

### DGKζ deletion dysregulates DNA repair mechanism

Toshiaki Tanaka[1], Kaoru Goto[1] (*[1]Department of Anatomy and Cell Biology, Yamagata University School of Medicine*)

Diacylglycerol kinase (DGK) converts diacylglycerol to phosphatidic acid by phosphorylation. Since both of these lipids are regarded as key molecules in lipid signaling, DGK is considered to be an important regulator in lipid-mediated signal transduction. Of DGKs, DGKζ is characterized by the presence of a nuclear localization signal and localizes to the nucleus in various types of cells. We previously show DGKζ-KO mice are more vulnerable to ionizing radiation than wild-type mice are. However, functional roles of DGKζ in DNA repair remain unknown. We check whether DGKζ depletion promotes DNA double strand break upon DNA damage. gH2AX accumulates at DNA damaged sites and serves as a marker for DNA damage. We found abundant gH2AX staining in siDGKζ-transfected U2OS cells and DGKζ-KO mouse embryonic fibroblasts compared with control cells after transient treatment with DNA damage agents. Moreover, DGKζ knockdown decreases BRCA1 expression. Immunofluorescence assay reveals that DGKζ depletion partially translocates DNA repairing molecules, including p95/NBS1 and DNA-PKcs, to the cytoplasm. These results suggest that DGKζ depletion impairs DNA repair upon DNA damage. (COI:No)

### PP-174

### CoMBI imaging method, its development and spread at the current situation in 2021

Yuki Tajika[1] ([1]*Gunma Univ Grad Sch Med., Dept Anatomy*)

CoMBI (Correlative Microscopy and Block-face Imaging) method has been developed and released in 2017 (Tajika Y et al, Sci. Rep. 3645). The characteristics of CoMBI is capability of obtaining both 3D image and microscopic images in a single biological specimen. The system consists of a cryostat and a digital camera with a macro lens. The camera images all blockfaces for reconstructing 3D images. Cryosections can be obtained as usual while pausing blockface imaging, and processed for microscopy. As a result, the correlation between 3D images and microscopic images can be performed, and improves the reliability of morphological analysis. In addition, CoMBI system is a low-cost 3D imaging system, and easy to construct. Currently, CoMBI system is spreading in the world, and used for analyzing mammals, amphibians, fishes, insects, and plants. I present here the current situation of CoMBI in 2021 and prospects for the future. (COI:No)

### PP-175

### Regulation of *chlamydia trachomatis* infection by autophagy related protein family

Michitaka Suzuki[1], Satoshi Waguri[1] ([1]*Dept. Anat. and Hist., Fukushima Medical Univ*)

*Chlamydia trachomatis* is obligate intracellular bacteria that reside in a membrane bound vacuole called inclusion during their proliferation. Chlamydial proteins are secreted into the cytoplasm of a host cell via type III secretion systems, thereby the host cell is manipulated to ensure *chlamydia* invasion and proliferation. On the other hands, host cells have innate immune-systems including autophagy to protect them from the bacterial infection. However, how autophagy is related to chlamydia infection has been controversial, which should be clarified. To this end, inclusion size was measured in some HeLa cell lines deficient in one of core Atgs. The inclusions were bigger in FIP200-KO or Atg5-KO cells than in control cells, whereas Atg9A-KO or Atg3-KO cells showed smaller inclusions. Also, an infectious progeny assay revealed that *chlamydia* proliferation was inhibited in Atg9A-KO cells. These results suggest that some Atgs act on *chlamydia* invasion or proliferation via mechanisms other than autophagy. (COI:No)

### PP-176

### Elucidation of the mechanisms for the underlying depolarization and reversibility by photo-induced charge separation molecule

Tomohiro Numata[1,4,5], Kaori Sato-Numata[1,2], Hiroshi Imahori[4,5], Tatsuya Murakami[3,5] ([1]*Department of Physiology, Fukuoka University, Japan,* [2]*Japan Society for the Promotion of Science, Tokyo 102-0083, Japan.,* [3]*Department of Biotechnology, Graduate School of Engineering, Toyama Prefectural University, Toyama 939-0398, Japan,* [4]*Department of Molecular Engineering, Graduate School of Engineering, Kyoto University, Kyoto 615-8510, Japan,* [5]*Institute for Integrated Cell-Material Sciences [WPI-iCeMS], Kyoto University, Sakyo-ku, Kyoto 606-8501, Japan*)

Light-induced control of cell membrane potential enables important advances in the study of biological responses. In particular, charge separation molecules (CS molecules) can depolarize cells by photoexcitation without genetic processing. However, the molecular mechanism underlying cell membrane depolarization is unclear, slowing applied research. Here, we show that CS molecules localized in the cell membrane excite cells through a new membrane current regulation mechanism by light irradiation. Photoactivated CS molecules inactivate voltage-gated potassium currents and, at the same time, generate leakage currents due to the loss of cell membrane capacitance. This activity maintains the depolarization of target cells. After depolarizing for a period of time, the cells repolarize with the restoration of membrane conductance function by the cell membrane's regeneration mechanism.

The elucidation of the underlying mechanism of cell photoexcitation by CS molecules highlights unexpected membrane excitability regulatory mechanisms and their potential medical applications. (COI:No)

### PP-177

### The effects of cadmium exposed cell-derived extracellular vesicles on osteoblast differentiation

Wataru Miyazaki[1], Kyoko Mekata[2], Akiko Ido[2], Tsuyoshi Nakanishi[2] ([1]*Dept Biosci and Lab Med, Hirosaki Univ, Hirosaki, Aomori, Japan,* [2]*Lab of Hygienic Chem and Mol Toxicol, Gifu Pharmaceutical Univ, Gifu, Japan*)

Extracellular vesicles (EVs) are released from all cells, and act as one of the intracellular communication pathways in organ homeostasis and diseases. Some stimulus including environmental pollutants also induce the release of EVs, and they cause several effects on the target cells. In this study, we investigated whether EVs from cadmium (Cd)-exposed cells affect osteoblast differentiation. EVs were extracted from the cell culture medium of Cd-exposed human renal proximal tubular epithelial cells: RPTEC/TERT1, and human hepatocellular carcinoma cells: HepG2. The EVs were added at the starting of osteoblast differentiation using human osteoblast cell line: hFOB1.19. We examined the state of differentiation by osteoblastic markers. Cd exposed HepG2-derived EVs suppressed osteoblast differentiation, but not that of RPTEC/TERT1. To clarify the mechanism, we investigated the expressions of both miRNA in EVs and integrins, there were several differences on EVs between RPTEC/TERT1 and HepG2, and also between Cd-exposed cells and no exposed. These results indicated that aberrant EVs released from environmental pollutant-exposed cells may induce several adverse effects to target cells. (COI:No)

### PP-178

### Myotubes aligned by linear-patterning matrix in chick primary culture

Nobuaki Sasai[1] ([1]*Dept Phys Ther, Grad Sch Health sci, Suzuka Univ of Med Sci*)

Myotubes in culture are convenient in the research of muscle hypertrophy and atrophy. However, there are some difficulties on the morphologic evaluation, because the direction of myotube formation is random and diameters of myotubes are very different in usual culture dishes. Consequently, linear-patterning matrix is useful to get myotubes aligned and to improve the evaluation efficiently. The micro-meter width of linear-patterning matrix may function sufficiently; diameters of myotubes are in the order of micro-meters usually. In this study, the commercial products of 15, 30, 60 and 120 μm width were tried in chick primary culture. In 15 μm width, the diameter of myotubes was 12.4 ± 3.2 μm; in the same fashion, 19.0 ± 4.3 μm diameter in 30 μm width, 18.4 ± 6.2 μm diameter in 60 μm width, 16.0 ± 3.1 μm diameter in 120 μm width. Additionally, in 60 and 120 μm width, 2 and 3 myotebes developed abreast, respectively. Though, in this result, 30 μm width is very useful for thick and robust myotube aligned, addional work may be needed. Myotubes become thicker than 30 μm by mechanical or electrical stimulation. (COI:No)

### PP-179

### TMEM35A is differentially allocated in the central nicotinic circuit of the adult mouse brain

Miwako Yamasaki[2], Kotomi Otsubo[1], Kohtarou Konno[1], Taisuke Miyazaki[2], Masahiko Watanabe[1] ([1]*Dept. Anat. Grad. Sch. Hokkaido Univ.,* [2]*Dept.Health. Grad.Sch.Hokkaido Univ*)

Nicotinic acetylcholine receptors (nAChR) are ligand-gated ion channels that mediate physiological actions. Brain nAChRs are assembled from various combinations of a2-7 and b2-4 subunits. TMEM35A is a master regulator in biogenesis of most nAChRs, but its expression profile remains unclear. Here, we investigated TMEM35A expression in the adult mouse brain. In situ hybridization and immunofluorescence showed broad but distinct pattern. It was especially high in glutamatergic neurons in the olfactory bulb, piriform cortex, amygdala, ventromedial hypothalamus, and noradrenergic neurons in the locus coeruleus. Expression was also high in cholinergic neurons in cranial nerve motor nuclei and medial habenula (Ch7), while it was low in other cholinergic neuron groups (Ch1-6). It was extremely low or negative in midbrain dopaminergic neurons. High expression was not related to specific nAChR subunits. Immunoelectron microscopy showed exclusive localization in the endoplasmic reticulum and absence from the cell surface and axon terminals. Thus, TMEM35A is differently allocated in nicotinic circuit and that this may help to accentuate nAChR function in distinct neuronal populations. (COI:No)

### PP-180

### A novel leak channel in cochlear hair cells

Yasuhiro Shibata[1], Yuto Yokoi[1], Natsuko Kumamoto[1], Takashi Ueda[1], Shinya Ugawa[1] ([1]*Dept. of Anatomy and Neuroscience, Grad. Sch. Med. Sci, Nagoya City Univ*)

It is known that background leak conductance exists in cochlear hair cells. In the present study, we investigated the distribution and electrophysiological function of a novel leak channel, designated here as the 'channel X'. Two-electrode voltage clamp of Xenopus oocytes expression system showed that the channel X has inward leak channel activity that is $Gd^{3+}$- and $Zn^{2+}$-sensitive whereas amiloride-insensitive. This inward leak current was increased by a pH drop. Distribution of the channel X in the mouse cochlea was investigated by X-gal staining experiments with channel X LacZ reporter mice. The channel X was found in both inner and outer hair cells in the cochlea. Patch-clamp experiments with outer hair cells from newborns indicated that acid stimuli evoked transient inward currents followed by sustained currents, whereas there were no sustained components in channel X knockout mice. These findings indicate that the functional channel X is located on the plasma membrane of hair cells in the cochlea at least shortly after birth. Further experiments are needed to determine the functional role of the channel X in cochlear hair cells. (COI:No)

### PP-181

### Cellular mechanisms responsible for the rapid depolarization induced by oxygen and glucose deprivation in the mouse somatosensory cortex

Hiroki Toyoda[1], Tsutomu Kawano[1], Hajime Sato[1], Takafumi Kato[1] ([1]*Department of Oral Physiology, Osaka University Graduate School of Dentistry, Japan*)

In the present study, we investigated cellular mechanisms responsible for rapid depolarization caused by oxygen-glucose depolarization (OGD) in layer III pyramidal neurons of the mouse somatosensory cortex. When OGD solution was perfused in the presence of $Ca^{2+}$ chelator, inhibitors of ryanodine receptors (RyRs), inositol 1,4,5-trisphosphate receptors ($IP_3Rs$), NMDA receptors (NMDARs), voltage-gated $Ca^{2+}$ channels (VGCCs) and canonical transient receptor potential (TRPC) channels, the latency of the rapid depolarization was significantly prolonged compared to the control. In addition, when OGD solution was perfused in the presence of calcineurin inhibitors and scavengers of nitric oxide and reactive oxygen species, the latency of the rapid depolarization was significantly prolonged compared to the control. These data indicate that OGD-induced intracellular $Ca^{2+}$ increases mediated by $Ca^{2+}$ influx through NMDARs, VGCCs and TRPC channels as well as by $Ca^{2+}$ release from RyRs and $IP_3Rs$ lead to mitochondrial impairment, which may facilitate the generation of the rapid depolarization via dysfunction of $Na^+$-$K^+$-ATPase due to decreased ATP production. (COI:No)

### PP-182

### TMEM16A inhibition by a licorice-derived flavonoid and estrogen.

Mami Kato[1], Yasunori Takayama[1], Masataka Sunagawa[1] ([1]*Dept Physiol, Showa Univ Sch Med,* )

Transmembrane protein 16A (TMEM16A), a calcium-activated chloride channel, is expressed in primary sensory neurons and pancreatic beta cells. Previous reports suggest that TMEM16A activation enhances pain sensation and insulin secretion. Moreover, TMEM16A is inhibited by some natural compounds. We found that a licorice-derided flavonoid, liquiritigenin, inhibited TMEM16A currents. However, liquiritigenin had no effects on TRPV1, which interacts with TMEM16A in primary sensory neurons. It is also known that liquiritigenin activates estrogen receptor. Therefore, we investigated the effects of sex hormone, including estrone, estradiol, estriol, estetrol and testosterone. Intriguingly, estriol inhibited TMEM16A currents whereas the others were no or slight effects. Estriol is highly synthesized during pregnancy. These results suggest that TMEM16A inhibition by estriol causes diseases of gestation period such as diabetes. (COI:No)

### PP-183

### A novel transporter-independent pathway for acetylcholine synthesis in the mouse retina

Toshiyuki Ishii[1], Kohei Honma[1], Takuma Maruyama[1], Asuka Mano[1], Takumi Akagi[1], Yoshihiko Kakinuma[1], Makoto Kaneda[1] ([1]*Dept. Physiol., Nippon Med. Sch., Tokyo, Japan*)

Choline transport through high affinity choline transporter (hChT) is currently thought to be the only pathway for an acetylcholine (ACh) synthesis in cholinergic neurons. In the retina, cholinergic neurons in ON- and OFF-pathway are thought to function evenly as a parallel processing system. However, we found that the immunoreactivity for hChT was significantly stronger in the ON-cholinergic amacrine cells (ON-CACs) than in the OFF-CACs. We have previously reported that P2X2-purinoceptors ($P2X_2R$), which are permeable to large cations, are specifically located in OFF-CACs. In this study, therefore, we examined whether $P2X_2Rs$ are permeable to $choline^+$, and the $choline^+$ taken up through $P2X_2Rs$ is used for ACh synthesis. An activation of $P2X_2Rs$ by ATP in OFF-CACs induced $choline^+$-mediated inward current in a dose-dependent manner. The permeability to $choline^+$ was also found in $P2X_2R$ expressing HEK293 cells. In this HEK293 cells which are generally expressing choline acetyltransferase, a significant increase in ACh synthesis was detected. These results support the notion that $P2X_2Rs$ can work as choline transport pathway for ACh synthesis especially in the OFF-CACs of the mouse retina. (COI:No)

### PP-184

### Novel mechanisms underlying the abnormal ion selectivity of inherent GIRK mutants

I-Shan Chen[1,2], Yoshihiro Kubo[1,2] ([1]*Division of Biophysics and Neurobiology, National Institute for Physiological Sciences,* [2]*Physiological Sciences, SOKENDAI*)

Inherent gene mutations of G-protein-gated inwardly rectifying $K^+$ (GIRK) channel are relevant to some diseases in human, such as Keppen-Lubinsky syndrome and aldosterone producing adenoma. These mutations induce an abnormal ion selectivity and thereby triggering a disorder of cell functions. To clarify the mechanisms underlying the abnormal ion selectivity, we examined the ion selectivity profile of several mutants of GIRK2 using various size of ions. We observed that G156S located in the selectivity filter (SF) shows high $Li^+$ and $Na^+$ selectivity, that L173R in the transmembrane domain 2 shows high $Rb^+$ and $Cs^+$ selectivity and that S148F and T151A in the pore helix behind the SF also show abnormal ion selectivity. Applications of pore blockers, $Ba^{2+}$ or TPN-Q, change the ion selectivity of G156S and S148F but not those of L173R and T151A, showing that G156S and S148F may possess a second ion permeation pathway besides the central pathway formed by SF and that L173R and T151A may possess only a single ion pathway. These results provide us with a novel insight into the mechanism underlying the abnormal ion selectivity of GIRK mutants. (COI:No)

### PP-185

### Regulation of TRPM7 channel activity via interdomain interaction

Hana Inoue[1], Takashi Murayma[2], Takuya Kobayashi[2], Masato Konishi[1], Utako Yokoyama[1] ([1]*Dept Physiol, Tokyo Med Univ,* [2]*Dept Cell Mol Pharm, Juntendo Univ Grad Sch Med*)

TRPM7 is comprised of two functional domains; the channel domain and the kinase domain. To investigate the regulation of TRPM7 channel activity via the interdomain interaction, patch-clamp recordings were performed in the TRPM7 channel domain (M7cd)-expressing HEK293 cells with or without co-expression of the kinase domain (M7kd) as a separate cytosolic protein. The current was strongly inhibited by intracellular $Mg^{2+}$ ($[Mg^{2+}]_i$) with an $IC_{50}$ of 5.6 μM when M7cd was expressed alone, whereas co-expression with M7kd attenuated the inhibition with an $IC_{50}$ of 349 μM, which was comparable to that of full-length TRPM7. The kinase-inactive mutant M7kd-K1645R also attenuated the $[Mg^{2+}]_i$-dependent inhibition, thus the catalytic activity is suggested to be dispensable for the regulation of $[Mg^{2+}]_i$-sensitivity. On the other hand, mutations in the zinc-binding motif which is important for the structural integrity of M7kd failed to attenuate $[Mg^{2+}]_i$-dependent inhibition. These results indicate that the interaction of M7kd and M7cd attenuates $[Mg^{2+}]_i$-dependent inhibition via kinase-independent mechanisms regardless of whether M7kd is tethered to M7cd. (COI:No)

### PP-186

### Cysteine scanning analysis of the 2nd S4 in two-pore Na+ channel 3

Takushi Shimomura[1,2], Ki-ichi Hirazawa[1,2], Yoshihiro Kubo[1,2] ([1]*Div Biophys and Neurobiol, Natl Inst Physiol Sci,* [2]*Dept Physiol Sci, School of Life Science, SOKENDAI*)

Two-Pore $Na^+$ Channels (TPCs) contain two repeats of a functional unit of voltage-dependent cation channels. In TPCs, the S4 helix in domain II (DII-S4) plays a major role in their voltage sensing. We investigate the activation mechanism of DII-S4 of the TPC3 from *Xenopus tropicalis* expressed in *Xenopus laevis* oocyte, by two-electrode voltage-clamp recording.
Cysteine replacement of each residue in DII-S4 showed position dependent changes of the voltage dependence, which highlighted the prominent contribution of a negative charge (Asp511) in the voltage sensing. $Cd^{2+}$ application to these single cysteine mutants further modified their voltage dependence. We identified, as the counterparts for the mutated cysteines to coordinate $Cd^{2+}$, some endogenous negatively charged residues in DII-S1 and DII-S2. These negative charges are interpreted to locate in the close proximity of introduced cysteines in specific states, providing us with an insight of structural rearrangement of DII-S4. The predicted movement of DII-S4 is different from the S4 movement of canonical *Shaker* $K^+$ channel. Taken together with the importance of Asp511, the results show the unique movement of DII-S4 in TPC3. (COI:No)

### PP-187

### Activation of Voltage-gated proton channel Hv1 via direct interaction with unsaturated tail of fatty acids.

Masataka Inada[1], Akira Kawanabe[1], Yuichiro Fujiwara[1] ([1]*Dept. Mol. Physiol., Fac. Med., Kagawa Univ*)

Although it has been demonstrated that voltage-gated proton channels (Hv1) were activated by the arachidonic acids, the inflammatory lipid mediator, in phagocytes, the detailed mechanism of Hv1 activation is still unclear, due to lack of attention towards each molecular structure of Hv1 and fatty acids (FAs).
We first carried out the voltage clamp measurements, to evaluate the Hv1 activity. According to the analyses with both perfusion of FA solution and cholesterol depletion, we confirmed that more unsaturated FA activated Hv1 more efficiently by their direct interactions, not by modifying the physical property of membrane.
We further quantitatively evaluated Hv1-FA interactions using the surface plasmon resonance (SPR) method. In our analysis, we immobilized Hv1 molecules onto the sensor chip and added solubilized FA solution. Our results indicated that unsaturation of the carbon tail could affect the association of FAs with Hv1.
Thus, our voltage clump measurements and SPR-based analyses succeeded in not only clarifying the effect of FA structure on the Hv1 activity, but also showing the mechanism of Hv1 activation by unsaturated FAs. (COI:No)

### PP-188

### Cytoplasmic structural changes of voltage-sensing phosphatase detected by patch clamp fluorometry

Akira Kawanabe[1], Yuichiro Fujiwara[1] ([1]*Fac. Med., Kagawa Univ*)

Detection of structural changes in the cytoplasmic region of membrane proteins is a challenging work. We previously reported the motion of the cytoplasmic catalytic region of voltage-sensing phosphatase (VSP) by voltage clamp fluorometry with an environment-sensitive unnatural fluorescent amino acid (Anap) (Sakata et al. 2016, Kawanabe et al. 2018). This powerful method sheds light on the intracellular structural changes of membrane proteins in living cells. However, Anap has various disadvantages for fluorescence analysis, a rapid photo-bleaching, a short-wavelength (UV) excitation, and a weak fluorescence. To overcome these problems, we attempted to employ commonly used brighter fluorescent molecules for the cytoplasmic fluorescent labelling under the excised inside-out patch configuration.
We established an electrophysiological recording system equipped with the simultaneous fluorescence imaging (patch clamp fluorometry). Using this system, we succeeded in obtaining the fluorescence signal from a small excided patch membrane expressing mCherry-fused *Ciona intesinalis*-VSP. We are now optimizing the condition regarding the fluorescent labels in the cytoplasmic region. (COI:No)

### PP-189

### Hydrophobic amino acid residues at the C-terminal end of the voltage sensing segment S4 are critical for coupling to an enzymatic activity in voltage-sensing phosphatase

Natsuki Mizutani[1], Akira Kawanabe[2], Yasushi Okamura[1] ([1]*Integrative Physiol, Grad Sch Med, Osaka Univ, Suita, Japan,* [2]*Mol Physiol and Biophys, Fac Med, Kagawa Univ, Kagawa, Japan*)

Voltage-sensing phosphatase (VSP) consists of voltage sensor domain (VSD) and the cytoplasmic catalytic region (CCR). It has been reported that activation of VSD tightly couples with phosphatase activity (VSD-CCR coupling), however, its mechanism is still unclear. We previously found that phosphatase activity depends on hydrophobicity of amino acid side chains at I233 and F234 on the lowest part of S4 of VSD in *Ciona intestinalis* VSP. Among various amino acid mutants, I233Q and F234Y caused a remarkable decrease in phosphatase activity whereas the gating current is normal, indicating an alteration in VSD-CCR coupling. Using voltage clamp fluorometry, we then analyzed voltage-driven motion of the CCR by genetically incorporating unnatural fluorescent amino acid, Anap, in K555. Both mutations suppressed K555Anap signal change, which is tightly associated with inducing phosphatase activity, suggesting that the lowest part of S4 and its hydrophobicity play a critical role in coupling. We now hypothesize that the lowest part of S4 interacts with the hydrophobic spine, a critical residue in the CCR for phosphatase activity and VSD-CCR coupling, to regulate phosphatase activity. (COI:No)

### PP-190

### Biophysical and pharmacological properties of two HCN4 channels in zebrafish

Kaei Ryu[1], Go Kasuya[1], Koichi Nakajo[1] ([1]*Div. of Integrative Physiol., Dept. of Physiol, Jichi Med Univ*)

The hyperpolarization-activated cyclic nucleotide-gated (HCN) channel is a voltage-gated cation channel, which opens on hyperpolarization. Among the HCN subtypes, HCN4 is responsible for pacemaking in the heart's sinoatrial node; therefore, mutations in the HCN4 gene can cause cardiac arrhythmia. Zebrafish, a small tropical fish, is widely used as a vertebrate model animal. While only one HCN4 gene exists in the human genome (*HsHCN4*), two genes exist in the zebrafish genome (*DrHCN4, DrHCN4L*). Functional properties of the two HCN4 channels are, however, not yet characterized. We first compared the biophysical properties of human and zebrafish HCN4 channels expressed in *Xenopus* oocytes and found that DrHCN4L was faster in activation kinetics and showed a rightward-shifted G-V curve compared to HsHCN4 and DrHCN4. Next, we examined the HCN channel blockers such as cesium ions, ZD7288, and ivabradine on these HCN4 channels. Cesium ions and ivabradine similarly inhibited the human and zebrafish HCN4 channels, while ZD7288 was less effective on the zebrafish HCN4 channels. These results offer basic information to those who intend to use zebrafish as a vertebrate heart model. (COI:No)

### PP-191

### Regulation of the function of $K_v$ channels by Sigma-1 receptor

Chang Liu[1,2], I-Shan Chen[1,2], Ruth Murrell-Lagnado[3], Yoshihiro Kubo[1,2] ([1]*Div. Biophys and Neurobiol, NIPS, Okazaki, Japan,* [2]*Physiol. Sci, SOKENDAI, Hayama, Japan,* [3]*School of Life Sciences, University of Sussex, Brighton, UK*)

Sigma-1 receptor (S1R) is a protein expressed mostly on the endoplasmic reticulum membrane and relevant to many psychiatric and neurological disorders. It has been reported to interact with ion channels like $K_v$, $Na_v$ or $K_{ir}$ channels, while the underlying mechanisms are unknown. To clarify how S1R interacts with different types of ion channels, we performed experiments and observed the results as follows: (1) By immunohistochemical staining, we confirmed S1R can be expressed in the oocytes injected with S1R cRNA. (2) By recording the current by two-electrode voltage-clamp using *Xenopus* oocytes, we observed that coexpression of S1R with $K_v1.2$, $K_v2.1$ or $K_v2.2$ channel increases their total current amplitude. (3) By mutagenesis, we made the mutants of $K_v2.1$ (Ser583Ala and Ser586Ala) which disturb the channel clustering. S1R did not affect the current amplitude of these mutants, suggesting the effect of S1R on $K_v2.1$ may due to the influence on the clustered channels. Taken together, our data shows that S1R regulates the function of $K_v1.2$, $K_v2.1$ and $K_v2.2$ channels, and further experiments are needed to elucidate the underlying mechanisms. (COI:No)

### PP-192

### Analysis of protein candidates interacting with voltage-gated sodium channel Nav1.1

Ikuo Ogiwara[1] ([1]*Dept Physiol, Nippon Med Sch, Tokyo, Japan*)

Voltage-gated sodium channel, Nav1.1, is predominantly expressed in parvalbumin-expressing inhibitory cells. We have been screening protein candidates binding to Nav1.1. We here found that the two candidate proteins interacted with the intracellular loop I, but not the C-terminal domain (CTD), of Nav1.1 with immunoprecipitation assays using heterologous expressing systems with 293T cells. We also tested whether the CTD of Nav1.1 interacted with calmodulin and fibroblast growth factor 13, which have been reported to bind to other voltage-gated sodium channels and modulate their channel properties. As expected, we found that calmodulin interacted with the CTD, but not the intracellular loop I, of Nav1.1. However, we failed to detect interaction between fibroblast growth factor 13 and the C-terminal domain or intracellular loop I of Nav1.1. Our findings may suggest that these Nav1.1-binding partners may be involved in axonal localization and modulation of electrophysiological properties of Nav1.1. (COI:No)

### PP-193

### The distal C-terminal region of the THIK channels plays critical roles in the regulation of the channel activity

Michihiro Tateyama[1,2], Yoshihiro Kubo[1,2] ([1]*Biophys & Neurobiol, NIPS,* [2]*SOKENDAI*)

We previously reported that the THIK-1 and THIK-2 channels, the members of the two-pore-domain potassium channel family, are activated either by Gi/o- or Gq-coupled receptors. The THIK-1 channel activity was reported to be enhanced by the caspase 8 dependent cleavage at Asp330 of the C-tail. We observed that the THIK-1 channel mutant truncated at Asp330 showed a remarkable increase in the basal current density, and that the truncation decreased the channel responses to the activation of the Gi/o-coupled metabotropic glutamate receptor mGlu2 and Gq-coupled adrenergic receptor alpha1A-AR. These results suggested that the C-tail plays important roles in the regulation of the channel activity. To identify the important residues in the C-tail, we constructed several truncated mutants of the THIK-1 and THIK-2 channels. We observed that the last 11 residues play regulatory roles in the basal channel activity and the alpha1A-AR induced activation. These results showed that the distal C-terminal region of the THIK channels play important roles in the regulation of the channel activity. (COI:No)

### PP-194

### The actions of nondepolarizing neuromuscular blocking agents at zebrafish muscle nicotinic acetylcholine receptor isoforms

Souhei Sakata[1], Manami Yamashita[1], Yoshihiro Egashira[1], Fumihito Ono[1] ([1]*Dept. Physiol., Faculty of Medicine, Osaka Medical College*)

Nondepolarizing neuromuscular blocking agents (NMBAs) are muscle relaxants clinically used for anesthesia. Many NMBAs are believed to competitively inhibit the muscle nicotinic acetylcholine receptor (nAChR). Recent works using zebrafish demonstrated that nAChRs in fast fibers contain ε subunits , while those in slow fibers lack ε subunits. Because the δ subunit substitutes the ε subunit in slow fibers, we designate these two types of nAChRs as ε-type and δ-type.

We expressed these two types of nAChRs in Xenopus oocytes and found that the half maximal inhibition concentration ($IC_{50}$) of pacuronium, one of the NMBAs, was clearly lower in the ε-type nAChR than the δ-type receptor. Chimera experiments between the ε- and the δ-type nAChR revealed that $IC_{50}$ was associated not with the extracellular ligand binding region but the transmembrane region. This is inconsistent with the view that NMBAs are competitive inhibitors. We are currently analyzing the $IC_{50}$s of pancuronium analogs, such as vecuronium and rocuronium, to examine if these drugs are competitive inhibitors for ε- and δ-type nAChR. This study sheds light on the blocking mechanisms of NMBAs at the muscle nAChR. (COI:No)

### PP-195

### Analysis of the activation mechanism of Two-pore channel 3 by membrane voltage and phosphoinositide

Ki-ichi Hirazawa[1,2], Takushi Shimomura[1,2], Yoshihiro Kubo[1,2] ([1]*Div Biophys and Neurobiol, Natl Inst Physiol Sci, Okazaki, Japan,* [2]*Dept Physiol Sci, SOKENDAI, Hayama, Japan*)

Two-pore channel 3 (TPC3) is a voltage-gated cation channel and its polypeptide has two repeats of canonical 6 transmembrane motif. The 4$^{th}$ helix in the 2$^{nd}$ repeat (the 2$^{nd}$ S4) of TPC3 is important to sense the membrane voltage, while the phosphoinositide (PI) binds to the 1$^{st}$ repeat to potentiate the voltage sensitivity of TPC3. To reveal the effect of PI on the structural dynamics of the 2$^{nd}$ S4, we analyzed the accessibility of MTSES, a covalent modifier of Cys, to the introduced Cys at the extracellular side of the 2$^{nd}$ S4 (D511C) by two electrode voltage-clamp technique using *Xenopus* oocyte. PI binding accelerated the MTSES-modification, suggesting a potentiation of the structural change of the 2$^{nd}$ S4 by PI. Next, by the voltage clamp fluorometry of TPC3 that was labeled by a fluorescent unnatural amino acid, Anap, at S527 at the bottom of the 2$^{nd}$ S4, we succeeded in the detection of the structural change of the 2$^{nd}$ S4 as the fluorescent intensity change (F change). PI increased the extent of the F change. Taken these two results together, we conclude that the PI binding in the 1$^{st}$ repeat can potentiate the activation of the 2$^{nd}$ S4. (COI:No)

### PP-196

### Molecular basis for sugar specificity of $Na^+$/glucose co-transporters-How are sugars taken into our bodies?-

Kazuyo Kamitori[1,2], Yuichiro Fujiwara[1,2] ([1]*Department of Molecular Physiology and Biophysics, Faculty of Medicine, Kagawa University, Japan,* [2]*International Institute of Rare Sugar Research and Education, Kagawa University* )

Human $Na^+$/glucose cotransporters (SGLTs) are abundant in small intestine and kidney where they contribute to the glucose absorption. Tumor cells highly express SGLTs to facilitate glucose uptake resulting in unlimited tumor growth. These physiological and pathological roles made SGLTs target for diabetes or cancer treatment. Seven human SGLT members have been reported, and sugar specificity for each SGLT are precisely regulated depending on their roles. Understanding structural basis of SGLTs sugar specificity would provide important information for clinical strategies.

To clarify structural basis of SGLT sugar specificity, we have reported the molecular modeling of SGLT1 binding to the sugar. We have also performed electrophysiological studies for SGLT1 for various sugars. Here we analyzed sugar specificities of SGLT1 mutants at around the sugar binding pocket using two-electrode voltage clamp method. The results showed several of mutants changed sugar specificity similar to other SGLT members. The observation revealed sugar specificity of SGLTs are regulated by the structure around sugar binding pocket, and raised the possibility to control sugar uptake or selectivity via SGLTs. (COI:No)

### PP-197

### Mechanisms of permeation and selectivity in $Ca_V1.3$ L-type calcium channels

Futoshi Toyoda[1], Akinori Noma[2], Yukiko Himeno[2], Wei-Guang Ding[1], Hiroshi Matsuura[1] ([1]*Dept Physiol, Shiga Univ Med Sci,* [2]*Dept Bioinformatics, Col Life Sci, Ritsumeikan Univ*)

We have recently identified $Ca_V1.3$ L-type $Ca^{2+}$ channel as a molecular determinant for the sustained inward $Na^+$ current ($I_{st}$) that is a key player in cardiac pacemaker activity. However, it remains unclear how $Ca_V1.3$ could mediate both $Ca^{2+}$ and $Na^+$ currents. Here we report experimental and theoretical realization of competitive permeation of $Ca^{2+}$ and $Na^+$ through $Ca_V1.3$ channels. In patch-clamp recordings, $Ca_V1.3$ typically elicited L-type $Ca^{2+}$ current ($I_{Ca,L}$) at -10 mV, which was gradually decreased and mostly disappeared as the external $Ca^{2+}$ concentration ($[Ca^{2+}]_o$) was lowered from 1.8 to 0.01 mM. Further reduction in $[Ca^{2+}]_o$ («1 μM) conversely induced an inward current, indicating a switch of conducting ion from $Ca^{2+}$ to $Na^+$. On the other hand, $Ca_V1.3$ also evoked a sustained inward current at -60 mV, assumed to be $I_{st}$, which was only increased by lowering $[Ca^{2+}]_o$. Theoretical analysis using a classical permeation model (Almers & McCleskey,1984) well explained the experimental observation of anomalous mole-fraction dependence of $I_{Ca,L}$ as well as the generation of $I_{st}$, but predicted the presence of two different permeation modes with distinct $Ca^{2+}$ sensitivity in $Ca_V1.3$ channels. (COI:No)

### PP-199

### Hysteretic behavior of the tension-dependent gating of the KcsA potassium channel revealed by the dynamic manipulation of membrane tension

Masayuki Iwamoto[1], Shigetoshi Oiki[2] ([1]*Dept. Mol. Neurosci., Univ. Fukui Facul. Med. Sci.*, [2]*Biomed. Imaging Res. Center, Univ. Fukui*)

In the living cell, membrane tension varies upon cellar events such as cell migration, swelling, and mitosis. The ion channels are embedded in the membrane and function in such a dynamic tension environment. However, the dynamic aspect of the tension-sensitive feature of the ion channel has been unclear. In this study, we developed a time-lapse tension measurement system combined with electrophysiological recordings. A pair of monolayer-lined water bubbles were inflated at the tip of pipettes in the oil and contacted each other to form a lipid bilayer. Here, the bubble's pressure determines the bubble surface (lipid monolayer) and the lipid bilayer tensions through the Young-Laplace and the Young principles. Thus, the tension is evaluated or manipulated through intra-bubble pressure. The single-channel current of the prototypical KcsA potassium channel was examined under dynamically manipulated membrane tension (1–6 mN/m). We found that the activation gate of the KcsA channel exhibited distinct tension sensitivity upon stretching and relaxing. The hysteretic tension sensitivity suggests that the mode shift of the conformation occurs depending on the membrane tension. (COI:No)

### PP-200

### Mechanisms of hERG channel current inhibition by an anticonvulsant valproic acid

Shinichiro Kume[1], Xiufang Zhu[1], Pu Wang[1], Mengyan Wei[1], Kenshi Yoshimura[1], Tatsuki Kurokawa[1], Katsushige Ono[1] ([1]*Dept Patphysiol, Grad Sch Med, Oita Univ, Japan*)

Drug-induced QT-interval prolongation is known as a risk factor for lethal arrhythmias. Valproic acid is widely used as anticonvulsant, an inhibitor of histone deacetylase (HDAC) class I and IIa, and reported to be also involved in the QT prolongation. However, detailed mechanisms of valproic acid-associated QT prolongation are poorly understood. In this study, we evaluated the possible effects of valproic acid focusing the action on hERG channel current ($I_{Kr}$). Long-term (24 h) but not short-term (5 min) application of valproic acid decreased the $I_{Kr}$ amplitude with a dose-dependent manner in heterologous expression system using HEK293 cells expressing hERG channel. Among the four groups of HDAC inhibitors, only short-chain fatty acids derivatives (valproic acid and butyric acid) decreased $I_{Kr}$ amplitude as a long-term effect. When HEK293 cells expressing hERG channel were treated by inhibitors of transcription, translation or N-glycosylation, valproic acid was unable to reduce $I_{Kr}$ amplitude any further. These results suggest that valproic acid suppresses the hERG channel through HDAC-mediated processes as a class effect of short-chain fatty acid derivatives. (COI:No)

### PP-201

### Optical monitoring of voltage-dependent gating of ATP-gated channel P2X2 by utilizing fluorescent unnatural amino acid (fUAA)

Rizki Tsari Andriani[1,2], Yoshihiro Kubo[1,2] ([1]*Division of Biophysics and Neurobiology, National Institute for Physiological Sciences, Okazaki, Japan*, [2]*Department of Physiological Sciences, School of Life Science, The Graduate University for Advanced Studies [SOKENDAI], Hayama, Japan*)

P2X2 is a ligand-gated ion channel which opens upon the binding of extracellular ATP. The gating of this channel has been shown to be not only [ATP]-dependent but also voltage-dependent, in spite of the absence of a canonical voltage sensor domain within its structure. It remains unknown how the structural rearrangements occur during the voltage-dependent gating. This study aimed to analyze the structural movements of [ATP]- and voltage-dependent gating of P2X2 receptor by optical recording utilizing fluorescent unnatural amino acid (fUAA). We directly made a fUAA named Anap incorporated into amber codon mutants of the rat P2X2 in *Xenopus* oocytes and performed voltage-clamp fluorometry recordings. We found an electric field convergence at Ala337 and Ile341 in the TM2. We also observed a hyperpolarized-induced conformational changes at around Ala337 in TM2. Finally, mutagenesis studies at Ala337 in TM2 and Phe44 in TM1 suggested that the interaction between these two residues in the ATP-bound open state is important for the complex gating of P2X2 receptor. These findings provide us with a clue to understand the mechanism of voltage-dependent gating in P2X2 receptor. (COI:No)

### PP-202

### Relationship between polytheonamide B channel activity and the thickness of lipid bilayers

Yuka Matsuki[1], Masayuki Iwamoto[2], Masako Takashima[2], Shigetoshi Oiki[3] ([1]*Dept. Anesth. Reanimatol., Univ. Fukui Fac. Med. Sci.*, [2]*Dept. Mol. Neurosci., Univ. Fukui Facul. Med. Sci.*, [3]*Biomed. Imag. Res. Cent., Univ. Fukui*)

A peptide from marine sponge *Theonella swinhoei*, polytheonamide B (pTB), shows potent cytotoxic activity. pTB is a 48-mer peptide, forming a cation permeation pore in the $\beta$-helical structure . The length of pTB is about 4 nM, which is comparable to the thickness of lipid bilayers. pTB spontaneously penetrates the membrane and exhibits channel activity. In the lipid bilayer method, the membrane thickness is freely controlled and is evaluated by measuring the specific membrane capacitance using the membrane capacitance and membrane area. We measured single-channel currents of pTB channel in lipid bilayers having variable thickness using a lipid bilayer method called the contact bubble bilayer. We found that the single-channel activity of the pTB channel varied substantially depending on the membrane thicknesses. The molecular mechanism will be discussed. (COI:No)

### PP-203

### Investigation of the interaction between TRPV3 and TMEM79 in mouse keratinocytes

Jing Lei[1], Makoto Tominaga[1] ([1]*National Institute for Physiological Sciences [NIPS]*)

Itch-specific therapies of skin disease have been put in great effort to investigate the underlying mechanisms and potential targets. Cation-permeable transient receptor potential V3 (TRPV3) is predominantly expressed in skin keratinocytes, participating in physiological progress ranging from somatosensation to inflammation. TRPV3 level is increased in isolated keratinocytes from mouse model of atopic dermatitis (AD), a most common chronic skin disease with spontaneous itch and skin barrier disfunction, and gain of function mutation of TRPV3 is known to be related with a hereditary skin disease, Olmsted syndrome with severe itch. However, the underlying mechanism of how TRPV3 is implicated in itch signaling is still poorly understood. Interestingly, a less known transmembrane protein 79 (TMEM79) has been introduced to play roles for pathogenesis of AD in mice. These facts prompted us to consider a possible interaction between TRPV3 and TMEM79 in skin keratinocytes causing itch. This project will provide a novel insight into understanding the mechanism based on interaction between TRPV3 and TMEM79, which may introduce a novel therapy for itch and skin disease in the future. (COI:No)

### PP-204

### 5-HT$_{1A}$ and 5-HT$_{1B}$ receptor-mediated inhibition of glutamatergic synaptic transmission onto rat basal forebrain cholinergic neurones

Toshihiko Momiyama[1], Takuma Nishijo[2], Etsuko Suzuki[1] ([1]*Dept Pharmacol Jikei Univ Sch med*, [2]*Dept Mol Neurobiol Inst Dev Res Aichi Dev Disability Center,*)

A whole-cell patch-clamp study was carried out to elucidate 5-HT receptor mediated modulation of glutamatergic synaptic transmission onto BF cholinergic neurons. BF cholinergic neurons were identified with Cy3-192IgG and investigated in P12-20 rat brain slices. Excitatory postsynaptic currents (EPSCs) were evoked by focal electrical stimulation. 5-HT, a 5-HT$_{1A}$ receptor agonist or a 5-HT$_{1B}$ receptor agonist inhibited the amplitude of EPSCs. In the presence of both 5-HT$_{1A}$ and 5-HT$_{1B}$ receptor antagonists, most of 5-HT-induced effect disappeared. 5-HT- or a 5-HT$_{1B}$ receptor agonist-induced inhibition was significantly smaller in the presence of $\omega$-agatoxin TK than that without $\omega$-agatoxin TK. 5-HT- or a 5-HT$_{1A}$ receptor agonist reduced synaptic strength by changing AMPA/NMDA ratio. These results suggest that 5-HT inhibits glutamatergic transmission onto BF cholinergic neurons via both 5-HT$_{1A}$ and 5-HT$_{1B}$ receptors with different mechanisms. (COI:No)

### PP-205
### The role of the GON Domain on IP3R and calcium homeostasis

Sawako Yoshina[1], Shohei Mitani[1,2] ([1]*TWMU*, [2]*TIIMS*)

ADAMTS9 is a metalloprotease that cleaves components of the extracellular matrix and is also implicated in intracellular protein transport. ADAMTS9/GON-1 has a unique C-terminal domain called the "GON domain". The function of intracellular protein transport is dependent on the GON domain but independent of protease activity. However, molecular mechanisms of ADAMTS9 in cells remain unknown.
To investigate the intracellular role of ADAMTS9/GON-1, we searched for genes whose depletion suppressed the *gon-1* phenotype. We identified several suppressor genes. To determine whether the GON domain interacts with the suppressor genes, we performed immunoprecipitation experiments. We found that the GON domain interacts with several suppressor gene products. The suppressor genes included a molecule involved in ubiquitination of inositol 1,4,5-trisphosphate receptor (IP3R). We examined the ubiquitination level of the IP3R and found that the ubiquitination of IP3R was increased by ADAMTS9 depletion. Furthermore, we found that the GON domain depletion increased $Ca^{2+}$ leak from the ER lumen to the cytosol through IP3R. This leak was suppressed by inhibiting ubiquitination of IP3R. (COI:No)

### PP-206
### Structural basis for promiscuous action of monoterpenes on TRP channels

Thi Hong Dung Nguyen [1], Satoru Ito[2], Satoshi Okumura[2], Makoto Tominaga[1] ([1]*National Institute for Physiological Sciences*, [2] *Institute for Molecular Science*)

Monoterpenes Are Major Constituent S Of Plant-Derived Essential Oil S And Have Long Been Widely Used For Therapeutic And Cosmetic Applications . The Monoterpenes Menthol And Camphor Are Agonists Or Antagonists For Several TRP Channels Such As TRPM8, TRPV1, TRPV3 And TRPA1. However, Which regions within TRPV1 and TRPV3 confer sensitivity to monoterpenes or other synthesized chemicals such as 2-APB are unclear. In this study we identified conserved arginine and glycine residues in the linker between S4 and S5 that are related to the action of these chemicals and validated these findings in molecular dynamics simulations. The involvement of these amino acids differed between TRPV3 and TRPV1 for chemical-induced and heat-evoked activation. These findings provide the basis for characterization of physiological function and biophysical properties of ion channels. (COI:No)

### PP-207
### Investigation of the possible relation between TRPM2--ANO1 ion channels

Aykut Deveci[1,2], Makoto Tominaga[1] ([1]*Division of cell signaling NIPS OKAZAKI*, [2]*SOKENDAI*)

The divalent cation calcium ($Ca^{2+}$) is described as one of the most important biological cation. It is used by all living cells as an intracellular signaling messenger that controls many biological processes. It is also involved in the pathophysiology of the cells and cell fate. $Ca^{2+}$ is maintained at low levels in the cytosol and is mainly concentrated outside the cell or in intracellular compartments, particularly in the endoplasmic reticulum (ER), mitochondria and Golgi apparatus. Fluxes of $Ca^{2+}$ through the membranes are provided by transporters and ion channels present at the plasma membrane and the intracellular compartments. Among these, TRP Melastatin 2 (TRPM2) is highly expressed in several tissues. It is a $Ca^{2+}$-permeable, non-selective cation channel which exhibits heat sensitivity. It acts as a biosensor of oxidative and osmotic stresses under physiological and pathological conditions. Moreover, $Ca^{2+}$ influx induced by TRPM2 could activate other $Ca^{2+}$-dependent channels like $Ca^{2+}$-activated chloride channel anoctamin-1 (ANO1) and intermediate $Ca^{2+}$-activated potassium channel ($IK_{Ca}1$). (COI:No)

### PP-208
### Development of Mathematical Models of Three Types of Ca2+ Channels in the Sinoatrial Node Pacemaker Cell Based on Kinetics Revealed in Expressed Cells

Yixin Zhang[1] ([1]*Graduate School of Life Sciences, University of Ritsumeikan, Japan*)

There are two subfamilies of voltage-gated Ca2+ channels in mammalian cardiac pacemaker sinoatrial node (SAN) cells, L-type (CaV1.2, CaV1.3) and T-type (CaV3.1) channels, which are distinguished by their electrophysiological and pharmacological properties. Mathematical models provide a promising tool for understanding different roles of ionic channels in the pacemaker mechanisms in SAN cells. However, the gating parameters of L- and T-type Ca2+ channels obtained from previous patch-clamp experiments in SAN cells vary considerably among studies, at least partly due to technical difficulties in pharmacological separation of the currents. In the present study, we investigated current properties of CaV1.2, CaV1.3 (and CaV3.1) channels by whole-cell patch-clamp analysis using heterologously expressed cells. Our current recordings successfully revealed distinct features of voltage-dependent activation and inactivation in each type of channels, providing a molecular-based functional subtyping of L-type and T-type channels. By incorporating those kinetics of the Ca2+ channels into a SAN cell model, it was concluded that CaV1.3 was more potent in increasing the firing frequency than CaV1.2. (COI:No)

### PP-209
### Functional rescue for disease-associated class II CFTR mutations frequent in Japanese cystic fibrosis patients

Yoshiro Sohma[1], Masahiro Shimizu[1], Shiori Okawa[1], Yuka Matsuzawa[1], Suzuna Kaneko[1], Yuki Fukada[1], Kazuki Narita[1], Yuta Naoi[1], Rio Kimishima[1], Hikaru Soma[1], Shogo Iwai[1], Nao Kobayashi[1], Kanako Nakao[1] ([1]*Division of Molecular Therapy, Graduate School of Pharmacy, IUHW* )

Cystic Fibrosis (CF) is reported to be very rare among Asiansand previous reports suggested that the profiles of CF-caused CFTR mutations found in Japanese CF patients are different from those in Caucasians.
At present twenty-four Japanese CF patients have been definitely diagnosed with their disease-associated CFTR mutations. Twenty-two mutations were identified in the CFTR proteins. 11 out of 22 mutations seemed to be classified into the class II (trafficking defect) and 10 mutations into the class III (regulatory dysfunction) or IV (ionic conduction defect). Recently a few chemical chaperons for the class II mutation F508del most frequent in Caucasians (CFTR correctors) have been developed and approved by FDA. Unfortunately the most frequent Japanese mutant Δ(G970-T1122)-CFTR could not be rescued by the Vertex CFTR correctors.
In this study, we investigated the function-loss mechanisms of the Japanese disease-associated CFTR mutations with test the effects of the CFTR correctors on them. (COI:No)

### PP-210
### Molecular basis for the reduced heat perception in frog tadpoles inhabiting hot springs

Shigeru Saito[1,2,3], Kurea Saito[1,2], Takeshi Igawa[4], Shohei Komaki[5], Makoto Tominaga[1,2,3] ([1]*Cell Signaling, NIPS*, [2]*Thermal Biology, ExCELLS*, [3]*Physiol Sci, SOKENDAI*, [4]*Amphibian Research Center, Hiroshima Univ*, [5]*Iwate Tohoku Medical Megabank Organization*)

Environmental temperature is a critical factor for most of lives, and the upper and lower thermal limits for survival define habitable niches of the species. However, evolutionary changes in thermal perception are required in addition to enhancement of thermal tolerance in order to utilize extreme thermal niches. Tadpoles of *Buergeria japonica* possess extreme heat resistance and inhabit in natural geothermal hot springs. We here examined behavioral responses of tadpoles and also examined the properties of heat sensors in order to understand the molecular basis of thermal perception. We found that warm acclimated tadpoles of *B. japonica* elevated avoidance temperatures up to 42℃, which was not seen in a closely related species *Buergeria buergeri*. In addition, activity of heat sensors such as TRPV1 and TRPA1 was nearly lost in *B. japonica*. Our findings highlight the importance of evolutionary changes in thermal perception in environmental adaptation. (COI:No)

### PP-211

### Pathophysiological role of the HCN4-positive, excitatory interneurons in the spinal dorsal horn of murine allodynia model.

Taku Nakagawa[1], Toshiharu Sayaka[2], Noriyuki Nakashima[1], Kensuke Oshita[3], Yuta Kouro[4], Makoto Tsuda[4], Makoto Takano[1] (*[1]Department of Physiology, Kurume University, Japan, [2]Department of Health and Nutrition, Niigata University of Hearth and Welfare, Niigata, Japan., [3]Department of Anesthesiology, Kurume University School of Medicine, Kurume, Japan., [4]Department of Life Innovation, Graduate School of Pharmaceutical Sciences, Kyushu University, Fukuoka, Japan*)

The hyperpolarization activated, cyclic nucleotide gated channels (HCN1-4) are recently implicated in the nociception. Using transgenic mice in which tetracycline genetic switch and luciferase cDNA were knocked into the genetic locus of HCN4 ($HCN4^{Luc/tTA\_TRE}$), we reported that HCN4 is expressed in the excitatory interneurons of ventral part of the inner lamina II ($II_{iv}$) ~ lamina III of the spinal dorsal horn (SDH). Because these areas may be responsible for the mechanical allodynia, we aimed to clarify pathophysiogical roles of HCN4-positive neurons in the mechanical allodynia. For this purpose, we injected adeno-associated virus vector carrying TRE sequence and cDNA of Gs-coupled designer receptors exclusively activated by designer drugs into the SDH of $HCN4^{Luc/tTA\_TRE}$ mice. Three weeks after, we generated allodynia model by injuring $4^{th}$ Lumbar spinal nerve. Immunohistochemical analysis revealed that cFos expression in lamina I was induced by paint brush stimulation 2 weeks after the nerve injury. The activation of HCN4 by CNO reduced the cFos expression. We speculated that the activation of HCN4 reduced the crosstalk from tactile circuitry to pain transmitting circuitry. (COI:Properly Declared)

### PP-212

### Adverse effects of A$\beta_{1\text{-}42}$ oligomers: contextual learning and $GABA_A$ synapses in CA1 pyramidal neurons

Yuya Sakimoto[1], Yutaro Tsukada[3], Thein-Oo Paw-Min[1], Hiroyuki Kida[1], Ryoichi Kimura[2], Dai Mitsushima[1] (*[1]Department of Physiology Yamaguchi University Graduate School of Medicine, [2]Cenr Liberal Arts and Scienceter fos Sanyo-Onoda City Univ, [3]Department of Medicine Yamaguchi University*)

Although A$\beta_{1\text{-}42}$ oligomers is considered as a pathogenesis of Alzheimer's disease, the adverse effects for hippocampal learning and CA1 synapses are not well known. To analyze the effects, rats were microinjected the A$\beta_{1\text{-}42}$ oligomers or saline into the bilateral CA1. Although the oligomers did not affect the performance in elevated-plus maze or hanging wire, it impaired the contextual learning.
To analyze the influence at CA1 synapses, the oligomers were unilaterally microinjected into the CA1. One week after the microinjection, we prepared acute brain slices to analyze miniature inhibitory post-synaptic currents(mIPSC). The oligomers significantly impaired both amplitude and frequency of mIPSC in CA1 pyramidal neurons. Moreover, the oligomers significantly increased the paired-pulse ratio, suggesting an impairment of presynaptic GABA release probability. To further specify the postsynaptic target, we will use non-stationary fluctuation analysis to analyze the single channel current as well as the number of postsynaptic $GABA_A$ receptor channel(Sakimoto et al, CerebCortex 2019). The analysis of adverse effects would be necessary to identify the pathogenesis of Alzheimer's disease. (COI:No)

### PP-213

### Analyses of two fused teeth of the upper second molar and third molar using histological and analytical study

Hiroyuki Mishima[1], Toshihide Niimi[2], Yasuo Miake[3] (*[1]Department of Dental Engineering, Tsurumi University School of Dental Medicine, [2]Niimi Oral Care, [3]Department of Oral Anatomy, Tsurumi University School of Dental Medicine*)

The purpose of this study is to investigate the structure and development process of the two fused teeth. In the case of the 26 years old, enamel was observed in the pulp cavity of the third molar. The enamel cavity was more radiolucent than normal enamel. The interglobular dentin was observed remarkably in the third molar. Vasodentin was present at the junction between the second molar and third molar. In the case of the 53 years old female, the surface of third molar was thickly covered with cementum. It is considered that these fused teeth were formed by the fusion of the second molar germ and third molar germ in the early development stage. (COI:No)

### PP-214

### Expression analysis of factors involved in NSC properties in the developing hippocampus.

Taichi Kashiwagi[1], Seiji Shioda[2], Tatsunori Seki[1] (*[1]Dept. Histol. and Neuroanat., Tokyo Med. Univ., [2]Dept. Anat., Showa Univ. Sch. of Med*)

In the dentate gyrus (DG) of the hippocampus, neural stem cells (NSCs) are maintained throughout life unlike most other brain regions where neurogenesis is completed around birth, suggesting that NSCs in the DG are endowed with special properties and/or special circumstances are existed to maintain undifferentiated status of NSCs. However, the property of NSCs in the DG have not been fully elucidated yet. Since NSCs are maintained by self-renewal, it is thought adult NSCs are derived from embryonic NSCs. Thus, to identify factors that involve in DG NSC properties, we focused on the developing hippocampus. We previously showed that Gfap-expressing cells that contain NSCs confined to the dentate primordium during early stages of neurogenesis and produce DG granular cells (GCs). Gfap-expression is a common feature with adult DG NSCs. In the current study, we isolated candidate factors from our previous DNA microarray analysis comparing gene expression between developing neocortex and hippocampus and investigated the expression of candidate factors in the Gfap-GFP transgenic mice. (COI:No)

### PP-215

### Hypoxia-induced downregulation of Sema3a and CXCL12/CXCR4 regulate the formation of the coronary artery stem at the proper site

Yuji Nakajima[1], Mayu Narematsu[1], Tatsuya Kamimura[2], Toshiyuki Yamagishi[3] (*[1]Osaka City Univ, Grad Sch Med, Anatomy and Cell Biology, [2]Asahi Univ, Sch Dent, Oral Anatomy, [3]Saitama Med Univ, Faculty of Health and Medical Care, Sch Medical Technology*)

During the formation of the coronary artery (CA) stems, endothelial cell (EC) strands from the EC progenitors surrounding the conotruncus penetrate into the aorta. VEGFs and CXCL12/CXCR4 are thought to play a role in the formation of CA stem. However, the mechanisms how EC strands exclusively invade into the aorta remain unknown. IHC showed that Sema3a was expressed in EC progenitors surrounding the great arteries. At the onset of/during invasion of EC strands into the aorta, Sema3a was downregulated and CXCR4 was upregulated in the EC strands. ISH showed that *Cxcl12* was expressed in the aortic wall. In culture, Sema3a blocking peptide induced an excess EC strands penetrating the pulmonary artery, whereas Sema3a protein inhibited the formation of EC strands. MAO detected that hypoxia was predominant in the aortic region and hypoxia downregulated the expression of Sema3a in culture. Results suggested that hypoxia in the aortic region downregulates the expression of Sema3a, thereby enhancing VEGF activity to induce the formation of CXCR4-positive EC strands, which are subsequently attracted into the *Cxcl12*-positive aortic wall. Published in J Mol Cell Cardiol 147:62–73, 2020. (COI:No)

### PP-216

### Histochemical assessment of $\alpha$SMA immunoreactive mesenchymal cells in the periodontal ligament of murine maxillary molar

Haruhi Maruoka[1,2], Tomoka Hasegawa[1], Ko Nakanishi[2,3], Kiichiro Inoue[1], Tomomaya Yamamoto[1,4], Yoshiaki Sato[2], Norio Amizuka[1] (*[1]Developmental Biology of Hard Tissue, Graduate School of Dental Medicine, Faculty of Dental Medicine, Hokkaido University, Japan, [2]Orthodontics, Graduate School of Dental Medicine, Faculty of Dental Medicine, Hokkaido University, Japan, [3]Biomaterials and Bioengineering, Graduate School of Dental Medicine, Faculty of Dental Medicine, Hokkaido University, Japan, [4]Northern Army Medical Unit, Camp Makomanai, Japan Ground Self-Defense Forces, Sapporo, Japan*)

Blood vessels and surrounding mesenchymal stem cells (MSCs) appear to play a pivotal role in remodeling and regeneration of periodontal ligaments (PDLs) during tooth development and tooth movement. To verify the chronological and spatial changing of blood vessels and MSCs in the PDLs, we have histochemically examined the PDLs of maxillary molars in the intact mice and tooth extraction model mice. In the PDLs of intact mice, endomucin-positive blood vessels were localized close to the alveolar bone. During the root development of intact mice, cells immunopositive for $\alpha$SMA, which is a hallmark of MSCs, were localized close to the endomucin-positive blood vessels in the apical region of the PDLs. The numbers of $\alpha$SMA-immunoreactive cells localized in the apical region were chronologically reduced after 3 weeks old mice. Thus, $\alpha$SMA-immunoreactive cells was hardly seen in the PDLs of 7-week-old intact mice. In contrast, the tooth extraction model mice exhibited abundant $\alpha$SMA-immunoreactive cells in the apical region of PDLs compared to the age-matched intact mice.
In conclusion, the population of $\alpha$SMA immunoreactive MSCs would be dynamically changed as mice grow and tooth movement. (COI:No)

### PP-217

### Examination of the usefulness of new antioxidants for frail associated with aging ~Aiming to extend healthy life expectancy~

Yoshihisa Koyama[1,2], Yuki Kobayashi[3], Hikaru Kobayashi[3], Shoichi Shimada[1,2] (*[1]Neuroscience and Cell Biology, Grad. Med., Osaka Univ., Osaka, Japan, [2]ARU., OPRC., OPMC., Osaka, Japan, [3]ISIR., Osaka Univ., Osaka, Japan*)

In Japan facing super-aging society, how to maintain the healthy life expectancy of the elderly is an important issue. Frail is a state of psychosomatic vulnerability due to a decrease in mental and physical vitality with aging. Because elderly is particularly susceptible to flail, it is considered that prevention of flail onset leads to extension of healthy life expectancy. Since reduction of *in vivo* antioxidant power is one of the factors causing frail, administration of antioxidants may lead to prevention of frail. Our Silicone-based agent (Si) made it possible to continuously generate a large amount of hydrogen in the intestinal tract by ingestion. Hydrogen is an antioxidant that selectively eliminate only the harmful hydroxyl radicals. Klotho-deficient mice exhibited various aging phenomena such as growth disorder, decreased activity and shortened lifespan. We investigated whether Si would be effective in preventing flail using Klotho-deficient mice and uncovered administration of Si significantly suppressed a reduction in the amount of spontaneous activity and behavioral performance associated with aging. Si will be effective preventive agents against flail and aging. (COI:No)

### PP-218

### Morphogenesis of pro to mesonephric vascular system in salamander, *Hynobius lichenatu* Micro vasculogenetic analyses with corrosive resin casts

Aki Murashima[1], Erina Saito[2], Sumio Isogai[1], Jiro Hitomi[1] (*[1]Dept. Anat., Iwate Med. Univ., [2]Dept. Neuroanat., Scho. Med., Hirosaki Univ*)

Inferior vena cava and its branches i.e. renal, adrenal, gonadal and ureteral veins vary in their origin, caliber, number and anatomical relationship to peripheral organs. To explain conspicuous variations in the inferior vena cava system of human, embryologists assumed embryonic cardinal veins (posterior-, sub- and supra-) in mammals. Using micro dye injection method, Ura phylogenetically verified these longitudinal embryonic veins in amphibian, reptile and mammalian embryos (1950s). However, the dye injection limits its usefulness for the analyses of glomerulus or portal sinusoid. Using micro resin casting method followed by SEM imaging (Isogai 1997), we have intended to reveal the vasculogenetic 3-D architectures within the pro, meso and metanephros. In this study, using salamander, we verified spatiotemporal formation of the glomus, sinusoid and portal posterior-cardinal vein in the pronephros, and the glomeruli, sinusoid and portal sub-cardinal vein in the mesonephros including parietal supra-cardinal vein. (COI:No)

### PP-219

### Mechanisms of the suppression of neuronal invasion into the marginal zone during layer formation of the mouse neocortex

Yuki Hirota[1], Rikaho Saito[1], Hitomi Sano[1], Kazunori Nakajima[1] (*[1]Dept. Anat. Keio. Univ. Sch. Med., Tokyo, Japan*)

During neocortical development, excitatory neurons radially migrate towards the pial surface and terminate their migration just beneath the marginal zone (MZ). Thus, suppression of neuronal invasion into the MZ is a crucial prerequisite for the formation of a fine laminar structure of the neocortex. We recently showed that Reelin receptor VLDLR is required for this process. Ectopic reelin overexpression in the *Vldlr*-deficient mouse cortex causes neuronal aggregation, but without an MZ-like cell-sparse central region that is formed when reelin is overexpressed in the normal cortex, raising a possibility that VLDLR suppresses the neuronal invasion into the MZ through regulation of molecules which function on the surface of neurons at the top of the cortical plate (CP). To identify the molecules that may function in these processes, we searched for the cell membrane proteins expressed in the neurons in the top of the CP using public single cell RNA-seq data. Among candidates, several cell membrane proteins localized to the dendrites of neurons in the top of the CP when exogenously expressed, suggesting their possible roles in the suppression of neuronal invasion in to the MZ. (COI:No)

### PP-220

### The early embryonic heart regenerates by compensation of proliferating residual cardiomyocytes after cryoinjury

Mayu Narematsu[1], Yuji Nakajima[1] (*[1]Grad. Sch. Med., Osaka City Univ*)

The adult mammalian heart is non-regenerative because cardiomyocytes do not re-enter the cell cycle after injury. To date, only a few literatures have been published on the embryonic heart regeneration, that it is uncertain whether the heart is capable of regenerating after cryoinjury. We established a cryoablation model in stage 16 chick embryonic hearts. In hearts at 5 hours post cryoinjury, cryoinjury-induced defects were approximately 200 μm in width in the primitive ventricle, thereafter the defect filled with mesenchymal cells accumulating between the epicardium and endocardium. IHC showed that there were no isl1-positive cells in either the scar tissue or residual cardiomyocytes. BrdU incorporation into residual cardiomyocytes was transiently downregulated in association with upregulation of p27, suggesting that cell-cycle arrest occurred at G1 phase immediately after cryoinjury. Estimated cell-cycle length was examined and the results showed that the shortest cell-cycle length at stage 19–23, and it increased with development due to elongation of the G2-M-G1 phase. Cryo-ablated defects in the early embryonic heart were restored by compensation by residual myocytes. (COI:No)

### PP-221

### On the species-specific traits of cervical motor neurons in chicken

Hiroshi Nagashima[1], Noboru Sato[1] (*[1]Division of Gross Anat Morphog., Niigata Univ. Grad. Sch. Med. Dent. Sci*)

Cervical spinal motor neurons in chickens show curious features in that they do not innervate infrahyoid muscles. Apparently, this morphological pattern is a secondary condition in terms of evolution, since the muscle receives the innervation in turtles and mammals. Furthermore, when the limb bud was implanted on the cervical portion in chicken embryos, the ectopic limb had muscles but not innervation from the cervical nerves. In chicken, these infrahyoid muscles and ectopic limb muscles were innervated by the hypoglossal nerve. Because ectopic limb on the interlimb portion was innervated by intercostal nerves, cervical nerves appear to lack spinal motor neurons which exist in hypoglossal and intercostal nerves. Molecular analysis, unfortunately, could not find the difference between the spinal motor neurons in the cervical, thoracic, and hypoglossal nerves. The experimental embryological study will be analyzed to unveil the peculiar feature of the cervical spinal motor neurons in chicken. (COI:No)

### PP-222

### Interstitial cell death in the developing plexus region in chick embryo

Ken-ichi Soma[1], Akina Chiba[1], Keisuke Watanabe[1], Hiroshi Nagashima[1], Noboru Sato[1] (*[1]Div Gross Anat, Niigata Univ*)

A large number of interstitial cells are known to undergo cell death (ICD) at the base of the hind limb when spinal nerves form the plexus and begin to enter the limb. Dying cells were observed at the nerve front, neighbor of the growth cone, but none of these cellular properties have been clear.

In the present study, we examined ICD at the nerve front of not only the lumbosacral level but also the cervical, the brachial, and the thoracic levels in chick by using TUNEL staining. Embryos were corrected from E3 to E6 when spinal nerves elongate to the base of limb buds to form the plexus. ICD was observed in forelimb bud as same as in hind limb bud. In contrast, there were few ICD in cervical and thoracic levels. Some dying cells expressed skeletal muscle precursor markers, Pax3 and Pax7. Introduction of GFP was observed in part of interstitial dying cells when primitive somites were labelled with GFP by *in ovo* electroporation. Further, transplantation of quail somites or somites from GFP transgenic chick resulted in the fact that part of dying cells were descended from transplanted cells. Together, ICD observed at the nerve front may derive in part from somites. (COI:No)

### PP-223

### Isolation and characterization of bone marrow-derived mesenchymal stem cells in *Xenopus laevis*

Rina Yamaguchi[1], Masaaki Kitada[2], Yasumasa Kuroda[1], Yoshihiro Kushida[1], Shohei Wakao[1], Mari Dezawa[1] ([1]*Department of Stem Cell Biology and Histology, Tohoku University Graduate School of Medicine,* [2]*Department of Anatomy, Faculty of Medicine Kansai Medical University*)

Mesenchymal stem cells (MSCs) are multipotent cells that exist in mesenchymal tissues such as bone marrow and are able to differentiate into osteocytes, chondrocytes, and adipocytes. MSCs are generally collected as adherent cells on a plastic dish, and are positive for markers such as CD44, CD73, and CD166, and negative for CD31. MSCs have been established from many kinds of mammals, but MSCs from amphibians have not yet been reported. We cultured adherent cells from the bone marrow of *Xenopus laevis* by modifying the protocol for culturing mammalian MSCs. The morphology of these cells was similar to that of mammalian MSCs. The amphibian MSCs were positive for amphibian CD44, CD73, and CD166, and negative for amphibian CD31. Moreover, they could be induced to differentiate into osteocyte-, chondrocyte-, and adipocyte-lineage cells by cytokine induction systems that were similar to those used for mammalian MSC differentiation. Thus, they are considered to be similar to mammalian MSCs. Unlike mammals, amphibians have high regenerative capacity. This work provides fundamental information for future studies to reveal the substantial differences between mammalian and amphibian MSCs. (COI:Properly Declared)

### PP-224

### Establishment of live imaging method in the developing mouse palatal shelves

Arata Nagasaka[1], Koji Sakiyama[1], Yasuhiko Bando[1], Yudai Ogasawara[2], Go Onozawa[3], Osamu Amano[1] ([1]*Div. Anat., Meikai Univ. Sch. Dent.,* [2]*Div. Second Oral Maxillofac. Surg, Meikai Univ. Sch. Dent.,* [3]*Div. First Oral Maxillofac. Surg, Meikai Univ. Sch. Dent*)

The palatal shelves first appear as a bilateral outgrowth of the palatal shelves on either side of the tongue. Subsequently, the palatal shelves elongate, elevate themselves into a horizontal position above the tongue, grow toward each other, and fuse at the midline to complete the formation. Perturbation in any of these steps can lead to cleft palate. In embryonic development, tissue morphogenesis requires the coordination of cell behaviors such as proliferation, differentiation and migration. During palate development, while mouse genetic approaches have been widely used to study, relevance of cell behaviors remains unknown. In this study, to directly observe cell behavior during palatal development, especially focusing on the palatal shelf elevation, we established confocal live imaging method using explant culture. We used E13 mouse embryo which occurs the palatal shelf elevation. Cross-sectional cultures of palatal shelves were microsurgically processed and embedded in a dish with collagen gel. For the observation, cells were visualized by staining dye. In this poster, we examined the suitable conditions of live imaging and observed the palatal elevation for 360 minutes. (COI:No)

### PP-225

### Effect of Sonic hedgehog on the gene expression of Wolffian duct

Kohei Johkura[1], Fengming Yue[1], Yali Men[1], Daihachiro Tomotsune[1] ([1]*Shinshu Univ. Sch. Med*)

[Background] The Wolffian duct (WD) and ureteric bud are known to secrete Sonic hedgehog (Shh), which facilitates the differentiation of surrounding mesenchyme through a paracrine manner. We assessed the effect of Shh on the gene expression of WD epithelial cells with a culture method.
[Materials and Methods] WD and its mesenchyme were isolated from rat mesonephros at embryonic day 13. Gene expression analysis of the isolated tissues was performed by real-time PCR. Matrigel-embedded culture of WD was maintained by FGF9 (50-125 ng/ml) and 10% BSA. Shh (1-10 μg/ml) was added to this culture, and the change in gene expression was evaluated at 24 h.
[Results] The gene expression of Shh was detected in the epithelium, but not in the mesenchyme, of WD, consistent with previous reports. When WD was cultured for 10 days with 125 ng/ml FGF9, Shh expression was maintained, suggesting its secretion to a certain extent in this condition. Addition of 10 μg/ml Shh to the WD culture maintained by FGF9 for 24 h significantly increased the gene expression of Ret, a receptor involved in kidney development.
[Conclusion] These findings suggest that an autocrine function of Shh may exist in WD. (COI:No)

### PP-226

### Gene expression patterns of *Gcm1* encoding chorion-specific transcription factor GCM1 in the extant actinopterygian fish, *Polypterus*

Takanori Shono[1], Masataka Okabe[1] ([1]*Dep Anat, Jikei Univ*)

*Gcm1* (Glial Cells Missing 1) gene encodes a transcription factor that is required for development of the trophoblast cells of chorion in mammals. *Gcm1* is a remarkable trigger for the placental evolution, however, when *Gcm1* expressing cells have been emerged during evolution of vertebrate is still unknown. We recently found that *Gcm1* locus is conserved in the genome of extant actinopterygian fish, *Polypterus*. This suggests that origin of *Gcm1* expressing cells for placental development can be traced back from early branched group of ray-finned fishes in vertebrates. We next investigated the gene expression of *Polypterus* by whole-mount *in situ* hybridization with *Gcm1* RNA probe. In the result, we found *Gcm1* is expressed scattered cells of external gills and yolk sac in the skin. These expression patterns suggest that *Gcm1* expressing cell of *Polypterus* may be ionocyte, is seen in most fishes to maintain their body fluid ionic and osmotic homeostasis. We also revealed by TEM that these cells contain characteristic large vacuoles in the cytoplasm. Further analyses such as mass spectrometry will be revealed the function of *Gcm1* expressing cells in *Polypterus*. (COI:No)

### PP-227

### Regulatory mechanism of morphogenetic cell movements via control of cell adhesion during zebrafish notochord formation

Masatake Kai[1] ([1]*Grad. Sch. Med., Osaka City Univ*)

Gastrulation, during which embryos establish basic body plans, is driven by a variety of collective cell migrations. For instance, during notochord formation in zebrafish, the cells in the axial mesoderm intercalate each other in a process known as convergence and extension (C&E). Thus, C&E involves neighbour exchange (NE) behaviour of cells. Recently, it was proposed that in order for the NE to occur, the cell-scale stress should exceed the supracellular stress within the tissue. Here, we focused on paraxial protocadherin (PAPC)/Pcdh8 as a key cell adhesion molecule in the notochord formation. PAPC is precisely regulated during gastrulation, such that its expression in the dorsal marginal zone is immediately suppressed specifically at the axial mesoderm, coinciding the onset of extensive C&E. We found that gain-of-function (GOF) of PAPC suppressed NE in the axial mesoderm. We inserted oil droplet in the axial mesoderm to visualize mechanical stresses in vivo. GOF of PAPC appeared to increase supracellular stress in the medial-lateral direction. We discuss the role of PAPC in orchestrating the cell-scale and supracellular stresses required for correct morphogenetic cell movements. (COI:No)

### PP-228

### The lncRNA *Malat1* regulates epithelial branching morphogenesis in fetal mouse submandibular gland

Toru Hayashi[1], Yuichi Kadoya[1] ([1]*Dept. of Anat. Sci., Sch. of Allied Health Sci., Kitasato Univ*)

Long non-coding RNAs (lncRNAs) are categorized as transcripts longer than 200-nucleotides without protein-coding region. The *Malat1* (metastasis-associated lung adenocarcinoma transcript 1) has been studied as one of the highly abundant lncRNAs in normal and cancer tissues. However, it remains poorly understood how *Malat1* regulates biological processes during organ development.
The fetal mouse submandibular gland (SMG) is a model organ for studying epithelial branching morphogenesis during development. We first investigated the developmental expression and spatio-temporal patterns of Malat1 in SMG. qPCR analysis showed that SMG at embryonic day 13 (E13) express *Malat1* transcript mainly in the mesenchyme. In addition, *Malat1* was detected at all tested developmental stages (E12-E17), showing highest relative expression levels at E15. Next, we repressed *Malat1* expression in the isolated epithelium at E13 with antisense LNA GapmeR that degrades complementary RNA in RNase-H-dependent manner. Treatment with GapmeR-*Malat1* resulted in an increase in the number and width of buds and in duct length, suggesting that *Malat1* expressed in E13 SMG epithelium regulates branching morphogenesis. (COI:No)

### PP-229

### Elucidation of the mechanisms underlying a novel Olig2 binding factor-mediated maintenance of neural and oligodendrocyte progenitor cells in central nervous system

Norihisa Bizen[1], Osim K Bepari[2], Masato Yano[1], Li Zhou[1,3,4], Manabu Abe[3,5], Kenji Sakimura[3,5], Katsuhiko Ono[6], Hirohide Takebayashi[1,4] (*[1]Div. of Neurobiol. and Anat., Grad. Sch. of Med. and Dent. Sci., Niigata Univ., [2]Dep. of Pharm. Sci., North South Univ., Dhaka, Bangladesh, [3]Dep. of Cell. Neurobiol., Brain Res. Inst., Niigata Univ., [4]CCRF, Niigata Univ., [5]Dep. of Animal Mod. Dev., Brain Res. Inst., Niigata Univ., [6]Dev. Neurobiol., Kyoto Prefect. Univ. of Med*)

Olig2 is indispensable for the fate specification of oligodendrocytes and motor neurons, and also involved in the proliferation and differentiation of several cell types, including neural progenitor cells (NPCs) and oligodendrocytes in CNS. However, it has remained unclear how Olig2 regulates multiple biological processes. Using yeast two hybrid screening, we identified a novel Olig2-binding factor, Obp2 which has been known to be involved in pre-mRNA splicing, translation, and transcriptional regulation. CNS-specific conditional knockout (*Nestin-Cre:Obp2* cKO) mice demonstrated drastic apoptosis and cell cycle arrest of NPCs and oligodendrocyte progenitor cells (OPCs) through activation of the p53 pathway in CNS. These results suggest that Obp2 is a key factor for the maintenance of NPCs and OPCs in the embryonic CNS. (COI:No)

### PP-230

### Reprograming of Muse cells, endogenous pluripotent stem cells, toward totipotent-like property

Kana Okawa[1], Yoshihiro Kushida[1], Shohei Wakao[1], Yasumasa Kuroda[1], Yasuhisa Matsui[2,3,4], Mari Dezawa[1] (*[1]Dept. Stem Cell Biology and Histology, Grad. sch. Med., Tohoku Univ., [2]Cell Resource Center for Biomedical Research, Institute of Development, Aging and Cancer [IDAC], Tohoku Univ., [3]Dept. Germ Cell Development, Grad. sch. Life Sciences, Tohoku Univ., [4]Grad. sch. Med., Tohoku Univ*)

Multilineage-differentiating Stress Enduring (Muse) cells are the pluripotent stem cell that exist in our body collectable as Stage-specific embryonic antigen 3 (SSEA-3) positive cells from the bone marrow, peripheral blood and connective tissue of every organ. Also, Muse cells show the ability of trilineage differentiation in vitro and in vitro without induction and self-renew. They have low safety concerns due to non-tumorigenicity for being endogenous. This study was performed to examine the differentiation ability into skeletal muscle cells-like cells (mesoderm) and β cells-like cells (endoderm) from human bone marrow derived-Muse cells and primordial germ cells-like cells (germ cell-lineage) from human umbilical cord derived-Muse cells after phagocytosing all these types of damaged/apoptotic mouse cells. Human Muse cells were supplied with debris of each apoptotic mouse cell types, cultured for 3 days, washed and cultured for another 2-3 weeks. Human Muse cells expressed genes relevant to each cell types. This study exhibits significance for the mechanism of stem cell development. (COI:Properly Declared)

### PP-231

### Toll-like receptor and scavenger receptor CD36 promote blastema cell proliferation during insect leg regeneration via macrophages

Tetsuya Bando[1], Misa Okumura[1], Hideyo Ohuchi[1] (*[1]Grad. Sch. Med., Okayama Univ*)

Hemimetabolous insects, such as the cricket *Gryllus bimaculatus*, can recover lost tissues, whereas regenerative abilitis in human are quite limited. When we amputate the cricket leg, a macrophage-mediated immune response is activated. To clarify the molecular link between immune response and tissue regeneration, we focused on the molecular function of innate immunity during leg regeneration. We found 11 Toll-like receptor (TLR) genes in cricket; expression of some TLR genes was upregulated during regeneration. RNAi against four TLR genes indicated regeneration-defective phenotype or small regenerates phenotype, mediated by decrease of cell proliferation. In these regenerating legs, expression of Jak/STAT signalling components and macrophage accumulation into the blastema were decreased. Macrophage-depleted crickets did not regenerate the lost parts of legs. CD36, which is a scavenger receptor in macriphages, was also required for leg regenetaion. These results suggest that innate immunity, mediated by TLR signalling and CD36 in macrophages, promotes leg regeneration. (COI:No)

### PP-232

### Elastin in the subapical area play important roles in the maintenance of neuroepithelial structure in developing cerebral cortex.

Tomoyasu Shinoda[1], Takaki Miyata[1] (*[1]Dept Anatomy and Cell Biology, Grad Sch Med, Nagoya Univ*)

Neural progenitor cells in developing mammalian cerebral cortex form epithelial structure with ventricular surface as 'apical' and pial surface as 'basal'. We previously showed that elastic property of the subapical space, a microzone ~10 μm from the apical surface, is essential for the nuclear migration of neural progenitor cells. However, the sources and the biological meaning of the elasticity still remains to be elucidated. Here, we found that Elastin, which is known to form an elastic extracellular protein complex called the elastic fiber, is highly responsible for the mechanical properties of developing cerebral wall. Elastin localized on the ventricular surface as well as in the space among densely-packed progenitor cells. Depletion of elastin by the treatment with elastase reduced contractility of the apical surface, followed to an increased nuclear density in the subapical space. These results suggest that elastin ensures structural and mechanical properties of developing cerebral wall by keeping elasticity in the subapical space and therefore contributes well-organized nucleaokinesis in the subapical space. (COI:No)

### PP-233

### Morphogenesis of vertebra in Japanese fire belly newt, *Cynops pyrrhogaster*

Shuichi Obata[1], Ryunosuke Yanaka[1] (*[1]Dept. Anatomical Sci., Sch. Allied Health Sci., Kitasato Univ*)

The vertebra is composed of 3 major parts (centrum, intervertebral disc, and vertebral arch). In many tetrapods including anuran amphibia, the centrum is formed by perichordal ossification, externally to the notochordal sheath. In fish, on the other hand, it is formed within the notochordal sheath. Morphogenesis of vertebra in urodelan amphibia has not been understood well. This study was addressed the morphogenesis and ossification of the vertebra in the Japanese newt, *Cynops pyrrhogaster*. Stages 30, 41, 51, 57, 59, and 60 embryos or larvae were studied. In stage 51 larva, notochordal sheath was clearly observed to be consisted of 3 layers under electron microscope. The ossification was not observed until the stage 51. In stage 59 larva, the cartilage was formed around the neural tube. Around the notochord, on the other hand, the cartilage was periodically formed along the anterior-posterior axis. The ossification was identified at the outermost area of the notochordal sheath along entire region of the notochord. These data suggest that the Japanese newt vertebra is formed by 2 different types of direct ossification (perichondral ossification and perichordal ossification). (COI:No)

### PP-234

### Analysis of limb bone patterns and lung alveolar cells in the *Fgf10* mosaic mutant embryos

Munenori Habuta[1], Akihiro Yasue[2], Ken-ichi Suzuki[3], Hirofumi Fujita[1], Keita Sato[1], Ayuko Takayama[3], Bando Tetsuya[1], Seiichi Oyadomari[4], Eiji Tanaka[2], Hideyo Ohuchi[1] (*[1]Department of Cytology and Histology, Okayama University Graduate School of Medicine, Dentistry and Pharmaceutical Sciences, Okayama, Japan., [2]Department of Orthodontics and Dentofacial Orthopedics, Institute of Biomedical Sciences, Tokushima University Graduate School, Tokushima, Japan., [3]Center for the Development of New Model Organisms, National Institute for Basic Biology, Aichi, Japan., [4]Division of Molecular Biology, Institute for Genome Research, Tokushima University, Tokushima, Japan*)

Fibroblast growth factor 10 (Fgf10) is an extracellular signaling molecule required for limb and lung formation. Studies on *Fgf10* heterozygous and hypomorphic mutants have shown *Fgf10* has dose-dependent functions to permit various aspects of organogenesis. However, the exact amount of Fgf10 for the formation of each limb element and other organ component remains elusive. Here we focused on *Fgf10* genome-edited founder (F0) mice generated by CRISPR-Cas9 system. The F0 mice are mosaic having cells of normal and mutant genotypes within the same individual and divided into three types, no limbs (I), limb defects (II) and normal limbs (III). By deep sequencing, we estimated percentage of putative functional Fgf10 (PFF) as 25.3±2.7% (mean and SEM) in the type II embryos. Skeletal staining showed typical type II limbs were truncated at the stylopod or zeugopod level. Histological analysis revealed the accessory lobe was firstly lost in the type II embryos. In the E18 type III lungs, the number of alveolar type 2 epithelial cells decreased as the PFF reduced. These results suggest the amount of Fgf10 is critical to define the number of differentiated cells and organ structures. (COI:No)

### PP-235

### Expression of calcitonin gene-related peptide (CGRP) and glutamate receptor, ionotropic, kainate 1 (GRIK1) mRNAs in the forming embryonic and postnatal maxillary molar in mice

Masataka Sunohara[1], Naomi Asada[1], Kouhei Kawata[1], Yuuki Maeda[2], Yoko Miwa[1], Yoshiaki Ide[1], Kingo Suzuki[1] (*[1]Dept. of Anatomy, Sch. Life Science Dentistry, NDU, Tokyo, JAPAN, [2]General Dentistry2, NDU Hospital*)

Calcitonin gene-related peptide (CGRP) is a well-characterized neuropeptide functioning as a neurotransmitter. Glutamate receptor, ionotropic, kainate 1 (GRIK1) has also been reported to generate high-affinity kainate receptors and to play an important role in synaptic potentiation. In this study, we aimed at the clarification of roles of CGRP and GRIK1 in tooth development, and attempted to analyze the expression of CGRP and GRIK1 mRNA in the mouse maxillary molar during development from the embryonic stage (E18.5) to after birth (P10, P15, and P20), by using *in situ* hybridization method. We found that CGRP mRNA was highly expressed in the maxillary molar at E18.5 as compared to at postnatal stages. GRIK1 mRNA, on the other hand, was detected at P10 and P15. It was of note that the signal of GRIK1 mRNA but not that of CGRP at postnatal stages was localized in the basal region of the dental pulp of molars. The cells expressing CGRP and GRIK1 mRNAs in the dental pulp decreased from E18.5 and disappeared at P20. The gene expression profiles of CGRP and GRIK1 may indicate that they have roles in tooth development at stages of which root formation and erupted movements may occur. (COI:No)

### PP-236

### Target gene analysis of transcriptional regulator Sox9 in retinal progenitor cells of mouse

Norihiro Sudou[1], Hiroki Fujieda[1] (*[1]Dept.of Anatomy,Sch.of Med., Tokyo Women's Med. Univ., Tokyo,Japan*)

The transcriptional regulator Sox9 is continuously expressed in retinal progenitor cells and the last differentiated Muller cells, and is considered to be a key regulator of the transcriptional control network of retinal formation. The sox9 gene is known as a factor necessary for maintaining the characteristics of retinal progenitor cells during retinal development, and has also been shown to be necessary for maintaining the characteristics of Muller cells (many reports). However, the target gene is not well understood.

In this study, we will clarify the target gene of the sox9 gene expressed in retinal progenitor cells of postnatal day1 mouse retina by ChIP-seq analysis. The results of these studies are expected to enable comparison with the target gene of the sox9 gene expressed in Muller cells in the adult retina, and to provide insight into the target gene transition of transcription factors. (COI:No)

### PP-237

### Forced expression of *Dlx5* in mouse neural crest cells reveals differentiation potential of bone and cartilage in early apical cranial mesenchyme

Masaki Takechi[1], Tri Vu Hoang[1], Miki Shimizu[2], Taro Kitazawa[2], Hiroki Higashiyama[2], Akiyasu Iwase[2], Hiroki Kurihara[2], Sachiko Iseki[1] (*[1]Dept. Mol. Craniofac. Embryol., Grad. Sch. Med. Dent. Sci., Tokyo Med. Dent. Univ., [2]Dept. Physiol. Chemi. Metabol., Grad. Sch. Med., The Univ. of Tokyo*)

Neural crest cells (NCCs) give rise to various tissues such as the pigment cell, bone and cartilage in the head. *Distal-less homeobox 5* (*Dlx5*) is involved in differentiation of NCC-derivatives in craniofacial development. In this study, we examined the differentiation potential of apical head mesenchyme by *Dlx5*-augumentation in the mouse NCC ($NCC^{Dlx5}$). In $NCC^{Dlx5}$ mice, ectopic cartilage (ec) and heterotopic bone (hb) were generated in different layers at the cranial vertex. The ec and hb were derived from the non-skeletogenic cell population in the apical head area (the early migrating mesenchyme: EMM). The ec developed within *Foxc1*$^{+}$-dura mater with increased Pdgfr$\alpha$ signaling, and the hb formed with enhanced Bmp and Wnt/b-catenin signaling in *Dermo1*$^{+}$-dermal layer from E11.5. Taken together with previous studies, we propose that in normal situation the EMM is committed to non-skeletogenic cell, and then it is divided into dermis and meninges by E11.5. Two distinct responses of the EMM, chondrogenesis and osteogenesis, to *Dlx5*-augmentation in the $NCC^{Dlx5}$ support this idea. (COI:No)

### PP-238

### Optical detection of neuronal activity in the olfactory-limbic network of the embryonic chick forebrain

Katsushige Sato[1], Yoko Sato[2] (*[1]Department of Health and Nutrition Sciences, Faculty of Human Health, Komazawa Women's University, Japan, [2]Department of Nutrition and Dietetics, College of Nutrition, Kanto Gakuin University*)

We have applied 1020-site optical recording with a voltage-sensitive dye to the embryonic chick olfactory system. In our previous study, we detected neural activity evoked by olfactory nerve (N.I) stimulation in the olfactory bulb (OB), and showed that (1) optical responses in the OB consisted of three components, viz., a fast spike-like signal (corresponding to the action potential), a delayed long-lasting slow signal (corresponding to the EPSP), and an oscillatory activity, (2) synaptic function in the OB emarged at arouud the embryonic 6-day (E6) stage, and (3) the oscillatory activity was observed from the E9 stage. In this study, we persued functional development of the olfactory-limbic system in the forebrain. We found that neural responses evoked by N.I stimulation spread into the forebrain from the E9 stage in normal physiological solution and the E8 stage in the $Mg^{2+}$-free solution. At the E10 stage, the responses were widely detected in the forebrain. Compared with anatomical data, the response area in the forebrain was considered to correspond to the limbic system. We also examined pharmacological profiles of the optical siganls in the olfactory-limbic network. (COI:No)

### PP-239

### Verification of changes over time of oxidative stress and antioxidant capacity in rats during moderate exercise.

Ohno Yoichi[1], Noriaki Shimokawa[2], Noriyuki Koibuchi[3] (*[1]Dept of Physical therapy, Faculty of Health care, Takasaki University of Health and Welfare, [2]Dept of Nutrition, Faculty of Health and Welfare, Takasaki University of Health and Welfare, [3]Dept of Integrative Physiology, Gunma University Graduate School of Medicine*)

**Background:** Although the maintenance of oxidation-antioxidant equilibrium is extremely important to the survival and health of the organism, our knowledge of the physiological underpinnings of these mechanisms is insufficient. In this study, we verified the changes in oxidative stress and antioxidant capacity over time in rats loaded to moderate exercise.

**Method:** Male Wistar rats were grouped into exercise group (n=4) and control group (n=3). In the exercise group, moderate treadmill exercise was carried out for 8 weeks. The amount of active oxygen and free radicals in serum (d-ROMs test) and antioxidant capacity (BAP test) were examined. The measurement was carried out every week for 8 weeks.

**Results:** There was no significant difference in oxidative stress between the exercise group and the control group. However, there was a tendency to show low values in the exercise group. Antioxidant capacity tended to be lower in the exercise group than in the control group. In addition, the results were significantly lower at the 1st, 2nd, 4th, 5th, and 7th weeks.

**Conclusion:** A decrease in oxidative stress due to exercise may induce the reduction of antioxidant capacity. (COI:No)

### PP-241

### Transcriptional profiles of the rat pulmonary vein during a perinatal period

Daiki Seya[1], Toru Akaike[1], Susumu Minamisawa[1] (*[1]Dept. of Cell Physiology, The Jikei University School of Medicine, Tokyo, JAPAN*)

Pulmonary veins (PV) are unique low-pressure vessels that are exposed to the highest oxygenated blood in the body and its hemodynamics dramatically changes before and after birth. However, the molecular mechanisms that contribute to the development and maintenance of PV-specific functions and structures during a perinatal period remained largely unknown. We examined the transcriptional profiles of PV and inferior vena cava (IVC) of late-fetal and neonatal Wistar rats by microarray analysis (SurePrint G3 Rat). We found that 123 genes were up-regulated and 233 genes were down-regulated in PV before and after birth. Compared to IVC, 1497 genes were up-regulated and 1032 genes were down-regulated in fetal PV, and 863 genes were up-regulated and 536 genes were down-regulated in neonatal PV. Pathway analysis revealed that genes related to the PPAR signaling were altered expression in the PV before and after birth, and genes related to serotonergic and cholinergic synapses were altered expression in the PV compared to the IVC. Future analyses may provide further insights into the molecular mechanisms for the unique functions of PV. (COI:No)

### PP-242

### Gli1-positive cells contribute to the alveolar bone formation during orthodontic tooth movement

Yuri Seki[1,2], Hiroaki Takebe[1], Toshihide Mizoguchi[3], Kazuharu Irie[4], Masahiro Iijima[2], Akihiro Hosoya[1] ([1]*Dept. Histol. Health Sci. Univ. Hokkaido, Japan,* [2]*Dept. Orthodont. Health Sci. Univ. Hokkaido, Japan,* [3]*Oral. Health Sci. Cent. Tokyo Dent. Coll., Tokyo, Japan,* [4]*Dept. Anat. Health Sci. Univ. Hokkaido, Japan*)

Although orthodontic tooth movement induces bone formation at the tension side of alveolar bone, the mechanism of osteoblast differentiation is controversial. Gli1 is an essential transcription factor of hedgehog signaling and functions in undifferentiated cells during embryogenesis. In this study, we investigated the differentiation of Gli1-positive cells in periodontal ligament during tooth movement. In iGli1/Tomato mice just after the final administration of Tamoxifen, Gli1/Tomato-positive cells were observed near the blood vessels in the periodontal ligament. Next, to move the first molar of iGli1/Tomato mice medially, closed-coil springs were attached between the maxillary first molar and incisors. After 10 days, the first molar has been moved medially, and a large number of osteoblasts aligned on the distal surface of alveolar bone. Numerous Gli1/Tomato-positive cells were observed at the distal side of the periodontal ligament. Some of these cells were immunopositive for Osterix, a marker of osteoblasts. Therefore, these results suggest that Gli1-positive cells in the periodontal ligament proliferate and differentiate into osteoblasts during orthodontic tooth movement. (COI:No)

### PP-243

### The decrease in Glycosaminoglycan (GAG) of articular cartilage (AC) with aging at the side of protrusion and fossa in same joints

Mikiko Kobayashi-Miura[1], Harumi Osago[1], Aoi Miyaji[1], Mineyoshi Hiyoshi[1], Nobumasa Hara[1], Mikako Tsuchiya[1] ([1]*Biochem. Med. Shimane Univ*)

Among the elderly, one of the most common diseases, osteoarthritis (OA), is caused by degeneration of AC. The large part of AC is consisted of mainly GAG and collagen (COL). It is known that the amount of GAG decreases with aging. Previously, we indicated that the decrease in the amount of GAG with aging is greater in femur (protrusion (凸)) than in tibia (fossa (凹)) ACs even in the same knee joint of male rat. It is little known whether the decrease in the GAG in other joints are different between protrusion side and fossa side, whether their changes are different between male and female.

We aimed to examine decrease in main components with aging in various ACs, and to compare them between male and female. Using young (7-8-week-old) and aged (over 1-year-old) adult rats, we collected ACs from shoulder (humerus, scapula), hip (femur, ischial), and knee (femur, tibia) joints. Then, we quantified GAG and an indicator of COL, hydroxyproline (Hyp).

The amount of GAG in most ACs decreased with aging. Relative decreasing ratio of the protrusion side tended to be larger than that of the fossa side within each joint. The ratio of each AC was greater in female than in male. (COI:No)

### PP-244

### Evaluation of cortical bone density using a clinical CT scanner images; detection of cortical porosity areas in the femoral diaphysis

Ayami Hamamoto[1], Keiko Takamura[1], Kazunobu Saiki[1], Kiyohito Murai[1], Daisuke Endo[1], Takeshi Imamura[1], Keishi Okamoto[1], Toshiyuki Tsurumoto[1] ([1]*Department of Macroscopic Anatomy,Nagasaki University Graduate School of Biomedical Sciences*)

It has been reported that in osteoporotic cortical long bone, under the microscope, the size of the central canal of bone increases, leading to cortical porosity and trabeculation, and eventually to enlargement of the medullary cavity. We have devised a method to detect low bone density areas in the cortical bone of the long bones quantitatively using a clinical CT scanner. The "Cortical bone quality index (CQI)" was defined by comparing the mean Hounsfield Unit (HU) with the values of each pixel of the cortical bone areas in the cross-sectional image. Analysis of the distribution of CQI in the cross-section of the bone showed similarity between the histological and CT images. Therefore, it seemed that the cortical bone porosity areas could be evaluated. CT images of 46 male femoral bones of known age at death were obtained, and the values of CQI of these bones were calculated and analyzed with the HU matrixes. Our previous study reported that the reduction in cortical bone area ratio in the femoral diaphysis was smaller in males than in females. This study indicated that even in males, cortical bone areas with reduced bone density increased significantly with age. (COI:No)

### PP-245

### Agenesis of auditory bullae in hairless dogs with *FOXI3* haploinsufficiency

Kazuhiro Koyasu[1], Yayoi Ikeda[1] ([1]*Dept. Anat., Sch. Dent., Aichi Gakuin Univ., Nagoya Japan*)

Hairless dogs with *FOXI3* haploinsufficiency show a form of ectodermal dysplasia characterized by a lack of hair and abnormal tooth morphology. We found malformation of auditory bulla in these dog skull specimens. We examined 23 adult hairless dogs, two adult beagles, one adult mongrel dog, and one juvenile mongrel dog. All the skull specimens belong to the AGU Dental Science Museum. The parts of auditory bullae and ossicle bones were analyzed using μCT imaging system. 9 out of 22 (40.9%) hairless dog specimens (one specimen was excluded because of having an artificially damaged bulla) have malformed auditory bullae. The floors of the tympanic cavity were incompletely formed in these specimens. This time, we observed malleus and incus among the occiles bones. Our specimens of hairless dogs were offspring of original Mexican hairless dog and beagles (Fukuta et al. 1991). To date, one case malformation of external and middle ears has been reported in a Peruvian hairless dog (Tassano et al. 2015). Here, we report for the first time bone malformation at the auditory bullae in all the kind of hairless dogs. (COI:No)

### PP-246

### Enhanced expression of FABP4 in septoclasts in FABP5-deficient mouse tibiae.

Yasuhiko Bando[1], Nobuko Tokuda[2], Go Onozawa[1], Yudai Ogasawara[1], Arata Nagasaka[1], Koji Sakiyama[1], Yuji Owada[3], Osamu Amano[1] ([1]*Meikai Univ. Sch. Dent.,* [2]*Sch. Med. Dokkyo Medical Univ.,* [3]*Grad. Sch. Med. Tohoku Univ*)

We previously reported that exclusive expression of epidermal-type fatty acid-binding protein (FABP5) in septoclasts of the epiphyseal plate of mice. In this study, we investigated localization of adipocyte-type FABP (FABP4) and peroxisome proliferator-activated receptor (PPAR) g in septoclasts of FABP5-deficient or PPARg agonist (GW1929)-treated mice.

Frozen sections of the epiphyseal plate were obtained from the proximal tibiae of FABP5-deficient mice, GW1929-treated mice and wild-type C57BL/6 mice. Immunohistochemical staining for FABP4 and double immunofluorescent staining for FABP4 and septoclast markers or PPARg were performed.

Number of FABP4-immunoreactive septoclasts of FABP5-deficient or GW1929-treated mice was significantly increased compared to those of controls. Imunoreacitivity for PPARg was detected in the nuclei of FABP4-positive septoclasts of FABP5-deficient or GW1929-treated mice, although no expression of PPARg was detected in FABP4-positive septoclasts of control mice.

Our results suggest that the functional exertion of FABP5 in septoclasts is compensated by enhanced expression of FABP4 and occurrence of PPARg in septoclasts of FABP5-deficient mice. (COI:No)

### PP-247

### Bone regeneration with a novel bone filling material containing tricalcium phosphate (TCP) and pulledulan phosphate (PPL).

Yasuhito Morimoto[1], Tomoka Hasegawa[1], Tomomaya Yamamoto[5], Hiromi Hongo[1], Keisuke Kubota[3], Yasuhiro Yoshida[4], Tsutomu Sugaya[2], Norio Amizuka[1] ([1]*Developmental Biology of Hard Tissue Faculty of Dental Medicine/Graduate School of Dental Medicine Hokkaido University,* [2]*Periodontology and Endodontology,* [3]*Oral Functional Prosthodontics,* [4]*Biomaterials and Bioengineering,* [5]*Northern Army Medical Unit, Camp Makomanai*)

Phosphorylated pullulan (PPL) is a novel polysaccharide (a polymer of glucose) with abundant phosphate residues. We have histochemically examined the new bone induced by a combined material of PPL and TCPs grafted in rat tibiae.

At the region of bone defects, TRAP-reactive/cathepsin K-positive osteoclasts and ALP-positive osteoblasts were localized on the both $\beta$-TCP granules and PPL after 1- or 2-weeks. In addition, non-collagenous bone protein such as osteopontin and DMP1 accumulated on the surface of $\beta$-TCP granules and PPL. At 4 weeks, new bones were formed on the grafted $\beta$-TCP granules and directly on the PPL. Interestingly, the grafted PPL allowed the invasion of vascular endothelial cells and osteoblastic cells inside, and also showed calcium phosphate deposition on the superficial layer when estimated by EPMA and von Kossa staining.

Taken together, it seems likely that PPL could effectively serve as an adequate scaffold for bone regeneration due to the accumulation of calcium and phosphates on the superficial layer, therefore indicating that the combined components with PPL and TCP could provide a favorable microenvironment for bone regeneration. (COI:No)

### PP-248

### Lineage-tracing analysis of Gli1-positive cells in periodontal ligament after tooth extraction

Saki Fujii[1], Hiroaki Takebe[2], Toshihide Mizoguchi[3], Tsuyoshi Shimo[1], Akihiro Hosoya[2] ([1]*Division of Reconstructive Surgery for Oral and Maxillofacial Region, Department of Human Biology and Pathophysiology, School of Dentistry, Health Sciences University of Hokkaido, Ishikari-Tobetsu, Hokkaido 061-0293, Japan,* [2]*Division of Histology, Department of Oral growth and development, School of Dentistry, Health Sciences University of Hokkaido, Ishikari-Tobetsu, Hokkaido 061-0293, Japan,* [3]*Oral Health Science Center, Tokyo Dental College*)

During the tissue repair process after tooth extraction, osteoblasts appear in the tooth socket and form alveolar bone. However, the source of these osteoblasts is still uncertain. Gli1, a downstream factor of Shh signaling, is known to exhibit stem cell properties during tooth development. Therefore, in this study, we investigated the localization of Gli1-positive cells and their progeny cells after tooth extraction using iGli1/Tomato mice possessing the Gli1-Cre$^{ERT2}$;Rosa26-loxP-stop-loxP-tdTomato gene. After 2 days of tamoxifen administration to iGli1/Tomato mice, Gli1/Tomato-positive cells were rarely observed in the periodontal ligament of the upper second molars. Although many cells existed in the tooth socket at 1 day after tooth extraction, there was no signal of Tomato-fluorescence. At 7 days, numerous Gli1/Tomato-positive cells were found in the tooth socket harboring proliferating cell nuclear antigen-positive cells. After 14 days, osterix-positive osteoblasts formed new alveolar bone in the tooth socket. These results suggest that Gli1-positive cells in the periodontal ligament proliferate after tooth extraction and might contribute to socket healing. (COI:No)

### PP-249

### The innervation of the tilapia pharyngeal jaw bones with special reference to the central projections

Kosuke Imura[1], Akihito Takeda[1], Masato Endo[2], Kengo Funakoshi[1] ([1]*Dept. Neuroanat. Yokohama City Univ.,* [2]*Dept.Marine Biosci. Tokyo Univ. of Marine Sci. Tech*)

Some studies have discussed that the nervous system contributes to teleost bone remodeling (Wada et al., 2013; Suarez-Bregua et al., 2017). However, the neuroanatomical basis of central connections for bone remodeling is not clear. We have investigated the innervation of a cichlid, *Oreochromis niloticus*, pharyngeal jaw bones and its central projections by means of stereomicroscopic observations and neural tract tracing respectively. Peripheral nerve innervating the superior pharyngeal jaw bone (sPJ) was identified as branches of the vagal nerve. We applied a neural tracer, DiI crystal, to the small branch of vagal nerve. The labeled fibers connected to vagal ganglion neurons. In turn, the labeled neurons projected to vagal lobe layers with small beaded terminals. In this report, we investigated the innervation of the inferior pharyngeal jaw (iPJ). The peripheral innervation was similar to the sPJ innervation. The labeled vagal fibers were traced dorsally running into vagal ganglion. We will discuss central projections from the iPJ with the projection patterns from the sPJ. (COI:No)

### PP-250

### Effects of aging and balance exercise on the knee joint of the senescence accelerated mouse (SAMP8)

Kosuke Norimatsu[1], Kazuki Nakanishi[1], Akira Tani[1], Keita Fukumaru[1], Shotaro Otsuka[2], Seiya Takada[2], Harutoshi Sakakima[3] ([1]*Department of Health Sciences, Kagoshima University Graduate School of Health Sciences,* [2]*Department of Systems Biology in Thromboregulation, Kagoshima University Graduate School of Medical and Dental Science,* [3]*Department of Physical Therapy, School of Health Sciences, Faculty of Medicine, Kagoshima University*)

We investigated the osteoarthritis (OA)-related changes and effects of balance exercise program on the knee joint of a SAMP8 mice. The mice were evaluated the motor functions and histological changes of knee joint aged of 1, 3, 5, 7, and 9-months. The exercise mice were required to run on a motorized rotarod treadmill from aged 7 to 9-months. The articular cartilage, synovium, and osteophyte formation were observed, and OA development were quantified by histological grading score. The mice were significantly decreased the knee joint angle and increased the lateral diameter of the knee at 9-months old. The cartilage destruction and osteophyte formation were increased with age. Notably, severe OA-related change was observed at 9-months old. Deterioration of the histological grading score was increased from 5-months old. Although the exercise mice improved motor functions, the OA-related change did not prevent by exercise. Our findings showed that SAMP8 mice is useful as a mouse model that spontaneously developing severe OA of knee joint with age. Balance exercise for a knee joint which are already observed in OA changes may contribute progression of OA. (COI:No)

### PP-251

### Reduced cortical bone thickness increases stress and strain in the female femoral diaphysis analyzed by a CT-based finite element method: Implications for fatigue fracture of the femur

Daisuke Endo[1], Keiko Ogami-Takamura[1,2], Takeshi Imamura[1], Kazunobu Saiki[1], Kiyohito Murai[1], Keishi Okamoto[1], Toshiyuki Tsurumoto[1,2] ([1]*Dept. Macroscopic Anat. Grad. Sch. Biomed. Sci. Nagasaki Univ.,* [2]*Center of Cadaver Surgical Training, Nagasaki Univ*)

The incidence of hip fractures is increasing and high among women older than 70 years. Age-related decreases in the morphological parameters of the femoral diaphysis, such as cortical bone thickness, were reported in Japanese women. Thus, the relationships between biomechanical and morphological parameters were analyzed using a CT-based finite element method.

Finite element models were constructed from 44 femurs of Japanese women aged 31-87 years using CT data. Loading conditions were set as the single-leg configuration.

All types of stresses and minimum principal strain in the femoral diaphysis scored higher absolute values in the high-risk group (≥70 years, n=28) than in the low-risk group (<70 years, n=16) ($p<0.05$). All biomechanical parameters strongly correlated with the reciprocal of cortical bone thickness.

These results demonstrated that biomechanical parameters may be predicted by calculating the cortical bone thickness of femurs. The present results will promote further investigations on the contribution of morphological parameters to the onset of atypical femoral fracture. (COI:No)

### PP-252

### Histological evaluation of bone regeneration in the vicinity of bone implant using the bone substitute mixed with phosphorylated pullulan (PPL) and beta-tricalcium phosphate (β-TCP)

Keisuke Kubota[1,2], Tomoka Hasegawa[1], Tomomaya Yamamoto[1,6], Hiromi Hongo[1], Yasuhito Morimoto[1,3], Miki Abe[1], Haruhi Maruoka[1,4], Yasuhiro Yoshida[5], Atsuro Yokoyama[2], Norio Amizuka[1] ([1]*Department of Developmental Biology of Hard Tissue, Faculty of Dental Medicine/Graduate School of Dental Medicine, Hokkaido University,* [2]*Department of Oral Functional Prosthodontics, Faculty of Dental Medicine/Graduate School of Dental Medicine Hokkaido University,* [3]*Department of Periodontology and Endodontology, Faculty of Dental Medicine/Graduate School of Dental Medicine Hokkaido University,* [4]*Department of Orthodontics, Faculty of Dental Medicine/Graduate School of Dental Medicine Hokkaido University,* [5]*Department of Biomaterials and Bioengineering, Faculty of Dental Medicine/Graduate School of Dental Medicine Hokkaido University,* [6]*Northern Army Medical Unit, Camp Makomanai, Japan Ground Self-Defense Forces*)

For the bone implant treatment, maintaining bone volume and trabecular connectivity around the implant is important. In order to evaluate the effect of a novel bone substitute composed of PPL and β-TCP for bone regeneration, the implant with vehicle (control), PPL, β-TCP and PPL+β-TCP were placed in the bone cavity, and then, the regenerated bone tissue around the implant were examined at 1,2, and 4 weeks after the surgery.

Newly formed trabeculae were observed around the implant in all groups at 1 week after surgery. However, the bone volume and trabecular connectivity of new bone were chronologically reduced in control and β-TCP groups, and consequently, the areas of implant surface covered by regenerated bone has been reduced after 4 weeks. In contrast, in PPL+β-TCP groups, new bone was abundantly formed, being associated with PPL and β-TCP. The bone volume and trabecular connectivity appeared to be increased in PPL and PPL+β-TCP groups after 2- and 4-weeks surgery, when compared with control and β-TCP groups. Taken together, it seems likely that PPL would play a pivotal role in maintaining the volume of regenerated bone and the trabecular connectivity around the implant. (COI:No)

### PP-253

### Effect of transcutaneous electrical stimulation on structural changes in tibial tuberosity caused by treadmill-downhill- running in rats

Hirai Suito[1], Xueqian Zeng[1], Wataru Minamizono[1], Sayumi Iwamoto[2], Tetsuro Suzuki[2], Masafumi Ohsako[2] ([1]*Grad. Sch. Life Design, Toyo Univ.,* [2]*Human Life Design, Toyo Univ*)

[Purposes] A purpose of this study was to investigate an effect of transcutaneous electrical stimulation (TES) on the structural changes in a tibial tuberosity caused by a treadmill-downhill-running (TDR) using rat.

[Materials and methods] Twenty-four male rats (wistar strain, 7-week-old) were used as materials. They were divided into an exercise group (EX) and a control group(CO), and furthermore, EX was subdivided into a running group(RE) and a running and TES group (TE). RE and TE performed a TDL exercise of a tilt angle of -15° and a speed of 17 m / min, for 1 hour / day, 5 days / week for 3 weeks. Moreover, TE was electrically stimulated for 10minuites after every TDR. Conditions of the electrical stimulations were a direct current using a carrier wave(80kHz).

[Results] There were more fibers in the superficial layer in RE and TE than in CO. Many chondrocytes in the deep layer in RE were larger than CO and TE. Furthermore, many interleukin-1β (IL-1β)-positive-cells were observed in RE but was rarely confirmed in CO and TE.

[Conclusion] It was understood that TES suppressed structural changes and inflammation of the tibial tuberosity caused by descending movement. (COI:No)

### PP-254

### Expression and function of GABA receptor rho2 in osteoclast differentiation

Yoshihiro Tamamura[1], Reiko Kido[1], Eisuke Shimokita[1], Yoshihiro Tsuruo[1]
(*[1]Tokushima University Graduate School of Biomedical Sciences Department of Anatomy and Cell Biology*)

GABA receptor rho2 (Gabrr2) is a subunit of GABAc receptor that functions as a chloride ion channel, however its expression and function in osteoclast differentiation are largely unknown. In this study, we examined the expression and function of Gabrr2 by immunohistochemistry and in vitro experiments using bone marrow-derived primary osteoclasts. Gabrr2 expression was colocalized with the expression of Cx3cr1 or Flt-3, a macrophage/preosteoclast marker, in adult mouse bone marrow. Retroviral overexpression of Gabrr2 or treatment with GABAc receptor agonist TACA resulted in the reduction of TRAP staining and the decreased expression of osteoclast differentiation marker gene such as Nfatc1 and Cathepsin K (Ctsk). In contrast, expression of shRNA against Gabrr2 (shGabrr2) enhanced TRAP staining or CtsK expression. Moreover, upregulation of Nfatc1 by shGabrr2 was suppressed by the treatment with calcineurin inhibitor FK506. These results indicate that Gabrr2 has a inhibitory role for osteoclast differentiation by inhibiting the activity of calcineurin. We are currently investigating the relationship between intracellular concentration of chloride and calcineurin activity. (COI:No)

### PP-255

### Study in effect of transcutaneous electrical stimulation of the different frequency on bone structure in hindlimb-suspended rats

Wataru Minamizono[1], Hirai Suitou[1], Xueqian Zeng[1], Sayumi Iwamoto[2], Tetsurou Suzuki[2], Masafumi Ohsako[2] (*[1]Cra.Sch.Human Life Design, Toyo univ., [2]Human Life Design, Toyo Univ*)

[Aims] This study was aimed to morphologically investigate the effects of transcutaneous electrical stimulation(TES) of different frequency on a bone structural changes caused by a hindlimb-suspension (HS) in rats.
[Materials and methods] Forty-eight male rats ( 7-week-old) were used as materials and were divided into a HS group: HS, a HS and TES group: TE and a control group: CO. Moreover, TE was subdivided into a once (TE1), three times (TE3) and five times (TE5) per week groups, due to differences in intervention frequency. HS and TE were hindlimb-suspended in the cage for two weeks. In TE, the TES using the carrier wave was performed 10min/day, 5days/week for two weeks. Femur was excised from each group, the bone strength was measured and the histological structure was observed. Calcein and alizarin were administered to each group 7 and 3days before sampling, respectively.
[Results] In HS and TE, the bone strength showed significant lower values than CO, but that of TE5 was significantly higher than HS.
[Conclusion] It was suggested that bone loss in the cancellous bone caused by hindlimb-suspension of rat and was suppressed by TES three or more times a week. (COI:No)

### PP-256

### Effect of electrical stimulation on structural changes in tibial articular cartilage with tail-suspension in rats

Xueqian Zeng[1], Hirai Suito[1], Wataru Minamizono[1], Tetsuro Suzuki[2], Sayumi Iwamoto[2], Masafumi Ohsako[2] (*[1]Grad.Sch.Human Life Design, Toyo Univ., [2]Human Life Design, Toyo Univ*)

[Purpose] This study was aimed to morphologically investigate the effect of electrical stimulation on the structural changes in the tibial articular cartilage of tail-suspended rats.
[Material and methods] Seventy-two male rats (wistar strain, 7-week-old) were used as materials, they were divided randomly into three groups: tail-suspended group(TS), electrical stimulation group(VP) and control group(CO). The experimental period of each group was 1, 2, or 3 weeks. TS and VP were tail-suspended for each experimental period, and furthermore, VP was electrically stimulated for each period using a vector potential generator. The electrical stimulation was performed , 30 minutes / day, 5 days / week.
[Results] In TS, a reduction of proteoglycan and an elevation of the tide-mark were observed in the tibial articular cartilage compared to CO. On the other hand, in VP, those changes weren't obvious, and the reduction of the proteoglycan less than TS.
[Conclusion] It was understood that the specific changes were found in the articular cartilage with the loss of the mechanical stress and the electrical stimulation by VP generator had the effect that inhibited those structural changes. (COI:No)

### PP-257

### High-resolution micro-computed tomography analysis of the microarchitecture in Klotho mutant mouse osteocyte lacunae.

Tomoko Minamizaki[1], Faisal Ahmed[1], Shohei Kohno[1], Davood Kharagani[1], Tomonori Hoshino[1], Yuji Yoshiko[1] (*[1]Hiroshima University Graduate School of Biomedical and Health Sciences, Department of Calcified Tissue Biology*)

Morphology of osteocytes and osteocyte lacunae are deeply related to bone mass and fragility associated with skeletal disorders. We used high-resolution micro-computed tomography (CT) at 700 μm resolution to evaluate osteocyte lacuna parameters in Klotho-hypomorphic (*kl/kl*) mice exhibiting osteopenia and aberrant osteocytes and compared them with wild-type (WT) mice. Six-week-old mouse tibiae were scanned, followed by reconstruction, and osteocyte lacuna parameters in the range of 1.4 mm thickness were analyzed. Of parameters tested, the lacunar surface per lacunar volume was lower in *kl/kl* mice than in WT mice. Average lacunar volume was smaller in WT mice than in *kl/kl* mice, whereas there was no significant difference in the average lacunar surface. Both changes in the eigenvalues in the longitudinal direction and the elongation of the minor axis of osteocyte lacunae in *kl/kl* mice indicated that the shape of *kl/kl* osteocyte lacunae becomes rounded. These data suggest the morphological anomalies of osteocyte lacunae at the microarchitecture level could be detected by high-resolution micro-CT. (COI:No)

### PP-258

### Preventive Effect of Boiogito, a Kampo medicine, on Surgically induced Osteoarthritis in Rats

Takayuki Okumo[1], Hideshi Ikemoto[1], Jun Oike[1,2], Yosuke Kunieda[1,2], Masataka Sunagawa[1] (*[1]Department of physiology, Showa University School of Medicine, Tokyo, Japan., [2]Department of Orthopedic Surgery, Showa University Fujigaoka Hospital, Yokohama, Japan*)

**Background**: The present *in vivo* study investigated the preventive effect of boiogito (BO), a Kampo medicine, on surgically induced knee osteoarthritis (KOA).
**Methods**: Twelve-week-old Wistar rats were subjected to destabilization of the medial meniscus (DMM) to induce KOA. Rats were orally administered BO at a concentration of 3% in standard powder chow for four weeks after surgery. The animals were divided into four groups: control, sham, DMM, and DMM + BO (each n = 6). The rotarod test was performed to monitor pain and the locomotive function. Histologically, KOA development was evaluated by toluidine blue staining, and the number of TRAP-positive osteoclasts in the subchondral bone was determined by TRAP staining.
**Results**: In the rotarod test, the walking time in the DMM + BO group was significantly improved than that in the DMM group. The administration of BO significantly suppressed cartilage degeneration and decreased the number of TRAP-positive osteoclasts in the subchondral bone.
**Conclusion**: The oral administration of BO may prevent posttraumatic KOA by inhibiting the degradation of articular cartilage and suppressing osteoclast proliferation in the subchondral bone. (COI:No)

### PP-259

### CXCR4$^+$CD45$^-$cells support osteoclastogenesis under hypoxic condition

Yuto Otsuka[1], Yo Goto[2], Takeo Sekiya[2], Hiromasa Aoki[1], Yuko Nagaya[3], Ken Miyazawa[2], Shigemi Goto[2], Mineyoshi Aoyama[1] (*[1]Department of Pathobiology, Nagoya City University Graduate School of Pharmaceutical Sciences, Japan, [2]Department of Orthodontics, School of Dentistry, Aichi-Gakuin University, Japan, [3]Department of Orthopedics, Nagoya City East Medical Center, Japan*)

Bone remodeling balance by bone-forming osteoblasts (OBs) and bone-resorbing osteoclasts (OCs) is important. Excessive activation of OCs causes many bone destruction diseases including osteoporosis. OCs can be differentiated from bone marrow cells (BMCs) under the tight regulation of the local bone environment. We previously reported that hypoxia enhanced OC differentiation and CXCR4$^+$CD45$^-$cells supported OC differentiation by secreting chemokines. In this study, we investigated the effect of CXCR4$^+$CD45$^-$ cells on OC differentiation under hypoxic condition. We removed CXCR4$^+$CD45$^+$cells (R1) or CXCR4$^+$ CD45$^-$cells (R2) from mouse BMCs, and cultured collected cells with RANKL and M-CSF. Removing R1 did not change OC formation, but removing R2 decreased total OC formation under both normoxic and hypoxic condition. Quantitative RT-PCR analysis revealed that RANKL and TNFR1 expression was increased in non-OC, and that TNFR1 expression was increased in OC. Under hypoxic condition, CXCR4$^+$CD45$^-$cells is also important for OC differentiation. The increase of TNFR1 expression by hypoxia suggested that hypoxia could enhance TNF-$\alpha$ signal and support a microenvironment for osteoclastogenesis. (COI:No)

### PP-260

### Contributions of the third and fourth digits and the second and fifth digits of the flexor digitorum superficialis muscle to elbow valgus stability

Kanta Matsuzawa[1], Mutsuaki Edama[1,2], Sae Maruyama[1], Noboru Sato[2] ([1]*Institute for Human Movement and Medical Sciences, Niigata University of Health and Welfare,* [2]*Division of Gross Anatomy and Morphogenesis, Niigata University Graduate School of Medical and Dental Sciences*)

The purpose of this study is to clarify the contributions of the 3rd and 4th digits and the 2nd and 5th digits of flexor digitorum superficialis (FDS) to elbow valgus stability.
13 Thiel cadaver elbows divided into two groups. The joint space (JS) of the humeroulnaris joint was measured in the order that the tendinous insertions of the FDS were preserved (intact), the tendinous insertions of 3rd and 4th digits or 2nd and 5th digits were cut (3/4 cut, 2/5 cut, respectively). With the elbow at 30° flexion, valgus stress was gradually increased to 0, 30, and 60 N using the Telos device, and the JS was measured by ultrasonography at each load. Paired *t*-test was performed to compare the JS of the intact and the 3/4 cut or 2/5 cut states. Student's *t*-test was performed to compare the JS of the 3/4 cut and 2/5 cut states.
The JS was significantly greater in the 3/4 cut and 2/5 cut states than in the intact state at 30 and 60 N. There was no significant difference in the JS between the 3/4 cut and 2/5 cut states at all loads.
This study suggested that the 3rd and 4th digits and the 2nd and 5th digits may be involved in valgus stability, but there may be no difference between them. (COI:No)

### PP-261

### Histological properties of the external urethral sphincter of rats with simulated birth trauma

Toshiko Tsumori[1], Wakako Tsumiyama[2] ([1]*Dept. Nurs., Fac. Hlth. Welf., Pref. Univ. Hiroshima,* [2]*Dept. Phys. Ther., Fac. Hlth. Welf., Pref. Univ. Hiroshima*)

The external urethral sphincter (EUS) plays a crucial role in urinary continence. Understanding of the morphological features of the EUS in female rats after vaginal distention (VD), a model of birth trauma, contributes to the evaluation of its functional and metabolic properties. Our recent study demonstrated that the EUS in female rats expressed one slow (type 1) and two fast (types 1A and 2B) myosin heavy chain (MHC) isoforms (Tsumori and Tsumiyama, 2017). We further showed that VD may induce changes in the MHC expression in the EUS, especially in the type 2B isoform (Tsumori and Tsumiyama, 2018). In the present study, we examined the expression pattern of MHC isoforms in the EUS of rats from 3 days to 8 weeks after VD using triple immunofluorescence staining. The type 2B fibers in the EUS were selectively damaged in the early stages post-VD and did not recover fully later. Electron microscopy indicated that some myoblasts and myotubes appeared in the sphincter layer 1 week after VD. However, myogenesis after VD may not contribute to the restoration of a normal fiber composition in the female rat EUS. (COI:No)

### PP-262

### The effects of high mobility group box 1(HMGB1) to the regenerating myofibers.

Koji Sakiyama[1], Yudai Ogasawara[1,2], Go Onozawa[1,3], Arata Nagasaka[1], Yasuhiko Bando[1], Osamu Amano[1] ([1]*Div. Anat., Meikai Univ. Sch. Dent.,* [2]*Div. Maxillofac. Surg. 2, Meikai Univ. Sch. Dent. ,* [3]*Div. Maxillofac. Surg. 1, Meikai Univ. Sch. Dent.*)

High mobility group box 1 (HMGB1) is present in the nucleus of all normal cells and known for its participation in the maintenance of homeostasis. We investigated the localization of HMGB1 in the carcinoma and myofibers adjacent and distal to the carcinoma. Furthermore, HMGB1 was expressed in regenerating myofibers where myofibers were destroyed by the carcinoma. The aim of this study is to clarify roles of HMGB1 in the process of myofiber regeneration in the muscle dystrophy model mouse. C57BL/10-mdx mice were used as the muscle dystrophy model mouse. Mice were observed at the age of 3, 4, 5 and 8 weeks. Anti-HMGB1, Anti-Pax7; the marker of muscle satellite cells, and Anti-MyoD antibodies were applied for immunohistochemistory. HMGB1 was expressed in necrotic at the age of 3 weeks and repaired myofibers at the age of 4 weeks. Further, Pax7 was expressed in surrounds of normal and repaired myofibers, but not expressed in necrotic myofibers. Therefore, HMGB1 was suggested to induce regeneration of muscles. (COI:No)

### PP-263

### Involvement of High mobility group box 1 (HMGB1) in muscle fibers periphery of tongue carcinoma.

Yudai Ogasawara[1,3], Koji Sakiyama[2], Go Onozawa[1,4], Arata Nagasaka[2], Yasuhiko Bando[2], Osamu Amano[2] ([1]*Div.Anat.,Meikai Univ.Grad,Sch,Dent,* [2]*Div.Anat.,Meikai Univ.Sch,Dent,* [3]*Div.Oral,Maxillofac,Surg Ⅱ.,Meikai Univ. Sch,Dent,* [4]*Div.Oral,Maxillofac,Surg Ⅰ.,Meikai Univ.Sch,Dent*)

Our previous study showed strongly expressed high mobility group box 1 (HMGB1) is involved in necrosis occurred in muscle fibers close to the tongue carcinoma. On the other hand, it has also been suggested that HMGB1 is involved in the repair of muscle fibers after inflammation. Therefore, we investigated how HMGB1 and its receptor RAGE (receptor for advanced glycosylation endproducts) play their roles in the process of regeneration after necrotic of muscle fibers. The SCC7 cells were injected once into the tongue of BALB/cAJcl nude mice in order to create the tongue carcinoma model. After the injection, samples were collected after 2, 3 and 4 weeks. Necrosis of muscle fibers accompanying immunoreactivity for HMGB1 and RAGE were observed in tongue of SCC7-injected mice. Regenerated muscle fibers with a central nuclear were observed 3 weeks after SCC7 injection. Regenerated muscle fibers expressed PAX7, a marker for satellite cells. In addition, HMGB1-expression without RAGE-immunoreaction was observed around the regenerating muscle fibers. These results suggested that HMGB1 is involved in muscle regeneration in tongue carcinoma. (COI:No)

### PP-264

### Ectopic UCP3 expression regulates muscle mass through the modification of mitochondrial turnover and redox state in skeletal muscle

Shohei Kohno[1], Yuji Yoshiko[1], Christopher Riley[2], Edward Mills[3] ([1]*Department of Calcified Tissue Biology, Hiroshima University Graduate School of Biomedical and Health Sciences,* [2]*Department of Cancer Biology, Dana-Farber Cancer Institute, Harvard Medical School, USA,* [3]*Division of Pharmacology and Toxicology, College of Pharmacy, The University of Texas at Austin, USA*)

Skeletal muscle (SKM) atrophy is a common feature of a variety of conditions such as starvation, cachexia and aging. Uncoupling protein 3 (UCP3), a SKM-enriched member of the mitochondrial solute carrier family, is markedly induced during SKM atrophy. To define the functional relevance of UCP3 induction for muscle atrophy, we utilized SKM specific UCP3 transgenic (Tg) mice. Tg mice exhibited a significant reduction in tissue weight and cross-sectional area of fast type tibialis anterior (TA) muscle. In Tg mice, mitochondrial unfolded protein response signaling pathway was activated, which is the potential mechanism of muscle mass reduction in Tg mice. Surprisingly, Tg mice had a resistance to starvation induced muscle wasting, whereas they had smaller muscle mass in the basal condition. To investigate the potential mechanism by which UCP3 prevent starvation induced muscle loss, we analyzed mitochondrial homeostasis in SKM. Results clearly indicated that UCP3 lowers mitochondrial turnover accompanied with increased mitochondrial fission and lower mitochondrial ubiquitination. This study demonstrated dual roles of UCP3 in the muscle mass regulation. (COI:No)

### PP-265

### Effect of beta-2 adrenergic agonist and calcitonin gene related peptide on mRNA expression of myosin heavy chain class II (MyHCII) and interleukin-6 in murine myocytes

Junko Yamaji[1], Yoshiaki Mori[2] ([1]*Dept. of Nutr. Sci., Kansai Univ. of Welf. Sci., Kashiwara, Japan,* [2]*Dept. of Rehab., Kansai Univ. of Welf. Sci., Kashiwara, Japan*)

At neuromuscular junctions, beta-2 adrenergic receptor and calcitonin gene related peptide (CGRP) receptor on skeletal muscle cells receive neurotransmitters from motor nerves and also activates adenylate cyclase pathway. However, the role of these neurotransmitter receptors and the cAMP in mRNA expression of MyHC and interleukin (IL) -6 in skeletal muscle remains unclear. In this study, we investigated that beta-2 agonists and CGRP on mRNA expression of $MyHCII_b$ and IL-6 in murine myocytes.
Then our study yielded the following results: (1) The $MyHCII_b$ mRNA level was significantly increased by IL-6 induced by calcineurin activators and was significantly attenuated by calcineurin inhibitor. (2) The $MyHCII_b$ mRNA level was not affected by medium supplemented with forskolin, with PKA inhibitor and with beta-2 agonists, isoproterenol. (3) The IL-6 mRNA level was not also affected by medium supplemented with forskolin, with PKA inhibitor and with beta-2 agonists, isoproterenol.
These results indicated that production of IL-6 by calcineurin activation increases $MyHCII_b$ mRNA but that CGRP-cAMP pathway is not participated for mRNA expression of IL-6 and $MyHCII_b$ in C2C12 cells. (COI:No)

### PP-266

### Effect of β-adrenergic receptor stimulation on MyHC I mRNA in C2C12 skeletal muscle cells.

Yoshiaki Mori[1], Junko Yamaji[2], Reiko Hiroshima[1], Manabu Miyamoto[1] ([1]*Dept of Rehabil Sci, Kansai Univ of Welf Sci, Kashiwara, Japan,* [2]*Dept of Nutr Sci, Kansai Univ of Welf Sci, Kashiwara, Japan*)

Our previous study using C2C12 cells indicated that calcineurin activation upregulates myosin heavy chain type I (MyHC I) mRNA level through production of interleukin-6 (IL-6). In this study, we examined the roles of β-adrenergic receptor which is known to express in skeletal muscle cells on mRNA level of MyHC I in C2C12 cells. C2C12 cells were induced to differentiate to myotubes by medium exchange to D-MEM containing 2%FBS. The cells were incubated in D-MEM containing 2%FBS with chemical compounds at the beginning of differentiation and removed after 24hr, and were maintained in differentiation medium for 3 days. MyHC I mRNA expression level was measured by the real-time PCR method. MyHC I mRNA level was significantly increased by the administration of β-agonist, isoproterenol. The effects of forskolin and 8Br-cAMP on the MyHC I mRNA level were identical to that of isoproterenol. Additionally, the effect of forskolin on MyHC I mRNA expression was significantly inhibited by the co-administration of PKA inhibitor, H-89. These results suggest that the upregulation of MyHC I mRNA level by the stimulation of β-agonist is involved in cAMP/PKA-mediated mechanisms in C2C12 cells. (COI:No)

### PP-267

### Effects of estrogen on satellite cells and domain size in skeletal muscles following resistance exercise

Yung-Li Hung[1], Toshiharu Natsume[2], Shuichi Machida[3] ([1]*Inst. of Health and Sports & Med., Juntendo Univ., Chiba, Japan,* [2]*Dept. of Human Structure & Function, Sch. of Med., Tokai Univ., Kanagawa, Japan,* [3]*Dept. of Health and Sports Sci., Juntendo Univ., Chiba, Japan*)

The muscle mass is maintained by the satellite cells present in the muscle fibers. Estrogen ($E_2$) regulates satellite cell maintenance, but the effects of $E_2$ on satellite cells in skeletal muscle following resistance exercise are unclear. 10 week-old female rats were divided into 6 groups: sham sedentary, sham training, ovariectomy sedentary (OVX-Sed), ovariectomy training (OVX-T), ovariectomy $E_2$ treatment sedentary (OVXE-Sed), and ovariectomy $E_2$ treatment training (OVXE-T) groups.8 weeks after the operation, the rats in both the training groups were trained to climb a ladder while bearing a load. $E_2$ treatment was administered using subcutaneous insertion of a 17β-estradiol pellet. After 8 weeks, the flexor hallucis longus muscles were collected and analyzed. The number of Pax7 cells per fiber was increased after climbing training ($p<0.05$), but the difference was not significant. The domain size was significantly increased in the rats in the OVXE-T group compared with those in the OVXE-Sed group, but it was not significantly different between the OVX-T and OVX-Sed groups. These results demonstrated that $E_2$ regulates exercise-induced muscle hypertrophy by increasing the domain size. (COI:No)

### PP-268

### Drug screening for muscle atrophy using murf1-transgenic zebrafish.

Genri Kawahara[1], Mami Nakayashiki[1], Yukiko Hayashi[1] ([1]*Dept Pathophysiol, Tokyo Med Univ, Tokyo, Japan*)

To monitor the expression of Muscle RING-finger protein-1 (*MURF1*) gene, which is one of marker molecules of muscle atrophy, a transgenic (Tg) zebrafish line (*murf1*:EGFP) was created with microinjection of *murf1* promoter-EGFP cDNA construct using tol2 transposon system.

During early development in the Tg fish (*murf1*:EGFP) line, EGFP signals were observed in skeletal muscle and heart from 1 day post-fertilization (dpf). RT-PCR analysis confirmed that the *murf1* gene expression was corresponded with EGFP expression after 1 dpf. In the adult Tg fish, murf1 expression corresponding with EGFP were mainly observed in skeletal muscle and heart. Treatment with dexamethasone solution for 24 hours induced up-regulation of EGFP expression in *murf1*:EGFP zebrafish. These results indicated that the murf1 expression could be monitored using the *murf1*:EGFP -Tg fish. We have screened 1,280 drugs to discovery drugs to reduce the expression of zebrafish murf1 using the *murf1*:EGFP -Tg fish, and . Five candidate drugs to reduce murf1 expression drugs were identified by drug screens using murf1-Tg fish. Our *murf1*:EGFP -Tg fish line might be excellent tool for therapeutic drug screening for muscle atrophy. (COI:No)

### PP-269

### Development of x-ray diffraction methods for in vivo skeletal muscle with maintained blood supply

Naoya Nakahara[1], Hideki Yamauchi[1], Maki Yamaguchi[1], Kazuhiro Hirano[1], Shigeru Takemori[1] ([1]*Dept Mol Physiol, Jikei Univ Sch Med, Japan*)

Tensile stress and metabolic demand of contracting skeletal muscle are so intense that repetitive contraction gradually develops so called muscle fatigue process deteriorating muscle function and structure. To monitor the progress of structural deterioration in muscle sarcomere with repetitive contraction, x-ray diffraction is a suitable technique. Regular arrangement of myoproteins in sarcomere gives rise to a specific series of fine reflections and layer-lines in the diffraction pattern. However, dissection of muscle tissue for an x-ray diffraction experiment limits the diffusion of gases and solutes to accelerate metabolic deterioration, and isolation of a muscle fiber may cause fragility to accelerate mechanical deterioration. To obtain x-ray diffraction patterns of skeletal muscle at states as physiological as possible, we tried to obtain x-ray diffraction patterns from skeletal muscle with maintained blood flow. X-ray diffraction patterns from extensor digitorum longus muscle of anesthetized 6-month female ICR mice were taken at BL-6A in KEK, Tsukuba. Diffraction patterns of high quality were obtained showing high orders of reflections and layer lines. (COI:No)

### PP-270

### Effects of omecamtiv mecarbil on the contractile properties of skinned porcine left atrial and ventricular muscles

Tomohiro Nakanishi[1], Takako Terui[2], Fuyu Kobirumaki[1], Norio Fukuda[1] ([1]*Dept Cell Physiol, Jikei Univ, Tokyo, Japan,* [2]*Dept Anesht, Jikei Univ, Tokyo, Japan*)

**Introduction:** Omecamtiv mecarbil (OM) is a compound that has been developed to treat heart failure via targeting myosin.

**Results:** Force was measured with skinned porcine left atrial (LA; ~100% α-MHC) and ventricular (LV; ~100% β-MHC) fibers (sarcomere length, 2.1 mm). OM left-shifted the mid-point ($pCa_{50}$) of the force-pCa curve (i.e., $\Delta pCa_{50}$) by 0.08 and 0.21 pCa units in LA at 0.5 and 1.0 μM, respectively. $\Delta pCa_{50}$ was significantly greater in LV; the values were 0.17 and 0.32 pCa units at 0.5 and 1.0 μM, respectively. Following thin filament reconstitution with troponin from rabbit fast skeletal muscle (sTn), the $Ca^{2+}$-sensitizing effect of OM was decreased by ~60%, with the $\Delta pCa_{50}$ values 0.08 and 0.13 in LA and LV, respectively, at 1.0 μM.

**Conclusions:** OM increases $Ca^{2+}$ sensitivity in both LA and LV, with the effect greater in LV (showing slower contractile dynamics). Under conditions where contractile dynamics is accelerated via sTn reconstitution, the compound's $Ca^{2+}$-sensitizing effect is diminished in both preparations. It is therefore likely that the $Ca^{2+}$-sensitizing effect of OM is more pronounced with deceleration of the cross-bridge cycling rate. (COI:No)

### PP-271

### Synchrony of sarcomeric movement regulates left ventricular pump function in the *in vivo* beating mouse heart.

Fuyu Kobirumaki-Shimozawa[1], Tougo Shimozawa[2], Kotaro Oyama[3], Jia Li[4], Tomohiro Nakanishi[5], Takako Terui[5], William E. Louch[4,6], Shinichi Ishiwata[7], Norio Fukuda[1] ([1]*Dept Cell Physiol, Jikei Univ Sch Med, Tokyo, Japan,* [2]*Tech Dev, Sch Sci, Univ Tokyo, Tokyo, Japan,* [3]*QST, Gunma, Japan,* [4]*Inst Exp Med Res, Oslo Univ hosp, Univ Oslo, Oslo, Norway,* [5]*Dept Anesthesiol, Jikei Univ Sch Med, Tokyo, Japan,* [6]*KG Jebsen Cent Card Res, Univ Oslo, Oslo, Norway,* [7]*Dept Phys, Fac Sci Eng, Waseda Univ, Tokyo, Japan*)

We previously developed a high speed (100 frames per second), high resolution (20 nm) imaging system for myocardial sarcomeres in living mice (Kobirumaki-Shimozawa et al., *J Gen Physiol* 2016). In the present study, we analyzed dynamic behaviors of neighboring sarcomeres within left ventricular (LV) myocytes of the *in vivo* beating mouse heart. Z-disks were imaged by expressing α-actinin-AcGFP. In order to quantify the contribution of individual sarcomeres to myofibrillar dynamics, we developed a contribution index (CI) to correlate movements between a sarcomere and a myofibril, with values ranging from -1 (full dyssynchrony) to 1 (full synchrony). We found that 1) CI varied markedly between sarcomeres, with values between ~-0.35 and ~0.65 during the cardiac cycle, and 2) the average CI during normal systole was linearly decreased from ~0.3 to ~0.15 in association with a decrease in LV developed pressure from 96.4 to 4.7 mmHg. The present findings suggest that sarcomere synchrony regulates myofibrillar dynamics and, accordingly, rhythmic myocyte contractions in the beating mouse heart *in vivo*. (COI:No)

### PP-272

### Madagascine induces vasodilatation via AMPK-mediated eNOS activation and ROCK inhibition

Hakuchou Ro[1], Depeng Chen[2], Hara Hayashi[2], Ying Zhang[1], Chika Morita[1], Hiroko Kishi[1], Sei Kobayashi[1] ([1]*Department of Molecular and Cellular Physiology, Yamaguchi University, Graduate School of Medicine,* [2]*Dalian Medical University*)

The effects of madagascine on contractions of rat mesenteric resistance arteries (MRAs) induced by $K^+$, methoxamine, and endothelin-1 were respectively studied. We aimed to characterize the vasodilatory effect of madagascine on vasoconstriction and reveal the potential mechanisms.
The vasodilatory effect of Madagascine in endothelium intact MRAs was blocked by an endothelial NO synthase (eNOS) inhibitor, L-NAME and an AMPK inhibitor, compound C. Madagascine also significantly relaxed the sphingosylphosphorylcholine (SPC)-induced contraction without endothelium and the effect was abolished by compound C. Madagascine significantly increased the phosphorylation level of eNOS and decreased both the SPC-induced phosphorylation levels of myosin phosphatase target subunit 1 (MYPT1) and myosin light chain 20 (MLC20), which were canceled by the small interfering RNA-induced knockdown of AMPK. In summary, madagascine exerted vasodilatation through activating AMPK, leading to the activation of eNOS in the endothelium and the inhibition of SPC/ROCK/MYPT1 in VSM. This study suggests the potential value of madagascine in amelioration of vasospasm-related cardiovascular diseases. (COI:No)

### PP-273

### Compound B from traditional Chinese medicine inhibits the Rho-kinase (ROK)-mediated $Ca^{2+}$-sensitization of vascular smooth muscle contraction induced by a spasmogen, sphingosylphosphorylcholine (SPC)

Minhui Xu[1], Min Zhang[1], Hakuchou Ro[1], Sen Ro[1], Nan Li[1], Ying Zhang[1], Tomoka Morita[1], Hiroko Kishi[1], Sei Kobayashi[1] ([1]*Department of Molecular and Cellular Physiology, Yamaguchi University Graduate School of Medicine, Ube, Japan*)

Rho-kinase (ROK)-mediated $Ca^{2+}$-sensitization of vascular smooth muscle (VSM) plays a critical role in abnormal VSM contractions such as vasospasm. As a novel spasmogen activating this pathway, we previously identified SPC, which activates Fyn by its translocation from the cytosol to the cell membrane and leads to the ROK-mediated $Ca^{2+}$-sensitization. We also found that eicosapentaenoic acid (EPA) selectively inhibits the SPC-induced $Ca^{2+}$-sensitization of VSM contractions by blocking the Fyn translocation and activation, without affecting the $Ca^{2+}$-dependent ones, and clinically prevents vasospasm after subarachnoid hemorrhage. However, the lipophilicity of EPA limits its intravenous injection for the urgent and serious cases.
After extensive screening for eatable and water-soluble molecule, we finally discovered compound B from traditional Chinese medicine, pre- or post-incubation of which inhibited the SPC-induced abnormal contraction of porcine coronary artery, with little inhibitory effect on the high $K^+$-induced $Ca^{2+}$-dependent contraction. These data suggest that compound B is the novel molecule which is useful for both prevention and therapy of the abnormal vascular contractions. (COI:No)

### PP-274

### Histological analysis of the murine duodenocolic fold at embryonic day 18.5.

Sawa Onouchi[1,2], Yasuro Atoji[1], Shouichiro Saito[1,2] ([1]*Lab. Vet Anat., Dept. Vet. Med., Fac. Appl. Biol. Sci., Gifu Univ. ,* [2]*Lab. Vet. Anat., Grad. Vet. Sci., Gifu Univ*)

For elucidating the murine duodenojejunal flexure formation, it is needed to investigate both intestinal and mesenteric factors. This study focused on the duodenocolic fold, a mesentery attaching the ascending duodenum to the descending mesocolon. In this study, histological structure of the duodenocolic fold was examined by using semi-serial crossing paraffin sections of a murine peritoneal cavity at embryonic day (E) 18.5 with trichrome method and immunohistochemistry. The duodenocolic fold was observed as triangle structure composed of a loose connective tissue, but an area near the duodenum showed a denser connective tissue extended from the longitudinal smooth muscle layer of the duodenum. In immunohistochemistry of $\alpha$ smooth muscle actin, the area contained smooth muscle cells. Because this area did not include blood vessels, the smooth muscle cells were not derived from blood vessels. Taken together, the murine duodenocolic fold had a smooth muscle bundle extended from the smooth muscle layer of the duodenum at E18.5. Although there were no reports about the ligament of Treitz in tetrapods except human, this smooth muscle bundle might be homogenous to the ligament. (COI:No)

### PP-275

### Age-related changes of estrogen synthesis in the rat gastric mucosa

Hiroto Kobayashi[1], Nobuyuki Shirasawa[2], Akira Naito[1] ([1]*Dept. Anat. Struct. Sci., Yamagata Univ. Sch. Med.,* [2]*Dept. Rehab., Tohoku Bunka Gakuen Univ. Facul. Med. Sci. Welf*)

Aromatase, which is enzyme converting androgen into estrogen, exists in the parietal cells of the rat stomach. We showed that the aromatase appeared at 20 days, increased gradually until 40 days, and persisted plateau until 3 months after birth. In this study, we investigated the aromatase in 6, 12, and 18 months old male Wistar rats compared to 3 months as a control. Gastric aromatase continued to be expressed until 18 months by immunohistochemistry. Aromatase protein by Western blotting and mRNA expression by qPCR levels decreased significantly at 18 months. $H^+/K^+$-ATPase immunostained area in the gastric mucosa decreased significantly at 12 and 18 months. It was clarified that gastric aromatase continued to be expressed even after 3 months of age, but expression level at 18 months were decreased significantly. Since $H^+/K^+$-ATPase is a marker of the parietal cells, the decreasing of gastric estrogen synthesis should be caused by the parietal cells decrement with aging. (COI:No)

### PP-276

### Visualization (optical biopsy) for intestinal tissue structure using multiphoton microscopy after the staining with an edible dye

Shujie Wang[1], Aika Kaito[2], Kazushi Kimura[3], Kouji Tanaka[1], Kyosuke Tanaka[5], Masahide Goto[1], Akira Mizoguchi[4] ([1]*Cell Biology and Histology, Graduate School of Medicine, Mie University,* [2]*Department of Physiology, Graduate School of Medicine, Mie University,* [3]*Department of Physical therapy, Hokkaido Bunkyo University,* [4]*Department of Personalized Cancer Immunotherapy, Graduate School of Medicine, Mie University,* [5]*Department of Endoscopic Medicine, Mie University Hospital*)

Our research goal is to noninvasively visualize not only normal tissue structure of large intestine but also but also ultra-early carcinoma, which means that the tumor size is less than a diameter of ~1 mm. Here, we have performed the screening of about 1,200 types of edible pigments approved by the FDA, which is considered to be safe for oral administration in humans.
In a screen using MDCK cells to express GFP-tagged Val-Ras (constitutively active form of Ras) in a tetracycline-dependent manner, we have identified Red No. 3, Merbromin, and Red No. 104 as putative dyes. In another screen using mice after exposure to a chemical carcinogen, we have found that multiphoton microscopy can visualize glandular crypts in the colon after the staining with curcumin, which accumulates at carcinomas rather than at normal glandular cells.
Thus, we have developed a new noninvasive technique using multiphoton microscopy after the staining with the above putative dyes. We will also discuss future plans including clinical applications in cancer patients. (COI:No)

### PP-277

### Characterization of cellular communication through membrane nanotubes in hepatoblastoma cells

Keiko Fujita[1], Sachiko Matsumoto[2], Kazumasa Fujita[1], Masumi Akita[3], Masabumi Nagashima[1] ([1]*Dept. Anat., Fac. Med., Saitama Med. Univ. Saitama, Japan,* [2]*Div. Morphol., Biomed. Res. Cent., Fac. Med., Saitama Med. Univ. Saitama, Japan,* [3]*Sept. Sapie, Tokyo, Japan*)

Cellular communication between tumor cells and tumor microenvironment is an important factor for pre-metastatic niche formation and metastasis. Recently, it was recognized the existence of thin membranous tubes among several cell types that named membrane nanotubes or tunneling nanotubes. The membrane nanotubes were identified as actin-based intercellular conduits, which facilitate the transfer of several cargoes such as lysosomes, mitochondria, viruses, and miRNAs.
Using 2D and 3D culture systems we confirmed the formation of membrane nanotubes connecting two distant hepatoblastoma cells. To confirm the existence of the microtubule- and actin-based transport systems in membrane nanotubes, we investigated the presence of molecular motors for intracellular cargo transport. Polyamine levels are elevated in cancer cells which contributes to malignant transformation and increased cell proliferation. Polyamine reactivity was observed both membrane nanotubes and interconnected hepatoblastoma cells.
This work was supported by JSPS KAKENHI Grant Numbers 16K11353, 20K08990.
(COI:No)

### PP-278

### Effects of polymethoxyflavone on liver and spleen in APC gene-mutated mice

Chika Tanaka[1], Kiyoka Inenaga[1], Takuto Miyazaki[1], Shinichirou Hino[1] (*[1]Dept. Anat. & Physiol. Grad. Sch. Nakamura Gakuen Univ. Fukuoka Japan*)

We found that 5,7,3',4'-tetra-methoxyflavone (5,7,3',4'-TMF) inhibits β-catenin-dependent gene induction in colon cancer cell line. According to a previous study, $APC^{min/+}$ mice, a mouse model of intestinal neoplasia, observed abnormal glycogen distribution near the central vein in liver and increased splenic megakaryocytes in spleen. This study was to examine the effects of long-term oral administration of 5,7,3',4'-TMF on the liver and spleen using $APC^{min/+}$ mouse. Oral administration was performed up to 9 to 20 weeks of age. At around 13 weeks of age, weight loss was observed in $APC^{min/+}$ mice, but $APC^{min/+}$ orally treated with 5,7,3',4'-TMF did not observe weight loss. PAS staining of liver observed abnormalities in glycogen distribution near the central vein in $APC^{min/+}$ mice, but not in $APC^{min/+}$ mice orally treated with 5,7,3',4'-TMF. $APC^{min/+}$ mice observed spleen hypertrophy, and the boundary between red and white spleen marrow was indistinct, and splenic megakaryocytes were increased. However, in $APC^{min/+}$ mice treated orally with 5,7,3',4'-TMF, the boundary between red and white spleen marrow could be observed, and the increase in splenic megakaryocytes was inhibited. (COI:No)

### PP-279

### Effect of polymethoxyflavone on intestinal neoplasia in APC gene-mutated mice

Shinichiro Hino[1], Takuto Miyazaki[1], Kiyoka Inenaga[1], Chika Tanaka[1] (*[1]Dept. Anat. & Physiol. Grad. Sch. Nakamura Gakuen Univ. Fukuoka Japan*)

Accumulation of β-catenin due to APC (adenomatous polyposis coli) gene mutation leads to the development of intestinal neoplasia. We have found 5,7,3',4'-tetra-methoxyflavone (5,7,3',4'-TMF) that inhibit β-catenin-dependent gene induction. In this study, we examined the effects of long-term oral administration of 5,7,3',4'-TMF to $APC^{min/+}$ mice, which are intestinal adenoma model animals. 5,7,3',4'-TMF was orally administered once a week. Oral administration was performed up to 9 to 20 weeks of age. From 13-week age, $APC^{min/+}$ mice weight loss was observed. On the other hand, weight loss was suppressed in $APC^{min/+}$ mice TMF administration group. Atypical glands were found in the epithelial cells of the small and large intestines of $APC^{min/+}$ mice, and β-catenin was abnormally accumulated in the nucleus at that site. Similar atypical glands were found in TMF-administered $APC^{min/+}$ mice, and β-catenin accumulation in the nucleus was also observed. When the expression of phosphorylated ERK1/2, which is involved in cell proliferation, was analyzed by immunostaining, strong activation was observed in $APC^{min/+}$ mice, but activation was attenuated in the TMF-administered group. (COI:No)

### PP-280

### Activation of p38 MAPK is required for the nuclear translocation of Nrf2 by lansoprazole in rat hepatic RL34 cells

Naoko Yamagishi[1], Yuta Yamamoto[1], Toshio Nishi[1], Takao Ito[1], Yoshimitsu Kanai[1] (*[1]Dept. of Cell Biol. and Anat., Wakayama Med. Univ*)

Lansoprazole is widely used gastrointestinal drug. Besides its inhibiting function against the acid secretion, lansoprazole has an antioxidant effect through the activation of Nrf2/HO1 pathway. We previously demonstrated the protective function of lansoprazole to the drug-induced hepatitis in rats. Lansoprazole is known to influence MAPK pathways, however, the responsible pathway for its hepatoprotective function remained unsettled. To begin with, we confirmed the Nrf2/HO1 pathway-mediated cytoprotective function of lansoprazole on RL34 cells. We then examined the effect of lansoprazole on the phosphorylation of the major MAPKs. Interestingly, lansoprazole induced the phosphorylation of p38 MAPK, but not of ERK1/2 or JNK, in RL34 cells. Moreover, a specific inhibitor of p38 MAPK blocked the lansoprazole-mediated cytoprotective function. Therefore, we propose that lansoprazole has a cytoprotective effect on hepatic cells against the oxidative-stress-induced cell death through the p38 MAPK/Nrf2/HO1 pathway. (COI:No)

### PP-281

### Effects of gastric estrogen on the liver in the gastrectomized rats

Takao Ito[1], Yuta Yamamoto[1], Naoko Yamagishi[1], Yoshimitsu Kanai[1] (*[1]Dept. Anat. & Cell Biol., Wakayama Med. Univ*)

Gastrectomy (GX) may lead to various disorders, called as postgastrectomy syndrome, including dumping syndrome, anemia, nutritional disorder, post gastrectomy gallstone and osteoporosis. To investigate the mechanism of the postgastrectomy syndrome, we used GX rat model and examined the effect on liver function after 10 days-gastorectomy.
Using a GX rat model, we showed that GX induced high blood glucose levels and hepatic lipid droplet accumulation. In Gx rat liver, the expression of L-type pyruvate kinase, the rate-limiting enzyme in glycolytic pathway, mRNA significantly decreased compared to sham operated. Further, the expression of carnitine palmitoyltransferase-1a, the enzyme in mitochondrial beta-oxidation pathway, mRNA decreased. (COI:No)

### PP-282

### Expression and neurochemical characterization of huntingtin-associated protein 1 with enteroendocrine cells in rat pylorus

Akie Yanai[1], Md Nabiul Islam Md Nabiul Islam[2], Maki Hayashi-Okada[2], Jahan Mir Rubayet[2], Tarif Abu Md Mamun [2], Kanako Nozaki[2], Koh-Hei Masumoto[2], Koh Shinoda[2] (*[1]Basic Labo. Sci. Yamaguchi Univ. Grad. Med., [2]Neurosci. Yamaguchi Univ. Grad. Med*)

Huntingtin-associated protein 1 (HAP1) is a neuronal cytoplasmic protein that is predominantly expressed in the brain and spinal cord. In addition to the central nervous system, HAP1 is also expressed in the peripheral organs including endocrine system. Different types of enteroendocrine cells (EEC) are present in the digestive organs. To date, the characterization of HAP1-immunoreactive (ir) cells remains unreported there. In the present study, the expression of HAP1 in pyloric stomach in adult male rats and its relationships with different chemical markers for EEC were examined employing single- or double-labelled immunohistochemistry and with light-, fluorescence- or electron-microscopy. HAP1-ir cells were abundantly expressed in the glandular mucosa but were very few or none in the surface epithelium. Double-labelled immunofluorescence staining for HAP1 and markers for EECs showed that almost all the gastrin (G) cells expressed HAP1. In contrast, HAP1 was completely lacking in delta -cells, EC-cells or enterochromaffin-like cells. Our current study is the first to clarify that HAP1 is selectively expressed in G-cells in rat pyloric stomach (COI:No)

### PP-283

### Short-chain fatty acid-evoked noncholinergic transepithelial ion transport in the mice terminal ileum

Kota Tsukamoto[1], Mao Ikeya[2], Ikuo Kimura[3], Shinichiro Karaki[1,2] (*[1]Laboratory of Physiology, Graduate School of Integrated Pharmaceutical and Nutritional Sciences, University of Shizuoka, [2]Laboratory of Physiology, School of Food and Nutritional Sciences, University of Shizuoka, [3]Department of Applied Biological Science, Graduate School of Agriculture, Tokyo University of Agriculture and Technology*)

Reflux of short-chain fatty acids (SCFAs) from cecum to ileum may induce a fluid secretion in the terminal ileum, but the SCFA-induced ion transport in small intestine is not fully understood. We therefore investigated the SCFA-induced ion transport in the mice terminal ileum. Mucosa-submucosal preparations of mouse terminal ileum were mounted on Ussing chambers, and short-circuit current ($I_{sc}$) were measured. Mucosal treatment of acetate and propionate concentration-dependently evoked phasic (P-1) and broad (P-2) increases in $I_{sc}$. These effects were insensitive for tetrodotoxin, atropine and piroxicam, but sensitive for amitriptyline, blocking tetrodotoxin-insensitive Nav1.9 channels. In the free fatty acid receptor (FFA)3 (GPR41)-KO mice, propionate and acetate evoked the same increases in $I_{sc}$. Whereas in FFA2 (GPR43)-KO mice, the propionate-evoked P-1 $I_{sc}$ increase was significantly reduced and the acetate-evoked P-1 $I_{sc}$ increase was completely abolished. It has reported that acetate selectively affects for FFA2, and propionate effects both of FFA2 and FFA3. Therefore, it is suggested that SCFA-evoked P-1 $I_{sc}$ increase is due to the activation of FFA2 and FFA3 (FFA2 > FFA3). (COI:No)

### PP-284

### Suppression of gastric reservoir function induced by anorexigenic substances in anesthetized rats

Motoi Kobashi[1], Yuichi Shimatani[2], Masako Fujita[1], Yoshihiro Mitoh[1], Ryusuke Yoshida[1] ([1]*Department of Oral Physiology, Okayama University, Japan,* [2]*Department of Medical Engineering, Faculty of Engineering, Tokyo City University, Tokyo 158-8557, Japan*)

Our previous studies revealed that appetite-enhancing peptides, such as orexin A, neuropeptide Y and ghrelin, facilitated phasic contractions of the distal stomach and induced relaxation of the proximal stomach. It is considered that the enhanced contraction of the distal stomach facilitates gastric emptying, and relaxation of the proximal stomach facilitates the accommodation of swallowed food. In the present study, we investigated that the effect of anorexigenic substances on gastric motility. The effects of glucagon-like peptide-1 or oxytocin on the gastric motility were examined. The administration into the fourth ventricle of anorexigenic peptides suppressed phasic contractions of the distal stomach and increased the intragastric pressure of the proximal stomach. Thus, anorexigenic peptides showed the opposite response to appetite-enhancing peptides. Furthermore, the actions of orexin-1 receptor antagonist on the effects of these anorexigenic peptides were also investigated. The experimental protocols were approved by the Okayama University Animal Use Committee. This work was supported by JSPS KAKENHI Grant Number 15K00818 and 18K11099. (COI:No)

### PP-285

### Roles of dopamine in regulating peristaltic contractions of rat proximal colon

Hiroyuki Nakamori[1], Kenta Noda[1], Retsu Mitsui[1], Hikaru Hashitani[1] ([1]*Dept Cell Physiol, Nagoya City Univ Grad Sch Med, Nagoya, Japan*)

Since Parkinson's disease patients often suffer from constipation associated with a loss of enteric dopaminergic neurons, roles of dopaminergic neurons in generating colonic peristalsis were investigated. Cannulated segments of rat proximal colon were abluminally perfused with Krebs solution and luminally perfused with 0.9% saline. Spatio-temporal maps of diameter changes were constructed from video recordings. All drugs were applied abluminally. Blockade of nitrergic transmission prevented oro-aboral propagation of peristaltic waves with a colonic constriction, while cholinergic transmission blockade prevented the propagation with a dilatation. A $D_1$-like receptor antagonist disrupted the peristaltic waves with a constriction, while a dopamine reuptake inhibitor diminished the peristaltic waves with a dilatation. Exogenous dopamine abolished the peristaltic waves with a dilation in a $D_1$-like receptor antagonist-sensitive manner. $D_1$ receptor immunoreactivity was co-localized to nitrergic neurons. Enteric dopamine appears to facilitate nitrergic neurons via $D_1$-like receptors to suppress asynchronous contractile activity resulting in the generation of coordinated colonic peristalsis. (COI:No)

### PP-286

### Segmental difference of aging in intestinal barrier and nutrient absorption function in aged mice

Fumiya Kurihara[1], Wendy Hempstock[1], Noriko Ishizuka[1], Hisayoshi Hayashi[1] ([1]*Laboratory of Physiology, Graduate School of Food and Nutritional Sciences, University of Shizuoka*)

Aging is characterized by a general decline in physiological function and increased probability of death. The rate of deterioration differs in each organ and it is thought that the functional decline in the intestine is slower than that of the musculoskeletal system, so little attention has been paid to the effect of aging on the intestine. The intestine absorbs nutrients as well as acting as a barrier to keep noxious substances out. We conducted experiments using senescence accelerated mouse prone-1 (SAMP1) mice and age-matched SAMR1 mice (mice of the same background that age normally). We isolated the jejunum, ileum, cecum, proximal and distal colon and measured nutrient-induced currents ($\Delta$Isc) and transepithelial electrical conductance (Gt) using Ussing chambers. Glucose-induced $\Delta$Isc was significantly decreased in SAMP1 mice and in the middle small intestine (SI), Gt was significantly decreased. To understand the reason of Gt decrease, $Na^+$ permeability was assessed by dilution potential and the $Na^+$ permeability of the middle SI was significantly decreased compared to SAMR1 mice. These results suggest that the effect of aging on intestinal function is segment-dependent. (COI:No)

### PP-287

### Activation of melanocortin 1 receptors in the spinal defecation center enhanced colorectal motility in rats.

Kiyotada Naitou[1], Hiromi Ueda[1], Mitsuya Shiraishi[1], Tatsunori Masatani[2], Hiroyuki Nakamori[3], Kazuhiro Horii[4], Takahiko Shiina[4], Yasutake Shimizu[4] ([1]*Department of Basic Veterinary Science, Joint Faculty of Veterinary Science, Kagoshima University, Japan,* [2]*Transboundary Animal Diseases Research Center, Joint Faculty of Veterinary Medicine, Kagoshima University, Kagoshima 890-0065, Japan.,* [3]*Department of Cell Physiology, Nagoya City University Graduate School of Medical Sciences,* [4]*Department of Basic Veterinary Science, Laboratory of Physiology, The United Graduate School of Veterinary Sciences, Gifu University*)

In general, it is considered that only melanocortin 3 and 4 receptors (MC3, 4R) are expressed in the central nervous system. Previously, we found intrathecally administered alpha-melanocyte-stimulating hormone (alpha-MSH), which binds to MC1 and 3-5R, activated the sacral parasympathetic nerves and enhanced colorectal motility in rats. Here, we investigated receptor subtypes responsible for the effect of alpha-MSH on the spinal defecation center in rats. To determine receptor subtypes expressed in the spinal defecation center, we examined expression of MC1-5R mRNA using RT-PCR. As a result, only MC1 and 4R mRNA were detected in the L6-S1 spinal cord. To confirm responsible receptor subtypes for the effect of alpha-MSH, we performed pharmacological experiments using subtype selective agonists. Although intrathecally administered THIQ, a MC4R selective agonist, failed to enhance colorectal motility, intrathecally administered BMS470539, a MC1R selective agonist, enhanced colorectal motility similarly to alpha-MSH. These results suggest that MC1R is the responsible receptor for the effect of alpha-MSH on the spinal defecation center in rats. (COI:No)

### PP-288

### Effect of the cysteine protease inhibitor cystatin D on growth of oral cancer cells

Junko Fujita-Yoshigaki[1], Megumi Yokoyama[1], Osamu Kato[1] ([1]*Department of Physiology, Nihon University School of Dentistry at Matsudo*)

Oral squamous cell carcinoma (OSCC) is the most common oral malignancy with poor prognosis. Cystatin D, a salivary cysteine protease inhibitor, has been reported to be have anti-cancer activity when transported to nuclear, but the effect on OSCC is not clear. In this study, the effect of cystatin D on HSC-3 cells derived from human OSCC was examined. HSC-3 cells were cultured in the absence and presence of the anti-cancer drug 1$\alpha$,25-dihydroxyvitamin $D_3$ [1$\alpha$,25(OH)$_2D_3$] and total RNA was purified to measure expression levels of cystatins C and D. 1$\alpha$,25(OH)$_2D_3$ enhanced expression of cystatin D but not cystatin C in HSC-3 cells. Cystatin D was localized in the Golgi apparatus, suggesting that it is secreted and function extracellularly. Then, we prepared recombinant cystatin D by *Brevibacillus* expression system (Takara). After addition of recombinant cystatin D in the medium of HSC-3 cell culture, cell viability was measured by Cell Counting Kit-8 (Dojin). Extracellular cystatin D significantly promoted cell viability. These results suggest that cystatin D did not suppress cell proliferation and its anti-cancer function may be involved in metastasis. (COI:No)

### PP-289

### Elucidation of the mechanism of prostaglandin $E_2$ receptor EP4 that regulates the migration in oral cancer cells.

Kohei Osawa[1,2], Masanari Umemura[1], Rina Nakakaji[1,2], Akane Nagasako[1], Hiroko Nemoto[1], Megumi Uchino[1], Kenji Mitsudo[2], Yoshihiro Ishikawa[1] ([1]*CVRI, Yokohama City Univ. Grad. Sch. of Med.,* [2]*Dept. of OMS, Yokohama City Univ. Grad. Sch. of Med*)

(Introduction)The EP4 prostanoid receptors is one of the four receptor subtypes for $PGE_2$. Little information is available regarding the function and cellular signaling pathway of EP4 in cancer, including oral cancer. In this study, we show that EP4 regulates cell migration and metastasis in oral cancer via the $Ca^{2+}$ signal pathway. (Material and Method)Human-derived tongue squamous cell carcinoma cell lines were used. Changes intracellular $Ca^{2+}$ level were measured by Fura-2. To ablate EP4 or Orai-1, shRNA was induced with lentiviral infection in HSC-3. In animal experiments, we established lung metastasis model mice to evaluate metastatic ability. (Result)The EP4 agonist rapidly increased intracellular $Ca^{2+}$ and increased phosphorylation of $Ca^{2+}$-dependent protein CAMKK2. In contrast, EP4-knockdown significantly reduced the cell migration. Orai1-knockdown also negated the EP4 agonist-induced $Ca^{2+}$ elevation. Immunoprecipitation showed that EP4 was colocalized and formed complexes to both Orai1 and TRPC1. Furthermore, EP4-Knockdown decreased lung metastasis in mice. (Conclusion)EP4 regulates intracellular $Ca^{2+}$ elevation via Orai1 resulting in promoting cell migration of oral cancer. (COI:No)

### PP-290

### Mash1 cell lineage analysis using taste bud organoid culture

Kae Matsuyama[1], Shinji Kataoka[1], Mitsushiro Nakatomi[1], Takashi Toyono[1], Yuji Seta[1] ([1]*Div. Anatomy, Dept. Health Improv., Kyushu Dent. Univ*)

Taste buds are composed of several distinct type cells. We demonstrated that Mash1, a transcription factor, is expressed in subsets of mature taste cells and basal cells in adult taste buds. However, it remains unclear whether Mash1 regulates the differentiation of both type II and III or only type III taste cells. In this study, we explored the cell lineage of Mash1-expressing cells utilizing taste bud organoid culture.
Using newborn mice of Mash1-$Cre^{ERT2}$ and CAG-floxed neo-tdTomato mouse lines, we observed many of tdTomato+ cells coexpressing Car4 (type III cell marker) and a few of them coexpressing gustducin (type II cell marker) in initially developed taste buds. To trace Mash1-expressing cell lineage *ex vivo*, taste organoids were cultured from the transgenic mice. Mash1-expressing cells within organoids were labeled by tdTomato expression after tamoxifen addition to the culture medium. Immunostaining of tamoxifen-treated organoids showed that tdTomato+ cells coexpressed Car4 and a subset of them coexpressed gustducin. These results suggest that Mash1 may play a role in the differentiation of gustducin-expressing type II taste cells in addition to type III taste cells. (COI:No)

### PP-291

### Gene expression profiling of Gustducin-expressing cells in mouse fungiform papillae and circumvallate papillae

Yu Yamada[1,2], Shingo Takai[2], Yu Watanabe[2], Ayana Osaki[2], Yuko Kawabata[2], Asami Oike[2], Ayaka Hirayama[2], Shusuke Iwata[2,3], Keisuke Sanematsu[2,3], Shotaro Nishimura[1], Shoji Tabata[1], Noriatsu Shigemura[2,3] ([1]*Lab. of Functional Anatomy, Graduate school of Biosource and Bioenvironmental Science,Kyushu Univ., Fukuoka, Japan,* [2]*Dept . Oral Neurosci., Kyushu Univ. Grad. Sch. Dent., Fukuoka, Japan,* [3]*R&D Cent. for Five-Sense Devices, Kyushu Univ., Fukuoka, Japan*)

Gustducin is known as a G-protein associated with sweet, umami, and bitter taste responses in taste buds of fungiform papillae (FP) and circumvallate papillae (CV). Previous studies reported that Gustducin-expressing cells had different taste responsivities between FP and CV, suggesting the functional difference of the Gustducin-expressing cells between them. However, it has still been unknown what gene could generate such distinctive cell characteristics of FP and CV. Here, we did transcrptome analysis on the Gustducin-expressing cells in FP and CV by single cell RNA-Seq. Clustering analysis based on the distinct gene expression identified the specific clusters that contained taste cells in FP or CV. Noteworthy, some cell adhesion molecules were found to be expressed specifically in the clusters expressed Tas1rs (sweet or umami taste receptors) or the clusters expressed Tas2rs (bitter taste receptors). It suggested that these adhesion molecules may contribute to the taste quality-specific connections between taste cells and taste neurons. We are going to explore the expression pattern of cell adhesion molecules in taste buds to understand the molecular mechanism in taste cording. (COI:No)

### PP-292

### Leptin suppress sweet responses via Ob-Rb-PI3K-$K_{ATP}$ channel pathway

Ryusuke Yoshida[1], Robert F. Margolskee[2], Yuzo Ninomiya[2,3] ([1]*Department of Oral Physiology, Graduate School of Medicine, Dentistry and Pharmaceutical Sciences, Okayama University,* [2]*Monell Chemical Senses Center,* [3]*Division of Sensory Physiology, Research and Development Center for Five-Sense Device, Kyushu University*)

Leptin suppresses neural and taste cell responses to sweet compounds. Such effect of leptin is mediated by the leptin receptor Ob-Rb, and the ATP-gated $K^+$ ($K_{ATP}$) channel expressed in some TAS1R3-positive taste cells. However, the signal pathway connecting Ob-Rb to the $K_{ATP}$ channel is not elucidated. In this study, we investigate the intracellular transduction pathway mediating leptin's effect in TAS1R3-positive taste cells. Leptin suppressed taste cell responses of TAS1R3-positive taste cells to sucrose. This effect was impaired by coadministration of phosphoinositide 3-kinase (PI3K) inhibitors. In contrast, coadministration of signal transducer and activator of transcription 3 inhibitor, or Src homology region 2 domain-containing phosphatase-2 inhibitor, had no effect on leptin's suppression of sucrose responses. In peeled tongue epithelium, leptin stimulated phosphatidylinositol (3,4,5)-trisphosphate ($PIP_3$) production and phosphorylation of Akt in TAS1R3-positive taste cells, which were suppressed by the PI3K inhibitors. Thus, leptin suppresses sweet responses of TAS1R3-positive taste cells by activation of the Ob-Rb–PI3K–$K_{ATP}$ channel pathway. (COI:No)

### PP-293

### Influences of vagotomy on ingestive behavior in vitamin C deficient rats

Toshiaki Yasuo[1], Takeshi Suwabe[1], Fumihiko Nakamura[1], Noritaka Sako[1] ([1]*Department of Oral Physiology, Division of Oral functional Sciences and Rehabilitation,School of Dentistry, Asahi University*)

It is not well known how manmarian regulate the consumption of the deficient micronutrients. Gut sends nutritional signals to brain, either directly, via the bloodstream, or indirectly, through the activation of the vagus nerve. Our previous studies indicate that vitamin C (VC) deficient rats increase their preferences for VC and citric acid, and that VC deficiency causes changes in peripheral taste sensitivity to acids.
To determine whether vagus nerve is involved in the ingestion of deficient micronutrient or not, we conducted behavioral experiments using Osteogenic Disorder Shionogi rats, which lack the ability to synthesize VC. These rats were assigned to either a vagotomy group (VAGO) or a sham operated group (SHAM). The preference for VC and citric acid in rats was measured before and after VC deficiency by 48-h two-bottle preference test. Preference scores for both VC and citric acid in SHAM significantly increased after VC removal, compared with before, nevertheless preference scores for those in VAGO slightly increased.
Our data suggest that visceral neural information may participate in ingestive behavior during VC deficiency, and that taste information may be important. (COI:No)

### PP-294

### Behavioral and neural responses elicited by thickeners and taste substances

Fumihiko Nakamura[1], Toshiaki Yasuo[1], Takeshi Suwabe[1], Noritaka Sako[1] ([1]*Dept. Oral Physiol., Asahi Univ. Sch. Dent*)

We conducted the behavioral and electrophysiological studies to investigate how the rats express their preference to viscous taste stimuli. As stimuli, 3 thickeners, such as xanthan gum (X), guar gum (G) and pectin (P) (all 0.3%), and 4 basic taste substances, such as 0.1M NaCl, 0.1-0.3M sucrose, 3mM HCl and 1mM quinine HCl were used. In two-bottle preference test, X and G were avoided rather than DW. When thickeners were mixed with one of taste stimuli, the preference percent of these mixtures were different between each combination of thickeners and taste stimuli. In electrophysiological study, chorda tympani nerve responses to mixtures of thickeners and taste stimuli were almost smaller than sum of those to their components, even if used taste stimulus were preferable or not. These results may show that rats express their preference to viscous taste stimuli by using both taste and somatosensory information. (COI:No)

### PP-295

### Postnatal changes of inhibitory synaptic inputs in jaw-closing and jaw-opening motoneurons in rats

Tsuyoshi Noguchi[1], Shiro Nakamura[2], Kiyomi Nakayama[2], Ayako Mochizuki[2], Masanori Dantsuji[2], Yoshiaki Ihara[1], Koji Takahshi[1], Tomio Inoue[2] ([1]*Department of Special Needs Dentistry, Division of Oral Rehabilitation Medicine, Showa University School of Dentistry,* [2]*Department of Oral Physiology, Showa University School of Dentistry*)

In mammals, sucking behavior becomes converted to chewing during postnatal development and neural correlates related to jaw movement can be altered during this period. This study was conducted to investigate developmental changes of miniature inhibitory postsynaptic currents (mIPSCs) in jaw-closing and jaw-opening motoneurons using brainstem preparations obtained from Wistar rats on postnatal day (P)2-5, 9-12, and 14-17. In the masseter motoneurons, the frequency of GABAergic mIPSCs was higher in neurons from P2-5 than in those from P9-12 and P14-17. In addition, both frequency and amplitude of the glycinergic currents were substantially increased with age. On the other hand, the frequency and amplitude of GABAergic components of the digastric motoneurons remained constant throughout the postnatal period, whereas those of glycinergic mIPSCs increased during that period. These results suggest that characteristics related to development of inhibitory synaptic inputs to the masseter and digastric motoneurons differ among motoneuron groups and transmitter types. Such developmental changes may contribute to the transition from sucking to mastication. (COI:Properly Declared)

### PP-296

### Inhibitory effects of daily treadmill running on orofacial hyperalgesia under psychosocial stress conditions in male mice.

Mana Hasegawa[1], Masanori Otake[2], Rantaro Kamimura[2], Isao Saito[2], Noritaka Fujii[1], Kensuke Yamamura[3], Keiichiro Okamoto[3] ([1]*Niigata Univ Grad Sch Med Dent Sci, Div Dent Clin Edu,* [2]*Niigata Univ Grad Sch Med Dent Sci, Div Orthod,* [3]*Niigata Univ Grad Sch Med Dent Sci, Div Oral Physiol*)

The aim of this study is to determine whether daily exercises with treadmill running (TR) can decrease stress-induced hyperalgesia in the orofacial regions in mice. Social defeat stress model was induced in C57BL mice, which was daily exposed to aggressor mice (ICR) for 10 min. Mice (C57BL) were subjected to SDS or sham conditionings for 9 days. After confirmation of mice being stressed by social interaction (SI) tests on Day 10, SDS and sham mice were divided into 2 groups, which are subjected to TR (6 m/min, 30 min/d) or sedentary (non-TR) 30 min after SDS from Day 11-19. On day 20 the time spent on nocifensive behaviors evoked by masseter muscle (MM) injection of formalin were quantified. Data for nocifensive behavioral activities were analyzed separately in the early and late phases. In non-TR group, SDS significantly increased the duration of nocifensive behaviors compared to sham mice in the late phase. In SDS group, TR decreased nocifensive behaviors compared to non-TR group, mainly in the late phase. TR had minor effects in sham group. These findings indicated that daily TR had inhibitory effects on enhanced MM pain-related behaviors under psychosocial stress conditions. (COI:No)

### Poster Presentations

### Day3

(March 30, Tue. 18：30～19：30)

| | |
|---|---|
| **PP-297～PP-301** | Blood, Lymph, Immunity |
| **PP-302～PP-325** | Circulation |
| **PP-326～PP-330** | Respiration |
| **PP-331～PP-334** | Urinary organ, Renal function, Urination |
| **PP-335～PP-348** | Reproduction |
| **PP-349～PP-359** | Endocrine |
| **PP-360～PP-377** | Autonomic nervous system |
| **PP-378～PP-380** | Environmental physiology :<br>Physical fitness and sports medicine |
| **PP-381～PP-390** | Environmental physiology :<br>Nutritional and metabolic physiology, Thermoregulation |
| **PP-391～PP-400** | Environmental physiology :<br>Behabior, Biological rhythm, Sleep |
| **PP-401～PP-403** | Environmental physiology :<br>Stress |
| **PP-404～PP-419** | Gross anatomy |
| **PP-420～PP-424** | Anthropology |
| **PP-425～PP-436** | Pathophysiology |
| **PP-437～PP-440** | Pharmacology |
| **PP-441～PP-446** | Medical education, Medical histology |
| **PP-448～PP-462** | Others |

### PP-297

### Activation, exitus and reconstitution of intra-epithelial lymphocytes (IELs) in mouse small intestine

Masaki Ogata[1], Yui Yamamoto[1], Keiju Kamijo[1], Yuji Owada[2] ([1]*Division of Anatomy and Cell Biology, Tohoku Medical and Pharmaceutical University,* [2]*Department of Organ Anatomy, Tohoku University Graduate School of Medicine*)

In our earlier study in mice, we stimulated intra-epithelial lymphocytes (IELs) by i.p. injection of anti-CD3 mAb to examine the role of IELs in the defense system of the small intestinal epithelium. Injection of mAb stimulated IELs and caused their rapid degranulation. Granzyme B released from their granules induced DNA fragmentation of jejunal epithelial cells. After the stimulation of IELs, the IELs exhibited a series of changes in morphology characteristic of typical dying cells. The fact that IELs with characteristic morphology of the dying cells first increased, then, they decreased in number, and finally they disappeared from the intestinal epithelium suggests that after activation, IELs completely disappeared in the intestinal epithelium without leaving the site. Thus, they should be recognized as "one-shot responders", or, "disposable guards", always present at the front line easily exposed to incoming foreign bodies like bacteria, and once they are somehow stimulated, they can be activated, release their granules, and finally die there. Now we are further investigating the dynamics of the IELs on the villous reconstruction process by morphological approach. (COI:No)

### PP-298

### DGKε deficiency renders mice resistant to LPS-induced inflammatory reaction.

Akiko Ozawa[1], Tomoyuki Nakano[1], Ken Iseki[2], Kaoru Goto[1] ([1]*Dept. Anat. & Cell Biol., Yamagata Univ. Sch. Med.,* [2]*Dept. Emerg. & Crit. Care Med., Fukushima Med. Univ*)

Systemic inflammatory response against bacterial and viral infections occurs through innate immunity activation. Toll-like receptors serves as pathogen receptors, which initiate NFκB signaling pathway. Diacylglycerol kinase (DGK) phosphorylate DG to produce phosphatidic acid (PA). Both of these lipids serve as second messengers to regulate various cellular mechanisms. Of DGK isozymes, DGKε is presumably involved in inflammatory reaction through its substrate specificity to arachidonoyl-DG. However, its functional role in innate immunity remains unknown. In the present study, we investigated how DGKε is implicated in the process of innate immunity using cell culture and animal experiments. In DGKε$^{-/-}$ MEF, nuclear translocation of NFκB p65 subunit was attenuated at 1 h after LPS stimulation. Phosphorylation level of Akt, an upstream mediator of NFκB, was also decreased. In the animal model of LPS-induced endotoxin shock, DGKε-KO mice exhibited increased survival (100%) compared with that of WT mice (60%) after 24 h of injection. Taken together, these findings suggest that DGKε deficiency downregulates NFκB pathway, which renders mice resistant to endotoxin shock. (COI:No)

### PP-299

### Fatty acid-binding protein 3 controls contact hypersensitivity through regulating skin dermal Vγ4$^+$ γδ T cell development

Shuhei Kobayashi[1], Yoshiteru Kagawa[1], Hirofumi Miyazaki[1], Yuji Owada[1] ([1]*Department of Organ Anatomy, Grad. Sch. Med., Tohoku Univ.* )

FABP3 is a cytosolic carrier protein of polyunsaturated fatty acids and regulates cellular metabolism. However, the physiological functions of FABP3 in immune cells and how FABP3 regulates inflammatory responses remain unclear. In this study, we addressed to identify the role of FABP3 in immune cells and the involvement in allergic diseases.
Fabp3$^{-/-}$ mice exhibit a more severe phenotype of CHS accompanied by infiltration of IL-17-producing Vγ4$^+$ γδ T cells that critically control skin inflammation. In *Fabp3*$^{-/-}$ mice, we found a larger proportion of Vγ4$^+$ γδ T cells in the skin, even though the percentage of total γδ T cells did not change at steady state. Similarly, juvenile *Fabp3*$^{-/-}$ mice also contained a higher amount of Vγ4$^+$ γδ T cells not only in the skin but in the thymus when compared with WT mice. Furthermore, thymic DN2 cells expressed FABP3, and FABP3 negatively regulates the development of Vγ4$^+$ γδ T cells in the thymus.
These findings suggest that FABP3 functions as a negative regulator of skin inflammation through limiting pathogenic Vγ4$^+$ γδ T cell generation in the thymus. (COI:No)

### PP-300

### Fatty-acid binding protein 7 modulates liver fibrosis by regulating the anti-inflammatory activation of liver macrophages

Hirofumi Miyazaki[1], Shuhan Yang[1], Yuji Owada[1] ([1]*Dept. of Organ Anatomy, Grad Sch of Med, Tohoku Univ* )

Liver macrophages (Mf) are polarized into pro- and/or anti-inflammatory function by microenvironment changes, and these functions play important roles in the pathophysiology of non-alcoholic steatohepatitis: NASH and fibrosis. However, the mechanism of Mf activation is almost unknown. In this study, we investigated the effect of FABP7 in Mf, an intracellular chaperones of long-chain fatty acids, on NASH and fibrosis.
C57BL/6 (WT) mice were transplanted with WT or *Fabp7*-gene knockout (KO) bone marrow cells, and fed the high-cholesterol diet. Then, histological analysis of liver and cell biological analysis of isolated hepatic Mf were performed.
Histologically, the levels of hepatic steatosis and hepatitis were no significant differences between WT and KO groups. But, the mRNA expressions of profibrotic factors such as *Acta2*, *Tgfb* and *Mertk* in KO mice were lower than WT. In isolated Mf, the mRNA expressions of *Tgfb* and *Mertk* were lower in KO Mf than WT. In addition, *Arg1* and *Pparg*, which show anti-inflammatory function, were reduced in KO Mf.
Taken together, it was suggested that FABP7 is involved in the process of hepatic fibrosis by controlling the anti-inflammatory function of Mf. (COI:No)

### PP-301

### Analysis of chondroitin sulfate in stem cells derived from rat umbilical cord blood

Keiko Nakanishi[1,2], Kyohei Higashi[3], Toshihiko Toida[4], Masato Asai[1] ([1]*Dept Dis Model, Inst Dev Res, ADDC,* [2]*Dept Pediatr, Central Hosp, ADDC,* [3]*Fac Pharm Sci, Tokyo Univ Sci,* [4]*Ctr Preventive Med Sci, Chiba Univ*)

Chondroitin sulfate (CS) is a complex glycosaminoglycan (GAG) with repeating disaccharide units and the major constituent of the extracellular matrix in the central nervous system (CNS) as well as other tissues. There are several CS-disaccharide units with a different number and position of sulfation. Of these, the highly sulfated disaccharides have been shown to bind to several growth factors and to be involved in neurite outgrowth, neural stem cell proliferation, and neural protection. We have previously shown that administration of the stem cell enriched-umbilical cord blood cells (SCE-UCBCs) attenuated HI brain injury in neonatal rat. Wharton jelly, a gelatinous substance within the umbilical cord, is well known to contain mucopolysaccharides such as hyaluronic acid and CS. To know the involvement of CS in physiological function of stem cells, we analyzed the CS-disaccharide units in expanded SCE-UCBCs. CS was detected in vasculatures of rat umbilical cord at E19 by immunohistochemistry. Disaccharide composition analysis revealed that CS was abundant in SCE-UCBCs and the major component of CS in UCBCs was A-unit. (COI:No)

### PP-302

### Morphological consideration of the coronary sinus valve in human heart.

Michiko Naito[1], Kazuyuki Shimada[1,2], Shin Aizawa[1] ([1]*Division of Anatomical Science, Department of Functional Morphology, Nihon University School of Medicine,* [2]*Department of Anatomy, Tokyo Medical University*)

The coronary sinus is a vein that runs through the coronary sulcus, starting with the oblique vein of the left atrium (Marshall's vein) and opening to the right atrium.
Since then, anatomy textbooks have described the valve, but it seems that there are few detailed descriptions of the shape of the valve.
This shape was investigated by using 135 anatomical donated bodies (62 males and 73 females) at Nihon University School of Medicine. As a result of roughly classifying the valves into three types, (1) membranous semilunar valve (73/135 cases), (2) reticulated cord-like material (29/135 cases), and (3) valve formation is unclear (33/135 cases).
From the results, it is thought that the venous blood that returned to the heart and the venous blood that circulated in the heart itself became a large swirling flow when returning to the right atrium, and the coronary sinus ostium was closed by atrial contraction. However, although this valve may work a little to prevent backflow, it is considered to be less effective as a valve. In addition, it was speculated that the catheter may be difficult to insert during cardiovascular angiography depending on the shape of the valve. (COI:No)

### PP-303

### Mechanism of vascular endothelial cell injury caused by Gemcitabine

Mariko Gunji[1], Chika Sawa[2], Shunpei Mukai[3], Minako Akiyama[1], Takashi Takaki[2,4], Dedong Kang[2], Kazuho Honda[2] ([1]*Showa Univ. Grad.school of Med., Dept.Anatomy,* [2]*Showa Univ. School of Med., Dept.Anatomy,* [3]*Showa Univ. Grad,school of Med., Dept.Pathology,* [4]*Showa Univ., Sec. Electron Microscopy*)

Gemcitabine(GEM) is an anticancer agent though its DNA elongation inhibition. Renal toxicity has been reported, but its mechanism is still unknown. In this study, we investigated the mechanism of the endothelial injury by GEM using HUVEC.
GEM(0.05uM to 1mM) was added to HUVEC during the growth and confluent phase for 2 days. MTT assay, immunocytochemistry, lectin staining and RT-PCR were performed to analyze the endothelial glycocalyx(GCX).
The GEM sensitivity for HUVEC was about 1/20,000 in the confluent phase compared to the growth phase. The cell shrinkage and intercellular space dilation were observed under the GEM 50uM addition in confluent phase. There were no significant changes in PECAM expression and WGA-lectin binding, but decreases in terminal α2,6-sialic acid(SNA) binding of GCX and mRNA expression of its transferase enzyme(ST6Gal1). Meanwhile, the mRNA expression of proinflammatory cytokines, such as IL-6 and IL-1β were increased.
It was suggested that GEM may damage the vascular endothelium due to inhibition of the GCX synthesis pathway and production of proinflammatory cytokines, in the different way from the anticancer effect by inhibiting DNA elongation. (COI:No)

### PP-304

### Cluster networks consisting of early afterdepolarization-evoked myocytes could cause reentrant arrhythmias in human ventricular tissue

Takao Shimamoto[1], Kunichika Tsumoto[2], Yasutaka Kurata[2], Akira Amano[1] ([1]*Department of Bioinformatics, College of Life Sciences, Ritsumeikan University,* [2]*Department of Physiology, Kanazawa Medical University*)

Arrhythmias in patients with long QT syndrome can be triggered by early afterdepolarizations (EADs), which transiently depolarize during action potential repolarization phase. Although numerous experimental and theoretical studies have elucidated mechanisms of EAD development in the cell level, EAD-mediated arrhythmogenesis mechanism in the tissue level remain to be elucidated. To examine how EADs locally evoked in ventricular tissue lead to arrhythmias, we constructed a mono-domain sheet model (6cm×6cm) consisting of 360,000 human ventricular myocardial units and performed computer simulations of AP propagation. Reentrant excitation wave, i.e., arrhythmias, occurred only when EAD-forming unit area (EAD cluster) were located at the center of myocardial sheet and occupied 80-90% of the myocardial sheet area. Furthermore, we found that when EAD clusters, consisting of about 30-40% of the total myocyte units, were discontinuously distributed in the myocardial sheet, the reentrant excitation wave can be triggered. These results suggested that not only the number of myocytes evoking EADs but also the EAD cluster distributed pattern is involved in fatal arrhythmia onset. (COI:No)

### PP-305

### Reduced gap-junction coupling facilitates the development of subcellular $Na^+$ channel expression changes-mediated ventricular arrhythmias

Kunichika Tsumoto[1], Takashi Ashihara[2], Takao Shimamoto[3], Narumi Naito[3], Akira Amano[3], Yoshihisa Kurachi[4], Yuichi Kuda[1], Mamoru Tanida[1], Yasutaka Kurata[1] ([1]*Dept Physiol, Kanazawa Med. Univ,* [2]*Dept Med Info and Biomed Eng , Shiga Univ Med Sci,* [3]*Coll Life Sci, Ritsumei Univ,* [4]*Dept Pharmacol, Grad Sch Med, Osaka Univ*)

Cardiac voltage-gated sodium ($Na^+$) channels play key roles in the action potential initiation and propagation. Its functional abnormalities are associated with fatal arrhythmia developments in the heart. We have recently reported simulation results that regional expression decreasing of $Na^+$ channel in cardiomyocytes can lead to fatal arrhythmias in Brugada syndrome. Here, we investigated the effect of reduced gap-junction coupling on the occurrence of proarrhythmic events in the myocardial strand model with specifically decreased $Na^+$ channel expression on the lateral membrane of each myocyte by extending our previous *in silico* study. We first estimated the gap-junction coupling and subcellular distributions of $Na^+$ channel conductance using electrophysiological experimental data from the right ventricular outflow tract in the actual human heart. And then, we found that the occurrence of subcellular $Na^+$ channel expression changes-mediated ventricular arrhythmias was accelerated with reducing gap-junction coupling. Reduced gap-junction coupling, as well as subcellular $Na^+$ channel expression changes, might be responsible for life-threatening arrhythmias in Brugada syndrome. (COI:No)

### PP-306

### Cell fusion and irregular nuclear division in multinuclear cardiac progenitor cells

Ryo Fukunaga[1], Mariko Omatsu[1], Hiroshi Matsuura[1] ([1]*Dept Physiol, Shiga Univ Med Sci, Otsu, Japan*)

The adult mammalian heart contains several kinds of cardiac stem or progenitor cells. Atypically-shaped cardiomyocytes (ACMs) are cardiac progenitor cells derived from mouse heart that spontaneously develop into beating cells. Although ACMs do not appreciably proliferate, most of those cells possess multiple nuclei. In this study, we examined the process of forming multiple nuclei in ACMs. We observed that ACMs fused with each other resulting in a formation of the multinuclear cells with complicated shapes. We also observed peculiar morphology of the nuclei, such as irregular shapes, clusterization, and DNA bridges that occur as a result of failure of sister-chromatid disjunction in anaphase using high-magnification microscope. In addition, immunostaining analyses confirmed that lamin B and lamin A/C were expressed even in nuclei of irregular shapes. These results suggest that ACMs possess multiple nuclei due to not only cell fusion but also nuclear division without cytokinesis. Such incomplete division of nucleus may be one of the obstacles to progress cell division in these cells. (COI:No)

### PP-307

### Arterial pressure and heart rate decreased during cold air caloric test in patients with vertigo.

Kunihiko Tanaka[1], Kayoko Kabaya[2], Akihiro Sugiura[3], Shinichi Esaki[2], Meihou Nakayama[2], Shinichi Iwasaki[2] ([1]*Grad Sch Health Med, Gifu Univ Med Sci,* [2]*Dept Oto Laryngo, Nagoya City Univ,* [3]*Dept Radio Tech, Gifu Univ Med Sci*)

Caloric test has been used to observe the semicircular canal function. In the present study, arterial pressure (AP) and heart rate (HR) were measured continuously during the caloric test with cold air irrigation at 15 °C in 22 outpatients with vertigo. Mean AP and HR or pulse rate were analyzed from the AP waveform instantaneously. Maximum slow phase velocity (SPV) of nystagmus was also analyzed after irrigation. AP and HR significantly decreased at the onset of air irrigation in, both, the left and right ears. Decrease in HR during the right ear irrigation was significantly greater than that during the left ear irrigation. The auricular branch of the vagus nerve near the external auditory meatus might be stimulated by cold air irrigation because the right vagus nerve predominantly controls the sinoatrial node. Changes in AP were not correlated with SPV. However, changes in HR during irrigation showed positive correlation with SPV for, both, the left and right ears. Patients with higher SPV showed greater decrease in HR. Cold air irrigation might decrease HR via the vagus nerve and the semicircular canals might suppress this decrease in HR. (COI:No)

### PP-308

### Interaction between KCNK2 and actin-tropomyosin skeletons

Yosuke Okamoto[1], Yasutoshi Fukuda[1], Kyoichi Ono[1] ([1]*Department of cell physiology, Akita University Graduate School of Medicine* )

KCNK2 is a member of two-pore domain potassium channel family. Most of ion channels are large protein, exceeding 200 kDa in molecular size, whereas size of KCNK2 equivalents to only 90 kDa totally unless other proteins bind to it. Here, we hypothesized that unknown proteins bind to the cannel and regulate its functions. In the patch-clamp conditions, KCNK2-like outward rectifier currents were recorded in ventricular, atrial, and pulmonary vein cardiomyocyte of rat. After Immunoprecipitation by anti-KCNK2 antibody from rat heart, CBB staining detected ~ 60 and ~ 35 kDa protein bands on the SDS-PAGE gel. Mass spectrometry identified peptide sequences of each band as a mitochondrial protein and an actin adaptor protein, respectively. Interaction with KCNK2 of them were confirmed by immunoblotting in rat, mouse and human heart samples. Immunocytochemistry revealed that these proteins co-localize with KCNK2 in near-plasma membrane. These results suggest that intracellular organelle and cytoskeletons interacts with KCNK2, and may influence on its functions. (COI:No)

### PP-309

### Effect of Sarcomere Length Change During Isovolumic Relaxation Phase on Hemodynamics

Kota Kishida[1], Akira Amano[1] ([1]*Dept Bioinfo, Grad Sch Life Sci, Ritsumeikan Univ, Shiga, Japan*)

Force velocity relation (FVR) is known to be one of the basic characteristics of the ventricular myocyte where the muscle contraction force decreases according to the increase in muscle shortening velocity. The left ventricle (LV) twist changes during contraction phase, but untwists during isovolumic relaxation phase (IVRP) associated with sarcomere length (SL) change. In addition, the timing of LV untwisting and SL change differ depending on the direction of twisting and layer / position of LV. Although the effect of the SL change on the hemodynamic is not clear, we can analyze the effect by using the mathematical model. In this study, we used a circulation model that can reproduce SL change during IVRP and compared with a circulation model whose SL do not change during IVRP. The model consists of Negroni & Lascano2008 cardiac contraction model, blood vessel model, LV physical model, and SL change model derived from reported data by Rodriguez. The results suggested that SL change during IVRP contributed in the shortening of IVR time by rapid force drop due to FVR. (COI:No)

### PP-310

### Chronic effects of ivabradine on baroreflex dynamic characteristics in rats with myocardial infarction.

Toru Kawada[1], Nan Li[1], Masaru Sugimachi[1] ([1]*Dept Cardiovasc Dynamics, National Cerebral and Cardiovascular Center*)

Ivabradine is a selective bradycardic agent that reduces heart rate by blocking hyperpolarization-activated cyclic nucleotide-gated (HCN) channels. Since HCN channels play various roles in controlling the excitability of the neurons, we examined whether chronic administration of ivabradine modifies the baroreflex dynamic characteristics in rats with myocardial infarction (MI). From two weeks after MI, ivabradine was administered at 10 mg/kg/day in drinking water. Six-week later, the baroreflex function was estimated in ivabradine-treated and untreated MI rats under anesthesia (n = 7 each). The neural arc transfer function from carotid sinus pressure to efferent sympathetic nerve activity approximated a mathematical model with the corner frequency for the derivative characteristics ($f_{C1}$), the corner frequency for the high-cut characteristics ($f_{C2}$), and pure dead time ($L_N$). There was no significant difference in $f_{C1}$ (0.076 ± 0.010 vs. 0.088 ± 0.012 Hz), $f_{C2}$ (0.814 ± 0.104 vs. 0.834 ± 0.133 Hz), or $L_N$ (0.090 ± 0.013 vs. 0.107 ± 0.011 s). Chronic administration of ivabradine does not significantly affect the baroreflex dynamic characteristics in rats with MI. (COI:No)

### PP-311

### Establishing an artificial heart model on a chip by using cardiomyocytes differentiated from human induced pluripotent stem cells

Mengxue Wang[1], Yun Liu[1], Kenji Naruse[1], Ken Takahashi[1] ([1]*Dept Cardiovasc Physiol, Grad Sch Med Dent Pharm Sci, Okayama Univ, Okayama, Japan*)

Heart disease is the most common cause of death worldwide. Although disease models using animals, such as rodents, are often used in research, they do not accurately recapitulate the human pathophysiology. Hence, we have established a human heart model on a two-channel organ chip. We seeded human induced pluripotent stem cells and human gingival fibroblast cells in the top channel, and human umbilical vein endothelial cells in the bottom channel. When the cardiac differentiation protocol was applied on the chip, the intracellular calcium level spontaneously fluctuated under fluorescent microscopy. At day 20 of cardiac differentiation, cardiomyocytes synchronously contracted. Therefore, a model of a spontaneously contracting heart from human cells has been developed. We will further improve the efficiency of cardiac differentiation and develop a disease model on a chip. (COI:No)

### PP-312

### Optimization of the cardiac differentiation of human pluripotent stem cells

Yun Liu [1], Mengxue Wang[1], Kenji Naruse[1], Ken Takahashi[1] ([1]*Dept Cardiovasc Physiol, Grad Sch Med Dent Pharm Sci, Okayama Univ, Okayama, Japan*)

Differentiation of induced pluripotent stem cells (iPSCs) into cardiomyocytes is necessary in heart disease research and drug development. However, differentiation conditions still need to be optimized. Hence, we compared the conditions of iPSC types (201B7 and 1383D6), densities ($3 \times 10^4$–$10^5$ cells/well [9.5 cm$^2$]), extracellular matrix types, and coculture conditions. The extracellular matrix was coated by laminin-511 (0.5–1.0 μg/ml) and Matrigel (1:30 dilution). For coculture conditions, we used human gingival fibroblasts (HGFs). In both 201B7 and 1383D6, cell adhesion was more robust in Matrigel than in laminin-511. At day 20, 201B7 monoculture successfully showed spontaneous contraction. When cocultured with HGFs, 201B7 underwent cardiac differentiation. In the HGF/iPS ratio range between 0 and 12, 2 showed the highest rate of cardiac differentiation. Meanwhile, 1383D6 did not show spontaneous contraction even with the coculture conditions. Of the conditions examined, 201B7 iPSCs, seeded with the HGF/iPS ratio of 2, on Matrigel is the most efficient in obtaining spontaneous contraction of cardiomyocytes. (COI:No)

### PP-313

### Nuclear heterogeneity in multinucleated ventricular myocytes in mice

Xinya Mi[1], Ryo Fukunaga[1], Wei-Guang Ding[1], Hiroshi Matsuura[1], Mariko Omatsu[1] ([1]*Department of Physiology, Cell Physiology, Shiga University of Medical Science*)

The mammalian heart is one of the organs unable to actually regenerate after birth. The turnover of the cells through proliferation of resident cardiomyocytes is estimated to occur at a rate of ~1.3 % per year in mice. It has been reported that the gene replication without cell division occurs peaks on the 4th day after birth, and by the 10th day, almost all cardiomyocytes become multinuclear or polyploid in mice. In the present study, we examined the heterogeneity of nuclei in mouse ventricular myocytes. Ventricular myocytes isolated from adult mice mostly contain two or more nuclei. Interestingly, we often found that nuclei with different morphologies existed in the same cardiomyocyte, suggesting that the nucleus was unevenly divided. The ratio of such irregular shaped nuclei was higher in the cells with odd number of nuclei. Immunostaining analysis confirmed that nuclear lamina proteins lamin A/C and lamin B were expressed on the envelope of the irregular nuclei. The results suggest the possibility that the abnormal division of nucleus makes one of the obstacles to progress cell division in the ventricular myocytes in the adult heart. (COI:No)

### PP-314

### Blood pressure in rats is regulated by signal transducer and activator of transcription 3 in the amygdala central nucleus

Hidefumi Waki[1], Keisuke Tomita[2], Kei Tsukioka[1], Van Thu Nguyen[1], Sabine Gouraud[3], Ko Yamanaka[1] ([1]*Dept Physiol, Grad Sch Health & Sports Sci, Juntendo Univ,* [2]*Fac Med Sci, Teikyo Univ of Sci,* [3]*Dept Biol, Fac of Sci, Ochanomizu Univ*)

Chronic restraint stress in rats increases blood pressure and decreases the expression of the transcription factor signal transducer and activator of transcription 3 (STAT3) gene in the amygdala. To examine the role of change in STAT3 expression in stress-induced hypertension, the localization of STAT3 in the amygdala and its involvement in cardiovascular system regulation were studied in male Wistar rats. STAT3 protein localization in both neurons and astrocytes was studied using immunohistochemistry. Subsequently, small interfering RNAs (siRNAs) were used to silence STAT3 expression in the amygdala to clarify its role in regulating blood pressure. Phosphorylated STAT3 was identified in both the neurons and astrocytes of the amygdala, including in the central nucleus of the amygdala (CeA). Microinjection of siRNAs targeting STAT3 in the CeA was found to affect blood pressure levels. These results suggest that altered STAT3 expression in the amygdala is related to stress-induced high blood pressure in rats. (COI:No)

### PP-315

### Roles of nitric oxide-mediated signal transmissions in regulating bladder vascular contractility: A therapeutic target of tadalafil

Retsu Mitsui[1], Hidekazu Tanaka[1], Hikaru Hashitani[1] ([1]*Dept Cell Physiol, Grad Sch Med Sci, Nagoya City Univ*)

Tadalafil, an inhibitor of cGMP-specific phosphodiesterase 5 (PDE5), induces improvement of bladder storage symptoms that could be attributable to its vasodilatory actions. Here, effects of tadalafil on contractility or $Ca^{2+}$ dynamics in different vascular segments of the bladder were investigated. Changes in the contractility of rat bladder vasculature were examined using a video tracking system, while $Ca^{2+}$ dynamics in pericytes were visualised by intracellular $Ca^{2+}$ imaging using GCaMP reporter mice. Tadalafil diminished electrical field stimulation (EFS)-evoked sympathetic vasoconstrictions in the rat submucosal arterioles and feeding arteries in a manner sensitive to nNOS inhibitor L-NPA or non-selective NOS inhibitor L-NA. In mouse bladder pre-capillary arterioles, EFS induced transient reductions in pericyte $Ca^{2+}$ level that were shortened by L-NPA and abolished by L-NA. nNOS-positive nerves were found around the vessels. Thus, tadalafil enhances the nitrergic nerve-mediated inhibition of sympathetic vasoconstrictions in bladder arterioles/arteries. Intracellular $Ca^{2+}$ dynamics in pericytes of pre-capillary arterioles appear to be suppressed by both neuronal and endothelial NO. (COI:No)

### PP-316

### Improvement effect of vascular endothelial function by trigonelline in *Raphanus sativus* cv. Sakurajima Daikon

Katsuko Kajiya[1], Maho Sasaki[1], Yuri Nonoshita[1], Takashi Kajiya[2], Yuji Minami[1] ([1]*Faculty of Agriculture, Kagoshima University*, [2]*Tenyoukai Central Hospital*)

We revealed that trigonelline, an active constituent of Sakurajima Daikon, improved nitric oxide (NO) production in vascular endothelial cell cultures in previous study. However, the mechanisms of action trigonelline in the human body has not yet been clarified. Sakurajima Daikon, 170g/day (trigonelline; 61.2 mg), was given to 14 healthy volunteers (7 males, 7 females, age 33.9±6.7 years) for 10 days. Peripheral blood samples were collected and the concentrations of trigonelline general biochemical tests were measured. Flow-mediated dilation (FMD) and brachial-ankle pulse wave velocity (baPWV) were compared before and after taking Sakurajima Daikon for 10 days. Trigonelline was not detected in any of the blood samples before Sakurajima Daikon administration. However, 1.6±0.2 mg/mL of trigonelline was detected after taking Sakurajima Daikon for 10 days. Significant improvement in %FMD (6.7±1.6% vs 9.4±1.9%, p=0.0016) was observed. There was no change (1184.4±201.4 vs 1179.8±197.8, p=0.4) in baPWV. Trigonelline, an active constituent of Sakurajima Daikon, improved NO production and %FMD. As a functional food, Sakurajima Daikon may improve vascular endothelial function. (COI:No)

### PP-317

### Effect of intragastric administration of ninjin'yoeito on cerebral blood flow in anesthetized mice

Nobuhiro Watanabe[1], Kaori Iimura[1], Harumi Hotta[1] ([1]*Dept. Auton. Neurosci., Tokyo Metro. Inst. Gerontol*)

Maintenance of cerebral blood flow (CBF) is important for cognitive functions. We examined the effect of intragastric administration of ninjin'yoeito (NYT) on CBF in mice. Male C57BL/6J mice were anesthetized with urethane and artificially ventilated. CBF in the neocortex was measured with laser-speckle contrast imaging, for 10 min before and 60 min after administration begins. NYT solution (1 g/kg) or vehicle (distilled water; DW) was administered over 5 min via an intragastric catheter. CBF decreased after DW, starting from 20 min onward. In contrast, CBF did not change after NYT. Systemic arterial pressure recorded from a femoral artery was unchanged by either solution. CBF after NYT was not affected by vagotomy, but was decreased by additional atropine. Based on the above results, CBF response during cutaneous brushing was examined within 20 min after administration. CBF during brushing was unchanged by DW, but was enhanced by NYT compared to that before administration. These results suggest NYT prevents from CBF decrease, via cholinergic activation independently of vagal activity, and enhances CBF response to somatic stimuli. This study was funded by Tsumura & Co. (COI:Properly Declared)

### PP-318

### Effects of NOX4-induced ROS on single cell mechanics in mouse ventricular cardiomyocytes.

Keiko Kaihara[1], Kenji Naruse[1], Gentaro Iribe[1,2] ([1]*Okayama University Graduate School of Medicine, Dentistry and Pharmaceutical Sciences, Cardiovascular Physiology*, [2]*Asahikawa Medical University, Department of Physiology*)

It is known that myocardial stretch increases reactive oxygen species (ROS) production derived from NADPH oxidase 2 (NOX2). We have previously reported that not only NOX2 but NOX4 is also involved with stretch-induced increase in ROS production. However, role of NOX4-derived ROS on physiological cellular function is not known.

To investigate the effects of NOX4-derived stretch-induced ROS in cellular mechanics, ventricular cells were enzymatically isolated from either 8~12 week old mice (WT) or NOX4 knock out (KO) mice hearts. Cells were exposed to 8~10% axial stretch using computer-controlled piezo-manipulated carbon fibers, attached to both cell ends.

KO or pharmacological NOX4 inhibition by GKT136901 abolished stretch-induced increase in ROS production and significantly reduced the slope of end-systolic force-length relation curves, which is an index of cellular contractility. Applying $H_2O_2$, mimicking stretch-induced ROS, without stretch significantly increased twitch force both in WT and KO groups. The results indicate that NOX4-derived stretch-induced ROS plays a role in keeping cellular contractility during stretch. (COI:No)

### PP-319

### CAVI as a predictor of hypertensive disorders of pregnancy

Shin-ichiro Katsuda[1], Kaori Kamijo[2], Fumihiko Yoshikawa[2], Yasuhiro Netsu[2], Tsuyoshi Shimizu[3], Koji Shirai[4] ([1]*Department of Cellular and Integrative Physiology, Fukushima Medical University School of Medicine, Japan*, [2]*Suwa Maternity Clinic*, [3]*Shimizu Institute of Space Physiology, Suwa Maternity Clinic*, [4]*Seijinkai Mihama Hospital*)

In hypertensive disorders of pregnancy (HDP), endothelial dysfunction could induce increased vascular resistance and blood pressure (BP), which is responsible for maternal and fetal complications. We studied whether or not cardio-ankle vascular index (CAVI), a pressure-independent index of arterial stiffness is useful for predicting HDP. We examined 197 normal pregnant women and 21 pregnant women with HDP older than 35 years who received a routine prenatal checkup at Suwa Maternity Clinic. BP and CAVI were measured every 4 weeks (±1W) from 11-14W of gestation until 37W. Systolic (SBP) and diastolic (DBP) blood pressures were almost constant in normal pregnant women (Norm) and increased slightly at 37W of gestation. CAVI decreased significantly at 19W compared with that at 11-14W and then kept relatively low value. In the HDP group, SBP and DBP were significantly greater than those in the Norm group. CAVI did not fall at 19W and showed a significantly higher value compared with that in the Norm Group. In conclusion, arterial elasticity is not ameliorated during pregnancy in the HDP group. CAVI may be a useful predictor of HDP. There was no COI to declare in the present study. (COI:No)

### PP-320

### Characterization and Expression Regulation of Inward-rectifier $K^+$ Channel in HL-1 Mouse Atrial Myocytes

Yuhichi Kuda[1], Yasutaka Kurata[1], Takayuki Ikeda[2], Mamoru Tanida[1], Kunichika Tsumoto[1], Toshishige Shibamoto[1], Hideto Yonekura[2] ([1]*Dept Physiol2, Sch Med, Kanazawa Med Univ, Japan*, [2]*Dept Biochem2 , Sch Med, Kanazawa Med Univ, Japan*)

**Aim:** HL-1 mouse atrial myocytes exhibit automaticity, which may depend on the inwardly-rectifying $K^+$ channel current ($I_{K1}$) density. We examined expression and characteristics of $I_{K1}$ channels in HL-1 cells and whether automaticity can be controlled by genetic regulations.

**Methods:** We determined Kir channel subtypes expressed in HL-1 cells by qPCR. Using whole-cell patch-clamp and fluorescence imaging of membrane potentials, we analyzed dynamics of $I_{K1}$, effect of the $I_{K1}$ blocker $Ba^{2+}$, and effect of replacing extracellular $Na^+$ with TMA. Kir2.1 was overexpressed or knocked down by genetic manipulation.

**Results:** 1) Kir2.1 and its variant were expressed in HL-1 cells. 2) Automaticity was promoted by $Ba^{2+}$. 3) Inward $I_{K1}$ current was attenuated by hyperpolarization, which was abolished by replacing $Na^+$ with TMA. 4) Kir2.1 overexpression led to hyperpolarization and abolished automaticity, while its knockdown depolarization without automaticity.

**Conclusions:** Kir2.1 channels are expressed in HL-1 cells as in mouse cardiomyocytes, with $I_{K1}$ attenuated by hyperpolarization. Whether automaticity emerges or not depends on $I_{K1}$ density. HL-1 automaticity can be regulated by genetic regulations of $I_{K1}$. (COI:No)

### PP-321

### Mislocalization of junctophilin-2 in the ventricular cardiomyocytes of diabetic cardiomyopathy model mice

Yoshinori Mikami[1], Masanori Ito[1], Keiichiro Tomida[1], Daisuke Oshima[1], Satomi Akahane[1] (*[1]Dept Physiol, Fac Med, Toho Univ*)

Left ventricular (LV) diastolic dysfunction is one of the earliest symptoms in diabetic cardiomyopathy. The defective $Ca^{2+}$ signaling has been considered as the cause of diastolic dysfunction. However, the underlying mechanism has not yet been clarified. We aimed to elucidate the mechanism of the $Ca^{2+}$ signaling defects in diabetic cardiomyopathy. In the streptozotocin (STZ)-induced type 1 diabetic model mice 4 weeks after STZ injection (STZ-4W), diastolic function was impaired without reduction of ejection fraction or fibrosis. In the ventricle of STZ-4W mice, the protein expression level of junctophilin-2 (Jph2) was significantly lower, although expression levels of Cav1.2, RyR2, and SERCA2 were the same as those of control mice. In the ventricular myocytes of STZ-4W mice, the localization of Cav1.2 and Jph2 to the dyad junction was impaired despite the normal localization of cardiac troponin C. These results suggest that the impairment of the localization of Cav1.2 and Jph2 to the dyad junction is already in progress in the early stage of diabetic cardiomyopathy and predisposes to $Ca^{2+}$ signaling defects underlying LV diastolic dysfunction. (COI:No)

### PP-322

### Coronary nitric oxide bioavailability is reduced by xanthine oxidase and nitrosative stress on a high fat diet but restored by high intensity exercise in senescence-accelerated mice

James Pearson[1,2], Jennifer Ngo[1], Connie Ow[1], Takashi Sonobe[1], Hirotsugu Tsuchimochi[1], Mark Waddingham[3], Cheng-kun Du[1], Misa Yoshimoto[4] (*[1]Dept Cardiac Physiol, National Cerebral & Cardiovascular Center, [2]Dept Physiol, Monash Univ, Australia, [3]Dept Adv Med Res Pulm Hypertension, National Cerebral & Cardiovascular Center, [4]Dept Physiol, Nara Women's Univ*)

Vascular aging and systemic endothelial dysfunction has recently been shown to be exacerbated in female senescence-accelerated (SAMP8) mice after exposure to a western diet relative to senescence-resistant (SAMR1) mice. Here we investigated if excess oxidative/nitrosative stress due to a high fat diet (HFD, 16 weeks with 1% NaCl in the drinking water) causes coronary endothelial dysfunction in male SAMP8 mice. Synchrotron microangiography of coronary microvessel function was performed in vivo at 24 weeks of age. In contrast to SAMR1 mice on a normal chow diet (ND), SAMP8 mice in both ND and HFD groups showed variable responses to acetylcholine and nitric oxide bioavailability was limited as coronary perfusion was reduced following blockade of prostanoids and calcium-activated K channels. Further, acetylcholine evoked constriction post-blockade in SAMP8 HFD mice. Immunoblotting and metabolome analysis revealed that arginine bioavailability and eNOS/PKG signaling were maintained in SAMP8 HFD mice, but myocardial 3-nitrotyrosine and XO activity were increased. High intensity exercise training restored endothelium-dependent dilation in SAMP8 HFD mice. (COI:No)

### PP-324

### Effects of Epac activator on phosphorylation of myofilament proteins and contractility in cardiac muscle.

Yoshiki Ohnuki[1], Kenji Suita[1], Satoshi Okumura[1] (*[1]Dept Physiol, Tsurumi Univ Sch Dent Med*)

To elucidate the contribution of exchange protein activated by cAMP (Epac), a PKA-independent cAMP effector, to cardiac myofilament response to $\beta$-AR stimulation, we examined the phosphorylation status of myofilament proteins in the isolated hearts of mice perfused in the presence or absence of 8CPT-AM, an Epac-specific cAMP analogue, plus H-89, a PKA inhibitor. Pharmacological activation of Epac with 8CPT-AM significantly increased phosphorylation level of myosin regulatory light chain (RLC) without any changes in the phosphorylation of troponin I or myosin binding protein C. In addition, the 8CPT-AM treatment significantly increased phosphorylation of myosin phosphatase target subunit (MYPT), a negative regulator of myosin light chain phosphatase (PP1c$\delta$). We also observed that $Ca^{2+}$ sensitivities of force and ATPase activity as well as tension cost (ATPase activity/force) were significantly increased in skinned myocardium by the 8CPT-AM treatment. These results suggest that Epac activation by $\beta$-AR stimulation increases $Ca^{2+}$ sensitivity and tension cost in cardiac myofilaments by promoting MYPT phosphorylation and subsequent increase in phosphorylation level of RLC. (COI:No)

### PP-325

### Different contributions of SK and IK channels to endothelium-dependent hyperpolarization in gastroepiploic arteries of septic rats

Hiromichi Takano[1], Hiroyuki Nakamori[1], Tomonori Hattori[2], Hikaru Hashitani[1] (*[1]Nagoya City Univ, Gra Sch Med, Cell Physiol, [2]Nagoya City Univ, Gra Sch Med, Advancing Acute Med*)

In rat sepsis models, time-dependent impairments of endothelium-derived NO (EDNO) mediated vasorelaxations are reported, while changes in the endothelium-derived hyperpolarization (EDH) remains to be elucidated. Here, the relative contributions of SK and IK to EDH in LPS-induced sepsis model of rats were investigated. Septic rats were established by LPS injection. Experiments were carried out on the same day (day 0), the next day (day 1), the 3rd (day3) and the 6th day (day6). Short segments of right gastroepiploic arteries that had been pretreated with nifedipine and nitro L-arginine were impaled by a sharp microelectrode to record changes in the membrane potential of arterial smooth muscle cells. In control rats, acetylcholine (ACh) induced EDH consisted of apamin (SK inhibitor) sensitive- and TRAM-34 (IK inhibitor) sensitive-components. The EDHs on day 3 were diminished compared to control responses but restored on day 6. However, the EDHs were largely attenuated by apamin, while the TRAM-34 sensitive components were hardly existed. Thus, LPS induced sepsis impair the EDH in gastroepiploic artery of the rat, predominantly by the suppression of IK component. (COI:No)

### PP-326

### Morphology and function of GNAT3-immunoreactive chemosensory cells in the rat pharynx

Yoshio Yamamoto[1], Takuya Yokoyama[2], Nobuaki Nakamuta[1] (*[1]Lab. Vet. Anat., Iwate Univ., [2]Dep. Anat. [Cell Biol], Iwate Med. Univ*)

Solitary chemosensory cells and chemosensory cell clusters are distributed in the airway. In the present study, morphology and reflexogenic function of the chemosensory cells in the rat pharynx were examined using immunofluorescence for GNAT3 and electrophysiology. In the nasopharynx, GNAT3-immunoreactive solitary chemosensory cells were distributed, and were barrel-like or slender in shape with long lateral processes. Furthermore, chemosensory cell clusters containing GNAT3-immunoreactive cells were also distributed in the nasopharynx, oropharynx and laryngopharynx. In the chemosensory cell clusters, GNAT3-immunoreactive cells gathered with SNAP25-immunoreactive cells. GNAT3-immunoreactive chemosensory cells were in close contact with a few SP-, CGRP-and P2X3-immunoreactive nerve endings. Electrophysiologically, application of 10 mM quinine hydrochloride (QHCl) to the pharyngeal mucosa induced ventilatory depression. The QHCl-induced reflex was diminished by bilateral section of the glossopharyngeal nerve. The present results indicate that the pharyngeal chemosensory cells are one of the important sensors for respiratory depression. (COI:No)

### PP-327

### In vitro generation of goblet cell metaplasia model using iPS cell-derived airway epithelium

Susumu Yoshie[1], Masao Miyake[1], Akihiro Hazama[1] (*[1]Department of Cellular and Integrative Physiology, Fukushima Medical University, Fukushima, Japan*)

[Background] Goblet cell metaplasia caused by asthma and habitual cigarette smoking leads to excessive mucus production and airway obstruction. However, the pathogenic mechanism of goblet cell metaplasia has not yet been fully elucidated. The aim of this study is to generate goblet cell metaplasia model using iPS cell-derived airway epithelium in order to elucidate the pathogenic mechanism of goblet cell metaplasia.

[Methods] We generated airway epithelium via spheroid formed from iPS cells based on serum-free conditions. Goblet cell metaplasia model was generated from iPS cell-derived airway epithelium by the use of cigarette smoking solution.

[Results] Airway epithelium generated from iPS cells expressed airway epithelium markers and had functional characteristics such as ciliary movement and $Cl^-$ transport. Furthermore, iPS cell-derived airway epithelium treated with cigarette smoking solution strongly expressed goblet cell markers. Mucin-positive cells were also appeared.

[Conclusions] We succeeded in the generation of goblet cell metaplasia model using iPS cell-derived airway epithelium. (COI:No)

### PP-328

### Phox2b-positive neurons located in the solitary nucleus is essential to trigger sucking

Makito Iizuka[1], Keiko Ikeda[2], Hiroyuki Igarashi[3], Kazuto Kobayashi[4], Hiroshi Onimaru[1], Masahiko Izumizaki[1] (*[1]Dept Physiol, Showa Univ Sch Med, Tokyo, Japan, [2]Murayama Med Ctr, Tokyo, Japan, [3]Dept of Physiol and Pharmacol, Western Univ, London, Canada, [4]Dept Molecular Genetics, Inst Biomed Sci, Fukushima Med Univ Sch Med, Fukushima, Japan*)

We have shown the photo-stimulation of the Phox2b-positive neurons from the dorsal skull causes sucking movement in a neonatal transgenic rat in which Phox2b-positive neurons expressed one of channelrhodopsin variants; ChRFR(C167A). To clarify precise location of the Phox2b-positive neurons involving in the sucking, areas showing sucking-related rhythmic membrane potential changes were explored using voltage sensitive dye. The depolarizing areas synchronized to peak of the sucking motor bursts were mainly localized within the solitary nucleus (NTS) and area postrema (AP). The Phox2B-positive neurons also distributed in AP and NTS. Therefore, we examined the effects of electrical coagulation of these areas on sucking. When AP was electrically coagulated, the photo-evoked sucking was largely depressed just after the coagulation. However, after 1-2 hours, the sucking rhythm was largely recovered. The coagulation of left/right NTS suppressed but not ceased the photo-evoked sucking rhythm. The coagulation of NTS on both sides ceased the rhythm completely. These results indicate that the neurons in NTS are essential to evoke the sucking rhythm, but not neurons in AP. (COI:No)

### PP-329

### The respiratory generation requires endogenous hydrogen sulfide in anesthetized rats and *in situ* arterially perfused preparations of rats.

Minako Okazaki[1,2], Tadachika Koganezawa[1,3] (*[1]Dept Physiol, Fac Med, Univ Tsukuba, Tsukuba, Ibaraki, Japan, [2]Mstr Prog Med Sci, Grad Sch Comp Human Sci, Univ Tsukuba, Ibaraki, Japan, [3]Transborder Med Res Ctr, Univ Tsukuba, Ibaraki, Japan*)

Hydrogen sulfide ($H_2S$) is generally known as a toxic gas. On the other hand, endogenous $H_2S$ also has physiological roles in the central nervous system. However, its central roles for respiration, which is generated by neural circuit at the respiratory center, are still unclear. In this study, we aimed to clarify the roles of endogenous $H_2S$ in generating respiration. We used anesthetized rats and recorded the diaphragmic electromyogram (EMG) to observe the respiratory outputs. After the $H_2S$ synthase inhibitor was intravenously administered, the diaphragmic EMG temporarily became silent, and showed the gasping-like respiration with decremental shape before the respiration was completely stopped. We also performed *in situ* arterially perfused preparations of decerebrated rats and recorded the phrenic nerve activity. With inhibiting $H_2S$ synthesis, the central respiratory outputs switched from eupnea into gasping-like pattern as observed in anesthetized rats. These results suggest that endogenously produced $H_2S$ has essential roles to generate eupnea and the eupneic pattern changes into gasping when $H_2S$ level is decreased. (COI:No)

### PP-330

### Establishment of human pulmonary fibrosis model with iPS cells-derived organoids.

Tomomi Tadokoro[1], Mimoko Kato[1], Tatsuya Kobayashi[1], Takanori Takebe[2,4], Hideki Taniguchi[1,3] (*[1]Dept of Regen Med, Grad Sch of Med, Yokohama City Univ, [2]Inst of Res, Div of Adv Res, TMDU, [3]Div of Regen Med, Ctr for stem cell biol and Regen Med, The Inst of Med Sci, The Univ of Tokyo, [4]Dept of Pediatrics, Univ of Cincinnati Coll of Med*)

Pulmonary fibrosis is a chronic and progressive disease, which gradually thickens, stiffs, and scars alveoli and leads to the difficulty of breathing. One of the main causes for pulmonary fibrosis is the proliferation and activation of lung fibroblasts. In this study, we established the in vitro pulmonary fibrosis model from human induced pluripotent stem cells (hiPSC). First, hiPSCs were differentiated into the FOXA2 and SOX2 positive anterior foregut endoderm cells containing mesenchymal cells, and then differentiated into SOX2 and SOX9 positive lung bud tip cells. After that, lung progenitor cells which express NKX2-1 were induced and cultured as 3D organoids in Matrigel. After 2 weeks of culture, expression of AQP5 (alveolar type I marker), SFTPC and LPCAT1 (alveolar type II markers) was confirmed. Next, pulmonary organoids were treated with fibrotic stimulus such as TGF-b1 to induce fibrosis by activating mesenchymal cells. As a result, increase of SMA positive cells was observed after fibrotic stimulus. Taken together, in vitro pulmonary fibrosis model was developed. This model will be used for screening to find effective drugs of pulmonary fibrosis in the near future. (COI:No)

### PP-331

### Expression of leukocyte adhesion molecules, and FGF23 and ACE2 in *P. gingivalis* LPS-induced diabetic nephropathy

Koichiro Kajiwara[1], Yoshihiko Sawa[2], Sachio Tamaoki[1] (*[1]Section of Orthodontics, Department of Oral Growth & Development, Division of Clinical Dentistry, Fukuoka Dental College, [2]Deparment of Oral Function & Anatomy Okayama University Graduate School of Medicine, Dentistry and Pharmaceutical Sciences*)

This study aims to examine the expression of leukocyte adhesion molecules and renal metabolic factors in diabetic mouse kidneys with *Porphyromonas* (*P.*) *gingivalis* LPS-induced nephropathy. The immunohistochemical test was performed on diabetic mouse kidney with Pg-LPS-induced nephropathy. There were no vessels which expressed VCAM-1, E-selectin, or FGF23 in streptozotocin (STZ)-induced diabetic ICR mice (STZ-ICR), or in healthy ICR mice administered Pg-LPS (LPS-ICR). In diabetic ICR mouse kidneys with Pg-LPS-induced nephropathy (LPS-STZ) the expression of VCAM-1 and the accumulation of FGF23 were observed in renal tubules and glomeruli, and the expression of E-selectin was observed in renal parenchyma and glomeruli. The ACE2 was detected in the proximal tubules but not in other regions of ICR, STZ-ICR, or LPS-ICR. In LPS-STZ ACE2 was detected both in renal tubules as well as in glomeruli. The Pg-LPS may induce diabetic renal inflammation via glomerular overexpression of VCAM-1 and E-selectin, resulting in accumulation of both ACE2 and FGF23 which were unmetabolized with the inflammation-induced kidney damage under the diabetic condition. (COI:No)

### PP-332

### Proteomic analysis of urine samples in rat active Heymman nephritis models using LCMS/MS method

Dedong Kang[1], Toshihiro Aiuchi [2], Takashi Takaki [1,3], Mariko Gunji [1], Chika Sawa [1], Hiroyuki Itabe [2], Kazuho Honda[1] (*[1]Department of Anatomy, Showa University School of Medicine,, [2]Department of Pharmaceutical Sciences, Division of Biological Chemistry, Showa University School of Pharmacy, [3]Division of Electron Microscopy, Showa University*)

**Background:** LCMS/MS serves as a useful diagnostic tool in renal diseases. Here we present the results of LCMS/MS performed on urine samples of rat AHN models as an animal model of human MN. This study aims to establish a method for analyzing proteomics of urine components and explore the biomarker proteins of MN and proximal tubular injury.

**Method:** Clean-catch urine samples were obtained from rats of control (n=3) and AHN (n=6) groups. All samples were centrifuged to obtain supernatants and sediments. Precipitated supernatant and sediment proteins were digested by trypsin into peptides for LCMS/MS analysis.

**Results:** 203 proteins in urine supernatants and 553 proteins in urinesediments were identified by comparison to the Swissport database.The most significantly altered proteins Included immunoglobulin, complement components, vitamin and lipid metabolites, coagulation factors and constitutional proteins derived from renal tubular damage.

**Conclusion:** The proteome of the urine samples revealed by LCMS/MS is a useful diagnostic tool for evaluating glomerular and renal tubular injuries of rat AHN models and may help to explore the biomarker proteins of human kidney diseases (COI:No)

### PP-333

### Effects of angiotensin type 1 receptor blocker losartan on prostatic enlargement in spontaneously hypertensive rats

Shogo Shimizu[1], Yoshiki Nagao[2], Tamaki Takaoka[1,3], Shiho Kamada[1,3], Takahiro Shimizu[1], Yoichiro Higashi[1], Motoaki Saito[1] (*[1]Department of Pharmacology, Kochi Medical School, Kochi University, [2]Department of Pediatrics, Kochi Medical School, Kochi University, [3]Center for Innovative and Translational Medicine, Kochi Medical School, Kochi University*)

Purpose: We examined the effects of losartan, an angiotensin II type 1 receptor blocker, on prostatic enlargement in SHRs.

Methods: Male thirty six weeks old SHRs were perorally treated with losartan (3 or 10 mg/kg) or vehicle once daily for 18 weeks. Vehicle-treated normotensive Wistar Kyoto rats (WKYs) were used as controls. After the treatments, prostate weight, blood pressure and prostatic blood flow (PBF) were measured. The tissue levels of oxidative stress marker (MDA), pro inflammatory cytokine (IL-6), and growth factor (bFGF) levels were measured. Histological analysis was evaluated by HE staining.

Results: The vehicle-treated SHRs demonstrated significant increases in blood pressure, prostate weight/body weight ratio (PBR), glandular epithelial area, and tissue MDA, IL-6, and bFGF levels in the ventral prostate and a decrease in PBF compared to the vehicle-treated WKYs. Treatment with losartan dose dependently ameliorated PBF, and decreased PBR, blood pressure and glandular epithelial area as well as tissue MDA, IL-6, and bFGF levels in the SHR ventral prostate.

Conclusions: Chronic treatment with losartan could ameliorate prostatic enlargement in SHRs. (COI:No)

### PP-334

### Kidney-on-a-chip model using human renal proximal tubular epithelial cells and human umbilical vein endothelial cells

YIN LIANG [1], Mengxue Wang[1], Keiji Naruse[1], Ken Takahashi[1] ([1]*Dept Cardiovasc Physiol, Grad Sch Med Dent Pharm Sci, Okayama Univ, Okayama, Japan*)

The kidney is a vital organ that controls the reabsorption and excretion of substances. To develop a disease model of the human kidney and to evaluate drug efficacy, researchers need to use an experimental system using human cells instead of animal cells. Hence, to mimic the nephron, we used human renal proximal tubular epithelial cells (RPTECs) and human umbilical vein endothelial cells (HUVECs). RPTECs and HUVECs were seeded on the top side and the other side, respectively, of a 50 µm-thick membrane with 7 µm pores hexagonally packed at 40 µm interval. Blood and urine flows were stimulated using microfluidics. The activities of alkaline phosphatase and CD13, which are RPTEC markers, were confirmed in the cells on the top channel by live imaging and immunocytochemistry, respectively. In addition to these histological analyses, we will conduct functional analysis such as glucose transport assay and proceed with the development of human kidney-on-a-chip model. (COI:No)

### PP-335

### The transcription factor HAND2 up-regulates transcription of the *IL15* gene in human endometrial stromal cells

Susumu Tanaka[1], Hiromi Murata[2], Hidetaka Okada[2], Masaaki Kitada[1] ([1]*Dep. Anat., Kansai Med Univ.,* [2]*Dep. Obstet. Gynecol., Kansai Med Univ*)

Proliferation, secretion, and decidualization occur during regular menstrual cycles in the human endometrium. HAND2 is a key transcription factor in decidualization of human endometrial stromal cells (ESCs). Alternatively, IL15, a key immune factor required for the activation and survival of uterine natural killer (uNK) cells and activated uNK cells promote spiral artery remodeling and induce immunotolerance. To date, no studies have evaluated the transcription factors for IL15 expression in human ESCs. In the present study, we examined whether HAND2 controls IL15 transcription in human ESCs. qRT-PCR and histological analyses revealed HAND2 and IL15 levels increase considerably in the secretory phase. ChIP-qPCR showed HAND2 binds to a putative HAND2 motif. Using a luciferase reporter assay, human IL15 upstream region up-regulates luciferase activities in response to estradiol and a progesterone in ESCs. The human IL15 upstream region also exhibited increasing responsiveness to HAND2. Of note, deletion and substitution variants of the motif did not respond to HAND2. These findings confirm HAND2 directly up-regulates human IL15 transcription in ESCs. (COI:No)

### PP-336

### Cytonuclear estrogen receptor alpha regulates proliferation and migration of endometrial carcinoma cells

Zhong-Lian Li[1], Saimi Mierxiati[1], Shota Moriya[2], Hidenobu Miyaso[1], Kenta Nagahori[1], Shinichi Kawata [1], Takuya Omotehara[1], Yuki Ogawa[1], Hirotsugu Hino[3], Keisuke Miyazawa[2], Masahiro Itoh[1] ([1]*Dept. of Anat., Tokyo Med. Univ.,* [2]*Dept. of Biochem., Tokyo Med. Univ.,* [3]*Dept. of Func. Morph., Div. of Anat. Sci., Nihon Univ*)

**Objective:** The effects of estrogen on cells are mediated by the estrogen receptor α (ERα) which localizes at the peri-membrane, cytoplasm, and the nucleus of cells. Therefore, we intended to investigate how cytonuclear ERα plays its roles in different cellular activities.
**Methods:** ERα-negative endometrial carcinoma cells (ERα-) were stably transfected with plasmid of human ERα carrying a substituted phenylalanine at position 445 with alanine (ERα-F445A).
**Results:** E2 significantly activated proliferation and migration in ERα-F445A cells. While no obvious change in the amount of the non-phosphorylated mammalian target of Rapamycin (mTOR), the expression of mTOR phosphorylated at serine 2448 decreased, which was recovered in presence of 17β-estrogen (E2) in the ERα-F445A cells. On the other hand, the expression of focal adhesion kinase (FAK) phosphorylated at tyrosine at 297 was attenuated in the ERα-F445A cells treated with E2.
**Conclusion:** It is suggested that the cytonuclear ERα-F445A induces phosphorylation of kinases in downstream pathways, which regulate cell proliferation and migration. (COI:No)

### PP-337

### Expression of ephrin-B1 and EphB4 in steroidogenic cells in mouse ovaries.

Jahagir Alam[1], Kazushige Ogawa[1] ([1]*Vet. Anat., Grad. Sch. Life Environ. Sci., Osaka Prefect. Univ*)

The expression and function of Eph receptors and ephrin ligands in the ovary are virtually unknown. We previously found ephrin-B1 and EphB4 expression in fetal and adult Leydig cells and therefore have examined expression and localization of ephrin-B1 and EphB4 in the ICR mouse ovaries, especially focused on their expression in steroidogenic cells. By immunofluorescence microscopy, we found that ephrin-B1 and EphB4 immunoreactivity were localized in granulosa cells, theca cells, luteal cells, and interstitial gland cells. Interestingly, we found that ephrin-B1 immunoreactivity in luteal cells was weak likely in developing CL and temporally mature CL, and strong in regressing CL. These results at least suggested that ephrin-B1 and EphB4 are commonly expressed in sex steroid producing cells, and therefore are likely good maker molecules for these cells. (COI:No)

### PP-338

### Prosaposin in the rat oviductal epithelial cells

Tetsuya Shimokawa[1], Hiroaki Nabeka[1], Khan Md. Sakirul Islam[1], Takuya Doihara[1], Hiroyuki Wakisaka[2], Naoto Kobayashi[3], Seiji Matsuda[1] ([1]*Department of Anatomy and Embryology, Ehime University Graduate School of Medicine,* [2]*Department of Liberal Arts, Ehime Prefectural University of Health Sciences,* [3]*Medical Education Center, Ehime University Graduate School of Medicine*)

Prosaposin (PSAP) has two forms: a precursor and a secreted form. Immunoblots of oviducts showed that oviductal tissues contain PSAP proteins, and a significant increase in PSAP was observed in the estrus-metestrus phase compared to the diestrus-proestrus phase in the ampulla. To identify PSAP trafficking in cells, double-immunostaining was performed with antibodies against PSAP in combination with sortilin, mannose 6 phosphate receptor (M6PR), or low-density lipoprotein receptor-related protein 1 (LRP1). PSAP and sortilin double-positive reactions were observed near the nuclei of both epithelial cells. PSAP and M6PR double-positive reactions were also observed near the nuclei of both epithelial cells. PSAP and LRP1 double-positive reactions were observed in the plasma membrane and apical portion of both epithelial cells. Immunoelectron staining revealed PSAP immunoreactive small vesicles with exocytotic features at the apical portion of microvillous epithelial cells. These findings suggest that PSAP is present in the oviductal epithelium and has a pivotal role during pregnancy in providing an optimal environment for gametes and/or sperm in the ampulla. (COI:No)

### PP-339

### Ephrin-B1, EphB2, and EphB4 expression in the mouse testis during postnatal development

Kazushige Ogawa[1], Md. Royhan Gofur[2], Jahagir Alam[1] ([1]*Dept. Vet. Anat., Grad. Sch. Life Environ. Sci., Osaka Prefect. Univ.,* [2]*Dept. Vet. Anim. Sci., Fac. Agri., Univ. Rajshahi*)

Ephrin-B and EphB have been implicated in boundary formation in various epithelia. We previously found that ephrin-B1 and EphB2/EphB4 exhibit complementary expression in the epithelia along the excurrent duct system in the adult mouse testis. Here, we examined the expression of ephrin-B1, EphB2 and EphB4 in the mouse testis during postnatal development. RT-PCR analysis revealed that the relative expression levels of these molecules decreased with age in early postnatal development, and showed values close to the adult levels by 4 weeks of age. By immunostaining, we found that compartments with complementary expression of ephrin-B1 and EphB2/EphB4 in the excurrent duct system observed in the adults were formed by 2 weeks of age. This study is the first to investigate the expression of ephrin-B1, EphB2, and EphB4 in the normal mouse testis during postnatal development. The expression patterns of the ephrin-B and EphBs may represent suitable tools for examining organization of the excurrent duct system during postnatal development. (COI:No)

### PP-340

### Histochemical analysis of DROSHA in a trophoblast cell line BeWo with syncytial fusion

Syunya Noguchi[1], Toshihiro Takizawa[1] ([1]*Department of Molecular Medicine and Anatomy, Nippon Medical School*)

**[Objective]** DROSHA, a nuclear RNase III enzyme, cleaves pri-miRNA into pre-miRNA in miRNA biosynthesis. However, there is little information available on DROSHA dynamics in the syncytial fusion of trophoblast cells. In this study, we investigated the expression of DROSHA in a trophoblast cell line BeWo by immunohistochemistry.
**[Methods]** Syncytial fusion of BeWo cells was induced by stimulation of forskolin; fusion efficiency was evaluated by the fusion index. BeWo cells were treated with forskolin for 72 h, immunostained with anti-DROSHA antibody and then visualized with Alexa Fluor conjugated secondary antibody.
**[Results]** Immunohistochemical analysis showed that prior to fusion, DROSHA fluorescence signals in BeWo cells was both diffuse and dot-like in the nucleoplasm. After syncytial fusion, the expression of DROSHA was increased in some nuclei and decreased in others in the same syncytialized cells.
**[Conclusion]** Uneven nuclear expression of DROSHA in multinucleated BeWo cells suggests alteration of miRNA biosynthesis in each nucleus; however, further studies are needed to understand the dynamics of DROSHA in the syncytial fusion of trophoblast cells.

(COI:No)

### PP-341

### Cellular and subcellular localization of mouse placenta-associated lncRNA *1600012P17Rik*

Junxiao Wang[1], Syunya Noguchi[1], Takami Takizawa[1], Aya Misawa[1], Shan-Shun Luo[2], Toshihiro Takizawa[1] ([1]*Department of Molecular Medicine and Anatomy, Nippon Medical School, Tokyo, Japan,* [2]*Department of Geriatrics, the First Hospital of Harbin Medical University, Harbin, China*)

[**Objective**] We have previously reported that long noncoding RNA (lncRNA) *1600012P17Rik* (designated as P17Rik) was expressed in the mouse placenta during late gestation. In this study, we further investigated the cellular and subcellular localization of P17Rik in the mouse placenta.
[**Methods**] The placenta samples (E16.5) of B6D2F1 mice was fixed with paraformaldehyde and embedded in paraffin. In situ hybridization analysis was performed using a branched DNA signal amplification method; P17Rik signals were visualized with alkaline phosphatase with Fast Red. Fast Red signals was captured in both bright-filed and fluorescence modes.
[**Results**] P17Rik was positive in spongiotrophoblast and glycogen trophoblast cells, but not in trophoblast giant cells in the junctional zone of the mouse placenta. A combination of fluorescent Fast Red signals and nuclear DAPI staining revealed that the intracellular localization of P17Rik was mainly in the cytoplasm of these trophoblast cells.
[**Conclusion**] Our data suggest that a functional site on P17Rik may be the cytoplasm in these cells. Fast Red is valuable for identification of the cellular and subcellular localization of molecules of interest.

(COI:No)

### PP-342

### Autophagy induction on impaired spermatogenesis of xeroderma pigmentosum group A gene-deficient mice

Hironobu Nakane[1], Katsumi Higaki[2], Yuka Koyama[1], Eiji Nanba[3], Toshiyuki Kaidoh[1] ([1]*Department of Anatomy, Faculty of Medicine, Tottori University,* [2]*Research InitiativeCenter, Research Strategy Division, Organization for Research Initiative andPromotion, Tottori University,* [3]*Research Strategy Division, Organization for Research Initiative andPromotion, Tottori University*)

Xeroderma pigmentosum (XP) has a defect in the initial step of nucleotide excision repair (NER) and consists of eight genetic complementation groups (groups A-G and a variant). XP group A (XPA) patients have a high incidence of UV-induced skin tumors, immature testicular development, and neurological symptoms. We previously indicated that XP group A (*Xpa*) gene-knockout mice [$Xpa^{-/-}$mice] were highly sensitive to UV-induced skin carcinogenesis with a defect in NER and were highly susceptibility to spontaneous tumorigenesis with impaired spermatogenesis. However, the pathology of impaired spermatogenesis in $Xpa^{-/-}$mice is unknown. To elucidate the pathology, we examined the testis of 3-month-old $Xpa^{-/-}$mice.We found a lot of large vacuoles in the testis of $Xpa^{-/-}$mice, while there were no large vacuoles in that of $Xpa^{+/+}$mice. Immunohistochemistry of microtubule-associated protein 1 light chain 3(LC3), an autophagosome marker, showed degenerating cells with intense signal of LC3 in the testis, and western blotting revealed induction of LC3-II in the $Xpa^{-/-}$mice. These results suggest autophagy induction as the possible mechanism underlying the impaired spermatogenesis in $Xpa^{-/-}$ mice.

(COI:No)

### PP-343

### Odf2 haploinsufficiency causes a new type of decapitated and decaudated spermatozoa, Odf2-DDS, in mice

Chizuru Ito[1], Hidenori Akutsu[2], Ryoji Yao[3], Keiichi Yoshida[1,4], Kenji Yamatoya[1,5], Tohru Mutoh[1], Tsukasa Makino[6], Kazuhiro Aoyama[7], Hiroaki Ishikawa[8,9], Koushi Kunimoto[8,10], Sachiko Tsukita[8], Tetsuo Noda[3], Masahide Kikkawa[6], Kiyotaka Toshimori[1] ([1]*Department of Functional Anatomy, Reproductive Biology and Medicine, Graduate School of Medicine, Chiba University,* [2]*Department of Reproductive Biology and Pathology, National Research Institute for Child Health and Development,* [3]*Department of Cell Biology, Japanese Foundation for Cancer Research [JFCR] Cancer Institute,* [4]*Osaka International Cancer Institute, Next-generation Development Center for Cancer Treatment,* [5]*Institute for Environment & Gender-specific Medicine, Juntendo University Graduate School of Medicine,* [6]*Department of Cell Biology and Anatomy, Graduate School of Medicine, The University of Tokyo,* [7]*Materials and Structural Analysis [ex FEI], Thermo Fischer Scientific,* [8]*Graduate School of Frontier Biosciences and Medicine, Osaka University,* [9]*Department of Biochemistry and Biophysics, University of California,* [10]*Department of Pathology, Stanford University School of Medicine*)

Outer dense fiber 2 (Odf2) is a cytoskeletal protein required for tail-beating and stability to transport sperm cells from testes to the eggs. There are infertile males who have a high percentage of decapitated and decaudated spermatozoa (DDS), whose semen contains abnormal spermatozoa with tailless heads and headless tails due to head-neck separation. DDS is untreatable in reproductive medicine. We report a new type of Odf2-DDS in heterozygous mutant $Odf2^{+/-}$ mice. $Odf2^{+/-}$ males were infertile due to sperm neck-midpiece separation (headneck sperm cells and neckless sperm tails) caused by Odf2 haploinsufficiency. The headnecks were immotile but alive and capable of producing offspring by intracytoplasmic headneck sperm injection (ICSI). The neckless tails were motile and could induce capacitation but had no significant forward motility. It is a possible that there are human Odf2-DDS patients, and that ICSI will be effective treatment for those patients.

(COI:No)

### PP-344

### Analysis of the gene expression in the fetal gonads of Sertoli cell-specific conditional SF-1 knockout mice

Mamiko Maekawa[1], Ayako Tagami[1], Akiko Nagai[1], Yayoi Ikeda[1] ([1]*Dept Anat, Sch Dent, Aichi-Gakuin Univ*)

The nuclear receptor steroidogenic factor 1 (SF-1) is essential for gonadal development. To study the importance of SF-1 during early gonadal differentiation, we generated conditional knockout (cKO) mice, in which SF-1 was specifically inactivated in Sertoli cells using Cre-loxP recombination with Sox9-Cre. Two different cKO genotypes, *Sox9:Cre;* $Sf\text{-}1^{flox/-}$ (cKO$Sf\text{-}1^{f/-}$) and *Sox9:Cre;* $Sf\text{-}1^{flox/flox}$(cKO$Sf\text{-}1^{f/f}$) mice were obtained, and the XY cKO mice exhibited different phenotypes in the reproductive organs. XY cKO$Sf\text{-}1^{f/-}$mice had very small ovaries, while the gonads of XY cKO$Sf\text{-}1^{f/f}$were small ovotestes. To compare the expression levels of genes involved in gonadal development, qRT-PCR was performed in XY cKO mice gonads. The expression levels of *Sf-1*,*Sox9*, *Amh*,*Dhh*, *Cyp11a1*(testis markers), *Foxl2*,*Fst*(ovary markers), and *Ddx4*(a germ cell marker) were analysed in E12.5 and E13.5 gonads. The results revealed a reduction and an elevation in the levels of testis and ovary markers, respectively, in the gonads of XY cKO mice. The degrees of the reduction and elevation were greater in cKO$Sf\text{-}1^{f/-}$mice than in cKO$Sf\text{-}1^{f/f}$mice, showing good agreement with their phenotypes.

(COI:No)

### PP-345

### Expression and localization of AQP11 mRNA in the mouse testis

Maiko Ikezawa[1], Hiroshi Kogo[1], Akiko Kogo[1], Keinichi Ishibashi[2], Toshiyuki Matsuzaki[2] ([1]*Department of Anatomy and Cell Biology, Gunma University Graduate School of Medicine,* [2]*Division of Pathophysiology, Meiji Pharmaceutical University*)

Aquaporin 11 (AQP11) is a membrane water channel, and its mRNA is highly expressed in the mouse testis. The physiological function of AQP11 has not been elucidated in the testis because most AQP11 knockout mice died within one month after birth, that is before sperm production, due to renal failure with polycystic kidneys. Conditional knockout mice are required to investigate the testicular function of AQP11. However, it is not well determined what cells express AQP11. In this study, we used in situ hybridization to examine AQP11 mRNA expression and localization in the testis by RNAscope technology using paraffin sections of testes from 6 week-old wild-type and rarely survived knockout mice. AQP11 mRNA was detected in late spermatocytes after stage VIII and spermatids of all stages, but not in earlier spermatocytes, spermatogonia and Sertoli cells. Therefore, to generate testis-specific AQP11 deleted mice, Cre should be driven by a promoter which is active in earlier spermatogenic cells. We are currently re-evaluating the immunolocalization of the AQP11 protein, and trying to generate the conditional knockout mice.

(COI:No)

### PP-346

### Localized HORMAD1 Ser378 phosphorylation appears to be associated with, but independent of pseudo sex bodies in SPO11-deficient spermatocytes

Hiroshi Kogo[1], Akiko Kogo[1], Maiko Ikezawa[1], Toshiyuki Matsuzaki[1]
([1]*Department of Anatomy and Cell Biology, Gunma University Graduate School of Medicine*)

HORMAD1 is a meiosis-specific phosphoprotein, having multiple function including sex body formation and asynapsis surveillance during meiotic prophase I. Previous results showed that the Ser378 phosphorylation (pSer378) was associated, but not always, with pseudo sex body in *Spo11*$^{-/-}$ spermatocytes, leaving the association unclear. Here, we carefully examined the pSer378 distribution with detailed substaging of *Spo11*$^{-/-}$ spermatocytes based on axes morphology. We found that pSer378-positive pseudo sex bodies gradually increased in early-zygotene (17%), mid-zygotene (70%) and pachytene-like (96%) substages, showing their close association in later substages. Interestingly, some pSer378 signals were observed outside pseudo sex bodies in early-zygotene (74%), mid-zygotene (23%) and pachytene-like (7%) substages, indicating that the Ser378 phosphorylation and pseudo sex body are formed independently, and merge afterwards. Although the functional significance of pSer378 remains unknown, this result predicts the as yet unidentified kinase-activated domain in extensively asynaptic spermatocytes, which might be relevant with the asynapsis surveillance signaling mechanism. (COI:No)

### PP-347

### Repetitive Ca$^{2+}$ increases coordinate the reorganization of cortical actin cytoskeleton and meiotic resumption in mammalian eggs

Hideki Shirakawa[1], Kento Kondo[1] ([1]*Department of Engineering Science, The University of Electro-Communications*)

Cortical actin filaments (F-actin) lining the plasma membrane of mammalian oocytes are involved in various events during oocyte maturation and egg activation at fertilization, including polarity formation, chromosome localization, polar body emission, and polyspermy block. We investigated how the cortical F-actin organization in mouse eggs is regulated by repetitive rises in cytosolic Ca$^{2+}$, or Ca$^{2+}$ oscillations, which are induced by sperm-borne egg-activating protein PLC$\zeta$. A thick layer of F-actin (actin cap; AC) near the chromosomes located in the animal pole rapidly degraded after a few Ca$^{2+}$ rises, and was reorganized into the contractile ring to emit the polar body. The timing of AC degradation coincided reasonably with the chromosome separation. Interestingly, the inhibition of calpain, a Ca$^{2+}$-dependent protease, delayed not only the onset of AC degradation, but also that of chromosome separation, suggesting that the reorganization of F-actin is regulated coordinately with the metaphase-to-anaphase transition in meiosis. In the presentation, the results that indicated the alternative Ca$^{2+}$-dependent regulatory mechanism of the cortical microvillar F-actin will be discussed. (COI:No)

### PP-348

### Selection of mouse fertilized ova by membrane potential measurement after freeze-thaw cycle

Masao Miyake[1], Susumu Yoshie[1], Satoru Kaneko[2], Akihiro Hazama[1]
([1]*Department of Cellular and Integrative Physiology, Fukushima Medical University, Fukushima, Japan,* [2]*Ichikawa General Hospital, Tokyo Dental Coll, Ichikawa, Japan*)

Morphological inspection is the most commonly used technique to pick quality oocytes and embryos for artificial fertilization. To raise reproductive ratio, a new selection method from a new point of view is needed. The membrane potential reflects expression of ion channels and completeness of cell membrane, it may evaluate ovum quality. We previously showed that there was a wide dispersion of membrane potential among eggs without morphological difference. It implied this technique could be applied for quality selection. In this study, we analyzed the relationships between embryogenic outcome and membrane potential of mouse embryos after freeze-thaw cycle. Two-cell embryos were applied to the freeze-thaw cycle, and measured membrane potential. Some embryos performed good morphological characteristics, and could reach blastocysts. But most embryos which performed near zero voltage stopped development. The near zero voltage embryos are possible to be scratched during conventional protocol. This method may be applicable to ignore damaged embryos. All animal experiments were planned toward institutional guidelines and reviewed by institutional animal care and use committee. (COI:No)

### PP-349

### Optimization of immunohistochemical detection of rat ESR2 proteins using anti-human ER$\beta$-specific antibody PPZ0506

Yujiro Hattori[1], Hirotaka Ishii[1], Shimpei Higo[1], Mai Otsuka[1], Moeko Kanaya[1], Keisuke Matsumoto[1], Mina Ozawa[1], Hitoshi Ozawa[1] ([1]*Dept. Anat. Neurobiol., Grad. Sch. Med., Nippon Med. Sch., Tokyo, Japan*)

Although absence of specific antibodies against ESR2 (which is also known as estrogen receptor $\beta$: ER$\beta$) has caused serious hindrance to promotion of ESR2 research, a specific anti-human ESR2 monoclonal antibody (PPZ0506) was identified in 2017. Our previous study confirmed its cross-reactivity and specificity against rodent ESR2 proteins, enabling the elucidation of true ESR2 distribution in rodents. Here, we attempted to determine for immunostainability of PPZ0506 and analyze distribution of ESR2 in rats. We evaluated several staining conditions using paraffin-embedded and frozen ovary sections. Immunohistochemical staining with PPZ0506 required appropriate antigen retrieval and antibody dilution. Subsequent immunohistochemical analysis in multiple tissues under optimized conditions revealed that rat ESR2 proteins are expressed in a more localized manner than previously assumed, implying that previous immunohistochemical studies using inadequately validated antibodies against ESR2 proteins overestimated their distribution profiles. We expect that optimized immunohistochemistry using PPZ0506 antibody can help researchers resolve challenges in ESR2 research. (COI:No)

### PP-350

### Expression of hypothalamic kisspeptin neurons in diabetic female rats

Hiroyuki Enomoto[1], Kinuyo Iwata[1], Hitoshi Ozawa[1] ([1]*Dept. Anat. Neurobiol., Grad. Sch. Med., Nippon Med. Sch., Tokyo, Japan*)

Hypothalamic kisspeptin neurons regulate luteinizing hormone (LH) release through GnRH neurons. Diabetic women often show menstrual disorder and infertility, but it is not clear whether kisspeptin neurons are associated with reproductive dysfunction in them. In this study, we investigated the expression of hypothalamic kisspeptin neurons in streptozotocin (STZ)-induced diabetic female rats. These rats were perfused with 4% paraformaldehyde 8 weeks after STZ injection. Kisspeptin neurons in the arcuate nucleus (ARC) coexpress tachykinin 3 and dynorphin A encoded by *Tac3* and *Pdyn*, respectively; they are involved in pulsatile LH secretion. Each mRNA-expressing cell was detected by ***in situ*** hybridization. The numbers of *Kiss1*, encodes kisspeptin, *Tac3* and *Pdyn* cells in the ARC of diabetic rats were significantly decreased compared with non-diabetic rats. Alternatively, the number of *Kiss1* cells in the anterior ventral periventricular nucleus (AVPV), which induce LH surge, did not differ significantly between the two groups. These results suggest that diabetes mellitus in females may negatively affect ARC kisspeptin neurons, resulting in menstrual disorder and infertility. (COI:No)

### PP-351

### Distributions and morphologies of serotonin (5-HT) containing enteroendocrine cells in the mouse large intestine

Hirofumi Kuramoto[1], Ryoichi Yoshimura[1], Furness John[2] ([1]*Cell Function, Applied Biology, Kyoto Inst. Tech.,* [2]*Anatomy & Neuroscience, Melbourne Univ*)

5-HT containing enteroendocrine cells in the gut contribute to the regulation of functions, including motility and secretion. This study identified morphologies and localization of subtypes of 5-HT cells in the large intestine. 5-HT cells were most frequent in the proximal colon compared with the cecum and distal colon. The large intestine had both open (O) cells with apical processes reaching the lumen, and closed (C) cells, not contacting the lumen. In the colon, 5-HT cells with long basal processes occurred. The open cells were subdivided into O1, O2 and O3 and the closed cells into C1, C2 and, C3 based on the lengths of their processes. The long processes of O1 and C1 cells ran close to the inner surfaces of the mucosal epithelial cells. O2 and C2 cells had similar but shorter processes and O3 and C3 cells had no or very short processes. Numerous O3 type 5-HT cells in the upper region of the crypts in the proximal colon may be involved in intraluminal 5-HT signaling. Predominant O3 type 5-HT cells in the lower region of the crypts in all segments of the large intestine may be related with cell differentiation, proliferation or renewal in the deeper area of the crypts. (COI:No)

### PP-352

### Expression and localization of the granin protein family in the hypothalamo-neurohypophyseal system

Ryosuke Morinaga[1], Tsuyoshi Watanabe[1] ([1]*Department of Microscopic Anatomy and Cell Biology, Asahikawa Medical University, Asahikawa, Japan*)

The granin protein family in secretory granules (SGs) of endocrine cells accelerates aggregation of hormones. Although neurons located in the paraventricular (PaV) and supraoptic (SO) nuclei in the hypothalamus (HPT) also release peptide hormones, vasopressin (VP) and oxytocin (OXT), at the posterior pituitary (pp), it is unclear that the granins are also involved in the SGs formation within VP and OXT neurons. Thus, we immunohistochemically examined the expression and localization of the granins including chromogranin A (CgA), chromogranin B (CgB), secretogranin II (Sg2), and secretogranin III (Sg3), in the rat HPT and pp. Sg2 and Sg3 were localized in the nerve terminals of pp, whereas neither CgA- nor CgB-immunoreactivities were observed in the pp. In the PaV and SO, Sg3 was localized in the soma of both VP and OXT neurons, while Sg2-immunoreactivity was observed not in the soma but at the button-shaped nerve terminals around these neurons in HPT. These results confirmed the expression of the granins in the VP and OXT neurons, and the distinct localization of Sg2 and Sg3 suggested the difference in the physiological roles of these granins in VP and OXT neurons. (COI:No)

### PP-353

### Depletion of secretogranin III in mice causes functional maladaptation of pancreatic beta cells to an inadequate high-fat/ high-sucrose diet.

Tsuyoshi Watanabe[1], Daisuke Koga[1], Ryosuke Morinaga[1], Masahiro Hosaka[2] ([1]*Dept. Microsc. Anat. Cell Biol., Asahikawa Med. Univ., Asahikawa, Japan,* [2]*Dept. Biotech., Akita Pref. Univ., Akita, Japan*)

Secretogranin III (Sg3), a member of the granin family, binds both to another granin, chromogranin A (CgA), and to a cholesterol-rich membrane that is destined for secretory granules (SGs). Although the knockdown of Sg3 in ACTH-producing AtT-20 cells largely impairs formation of SGs containing CgA and ACTH, the functional significance of Sg3 in vivo is still unclear. To clarify the physiological roles of Sg3 in vivo, we analyzed hormone secretion and SG biogenesis in newly established Sg3 knockout (KO) mice. Although the Sg3-KO mice exhibited no overt abnormalities under ordinary rearing conditions, a high-fat/high-sucrose diet caused pronounced obesity in the mice. The stimulated secretion of active insulin significantly decreased in the Sg3-KO mice compared with wild-type mice, whereas storage of proinsulin increased in the islets. These findings suggest that the lack of Sg3 causes maladaptation of pancreatic beta cells to an inadequate diet by impairing the proteolytic conversion of prohormones in the SGs, whereas SG biogenesis and the basal secretion of peptide hormones under ordinary conditions are ensured by the compensative upregulation of other residual granins. (COI:No)

### PP-354

### Identification of melanin concentrating hormone receptors expressing in the adenohypophysis of a basal actinopterygian fish, *Polypterus senegalus*

Morio Azuma[1], Taka-aki Koshimizu[1] ([1]*Div. of Mol. Pharmacol., Dept. of Pharmacol., Jichi Med. Univ*)

Melanin concentrating hormone (MCH) is a peptide conserved from fish to mammal, and well known as an orexigenic neuropeptide in mammals. Recently, we showed MCH-immunopositive fibers in the median eminence of a basal actinopterygian fish, *Polypterus senegalus*, that is located in the phylogenetic branch of fishes and tetrapod. However, the functions of MCH on the endocrine cells in the adenohypophysis are unknown. To investigate that, in this study, we examined the expression of MCH receptors in the gland of this fish. We cloned MCH receptor cDNAs from *P. senegalus* and characterized phylogenetically. According to phylogenetic tree of MCH receptors, *P. senegalus* has two-MCH receptor 1 and two-MCH receptor 2 that are close to the receptors of mammal or the receptors of teleost. To investigate the expression of MCH receptors in the adenohypophysis, we performed *in situ* hybridization. We found that mammal-like MCH receptor 2 and teleost-like MCH receptor 2 express in the endocrine cells of the gland. These findings suggest that two- MCH receptor 2 are contributed to the regulation of adenohypophyseal hormone in this fish. (COI:No)

### PP-355

### Maternal prolactin levels during late pregnancy and the influence on generating the nurturing behavior in the offspring

Taku Sairenji[1], Shinnosuke Masuda[1,2], Kwan Ee Oh[1], Seika Sato[1], Hiroyuki Yajima[1], Izuki Amano[1], Noriaki Shimokawa[1,3], Noriyuki Koibuchi[1] ([1]*Department of Integrative Physiology, Gunma University, Japan,* [2]*Epigenetics and Metabolism, IMCR, Gunma Univ, Japan,* [3]*Dept Nutr, Takasaki Univ Health Welf*)

Prolactin (PRL) secreted during late pregnancy is involved in initiating maternal behavior in rodents. We previously reported the possibility of this maternal PRL also to be is important in developing nurturing behavior in the offspring during the fetal stage. This transgenerational effect was discovered by using Cbl-interacting protein of 85 kDa (CIN85) deficient mice (KO), which show a low PRL secretion capability. In this study, we aimed to gain an accurate view of PRL secretion during late pregnancy and how the PRL concentration would affect the fetus, by using C57BL/6 wild type mice. First, we measured the plasma PRL concentration every 4 hours from gestational day 17(G17) to delivery. Second, we suppressed the PRL secretion during late pregnancy by bromocriptine (BC, dopamine agonist) injection. Then the nurturing behavior of the offspring were investigated when they matured. Mice born to the BC injected dams did not show any abnormality in maternal behavior compared to the control group. These results suggest that the development of maternal behavior cannot be disrupted by only suppressing PRL secretion during late pregnancy. (COI:No)

### PP-356

### Sucralose activates intracellular $Ca^{2+}$ and cAMP signaling in enteroendocrine L cells

Marie Mita[1], Mai Takizawa[1], Rei Nagata[1], Kazuki Harada[1], Hiroshi Ueda[2], Tetsuya Kitaguchi[2], Takashi Tsuboi[1] ([1]*Dept. Life Sci., Grad. Sch. Arts. Sci., Univ. Tokyo, Tokyo, Japan,* [2]*CLS, IIR, Tokyo Tech., Kanagawa, Japan*)

An artificial sweetener, sucralose is widely used for various food instead of sugar. Sucralose induces glucagon-like peptide-1 (GLP-1) secretion from enteroendocrine L cells, but the mechanisms of GLP-1 secretion by sucralose is unknown. Here, we visualized intracellular $Ca^{2+}$ and cAMP dynamics ($[Ca^{2+}]_i$ and $[cAMP]_i$) by live-cell imaging analysis on mouse enteroendocrine L cell line, GLUTag cells. We found that sucralose increased not only the $[Ca^{2+}]_i$ but also the $[cAMP]_i$. To explore the signaling pathway, we applied sucralose with the inhibitors of $G_q$ protein, adenylyl cyclase, or calcium-sensing receptor (CaSR), one of the class C G-protein coupled receptors. The results revealed that the $[Ca^{2+}]_i$ was increased via $G_q$ signaling. Surprisingly, it further suggested that CaSR signaling induces $[cAMP]_i$ elevation. Finally, by using the green fluorescent protein-based glucose indicator, Green Glifon, we revealed the elevation of intracellular glucose levels ($[glucose]_i$) was triggered by sucralose application. In conclusion, sucralose may induce the elevation of $[Ca^{2+}]_i$, $[cAMP]_i$, and $[glucose]_i$ and regulate the pathway of GLP-1 secretion in L cells. (COI:No)

### PP-357

### Effects of the activation of oxytocin neurons by employing Designer's Receptor Exclusively Activated by Designer's Drugs (DREADDs) on antinociceptive behavior

Mitsuhiro Yoshimura[1], Haruki Nishimura[2], Makoto Kawasaki[2], Satomi Sonoda[3], Akinori Sakai[2], Yoichi Ueta[1] ([1]*Dept. Physiol., Sch Med, UOEH, Kitakyushu, Japan,* [2]*Dept. Orthopaedic Surgery., Sch Med, UOEH, Kitakyushu, Japan,* [3]*Dept. Internal Med* [1]*., Sch Med, UOEH, Kitakyushu, Japan*)

Oxytocin, synthesized in the supraoptic (SON) and paraventricular nuclei (PVN), has diverse central effects as well as peripheral action. Antinociceptive effect is considered as one of the central actions of oxytocin. Here, we have generated a transgenic rat line that expresses hM3Dq and mCherry exclusively on oxytocin neurons. Perfusion and fixation were carried out 120 min after subcutaneous (s.c) injection of Clozapine-N-oxide (CNO, 1 mg/kg), which is an agonist of hM3Dq, or saline in these transgenic rats, then immunofluorescence was performed. Fos expression was dramatically increased in CNO treated rats compared to saline exclusively in oxytocin neurons in the SON and PVN that were tagged with mCherry fluorescence, which indicated that inserted *hM3Dq* was functioning. We evaluated the effect of oxytocin on antinociceptive behaviors after subcutaneously administered CNO by von Frey test (mechanical sensitivity) and hot plate test (heat sensitivity). Mechanical/heat sensitivity was significantly blunted 30 min and 60 min after s.c. injection of CNO, respectively. The present study could be a breakthrough as it would provide the direct evidence of oxytocin on antinociception. (COI:No)

### PP-358

### Circulating GnRH and FSH in castrated male rats attract sexual mature male rats

Himeka Hayashi[1], Yasuhiko Kondo[1] ([1] *Teikyo univ sci*)

Sexually mature male rats prefer not only estrous female but castrated male odor to gonadally intact male odor. We investigated why castrated males attract sexually active males. To measure the attractivity, we prepared, as probes, sexually experienced male rats that showed clear preference for estrous odor. Three groups of stimulus males were made: (1) castrated males (Cast), (2) castrated plus hypophysectomized males (HPx) and (3) gonadally intact males (Sham). The probe males spent significantly longer time to explore odor of Cast than that of Sham and HPx, indicating that the attractant depends on circulating gonadotropin. Interestingly the probe males also preferred HPx to Sham males, suggesting that the attractant depends also on GnRH. Then we examined the effect of exogeneous gonadotropin injection in HPx males, human chorionic gonadotropin (hCG, acts on the rat LH receptor) and equine chorionic gonadotropin (eCG, acts on the rat FSH receptor). The probe males preferred odor of eCG- but not hCG-injected HPx males than that of saline-injected HPx. The results demonstrate that the attractivity of castrated males was produced by GnRH and FSH elevated by a lack of androgen. (COI:No)

### PP-359

### The nociceptive pain control of oxytocin in the hypothalamo-neurohypophisial/-spinal pathway in the knee osteoarthritis model rats

Haruki Nishimura[1], Makoto Kawasaki[1], Kazuhiko Baba[1], Naofumi Ikeda[1], Takanori Matsuura[1], Hitoshi Suzuki[1], Teruaki Fujitani[1], Yoshiaki Yamanaka[1], Hideo Onishi[1], Mitsuhiro Yoshimura[2], Takashi Maruyama[2], Kazuaki Nishimura[2], Kenya Sanada[2], Yoichi Ueta[2], Akinori Sakai[1] ([1] *Department of Orthopaedic Surgery, School of Medicine, University of Occupational and Environmental Health,* [2] *Department of Physiology, School of Medicine, University of Occupational and Environmental Health*)

**Object:** The neurological reaction of oxytocin (OXT) in the hypothalamo-neurohypophisial/-spinal pathway using the knee osteoarthritis (OA) model rats was evaluated.
**Methods:** The right knee OA was induced by intra-articular injection of 1mg/0.05mL mono-iodoacetate. First, with male Wistar rats, the nociceptive thresholds were measured. These rats were perfused or decapitated at 28 days after injection for immunohistochemistry or *in situ* hybridization. Second, with male OXT-mRFP1 transgenic rats, these rats were perfused at 28 days after injection for evaluation of OXT-mRFP1 appearance in the hypothalamus and spinal cord.
**Results:** In knee OA rats, the nociceptive thresholds were significantly decreased. In addition, the number of the FosB-LI positive cells in the hypothalamus and L4 ipsilateral spinal dorsal horn were significantly larger. Further, the expressions of OXT mRNA and OXT-mRFP1 in the dorsal-parvo paraventricular nucleus and the number of OXT-mRFP1 positive granules in the L4 ipsilateral spinal dorsal horn were significantly larger.
**Conclusion:** Our results suggested the haypothalamo-spinal, not hypothalamo-neurohypophysial, pathway of OXT was activated by knee OA. (COI:No)

### PP-360

### Development of non-contact measuring system of pulse wave of rodents using RGB camera to monitor the activities of autonomic nervous system

Norio Iijima[1], Masato Takahashi[2], Ryo Takahashi[2], Reimei Koike[2], Takeshi Yamaguchi[1], Norimichi Tsumura[2] ([1] *Center for Medical Research, International University of Health and Welfare,* [2] *Graduate School of Engineering, Chiba University*)

Most of measuring methods to detect biological information such as pulse wave require direct contact with the measuring targets giving non-negligible impacts on them. As for application of remote photoplethysmography (rPPG) technique to human, a system has already been developed to obtain biological information even in non-contact, non-invasive and anesthesia-free conditions, in which, only the blood color is extracted from the multicolor images obtained by RBG camera for the purpose of detection of pulse wave. In this study, we applied rPPG technique to rodents devising following points.
1) Transparent acrylic floor was installed for video recording of the palms and soles of the rodents from under the floor considering the fact that their bodies are covered with hair.
2) The pulse of rats is about 300/min which is faster than human pulse that is about 60-70/min, thus, the images were taken with 250fps.
The pulse wave came out of the signals of the processed image, which peak corresponds to the R-peak in the ECG result. We consider that the pulse wave information obtained from the image can be used for analysis on activity of autonomic nervous system.
(COI:Properly Declared)

### PP-361

### Distributions of sympathetic nerve terminals and adrenergic receptors in the rat kidney

Seishi Maeda[1], Yusuke Minato[1], Sachi Kuwahara-Otani[1,2], Hideshi Yagi[1] ([1] *Dept. Anat. Cell Biol., Hyogo College Med.,* [2] *General Edu., Hyogo Univ. Health Sci*)

The renal function is affected by sympathetic nerves to increase blood pressure and regulate fluid homeostasis. On the other hand, pharmacological effects of antihypertensive drugs also affective to these functions. Adrenergic receptors (ARs) are classified into three groups and nine subtypes, however, little is known about the relationships between renal nerve terminals and ARs. In this study, we examined the distributions of renal ARs and nerve terminals by immunohistochemistry. Renal nerve axons had varicosities and attached to the arterioles and urinary tubules. ARs were observed in arteries ( $\alpha$ 1A, $\alpha$ 1D, $\alpha$ 2A, $\beta$ 2), proximal ( $\alpha$ 1B, $\beta$ 1) and distal tubules ( $\alpha$ 2B), and collecting ducts ( $\alpha$ 1A, $\alpha$ 1D, $\beta$ 1). Additionally, they were also found in the renal nerve terminals ( $\alpha$ 2C) and glomeruli ( $\alpha$ 1D, $\beta$ 2). They were revealed that some of them were closely localized to the terminals, while others showed no positional association with them. These results suggest that the activation of ARs includes the direct stimulation by renal nerve terminals and the access of catecholamines from plasma and urine, and that the renal functions may be regulated by various AR stimulation pathways in the kidney. (COI:No)

### PP-362

### Immunohistochemical features of substance P (SP)-containing intrinsic neurons in the rat esophagus

Ryo Morishita[1], Ryoichi Yoshimura[1], Hirohumi Kuramoto[1] ([1] *Cell Function, Applied Biology, Kyoto Inst. Tech*)

Substance P (SP)-containing neurons in the gastrointestinal tract are involved in secretion and motility. SP intrinsic neurons also occur in the myenteric plexus of the esophagus, but their number and function are unclear. In this study, we examined the number and distribution of SP neurons in the rat esophagus using immunohistochemistry to discuss their functional roles. In wholemount preparations, the number of SP neurons in the entire esophagus was 826±147 (n=8), decreasing from the cervical toward abdominal esophagus. Double staining showed that 95% of esophageal SP neurons were choline acetyltransferase (ChAT) positive, while 1% of them were nitric oxide synthase (NOS) positive, indicating that most of SP neurons are cholinergic. In frozen sections, numerous SP nerve fibers were found in the muscularis mucosa, most of which were ChAT positive, but a few were NOS positive. Some SP nerve fibers were also present in the lower esophageal sphincter (LES), where a few were positive for ChAT or NOS. The present results suggest that ChAT/SP intrinsic neurons in the rat esophagus project to the muscularis mucosa and LES as excitatory motor neurons to modulate esophageal motility. (COI:No)

### PP-363

### Effects of microstimulation of the peripheral sympathetic nerve on glucose uptake in normal chow- and high-fat-fed rats

Daisuke Sato[1], Jota Amarume[2], Yoshihiro Ito[3], Ryouichi Banno[4], Masataka Kusunoki[4] ([1] *Department of Biochemical Engineering, Graduate School of Science and Engineering, Yamagata University,* [2] *Department of Bio-Systems Engineering, Graduate School of Science and Engineering, Yamagata University,* [3] *Department of CKD Initiatives, Nagoya University Graduate School of Medicine,* [4] *Research Center of Health, Physical Fitness and Sports, Nagoya University*)

It was pointed out that sympathetic nerve activity is involved with glucose uptake. In the present study, we evaluated the effects of electrical microstimulation (MS) of peripheral sympathetic nerve on glucose uptake. Male rats were divided into 2 groups: normal group fed on a standard laboratory chow (n=5) and HFF group fed on a high-fat diet (n=5). Under anesthetic condition, glucose uptake was assessed as glucose infusion rate (GIR) measured before (baseline), during, and after the MS with euglycemic clamp. Sympathetic nervous signal in the unilateral sciatic nerve was detected with a microelectrode, and then the MS was conducted via the electrode. As a result, GIR was significantly increased with the MS in both the groups ($P<0.01$), and the rate remained high after the termination of the MS ($P<0.01$). Although GIR in the HFF group was significantly lower ($P<0.01$) than that in the normal group with or without the MS, the MS increased GIR to the level close to that at baseline in the normal group. In addition, the MS did not change plasma insulin level. The results suggest that the MS enhances glucose uptake independently of insulin secretion even under the insulin resistance. (COI:No)

### PP-364

### Interactions between oxytocin and parasympathetic vasodilation during trigeminal afferent inputs

Rina Ishikawa[1], Toshiya Sato[1], Kouhei Mito[1], Ramadhani Ratna[1], Hisayoshi Ishii[1] (*[1]Div of Physiol, Dept of Oral Biol, Sch of Dent, Health Sci Univ of Hokkaido*)

Oxytocin regulates reproductive behavior in women, and mother-infant interactions. Similar levels of blood plasma oxytocin have been reported in men and women, but its physiological functions are unclear. Current reports suggest that oxytocin modulates autonomic cardiovascular responses. Parasympathetic vasodilation evoked by trigeminal-mediated reflex may be important for orofacial hemodynamics because the response induces a marked orofacial blood flow increase. However, the humoral regulatory system of parasympathetic vasodilation has not been evaluated. we explored the effect of oxytocin onblood flow in the masseter muscle (MBF; cholinergic) and the lower lip (LBF; noncholinergic) during trigeminal afferent stimulation in-anesthetized rats. Electrical stimulation of the lingual nerve elicited increases of MBF and LBF. Oxytocin inhibited these increasesin a dose-dependent manner and the effect was greater in MBF than in LBF.OXY receptor mRNA was higher in the masseter muscle and lower lip, than in the submandibular gland. These results suggest that oxytocin inhibits parasympathetic vasodilation and this inhibition is more effective on cholinergic than noncholinergic mechanisms. (COI:Properly Declared)

### PP-365

### Involvement of sex hormones in sexually dimorphic response of colorectal motility to noxious stimuli in rats

Kazuhiro Horii[1], Ayaka Onishi[1], Takahiko Shiina[1], Yasutake Shimizu[1] (*[1]Lab Vet Physiol, Unit Grad Sch Vet Sci, Gifu Univ, Gifu, Japan*)

We have previously demonstrated noxious stimuli enhance colorectal motility via descending pain inhibitory pathways in male, but not female, rats. The aim of this study is to clarify involvement of sex hormones in the sexually dimorphic response. We used an *in vivo* method to record colorectal motility in anesthetized rats. Administration of a noxious stimulant capsaicin into the colorectal lumen enhanced colorectal motility in male rats. Orchiectomy had no effect on the capsaicin-induced response. On the other hand, intracolonic injection of capsaicin did not enhance colorectal motility in female rats. However, the colorectal response to capsaicin became evident after ovariectomy, suggesting involvement of female sex hormones. In accordance,17beta-estradiol ($E_2$) treatment abolished capsaicin-induced response in orchiectomized male rats. The impaired motility response in $E_2$-treated orchiectomized males was restored by a pre-injection of $GABA_A$ receptor antagonist into the lumbosacral spinal cord. These results suggest GABAergic descending inhibitory regulation would be manifested by the action of $E_2$, resulting in unresponsiveness of the colorectum to noxious stimuli in female rats. (COI:No)

### PP-366

### Involvement of dopamine D1 receptors in the nucleus of the solitary tract in stress-induced hypertension and its counteracting process by daily exercise

Ko Yamanaka[1], Makoto Suzuki[1], Keisuke Tomita[1,2], Miwa Takagishi[3], Kei Tsukioka[1], Sabine Gouraud[4], Hidefumi Waki[1] (*[1]Dept Physiol, Grad Sch Health and Sports Sci, Juntendo Univ, Chiba, Japan, [2]Faculty of Med Sci, Teikyo Univ of Sci, Tokyo, Japan, [3]Dept Therapeutic Health Promotion, Kansai University of Health Sciences, Osaka, Japan, [4]Dept Biol, Faculty of Sci, Ochanomizu Univ, Tokyo, Japan*)

The nucleus of the solitary tract (NTS) is a key structure in stress-induced hypertension and destress induced by daily exercise. In this study, we examined the gene expression profiles in the NTS and aimed to determine its function in the cardiovascular regulation of related genes. Wistar rats (n = 18) were allocated to three groups: sedentary (SED), restrained stress for 1 h a day over 3 weeks (ST), and restrained stress + voluntary exercise (ST+EX). The results showed that the blood pressure was significantly higher in the ST than in the SED but did not increase in the ST+EX. Using PCR array, we found that the expression levels of six genes in the NTS, such as the dopamine D1 receptor (D1R) gene *Drd1*, were significantly different among groups. Microinjection of the D1R agonist into the NTS in anesthetized rats induced hypotensive effects. Furthermore, we observed the D1R expression in the NTS and projections from tyrosine-positive cells in the midbrain using immunohistochemistry and retrograde tracing methods. These results suggest that dopamine D1R in the NTS may be involved in the mechanism underlying both stress-induced hypertension and destress induced by daily exercise. (COI:No)

### PP-367

### Effects of chronic restraint stress and stress prevention through exercise on gene expression profiles of the hypothalamus

Keisuke Tomita[1,2], Ko Yamanaka[1], Kei Tsukioka[1], Thu Nguyen Van[1], Sabine Gouraud[3], Hidefumi Waki[1] (*[1]Grad Sch Health and Sports Sci, Juntendo Univ, Chiba, Japan, [2]Faculty of Med Sci, Teikyo Univ of Sci, Yamanashi, Japan, [3]Faculty of Sci, Ochanomizu Univ, Tokyo, Japan*)

[Aims] It has been reported that chronic restraint stress (CRS) induces hypertension, whereas exercise has anti-stress and antihypertensive effects. The hypothalamus (HYP) plays important roles in regulating the autonomic nervous system including cardiovascular response. This study aimed to identify the HYP gene associated with CRS and exercise. [Methods] Wistar rats were allocated into three groups (n = 6, each): CRS, CRS + voluntary exercise, and control groups. CRS was produced by immobilization of these rats for three weeks (one hour per day, five days per week). Differentially expressed genes (DEG) in the HYP among the three groups were investigated using gene ontology (GO) and pathway analyses. [Results] DEG were associated with GO terms such as neuron development, neuron apoptotic, and glutamatergic transmission. Moreover, these genes were found to be associated with various cardiovascular and nervous system diseases. [Conclusions] These results suggest that altered gene expression of the HYP may be involved in the mechanisms of stress-induced high blood pressure and preventive effects of exercise.
(COI:No)

### PP-368

### Effect of chronic electrical stimulation of the superior laryngeal nerve on bone mineral density in the ovariectomized rats

Kaori Iimura[1], Nobuhiro Watanabe[1], Philip Milliken[2], Arun Sridhar[2], Harumi Hotta[1] (*[1]Department of Autonomic Neuroscience, Tokyo Metropolitan Institute of Gerontology, [2]Galvani Bioelectronics, United Kingdom*)

Electrical stimulation of the superior laryngeal nerve (SLN) induces calcitonin (CT) secretion from the thyroid gland in anesthetized rats. The aim of this study was to examine whether the electrical stimulation of SLN increases systemic CT in conscious rats and to then clarify effects of chronic SLN stimulation on bone mineral density (BMD) in ovariectomized (OVX) rats. Cuff electrodes were implanted bilaterally on SLNs and were stimulated (0.5ms, 90 microampere) at 40 Hz for 8 min. Immunoreactive CT (iCT) was quantified in systemic blood plasma collected from via a venous catheter. SLN stimulation increased iCT concentration. For chronic SLN stimulation, stimuli were applied intermittently for 3-4 weeks, starting at five weeks after OVX, and thereafter BMD of the femur and tibia was measured. BMD in metaphysis of tibia and femur in chronically SLN-stimulated rats was 4-5% higher than in sham-stimulated rats. The results suggest chronic electrical stimulation of the SLNs partially inhibits bone loss in OVX rats, presumably due to an increase in systemic CT. This study was funded by Galvani Bioelectronics UK.
(COI:Properly Declared)

### PP-369

### Differences in autonomic vascular responses between free and attached gingiva during trigeminal afferent stimulation in rats

Yunosuke Okada[1], Masato Saito[1], Hisayoshi Ishii[2] (*[1]Div. Pediatr., Dept. Sch. Dent., Health Sci Univ.Hokkaido, [2]Div of Physiol, Dept of Oral Biol, Sch of Dent, Health Sci Univ of Hokkaido, Japan*)

Trigeminal afferent stimulation is known to induce parasympathetic vasodilation in gingiva resulting in a broad and rapid increase in blood flow. This observation suggests that parasympathetic vasodilation is important in maintaining gingival hemodynamics and function. Gingiva is classified into two types: free and attached. These gingivae have been reported to show differences in their capillary networks. However, no reports have described a site specificity of hemodynamics in gingiva. Here, we explored changes in free gingiva blood flow (FBF) and attached gingiva blood flow (ABF) during electrical stimulation of the central cut end of the lingual nerve (LN) in anesthetized rats. Electrical stimulation of the LN elicited frequency-dependent increases in FBF and ABF. The increase in FBF was greater than that in ABF. These increases were significantly reduced by hexamethonium and atropine. Activation of the superior cervical sympathetic trunk (CST) reduced FBF and ABF, and inhibited these increases evoked by LN stimulation. Our results suggest that parasympathetic vasodilation is more involved in the regulation of FBF than ABF, and that excess CST activation inhibits the response. (COI:No)

### PP-370

### Immunohistochemical study on the distribution of orexinergic nerve fibers and receptors in the superior salivatory nucleus

Yoshihiro Mitoh[1,3], Tadasu Sato[2], Takehiro Yajima[2], Hiroyuki Ichikawa[2], Motoi Kobashi[1], Ryusuke Yoshida[1] ([1]*Dept Oral Physiol, Okayama Univ Grad Sch Med Dent Pharm Sci,* [2]*Div Oral Craniofac Anat, Tohoku Univ, Grad Sch Dent,* [3]*ARCOCS*)

Orexins (OXA and OXB) are neuropeptides localized in the hypothalamus, which influences sleep and arousal, appetite. The lateral hypothalamus (feeding center) projects directly to the superior salivatory nucleus (SSN) that is the primary parasympathetic center for the submandibular and sublingual salivary glands. Here, we immunohistochemically examined the distribution of orexinergic nerve fibers and receptors in the rat SSN neurons retrogradely labeled with Fast blue (FB) from the chorda-lingual nerve that contains preganglionic fibers of SSN neurons. OXA- and OXB-immunoreactive (-ir) fibers were seen throughout the SSN. About half of FB-positive neurons had pericellular OXA- and OXB-ir fibers. For OX receptors (OX1R and OX2R), the majority of FB-positive SSN neurons contained OX1R- or OX2R-immunoreactivity. In addition, half of FB-positive SSN neurons that were immunoreactive for OX1R and OX2R had pericellular OXA- and OXB-ir fibers, respectively. Patch-clamp recordings revealed generation of action potentials in SSN neurons by application of OXs. Activation of OX-producing neurons may induce the salivation by promoting SSN neuronal activity, in addition to facilitating feeding. (COI:No)

### PP-371

### Higher parasympathetic nervous activity after waking relates psychological stress in older women.

Kentaro Taniguchi[1], Naoya Okumura[2], Naoya Jinno[2], Makoto Sata[3], Akito Shimouchi[2] ([1]*Nagahama Institute of Bio-science and Technology,* [2]*College Life and Health Science, Chubu University ,* [3]*National Cerebral and Cardiovascular Center* )

Heart rate variability can analyze mental stress with non-invasive and quantitatively way. It is well established that High frequency (HF) reflects parasympathetic nervous activity. In this study, we examined whether or not transitional HF alterations before and after night sleep represent psychological stress. Forty-five older women (aged 60 or more) participated in this study. Their HF trends were obtained as spectral power in every minute for 24 hours during the free-moving day. We compared the averaged HF during 60min before and after night sleep in each individuals. Subjects were divided into two groups with higher HF either before night sleep (n=19) or after waking (n=26). Cornel Medical Index was used to determine the degree of physical and psychological symptoms. In the older women with higher HF after waking, their scores of fatigability, depression, anxiety, and their subtotal scores of physical and psychological symptoms, were significantly higher than those of the subjects with higher HF before night sleep, respectively. These results suggest that older women with higher HF after waking complicate fatigability or psychological stress. (COI:No)

### PP-372

### Involvement of the dopaminergic system into autonomic cardiovascular responses evoked by activation of the lateral habenula

Yuma Sato[1,2], Tri Doan[1,3], Masayuki Matsumoto[4,5], Tadachika Koganezawa[1,5] ([1]*Department of Physiology, Faculty of Medicine, University of Tsukuba, Tsukuba, Ibaraki, Japan,* [2]*Master's Program in Medical Sciences, Graduate School of Comprehensive Human Sciences, University of Tsukuba, Tsukuba, Ibaraki, Japan,* [3]*Doctoral Program in Biomedical Sciences, Graduate School of Comprehensive Human Sciences, University of Tsukuba, Tsukuba, Ibaraki, Japan,* [4]*Department of Cognitive and Behavioral Neuroscience, Faculty of Medicine, University of Tsukuba, Tsukuba, Ibaraki, Japan,* [5]*Transborder Medical Research Center, University of Tsukuba, Tsukuba, Ibaraki, Japan*)

Stress behavior like "fight or flight" and "freezing" accompanies autonomic cardiovascular changes. The lateral habenula (LHb), a main input nucleus of the midbrain dopaminergic system, is known to play crucial roles in stress behavior. However, it is unclear whether the LHb-dopamine circuit is involved in the stress-induced cardiovascular responses. Here we investigated effects of electrical stimulations of the LHb on blood pressure and heart rate with dopaminergic receptor blockers. We used Wistar male rats that were anesthetized by urethane. Administration of clozapine, a non-selective dopamine receptor antagonist, attenuated the LHb stimulation-induced bradycardia and pressor responses. We also administered selective dopamine receptor blockers (SCH23390, a D1 and D5 receptors antagonist; sulpiride, a D2 and D3 receptors antagonist; L-745,870, a D4 receptor antagonist). In the presence of each antagonist, the LHb stimulation-induced pressor responses were attenuated but the bradycardia was not affected. These results suggested that multiple subtypes of dopaminergic receptors are involved in the LHb-related cardiovascular responses to stress events. (COI:No)

### PP-373

### The lateral habenula regulates cardiovascular activity via the serotonergic system

Huu Tri Doan[1,2,6], Yuma Sato[1,3], Masayuki Matsumoto[4,5], Tadachika Koganezawa[1,5] ([1]*Department of Physiology, Faculty of Medicine, University of Tsukuba, Tsukuba, Ibaraki, Japan,* [2]*Doctoral Program in Biomedical Sciences, Graduate School of Comprehensive Human Sciences, University of Tsukuba, Tsukuba, Ibaraki, Japan,* [3]*Master's Program in Medical Sciences, Graduate School of Comprehensive Human Sciences, University of Tsukuba, Tsukuba, Ibaraki, Japan,* [4]*Department of Cognitive and Behavioral Neuroscience, Faculty of Medicine, University of Tsukuba, Tsukuba, Ibaraki, Japan,* [5]*Transborder Medical Research Center, University of Tsukuba, Tsukuba, Ibaraki, Japan,* [6]*Center for Advanced Training in Clinical Simulation, University of Medicine and Pharmacy at Ho Chi Minh City, Ho Chi Minh City, Vietnam*)

The lateral habenula (LHb) is thought to play a critical role in negative stimuli-induced behavioral and autonomic responses including cardiovascular responses. However, the question remains as to how the LHb regulates the cardiovascular system to aversive stimuli. We investigated regulation of the LHb to the stress-induced cardiovascular activity with focusing on the serotonergic system. We employed anesthetized male Wistar rats to assess cardiovascular responses. We observed that electrical stimulation of the LHb evoked bradycardia and pressor responses. Intravenous administration of a nonselective 5-HT receptors antagonist, methysergide, or a 5-HT$_2$ receptors antagonist, mianserin, attenuated these responses. Administration of low dose of a 5-HT$_{1A}$ receptor antagonist, NAD-299, enhanced these responses, but high dose of the drug attenuated them. Inhibiting the DRN neurons with microinjection of muscimol, a GABA$_A$ receptor agonist, attenuated the pressor response but did not change the bradycardia. These results suggest that the LHb regulates cardiovascular autonomic system via the serotonergic system, and especially the blood pressure regulation might be mediated by the DRN. (COI:No)

### PP-374

### Regulation of neuronal excitability by multiple G-protein-coupled receptors in rat intracardiac ganglia

Hitoshi Ishibashi[1], Shiho Arichi[1], Aya Sato[2] ([1]*Dept Physiol, Sch Allied Health Sci, Kitasato Univ,* [2]*Dept Pediatrics, Shiga Univ Med Sci*)

The cardiac plexus, which contains parasympathetic ganglia, plays an important role in regulating cardiac function. We have previously shown that multiple G$_{q/11}$-protein coupled receptors including histamine, muscarinic, and adrenergic receptors excite intracardiac neurons. However, the underlying mechanisms remains to be clarified. In the present study, therefore, the effects of histamine, bradykinin and muscarinic agonist oxotremorine M on rat intracardiac ganglion neurons were investigated using perforated patch-clamp recordings. Under voltage-clamp conditions, application of these agonist evoked an inward current that was potentiated by extracellular $Ca^{2+}$ removal and attenuated by extracellular $Na^+$ replacement with N-methyl-D-glucamine. This implicated the involvement of non-selective cation channels, which given the link between these receptors and G$_{q/11}$-protein-phospholipase C signalling, were suspected to be transient receptor potential canonical (TRPC) channels. In fact, TRPC blockers $Gd^{3+}$ and ML204 markedly inhibited the depolarizing response. These results suggest that TRPC channels serve as the predominant mediator of neuronal excitation by G$_{q/11}$-coupled receptor. (COI:Properly Declared)

### PP-375

### Effects of cervical sympathetic trunk and renal sympathetic nerve activity on the regulation of cerebral blood flow during head-down postural rotations

Noriko Matsuo[1], Yosuke Nakamura[1], Felix Ojeiru Ezomo[1], Satoshi Matsuo[1], Yasuaki Kawai[2] ([1]*Div of Adaptation Physiol, Dept of Physiol, Tottori Univ Faculty of Med, ,* [2]*YMCA College of Medical & Human Services in Yonago*)

This study attempts to clarify the neural control of cerebral blood flow (CBF) during head-down postural rotation (HDR) in urethane-anesthetized rats. The animals were tilted to a 45° head-down position in 5 s, and were maintained in that position. HDR induced a transient decrease in the mean arterial blood pressure (ABP) after the onset of HDR. The pressure returned to the pre-HDR level within one minute. The administration of phenoxybenzamine (PB) eliminated the HDR-elicited decrease in ABP, suggesting that the decrease was elicited by the suppression of $\alpha$-adrenergic vascular tone. Renal sympathetic nerve activity (RSNA) was suppressed at $2.3 \pm 0.4$ s after HDR onset, followed by a decrease in ABP. Cervical sympathetic trunk (CST) activity (CSTA) did not change significantly during HDR. CBF did not change significantly during HDR in the control, after the administration of PB, or after denervation of CSTs. These results suggest that the impact of CSTA on CBF is likely to be limited against a rapid increase in CBF due to HDR-elicited cephalad fluid shift and that CBF autoregulation proceeds through an alternative mechanism involving the myogenic properties of cerebral vessels. (COI:No)

### PP-376

### Effects of win or lose situation on performance and autonomic cardiovascular responses during cycling exercise using virtual reality

Shouta Katsuki[1], Yoshimitsu Koumura[1], Hidefumi Waki[2], Ko Yamanaka[1] ([1]*Graduate School of Health and Sports Science, Juntendo University,* [2]*Institute of Health and Sports Science and Medicine, Juntendo University*)

Many sports competitions have the participants compete with others in terms of performance. We aimed to test the hypothesis that the presence and behavior of competitive opponents affect the athletic performance through emotional and autonomic responses to changes. We measured athletic performance (cadence), blood pressure, and heart rate of the participants (n = 13) while performing cycling exercise at a constant speed (60 rpm) in a virtual reality environment that mimicked a situation in which the participants were "overtaking (win)" or "overtaken (lose)" by competitors. We observed that cadence, mean and diastolic blood pressures, and heart rate 2.5 s immediately before the loss situation were significantly higher than in the win situation. In addition, multiple linear regression analysis showed that blood pressure significantly correlated not only with the effects of motor performance (cadence) but also with others (win or lose situation) in a time-dependent manner. These results suggest that the presence and behavior of competitors elicit emotion and affect motor performance and autonomic responses. (COI:No)

### PP-377

### Abdominal afferent vagal activation by CCK signal in anesthetized mice

Mamoru Tanida[1], Yusaku Iwasaki[2], Liu Li[1], Yuichi Kuda[1], Kunichika Tsumoto[1], Toshishige Shibamoto[1], Yasutaka Kurata[1] ([1]*Dept of Physiol*[2]*, Kanazawa Med Univ,* [2]*Lab of Animal Sci, Kyoto Pref Univ*)

CCK (cholecystokinin) is a feeding suppressor hormone. Here, we examined effects of intravenous injection of CCK on afferent vagal nerves in the abdominal organs in anesthetized mice after overnight fasting. Firstly, CCK injection dose-dependently stimulated afferent vagal nerve in the stomach branch, and that it increased vagal afferents of celiac blanch and hepatic blanch. And, the gastric vagal response was blocked by selective agonist of CCK-A receptor. On the other hand, vagal afferent activation by CCK was preserved in the leptin receptor-deficient mice. It seems that afferent vagal nerve activation by CCK exits independently of leptin pathway. Secondly, we examined effects of CCK injection on afferent vagal signals in the obese mice fed by high-fat diet (HFD mice). Regarding to celiac vagal afferent activation by CCK, no significant difference was found between the normal mice and the HFD mice. In addition, there was not significant difference in the expression level of the CCK-A receptor in the nodose ganglion between the both groups. Thus, these data suggest that CCK resistance in the celiac afferent vagal signals may not detect in the dietary obesity. (COI:No)

### PP-378

### Influence of occlusal contact state on posture control and physical fitness of elite athletes

Mutsumi Takahashi[1], Yougetsu Bando[2], Katsuhiko Kitaoka[3,4], Shinnosuke Kimura[4,5], Yoshihide Sato[1] ([1]*Dept Physiol, Nippon Dent Univ, Niigata, Japan,* [2]*BANDO Dental Clinic, Ishikawa, Japan,* [3]*Dept Orthopedic Surgery, Kijima Hospital, Ishikawa, Japan,* [4]*Japan Handball Association, Tokyo, Japan,* [5]*weave KOMATSU, Ishikawa, Japan*)

The aim of this study was to clarify the effect of occlusal state on the posture control and physical fitness of elite handball athletes. Dental Prescale was used to evaluate the occlusal stability. Posture control function was evaluated by a gravity center fluctuation meter. Measurements were performed in the mandibular resting position (RP), the intercuspal position (ICP), and wearing a mouthguard (with-MG). Physical fitness tests were a total of nine items that evaluated agility, instantaneous force, muscle strength, jumping power, flexibility, and running ability. Correlations between the occlusal stability and the posture control function were analyzed. Differences in the scores of physical fitness tests with and without a mouthguard were analyzed. Significant correlations were not found between occlusal stability and postural control function. Posture control function was better for with-MG than for RP and ICP. This study suggested that the posture control function was improved by equalizing the occlusal contact state, and that brought about the improvement of physical performance, which is mainly agility, instantaneous power, muscle strength and jumping power. (COI:No)

### PP-379

### Bafilomycin A1 suppresses the repeated cold stress-induced mechanical sensitization in rat thin-fiber muscle afferents

Amane Hori[1], Norio Hotta[1,2], Teruaki Nasu[2], Kimiaki Katanosaka[1,2], Kazue Mizumura[3] ([1]*Graduate School of Life and Health Sciences, Chubu University,* [2]*College of Life and Health Sciences, Chubu University,* [3]*Department of Physiology, Nihon University School of Dentistry*)

Muscle acidification has been reported in a rat model of fibromyalgia, characterized by mechanical hyperalgesia induced by repeated cold stress (RCS). We hypothesized that vacuolar ATPase (V-ATPase)-dependent muscle acidification is involved in RCS-induced muscular mechanical hyperalgesia. Therefore, we investigated whether bafilomycin A1 (BafA1), a V-ATPase inhibitor, could modify the RCS-induced muscle afferent sensitization. Single group IV fiber recording was performed on nerve-muscle preparations of rats exposed to RCS. The mechanical threshold (MT) and magnitude of response to mechanical stimuli in the RCS group were significantly lower ($P<0.01$) and higher ($P<0.05$) than those in the control group, respectively. After injecting BafA1 in the RCS group, the decreased MT and augmented response magnitude significantly ($P<0.05$) increased and decreased, respectively. The data demonstrated that BafA1 suppressed the RCS-induced mechanical sensitization of thin muscular afferents. These results suggest that V-ATPase-dependent sensitization of group IV muscle afferents is involved in RCS-induced muscular mechanical hyperalgesia. (COI:No)

### PP-380

### Does sustained exercise with vocalization lead to increased oxidative stress as well as increased oxygen supply to active muscles?

Hajime Arikawa[1], Tomoyoshi Terada[2], Kanako Yamada[3], Teppei Takahashi[4], Hajime Imai[5], Seiichi Era[6] ([1]*Faculty of Sports and Health Science, Chubu Gakuin University, Japan,* [2]*United Graduate School of Drug Discovery and Medical Information Sciences, Gifu Univ, Gifu, Japan,* [3]*Faculty of Nursing and Rehabilitation, Chubu Gakuin Univ, Seki, Japan,* [4]*Takahashi Dental Clinic, Ichinomiya, Japan,* [5]*Faculty of Education, Gifu Univ, Gifu, Japan,* [6]*Clinical Laboratory Center, Japanese Red Cross Gifu Hospital, Gifu, Japan*)

[Aims] We reported that sustained upper-body exercise with vocalization tended to increase $O_2$ supply to active muscles. Our results suggest that oxidative stress may increase accordingly. In this study, we investigated whether increased $O_2$ supply increases oxidative stress. [Methods] Nine men performed sustained upper-body exercise in two trials: with or without vocalization. We measured the ventilation indexes (e.g., $FetCO_2$) and muscle oxygen status (TSI%) of the triceps during exercise, and the blood $O_2$ saturation (arterial side: $SpO_2$, venous side: venous oxygenation index [VOI]), oxidative stress (d-ROMs test), and rate of perceived exertion (RPE) after exercise. [Results] $FetCO_2$ levels tended to increase. $SpO_2$ showed a significantly suppressed decrease and VOI tended to decrease; thus, more $O_2$ was dissociated. The decrease in TSI% tended to be suppressed. Oxidative stress tended to be high but within the standard value. RPE tended to be high, suggesting the involvement of dyspnea. [Conclusions] The increased oxidative stress was within the standard value, but vocalization tended to cause dyspnea. Increased O2 supply can only be beneficial for short periods of exercise. (COI:No)

### PP-381

### DGK ε -deficient mice show impaired adaptive thermogenesis under cold exposure

Tomoyuki Nakano[1], Kaoru Goto[1] ([1]*Dept. Cell Biol., Yamagata Univ Sch. Med*)

Brown adipose tissue (BAT) is known to play a central role in adaptive thermogenesis via uncoupling protein 1 (UCP1). BAT represents as glucose-consuming organ for heat generation under cold environment. Diacylglycerol kinase (DGK) phosphorylates DG to produce phosphatidic acid. Since DG serves as a second messenger as well as an intermediate metabolite for triglyceride, DGK is thought to participate in signal transduction and energy metabolism. In this study we aim to elucidate functional significance of DGK ε in cold acclimatization. Under cold exposure conditions (6°C, 14 days), DGK $\varepsilon^{-/-}$ mice exhibited lowered levels of rectal temperature and plasma glucose than those of WT mice. Those mice also showed increased BAT mass. In immunoblot analysis, expression levels of UCP1, tyrosine hydroxylase (TH) and GLUT1 were increased in DGK ε -deficient BAT. UCP1 is known to generate heart instead of ATP under the control of TH-immunoreactive sympathetic nerves. These results suggest that DGK $\varepsilon^{-/-}$ mice properly respond to cold exposure via sympathetic activity and UCP1-mediated thermogenesis, but are not able to generate heat sufficient to maintain body temperature. (COI:No)

### PP-382

### Natural herbal estrogen-mimetics (phytoestrogens) promote the differentiation of fallopian tube epithelium into multi-ciliated cells via estrogen receptor beta

Tomohiko Iwano[1], Maobi Zhu[1], Sen Takeda[1] ([1]*Univ. of Yamanashi, Grad. Sch. of Med, Anatomy and cell biology*)

The fallopian tube (FT) lumen is lined with secretory cells and multi-ciliated epithelial cells. Elucidation of the mechanism in the maintenance of homeostasis in FT is an important study that is the basis for understanding and treating problem in pregnancy. Phytoestrogens, which are included in soybeans that we usually take, can affect the FT homeostasis and thereby affect the fecundity. As we showed that estrogen promotes multi-ciliogenesis, the effect of phytoestrogens for cell differentiation was addressed. We added genistein, daidzein, glycitin and coumestrol to primary culture under air–liquid interface condition and assessed the epithelial cell differentiation by immuostainings. All phytoestrogens except for glycitin induced multi-ciliated cell differentiation over secretory cell differentiation. This occured before the downregulation of Notch signaling. Furthermore, the ciliated-cell differentiation was inhibited by ERβ antagonist, PHTPP. Thus, this study suggests that phytoestrogens can act similar to estrogen and have potential beneficial effect of FT homeostasis by facilitating the genesis of multi-ciliated cells in the case of reduced endocrinal estrogen. (COI:No)

### PP-383

### Alternative splicing of cold-inducible RNA-binding protein mRNA in hypothermic animals including hibernator

Yuuki Horii[1,2] ([1]*Division of Animal Experiment, Life Science Research Center, Gifu University,* [2]*Department of Basic Veterinary Science, Laboratory of Physiology, The United Graduate School of Veterinary Sciences, Gifu University*)

Although low temperature damages organ functions of mammals, hibernators can survive even under severe hypothermic conditions less than 10°C. To investigate the mechanisms for tolerance to hypothermia, we focused on cold-inducible RNA-binding protein (CIRP). We revealed that CIRP mRNA is constitutively expressed in various organs of non-hibernating euthermic hamsters with three alternative splicing variants as assessed by RT-PCR. In contrast, hibernating hamster expressed only the short product which contains open reading frame for full-length CIRP. This alternative splicing mechanism might permit rapid expression of CIRP function by switching the splicing pattern during entering hibernation to avoid hypothermic damages. Importantly, the regulation of CIRP expression at the level of alternative splicing was commonly observed in non-hibernators. We found that the shift in splicing pattern was elicited in mice and rats, when mild hypothermia (~28°C) was maintained for few hours. These results suggest that the protective effects of CIRP against a harmful low temperature can be reproduced in non-hibernators, enabling establishment of therapeutic hypothermia. (COI:No)

### PP-384

### Postnatal white-to-brown conversion of adipose tissue in Syrian hamsters

Yuko Okamatsu-Ogura[1], Kazuki Nagaya[1], Junosuke Mae[1], Ayumi Tsubota[1], Junko Kobayashi[2], Kazuhiro Kimura[1] ([1]*Laboratory of Biochemistry, Faculty of Veterinary Medicine, Hokkaido University,* [2]*Laboratory of Histology and Cytology, Faculty of Medicine, Hokkaido University*)

Brown and white adipose tissues (BAT and WAT) are quite different in morphology and function; however, the boundary between these tissues is obscure. In this study, we evaluated the process of BAT formation in Syrian hamsters, which shows postnatal conversion of WAT to BAT. Histological analysis revealed that interscapular fat is occupied with white adipocytes at birth, and progenitors appear and proliferate to fill the whole tissue, and then differentiate into brown adipocytes. Immunostaining for uncoupling protein 1 (UCP1), a responsible protein for BAT thermogenesis, indicated tissue maturation as BAT by postnatal day 14. Consistently, pups before 14-day-old were unable to maintain body temperature at 23°C. Environmental temperature seems not to be critical for BAT formation because it was similar between the pups raised at 23°C and 30°C. Progenitors spontaneously differentiated into brown adipocytes *in vitro*, which was suppressed by co-culture with white adipocytes, indicating that inhibitory factors of progenitor differentiation are secreted from white adipocytes. These results suggest that white adipocyte disappearance is essential for the BAT formation in hamsters. (COI:No)

### PP-385

### What determines the *set value* of our core body temperature?

Yuki Yoshimura[1], Kazuomi Nakamura[2], Akira Futazuki[3], Katsuhiko Mikoshiba[4], Tatsuo Watanabe[1] ([1]*Division of Integrative Physiology, Tottori University Faculty of Medicine,* [2]*Division of Experimental Pathology, Faculty of Medicine, Tottori University,* [3]*Dept. Basic Med Sci., Kobe City College of Nursing,* [4]*SIAIS, ShanghaiTech University*)

Core body temperature ($T_c$) of homeothermal animals is set at around 37°C ; however, little is known about what determines the *set value* (i.e., 37) of $T_c$. We examined the possibility that the $T_c$ in pregnant mice establishes the *set value*of the $T_c$ in their offspring. We cultured fertilized mice embryos *in vitro* at 37°C or 38°C, then transferred these blastocysts into uteri of pseudo-pregnant mice. In 9-weeks old male offspring, the $T_c$ of mice derived from 38°C cultured embryos (38°C-group) was significantly lower than that of the control mice (37°C-group). The RNA-Seq and real-time RT-PCR revealed that the hypothalamus of 38°C-group had expressions of Insulin-like growth factor (Igf-1) and Igf-binding protein 2 (Igfbp2) that were significantly higher than that of the control mice. We established the forebrain-specific Igfbp2 KO (cKO) mice. The cKO mice showed significantly higher Tc than the control mice. These results suggest that Tc value depends on the environmental temperature at an extremely early period of life and that the increase in hypothalamic igfbp2 seen in the 38°C-group inhibited the action of thermogenic Igf-1, leading to the lower Tc in that group. (COI:No)

### PP-386

### Mass spectroscopy of phlorizin contained in the peal of Fuji apples cultivated using neither pesticides nor fertilizers and isolation of endophytic fungi from the peal

Koki Ono[1], Ryosuke Sugita[2], Machiko Imai[1], Kazuaki Tanaka[3], Katsuya Yamada[1] ([1]*Hirosaki University Graduate School of Medicine, Department of Physiology,* [2]*Iwate University, the United Graduate School of Agricultural Sciences,* [3]*Hirosaki University, Faculty of Agriculture and Life Science, Department of Applied Biology and Food Sciences*)

As the saying goes, an apple a day keeps the doctor away, phlorizin, a polyphenol abundantly contained in the apple peal, has anti-cancer/hypoglycemic effects. Its derivatives indeed are used widely as a new class of orally active anti-diabetic drugs. However, health risk of pesticides would be a matter of concern when eating the whole fruit. In Hirosaki area, there is an orchard wherein apples have been cultivated with neither fertilizers nor pesticides for over 30 years. No study had reported polyphenols in fruits of such "natural" farming. Here we show the phlorizin content in the peal of Fuji of this farm by LC-MS/MS. Fuji specially grown with reduced pesticide use in a nearby orchard was used as a control. The phlorizin content in the "natural" peal was significantly larger than the control in all months tested, whereas the size and weight of the fruits were smaller. Phlorizin is a phytochemical against insects/animals, but is a carbon source for most fungi. Interestingly, we found endophytic fungi in the "natural" peal and determined the DNA sequences for species identification. Phytochemicals and endophytes may cooperate in the peal to protect the fruit from intruders. (COI:Properly Declared)

### PP-387

### Changes in salivary IgA secretion in heat-acclimated rats

Kentaro Matsuzaki[1], Naotoshi Sugimoto[1,2], Islam Rafiad[1,3], Md Emon Hossain[1,4], Eri Sumiyoshi[1], Masanori Takakura[1,5], Osamu Shindo[1] ([1] *Dept. Environmental Phys. Facult. Med. Shimane Univ.,* [2]*Dept. Phys. Grad. Sch. Med. Sci. Kanazawa Univ.,* [3]*Dept Psy. Sch. Med. Yale Univ.,* [4]*Dept. Biochem Mol Genet. Univ. Alabama.,* [5]*Dept. Nutritional Phys. Facult. Pharm. Sci., Josai Univ.* )

Salivary immunoglobulin A (IgA) serves as the first line of defense in protecting the oral cavity and upper respiratory tract. Chronic exposure to moderate heat provides heat acclimation, which alters salivary functions. However, the changes in salivary IgA secretion in heat-acclimated rats are unclear. In this study, we investigated salivary IgA secretion and the expression of polymeric Ig receptor (pIgR), a key mediator of mucosal IgA secretion, in the submandibular glands (SMGs) of heat-acclimated rats. Male Wistar rats were subjected to an ambient temperature (Ta) of 32 ± 0.2 °C for 5 days (HE) for heat acclimation, while control rats were maintained at an Ta of 24 ± 0.1°C (CN). The rats were then anesthetized, pilocarpine (0.5 mg/kg) was intraperitoneally injected, and saliva was collected. Then, the SMGs and plasma were sampled. The salivary IgA concentration and IgA flow rate were significantly greater in HE than in that of CN. Similarly, SMG pIgR expression was significantly higher in HE. Heat acclimation may enhance oral immunity through improved salivary IgA secretion and pIgR upregulation in the SMGs. (COI:No)

### PP-388

### The effects of ultrafine bubble or microbubble exposure during bathing on physical parameters.

Hiroshi Iida[1], Eri Takahashi[1], Noriyuki Koibuchi[2] (*[1]Kinboshi Inc., [2]Department of Integrative Physiology, Gunma University Graduate School of Medicine*)

The effects of microbubbles (MB), ultrafine bubbles (UFB) or combined MB and UFB (UMB) exposures on physical parameters were studied in adult males who bathed for 10 minutes at 40°C with either MB, UFB, UMB. Parameters were: sublingual and skin temperatures, blood pressure, SpO2, skin pH and moisture, and pseudo lipid pollutant removal. The sublingual temperature of UMB group was higher than UFB and MB groups during bathing. The temperatures of UFB and UMB groups at 15 min after bathing were still higher than those before bathing. The pseudo lipid pollutant removal tended to be highest in the UFB group. Other parameters showed no significance. MB may inhibit skin heat inflow by forming a bubble layer, whereas UFB may produce heat by physically acting to skin. Since UFB can infiltrate through MB, physical skin stimulation may cause higher body temperature in the UMB group. In the UFB group, MB removal from UMB may decrease the UFB level, causing a weaker effect. These results indicate that UMB causes a thermal effect through UFB physical effect. *Gunma University Hospital Clinical Research Review Board UMIN-CTR No. UMIN000029182. *COI: properly declared.

(COI:Properly Declared)

### PP-389

### Effects of nanoplastics exposure on the rat physiological and biological parameters.

Thuy Linh Pham[1,2], Ko Yamanaka[5], Yasunori Miyamoto[1,3,4], Hidefumi Waki[5], Sabine Gouraud[2,3] (*[1]Graduate School of Humanities and Sciences, Ochanomizu University, Tokyo, Japan, [2]Program for Leading Graduate School, Ochanomizu University, Otsuka, Bunkyo-ku, Tokyo, Japan, [3]Department of Biology, Ochanomizu University, Otsuka, Bunkyo-ku, Tokyo, Japan, [4]Institute for Human Life Innovation, Ochanomizu University, Otsuka, Bunkyo-ku, Tokyo, Japan, [5]Department of Physiology, Graduate School of Health and Sports Science, Juntendo University, Inzai-city, Chiba, Japan*)

Recent data suggests that humans are regularly exposed to plastic nanoparticles (NPs) via the food chain. Moreover, NPs were shown to cross biological membranes, alter gene expression, and trigger behavior changes in marine animals. However, the impact of NPs on terrestrial mammalian health is poorly studied. In this study, we examined physiological and biological parameters in rats orally exposed to NPs chronically.
Five weeks-old male Wistar rats were trained to drink a 3% sucrose solution from a syringe and were fed daily with the solution supplemented with 50 nm polystyrene NPs (12.5 mg/kg body weight) either amino-modified (NP+) or carboxy-modified (NP-), or with water (CONTROL), for two months. Body weight, blood pressure, water and food intake, and physicochemical characteristics of urine were regularly recorded.
Whist the basal level of blood pressure exhibited a tendency to decrease in NP groups, the urine osmolality of those rats significantly increased after two months of exposure. Our data suggests that a chronic oral exposure to NPs affects osmoregulation mechanisms in rats. Whether the nervous system, cardiovascular, or renal function is affected is still unknown.

(COI:No)

### PP-390

### Analysis of Vitamin E contents in plasma and tissues in a mammalian hibernator, Syrian hamster.

Reo Otsuka[1,2], Masamitsu Sone[1,2], Yoshifumi Yamaguchi[1,2] (*[1]Division of Biosphere Science, Graduate School of Env. Science, Hokkaido University, [2]Hibernation metabolism, physiology, and development Group, Institute of Low Temperature Science, Hokkaido University*)

Mammalian hibernators, including Syrian hamsters and ground squirrels, can survive under prolonged hypothermia and rewarming from it during hibernation. This is in contrast to other non-hibernators, including human and rats, which suffer from organ dysfunctions and the cell death accompanying lipid peroxidation under such conditions. However, its mechanisms of resistance to hypothermia remain to be elucidated.
In this study, we focused on an antioxidant Vitamin E that can prevent lipid peroxidation and cell death. We found that the levels of plasma α-Tocopherol (αT), a type of Vitamin E that can be retained in animal bodies, were significantly higher in hibernating Syrian hamsters in the cold than the animals at non-hibernating state. To examine whether the rise in αT levels is due to acclimatization to the cold, we measured the amount of vitamin E in tissues after 2 weeks of cold exposure in Syrian hamsters and in non-hibernator, mouse. We found that the hamsters exposed to winter-like condition elevated αT in several tissues but the mice did not, indicating that there is a difference in distribution of Vitamin E in response to the cold between Syrian hamster and mouse.

(COI:No)

### PP-391

### Effects of intravenous anesthesia on behavioral circadian rhythms and clock gene expression in the suprachiasmatic nucleus in rats

Tomoki Mizuno[1,2], Shinpei Higo[2], Hitoshi Ozawa[2] (*[1]Dept. Anesthesiology and pain medicine ., Grad. Sch. Med., Nippon Med. Sch., Tokyo, Japan, [2]Dept. Anat. Neurobiol., Grad. Sch. Med., Nippon Med. Sch., Tokyo, Japan*)

The suprachiasmatic nucleus (SCN) is the principal clock of the brain, directing circadian rhythms by regulating the expression of clock genes. Postoperative adverse events related to circadian rhythms, such as insomnia, are partly due to anesthesia-induced disruption of clock gene expression. We examined the effects of two anesthetics on the behavioral circadian rhythms and on the expression cycle of the clock gene *Per2* in the SCN using rats.
Rats were treated with sevoflurane or dexmedetomidine, and their behavioral rhythms were compared with those of the control groups. We also performed *in situ* hybridization to analyze the expression cycle of *Per2* in the SCN after anesthesia.
No apparent phase shift in the behavioral rhythm was observed in all three anesthetic groups. *Per2* expression in SCN showed a transient significant decrease immediately after anesthesia in all groups, but did not cause a shift in the *Per2* expression cycle
The suppression of *Per2* expression in SCN may be common to general anesthetics that cause loss of consciousness. These results should be translated into efforts to establish safer methods of anesthetic management.

(COI:No)

### PP-392

### Inhalation anesthetic sevoflurane induces phase-shift of circadian clocks in the brain specifically at night

Takeshi Yamaguchi[1], Toshiyuki Hamada[2], Norio Iijima[1] (*[1]Center for Basic Medical Research, International University of Health and Welfare, [2]Department of Pharmacology, International University of Health and Welfare*)

The suprachiasmatic nucleus (SCN), choroid plexus in lateral ventricle (CP-LV) and choroid plexus in forth ventricle (CP-4V) are the robust circadian oscillators within the brain. The three types of tissues are considered to be interacting each other in maintenance of stable rhythm *in vivo*.
We previously reported inhalation anesthetic sevoflurane affect *Per2* expression in SCN, CP-LV and CP-4V *in vitro* and *in vivo*. However, little known about the regulation system of circadian activity in whole brain level by sevoflurane although inhalation anesthetics have no effect on locomotor activity rhythm. In this study, the effect of sevoflurane on circadian activity in each 3 tissue were investigated.
SCN, CP-LV and CP-4V explants which were prepared from the Per2::Luc transgenic rats that were anesthetized late at night showed significant phase-delay in comparison to the non-anesthetized rats.
It indicates that shifted *Per2* expression rhythm of the 3 explants was not corrected by themselves. Examining the 3 explants of the rats on the 7th day after anesthetic treatment, only the SCN explants showed the phase-delay, which suggests that the effect of the anesthetic remains in the *in vivo*.

(COI:No)

### PP-393

### Melinjo (*Gnetum gnemon L.*) seed extract rescues fragmented non-rapid eye movement sleep in mice with diet-induced obesity

Akira Terao[1], Mao Sato[1], Chiaki Sugiura[1] (*[1]School of Biological Sciences, Tokai University*)

We investigated the effect of dietary melinjo (*Gnetum gnemon L.*) seed extract (MSE) on sleep architecture in high-fat diet (HFD)-induced obese mice. Forty C57BL/6J male mice were fed different diets for 17 weeks: normal diet (ND), ND with 1% MSE (ND+MSE), HFD, and HFD with 1% MSE (HFD+MSE). Body weight and sleep architecture were examined in all mice after the study period. The body weight of HFD-fed mice increased by 50% compared to that of ND-fed mice. Although HFD did not affect the amount of non-REM (NREM) sleep, the average duration of NREM sleep bout was significantly shortened, and the number of NREM sleep bout was significantly increased. These findings indicate fragmented NREM sleep and altered sleep architecture in HFD-fed mice. Dietary MSE did not affect body weight or sleep architecture in the ND+MSE-fed mice. In contrast, the body weight and sleep architecture of HFD+MSE-fed mice were almost identical to those of ND-fed mice, indicating that dietary MSE completely blocked HFD-induced weight gain and sleep fragmentation. Our data provide compelling evidence that MSE is a novel and promising dietary supplement that restores obesity-induced impaired sleep architecture.

(COI:Properly Declared)

### PP-394

### New methodology to evaluate itch levels in mice

Kotaro Honda[1], Mitsutoshi Tominaga[1], Kenji Takamori[1,2] ([1]*Institute for Environmental and Gender Specific Medicine, Juntendo University Graduate School of Medicine,* [2]*Department of Dermatology, Juntendo University Urayasu Hospital*)

Itch is defined as an unpleasant sensation that causes scratching behaviors. In normal skin, itch sensation that occurs once is diminished by subsequent scratching behaviors. On the other hand, itch in pathological conditions becomes more itch by scratching behaviors, and this is called the Itch-scratch cycle. The vicious cycle of Itch is regarded as a cause to delay the healing of dermatitis, but no studies have been conducted on the itch-scratch cycle itself.
In this study, we analyzed the scratching behavior of asymptomatic mice to clarify the baseline of itch before developing the vicious itch-scratch cycle. As a result, we found a mouse that showed certain characteristic behaviors, and hypothesized that they were indexes of the vicious itch-scratch cycle and verified it. Moreover, the indexes was applied to dry skin model mice induced by mixture of aceton/diethylether and water treatments. We are going to introduce the new methodology to evaluate the itch-scratch cycle in mice and recent progress. (COI:No)

### PP-395

### Alterations of cholecystokinin and parvalbumin expressions in the limbic system and increased anxiety-like behavior in OLETF rats of different ages

Ryosuke Ochi[1], Naoto Fujita[1], Natsuki Goto[1], Kaho Takaishi[1], Takaya Oshima[1], Tien Son Nguyen[1], Hisao Nishijo[2], Susumu Urakawa[1] ([1]*Dept Musculoskeletal Functional Research and Regeneration, Hiroshima Univ, Hiroshima, Japan,* [2]*Dept System Emotional Science, Univ Toyama, Toyama, Japan*)

We have reported that 20-week-old Otsuka Long-Evans Tokushima fatty (OLETF) rats exhibit increased anxiety-like behavior and cholecystokinin (CCK) positive neurons in the limbic system. However, those alterations of OLETF rats in different ages remain unclear. We investigated anxiety-like behavior and CCK and parvalbumin (PV) expressions of 8- and 30-week-old OLETF rats. In the open field test, OLETF rats exhibited lower locomotion in the center zone and longer latency to leave the center zone at 8 and 30 weeks of age, respectively. The densities of CCK positive neurons of OLETF rats were higher in the lateral and basolateral amygdala at only 8 weeks of age, and in the anterior cingulate, infralimbic cortices and hippocampal CA3 at both ages. Those of OLETF rats in the amygdala and infralimbic cortex were decreased with age. The densities of PV positive neurons of OLETF rats were lower in the prelimbic and infralimbic cortices at both ages, and in the hippocampal CA2 at only 8 weeks of age. These results suggest that OLETF rats exhibit anxiety phenotypes from early adulthood and distinct alterations of CCK and PV expressions in the limbic system at 8 and 30 weeks of age. (COI:No)

### PP-396

### Sleep architecture analysis of secretin receptor knockout mouse

Aiko Moridera[1], Hiroaki Fujihara[1], Nobuhiro Fujiki[1] ([1]*Dept of Ergonomics, Institute of Industrial Ecological Science, UOEH, Japan*)

Secretin is a peptide hormone secreted by the duodenum, but also functions as a neuropeptide. It has recently been reported that secretin may be involved in the pathogenesis of autism. As sleep problem have been reported as a symptom of autism, we hypothesized that secretin peptide might be involved in sleep control. To examine this, we analyzed the sleep profile of secretin receptor knockout mice (SRKO). Under anesthesia, electrodes for EEG and EMG, and radio transmitters for measuring core body temperature and activity were installed in both SRKO and wild type mice (WT). After recovery, the baseline sleep recording and four hours of sleep deprivation (SD) were performed. The baseline sleep architecture and responses to SD were compared between SRKO and WT. Although there were no significant differences in basic sleep architecture or in immediate response to SD, SRKO showed a significant fragmentation of arousal and NREM sleep in the first dark period after SD compared to WT. These results suggest that secretin system is not necessary for normal sleep control, but it may be involved in the mechanism of maintenance of sleep and wakefulness after sleep deprivation. (COI:No)

### PP-397

### Circadian regulation of wakefulness is mediated by CRF neurons in the paraventricular nucleus of the hypothalamus

Daisuke Ono[1], Yasutaka Mukai[1], Chi Jung Hung[1], Srikanta Chowdhury[1], Takashi Sugiyama[2], Akihiro Yamanaka[1] ([1]*Research Institute of Environmental Medicine, Nagoya University, Japan,* [2]*Olympus Corporation*)

Living organisms exhibit endogenous circadian rhythms and adapt to the 24-h daily cycles of the Earth. Circadian rhythms are known to organize the temporal timing of physiology and behavior. In mammals, the suprachiasmatic nucleus (SCN) of the hypothalamus functions as the master circadian pacemaker. However, little is known about neuronal projections from the SCN which regulate sleep/wakefulness. Taking advantage of optogenetics and optical imaging, we identified that activation of corticotropin-releasing factor (CRF) neurons in the paraventricular nucleus (PVN) of the hypothalamus increased time in wakefulness in mice. This wake promoting effect was due to further activation of orexin neurons which are important of maintaining of wakefulness. Furthermore, in vivo $Ca^{2+}$ recording revealed that CRF neurons in the PVN were active during wakefulness. On the other hand, when we suppressed or ablated CRF neurons, time in wakefulness or locomotor activity were reduced. We also found that neuronal activity of CRF neurons in the PVN was regulated by GABAergic neurons located in the SCN. Our findings provide significant insights into circadian regulation of sleep/wakefulness in mammals. (COI:No)

### PP-398

### Neonatal dopamine depletion results in a decrease in a spontaneous locomotor activity in a familiar environments in adulthood.

Masanori Ogata[1], Saki Nishikawa[1], Emiri Tago[1], Ikumi Abe[1], Hitoshi Ishibashi[1] ([1]*Dept Physiol, Sch Allied Health Sic, Kitasato Univ*)

The dopaminergic neural system play a crucial role in motor regulation as well as regulation of anxiety-related behaviors. Although rats with neonatal dopamine depletion show motor hyperactivity in unfamiliar environments, characterization of their behavior in familiar environments is unknown. In the present study, we examined the effects of neonatal dopamine depletion on spontaneous locomotor activity in familiar (24-hour home cage test) and unfamiliar (open field test) environments in adult rats. Rats that received intraventricular injection of 6-hydroxydopamine (6-OHDA) 4 days of age showed increased distance traveled and percentage of time spent in the center area of the open field, and decreased exploratory behavior (rearing) in open field test. On the other hand, the 6-OHDA-treated rats showed decreased locomotor activities in both light and dark periods in the 24-hour home cage test. There is no significant correlation between the spontaneous locomotor activities in these behavioral tests in the 6-OHDA-treated rats. There data suggested that neonatal dopamine depletion results in abnormal behaviors of which pattern depend on a stress intensity from the environments. (COI:No)

### PP-399

### A study on sleep quality and autonomic nervous response before and after sleep onset in working menopausal women

Michiko Tanaka[1], Chiyomi Egami[2], Miyuki Matsukawa[2], Aki Nozue[3], Tomoko Tsuda[1], Misao Arimatsu[4], Mou Nagasawa[1] ([1]*School of Nursing, Miyazaki Prefectural Nursing University,* [2]*Fukuoka Prefectural University,* [3]*Miyazaki University,* [4]*Kagoshima Immaculate Heart University*)

In this study, we investigated the sleep parameters in daily life of healthy menopausal women (n=13, 49.8±3.2 years old) through home monitoring. We compared the sleep parameters (Nemuri Scan mat), perceived sleep quality using VAS or OSA sleep inventory MA version and autonomic nervous response using heart rate variability (myBeat) in working day (WD) and holiday (HD). The total sleep time and the time in bed during WD were significantly shorter than those during HD. However perceived sleep quality using VAS or OSA was not significant different between WD and HD. The RR interval before sleep onset of HD was significantly higher than that of WD. The time course of RR interval after sleep onset in both WD and HD initially increases and then gradually decreases. The correlation between sleep quality and the total sleep time revealed the statistically positive significance in HD ($r = 0.302$, $p < .1$), but not in WD ($r = -0.160$, $p > .1$). Thus, it is suggested that the responsiveness of the sleep evaluation to the total sleep time is different between WD and HD in menopausal women. Hence, different interventions are necessary to improve the sleep both in WD and HD. (COI:No)

### PP-400

### A behavioral assay system to analyze tone discrimination and auditory preference in rats

Kosuke Matsuzaki[1], Masayuki Okubo[1], Yutaka Komura[2], Riichi Kajiwara[1] (*[1]Meiji University, [2]Kyoto University*)

To investigate rats' preference behavior for complex auditory stimuli like music, we developed an experimental system using a U-shaped maze presenting different sounds at both ends. We used six sound sources, consisting of five that differ only in tone (6 seconds of music with the same melody) plus white noise. In the auditory discrimination task, we divided the six sound sources into the rewarded and non-rewarded group. We confirmed that the rats could discriminate differences in tone with 65 - 91% accuracy (n=12).
In the preference behavior test, we presented rats with piano, bell, and violin tones as novel sounds. The results showed that 7 out of 8 rats tended to stay around the piano more often than the violin. Even after learning to associate these sounds with a drinking reward, the violin tone showed relatively low preference. In the experiment in which white noise conditioned as a rewarded sound was pair presented to these three sounds conditioned as the non-rewarded group, rats rarely preferred noise. Under the present experimental condition, the effect of auditory reinforcer should be more robust than that of edible reinforcer in the avoidance behavior to white noise (COI:No)

### PP-401

### Relationship between coping styles and saliva components in the first-year university students.

Mitsuo Nagane[1], Yoshinori Oyama[2], Hiromasa Sato[1], Shuichi Watanabe[1], Naofumi Miwa[1] (*[1]Dept.Physiol., Saitama Med. Univ., [2]Dept.Educ. Psychol., Chiba Univ*)

Students' mental health often depends upon how they cope with stress at university life, and therefore, it is of great significance to identify molecular clues that reflect the status of their coping adaptation. The first-year students encounter substantial stress at entrance into the university in April. In this study, the first-year students voluntarily submitted the questionnaire that addressed several coping styles, including problem focused, emotion focused and escape focused ones; on that occasion, we simultaneously collected saliva samples from the students across four months (April - July), and examined the relationship between the coping styles and the concentrations of saliva components. We found that the averaged cortisol concentration in saliva of those who obtained higher Lickert scores in problem-focused style was significantly lower, compared with those who did lower scores. The difference in the averaged cortisol concentrations between two groups became greater with the lapse of the time. These results suggest that salivary cortisol reflects the stress-coping status, especially for problem-focused style and may help to find warning signs of students. (COI:No)

### PP-402

### Antidepressant-like effect of Kamikihito against chronic restraint stress-induced depressive-like behaviors and impaired hippocampus neurogenesis in rats

Naoki Adachi[1], Fatma Zahra Sakhri[1,2], Yusuke Ohashi[1], Tsukada Mana[1], Mami Kato[1], Hideshi Ikemoto[1], Masataka Sunagawa[1] (*[1]Dept Physiol, Sch Med, Showa Univ, Tokyo, Japan, [2]Dept Biol Animal, Univ Frères Mentouri-Constantine1, Algeria*)

Substantial evidence suggests the effectiveness of plant-based medicine in stress-related diseases. Kamikihito (KKT), a Kampo medicine, has been used for anemia, insomnia, and anxiety. Recent studies revealed its ameliorating effect in cognitive and memory dysfunction in several animal models.
KKT was orally administered to rats daily at 300 or 1,000 mg/kg during 21 consecutive days of chronic restraint stress (CRS) (6 h/day). The effect of KKT against the stress-induced changes in anxiety- and depressive-like behaviors and hippocampal neurogenesis were determined.
CRS for 21 days caused a significant decrease in body weight gain and increase in plasma corticosterone levels and percentage of adrenal gland weight to body weight, which were ameliorated by KKT treatment. KKT also rescued the CRS-induced anxiety- and depressive-like behaviors assessed in the open field test, sucrose preference test, and forced swimming test. Impairment of hippocampal neurogenesis caused by CRS also rescued by KKT administration.
These data suggest that daily supplementation of KKT has a protective effect against physiological, neurological, and behavioral changes in a rat model of depression. (COI:No)

### PP-403

### The characteristics of $Ca^{2+}$ release via ryanodine receptors are altered in the hippocampus of depression-like model mice.

Emi Nakamura-Maruyama[1], Naoyuki Himi[1], Kazuhiko Narita[1], Risa Kai[1], Osamu Miyamoto[1] (*[1]Dept Physiol 2, Kawasaki Med Sch*)

Although depressive disorders are common diseases, their pathogenic mechanisms are not fully understood. In this study, we measured $Ca^{2+}$ release via ryanodine receptors (RyRs) to investigate the functional changes of RyRs under depressive condition.
We used the depression-like model mice which were suffered water immersion with restraint stress. Fluorescence intensity changes of pyramidal cells in the dentate gyrus of hippocampal slice were measured by using $Ca^{2+}$ ion indicators. $Ca^{2+}$ release via RyRs was induced by flushing caffeine solution into the recording chamber. We compared it with the changes in $Ca^{2+}$ release due to electroconvulsive shock (ECS) with antidepressant effect.
The results showed that the number of cells which showed caffeine-induced $Ca^{2+}$ release decreased in the hippocampal dentate gyrus of the model mice, and the frequency of caffeine-induced oscillation also decreased. In addition, the rise time of caffeine-induced $Ca^{2+}$ release was prolonged. All of these changes were improved by ECS.
It is suggested that the degree of the depressive condition depends on the degree of Ca2+ release ability via RyRs. (COI:No)

### PP-404

### Relationship among the inferior fascicle of the anterior talofibular, calcaneofibular ligaments, and joint capsule in the lateral ankle ligament complex

Akira Kakegawa[1,2], Norimi Sumitomo[2], Ayata Nagira[2], Yuko Ichinose[2], Nanae Fukushima[2] (*[1]Faculty of Health Care, Teikyo Heisei University, [2]Department of Anatomy, Shinshu University School of Medicine*)

The inferior fascicle of the ATFL and the CFL forms the lateral fibulotalocalcaneal ligament (LFTCL) complex, which is thought to be the extracapsular ligaments. This study aims to clarify the detailed structure of the LFTCL complex. A total of 54 formalin-fixed ankles were amputated 10 cm above the inferior tip of the lateral malleolus. The soft tissues of the lateral ankle ligament complex were resected and carefully dissected from outside. We examined the LFTCL complex, and evaluated the connection between the inferior fascicle of ATFL and the anterior part of CFL. The lateral ankle ligament complex at the lower end of the fibula was harvested, including the fibula and talus bone. The specimen was decalcified with K-CX and was sliced into 10 μm thickness sections along the ATFL axis and then stained with H&E. The LFTCL complex was composed of two layers: the surface layer of LFTCL, which consisted of the inferior fascicle of the ATFL and the anterior part of the CFL, and the deep layers of the LFTCL, wherein the inferior fascicle of the ATFL was integrated with the capsule to form the lower joint capsule and the CFL was attached to the fibula from outside the capsule. (COI:No)

### PP-405

### Lymphatic drainage pathway from epineurium around trunk of spinal nerve in human

Shinichi Kawata[1], Takuya Omotehara[1], Michiko Naito[2], Kazuyuki Shimada[1], Masahiro Itoh[1] (*[1]Department of Anatomy, Tokyo Medical University, [2]Division of Anatomical Science, Department of Functional Morphology, Nihon University School of Medicine*)

In recent years, the presence of lymphatic vessels in the meninges was reported as a route for spinal fluid drainage in mice. The objective of this study is to clarify the connection of lymphatic vessels from the spinal meninges to the venous angle in human.
The lymphatic vessels and lymph nodes around the neck region were dissected in cadavers embalmed with Saturated Salt Solution method in Tokyo Medical University. The spinal cord at the C2-4 level including the spinal nerves was collected from formalin-fixed cadavers for histological analysis. The marker protein for lymphatic vessels was detected by immunohistochemistry.
The lymphatics around the internal jugular vein, which was joined to the venous angle, was connected to the epineurium around the trunk of the spinal nerve. Indeed, immunopositive reactions for the lymphatics marker protein were detected on the vessels in the epineurium around the trunk of the spinal nerve.
The present study suggests that fluid from the central nervous system is drained into the epineurium lymphatics and flows into the venous angle through the lymphatic vessels along the internal jugular vein in human. (COI:No)

### PP-406

### Anatomical evaluation of the lateral pterygoid insertion on the medial surface of the condylar process

Masahiro Tsutsumi[1], Sasin Sritara[2], Keiko Fukino[2], Keiichi Akita[1] ([1]*Department of Clinical Anatomy, Tokyo Medical and Dental University*, [2]*Department of Orthodontic Science, okyo Medical and Dental University*)

Lateral pterygoid (LP) is vital in coordinating jaw movements. Recently, we reported that the insertion of the LP is not limited on the pterygoid fovea but also located on the medial surface of the condylar process (mCP). Morphological features of this insertion may contribute to provide the precise understanding how the LP coordinate jaw movements. This study aimed to investigate the morphological features of the LP insertion on the mCP. We analyzed 10 jaws from seven Japanese cadavers. Insertion area on the mCP was measured by using microcomputed tomography and it occupied 28.8±5.0% of the entire insertion areas. In addition, muscle bundles inserting into the mCP originated from the posterior portion of the lateral plate of the pterygoid process. Therefore, muscle bundle of the LP inserting on the mCP had a broad insertion area and a specific origin which was distinguishable from the remaining origin. Although this muscle bundle was not the independent part of the LP, it can act as one of the functional subunits. Especially, its different insertion dimension from the pterygoid fovea on the horizontal plane suggested that it contributed to coordinate the lateral jaw movement. (COI:No)

### PP-407

### Ramification pattern of the middle colic artery in the rat

Tetsuhito Kigata[1], Yasushi Kobayashi[1] ([1]*Dept. Anat. & Neurobiol., Natl. Def. Med. Col*)

Intestinal surgery is commonly performed in rats for the purpose of the experiment or surgical training, and such surgery should be conducted based on the detailed arterial anatomy including the species differences. The human middle colic artery arises from the cranial mesenteric artery and bifurcates into the right and left branches to supply the transverse colon. Although rats have usually two middle colic arteries, their distributions have not been sufficiently studied compared with that of humans. Hence, in this study, we traced the middle colic artery in 30 Wistar rats (15 males and 15 females). One (12 cases, 40%), two (16 cases, 53%) or three (2 cases, 7%) middle colic arteries arose from the cranial mesenteric artery. In 14 cases (47%) with two middle colic arteries, the proximal one supplied the distal transverse colon and anastomosed with the left colic artery, and the distal one distributed to the proximal transverse colon and anastomosed with the right colic artery. Moreover, these two middle colic arteries anastomosed each other in 12 (40%) out of 14 cases, while in the remaining two cases (7%), the anastomosis did not exist. (COI:No)

### PP-408

### Running courses of cutaneous nerves on the radial and dorsal surface of the forearm in pronated position.

Masatoshi Komiyama[1], Momoka Okabe[2], Tatsuya Jitsuishi[3] ([1]*Grad. Sch. Nurs., Chiba Univ.*, [2]*Facult. Nurs., Chiba Univ.*, [3]*Grad. Sch. Med., Chiba Univ*)

To avoid nerve injury on the venipuncture for an intravenous drip infusion, running courses of cutaneous nerves on the radial and dorsal surface of the forearm in pronated position were analyzed. Data were collected from six arms of three donated cadavers. Running courses were expressed based on the longitudinal axis (LA) which was set between the lateral epicondyle of humerus and the radial styloid process (basic point was set at the epicondyle). The superficial branch of radial nerve (SRN) emerged at the distance of 61.2-77.5% of LA and ran along palmar side (< 10 mm) of LA toward the thumb. The lateral cutaneous nerve of forearm (LCN) appeared just lateral to the biceps brachii tendon in the cubital fossa and ran toward the radial styloid process. On the way of LCN a few branches ramified and ran toward the dorsoradial surface of the forearm beyond LA at various height. The cephalic vein ran initially along SRN and then along LCN and/or its branches. The posterior cutaneous nerve of forearm (PCN) ran along palmar side of LA and ramified to be distributed to the dorsal surface of the forearm. These courses of SRN, LCN and PCN should be concerned on the venipuncture. (COI:No)

### PP-409

### Innervation of the human sternoclavicular joint

Kenji Emura[1], Takamitsu Arakawa[2] ([1]*Himeji Dokkyo Univ. Faculty of Health Care Sci.*, [2]*Kobe Univ. Grad School of Health Sci*)

The proprioception of the sternoclavicular joint (SCJ) seems important to control the movement of the pectoral girdle. According to our previous study (Emura et al., 2019), we focused on the most medial branch of the medial supraclavicular nerve (MSN) and branches from the lateral pectoral nerve (LPN) as possible nerve branches innervating the SCJ. In this study, four sides of these nerve branches and the SCJs were observed under stereomicroscope, after maceration in trypsin solution to facilitate removing soft tissue and pursuing fine nerve branches to the SCJ. All cadavers were provided for the dissection course in Kobe University faculty of medicine. In all four sides, the most medial branch of the MSN reached the anterior and/or superior part of the articular capsule of the SCJ. In two sides, nerve branches from the LPN reached the inferior part of the articular capsule of the SCJ. In other two sides, similar branch from the LPN did not innervate the SCJ but ended at the periosteum of manubrium and the perichondrium of the first costal cartilage. These findings provide the anatomical basis for discussing motor control of the pectoral girdle. (COI:No)

### PP-410

### Branching pattern of nerve fascicles and intramuscular distribution pattern of the soleus muscle in the gibbon.

Tohma Sakuraya[1], Kenji Emura[2], Eishi Hirasaki[3], Takamitsu Arakawa[1] ([1]*Grad. Health Sci., Kobe Univ.*, [2]*Fac. Health Care Sci., Himeji Dokkyo Univ.*, [3]*PRI, Kyoto Univ*)

The human soleus muscle is innervated by anterior and posterior branches from the tibial nerve. In a previous study examining branching patterns of nerve fascicles in humans, anterior branches ramified from the muscular branches to deep flexor group and posterior branches separated from them to gastrocnemius, suggesting that the soleus muscle may have different origins (Okamoto et al., 2013). In the gibbon, we encountered anterior and posterior branches to the soleus, then branching pattern of nerve fascicles in the tibial nerve and intramuscular distribution pattern of the soleus were examined. Two sides of soleus in one cadaver were used. In one case, anterior branch bifurcated from the branch to flexor digitorum fibularis and two posterior branches separated from the branch to gastrocnemius. Within the soleus, the anterior branches communicated with the posterior branch in three points. The other case of the soleus had no anterior branch. Distribution pattern for the posterior branch of the soleus in the gibbon was similar to the human (Sekiya, 1991) but independent distribution area of the anterior branch was narrow and not similar to human. (COI:No)

### PP-411

### Three dimensional positional arrangement of the intestinal tract in the fetal pigs

Ryuhei Kojima[1], Nanako Fuchigami[1], Konosuke Tokita[1] ([1]*Faculty Health Medical Care, Saitama Med. Univ*)

Three dimensional positional arrangement of the intestinal tract is complex. In the pigs the positional arrangement differs from that of the humans in the midgut portion. The differences have not been noticed except a characteristic coiled ascending colon. We described the three dimensional positional arrangement of the intestinal tract with the peritoneum and mesentery in fetal pig specimens just before birth and compared it with that of humans and macaques. In the pigs the duodenum crossed the colon behind it after turning left at the caudal flexure of the duodenum similarly in the humans. The ascending colon crossed the root of the mesentery behind it after recoiling from the centrifugal turns of the conical ascending colon. This crossing did not exist in the humans. In the macaques the positional arrangement of the intestinal tract was similar to that of the humans except having the mesocolon along all the length. The three dimensional arrangement of the intestinal tract is formed during the period of the elongation, physiological umbilical herniation and return from it at the early developmental stages. The differences observed here may occur in this period. (COI:No)

### PP-412

### An anatomical reinvestigation of the palmar arches

Jun Kanazawa[1], Shoichiro Yamashita[2], Jiro Hitomi[1] ([1]*Division of Human Embryology, Department of Anatomy, Iwate Medical University,* [2]*Medical Student, Faculty of Medicine, Iwate Medical University*)

Understanding the morphology of the palmar arches, which may exhibit wide variation, is important for vascular reconstruction. We therefore dissected 40 hands of 20 cadavers and carried out an anatomical reinvestigation of the overall forms of the superficial palmar arch (SPA) and deep palmar arch (DPA). The SPA was divided into four types (Types A-D), while the DPA was divided into two (Types E-F). Most examined SPAs were Type A, i.e., the arterial arch was formed solely by the ulnar artery (UA). Type B was observed in fewer SPAs; these SPAs are didactically formed by the UA and the superficial palmar branch of the radial artery (RA). The involvement of the arterial arch in the RA was limited, and only a few typical cases were observed. On the other hand, while the types of the DPA divided depending on the variant of the deep branch (DB) of the UA, the RA was always involved in the arch. The palmar arches predominantly involved Type A and Type E. Type E is formed by the proximal DB of the UA and the RA, which enters the 1st dorsal interosseus. From these results, we discussed the development of the RA, UA, and medial artery, which are all involved in the formation of these arches. (COI:No)

### PP-413

### Double sided superior vena cava -Consideration of the relationship with the development of the thymic vein-

Keiko Takamura[1,2], Kazunobu Saiki[1], Daisuke Endo[1], Kiyohito Murai[1], Keishi Okamoto[1], Toshiyuki Tsurumoto[1,2] ([1]*Department of Macroscopic Anatomy, Graduate School of Biomedical Science, Nagasaki University,* [2]*Center of Cadaver Surgical Training, Nagasaki University School of Medicine*)

The superior vena cava (SVC) is usually present only on the right side of the body. While performing routine dissection of a 91-year-old Japanese female cadaver, a victim of sepsis, we recognized the double-sided SVC. The left subclavian vein, the left internal jugular vein, and the anterior jugular vein joined to form the left SVC, and it opened into the coronary sinus. On the right side, the right subclavian vein and the right internal jugular vein joined to form the right SVC, and it opened into the right atrium. A single anastomosing branch was present between the bilateral SVCs. The first to 11th right intercostal veins, the right subcostal vein, and the left ninth intercostal vein flowed into the azygos vein. The azygos vein opened into the right SVC. The left tenth and 11th intercostal and subcostal veins flowed into the hemiazygos vein, and it opened into the azygos vein. The left second to eighth intercostal veins flowed into the accessory hemiazygos vein. The accessory hemiazygos vein opened cranially into the left SVC and opened caudally into the azygos vein. We present consideration of SVC formation as it relates to the development of the thymic vein. (COI:No)

### PP-414

### A case of the sural nerve communicating with the posterior femoral cutaneous nerve

Kanae Umemoto[1], Daisuke Kiyoshima[1], Shogo Hayashi[1], Ning Qu[1], Kaori Suyama[1], Kou Sakabe[1] ([1]*Department of Anatomy, Division of Basic Medical Science, Tokai University School of Medicine* )

The sural nerve (SN) is a cutaneous nerve, usually formed by the union of the medial sural cutaneous nerve (MSCN) and the lateral sural cutaneous nerve (LSCN), which originate from the tibial nerve (TN) and common fibular nerve (CFN), respectively. During the dissection of a 90 years old male Japanese cadaver, we found the right SN which consisted of the confluence of the MSCN and peroneal communicating branch (PCB) which originated from LSCN. The SN was descended in company with the lesser saphenous vein on the posterior side of lower limbs. The posterior femoral cutaneous nerve (PFCN) joined at 40mm distal to the MSCN/PCB connection to form the SN. The TN, CFN and PFCN was derived from the ventral divisions (VDs) of the L4-S4, the dorsal divisions (DDs) of the L4-S3, and the VDs and the DDs of S3-S4, respectively. Although the variations of the SN have been reported several times, the SN including the PFCN has been seldom reported in adult cadavers. To our knowledge, our case is the first report of the SN including the PFCN in which the nerve roots are identified. Our finding may be important for diagnosis and treatment of cutaneous sensory disorder of lower thigh. (COI:No)

### PP-415

### Effect of sacroiliac joint morphology on the biomechanics of the pelvis during single-leg stance: a finite element analysis with elderly women

Keita Nishi[1], Toshiyuki Tsurumoto[2], Daisuke Endo[2], Kazunobu Saiki[2], Joichi Oyamada[1], Yoshitaka Mnabe[1] ([1]*Dept of Oral Anatomy and Dental Anthropology, Nagasaki Univ, Nagasaki,Japan,* [2]*Dept of Macro Anatomy, Nagasaki Univ, Nagasaki, Japan*)

Recent studies on the biomechanics of the human pelvis have predominately using simulation analysis methods with finite element (FE) models based on morphological data collected from computed tomography (CT) or magnetic resonance imaging. Although sacroiliac joint (SIJ) showed various morphology, pelvic FE studies conducted thus far have not examined how this variation affect pelvic biomechanics. Therefore, this study aimed to clarify the biomechanical differences caused by SIJ morphology with FE methods.
Among the cadavers donated for the anatomical practice at the Nagasaki University, the pelvis of the 4 women in their 70s who gave consent when they were alive were included in the study. The FE model of the pelvis was created using image data obtained from CT performed shortly after death. The distribution of stresses applied to the SIJ were visually compared between cadavers under the condition assuming a single-legged standing position. The results revealed that the location of strong stress on the articular surface differed depending on the morphologies of the posterior border of the SIJ. Hence, this study suggests that SIJ morphology influences biomechanics of the pelvis. (COI:No)

### PP-416

### The lumbosacral plexus organization associated with changes of thoracolumbar vertebrae in fetal pigs

Masaki Sakamoto[1], kounosuke Tokita[2], Ryuhei Kojima[2] ([1]*Area of physical therapy, Specialism of medical science, Graduate School of Saitama Medical University,* [2]*Department of Physical Therapy, Faculty of health and Medical Care, Saitama Medical University*)

The pigs have variations in the number of thoracolumbar vertebrae. We investigated how changes in the number of thoracolumbar vertebrae affect the lumbosacral plexus in the fatal pigs. We observed on the nerve roots, course, and distribution of ramus cutaneus lateralis (Rcl), nerves innervating m. rectus abdominis (Rra), n. cutaneus femoris lateralis (CFL), n. obturatorius (O), n. femoralis (F), truncus lumbosacralis (Tr), n. ischadicus (I) and n. pudendus. The number of thoracolumbar vertebrae were 20 (Th14 + L6), 21 (Th14+L7, Th15+L6, Th16+L5) and 22 (Th16+L6). As the number of thoracolumbar vertebrae increased, the nerve roots of the nerves distributed to the muscles of the trunk and lower limbs shifted caudally. Counting the lumbosacral plexus constituents from the first sacral nerve (S1), the lower limit of the Tr roots was located the 1 cranial from S1. The lower limit of the O and F roots was located on the 1 to 2 cranial from S1. The lower limit of the I roots was located on the 0 to 1 caudal from S1. Thus, it was suggested that the position of sacral vertebrae is related to the morphogenesis of nerves distributed in the lower limb muscles. (COI:No)

### PP-417

### Angiotensin converting enzyme 2 expression in experimental animals and human cadavers

Naho Suzuki[1], Miki Nagase[1] ([1]*Dept. Anatomy, Sch. Med., Kyorin Univ*)

Angiotensin converting enzyme (ACE) 2 (ACE2), a homologue of ACE, converts angiotensin II to angiotensin 1-7, which exerts protective effects on the cardiovascular system via MAS receptor. Recent studies highlighted the role of ACE2 as a receptor for SARS-CoV-2/2019-nCoV. The aim of the present study was to examine the expression and localization of ACE2 in mice and human cadavers. RNA was extracted from various tissues of normal mice. Gene expression was analyzed by quantitative PCR. ACE2 expression was high in the intestine, followed by the kidney, aorta, lung, brain, and heart. Next, immunohistochemistry was performed in several tissues obtained from formalin-fixed human cadavers. Strong ACE2 expression was observed in the mucosal cells of the jejunum. In the kidney, distinct signals were detected in the tubular cells. Double immunostaining revealed tubular segments in which ACE2 was expressed. In conclusion, we demonstrated ACE2 expression in several tissues of human cadavers and normal mice, suggesting the role of ACE2 in COVID-19 infection or cardiovascular protection in these tissues. (COI:No)

### PP-418

### Inferior vena cava duplication with left-side predominance

Takao Mukuda[1], Sawako Hamasaki[1], Yuka Koyama[1], Hironobu Nakane[1], Kenji Okazaki[1], Yuki Ohneda[2], Rei Terawaki[2], Yuuka Kubo[2], Wataru Kurimasa[2], Shunya Kuroda[2], Naoki Kohno[2], Toshiyuki Kaidoh[1] ([1]*Department of Anatomy, Faculty of Medicine, Tottori University,* [2]*School of Medicine, Tottori University*)

Duplication of the inferior vena cava (IVC) was found in a cadaver (87-year-old Japanese male) during a dissection course at Tottori University in 2020. The duplicated IVC was predominant on the left side. At the confluence, the width of the left IVC was 35 mm, while that of the right IVC was 13 mm. The thick left IVC ascended along the left side of the abdominal aorta and crossed over it at the level of the L2 vertebra, forming the preaortic trunk which continued to the IVC. On the right side, the right common iliac artery was divided into the right IVC and interiliac vein behind the right common iliac artery. Then the thin right IVC ascended along the right side of the abdominal aorta and connected to the left IVC at the level of the L1 vertebra, while the interiliac vein ascended obliquely and connected to the left IVC at the level of the L4 vertebra. The left IVC received the ipsilateral common iliac, testicular, renal, and suprarenal veins. The right testicular and renal veins join the IVC proximal to the confluence of the left and right IVCs. According to the classification of Takemoto (1978), this case can be classified as type IIc, a rare case of IVC duplication. (COI:No)

### PP-419

### Examination of zygomatic bone structure and distribution of the zygomatic foramen related to zygomatic nerve, using micro-CT

Kouhei Kawata[1], Yoshiaki Ide[1], Kingo Suzuki[1], Masataka Sunohara[1] ([1]*Dpt. Anat. Nippon Dent. Univ. Sch. of Life Dent. at Tokyo*)

Purpose: The zygomatic nerve can be easily injured during facial surgery in the periorbital region. Hence, care should be taken to prevent injury to the nerve. The purpose of the present study is to examine the anatomical structures of zygomatic bone, and distribution of the zygomatic foramen related to zygomatic nerve.

Materials and methods: Thirty zygomatic bones were imaged using Micro-CT device. The internal structures of the zygomatic bone was observed, and the number of foramina in the zygomatic bone was counted.

Results and discussion: The relative frequency of zygomatico-orbital foramen was as follows: none 0 %, 1 foramen 36.7 %, 2 foramina 36.7 %, 3 foramina 23.3 %, 4 foramina 3.3 %. The relative frequency of zygomaticofacial foramen was as follows: none 6.7 %, 1 foramen 56.7 %, 2 foramina 23.3 %, 3 foramina 6.7 %, 4 foramina 6.7 %. The relative frequency of zygomaticotemporal foramen was as follows: none 13.3 %, 1 foramen 30.0 %, 2 foramina 33.3 %, 3 foramina 16.7 %, 4 foramina 6.7 %. It is important to clear understanding of these variations, which must be taken into consideration, in order to prevent unnecessary damage to the nerves, which exit the respective foramina. (COI:No)

### PP-420

### Developmental sequence of cusp formation in the modern shrew's molar: an implication for the evolution of tribosphenic molar in Mesozoic mammals

Atsushi Yamanaka[1], Yasin Haier[1], Urara Taguchi[1], Eriko Kuramoto[1], Haruki Iwai[1], Tetsuya Goto[1] ([1]*Dep. Oral Anat., Grad. Sch. Med. & Dent., Kagoshima Univ*)

An extremely diversified array of molar morphologies observed in extant mammals is evolutionary derived from one original type of molar, what is called the tribosphenic molar, which appeared in the Mesozoic Era. Started with reptilian-typed conical unicuspid tooth, Mesozoic mammals had spent more than 100 million years to complete this type of multicuspid molar, where many cusps were regularly arranged for efficient occlusion. On the other hand, developmental studies on mammalian molars have revealed that morphogenesis of occlusal surface is regulated by the signaling centers, the enamel knots. For a short embryonic period of time, several enamel knots appear reiteratively at the sites of future cusps. The present study examines the developmental process of modern shrew's molar, which is the least modified from the tribosphenic molar, using serial section in situ hybridization for the enamel-knot marker genes. By comparing the developmental process of shrew's molar with the evolutionary process of molar in Mesozoic mammals, we consider how developmental regulation in numbers and spacing of cusps could have affected the evolutionary transition of mammalian multicuspid molar. (COI:No)

### PP-421

### Quantitative analysis of the third-to-sixth thoracic vertebrae in primate using three-dimensional geometric morphometrics

Yasuhiro Kikuchi[1], Naomichi Ogihara[2] ([1]*Saga Univ., Faculty of Medicine.,* [2]*Univ. Tokyo, Graduate School of Science*)

This study characterized and visualized the species' traits in the shape of the thoracic vertebrae. Sixty vertebral samples were scanned by computed tomography (CT) and seventy-nine landmarks on the 3D bone surface reconstructed by CT images in each skeleton were detected. Generalized Procrustes Analysis was used to obtain size and shape variables for statistical analysis. The calculation for the variance–covariance matrix of the Procrustes residuals clarified Principle components of shape variation among samples. The vertebrae of hominoids exhibit wide body, dorsally oriented transverse process and short spinous process. In contrast, non-hominoid primates have a narrow vertebral body, ventrally oriented transverse process and long spinous process. The vertebrae of terrestrial cercopithecoids show dorsally oriented spinous process, and dorsoventrally long and craniocaudally short shape. On the other hand, arboreal platyrrhines possess a caudally oriented spinous process, and dorsoventrally short and craniocaudally long vertebra. The interrelationships between the functional anatomy in relation to positional behavior and thoracic vertebral morphology are discussed. (COI:No)

### PP-422

### Developmental process of cranial bones and closure of anterior fontanelle in fetal and perinatal Japanese macaques

Wataru Yano[1], Naomichi Ogihara[2] ([1]*Biol. National Defense Medical College,* [2]*Grad. Sch. Sci. Tokyou Univ*)

Mobility of cranial bones in neonates is essential for human parturition. Prolonged opening of cranial fontanelles has been listed as one of the features unique to humans, because of large neonate brain size compared to the size of the mother's birth canal. Previous work on fontanelle opening/closing has focused to humans. However, due to limited availability of fetal and perinatal specimens, developmental association between fontanelle closure and parturition in non-human primates has not been well documented. We explored development of cranial bones and closure process of anterior fontanelle in fetal and infantile Japanese macaques (*Macaca fuscata*). A total of 48 specimens from the Japanese Monkey Centre were scanned with micro and medical CTs. The process of closure of calvaial bones was quantified with respect to the developmental change of width of anterior fontanelle. The calcification of dental cusp was used as a proxy of relative age. We found that while there remained the opening of anterior fontanelle at parturition, it rapidly closed afterward. The results suggested the heterochronic modification in the development of the cranium between humans and Japanese macaques. (COI:Properly Declared)

### PP-423

### Crown size of maxillary first molars in native Taiwanese; Yami, Ami and Bunun tribes

Shintaro Kondo[1], Yoshitaka Manabe[2], Joichi Oyamada[2] ([1]*Dept. Anat., Nihon Univ. Sch. Dent. at Matsudo,* [2]*Dept. Oral Anat. Dent. Anthrop., Nagasaki Univ. Grad. Sch. Biomed Sci*)

The sexual and inter-population differences in the crown structures of the maxillary first molar were analyzed. The materials were the plaster casts of native Taiwanese of Yami, Ami and Bunun tribes, and Japanese. The mesiodistal and buccolingual crown diameters, and the four cusp diameters were measured using by a digital caliper. The cusp diameter was defined as the diagonal distance from the central pit to the most prominent convexity on the crown outline corresponding to the relevant cusp. Sexual and inter-population differences were detected by Tukey-Kramer HSD. Sexual differences were greater in the buccolingual diameters than in the mesisodistal, and also greater in the distal cusp diameters than in the mesial cusp diameters. Since sexual differences were not evident in most of the index values, intra-population variances were mainly observed in size, and it is considered that they were unlikely to appear in shape. Interpopulation differences were noted in the metacone diameter and index. It was revealed that Taiwanese and Japanese had almost the same crown in size, but the distobuccal portion was more reduced in Taiwanese than in Japanese. (COI:No)

### PP-424

### Application of Artificial Intelligence to Dental Science

Yuriko Igarashi[1], Fumio Uchikoba [2], Minami Kaneko[2], Megumi Aibara[2], Yu Yoshikawa[2], Shintarou Kondou[1] ([1]*Nihon Univ. School of Dent. at Matsudo,* [2]*Collagge of Sci. and Tech*)

In order to investigate the effectiveness of artificial intelligence (AI) in identifying the morphology of teeth, we attempted to classify the shape of teeth using a deep learning system. Dental plaster casts were obtained for 24 students at the Nihon University School of Dentistry at Matsudo (12 males and 12 females). The buccal (labial), lingual and occlusal surfaces of the cast teeth were photographed to obtain static images of the lower left central incisors, first molars, first premolars, second premolars, lower right first premolars and second premolars. The lower left central incisors and molars could be classified with 100% rate of concordance with a model trained using 0.01 as the learning rate. For the lower premolars, the accuracy could not be more than 28% for a 0.01 learning rate model, so a model with a 0.001 learning rate was constructed. As a result, it was proved that the model was valid. At the same time, the accurate identification of human premolars appears to be a difficult task for AI. This difficulty might result from the variability of the shape of lower premolars, especially first premolars.
*Conflict of Interest*: The authors declare no conflict of interest. (COI:No)

### PP-425

### Mitochondrial dysfunction in human iPSC-derived neurons under enhanced carbonyl stress: a possible role in the pathogenesis of schizophrenia

Tomonori Hara[1,2], Manabu Toyoshima[2], Yasuko Hisano[2], Yuji Owada[1], Takeo Yoshikawa[2] ([1] *Laboratory of Molecular Psychiatry, RIKEN Center for Brain Science,* [2] *Department of Organ Anatomy, Tohoku University Graduate School of Medicine*)

Schizophrenia (Sz) is a debilitating mental disorder elicited by multiple genetic and environmental components and their interactions. Recently, a clinical subgroup of Sz is proposed, which displays enhanced carbonyl stress. Carbonyl stress is a state of excess accumulation of glycated proteins, named advanced glycation end products (AGEs).
We established "*In vitro* enhanced carbonyl stress model" using human iPS cells (iPSCs) and iPSC-derived neurons. Carbonyl stress was induced intrinsically by disruption of the gene for enzyme that catalyzes AGEs detoxification, and extrinsically by administration of the precursor for AGEs. Carbonyl stress evoked decreased cell viability and increased apoptosis, and disturbed differentiation of iPSCs into neurons. In cellular levels, it caused mitochondrial dysfunction, as revealed by JC-1 staining, ATP assay and metabolic analysis using extracellular flux analyzer. Western blotting showed increased accumulation of AGEs in isolated mitochondria.
These data suggest that carbonyl stress may have a role in the pathophysiology of Sz through mitochondrial dysfunction.
I have no financial relationships to disclose. (COI:No)

### PP-426

### Distinct *dystonin* gene mutations differently affect epidermal *Dst-e* isoform and hemidesmosomes.

Nozomu Yoshioka[1], Yudai Kabata[2], Momona Kuriyama[1], Norihisa Bizen[1], Li Zhou[1], Dang Tran[1], Masato Yano[1], Atsushi Yoshiki[3], Tatsuo Ushiki[4], Thomas Sproule[5], Riichiro Abe[2], Hirohide Takebayashi[1] ([1]*Niigata Univ., Grad Sch Med., Neuroanat,* [2]*Niigata Univ., Grad Sch Med., Div Dermat,* [3]*RIKEN BioResource Res Cent,* [4]*Niigata Univ., Grad Sch Med., Div Microscop Anat,* [5]*The Jackson Laboratory*)

Loss-of-function mutations in *dystonin* (*DST)* cause neurological disease or skin disease. A single *DST* gene encodes three *DST* isoforms: *DST-a* (neuronal isoform), *DST-b* (muscular isoform), and *DST-e* (epidermal isoform). To reveal the mechanisms of the phenotypic heterogeneity of *DST*-related diseases, we investigated two mutant strains with different mutations: a spontaneous *Dst* mutant ($Dst^{dt\text{-}23Rbrc}$ mice) and a gene-trap mutant ($Dst^{Gt}$ mice). The $Dst^{dt\text{-}23Rbrc}$ allele harbors a nonsense mutation in an exon shared by all *Dst* isoforms. The $Dst^{Gt}$ allele is predicted to inactivate *Dst-a* and *Dst-b* isoforms but not *Dst-e*. The expression of *Dst-a* decreased in the neural tissue of $Dst^{dt\text{-}23Rbrc}$ and $Dst^{Gt}$ mice. In contrast, *Dst-e* mRNA expression was reduced in the skin of $Dst^{dt\text{-}23Rbrc}$ mice but not in $Dst^{Gt}$ mice. *Dst-e* encodes a structural protein in hemidesmosomes (HDs). Structural abnormalities of HDs were observed in the basal keratinocytes of $Dst^{dt\text{-}23Rbrc}$ mice but not in that of $Dst^{Gt}$ mice. These results indicate that distinct mutations within the *Dst* gene can cause different loss-of-function patterns among *Dst* isoforms, which accounts for the heterogeneous phenotypes of *DST*-related diseases. (COI:No)

### PP-427

### Maternal immune activation impairs neurogenesis and increases the expression of Atf4 in mouse fetal brain

Tsuyoshi Tsukada[1], Hiromi Sakata-Haga[1], Hiroki Shimada[2], Hiroki Shoji[3], Toshihisa Hatta[1] ([1]*Dept. Anatomy, Kanazawa Medical University,* [2]*Dept. Medical Science, Kanazawa Medical University,* [3]*Dept. Biology, Kanazawa Medical University*)

Maternal immune activation (MIA) in mid-pregnancy period is a risk factor for neurodevelopmental disorders. Recently, the notion that unfolded protein response (UPR) signaling regulates neurogenesis has been suggested. However, little is known about the UPR in neurogenesis of MIA. In this study, C57BL/6J mice were received a single intraperitoneal injection of 20 mg/kg polyriboinosinic–polyribocytidylic acid (poly[I:C]) on embryonic day 12.5, and the effects on fetal brain were examined using quantitative real time PCR, immunohistochemistry. In MIA model, the proportion of Pax6-positive neural progenitor cells and Pax6/Tbr2 double positive cells increased at 24 h after the poly(I:C) injection. On the other, there were no difference in the proportion of Tbr1-positive post-mitotic neuron at 48 h after the poly(I:C) injection. At late embryonic day, there were more Pax6-positive and Tbr2-positive neural progenitor cells in the poly(I:C) injected group. One of the UPR signaling molecules, Atf4 was significantly upregulated at 24 h after the poly(I:C) injection. MIA may impair neurogenesis via Atf4 mediated UPR.
(COI:Properly Declared)

### PP-428

### High-salt diet failed to suppress $Na^+/K^+$-ATPase activity on colonic epithelium in hypertensive Dahl salt-sensitive rats

Megumi Tandai-Hiruma[1], Yuji Morimoto[1] ([1]*Dept Physiol, Natl Def Medical Coll*)

We have previously demonstrated that high-salt diet decreases $Na^+$ transport driven by $Na^+/K^+$-ATPase (NKA) pump on the basolateral membrane of colonic epithelium in normotensive Sprague Dawley (SD), but not in hypertensive Dahl salt-sensitive (DSS) rats. The aim of this study is to investigate whether high-salt diet decreases the NKA protein expression and its enzymatic activity on the colonic epithelium in SD and DSS rats. Male SD and DSS rats were divided into two groups: one fed on a high-salt diet (SD-H, DSS-H), and the other fed on a regular diet (SD-R, DSS-R) for 4 weeks. The colon was removed from each rat group and mucosa-submucosal preparations were obtained. The expression of NKA protein on the membrane was measured by Western blotting. NKA activity was measured as an ouabain-sensitive ATPase activity. Both the NKA protein expression and its enzymatic activity in SD-H was less than those of SD-R. In contrast, any difference was not found in those values between DSS-H and DSS-R. These results imply that high-salt diet decreases the NKA protein expression on the membrane and its enzymatic activity in normotensive SD rats, but not in hypertensive DSS rats. (COI:No)

### PP-429

### Immunohistochemical distribution of the b-amyloid accumulation in the brain of the triple transgenic Alzheimer's disease model mice.

Munenori Ono[1], Tetsufumi Ito[2], Yoshie Hori[1], Shinji Muramoto[1], Sachiko Yamaki[1], Ryo Yamamoto[1], Nobuo Kato[1] ([1]*Dept. physiol, Kanazawa Medical Univ,* [2]*Dept. Syst. Func. Morphol, Toyama Univ*)

The triple transgenic Alzheimer's disease model (3xTg ) mouse is a popular model animal of AD. It is well documented that the brain of 3xTg mice begins to bear it with aging. However, how age-dependent accumulation of Aβ proceeds in time and space remains much less known in 3xTg mice than in human patients.
Here, we examined the distribution of Aβ in the brain of 3xTg mice of different ages by immunohistochemical staining. At 3 months old, Aβ was already broadly accumulated in brain structures including the olfactory area, neocortex, hippocampus, amygdala, midbrain, and brainstem. In the midbrain and brainstem, motor-related nuclei frequently contained beta-amyloid. The regional and age differences of accumulation were quantitatively examined by counting immuno-positive neurons, as well as extracellular Aβ deposits. It was shown that in the neocortex and hippocampus, Aβ accumulation substantially increased from 3 to 6 months old, whereas in other regions, the accumulation was not drastically changed after 3 months old. In the neocortex, Aβ accumulation in female mice was more broadly distributed than in males, particularly extending into layer VI. (COI:No)

### PP-430

### A grape pomace, a recyclable resource from winemaking, inhibits ultraviolet B-induced tanning in hairless mice

Mana Tsukada[1], Wakako Yogi[1], Tatsuki Inoue[2], Mami Kato[1], Takayuki Okumo[1], Tadashi Hisamitsu[1], Masataka Sunagawa[1] (*[1]Department of Physiology, School of Medicine, SHOWA University,, [2]Department of Urology, School of Medicine, Showa University*)

**Introduction:** Our previous study revealed that aqueous extract of grape pomace (GP) obtained from a winemaking process exerts a strong antioxidative effect. We therefore investigated the preventive effect of GP on ultraviolet B (UVB) -induced tanning.
**Methods and Results:** Hairless mice were divided into four groups: no-radiation (control), mice exposed to UV radiation (UV), GP-treated mice (GP), and GP+UV-treated mice groups. GP was mixed with powdered chow at a concentration of 3% and administered 14 days prior to the radiation and finally for 28 days. The UV-treated groups were exposed to UV light 3 times a week for 14 days. The melanin and erythema values were measured using Mobile Skin tone TP20, and the expression of Microphthalmia transcription factor (Mitf) and melanin production were investigated morphologically. All values were increased following UV radiation, but the administration of GP inhibited these increases.
**Discussion:** A melanin production is mediated by tyrosinase whose expression is regulated by Mitf. These results suggest that GP might have a preventive effect on sunburn via the inhibition of melanin production. (COI:No)

### PP-431

### Reduction of brain damage size and recovery of neurological dysfunction were different between different strains in mice: Evaluation using a novel ischemic stroke model.

Yasuki Matano[1], Yuto Nojiri[1], Mizuki Nomura[1], Akira Masuda[1], Yuki Moriike[1], Yasuhiro Suzuki[2], Kazuo Umemura[3], Nobuo Nagai[1] (*[1]Laboratory of Animal Physiology, Division of Bioscience, Nagahama Institute of Bio-Science and Technology., [2]School of Pharmaceutical Sciences, Faculty of Pharmacy, Ohu University., [3]Department of Pharmacology, Hamamatsu University School of Medicine*)

We established a novel murine ischemic brain damage model by using photochemical reaction, in which a reproducible damage was induced in the frontal lobe of the cortex, and accompanied by neurological dysfunction. Using this model, the sequential changes in both damage size and neurological dysfunction were studied in mice of two different strains, C57BL/6J and BALB/c. Since the initial damage size was comparable in both strains, its reduction was faster in BALB/c mice than in C57BL/6 mice. In neurological dysfunction, the sensory dysfunction shown by the von Frey test was comparable between two strains. Although the motor dysfunction shown by the balance beam test was comparable in both strains, it shown by the tail suspension test was more prolonged in C57BL/6J mice than in BALB/c mice. These findings show that the novel ischemic stroke model is useful for evaluating the recovery of neurological dysfunction together with brain repair reactions. In addition, it was found that both the recovery of neurological dysfunction and the brain repair reactions were prolonged in C57BL/6J than in BALB/c. (COI:No)

### PP-432

### Generation of the epileptic focus by glia

Shun Araki[1], Ryo Ito[2,3], Ko Matsui[1,2] (*[1]Super-network Brain Physiology, Graduate School of Medicine, Tohoku University, [2]Super-network Brain Physiology, Graduate School of Life Sciences, Tohoku University, [3]Department of Physics, Faculty of Science, Tohoku University*)

Partial seizures often originate from a localized region within the brain called the epileptogenic focus. Astrocytes modulate neuronal activity by controlling neurotransmitters and ion concentrations in the extracellular space. The abnormal local environment created by the activity of astrocytes could result in the generation of the epileptic focus. Frequently occurring spontaneous focal epilepsy was observed in a mouse model. To observe the astrocytes' activity *in vivo*, Ca2+ sensor fluorescent protein selectively expressed in astrocytes was monitored using the fiber photometry method. Before generalized seizures started to occur, large amplitude Ca2+ elevations were frequently observed in astrocytes which was accompanied with a subtle epileptic behavior; however, surprisingly, no noticeable neuronal discharge was observed. When the mouse started to generate secondary generalized seizures, strong neuronal oscillatory discharges were observed which was coincident with the occurrence of the astrocytic Ca2+ elevations. These findings suggest that hyperactive environment at the focus is first sensed and probably created by the astrocytes. (COI:No)

### PP-434

### Schizophrenia-like behavior and increased oxidative stress of parvalbumin-expressing interneurons in PlexinA1-deficient mice

Kazunori Yukawa[1], Mst Sharifa Jahan[1], Asami Suzuki[1], Momomi Kutsuna[1], Ikuko Takahashi[2], Takamasa Tsuzuki[1], Takayuki Negishi[1] (*[1]Department of Physiology, Faculty of Pharmacy, Meijo University, Japan, [2]RI Center, Facul Pharm, Meijo Univ*)

PlexinA1 (PlxnA1) is a transmembrane receptor for semaphorins, a large family of molecules acting as axonal guidance cues during the development of nervous system. PlxnA1 is expressed in embryonic interneurons, and deletion of PlxnA1 in mice leads to a decrease of interneurons in the developing cortex. In the adult mouse cortex, PlxnA1 is found to be expressed in the parvalbumin-expressing interneurons (PVIs). PlxnA1 has also been identified as a schizophrenia susceptibility gene. However, PlxnA1 function in neurobehavior remains to be fully investigated. In the behavior analysis to investigate the role of PlxnA1 in complex behavior, PlxnA1 KO mice showed significantly enhanced self-grooming and rearing in the open-field and reduced prepulse inhibition, a reliable phenotype for exploring the neurobiology of schizophrenia. The immunohistochemical analysis (IHC) to explore the cause of the abnormal behaviors revealed a decreased tendency of PVIs in medial prefrontal cortices of PlxnA1 KO mice. Significantly increased oxidative stress in PlxnA1-deficient PVIs was revealed by IHC of 8-oxo-dG, a marker of oxidative stress, which may account for the abnormal behavior in PlxnA1 KO mice. (COI:No)

### PP-435

### Inhibitory effect of lactulose on AOM/DSS-induced inflammatory tumorigenesis of colorectal cancer in mice

Keizo Hiraishi[1], Zhao Feiyan[2], Li Xiaodong[1], Lin Kurahara[1], Zhang Heping[2], Katsuya Hirano[1] (*[1]Dept. Cardiovasc. Physiol., Fac. Med., Kagawa univ., Kagawa, Japan, [2]Key Laboratory of Dairy Biotechnology and Engineering, Ministry of Education, Inner Mongolia Agricultural univ., P.R.China*)

The ulcerative colitis is associated with an increased risk of colorectal tumors. Whether this tumorigenic process involves intestinal flora remains unknown. Lactulose is a non-digestible galactose-fructose disaccharide, which might alter the diversity of intestinal flora. The present study investigated the effect of lactulose on the inflammatory tumorigenesis using a mouse model of colorectal cancer. Eight-week old female C57BL/6NCrSlc mice received a single i.p. injection of 12 mg/kg BW azoxymethane (AOM), followed by administration of 2 % sodium dextran sulfate (DSS) in drinking water for 7 days/set by 3 sets with a 7-day interval. For lactulose treatment, mice were fed with 2 % lactulose-containing chow after AOM/DSS treatment for 14 weeks. The evaluation was performed 21 weeks after AOM injection. AOM/DSS treatment induced development of colorectal tumor, decreased the microbial diversity of intestinal flora. Lactulose treatment significantly suppressed the tumor development and increased microbial diversity and short-chain fatty acid-producing bacteria. The findings suggest that lactulose suppressed inflammatory tumorigenesis by improving the diversity of intestinal flora. (COI:No)

### PP-436

### *In vivo* glial pH in epilepsy

Yoko Ikoma[1], Ko Matsui[1] (*[1]Super-network Brain Physiology, University of Tohoku, Japan*)

Glial cells react to their surrounding neuronal activity. Such glial reaction can also feedback to neurons influencing their activity in return. We focused on the intracellular pH dynamics of astrocytes in response to epileptiform activity in *in vivo* mice. We have shown previously that intracellular acidification leads to glutamate release and alkalization leads to gap junction closure between astrocytes. Therefore, the dynamics of pH in astrocytes would likely have a large consequence on the epileptiform activity. In this study, intracellular sub-membrane pH was studied using Lck-E2GFP pH sensor expressed specifically in astrocytes. Fiber photometry method was employed to monitor the changes in the pH response over time course of days. Hippocampal electrical stimuli were repeated every hour for 12 hours during dark phase to induce efficient kindling. We found that astrocytic pH dynamics vary between hippocampus, central thalamus, and hypothalamus. Countering the pH changes with optogenetic tools may prove to be an effective therapeutic strategy; however, the changes need to be carefully tuned as the changes vary between regions and during various stages of epilepsy. (COI:No)

### PP-437

### Lansoprazole exacerbated bleomycin-induced peritoneal sclerosis

Yuta Yamamoto[1], Naoko Yamagishi[1], Takao Ito[1], Yoshimitsu Kanai[1] ([1]*Dep. Cell Biol. Wakayama Med. Sch*)

Bleomycin is used as an anticancer agent and used to prepare a pulmonary fibrosis model in experimental medicine. We investigated whether the gastric ulcer drug lansoprazole could also be used to suppress pulmonary fibrosis because it suppressed liver fibrosis through suppression of Tgf-$\beta$ activation in a dietary nonalcoholic steatohepatitis model. Rats were subcutaneously treated with lansoprazole (LAP), bleomycin (BLM) or bleomycin and lansoprazole (LAP + BLM) for 28 days. The hypertrophic visceral peritoneum was strongly induced in livers of LAP+BLM group, which could be macroscopically confirmed. Histologically analysis indicated that a strong thickening of the visceral peritoneum was observed in LAP+BLM group but neither LAP or BLM group. Furthermore, the expression of Tgf$\beta$ and Col1a1 gene were increased in the LAP + BLM group. The bleomycin interview form reported that the hypertrophic visceral peritoneum was observed in the rats treated with bleomycin continuously. Thus, lansoprazole may have promoted the hypertrophic visceral peritoneum in the liver of rats treated with bleomycin. (COI:No)

### PP-438

### Development of microparticle inhalant containing hyaluronic acid-coated lipid nanoparticles for pulmonary administration of siRNA

Kaori Fukushige[1,2], Tagami Tatsuaki[2], Eiichi Goto[2], Hirai Shuichi[1], Naoyuki Hatayama[1], Hiroki Yokota[1,3], Takashi Nakano[1], Tetsuya Ozeki[2], Munekazu Naito[1] ([1]*Dept. Anatomy, Sch. Med., Aichi Med. Univ., Aichi, Japan,* [2]*Dept. Drug Delivery and Nano Pharmaceutics, Grad. Sch. Pharmaceutical Sciences, Nagoya City Univ., Aichi, Japan,* [3]*Dept. mechanical engineering, Fac. science and technology, Meijo Univ., Aichi, Japan*)

Development of medicines using small interfering RNA (siRNA) is expected. The liposome-protamine-DNA complex (LPD) is an effective cationic carrier of genes. In a previous study, we used hyaluronic acid (HA) coating of LPD (LPDH) with the goal of improving cationic drug carrier cytotoxicity.
In this study, we aim to develop LPD- or LPDH-containing spray-freeze-dried particles (SFDPs) for therapeutic delivery of siRNA to the lungs. Pulmonary administration of biopharmaceuticals consisting of functional polymers is expected to be more widely used in the future. LPD- or LPDH-containing SFDPs (LPD- or LPDH-SFDPs) were synthesized and their structure and function as gene carriers were evaluated using physical and biological methods.
The particle size of LPDH, but not of LPD, was constant after re-dispersal from the SFDPs and the amount of siRNA encapsulated in LPDH was larger than that in LPD. The in vitro pulmonary inhalation properties were almost the same. The cytotoxicity of LPDH-SFDPs was greatly decreased and only LPDH-SFDPs had a significant gene silencing effect. These results indicate that compared with LPD, LPDH is more useful for developing SFDPs for siRNA pulmonary inhalation. (COI:No)

### PP-439

### Donepezil, a drug for Alzheimer's disease, promotes muscle differentiation during the process of regeneration

Hiroshi Todaka[1], Mikihiko Arikawa[2], Tatsuya Noguchi[3], Atsushi Ichikawa[1], Takayuki Sato[1] ([1]*Dept Cardiovasc Control, Kochi Med Sch, Kochi, Japan,* [2]*Dept Biol Sci, Fac Sci Tech, Kochi Univ, Kochi, Japan,* [3]*Dept Med Cardiol and Geriatr, Kochi Med Sch, Kochi, Japan*)

The skeletal muscle regeneration is suppressed by severe stress, resulting in a development of muscle disease. Therefore, regulating the regeneration processes such as proliferation, differentiation and fusion of satellite cells under severe stress conditions is quite important for therapy of muscle disease. In this study, we showed that donepezil, an acetylcholinesterase (AChE) inhibitor for Alzheimer's disease, elevated myogenic regulatory factors and accelerated the myotube formation in C2C12 myoblast cells. Furthermore, the incrementally shift of the cross-sectional area of myofibers occurred in the donepezil-treated mouse model of cardiotoxin injury. These results suggest that donepezil has a promotive effect on muscle differentiation. However, other kinds of AChE inhibitors could not reproduce such effect, indicating that the specific characteristics of donepezil in the promotion of muscle differentiation would be independent of its AChE inhibitory action. Further studies are needed for understanding the molecular mechanism of the donepezil-specific promotive effect on the muscle differentiation to establish a new therapeutic strategy for the treatment of muscle disease. (COI:No)

### PP-440

### Use-dependency of $I_{Kr}$ channel inhibition in cardiac ventricular myocyte: a simulation study

Tomohisa Kawakami[1], Chinatsu Kobayashi[1], Rina Sato[1], Kazuki Okumura[1], Akira Amano[1], Yukiko Himeno[1] ([1]*Dept Bioinfo, Grad Sch Life Sci, Ritsumeikan Univ, Shiga, Japan*)

It is known that the effect of $I_{Kr}$ channel blockers on cardiac myocytes depends on the frequency of the electrical stimulation. However, the details of the mechanisms of this use-dependency are unknown. In this study, we hypothesized that the use-dependency stems from the differences in the rate constants of the binding of the drug depending on whether the channel is in open or closed. We incorporated inhibition states into two types of activation gates ($y_1$ and $y_2$ gates), but not for the inactivation gate ($y_3$ gate of $I_{Kr}$ channel model). Then, we evaluated the pharmacological action from the monophasic action potential waveform obtained by the suction electrode experiment using the Langendorff heart, and tried to refine the rate constant of the model. From the experiment, we adjusted the rate constant of $I_{Kr}$ channel inhibition by using the dissociation constant ($K_d$) and the time constant (t) of the time course of the prolongation of the action potential duration at 90 % repolarization (APD90) measured experimentally at different stimulation frequencies under the effect of E-4031. (COI:No)

### PP-441

### Development of bone model with rewritable surface

Takeshi Baba[1], Daisuke Hoshi[1], Takahiro Suzuki[2] ([1]*Ibaraki Pref.Univ.Med.Sci.,* [2]*Shigray*)

It is difficult to carry out anatomy training using the actual human body at paramedical universities lacking medical school in Japan. Instead, we are conducting anatomy practices using human body models, bone models, and paper-based teaching materials. However, there is a limit to understanding the three-dimensional structure with those materials.
We developed a "color-paintable bone model" that can be repeatedly erased by adding a special coating to the bone model surface. We report here on the experience of using it in the actual class. (COI:No)

### PP-442

### Two dimensional models showing action of the pterygoid muscles

Mineo Yasuda[1], Yu Kozakai[1], Manami Suyama[1], Keiju Kamijo[1] ([1]*Div. Anat., Tohoku Med. Pharm. Univ. Fac. Med*)

Recent reduction of teaching hours for gross anatomy in Japanese medical schools necessitates improvements on teaching materials. Here we demonstrate models showing action of pterygoid muscles. The skull base including the pterygoid process and the mandible including the ramus of mandible in frontal and lateral aspects are schematically drawn on sheets of cardboard, and the mandible is cut out. Lines showing the main direction of contraction of the lateral and medial pterygoid muscles are drawn between the pterygoid process (origin) and the mandibular ramus (insertion). At the origin and insertion points, holes are drilled and sprinted pins are inserted at the insertion. Grooves are cut on the card board following the lines of movement. Strong strings are fixed at the insertion pins, and the other free end of the strings are pulled out through the holes at the origin points. Contractions of the muscles are represented by pulling the strings toward the origins. By these models, students can easily understand functions of the pterygoid muscles. (COI:No)

### PP-443

### Questionnaires of Anatomical dissection course amid COVID-19 pandemic for students at Kurume University School of Medicine

Yoko Tabira[1], Aya Han[1], Akihiro Yamashita[1], Tsuyoshi Saga[2], Keishirou Kikuchi[3], Kunimitsu Nooma[1], Eiko Inoue[1], Daisuke Uejo[1], Koichi Watanabe[1] (*[1]Department of Anatomy, Kurume University School of Medicine, [2]Domain of Anatomy, Kurume University School of Nursing, [3]Department of Orthopedic Surgery, Kurume University School of Medicine*)

Our department has been conducting questionnaire surveys for the students finishing the anatomical dissection course since 2018. The course is usually held 13 weeks (38 sessions). In this year, we had to change the program due to COVID-19 pandemic. We gave subjects on line to students until the course started. In the dissection course, the countermeasures for avoiding three Cs were done including reduction of the number of dissection groups, division of the members in the group into two, and each sub-group joining the course in turn.

We compared the results of the questionnaires in 2018 and 2019 with that in 2020 to analyze the effectiveness of the course in this year. The achievement level in the affective domain was similar to the previous year. However, the level in the cognitive and psychomotor domains decreased in 2020. The level solving the problems and shared the knowledge in the group were also insufficient. On the other hand, the ability of collection of the findings were improved due to the pre-work. In this presentation, we discuss the improvement of our dissection course based on the results of the questionnaires. (COI:No)

### PP-444

### Video content for self-learning of medical images useful for online education

Hitoshi Ueno[1], Tohru Murakami[2], Sachiko Awata[3], George Matsumura[1], Miki Nagase[1] (*[1]Department of Anatomy, School of Medicine, Kyorin University, [2]Department of Anatomy, Graduate School of Medicine, Gunma University, [3]Department of Diagnostic Radiology, Interventional Radiology and Nuclear Medicine, Gunma University Hospital*)

In recent years, with the spread of autopsy imaging (Ai), the number of universities that provide medical imaging education using body donations in gross anatomy training is increasing. However, it is very difficult to educate medical images at the same time as gross anatomy because sufficient educational time is not available in this corona-damaged situation. In addition, it is very troublesome to teach the operation of medical image analysis software by online learning. Therefore, this time, I made a video of an anonymized normal human body CT, not a body donation, with annotated cross-sectional images from the neck to the pelvis. This video can be viewed not only on PCs but also on smartphones and tablets, which is useful for self-learning. In addition, it can be used without the need for special applications and is easy to operate. Furthermore, since it is a moving image, the faults can be seen continuously, making it easier to understand the vertical connection. We have also created videos of the lung and liver areas, which are expected to be useful not only for anatomical training but also for clinical education. (COI:No)

### PP-445

### Assessment of the relationship between learning behavior and examination results using "Learning Analytics" in Moodle LMS.

Junna Nakamura[1], Chihiro Mochizuki[1], Hyungjin Kim[1], Michihiro Nakamura[1] (*[1]Dept.Organ Anat. & Nanomedicine Grad. Sch. Med. Yamaguchi Univ*)

We have introduced the Moodle online learning management system (LMS) for anatomy education. In histological class, we are using Moodle for distributing handouts, providing self-study exercises, the conduct quizzes and final exams, the submission of assignments, peer assessment in student's presentation, and aggregating the questionnaire, etc, these activities are recorded in the Moodle log file. We can obtain quantitative and objective data on student learning behavior and subjective information of the students such as opinions and self-evaluation by the questionnaire. We have analyzed learning behavior data in the Moodle logs and have begun investigating the relationship between learning behavior and examination scores. The subjects were the top 20 and bottom 20 students in the final exam of histology. We compared their quizzes average score, the submission date of the assignment, the number of views of the preparation video, and when, how long, and how many times they took the self-study exercise and what they are answering in the questionnaire. In this study, I would like to report on the evaluation method of learning behavior and the results so far. (COI:No)

### PP-446

### Educational use of  visualization of locomotion

Anshin Asano-Hoshino[1] (*[1]Department of sport and Medical Science, Faculty of Medical Technology, Teikyo University*)

Although locomotion of human is it is possible to move smoothly by bone and muscle, the two legged walking robot can not be smooth. The difference between the two is that humans have evolved to acquire special muscles that exceed two joints called biarticular muscles, but one reason is that robots are not equipped with that mechanism. The biarticular muscles include the biceps brachii muscle, but in reality, the function of the biarticular muscle cannot be said to be fully functionally understood. So far, we have observed the acquisition process of walking movement by exposing to low water level using Sturgeon as an experimental animal approach, development of educational and research equipment for biarticular muscle using Kuma model, and real time instruction system using AI and Smart-phone technology . Recently, due to the influence of the covid-19, the social distance is maintained, and online lectures and on-demand lectures are increasing rather than face-to-face lessons. Therefore, by using the system of "technological development to visualize locomotion" for education, it may be possible to evaluate the ability by objective visualization and quantification. (COI:No)

### PP-448

### Rosette agent (*Sphaerothecum denstruens*) infection in laboratory medaka

Toshiyuki Nishimaki[1], Takafumi Katsumura[1], Noriko Nemoto[2], Kazuko Fujitani[3], Hiroki Oota[4], Atsunori Oga[5], Isao Okayasu[6], Michael Kent[7], Shoji Oda[8], Motoyuki Ogawa[1] (*[1]Department of Anatomy, Kitasato University School of Medicine, [2]Research Center for Biological Imaging, Kitasato University School of Medicine, [3]Gene Analysis Center, Kitasato University School of Medicine, [4]Graduate School of Science,The University of Tokyo, [5]Department Molecular pathology, Yamaguchi University Graduate School of Medicine, [6]Kitasato University School of Medicine, [7]Department of Microbiology and Biomedical Sciences, Oregon state University, [8]Department of Integrated Bioscience, Graduate School of Frontier Science, The University of Tokyo*)

**Abstract**

It is a prerequisite to determine the cause of experimental animals' morbidity and death for the reliability and reproducibility of life science experiments. In medaka (*Oryzias latipes*), which has been used as small experimental fish, some reports have documented microbial infections observed in the other aquarium fish; however, the causes of morbidity and death in a laboratory are seldom determined. To accumulate the histological and pathological knowledge in medaka, we have performed histopathological examinations to address the cause of morbidity and death in medaka.

In this study, we conducted the histopathological examinations of dying medaka with a swollen abdomen and the severe `deep mycoses` in the multiple organs and found that medakas were seriously infected with *Sphaerothecums destruens*, formerly known as rosette agent. *S. destruens* sits at the animal-fungal boundary and has been first reported in farmed salmon in the United States in 1986. As far as we know, this is the first report of *S. destruens* infection in Japan and supposes the possibility that *S. destruens* can be a common parasite in the Asian wild ecosystem. (COI:No)

### PP-449

### The reaction of FEL irradiation to calculus formation causative bacteria *Corynebacterium matruchotti.*

Yuma Sasamoto[1,2], Tetsuro Kono[2], Takeshi Sakai[3], Ken Hayakawa[3], Heishun Zen[4], Toshiteru Kii[4], Masanori Saito[5], Arata Watanabe[2], Miyuki Toda[1,2], Ryo Tamamura[2], Hideaki Ohgaki[4], Yasushi Hayakawa[3], Toshiro Sakae[2], Hiroyuki Okada[2] (*[1]Grad. Sch. Dent. Matsudo., Nihon Univ., [2]Nihon Univ., Sch. Dent.Matsudo.,Dept.Hist., [3]Nihon Univ., Inst.Quantum Sci., [4]Kyoto Univ., Inst. Advanced Energy., [5]Nihon Univ., Sch.Dent.Matsudo., Dept. Microbio Immun*)

The purpose of this research was to find a light reaction that promotes the formation of carbonates in organisms with carbonate shells (bivalves, brachiopods, corals, foraminifera, bacteria with lime potential, etc) to immobilize carbon dioxide in the atmosphere. Using Free Electron Laser (*FEL) by* KU-FEL at Kyoto University and Nihon University's FEL (LEBRA-FEL), the study was carried out for the optimal light wavelength for this reaction. As an experiment target organism, *Corynebacterium matruchotti* (*C.m*), a causative agent of calculus formation, was selected, and the result of some weeks was compared.

In this research, we confirmed changes in FTIR and XRD when FEL was both irradiated at each wavelength. This result suggests that *C.m* was changing its organic matter during calcification. There are still fluctuations in the band and peak that require detailed analysis, and further data will be searched in the future. In addition, we plan to search for wavelengths that suppress calcification ability from the wavelengths of both KU-FEL and LEBRA-FEL. (COI:Properly Declared)

### PP-450

### The Cancer Stem Cell Marker SSEA-1 in a Mouse Glioma Model

Yousuke Nakano[1], Yuki Nakai[1], Masato Maruyama[2], Susumu Tanaka[1], Shinichi Hayashi[1], Soichi Oe[1], Masaaki Kitada[1] ([1]*Anatomy. Med., KMU.*, [2]*Biopharmaceutics. Faculty of Pharma Sci., Okayama univ*)

Gliomas are tumors in the brain which recur frequently and have a very poor prognosis even after the treatment by medication or surgical intervention. Since cancer stem cells have been focused on as a cause of recurrence and metastasis after tumor treatment, we established cancer stem cell-like clones with high tumorigenic potential (U87-CSCs) from U87MG cells derived from human gliomas and found that Stage specific embryonic antigen-1 (SSEA-1), a known cancer stem cell marker in gliomas, was highly expressed in U87-CSCs compared to U87MG cells.

In the present study, we separately isolated SSEA-1(+) and SSEA-1(-) cells from U87-CSCs using fluorescent-activated cell sorter and compared the in vitro cell proliferation, sphere-forming ability, and sphere circularity. No significant differences were observed between SSEA-1(+) and SSEA-1(-) cells in all assays. On the other hand, when SSEA-1(+) and SSEA-1(-) cells were transplanted into the brain of nude mice, the size of the tumor was larger in the SSEA-1(-) cell-transplanted group compared with the SSEA-1(+) group. Furthermore, immunohistochemistry revealed a negative correlation between the density of SSEA-1(+) cells and tumor size. (COI:No)

### PP-451

### Quantification of spinal curvature and identification of its candidate genes in *wy* medaka (*Oryzias latipes*)

Takafumi Katsumura[1], Naohide Shimada[2], Kentaro Uchida[3], Toshiyuki Nishimaki[1], Masashi Takaso[3], Motoyuki Ogawa[1] ([1]*Dept. Anat., Kitasato Univ. Sch. Med.*, [2]*Dept. Biol., Kitasato Univ.*, [3]*Dept. Orthopaedic Surgery, Kitasato Univ. Sch. Med*)

The pathogenesis of adolescent idiopathic scoliosis has been studied in humans, but the causative genes have not been well-known. Focusing on *wavy* (*wy*) medaka (*Oryzias latipes*), which spontaneously develop curvature, we tackle to explore the causative gene(s) of *wy* medaka to find the candidates of novel genes in human scoliosis. In this study, we performed a quantitative analysis of the spinal curvature in *wy* medaka and searched for the genetic mutations responsible for the curvature. We measured the curvature of 31 *wy* medaka from $\mu$CT images and classified the curvature's degree by the k-means method. We next performed pool-seq to extract candidates of the spinal curvature-related mutations. We found that *wy* medaka exhibited scoliosis and were classified into five groups depending on spinal curvature. Filtered 4.7 million *wy* medaka-specific mutations obtained from the pool-seq, we detected 21 candidate genes. 11 of the 21 were on genes with a known function. We plan to generate genome-edited variants about these candidate scoliosis genes to determine which genes cause the *wy* medaka scoliosis. (COI:No)

### PP-452

### Rapid test of Mycobacterium tuberculosis in cadaver using the LAMP method

Masaaki Miura[1], Yuta Arai[1], Hideaki Tamaki[2], Takafumi Katsumura[1], Motoyuki Ogawa [1] ([1]*Department of Anatomy, Kitasato University School of Medicine*, [2]*Research and Development Center for Medical Education, Kitasato University School of Medicine* )

The cadaver used for clinical anatomy is not the formalin fixation, but an unfixed or low formaldehyde-fixed cadaver is often used. The unfixed cadaver is more flexible than formalin fixation and can perform thoracotomy and membrane peeling similar to actual clinical surgery and has many advantages over formalin fixation. It also has defect that blood tests are required due to the risk of infectious diseases. Department of Anatomy Kitasato University tests for hepatitis B, hepatitis C, syphilis, and HIV. Mycobacterium tuberculosis is also danger to be infected but usually the cadaver is not tested. Therefore, when using an unfixed cadaver for clinical anatomy, it is necessary to be careful about infection with M. tuberculosis. At the 123th Annual Meeting of The Japanese Association of Anatomists, we presented the results of M. tuberculosis test of the cadaver using the LAMP method, which can quickly determine the results. As a result, 2 out of 10 samples had a positive reaction for M. tuberculosis. This time, together with the previous results, DNA was extracted from 52 cadavers and the results of the LAMP method will be presented. (COI:No)

### PP-453

### Vitamins synergistically exert mast cell-stabilizing properties

Yukine Sato[1], Misaki Yashima[1], Itsuro Kazama[1] ([1]*Nursing, Miyagi Univ*)

Besides the physiological properties, studies revealed the roles of vitamins, such as ascorbic acid (vitamin C) and pyridoxine (vitamin $B_6$), in ameliorating the symptoms of allergic disorders. In the present study, using the differential-interference contrast (DIC) microscopy, we examined the effects of these vitamins on the degranulation from rat peritoneal mast cells. Both vitamins dose-dependently decreased the numbers of degranulating mast cells. At higher concentrations (5, 10 mM), they markedly suppressed the numbers of degranulating mast cells. At relatively lower concentrations (1, 2 mM), pyridoxine did not significantly affect the numbers of degranulating mast cells. Surprisingly, however, pyridoxine with such low doses synergistically augmented the suppressive effects of ascorbic acid. These results provided *in vitro* evidence that vitamins, such as ascorbic acid and pyridoxine, dose-dependently inhibited the process of exocytosis. Pyridoxine alone with lower doses did not stabilize mast cells. However, it synergistically potentiated the mast cell-stabilizing property of ascorbic acid. (COI:No)

### PP-454

### Caffeine and catechin synergistically exert mast cell-stabilizing properties

Misaki Yashima[1], Yukine Sato[1], Itsuro Kazama[1] ([1]*Nursing, Miyagi Univ*)

Besides health promoting functions, such as anti-oxidant, anti-cancer and anti-bacterial properties, caffeine and catechin exert anti-allergic effects. In the present study, using the differential-interference contrast (DIC) microscopy, we examined the effects of these substances on the degranulation from rat peritoneal mast cells. Both caffeine and catechin dose-dependently decreased the numbers of degranulating mast cells. At concentrations equal to or higher than 25 mM, they markedly suppressed the numbers of degranulating mast cells. In contrast, at relatively lower concentrations, both caffeine and catechin did not significantly affect the numbers of degranulating mast cells. However, low concentrations of catechin synergistically enhanced the suppressive effect of 10 mM caffeine on mast cell degranulation. The results provided *in vitro* evidence that caffeine and catechin dose-dependently inhibited the process of exocytosis. At relatively lower concentrations, caffeine or catechin alone did not stabilize mast cells. However, low concentrations of catechin synergistically potentiated the mast cell-stabilizing property of caffeine. (COI:No)

### PP-455

### Signaling in temperature-induced resting cyst formation in the ciliated protozoan *Colpoda cucullus*

Mikihiko Arikawa[1], Yuto Shimada[2], Yuya Hasegawa[2], Yuya Harada[2], Tatsuomi Matsuoka[1] ([1]*Dept Biol Sci, Fac Sci Tech, Kochi Univ*, [2]*Grad Sch Integr Art Sci, Kochi Univ*)

When habitat conditions become unfavorable, vegetative cells of the terrestrial ciliated protozoan *Colpoda cucullus* quickly transform into resting cysts. However, the critical stimulus for *C. cucullus* encystment in natural environments remains unknown. Therefore, we investigated the effect of temperature on the resting cyst formation in *C. cucullus*. The findings are as follows; 1) the encystment was induced by a rapid increase of temperature but not by a decrease, 2) an increase of intracellular $Ca^{2+}$ concentrations is essential, 3) a temperature receptor, TRP channel, was found to express in the vegetative cells, 4) the TRP channel was localized not on the cell membrane but on the vesicle membrane, and 5) $Ca^{2+}$ is stored in vesicular structures and released into the cytoplasm just after temperature stimulation. Based on these results, we concluded that when temperature stimulation was applied to vegetative cells of *C. cucullus*, $Ca^{2+}$ was released from vesicles into the cytoplasm through the TRP channels localizing on the vesicle membrane, thus increasing the intracellular $Ca^{2+}$ concentration followed by the formation of resting cysts. (COI:No)

### PP-456

### Analysis of Interrelationship between Cell Model Variables Using Jacobian Matrix Generated from CellML File

Yutaro Yagi[1], Yoshitoshi Kunieda[2], Akira Amano[1] ([1]*Department Bioinformatics, Graduate School of Life Science, Ritsumeikan Univ ersity, Shiga, Japan Zip code of the first author's contact address,* [2]*Department of Information Science and Engineering, College of information Sc ience and Engineering, Ritsumeikan University, Shiga, Japan*)

Since large number of variables and complex numerical expressions are included in biological function models, its simulation programs often include errors. To avoid such errors, usage of mathematical model described by description language such as CellML to generate simulation programs automatically is considered. Jacobian matrix is often used for model analysis such as bifurcation analysis and equilibrium point analysis. Jacobian matrix usually have many elements, which often leads to errors in its calculation program. To address this problem, we proposed a system that automatically generates Jacobian matrix calculation program by using a biological function model described by CellML. To analyze the interrelationship between cell model variables, we propose a visualization method which displays Jacobian matrix elements as a directed graph. By the method, we realized that the relationships between model variables can be visualized efficiently. (COI:No)

### PP-457

### Physiological functions of lipid enzymes involved in thermotaxis in *Drosophila melanogaster*

Xiangmei Deng[1], Takaaki Sokabe[1] ([1]*Department of physiological sciences, Sokendai, Japan*)

Finding optimal temperatures is important for survival and reproduction in living organisms. In *Drosophila melanogaster* larvae, recent studies proved that rhodopsins/Gq/phospholipase C (PLC) signaling cascade activates TRPA1 channel, which mediates larvae preferable temperature. However, how TRPA1 is activated by the downstream components of PLC remains unknown.

We hypothesized that lipid metabolites downstream of PLC regulates TRPA1 activation in larval temperature preference, including diacylglycerol (DAG), monoacylglycerol (MAG) and polyunsaturated fatty acids (PUFA). Therefore, we selected candidate genes involved in biosynthesis of DAG, MAG and PUFAs and performed temperature gradient assays using mutant larvae. As a result, we identified several putative lipases whose mutation caused defects in choosing preferable temperature. When knocking down these candidate genes in specific neurons, we observed altered temperature preference similar to their mutants. Besides, we observed $Ca^{2+}$ responses in TRPA1-expressing cells upon PUFA stimulation. These results suggest that lipases and the generation of lipid metabolites may play key roles in the temperature sensation in fruit flies. (COI:Properly Declared)

### PP-458

### Brain-derived neurotrophic factor-overexpressed transgenic mice exhibited the depressive behavior

Yui Kyobashi[1], Takashi Tsuboi[2], Rika Numano[1] ([1]*Toyohashi Univ. of Tech., Dept. of Applied Chem. and Life Sci.,* [2]*Dept. Life Sci., Grad. Sch. Art. Sci., The Univ. Tokyo, Tokyo, Japan*)

Brain-derived neurotrophic factor (BDNF) functions to survive of serotonergic neurons in the brain and to promote the neurite growth as neurotransmitters. Decreased levels of BDNF in the brain cause the inhibition of neurogenesis in the cortex and hypothalamus, result to depression. In the present study, we developed CMV:BDNF:Venus transgenic mice (CBDVe), with over-expressing BDNF by CMV promoter and detected by Venus fluorescence and investigated the behavioral phenotype of CBDVe mice for examining whether the overexpression of BDNF contributes to psychiatric disorders. We first examined the expression levels of BDNF protein in the whole brain of CBDVe mice, especially in the hypothalamus, by ELISA and found that twice as that of wild mice. We found that CBDVe mice exhibited less exploratory behavior, and more alert and fearful reveled by the open-field tests. Furthermore, the force swimming test and Morris water maze test revealed that CBDVe mice exhibited the depressive behavior and impaired memory. Taken together, these results suggest that the overexpression of BDNF in the brain may be related to cause of psychiatric disorders. (COI:No)

### PP-459

### The relationship between eye movements and emotional recognition of dynamically changed facial expressions

Yuki Harada[1,2], Shunji Oyama[2], Makoto Wada[1] ([1]*Dev Disorders Sect, Dept Brain Rehab, Res Inst of NRCD,* [2]*Human Aug Res Cent, National Inst of AIST*)

We investigated whether the recognition of facial emotion is influenced by the ratio of emotional expression, timing of changes, and eye movements. In two experiments, a face was presented on a display and changed between neutral and emotional expressions within 3 seconds. We manipulated the manner of the changes: ratio of emotional expression in Experiment 1 and the timing in Experiment 2. During the presentation, viewing times for eyes and a mouth of faces were recorded by an eye-tracking system. Subsequently, the emotionality of changes in the facial expressions was rated by using the 7-point scale. In Experiment 1, the ratio of facial expressions influenced the emotion recognition of faces. Moreover, the diameter of pupil became large when the presented face was anger or disgust. In Experiment 2, the timing of emotional expression also influenced the emotion recognition. However, in both two experiments, the viewing time of eyes and a mouth of the presented faces did not influence the emotion recognition of faces. These results suggest that the recognition of dynamically changed facial expressions is not explained by eye movements of faces. (COI:No)

### PP-460

### Tumor suppressor homologue *let-7* is regulated transgenerationally by starvation in *C. elegans.*

Luna Izuhara[1], Sawako Yoshina[1], Shohei Mitani[1] ([1]*Dept Physiol, TWMU, Tokyo, Japan*)

In previous research, reduced expression of the LET7 gene family has been described in many human tumors. *let-7* is a microRNA found in *C. elegans*, whose human homologue functions as a tumor suppressor. In addition, the human LIN28 / let-7 pathway is known to be closely involved in the maintenance of stem cell pluripotency. Recent studies in *C. elegans* suggest an intrinsic strategy in which parental experiences during developmental stages form transmissible epigenetic memories, that elicit enhanced robustness and viability in their descendants .

In this study, we investigated the transgenerational inheritance of the expression control of the nematode homologous gene *let-7*. We used temperature-sensitive *let-7* mutant allele, which has sterile and vulval malformation at the restrictive temperature. We found that starvation suppresses the phenotypes of *let-7* mutant animals. Also, starvation changes the expression stage of *let-7*; this effect is inherited through the F4 generation.

Thus, the regulation of *let-7* expression suggests that dependence of this microRNA on epigenetic regulation. It is of great interest how food-deprivation results in the epigenetic modulation. (COI:No)

### PP-461

### Voluntary running-induced an analgesic effect on persistent inflammatory pain via the inhibition of microglial activation

Hideshi Ikemoto[1], Risa Yamauchi[1,2], Naoki Adachi[1], Takayuki Okumo[1,3], Hiroyuki Horikawa [1,2], Akiou Nakamura[1], Satoshi Sakaue[1], Tadashi Hisamitsu[1], Masataka Sunagawa[1] ([1]*Department of physiology, School of Medicine, Showa University, Japan,* [2]*Faculty of Arts and Sciences at Fujiyoshida, Showa University, Japan ,* [3]*Department of Orthopaedic Surgery, Showa University Fujigaoka Hospital, Japan*)

**Introduction**: We reported that voluntary running (VR) induced an analgesic effect on persistent inflammatory pain. This study investigated the physiological mechanisms underlying the effects of VR-induced analgesia.

**Methods**: Rats were divided into control, non-running following formalin injection (NOR), and VR following formalin injection groups. Inflammation was induced by injecting formalin (50 µl, 1%) into the hindpaw, and VR was applied for 7 days after the injection. Inflammatory sensitization was tested with the von frey test. To examine the involvement of central sensitization, the expressions of activated microglia and phosphorylated p38 mitogen-activated protein kinase (p-p38) in the spinal cord were analyzed by Western blotting and immunofluorescent staining.

**Results**: In the NOR group, the withdrawal latency significantly decreased from day 1-7, and the expression levels of activated microglia and p-p38 were significantly increased compared with the control group; however, VR inhibited these changes.

**Conclusions**: These results suggest that VR may prevent the transition from acute to chronic pain through the inhibition of the activation of microglia and p-p38. (COI:No)

### PP-462

### Examining the association between a model of butyrate recovery in glyphosate-treated rats and changes in flora in a single dose of butyrate.

Takaya Inakawa[1], Kwong Soon Thomas Tiong[1], Ken Futagami[1], Yoko Nomura[2], Yasunari Kanda[3], Sachiko Yoshida[1] ([1]*Department of Applied Chemistry and Biotechnology, Toyohashi University of Technology,* [2]*Queens College, the City University of New York,* [3]*National Institute of Health Sciences*)

Glyphosate (GLY) is a major component of herbicides; however, we observed high-dose prenatal exposure to GLY induced neurological and neurobehavioral abnormalities, and butyric acid (BA) treatment after birth alleviated the neurotoxicity caused by GLY exposure. In this study, we examined the alteration of the gut microbiome in GLY-treated or GLY-BA-treated Wistar rats. Rat dams were treated with GLY (250 mg/kg-bw) on embryonic day 16 (E16), and some pups were treated additionally with BA (400 mg/kg-bw) from postnatal day 3 (P3) to P9. We also examined the effect of chronic GLY exposure (15.625 mg/kg-day, E5~E20) and low chronic GLY exposure (1 mg/kg-day, from E2 to E21). Fecal samples were collected from E7 to E21 for dams and P28 for pups. 16S rRNA sequencing was performed on DNA extracted from the fecal samples to profile the changes in the gut flora. The result showed that the Ruminococcaceae family of butyrate-producing bacteria tended to decrease in the GLY-treated group, and the chronic treatment group tended to decrease even more. We suggested that GLY exposure altered the gastrointestinal homeostasis and intestinal permeability. (COI:No)

### Student Presentations

(March 28, Sun. 18：30～19：30)

| | |
|---|---|
| **SP1-1～SP1-7** | Neurohistochemistry, Neurochemistry |
| **SP2-1～SP2-7** | Gross anatomy① |
| **SP3-1～SP3-7** | Gross anatomy② |
| **SP4-1～SP4-7** | Pathophysiology |
| **SP5-1～SP5-7** | Molecular anatomy, Molecular physiology① |
| **SP6-1～SP6-7** | Molecular anatomy, Molecular physiology② |
| **SP7-1～SP7-7** | Embryology, Regenerative Medicine, Development, Growth, Aging |
| **SP8-1～SP8-7** | Cartilage, Bone, Connective tissue / Muscle / Digestion, Digestive system, Oral physiology |
| **SP9-1～SP9-7** | Neuroanatomy, Neurophysiology, Neuronal cell biology |
| **SP10-1～SP10-6** | Ion channels, Receptors / Embryology, Regenerative Medicine, Development, Growth, Aging |
| **SP11-1～SP11-7** | Digestion, Digestive system / Circulation |
| **SP12-1～SP12-6** | Others |

### SP1-1

### Immunohistochemical analysis of huntingtin-associated protein 1 in the adult mouse brain stem

Emi Miyasato[1], Md Nabiul Islam[1], Mir Rubayet Jahan[1], Abu Md Mamun Tarif[1], Kanako Nozaki[1], Koh-hei Masumoto[1], Akie Yanai[1], koh Shinoda[1] ([1]*Yamaguchi University Graduate School of Medicine, Division of Neuroanatomy*)

Huntingtin-associated protein 1 (HAP1) is a neuronal interactor with causal agents of several polyglutamine diseases, being considered as a protective factor against neurodegeneration. In normal brains, it is abundantly expressed particularly in the limbic-hypothalamic regions that tend to be spared from neurodegeneration, whereas the areas with little HAP1 expression are targets in several neurodegenerative diseases. While the brain stem is another major neurodegenerative target, HAP1-immunoreactive structures have yet to be determined there. In this study, HAP1expression was immunohistochemically evaluated in the brain stem of the adult male mice. Our double-immunostaining for HAP1 and ChAT demonstrated that HAP1 is specifically expressed in autonomic neurons, but was specifically lacking in the brain stem motoneurons. The present study first demonstrated that HAP1 is abundantly expressed in brain stem autonomic regions but absent in motor neurons, suggesting that the motoneurons are, due to lack of putative HAP1 protectivity, more vulnerable to stresses in neurodegenerative diseases than other HAP1-expressing neurons probably involved in autonomic functions. (COI:No)

### SP1-2

### Immunohistochemical analysis of HAP1 in catecholaminergic neurons in the brainstem of adult mouse

Ayumi Yasukochi[1], Kanako Nozaki[1], Akie Yanai[1], Islam Md Nabiul [1], Tarif Abu Md Mamun[1], Masumoto Koh-hei[1], Koh Shinoda[1] ([1]*Yamaguchi University Graduate School of Medicine, Division of Neuroanatomy*)

Huntingtin-associated protein 1 (HAP1) is widely expressed as a core molecule of the stigmoid body in the limbic/hypothalamic regions and has protective functions against neurodegeneration. Recently, we found that HAP1 is highly expressed in the spinal autonomic neurons. HAP1 may also present in the other nuclei of autonomic system (catecholaminergic neurons) in the brain. In this study, we performed immunohistochemical analyses of the brains in adult male mice to determine whether these catecholaminergic neurons express HAP1. We found that most of the dopaminergic neurons localized in the substantia nigra were negative for HAP1 (a few dopaminergic neurons in the ventral tegmental area were positive for HAP1). In contrast,almost all noradrenergic and adrenergic neurons in the brainstem were HAP1 positive,indicating that HAP1-immunoreactive catecholaminergic neurons gradually increased from rostral dopaminergic neurons to caudal adrenergic neurons. These may suggest that HAP1 is related to the life-support functions of animals. Since HAP1-knock out mice died within 24 hours after birth, HAP1 deficiency in adrenergic neurons might be a critical factor in this pathological background. (COI:No)

### SP1-3

### Effects of neural activity on splicing of receptor-type PTP family molecules associated with neural circuit collateral formation

Yoshiharu Kato[1], Tokuichi Iguchi[1], Misato Yasumura[1], Yuichiro Oka[1,2], Makoto Sato[1,2] ([1]*Dept Anat Neurosci, Grad Sch Med, Osaka Univ, Osaka, Japan,* [2]*United Grad Sch of Child Dev, Osaka Univ, Osaka, Japan*)

Alternative splicing produces diverse molecules from a single gene. Some isoforms, which are encoded by mRNA species that incorporate or exclude short sequences of a gene, micro exons, are proved to play important but distinct roles in the brain development. It is shown that such an alternative use of micro exons is regulated depending on neural activity. We have previously found that some members of LAR-RPTP (receptor-type protein tyrosine phosphatase) family, which possess micro exons, are involved in axon collateral formation, one of the essential events for proper neural circuit formation. Here we asked effects of neural activity on the splicing of micro exons of LAR-RPTP family members. Neurons were collected from the cortices of embryonic day 16.5 ICR mice. After 11 days in culture, KCl was added to induce neural activity, then, the splicing isoforms were investigated. We found that the proportion of *LAR* isoform that incorporates micro exon was increased. This was unexpected since the enhancement of micro exon exclusion is reported in other family members such as *Ptprd*. This result suggests that members of LAR-RPTP family are spliced differently depending on neural activity. (COI:No)

### SP1-4

### Distribution and neurochemical characterization of HAP1 in the pituitary gland of rodents

Yui Kobayashi[1], Md Nabiul Islam[1], Kanako Nozaki[1], Akie Yanai[1], Koh-hei Masumoto[1], Abu Md Mamun Tarif[1], Koh Shinoda[1] ([1]*Yamaguchi University Graduate School of Medicine, Division of Neuroanatomy, Yamaguchi, Japan*)

Huntingtin-associated protein 1 (HAP1) has been regarded as a protective factor against neurodegeneration. We have previously showed that HAP1 is highly expressed in the hypothalamus. Pituitary gland is closely associated with the hypothalamus from the perspective of hormone secretion. To date, the distribution and neurochemical characterization of HAP1 in the pituitary gland have not been clarified. In this study, employing immunohistochemistry the expression of HAP1 in the pituitary gland and its relationships with hormone releasing cells were examined in adult rats (Wistar, BN/SsNSlc) and mice (BALB/c, C57BL/6J). HAP1-immunoreactivity was detected in all the lobes of pituitary (stronger in intermediate lobe, scattered in anterior lobe, dot-like structures in posterior lobe). The distribution pattern is similar in the pituitary gland of rats and mice. HAP1 was expressed in thyrotroph and melanotroph but not in somatotroph and lactotroph in rodent pituitary. Whereas, HAP1 was exclusively present in corticotroph of mouse pituitary gland. It will be of great interest to elucidate the pathophysiological roles of HAP1 in the pituitary gland. (COI:No)

### SP1-5

### Identification and characterization of novel markers for folliculo-stellate cells in the mouse pituitary gland

Momoka Tanaka[1], Konomi Takemoto[2], Ken Fujiwara[3], Junko Nio-Kobayashi[4] ([1]*Sch. Med., Hokkaido Univ.,* [2]*Hokkaido Univ. Hospital Clinical Training Center,* [3]*Dept. Biological Sciences, Fac. Sci., Kanagawa Univ.,* [4]*Lab. Histol. Cytol., Grad. Sch. Med., Hokkaido Univ*)

Folliculo-stellate (FS) cells in the pituitary gland are non-hormone-producing heterogenous cells and S100beta has been a major marker in rats. However, S100beta protein cannot be detected in FS cells of rats at embryonic and neonatal stages and of mice. We here revealed that aldolase C and 3-phosphoglycerate dehydrogenase (3PGDH) are differentially localized to FS cells in the pituitary gland of mice at adult and developmental stages.
Intense immunoreactivities for both aldolase C and 3PGDH were found in both stellate and follicular cells as well as marginal cell layer, whereas S100beta was limited to follicular cells in adult mouse pituitary gland. Certain populations of hormone-producing cells were weakly immunoreactive for 3PGDH, being identified as somatotrophs. Immunoreactivities for aldolase C and 3PGDH were slightly detected in the Rathke's pouch at embryonic day 13.5, and increased in both number and intensity at embryonic day 15.5. Intense immunoreactivities for aldolase C and 3PGDH were found in FS cells and marginal cell layer at neonatal day 1. These data suggest that both aldolase C and 3PGDH are localized to FS cells in mice, a former being more specific. (COI:No)

### SP1-6

### Three-Dimensional topography of the rat trigeminal ganglion neurons with combination of retrograde labeling and tissue clearing techniques

Makoto Fukushima[1], Eriko Kuramoto[1], Ryozo Sendo[1,2], Haruki Iwai[1], Atsushi Yamanaka[1], Mitsutaka Sugimura[2], Tetsuya Goto[1] ([1]*Dept. Oral Anatomy and Cell Biol., Grad. Sch. Med. Dent. Sci., Kagoshima Univ.* , [2]*Dept. Dental Anesthesiology, Grad. Sch. Med. Dent. Sci., Kagoshima Univ.* )

In the trigeminal ganglion (Tg), it is suggested that damaged neurons affect surrounding glial and/or neuronal cells via paracrine of inflammatory mediators and induce paresthesia in uninjured sites, and lead to heterotopic pain in the orofacial regions. Thus, for understanding of the mechanisms of heterotopic pain in the orofacial region, it is essential to precisely reveal somatotopic-organization of Tg neurons in three-dimension (3D). In the present study, we first optimized retrograde tracing technique with fast blue and tissue clearing techniques for the rat orofacial regions, and then revealed the 3D topography. Retrogradely labeled Tg neurons innervating 1st, 2nd and 3rd branch regions were distributed in separate areas of the Tg with considerable overlap. Especially, somata of Tg neurons innervating 2nd and 3rd branch regions, such as the maxillary and mandibular molar teeth, masseter muscle and tongue, were frequently located in adjacent each other. Since these oral regions are known to frequently occur heterotopic pain in clinical patients, the present results indicated that paracrine of inflammatory mediators in the Tg would cause heterotopic pain in the oral regions. (COI:No)

### SP1-7

### Aging affects the expression and distribution patterns of STB/HAP1 in mouse brain

Fuko Hamasaki[1], Kanako Nozaki[1], Ayumi Yasukochi[1], Md Nabiul Islam[1], Akie Yanai[2], Koh-hei Masumoto[1], Koh Shinoda[1] ([1]*Yamaguchi Univ., Div. Neuroanatomy,* [2]*Yamaguchi Univ., Dep. Basic Laboratory Science*)

Huntingtin associated protein 1(HAP1) is a neural huntingtin interactor, being considered as a core molecule of stigmoid body (STB). We have recently demonstrated *in vitro* that HAP1 prevents neuronal apoptosis induced by proteasome inhibitor MG132, which leads to form perikaryal reticulogranular clump (PRGC) besides STB. As proteasome activity gradually decreases during aging, changes in HAP1 expression levels and distribution patterns of STB/HAP1 may occur in aged mice. In the current study, we determined the effects of aging on the expression and distribution of HAP1 in Western blot and immunohistochemistry. Western blot analysis showed that HAP1 expression significantly decreased in aged brains compared to young ones. In addition, using immunohistochemistry, the number of STBs was also reduced in the aged brain. Furthermore, genetic downregulation of HAP1 in HAP1-hetero deleted mice confirmed the reduced HAP1-immunureactivity and decreased number of STBs. These findings suggest that vulnerability for neurodegenerative disorders in aged brain is attributable to the reduction of STB/HAP1-immunoreactivity. (COI:No)

### SP2-1

### Comparison of the distance from the puncture site to the superior gluteal nerve, artery and vein among three intramuscular gluteal injection sites.

Kana Kono[1], Masatoshi Komiyama[2] ([1]*Fuclt. Nurs., Chiba Univ.,* [2]*Grad. Sch. Nurs., Chiba Univ*)

The intramuscular gluteal injection has a risk of injuring nerves, arteries and veins. To assess the risk of such injury, distances from the puncture sites to branches of the superior gluteal nerve, artery and vain were compared among three intramuscular gluteal injection sites, the Clark's point (dorsogluteal site), the four- and three-way split point and the Hochstetter's area (ventrogluteal site). Three donated cadavers were used and right side of the gluteal area was dissected after puncturing the three sits with needles. The needles were found to prick the gluteus medius muscle directly at all the sites. The shortest distance from the needle to the branches on the surface of the gluteus medius muscle was 2.8 cm at the Hochstetter's area. The distances from the puncture sites to the branches running between the gluteus medius and minus muscles were less than 1.0 cm at the Clark's point as well as the Hochstetter's area. However, there was no significant difference among the three sites in one-way ANOVA. Thus, we could not determine which site is the safest. It is necessary to increase the sample size to assess the risk precisely. (COI:No)

### SP2-2

### A case of double inferior vena cava with inter-internal iliac veins

Shiori Yoshimura[1], Kentaro Yamamoto[1], Shintaro Fujimura[1], Shinichi Kawata[2], Takuya Omotehara[2], Kazuyuki Shimada[2], Masahiro Itoh[2] ([1]*Faculty of Medicine, Tokyo Medical University ,* [2]*Department of Anatomy, Tokyo Medical University*)

Double inferior vena cava (IVC) has been well reported as one of the venous congenital anomalies. In the present case, the IVC was located on the left side of the abdominal aorta, although the IVC was also on the right. The width of the right and left IVC at the L5 level was 11 mm and 7 mm, respectively. The left IVC crossed the abdominal aorta anteriorly at the L2 level and joined the right IVC at the L1 level. At each level, the renal veins on each side flowed into the each IVC. The lumbar veins at the L2 to L5 level entered each side of the IVC. No connection with the azygos and hemiazygos veins were confirmed. The internal and external iliac veins were merged at the S1 level on each side. Besides, a connecting branch (3mm) was found between the internal iliac veins near their origin. It had a further branch (1mm), which ran obliquely upward and reached the left inferior vena cava at the level of L5.

The inter-iliac vein has been reported in the double IVC cases, but the vein between the internal iliac veins has not yet been reported. The present case suggests that the inter-iliac veins could also occur between internal iliac veins in a double IVC case. (COI:No)

### SP2-3

### Autopsy imaging of cadaveric brain using magnetic resonance imaging

Momoka Otani[1], Takuya Asakawa[1], Kaoru Tsutsumi[1], Hitoshi Ueno[1], Kiichi Tadano[2], Tomoaki Yamamoto[2], George Matsumura[1], Miki Nagase[1] ([1]*Dept. Anatomy, Sch. Med., Kyorin Univ.,* [2]*Dept. Medical Radiological Technology, Sch. Health Sciences, Kyorin Univ*)

Autopsy imaging (Ai) (postmortem imaging) using X-ray computed tomograph (CT) is getting popular in dissection practice in Japan. Recently, our university began Ai using magnetic resonance (MR) imaging (MRI; Canon Vantage Titan 3T), in addition to CT (Canon Aquilion 16). The aim of the present study was to compare the postmortem MR images of the brain with postmortem CT and premortal ones, to examine factors influencing on MR signals, and to compare postmortem MR images with macroanatomical findings. We obtained high-resolution MR images (T1- and T2-weighted) of cadaveric brain, and contrast enhanced imaging was possible by infusing gadolinium acetate. The MR signals were affected by body temperature and formalin fixation. Major structures within the brain were still recognizable when excised formalin-fixed brain was analyzed, although signal intensities were greatly altered. In conclusion, we introduced postmortem MR imaging, and obtained high-resolution images of cadaveric brain without and with contrast enhancement. Linking MR images to dissected brain slices is useful for understanding three-dimensional structure of the brain. (COI:No)

### SP2-4

### Quantification of abdominal aortic tortuosity and examination of the causes of tortuosity

Atsushi Otani[1], Kazunobu Saiki[2], Keiko Ogami-Takamura[2], Daisuke Endo[2], Kiyohito Murai[2], Keishi Okamoto[2], Toshiyuki Tsurumoto[2] ([1]*Sch. of Med. Sci., Nagasaki Univ.,* [2]*Dep. Macroscopic Anatomy, Sch. of Biomed. Sci., Nagasaki Univ*)

Abdominal aortic tortuosity is an occasional case in the treatment of the aorta. We quantified the degree of abdominal aortic tortuosity (Tortuosity Index; TI) in CT images of 103 cadavers (female 52 cases, age; 66-98, average; 86.4: male 51 cases, age; 62-98, average; 81.2) and investigated the incidence of aortic tortuosity. The TI was calculated on the central coordinates of the aorta in 21 CT images by dividing the entire abdominal aorta length into 20 equal segments. The Calcification Index was also calculated from the ratio of calcification of the aortic wall in 21 CT images. Of 103 cases, 39 cases had a grossly visible aortic tortuosity with a TI of 1.07 or greater. The incidence of aortic tortuosity was significantly higher for females (26/52) than for males (13/51), and the mean of the TI was also significantly higher for females. The TI had a significantly positive correlation with age. It also had a significantly positively correlated with the calcification index. These results suggest that abdominal aortic tortuosity is associated with being female, older age, and more calcification. (COI:No)

### SP2-5

### Sex determination of the deficient Jomon human skulls with newly invented image process techniques on maxillary teeth

Tomohiro Tsuru[1], Koichi Tada[1], Wataru Yano[2], Akiyoshi Matsumura[3] ([1]*Med. National Medical College,* [2]*Biol. National Medical College,* [3]*Eng. Kanagawa Univ*)

The sexual identification in morphology of the excavated Jomon skull have been of essential for anthropological research. However, conventional morphometric methods are difficult to quantify the morphology of broken or partial bone specimens. We invented new techniques to analyze sex differences in 31 sex-identified Jomon skeletons (20 males and 11 females) from the University of Tokyo's Museum of Natural History by quantification of local features on medial incisor, canine, second premolar, and first molar. After contour lines of each tooth crown was detected using a series of image filters, they were subjected to a nonlinear diffusion filter to detect AKAZE features from the local ambient anisotropic luminance gradient. We counted for shared feature points with matching features between the test sample and the members of each sex population. The AKAZE feature points were distributed along ridge and occlusal cusps. The accuracy of sex determination was highest in the medial incisor, with more than 80%. (COI:Properly Declared)

### SP2-6

### The development of an original application for the human body anatomy course guide

Takumi Fujii[1], Shouichirou Kawauchi[1], Momoko Yanase[1], Kotone Uchiyama[1], Ryouta Koike[1], Kyouhei Yamashita[1], Naoki Abe[1], Kento Isoda[1], Kouhei Yamada[1], Natsuko Tashiro[1], Ririi Nomura[1], Michio Ono[2], Taketo Uji[2], Naohiro Hatori[2], Kazuyuki Ohbo[2] (*[1]Yokohama City Univ. Sch. Med., [2]Dept. Histology and Cell Biology, Yokohama City Univ. Sch. Med*)

<Background> Previously, we have edited original paper-based anatomy guide. Nowadays, COVID-19 decrease time for students to learn anatomy in face-to-face class, so more efficient educational tool is in demand.
<Purpose> We planned to develop an easy-to-understand and time-saving educational material in anatomy training.
<Method> We developed the original anatomy application for beginners available on tablets, referring to widely-used "LABORATORY MANUAL OF DISSECTION". We used easy explanations and corresponding illustrations and photographs. To keep students' motivation up, we wrote some columns of trivia of human body, connection of basic anatomical science between clinical medicine and so on. In a viewpoint of respecting human dignity, we limit the access to photographic contents only in the dissection room to avoid leaking the images to the Internet. In addition, we are planning to insert our original movies of how to dissect, which is the advantage of the electric guide.
<Conclusion> This application is going to be adopted in the anatomy training at Yokohama City University from next year. After receiving feedback, we will improve the quality of the application. (COI:No)

### SP2-7

### Gross anatomical and histological examination of the branches from the femoral nerve distributed near the patella

Mayu Hinada[1], Touma Sakuraya[2], Rintarou Yamamoto[2], Kenji Emura[3], Takamitsu Arakawa[2] (*[1]Fac. Health Sci., Sch Med., Kobe Univ., Hyogo, Japan, [2]Grad. Sch. Health Sci., Kobe Univ., Hyogo, Japan, [3]Fac. Health Care Sci., Himeji Dokkyo Univ*)

Sensory-related problems in the anterior part of knee joint, such as chronic pain and discomfort, often occur. However, the origin, course and distribution of the sensory nerve branches which are responsible to these problems of knee joint are still remained unclear. Nerve branches from the femoral nerve of 5 sides of 3 cadavers were examined these courses until near the patella macroscopically. Near the distribution, nerve branches were examined histologically in 2 sides. Some branches of the femoral nerve passed within the vastus lateralis and medialis, penetrated tendon-like structure proximal to the patella, then ran on the proximal surface of the patella. The anterior cutaneous branches of the femoral nerve ran on the surface of rectus femoris and vastus medialis, some branches of this nerve entered the tendon-like structure medial to the patella, and passed distally and laterally. These nerve fibers which distributed near the patella were unmyelinated nerve fibers histologically, suggesting that these could be related to pain sensation near the patella. (COI:No)

### SP3-1

### Gross anatomical study on the route of arteries within the transverse mesocolon

Tomokazu Okazaki[1], Takuya Omotehara[2], Shinichi Kawata[2], Masahiro Itoh[2] (*[1]Faculty of Medicine, Tokyo Medical University , [2]Department of Anatomy, Tokyo Medical University*)

**Introduction**
Previous studies on the arterial distribution to the transverse colon have focused only on the bifurcation of arteries. Due to a lack of the detailed studies on the positional relationship between the transverse colon and blood vessels, the pattern of the path of arteries in the transverse colon was investigated.
**Materials and methods**
The arteries in the transverse mesocolon ware dissected in cadavers at Tokyo Medical University, and their distance from the transverse colon was measured.
**Result**
The middle colic artery (MCA) entered the transverse mesocolon through the right half of the mesenteric root, and its branches directed toward the right and left side. In the left half of the mesentery, a left branch of the MCA ran along the transverse colon within 4 cm, as the marginal artery. In some cases, there was an artery, superior left colic artery (SLCA), crossing in more inner region of the mesentery than the marginal artery.
**Discussion**
The space without blood vessels is vaster on the left half of the transverse mesocolon compared to the right half, depending on the branching pattern of the MCA and SLCA. (COI:No)

### SP3-2

### Gross anatomical, histological, and immunohistochemical analyses of the recently-identified tubarial gland using human cadavers

Hiroki Takahata[1], Haruka Sugiyama[1], Kosuke Sasai[1], Saki Kamegai[1], George Matsumura[1], Miki Nagase[1] (*[1]Dept.Anatomy,Sch.Med.,Kyorin univ*)

In 2020, a new salivary gland named the tubarial gland was identified around the torus tubarius, based on the images of prostate-specific membrane antigen ligands (PSMA) PET/CT. The aim of the present study was to examine the presence, distribution, and characteristics of the tubarial gland, using human cadavers. The nasopharynx, the eustachian tube, and their surrounding tissues were dissected from formalin-fixed human cadavers. The mucosal surface was observed macroscopically and under stereomicroscope. Paraffin sections were prepared and stained with HE and PAS. Immunohistochemistry was performed to determine their characterization. Multiple openings were found on the mucosal surface of the torus tubarius, fossa of Rosenmüller, and nasopharyngeal wall. Histologically, glandular tissues were detected abundantly between the mucosal epithelium and cartilage of the eustachian tube, as well as in the submucosal layer of the nasopharyngeal wall. Most glandular cells contained PAS-positive mucin. In conclusion, the presence and distribution of the tubarial gland were demonstrated in the elderly cadavers. (COI:No)

### SP3-3

### Anatomical analysis of probable physical interaction between nerves and fasciae as a potential mechanism of low back pain

Izumi Kawarata[1], Ryoji Suzuki[1], Yshio Bando[1] (*[1]Grad.Sch.Med., Akita Univ., Dept. Anat*)

Some reports indicate 85% of the low back pain cases are undiagnosed. In this study, we examined probable fasciae compression and their effects on posterior rami of spinal nerve. Posterior rami of spinal nerves of male (age: 65) cadaver were track down from subcutaneous region to dorsal root ganglia (DRG) by dissection. They changed direction angle of around 110 degree at Thoracolumber fascia penetration sites. This observation indicates the possibility to cause fasciae compression. In addition, one of the L1 and the L2 nerves were found to share fascia penetration site. Therefore, the L1 nerve would have been subject greater forces. Since augmentation of satellite glial cells (SGCs) surrounding DRG neurons were formerly reported as an indicator of chronic pain (Shinder V., et al. 1999), sections of the L1 and the L2 DRGs were then histologically analyzed. Hematoxylin-eosin staining revealed that the L1 DRGs had more augmented SGCs than the L2 DRGs. Our findings suggest mechanical fasciae stress on the L1 nerve as a potential cause of low back pain. (COI:No)

### SP3-4

### Internal vibration sensation and its sensory receptors in the upper arm region

Yoshiyuki Onishi[1], Haruka Hisato[1], Seishi Maeda[2], Yusuke Minato[2], Sachi Otani[2], Hideshi Yagi[2] (*[1]Hyogo Col med, [2]Dept. Anat. Cell. Biol. Hyogo Col Med*)

The sensory system plays a role in detecting external and internal threats to the body. Pacinian corpuscles in the dermis detect vibrations related to mechanical stimulation of the skin. Reports have shown that Pacinian corpuscles are localized deep in the body, such as the pancreas, large nerves, large blood vessels, and lymph nodes. We recently reported that large sensory corpuscles that resemble Pacinian corpuscles are distributed in the vascular sheath of the femoral artery in humans. We investigated the localization and function of sensory corpuscles around the deep artery in the upper arm. Serial sectioning of the deep vessels and surrounding tissues revealed the detailed distribution of the sensory corpuscles. As the sensory corpuscles resemble Pacinian corpuscles that detect subtle stimulation, they are expected to detect vibration from the vessels. We sought to clarify this using physiological examination. (COI:Properly Declared)

### SP3-5

### Gross anatomical study in insertion of brachialis muscle and anterior surface of the articular capsule for the elbow joint

Fuki Yanagihara[1], Rintaro Yamamoto[2], Mizuki Izumida[2], Kenji Emura[3], Takamitsu Arakawa[2] (*[1]Fac. Health Sci., Sch Med., Kobe Univ., Hyogo, Japan, [2]Grad. Sch. Health Sci., Kobe Univ., Hyogo, Japan, [3]Fac. Health Care Sci., Himeji Dokkyo Univ*)

Although some candidate stabilizers of the anterior and lateral parts of the elbow joint have been well described by kinesiological and biomechanical studies, their anatomical basis are scarce. Brachialis muscle could attach to the anterior part of the elbow joint capsule, however, detailed insertion area including variations has not been well documented. Thus, 5 elbows of 3 cadavers were examined in detail to clarify the insertion site of brachialis. In all specimens, brachialis inserted onto the tuberosity of ulna. In 3 elbows, insertion site was spread the anterior surface of the elbow joint capsule and the anterior surface of annular ligament. In the same specimens aponeurotic expansions from brachialis tendon divided into two to envelop the insertion tendon of biceps brachii. These findings suggest that some insertion parts of brachialis could contribute to the brachioradial joint of the elbow and could provide tendon sheath-like structure for the biceps tendon. (COI:No)

### SP3-6

### Prevalence and distribution of colic diverticulum in cadavers donated for dissection practice

Maki Suzuki[1], Masatoshi Komiyama[2] (*[1]Fuclt. Nurs., Chiba Univ., [2]Grad. Sch. Nurs., Chiba Univ*)

Colic diverticulum is one of the risk factor of insufficient bowel preparation for colonoscopy. To investigate the prevalence and distribution of colic diverticulum, large intestines except the rectum were observed in 33 cadavers (20 males and 13 females) donated for dissection practice. Mean age of the cadavers was 87.2 ± 6.7 years. Diverticula were found in 16 (48.5%) cadavers (9 [45.0%] males and 7 [53.8%] females). There was no significant difference in prevalence of diverticulum between males and females. Mean number of diverticula per positive colon was 19.9 ± 19.8, ranging from 1 to 70. These diverticula were found in the cecum, ascending colon, transverse colon and descending and sigmoid colon, at a ratio of 0.6%, 31.8%, 0.9% and 66.7%, respectively. Many of them were observed between tenia libera and tenia mesocolica (38.1%) and between tenia mesocolica and tenia omentalis (58.8%), but only 3.1% was observed between tenia libera and tenia omentalis. These results suggest that about half of aged people have colic diverticula mainly in the ascending, descending and sigmoid colon, especially on its medial and dorsal surface. (COI:No)

### SP3-7

### Gross anatomical study of the tendon sheath for the flexor carpi radialis and lateral wall of the carpal tunnel

Chihiro Toma[1], Rintaro Yamamoto[2], Tohma Sakuraya[2], Mizuki Izumida[2], Kenji Emura[3], Takamitsu Arakawa[2] (*[1]Fac. Health Sci., Sch Med., Kobe Univ., Hyogo, Japan, [2]Grad. Sch. Health Sci., Kobe Univ., Hyogo, Japan, [3]Fac. Health Care Sci., Himeji Dokkyo Univ*)

The flexor carpi radialis muscle (FCR) inserts onto the base of the 2nd and 3rd metacarpals through the separate tendon sheath within the carpal tunnel. Detailed fibrous constitution and continuity of the FCR tendon sheath are remained unclear, and therefore, these structures were examined in detail macroscopically. Eight forearms of six cadavers were used. The FCR tendon ran through a groove dorsal to the tubercle of the trapezium and was covered by independent aponeurosis which spanned above the groove. The palmar intercarpal ligaments between the trapezium and trapezoid extended superficially along the ulnar side of the trapezium, then covered the independent aponeurosis and FCR tendon, and finally merged with the flexor retinaculum near the tubercle of trapezium. This finding suggests that the FCR tendon ran through independent tendon sheath that was distinguished by the carpal tunnel. The lateral wall of the carpal tunnel should be defined as the palmar extension of the palmar intercarpal ligaments, not the tendon sheath for the FCR tendon. (COI:No)

### SP4-1

### Pathologies of the trigeminal nervous system, and delayed masticatory rhythm in triple-transgenic mouse model of Alzheimer's disease

Ayano Kitawaki[1], Harukiyo Esaki[1], Hinano Oda[1], Kouki Katsuyama[1], Teruki Kinoshita[1], Megumi Susa[1], Masayoshi Taniguchi[1], Eriko Kuramoto[1], Mitsuru Saito[2], Hiroshi Kono[3], Takakazu Yagi[4], Shinei Matsumoto[5], Hiromitsu Hara[5], Haruki Iwai[1], Atsushi Yamanaka[1], Tetsuya Goto[1] (*[1]Dept. Oral Anatomy Cell Biol., Grad. Med. Dent. Sci., Kagoshima Univ., [2]Dept. Oral Physiol., Grad. Med. Dent. Sci., Kagoshima Univ., [3]Dept. Biomaterials Sci., Grad. Med. Dent. Sci., Kagoshima Univ., [4]Dept. Oral Health, Kobe Tokiwa Junior College, [5]Dept. Immunol.,Grad. Med. Dent. Sci., Kagoshima Univ*)

Alzheimer's disease (AD) patients have been reported to show impaired chewing function, but the underlying mechanism has been unknown. In the present study, by using triple-transgenic mouse model of AD (3xTg-AD), we immunohistochemically revealed the neuropathology of AD in the mesencephalic trigeminal nucleus (Vmes), which is essential for mastication, and electrophysiologically examined whether the AD pathology in the Vmes affected mastication. In 8-weeks-old 3xTg-AD male mice, the Vmes was immunopositive for amyloid β (Aβ). At 6-months-old, Aβ-immunoreactivity was also observed in the motor and sensory trigeminal nuclei, and further, phosphorylated tau was immunopositive in the Vmes. Electromyogram (EMG) of the masseter muscle during spontaneous mastication showed that the duration of muscle activity and the interval between muscle activities were significantly longer in 3xTg-AD mice than in control (C57BL/6J) mice. Interestingly, bite force per unit muscle activity of 3xTg-AD mice tended to be smaller than that of control mice. These results suggest that AD neuropathology occurs mainly in the Vmes, and it might cause delayed rhythm of mastication, and affect occlusal force. (COI:No)

### SP4-2

### Inhibition of HDAC increases BDNF expression and promotes neuronal rewiring and functional recovery after brain injury

Naoki Sada[1], Yuki Fujita[1,2], Toshihide Yamashita[1,2,3] (*[1]Department of Molecular Neuroscience, Graduate School of Medicine, Osaka University, [2]WPI Immunology Frontier Research Center, Osaka University, [3]Department of Neuro-Medical Science, Graduate School of Medicine, Osaka University*)

Brain injury causes serious motor, sensory, and cognitive disabilities. Accumulating evidence has demonstrated that histone deacetylase (HDAC) inhibitors exert neuroprotective effects against various insults to the central nervous system (CNS). In this study, we investigated the effects of the class I HDAC inhibition on the expression of brain-derived neurotrophic factor (BDNF) and functional recovery after traumatic brain injury (TBI) in mice. Knockdown of HDAC2 in cultured neurons increased the number of synaptic puncta. Administration of class I HDAC inhibitor CI-994 increased the number of synaptic boutons in rewiring corticospinal fibers and improved the recovery of motor functions after TBI. HDAC2 was mainly expressed in neurons and was increased in the premotor spinal interneurons, followed by decrease of BDNF expression in TBI. Knockdown of HDAC2 elevated H4K5ac enrichment at the BDNF promoter and increased BDNF expression after TBI. Together, our findings suggest that HDAC inhibition increases expression of neurotrophic factors, and promote neuronal rewiring and functional recovery following TBI. (COI:No)

### SP4-3

### Analysis of AIS length in 15q11-13 duplication associated ASD model mice

Yume Yamanaka[1], Sohei Katase[1], Yoshinori Otani[1], Masashi Fujitani[1] (*[1]Department of Anatomy and Neuroscience, Faculty of Medicine, Shimane University*)

Autism spectrum disorder (ASD) is a developmental disorder involving impairments in communication, reciprocal social interaction and restricted repetitive behaviors or interests. Duplication of the human chromosome 15q11-13 region is the most frequently seen chromosomal abnormality and a risk factor for the development of ASD. The axon initial segment (AIS) is located at the proximal axon and has a high density of ion channels, which occurs action potential initiation. In addition, the AIS regulates the excitability of neurons by changing the structures which include length and position. Many studies reported abnormalities in AIS are risk factors that cause various neurological diseases. This study showed analysis of abnormal neural circuits involved in ASD by using AIS. To address, we measured the length of AIS in pyramidal neuron in Layer 2/3 and 5 of medial prefrontal cortex (mPFC), primary motor (M1) and sensory (S1BF) cortex, and Purkinje cell of 15q11-13 duplication and wild type mice. As our preliminary data, there were differences in the length of AIS of neurons in the Layer 5 of mPFC, M1, S1BF cortex and Purkinje cells between 15q11-13 duplication and wild type mice. (COI:No)

### SP4-4

### *In vivo* analysis of food aversion associated with anticancer drug treatment

Tamon Shimizu[1], Yoshihisa Koyama[1,2], Shoichi Shimada[1,2] ([1]*Neuroscience and Cell Biology, Grad. Med, Osaka Univ., Osaka, Japan.,* [2]*ARU., OPRC., OPMC., Osaka, Japan*)

While anticancer drug treatment is excellent in killing cancer cells, it has the disadvantage that it easily affects normal cells that proliferate actively, such as intestines, skin, hair roots and bone marrow. The main side effects include nausea/vomiting, hair loss, myelosuppression and renal dysfunction etc. Among the side effects, food aversion adversely affects appetite and significantly impairs quality of life. However, since the detailed mechanism is unknown, there is no effective therapeutic agent. We conducted a taste aversion reflex experiment using mice to analyze the mechanism of food aversion in anticancer drug treatment. In this presentation, we will introduce the research results of the taste aversion experiment including brain analysis. (COI:No)

### SP4-5

### New treatment for interstitial pneumonia with new antioxidants

Masato Shimada[1], Yoshihisa Koyama[1,2], Yuki Kobayashi[3], Hikaru Kobayashi[3], Shoichi Shimada[1,2] ([1]*Neuroscience and Cell Biology, Grad. Med., Osaka Univ., Osaka, Japan,* [2]*ARU., OPRC., OMPC., Osaka, Japan,* [3]*ISIR, Osaka Univ, Osaka, Japan*)

Interstitial pneumonia are diseases that causes inflammation mainly in the interstitium of the lung and includes various types such as idiopathic pulmonary fibrosis. However, many of them have unknown causes and are difficult to treat, so there is an urgent need to develop therapeutic agents. Because lung fibrosis due to oxidative stress is one of the factors that worsen the prognosis, it is considered that the appropriate antioxidant may be an effective therapeutic agent.

Our silicon (Si)-based agent can neutralize active oxygen species by reacting with water to generate hydrogen, which is an antioxidant. As a result of morphological analysis using drug-induced interstitial pneumonia model mice using methotrexate, it was revealed that administration of Si-based agent alleviated inflammation and fibrosis of the lung interstitium and macrophage infiltration. From the above, Si-based agent will become an effective therapeutic agent for interstitial pneumonia. (COI:No)

### SP4-6

### Brain ischemia induces cellular and structural alterations in dura mater

Yuta Takahashi[1], Hiroyuki Konishi[2], Hiroshi Kiyama[2] ([1]*Dept. Anat., Nagoya Univ. Sch. Med., Nagoya, Japan,* [2] *Dept. Funct. Anat. & Neurosci., Nagoya Univ. Grad. Sch. Med., Nagoya, Japan*)

The meninges, which are composed of the dura, arachnoid and pia maters, contains abundant immune cells. Recent studies provided evidences that meningeal immunity affects brain functions; however, it remains unknown whether altered brain functions can modulate meningeal structure and/or immunity. In this study, we addressed this question by using a mouse model of brain ischemia. We found that dura mater underwent dramatic changes after middle cerebral artery occlusion. Dura mater became thickened coincidently with brain atrophy. Immunohistochemistry demonstrated that activated macrophages with hypertrophic morphology were massively accumulated in the thickened dura mater. Furthermore, we found that a bridge-like structure, which also contained a significant number of macrophages, was formed between dura mater and the surface of injured brain. These results indicate that ischemic brain damage could modulate structure and immune cell composition of dura mater, which may in turn affect fate of injured neurons. (COI:No)

### SP4-7

### Shooting vesicles dirived by endoplasmic reticulum fuse to plasma membrane in membrane repair

Yui Hirose[1], Katsuya Miyake[2] ([1]*Med, IUHW, Narita,* [2]*CBMR, IUHW, Narita*)

Plasma membrane disruptions are repaired by a mechanism involving $Ca^{2+}$-regulated exocytosis, but the origin of these vesicles have not been clarified. Here we have used to advantage a super-resolution system Zeiss Airyscan (Airyscan) to image the dynamics of endoplasmic reticulum (ER) which expressed Sec61beta-GFP in BS-C-1 cells during membrane repair of disruption created by the infrared laser of a two-photon. The tubular ER instantly collapsed into many vesicles at the disruption site. In the presence of extracellular calcium, the subdivided ER vesicles slowly fused with each other and returned to its original tubular ER. When observation was performed by Airyscan, a tube extending from the ER to the plasma membrane and the subsequent vesicle column were observed during membrane repair. Furthermore, we could observe slow vesicle fusion using Brefeldin A (BFA), which inhibits antegrade transport from the endoplasmic reticulum to the Golgi cis sac. Meteoric shower-like subdivided ER vesicles were observed in the repair membrane, suggesting that ER-derived membrane may be required for membrane repair. (COI:No)

### SP5-1

### Identification and Functional Analysis of Sulfatide Species in the Kidney

Keiko Nakashima[1], Yukie Hirahara[1], Taro Koike[1], Keizou Gamou[1], Susumu Tanaka[1], Souichi Oe[1], Chisato Oe[2], Takashi Yoshida[3], Masayuki Tsuda[5], Koichi Honke[4], Masaaki Kitada[1] ([1]*Department of Anatomy,Kansai Medical University,* [2]*Department of pathology and Laboratory Medicine,Kansai Medical University,* [3]*Department of Urology and Andrology,Kansai Medical University,* [4]*Department of Biochemistry,Kochi University Medical School,* [5]*Division of Laboratory Animal Science Research Center,Kochi University*)

In the kidney, sulfatide have diverse molecular species, but the physiological functions of specific sulfatide species have not yet been elucidated. The purpose of this study is to examine the distribution and their physiological functions. Using by imaging mass spectrometry seventeen major sulfatide species have been detected. The manner of how these sulfatide species are localized shown was divided into three patterns: one being confined in the outer medulla and a part of the cortex, another being in the collecting ducts, and the other being in the inner medulla only. Two sulfatide species with two hydroxyl groups on the ceramide moiety had in a collecting duct-specific localization. Electron microscopic analysis of the sulfatide-deficient mice showed abnormalities such as large vacuolar accumulations in the cytoplasm of intercalated cells of connecting tubules, whereas the proximal and distal tubules were unchanged. In an immunostaining analysis, accumulation of ATPases in intercalated cells was observed in the sulfatide-deficient mice, suggesting that these two intercalated cells-specific sulfatide species may play an important role in transporting renal materials. (COI:No)

### SP5-2

### Electron microscope findings of calcification-like structures in the spinal arachnoid mater

Yuliany Putri[1], Shinnosuke Yamada[1], Kyutaro Kawagishi[1], Katsuya Miyake[2] ([1]*Med, IUHW, Narita,* [2]*CBMR, IUHW, Narita*)

A number of white hardened depositions were found in a 65-year-old male cadaver during the opening of the spinal canal. The depositions were found under the spinal arachnoid mater from thoracic to lumbar level. Histological examination were proceed to make diagnosis. We thought that this might represent a case of Arachnoiditis Ossificans (AO) which was a term used to describe a partial ossification of spinal arachnoid mater. The process is said to be related to previous trauma or surgery which might lead to dural and arachnoid mater inflammation which eventually lead to ossification. However, we could not detect very much calcium in the samples by an EDS detector of a scanning election microscope. We would like to present electron microscopy findings and our hypothesis for the formation of calcification-like structures that might suggest relation to cell wound healing process mechanisms. (COI:No)

### SP5-3

### Molecular mechanism of fast motility of giraffe Kinesin revealed by cryo-EM

Yosuke Yamagishi[1], Tsuyoshi Imasaki[1], Yasushi Okada[2,3], Ryo Nitta[1] ([1]*Division of Structural Medicine and Anatomy, Kobe University School of Medicine.,* [2]*RIKEN Center for Biosystems Dynamics Research [BDR],* [3]*Graduate School of Science, Graduate School of Medicine, the University of Tokyo*)

Kinesins carry various cargoes such as synaptic vesicles or mitochondria along Microtubules (MTs) by using the energy from ATP hydrolysis. Kinesin family member 5A (KIF5A) is the most abundant kinesin in the axon and plays a crucial role in axonal transport. The MT-based motility of KIF5A is generated by the N-terminal motor domain and point mutations on this domain are known to cause some neurological disorders. However, detailed molecular mechanism of how KIF5A accomplish the effective motility are still under investigation. To understand the functional property of KIF5A motor domain, we focused on the giraffe KIF5A (gcKIF). GcKIF is a major kinesin for high-speed motility in the axon of vagus nerve. Using single-particle reconstruction of cryo-EM by Relion, we obtained the gcKIF-MT complex structure at 4.4 Å resolution. Comparison with the previously reported structures of human KIF5B revealed a significant decrease of the density around the loop L8, which is involved in the major MT binding sites of kinesin on MT. This suggests an increase of flexibility of L8, which may act as a suspension to smoothen the interaction with MTs, enabling high-speed movement of giraffe kinesin. (COI:No)

### SP5-4

### Morphological changes of primary cilia upon hypertonic shock

Hiroshi Otani[1], Ryota Nakazato[2], Koji Ikegami[2] ([1]*School of Medicine, Hiroshima Univerisity,* [2]*Anatomy and Developmental Biology Lab, Graduate School of Biomedical and Health Sciences, Hiroshima University*)

The primary cilium is a small protruding organelle known to function as a chemosensor or mechanosensor. Its deficiency in human bodies can cause a collection of diseases called ciliopathy. As clues of acquired deficiency of primary cilia that may cause ciliopathy remain to be elucidated, we aimed to investigate the possibility of the "acquired ciliopathy" by examining how they change upon environmental stresses. We herein focused on the kidney as it faces a wide range of osmolarity changes. We used mIMCD-3 cells derived from mouse intramedullary collecting duct and exposed them to different hypertonic conditions by adding NaCl to the isotonic medium. Primacy cilia shortened responding to the shock, and the number of primary cilia was decreased in a few hours. Taxol, which stabilizes microtubules by promoting the polymerization of it, neither blocked nor suppressed the loss and shortenings, indicating that microtubule disassembly itself is not involved in the hypertonic shock-induced loss and shortenings of the primary cilia. Microtubule-independent machineries seem to underlie the hypertonic shock-induced morphological changes of primary cilia, which we address in the future. (COI:No)

### SP5-5

### An analysis of homology between the lung and swim bladder by endodermal gene map of zebrafish

Rika Oshima[1,2], Norifumi Tatsumi[1], Shoko Himeiwa[1,3], Tatsuki Nagasawa[4], Tohru Yano[1], Masataka Okabe[1] ([1]*Department of Anatomy, Jikei University School of Medicine,* [2]*4th-year, Department of Medicine, Jikei University School of Medicine,* [3]*6th-year, Department of Medicine, Jikei University School of Medicine,* [4]*Department of Life Science and Technology, Tokyo Institute of Technology*)

The swim bladder has been known as an evolutionary modification of the lung. Recent studies of the swim bladder indicate that expressions of *BMPs* are altered, possibly leading to structural changes from the lung into the swim bladder. However, our previous anatomical analysis showed that the location of the pneumatic duct being connected to the gut was different from the location in the ray-finned fishes. Thus, this result casts doubt on the notion that the zebrafish swim bladder actually originated from the identical area to where the lung originates.

To answer this question, we made an endodermal gene map of zebrafish and presented it at the last annual meeting of JAA.

In our present presentation, we refined the gene map by adding new genes related to the development of the swim bladder including *BMPs* to the *Hox* genes. Moreover, *in situ* hybridization of paraffin sections was performed to reduce the background of *in situ* hybridization. This new zebrafish endodermal gene map will be, therefore, useful to clarify the exact location of development of the swim bladder in zebrafish and provide a clue for an evolutionary relationship between the lung and the swim bladder. (COI:No)

### SP5-6

### Cryo-EM structure of CAMSAP2 bound microtubule

Ryota Kitano[1], Tsuyoshi Imasaki[1], Hideki Shigematsu[2], Yosuke Yamagishi[1], Masatoshi Takeichi[3], Ryo Nitta[1] ([1]*Division of Structural Medicine and Anatomy, Department of Physiology and Cell Biology, Kobe University Graduate School of Medicine,* [2]*RIKEN SPring-8 Center,* [3]*RIKEN Center for Biosystems Dynamics Research*)

Microtubule is a tubular multi-protein complex composed of $\alpha$-, $\beta$-tubulin heterodimer, essential for the cell function through its dynamic structural rearrangement. Microtubule dynamics are strictly regulated by several microtubule association proteins (MAPs). Calmodulin-regulated spectrin-associated protein (CAMSAP) is a MAP which induces microtubule nucleation and polymerization to play the essential role in non-centrosomal microtubule network formation. CAMSAP2 forms an aster-like structure which serves as a nucleation center, resembling centrosomal aster in vitro. However, the molecular mechanism of how CAMSAP2 organizes tubulin to form microtubules are still unknown. Here we reconstituted CAMSAP2-microtubule complex in vitro and carried out high-resolution cryo-EM structural analysis. We have successfully determined near atomic resolution structure of the complex. Based on this structural analysis, I will discuss about the molecular mechanism of how CAMSAP2 regulates microtubule formation. (COI:No)

### SP5-7

### Verification of differences in ability of transcriptional activation of Gcm2 between fishes and tetrapods

Minori Takamura[1,2], Norifumi Tatsumi[2], Takanori Shono[2], Masataka Okabe[2] ([1]*3rd-year, Jikei University School of Medicine,* [2]*Department of Anatomy, Jikei University School of Medicine*)

The parathyroid gland is an endocrine organ that exists only in tetrapods but not in fishes. The transcription factor Glial Cell Missing-2 (Gcm2) is essential for not only the development of parathyroid glands but also the development of fish gills. It has been thought that the developmental program of gills has been changed evolutionarily to form parathyroid glands in tetrapods. However, it remains unclear what underlying developmental program(s) has been changed to produce parathyroid glands in tetrapods. To elucidate potential developmental changes, we have performed a series of experiments on transcriptional activities of Gcm2 in zebrafish and mice by analyzing Gcm reporter activity with HEK293 cells. The results showed that the binding activities to the reporter sequence were observed in Gcm2 of both zebrafish and mouse. However, only the Gcm2 containing the C-terminus of the mouse Gcm2 was discovered to activate the reporter in HEK293 cells. We hope that our finding may provide further clue for a developmental and evolutionary acquisition of parathyroid glands in tetrapods. (COI:No)

### SP6-1

### Functional analysis of microRNA-505 in glioma stem cells

Rio Kakizaki[1], Sumika Sakamoto[1], Souichi Oe[1], Masaaki Kitada[1] ([1]*Dept. Anat., Kansai Med. Univ.,*)

Glioblastoma multiforme (GBM) is the most frequently occurring primary brain tumor, and the existence of glioma stem cells (GSCs), which have the ability to form tumors, self-renew, and differentiate themselves, has been attracting attention as a cause of gliomas. Recently, two subtypes of GSCs are known to exist: proneural (PN) GSCs, which express a group of genes involved in neurogenesis, and mesenchymal (MES) GSCs, which have high tumorigenicity and are reported to be associated with poor prognosis. In this study, we identified microRNAs (miRNAs) specifically expressed in the MES GSCs and analyzed their functions to explore their potential as novel therapeutic targets. We have found that has-miR-505 is highly expressed in MES GSCs. To clarify its physiological significance, we identified target genes and investigated the changes in gene expression, proliferation, invasion, apoptosis, and drug resistance caused by inhibition of has-miR-505 function using a specific miRNA inhibitory oligonucleotide. (COI:No)

### SP6-2

### The chromatin remodeling factor BRM maintains the hematopoietic stem cell quiescence via TGF $\beta$ -Smad signaling.

Hiroki Miyachi[1], Hiroki Kiriyama[1], Naoki Itokawa[2], Motohiko Oshima[2], Toshio Suda[3], Atsushi Iwama[2], Ryo Nitta[1], Eriko Nitta[1] (*[1]Division of Structural Medicine, Department of Physiology and Cell Biology, Kobe University Graduate School of Medicine, [2]Division of Stem Cell and Molecular Medicine, Center for Stem Cell Biology and Regenerative Medicine, Institute of Medical Science, University of Tokyo, [3]Cancer Science Institute of Singapore*)

Current aspects support that most of the tissues are sustained by their innate stem cells. Recent observations shed light on the epigenetic systems as the great contributors to the decision of stem cell fates and their dysregulation leads to aging or the development of malignancies. Previous reports that the SWI/SNF ATP-dependent chromatin remodeling factor BRG1 maintains embryonic stem cell pluripotency lead us to its roles in hematopoietic stem cells (HSCs), which is one of the most well-studied tissue stem cells.
We first revealed that BRM, the homologue of BRG1, was expressed in stem cell specific manner rather than BRG1, therefore we assumed BRM as a critical piece for HSC stemness.
We next evaluated the role of BRM in HSCs with genetically modified BRM-null mice. BRM-deficient HSCs showed impaired reconstitution capacity in serial bone marrow transplantation, suggesting that BRM is essential to long-term repopulation of HSCs.
Finally, we identified the target genes of BRM by executing ChIP-sequences. BRM target genes are subject to repressive histone modifications and the regulatory element Smads, which may cooperate with TGF-beta signaling to maintain HSC quiescence. (COI:No)

### SP6-3

### Functional analysis of lncRNA MANCR in glioma stem cells

Sumika Sakamoto[1], Rio Kakizaki[1], Souichi Oe[1], Masaaki Kitada[1] (*[1]Dept. Anat., Kansai Med. Univ., Osaka, Japan*)

Glioblastoma multiforme (GBM) is the most frequently occurring primary brain tumor. In recent years, the existence of glioma stem cells (GSCs), which are capable of tumorigenesis, self-renewal, and multiple differentiation, has attracted much attention as a cause of GBM. In addition, there are several subtypes of GSCs, proneural (PN) GSCs expressing genes involved in neurogenesis and mesenchymal (MES) GSCs with high tumorigenic potential. We aim to elucidate the molecular mechanism of proneural-to-mesenchymal transition (PMT) by focusing on long non-coding RNAs (lncRNAs), which are involved in nuclear structure maintenance, transcriptional regulation, and RNA stability. We have identified several lncRNAs that are expressed in a subtype-specific manner by microarray analysis and real-time PCR. In this study, we focused on MES GSC-specific lncRNA, mitotically associated long non-coding RNA (MANCR), and analyzed the changes in gene expression, proliferation, invasion, apoptosis, and drug resistance caused by the lncRNA knockdown. (COI:No)

### SP6-4

### Gene expression evaluation in Muse cells by single-cell RNA sequencing analysis

Yo Oguma[1], Yasumasa Kuroda[1], Shohei Wakao[1], Mari Dezawa[1] (*[1]Department of Stem Cell Biology and Histology, Tohoku University Graduate School of Medicine*)

**Background and purpose**: MSC (Mesenchymal Stem Cell) are somatic stem cells that exist in mesenchymal tissues and include pluripotent stem cells, SSEA-3(+) Muse cells in a few percent, which differentiate into all three germ layers. Whereas, MSC other than Muse cells, SSEA-3(-) nonMuse cells do not differentiate into other lineage cells. (Stem Cell Dev. 2015) Although there are significant differences between Muse and nonMuse cells, its analysis has been limited to specific functions. In this study, we analyzed gene expression in Muse cells comprehensively by single-cell RNA sequencing(scRNAseq).
**Materials and methods**: BM-derived MSC were separated into Muse and nonMuse cells and performed scRNAseq. Expression variation genes were detected and subjected to GO analysis to estimate gene function.
**Result**: Gene expression of RNA splicing, ribosomal RNA, and electron transfer chain was higher in Muse cells than in nonMuse cells, and gene expression of MHC type2 and lysosome was lower.
**Conclusion**: The difference in metabolism and translation related gene expression suggest that MSC is the heterogeneous cell group constituted from a few Muse cells and the other cells. (COI:Properly Declared)

### SP6-5

### Unique characteristics of human and mouse amniotic membrane-derived Muse cells, non-tumorigenic pluripotent stem cells

Eiji Ogawa[1], Yoshihiro Kushida[1], Shohei Wakao[1], Kana Okawa[1], Yasumasa Kuroda[1], Mari Dezawa[1] (*[1]Tohoku University Graduate School of Medicine, Department of Stem Cell Biology and Histology*)

We newly found multilineage-differentiating stress-enduring (Muse) cells, endogenous non-tumorigenic pluripotent stem cells that can be identified as pluripotent surface marker SSEA-3(+), in human- and mouse-amniotic membrane as well as in mouse-placenta. SSEA-3(+)-Muse cells were collected as several percent of MSCs established from human/mouse-amniotic membrane and mouse-placenta. Similar to previous reports in the bone marrow and adipose tissues, we found that human/mouse- amniotic- and placental-Muse cells spontaneously generated cells representative of all three germ layers from a single cell. When compared with non-Muse cells in the human-amniotic membrane, Muse cells exhibited higher expression levels of pluripotency and extraembryonic markers. The same trend was seen when compared with bone marrow-Muse cells. Human-amniotic-Muse cells expressed markers relevant to germ-line stem cells when treated with a set of cytokines, suggesting the 'totipotency' for their differentiation not only into triploblastic cells but also into germ-line lineage cells. Since the amnion and placenta are easy accessible, their potential for clinical application is expected. (COI:Properly Declared)

### SP6-6

### Role of SNX25 in proinflammatory cytokine expression via NF- $\kappa$ B signaling

Kazuya Nishimura[1], Tatsuhide Tanaka[1], Syoko Takemura[1], Kouko Tatsumi[1], Akio Wanaka[1] (*[1]Dept. Ana. Neurosci. Nara. Medical. Univ*)

SNXs(Sorting Nexin) play key roles in membrane trafficking, cell signaling, membrane remodeling, and organelle motility and some of them are involved in the immune system. SNX25 belongs to the SNX family and negatively regulates TGF- $\beta$ signaling, which is an important component of the immune system. However, little else is known about the relationship between SNX25 and the immune system.
In the present study, we examined whether SNX25 modulates responses of the macrophage cell line RAW 264.7 to LPS stimulation by knocking down SNX25 expression with specific siRNA. SNX25 knockdown increased proinflammatory cytokine expression by activating the NF- $\kappa$ B pathway via ubiquitination of I $\kappa$ B $\alpha$ , an inhibitor of NF- $\kappa$ B. These findings reveal that SNX25 is an important regulator of the TLR4 signaling pathway. (COI:No)

### SP6-7

### Protective effect of HAP1 against the neuronal apoptosisunder the several adverse stresses.

Hazuki Tanaka[1], Kanako Nozaki[1], Fuko Hamasaki[1], Islam Md Nabiul[1], Akie Yanai[1], Koh-hei Masumoto[1], Koh Shinoda[1] (*[1]Division of Neuroanatomy, Yamaguchi University School of Medicine*)

Huntingtin-associated protein 1 (HAP1) as a core molecule of the stigmoid body (STB) and has protective functions against neurodegeneration. There are two HAP1 isoforms, HAP1A and HAP1B with different lengthsequence. HAP1A forms STBs intracellularly, while HAP1B is diffusely localized in the cytoplasm. We previously reported that HAP1A transfection could protect apoptosis induced by proteasome inhibition. On the other hand, it is not clear whether HAP1B has protective effects or not under certain stresses. In the present study, we investigated the effects of HAP1 on apoptosis induced by proteasome inhibition stress and endoplasmic reticulum (ER) stress. During proteasome inhibition stress, neuroprotective effect of HAP1A on the apoptosis were observed, whereas the apoptosis was not significantly protected in the HAP1B-transfected cells. Under ER stress condition, intriguingly, neuronal apoptosis was significantly inhibited in the HAP1B-transfected but not in the HAP1A-transfected cells. These suggest that HAP1 protects against apoptosis induced by different kind ofstresses (such as proteasome inhibition, ER stress), and the protection functions of HAP1 is isoform dependent. (COI:No)

### SP7-1

### Defective foliation and abnormal positioning of Purkinje cells occur when Dab1 is knocked out in postnatal mouse cerebellum

Takuto Kasai[1], Takao Honda[1], Kazunori Nakajima[1] ([1]*Keio. univ. sch. med, Anatomy*)

Reelin signaling pathway involving Dab1 is known to be essential for cerebellar development, because defect in this pathway causes severe cerebellar hypoplasia and abnormal Purkinje cell (PC) migration during embryonic stage. It is thus unclear whether the defective cerebellar foliation, observed postnatally in Reelin-signaling deficient mice, is caused directly by the postnatal defect in the Reelin signaling or caused secondarily to the failure in PC migration. To address this issue, we injected adeno-associated virus (AAV) expressing Cre into the *dab1*-floxed mice at postnatal day 1 (P1), when PC migration had already completed, and fixed the mice at P7-14. Infection of AAVs was mainly observed in PCs, which is a major cell type expressing the Reelin receptor VLDLR in cerebellum. Cerebellar foliation became significantly shallower for secondary folding compared to wild type, accompanied by disturbed layering of PCs. Furthermore, although *dab1* was mainly knocked out in PCs, reduction of Bergmann glial processes was observed. These results suggest that Reelin might control cerebellar folding postnatally through maintaining the PC positioning and Bergmann glial processes. (COI:No)

### SP7-2

### Sulfatide species during Schwann cell maturation in dorsal root ganglion

Keizo Gamo[1], Yukie Hirahara[1], Taro Koike[1], Souichi Oe[1], Katsuhiko Ono[2], Masaaki Kitada[1] ([1]*Dept. of Anatomy, Kansai Medical Univ.,* [2]*Dept. of Biology, Kyoto Prefectural University of Medicine*)

Sulfatide is a sphingolipid abundant in myelin in the nervous system. We previously reported the variation of sulfatide species during oligodendrocyte maturation and myelination processes. However, sulfatide synthesis in peripheral nerves system has not been examined. To analyze examine the appearance of sulfatide species in Schwann cell maturation at embryo stage, we examined cervical dorsal root ganglion (DRG) of chick and mouse embryos. DRGs in those embryos, four sulfatide molecular species were identified by imaging mass spectrometry. In adult mouse DRG, all four sulfatides were expressed in the fiber-rich areas, but only two species with short fatty acid among them were also localized in the neuronal cell body-rich areas. Moreover, immunohistochemistry analysis showed that Sox2 and Sox10, which were markers of immature Schwann cells, and sulfatides were colocalized in the early stages of the DRG maturation process. These observations suggest that the expression of sulfatide molecular species is maintained throughout life and the existence of molecular species with different roles than those with a myeline-specific role. (COI:No)

### SP7-3

### Plasma corticosterone levels during developmental stage in BTBR mouse model of autism spectrum disorder

Sayaka Goto[1], Nozomi Endo[1], Mayumi Nishi[1] ([1]*Nara Medical University, Department of Anatomy and Cell Biology*)

The autism spectrum disorder (ASD) is a neurodevelopmental disorder characterized by communication impairments and repetitive behaviors. It is known that ASD patients have altered stress response and dysregulation of the hypothalamic-pituitary-adrenal axis. BTBR $T^{+}$ $Itpr3^{tf}$/J (BTBR) mice have widely been used as a model of ASD. Previous reports showed an exaggerated peak corticosterone (CORT) concentration to stress stimuli in adult mice, but the stress response in developmental stage remains unknown. Considering that ASD is diagnosed in childhood, developmental stage analysis is necessary for the understanding of pathophysiology and improvement of therapy. In this study, we found that there was no difference in basal plasma CORT level between C57BL/6J (B6) and BTBR mice at P7, 14 and 21. Interestingly, after 30 minutes of acute restrained stress, the BTBR mice at P21 showed a significant increase in the CORT level compared to B6 mice. These results suggest that the stress response of juvenile BTBR mice is dysregulated. In the future studies, the detailed biological mechanism needs to be examined. (COI:No)

### SP7-4

### Studying the developmental process of lateral rectus muscle in human embryos with holoprosencephaly

Yasushi Okochi[1,2], Yutaka Yamaguchi[2], Shigehito Yamada[2] ([1]*Faculty of Medicine, Kyoto Univ.,* [2]*Congenital Anomaly Research Center, Kyoto University Graduate School of Medicine*)

Although the developmental origin of the extraocular muscles (EOM) has been widely studied, the mechanism of complex spatial organization of EOMs remains elusive. Here, we examined the migration of the primordia of EOMs to the eye through performing histological examination of human embryos. Previous studies have revealed that in the elasmobranchs, the primordia of lateral rectus (LR) muscle are innervated by the abducens nerve before it migrates to the eye. However, we showed that the primordia of human LR muscle is able to migrate without being innervated by the abducens nerve. Thereafter, we examined the abducens nerve in embryos with holoprosencephaly, and showed that the primordia of LR muscle are able to independently migrate to the eye from the primordia of other EOMs. In conclusion, these results suggest that the spatial organization of human LR muscle is established before the primordia of this muscle interacts with the eye. Furthermore, given the developmental similarity to the chick LR muscle, the developmental process in EOMs of amniotes is considered to be conserved in human embryos, which is the only placental mammal possessing head cavities during embryogenesis. (COI:No)

### SP7-5

### Analyses of migration profiles of the claustral neurons during development

Kota Oshima[1], Satoshi Yoshinaga[1], Ayako Kitazawa[1], Kazunori Nakajima[1], Ken-ichiro Kubo[1,2] ([1]*Department of Anatomy, Keio University School of Medicine,* [2]*Department of Anatomy, The Jikei University School of Medicine*)

The claustrum is a thin sheet of neurons located between the insular cortex and the striatum. Many studies have shown that the claustrum plays important roles in higher brain functions. Additionally, there is growing evidence that dysfunctions of the claustrum are associated with neuropsychological symptoms. However, analyses of the development of the claustrum have not been performed extensively. In the present study, we tried to analyze the development of the mouse claustrum, especially focusing on elucidating migration profiles of the claustral neurons. First, we determined the birthdate of the claustral neurons using bromodeoxyuridine (BrdU) labeling. Next, we analyzed migration profiles of the claustral neurons by taking advantage of the FlashTag technology, in which fluorescent dyes were injected into the ventricle of the developing forebrains. Our analyses showed that the claustral neurons were mainly generated between embryonic day (E) 10.5 and E12.5 and that some claustral neurons migrated radially outward and then inward after they reached the surface. Moreover, we confirmed these unique migration profiles by performing time-lapse imaging of GFP-labeled cells. (COI:No)

### SP7-6

### Pg-OMVs are involved in the development of placenta and transmission of bacterial virulence factors.

Airi Tanai[1], Yoko Fukuhara[1], Weng Yao[1], Mika Ikegame[1], Hirohiko Okamura[1] ([1]*Department of Oral Morphology, Okayama University Graduate School of Medicine, Dentistry, and Pharmaceutical Sciences, Okayama, Japan*)

The placenta is a vital organ, in charge of nutrient and gas exchange between the fetus and mother. Thus, the impairment of the placenta leads to maternal disabilities as well as developmental abnormalities to the fetus. Recent reports show that periodontitis leads to increased risk of maternal and fetus impairments. We have previously shown that outer membrane vesicles (OMVs) released from periodontopathic bacteria, *Porphyromonas gingivalis* (Pg), include virulence factors and travel to distant organs such as the lung, kidney, and brain. In this study, we examined the effect of Pg-OMVs on human placental trophoblast cells (BeWo). Periodontal model mice administered with Pg-OMVs showed significant decrease in the morphological size of the placenta and the fetus. In in vitro study, Pg-OMVs entered the BeWo cells and resided in the intracellular region. Pg-OMVs effectively transferred bacterial virulence factor, *Gingipain* (RgpA, KgpA), to BeWo cells without cellular damage. Our results indicate that Pg-OMVs affect the placenta and fetal development, at least in part, through the transmission of its virulence factors to trophoblasts.

Unregistered co-author: Hotaka Kawai, Okayama Univ. (COI:No)

### SP7-7

### Decreased proliferation in the neurogenic niche, disorganized neuroblast migration, and increased oligodendrogenesis in adult netrin-5-deficient mice

Shunsuke Ikegaya[1], Yurika Iga[1], Sumiko Mikawa[1], Li Zhou[2,3], Manabu Abe[2,4], Kenji Sakimura[2,4], Kohji Sato[1], Satoru Yamagishi[1] (*[1]Department of Organ and Tissue Anatomy, Hamamatsu University School of Medicine, [2]Department of Cellular Neurobiology, Brain Research Institute, Niigata University, [3]Center for Coordination of Research Facilities, Institute for Research Promotion, Niigata University, [4]Department of Animal Model Development, Brain Research Institute, Niigata University*)

In the adult mouse brain, neurogenesis occurs mainly in the ventricular-subventricular zone (V-SVZ) and the subgranular zone (SGZ) of the hippocampal dentate gyrus. We previously showed that netrin-5 (NTN5) is expressed in transit-amplifying cells and neuroblasts in the V-SVZ and SGZ of the mouse brain (Yamagishi et al., Front Cell Neurosci, 2015). However, the precise role of NTN5 has not been investigated. In this study, we show that proliferation in the neurogenic niche is impaired in NTN5 knockout mice. The number of proliferating (EdU-labeled) cells in NTN5 KO mice was significantly lower in the V-SVZ. By contrast, the numbers of EdU-labeled cells in the cortex, basal ganglia/lateral septal nucleus, and corpus callosum/anterior commissure were increased, which largely represented oligodendrocyte lineage cells. Lastly, we found that chain migration in the rostral migratory stream (RMS) of NTN5 KO mice was disorganized. These findings suggest that NTN5 may play important roles in promoting proliferation in the V-SVZ niche, organizing proper chain migration in the RMS, and suppressing oligodendrogenesis in the brain (Ikegaya et al., Front Neurosci, 2020). (COI:No)

### SP8-1

### Expression of CD38 in the vascular endothelial cells of the mouse pituitary gland

Ryo Sakaga[1], Yasuko Kitao[1], Yasuhiko Yamamoto[2], Osamu Hori[1] (*[1]Department of Neuroanatomy, Graduate School of Medical Sciences, Kanazawa University, [2]Department of Biochemistry and Molecular Vascular Biology, Graduate School of Medical Sciences, Kanazawa University*)

CD38 is a multifunctional protein with an ADP-ribosyl cyclase activity which produces cyclic ADP-ribose (cADPR) from nicotinamide adenine dinucleotide ($NAD^+$). In the brain, CD38 is highly expressed in astrocytes and, to a lesser extent, in neurons. In the present study, we examined the expression of CD38 in the mouse pituitary gland which has characteristics of both endocrine and nervous systems. Immunohistochemistry and western blotting revealed that CD38 is highly expressed in the vascular endothelial cells of the pituitary gland. Analysis using systemic and endothelial cell-specific CD38 knockout (KO) mice revealed that NAD concentration tended to be higher in CD38 KO pituitary. Although the expression pattern of CD31, a marker of vascular endothelial cells, was similar in both genotypes, that of GFAP, a marker of the astrocytes/pituicytes, was lower in the posterior lobes of CD38 KO pituitary gland. These results suggest that CD38 may contribute to the maintenance of local microenvironments around the vessels in the pituitary gland. (COI:No)

### SP8-2

### Molecular pathological analyses of calcified atherosclerosis in *Apoe*-knockout mice

Ryo Takahashi[1], Masa-Aki Shibata[1], Asuka Takei[2], Chinatsu Shiraoka[1], Azumi Hirata[1], Yoichi Kondo[1] (*[1]Department of Anatomy & Cell Biology,, Osaka Medical College, [2]Department of Plastic and Reconstructive Surgery, Osaka Medical College*)

Plaque calcification is frequently observed as an advanced atherosclerotic lesion. Therefore, understanding the mechanism of the calcification is clinically important. **Methods:** Normal aorta and specific atherosclerosis areas from formalin-fixed paraffin-embedded sections were obtained by a laser microdissection. RNA extraction and cDNA conversion were conducted, and *Runx2*, an osteoblast differentiation factor, was analyzed by a real-time RT-PCR. In addition, immunofluorescence staining was performed for osteogenic markers. **Results:** In real-time RT-PCR analysis, relative levels of *Runx2* were significantly elevated in atherosclerotic lesions compared with those of normal aorta. Immunofluorescence staining showed many osteoblasts expressing RUNX2 in early and progressive lesions. In contrast, osteoblasts in the calcified lesions expressed RUNX2 only weakly and Osterix intensely. **Conclusions:** Mechanism of the calcification in atherosclerosis of *Apoe*-KO mice may be closely related to osteogenesis. (COI:No)

### SP8-3

### Intracellular localization of dysferlin in skeletal muscle and requirement of the intracellular vesicle fusion in membrane repair

Takeshi Matsuda[1], Daiki Hakamata[1], Masateru Sato[1], Katsuya Miyake[2] (*[1]PT, IUHW, Narita, [2]CBMS, IUHW, Narita*)

Repair or die. Plasma-membrane disruption is a normal event in the life of many cells —for example, mammalian skeletal and cardiac muscle cells, which reside in mechanically active environments where the disruption frequency is directly dependent on the level of physical activity. Dysferlin is involved in membrane repair, but the mechanism is not clear yet. Here we have used to advantage a highly sensitive detector GaAsP and Ziess Airyscan detector(Airyscan) to image the dynamics of dysferlin-GFP during membrane repair of disruption created by the infrared laser of a two-photon microscope in mouse flexor digitorum brevis muscle. We also observed the vesicle fusions and the T-tubule dynamics during sarcolemma resealing using FM dye. The detailed localization of dysferlin was observed using an Airyscan and an electron microscope. Vesicle accumulation occurred at the laser-disruption site in membrane repair. Dysferlin was also accumulated as many vesicles at the disruption site. The Airyscan also showed that dysferlin localized in the sarcoplasmic reticulum or the T-tubule structures. (COI:No)

### SP8-4

### Immunolocalization of podoplanin and PHOSPHO1 in murine femora with intermittent administration of parathyroid hormone

Yuhi Nakajima[1,2], Tomomaya Yamamoto[2,3], Hiromi Hongo[2], Norio Amizuka[2], Tomoka Hasegawa[2] (*[1]School of Dental Medicine, Hokkaido University, [2]Developmental Biology of Hard Tissue of Dental Medicine, Hokkaido University, [3]Northern Army Medical Unit, Camp Makomanai, Japan Ground Self-Defense Forces, Sapporo, Japan*)

Podoplanin is a hallmark of the early stage of osteocytes and osteoblasts about to differentiate into osteocytes. PHOSPHO1 is a phosphoethanolamine/phosphocholine phosphatase in a matrix vesicle – an osteoblast-derived extracellular vesicle initiating bone mineralization. We have examined immunolocalization of podoplanin (osteoblasts differentiating into osteocytes) and PHOSPHO1 (mineralization) in murine femora with intermittent administration of parathyroid hormone (PTH), which has reported to increase bone volume by high bone turnover (frequent bone remodeling). Many podoplanin-positive osteoblasts were observed in the PTH administered bone, implicating accelerated osteoblast differentiation into osteocytes. However, there were fragmented lines and lines with long spans of PHOSPHO1-positive osteoblasts in the PTH-treated bone, indicating that some region is highly remodeled, while the others induce the continuous bone formation by means of minimodeling. Summarizing, intermittent PTH administration may stimulate osteoblast differentiation into osteocytes, according to facilitated bone formation, which is unevenly caused by bone remodeling and minimodeling. (COI:No)

### SP8-5

### Significance of aquaporin localization in human glymphatic system

Kota Tanaka[1] (*[1]Hirosaki Univ. Med*)

Recent studies have raised a possible role of astrocytes in regulation of cerebral fluid flow. This system in which cerebrospinal fluid drain prepared by astrocytes is currently "Glymphatic system". The glymphatic system is presumably associated with expression of water channel protein, aquaporin, in astrocytes. However, poor information is available regarding localization of AQP4, especially in human brain. In this study, to elucidate anatomical composition of glymphatic system, we investigated immunolocalization of the aquaporins with the distribution of astrocytes on tissue samples of human brain collected from cadavers with no diagnoses of neurological and psychological disorders. Three coronal slices, each of which included frontal, parietal and posterior lobe, were generally obtained from each cerebrum and processed for tissue preparation. Every cerebral lobe displayed higher area density of AQP4-immunopositive cells on ventral side than on dorsal side, and those densities became higher in posterior lobe. These findings suggest that tissue fluid clearance might be preserved towards caudal region in gerontological cerebrum. (COI:No)

### SP8-6

### Effects of light source wavelength on vascular enhancement imaging.

Yuki Naya[1,2], Hiroki Takanari[2] ([1]*The University of Tokushima Faculty of Science and Technology Department of Science and Technology*, [2]*The University of Tokushima. Institute of Post-LED Photonics*)

In recent years, technology development to visualize living organs non-labeled and non-invasively has been developed. In this study, we examined the effects of wavelength on the analysis images in the observation of blood vessels with different narrow-band light sources.
Light sources of three different wavelengths (400, 440, and 520 nm) with 10-nm linewidth were irradiated to the mouse ears sequentially, and images were recorded with a CMOS camera. The images were analyzed with Image J software to compare the sharpness of the vascular edges and the contrast inside the vessels.
When compared with the monochromatic light images, the sharpness of the blood vessels was highest at a wavelength of 400 nm. Comparing the composite images, the combination of 520-nm green light and 400-nm blue light resulted in the highest sharpness, while combination of 520-nm green light and 440-nm blue light resulted in the highest contrast.
The results suggested that 400-nm blue light would be better for accurately determining the shape of capillaries, while combination of 520-nm green light and 440-nm blue light would be better for applications where depth information is obtained from contrast changes.
(COI:No)

### SP8-7

### Expression and neurochemical characterization of HAP1 in the lingual ganglia of the mouse tongue

Yurie Hiwaki[1], Islam Md Nabiul[1], Tarif Abu Md Mamun[1], Kanako Nozaki[1], Koh-hei Masumoto[1], Akie Yanai[1], Koh Shinoda[1] ([1]*Yamaguchi University Graduate School of Medicine, Division of Neuroanatomy*)

Huntingtinassociated protein 1 (HAP1) is a polyglutamine lengthdependent interactor with causal agents in several neurodegenerative diseases and considered as a marker of stigmoid body. We have clarified that HAP1 is highly expressed in excitatory and inhibitory motor neurons in the Auerbach's plexus of enteric nervous system. It is possible that HAP1 might also be present in the lingual ganglia as tongue is continuation of gastrointestinal tract. To date, the expression of HAP1 and its neurochemical characterization have never been examined in the tongue. In the current study, we clarified that HAP1 was intensely expressed in the intralingual ganglia (ILG) and in the ganglia near the root of tongue (we coined as lingual root ganglion; LRG). The expression of HAP1 was substantially higher in the ILG of P0 mice than that in embryonic/adult mice. The majority of the ILG and LRG cells showed intense coexpression of HAP1 with ChAT and NOS. In addition, almost all the HAP1immunoreactive cells were coexpressed with calretinin, calbindin SP, and VIP in both ILG and LRG. These suggests that HAP1 might play a vital role in physiological functions of the tongue such as sucking behavior. (COI:No)

### SP9-1

### A chemogenetic approach to identify brain regions regulating colorectal motility in rat

Tomoya Sawamura[1], Kazuhiro Horii[2], Takahiko Shiina[2], Hiroshi Yamaguchi[3], Akihiro Yamanaka[3], Yasutake Shimizu[2] ([1]*Lab vet Physiol,Fac Appl Biol Sci,Gifu Univ,Gifu,Japan*, [2]*Lab Vet Physiol, Unit Grad Sch Vet Sci, Gifu Univ, Gifu, Japan*, [3]*Dept Neurosci II, Res Inst Environ Med, Nagoya Univ, Nagoya, Japan*)

We previously demonstrated injection of dopamine and 5-HT into the L6-S1 spinal cord enhance colorectal motility in rats. In this study, we aimed to identify brain regions regulating colorectal motility by using DREADD system. Adeno-associated virus (AAV) encoding Cre recombinase was injected into the L6-S1 spinal cord in male SD rats. Another AAV encoding hM3Gq was injected into the A11 region ormedullary raphe nuclei. Colorectal motility was assessed in vivo in anesthetized rats. CNO injection had no effect on colorectal motility in rat expressing hM3Gq in A11 region. However, when GABAergic inhibitor was preinjected into the L6-S1 spinal cord, colorectal motility was enhanced in response to CNO. The CNO-induced enhancement of motility was blocked by dopaminergic inhibitor injected into the spinal cord. Similarly, in rats expressing hM3Gq in raphe nuclei, CNO injection enhanced colorectal motility in the presence of prior spinal injection of GABAergic inhibitor. Our results show that monoaminergic neurons projecting from the raphe nuclei or A11 region to the spinal defecation center enhance colorectal motility, whereas GABAergic neurons have a negative impact on these pathways. (COI:No)

### SP9-2

### Dynamic changes in GAD65 expression in PTZ kindling rats

Yuki Fukuda[1], Yuki Kajita[1], Takanori Oyanagi[1], Riho Kawamatsu[1], Hajime Mushiake[1] ([1]*Dept Physiol, Grad Sch Med, Tohoku Univ*)

GABA is synthesized from glutamate by two isoforms of glutamic acid decarboxylase (GAD): GAD67 and GAD65. Especially, GAD65 synthesizes GABA in activity-dependent manner and closely related to epileptic seizures. However, the GABAergic interneurons have many subpopulations, and it is unclear which interneuron subtypes contribute to the acquisition of epileptic seizures. In this study, we examined GAD65 expression among GABAergic subtypes during the acquisition process of epileptic seizures.
We made chemical kindling rats using the $GABA_A$ antagonist, Pentylenetetrazol (PTZ). We injected PTZ repeatedly (40 mg/kg, i.p., 10 [short term] or 20 injections [long term]). We majored and compared the GAD65 expression in the short term or long term kindling rats brains among several GABAergic subtypes.
GAD65 expression increased in the short term (acquisition process of epileptogenesity) and decreased in the long term (after acquisition of epileptogenesity). This tendency was pronounced in Somatostatin-positive ($SOM^+$) interneurons compared with other GABAergic subtypes. (COI:No)

### SP9-3

### Physiological function of NPGL/NPGM system in the feeding behavior

Haruna Mino[1], Kenshiro Shikano[1], Ryoko Higa[1], Reiko Hanada[1] ([1]*Dept Neurophysiol, Fac Med, Oita Univ, Japan*)

Neurosecretory protein GL (NPGL) and Neurosecretory protein GM (NPGM) were isolated from hypothalamus of birds as a neurotransmitter. Our previous study showed that NPGL/NPGM system regulates the appetite and lipid metabolism. It has been suggested that NPGL/NPGM system relates to the food preference. Recently, we have generated NPGL/NPGM double-knockout mice (NPGL/NPGM dKO) to examine the physiological functions of NPGL/NPGM system. NPGL/NPGM dKO mice showed lean phenotype because of reducing food intake and increasing energy metabolism compared to wild type (WT) mice. However, it has not been elucidated whether the loss of appetite in NPGL/NPGM dKO mice is related to the brain reward system or not.
To investigate the effect of NPGL and NPGM on feeding behavior via the brain reward system, we have tried food preference test and two-bottle sucrose preference test. In food preference test, NPGL/NPGM dKO mice decreased HFD intake compared with WT mice. In two-bottle sucrose test, sucrose intake was decreased in NPGL/NPGM dKO mice. Taken together, we showed that NPGL/NPGM system may relate in food preference and this regulation may contribute to feeding behavior. (COI:No)

### SP9-4

### Neuromedin U/Neuromedin S system has a role in fear memory formation

Akinobu Soda[1], Ryoko Higa[1], Kenshiro Shikano[1], Takatoshi Hikida[2], Reiko Hanada[1] ([1]*Oita University Faculty of Medicine Department of Neurophysiology*, [2]*Osaka University Institute for Protein Research Laboratory for Advanced Brain*)

Neuromedin U (NMU) and Neuromedin S (NMS) are neuropeptides having various physiological functions. In recent years, it was reported that the NMU/NMS system is involved in higher brain functions, however, the detailed insight has not been clarified yet. In our preliminary data, it was suggested that the NMU/NMS system had involved in "anticipatory anxiety" in fear conditioned test. To investigate the role of the NMU/NMS system in fear memory formation, we have established NMU and NMS gene double knockout mice (NMU/NMS dKO), and examined the passive avoidance test in NMU/NMS dKO and its control mice (WT). One day after the electric foot shock (EFS) in the passive avoidance test, NMU/NMS dKO showed the same extend of fear memory formation compared with WT. On the other hand, NMU/NMS dKO had augmented fear memory formation compared with WT 28 days after the EFS. We have also performed the extinction study 1 day after the EFS. Even after the extinction treatment, NMU/NMS dKO had clearly shown the increase in fear memory formation compared to WT 28 days after the EFS. These data suggest that NMU/NMS system has a critical role in fear memory formation.
(COI:No)

### SP9-5

### Hypothermia attenuates the neurotoxic activation of LPS stimulated microglia

Naoya Fukuda[1], Tomoka Kimura[1], Kohki Toriuchi[1], Hiromasa Aoki[1], Hiroki Kakita[1,2], Tetsuya Tamura[3], Satoru Takeshita[1,2], Yasumasa Yamada[2], Mineyoshi Aoyama[1] ([1]*Department of Pathobiology, Nagoya City University School of Pharmaceutical Sciences,* [2]*Department of Perinatal and Neonatal Medicine, Aichi Medical University,* [3]*Department of Anesthesiology and Intensive Care Medicine, Nagoya City University School of Medicine*)

Therapeutic hypothermia (TH) provides neuroprotection, however, the cellular mechanisms underlying the neuroprotective effect of TH are not clarified. In the present study, we investigated whether hypothermia attenuates neuronal damage via microglial activation. After lipopolysaccharide (LPS) stimulation, BV-2 microglia cells were cultured under normothermic (37℃) or hypothermic (33.5℃) conditions. Hypothermic culture suppressed the expression of proinflammatory cytokines and inducible nitric oxide synthase (iNOS). In addition, phagocytosis of latex beads was significantly suppressed in BV-2 cells under hypothermic conditions. Moreover, nuclear factor-kappa B signaling was inhibited under hypothermic conditions. Finally, neuronal damage was attenuated in neurons co-cultured with BV-2 cells exposed to hypothermic conditions. Hypothermia attenuates neuronal damage via inhibition of microglial activation, including microglial iNOS and proinflammatory cytokine expression and phagocytic activity. (COI:No)

### SP9-6

### Transplantation of oligodendrocyte progenitor cells with cyclosporine A protects from neonatal white matter injury-induced motor and histological impairments

Haruka Sugiura[1], Kyotaro Anazawa[1], Ayano Otani[1,2], Shino Ogawa[1,2], Tajiri Naoki[1], Hideki Hida[1] ([1]*Dept Neurophysiol & Brain Sci, Grad Sch Med, Nagoya City Univ,* [2]*Dept Ob-Gyn, Grad Sch Med, Nagoya City Univ*)

Neonatal white matter injury (NWMI) caused by hypoxia-ischemia (H-I) in preterm infants is associated with paralysis. However, effective treatment for NWMI was unestablished. To find out new effective treatment for NWMI, we tested whether the grafted oligodendrocyte progenitor cells (OPCs) with Cyclosporine A (CsA) administration promotes motor function in NWMI model. Male rats that received right common carotid artery occlusion followed by 6% hypoxia for 1 hour at P3, were grafted green fluorescent protein (GFP)-positive OPCs into the corpus callosum (CC) 2 days later. CsA was administered by orally every day from P19. Behavior tests were performed at 8 weeks, followed by immunohistochemical investigations. The OPC-grafted group showed better behavioral recovery compared to NWMI group. OPC-grafted with CsA group demonstrated more preservation of transplanted GFP-positive OPCs in the CC compared to OPC-grafted without CsA group. Interestingly, the number of microglia was significantly decreased in the OPC-grafted with CsA group compared to OPC-grafted without CsA and NWMI groups. Data suggest that OPC transplantation with CsA couldbe one of the effective treatments for NWMI. (COI:No)

### SP9-7

### Optical control of neurons by the multi-points stimulation

Akihito Morinaga[1], Daisuke Kato[1,2], Takuya Okada[1,3], Hiroaki Wake[1,2] ([1]*Division of System Neuroscience, Kobe University Graduate School of Medicine, Kobe, Japan.,* [2]*Department of Anatomy and Molecular Cell Biology, Nagoya University Graduate School of Medicine, Nagoya, Japan.,* [3]*Division of Anesthesiology, Kobe University Graduate School of Medicine, Kobe, Japan*)

Recent advances in bioimaging and optogenetic techniques allow us to monitor and manipulate neural activities in the awake animal. However, this information lacked higher spatial and temporal resolution. To resolve this issue, we have developed a novel two photon microscope coupled with optogenetics using digital holographic technique which can mold the laser to reconstruct a three-dimensional image of an object.

Here, we first investigated whether the efficiency of opsin activation differed depending on laser shape. We found that different laser shape such as circular and donut-shaped lasers, had a different effect on opsin activation. We further assess functional connectivity which is one of the properties of local neural circuitry, we stimulated a single neuron and simultaneously quantified the responses of surrounding neurons in WT and compared them with the responses of pain model mice. The number of responding neurons to stimulation in WT mice was smaller than that in pain model mice. These results indicated that our microscope was suitable for assessing physiological function of brain *in vivo*. (COI:No)

### SP10-1

### Analysis of scn4aa and scn4ab double-knock out zebrafish

Chifumi Terai[1], Fumihito Ono[1], Souhei Sakata[1] ([1]*Dept. of Physiology, Faculty of Medicine, Osaka Medical College*)

The mechanism of skeletal muscle contraction is well studied. Most studies posit that the action potential is indispensable for the skeletal muscle contraction. However, it has been reported that some types of muscle fibers contract without the action potential (Buckingham et al., Nishino et al). To examine the significance of the action potential, we knocked out scn4aa and scn4ab, two genes encoding the sodium channel in zebrafish skeletal muscle, and found that the double-knock out (DKO) fish had swimming capability comparable to that of wild-type fish. To confirm the loss of sodium channel function, we performed the patch clamp experiment on the isolated trunk muscle fibers and found that the sodium current recorded in wild-type fish was absent in the DKO fish. Furthermore, the acetylcholine stimulation raised the cytoplasmic $Ca^{2+}$ ($[Ca^{2+}]i$) of isolated fibers from wild-type fish in the presence of 1μM tetrodotoxin. These results suggest that the action potential is not necessary for the muscle contraction. The local depolarization induced by the opening of acetylcholine receptor may be sufficient to activate the dihydropyridine receptor to increase $[Ca^{2+}]i$ in the muscle fiber. (COI:No)

### SP10-2

### The molecular mechanism of intracellular $Cl^-$ in tumor cell migration and invasion by regulating matrix metalloproteinases (MMPs).

Sasuga Otonari[1], Junichi Sato[1], Hiroaki Miyazaki[1] ([1]*Dep Life Sci, Fac Sci Eng, Setsunan Univ*)

A malignant tumor is a serious health problem because cancer cells can spread to distant parts of the body, so-called metastasis. Since metastasis is the most common cause of death from cancer, this process is an important therapeutic target. However, the molecular mechanisms of the metastasis are not fully understood. We previously clarified that the reduction of intracellular $Cl^-$ concentrations ($[Cl^-]_i$) inhibits the cell proliferation of gastric cancer cells. If it is clarified that the intracellular chloride acts as a signal to regulate function of cancer cells, it may lead us to development of novel and unique therapeutic approaches. In the present study, we investigated whether the intracellular chloride regulates cell migration and invasion abilities in human esophageal squamous cancer cell lines, TE-5. The decline of $[Cl^-]_i$ significantly enhanced cell migration and invasion in wound-healing, migration and invasion assays *in vitro* and also increased mRNA expression of matrix metalloproteinases (MMP1, MMP10 and MMP11), which have key roles in tumor invasion. These results strongly suggest that changes of $[Cl^-]_i$ would play important roles in cancer migration and invasion. (COI:No)

### SP10-3

### Electrical field stimulation to the enteric nervous system decreases the transepithelial ion permeability in the mouse small intestine

Mao Ikeya[1], Kota Tsukamoto[2], Shinichiro Karaki[1,2] ([1]*Laboratory of Physiology, School of Food and Nutritional Science, University of Shizuoka,* [2]*Laboratory of Physiology, Graduate School of Integrated Pharmaceutical and Nutritional Sciences, University of Shizuoka*)

"Leaky gut syndrome" is thought to be caused by a decrease in intestinal barrier function, and has become a hot topic in relation to disorders, such as allergic and autoimmune diseases. However, little is known about the control of intestinal barrier function by the enteric nervous system (ENS). Therefore, I investigated the control of intestinal barrier function by ENS using the Ussing chamber. Mouse small intestinal (SI) mucosa-submucosal preparations containing submucosal plexus were mounted on the Ussing chambers and short-circuit current ($I_{sc}$) and tissue conductance ($G_t$) were measured. Electrical field stimulation (EFS) increased $I_{sc}$ but decreased $G_t$ in frequency-dependent manners. Potency order of the EFS (5 Hz)-evoked decreases in $G_t$ were middle of the SI > jejunum > duodenum > ileum. Tetrodotoxin (TTX), did not suppress the EFS-evoked $I_{sc}$ increase and $G_t$ decrease in the mouse jejunum. Whereas, amitriptyline, which is reported to suppress TTX-resistant Nav1.9 channels, suppressed the EFS-evoked $I_{sc}$ and $G_t$ responses. These results suggest that EFS to the ENS induces an increase in $I_{sc}$ and a decrease in $G_t$ via TTX-resistant and Nav1.9-involved neural conducting in the SI. (COI:No)

### SP10-4

### Functional Characterization and Molecular Basis of KcsA Modulation by Phosphoinositides

Kiya Takunari[1], Akira Kawanabe[1], Yuichiro Fujiwara[1] ([1]*Molecular Physiology and Biophysics, Faculty of Medicine Kagawa University*)

The biological membrane is composed of a wide variety of lipids. Phosphoinositides (PIPns) which account for a few percent of total membrane lipids are known to modulate functions of various membrane proteins, playing important roles in cell signaling. The bacterial KcsA channel, considered as a model of potassium channel, has been analyzed broadly from the stand point of crystallography, in silico molecular analysis and electrophysiology. However, no study has reported whether PIPns modulate the function of KcsA. In this study, we analyzed the activity of KcsA in the presence of PIPns using a contact bubble bilayer method. Here we report that, in the pure phosphatidylcholine (POPC) membrane, the current through KcsA was detected in a short time. The open probability became higher significantly (Po=0.17 -> 0.94-0.99), when just a few percent content of PIPns were mixed in the POPC membrane in the side of inner leaflet. The addition of other negatively charged lipids also increased the open probability. We also analyzed the direct interaction between PIPns and KcsA using the Surface Plasmon Resonance analysis. We will discuss the molecular mechanisms of the PIPns modulation of KcsA. (COI:No)

### SP10-5

### Analysis of fetal movement during development process in inflammation

Saki Yoshida[1,2], Daisuke Sakuma[1,2], Kazuyuki Nakahara[3], Akira Tamaki[2], Seiichi Morokuma[3], Akiko Arata[1] ([1]*Dept Physiol, Hyogo Coll Med,* [2]*Hyogo Uviv of Helth Sci, Sch of Rehabilitation, Physical Ther for Int Disorders,* [3]*t of Health Sciences, Faculty of Med Sci*)

Fetal movement has a great influence on fetal development and survival rate. Our previous study found that fetal movements of rat embryos 15-21 days old (E15-21) corresponded to early human pregnancy, revealing similarities between rat and human fetal movements. Therefore, in this time, we created an infection model by administering poly: IC, which is an inflammatory substance, to pregnant rats, and investigated the effect of infection on fetal movement using fetal movement activity as an index. We observed the fetal movement by using the ultrasonic tomographic imaging system in 10 minutes observation time in E17, E19. In the poly: IC group, we measured points for 1 hour, 5 hours, and 24 hours after the administration of poly: IC. The data was compared with human fetal movement and analyzed by classifying it into GM, peristaltic movement, and reflex movement. Administration of inflammatory substances reduced the fetal movements such as GM and reflex movements and increased abnormal peristaltic movement, suggesting that infection may reduce normal fetal movements. It is also thought to be useful for elucidating neural circuit development and developmental disorders. (COI:No)

### SP10-6

### The roles of coiling as an early fetal movement of Zebrafish

Reona Furukawa[1], Masashi Tanimoto[2], Shinichi Higashijima[2], Akiko Arata[1] ([1]*Dept.Physiol, Hyogo Coll Med, Nishinomiya, Japan,* [2]*Exploratory Research Center on Life and Living Systems* )

The early fetal movements of humans and animals have a great influence on the subsequent life development. Babies with low early fetal movement seemed to be increased risk of developmental disorders and autism. Not only humans and rodents, but also zebrafish can be seen to make one full rotation. This coiling is seen 16 to 18 hours after ovulation, and its activity is continued for 6 hours. This fetal movement of coiling stage was blocked by the treatment of anesthetic MS-222. The coiling suppression zebrafish could not bend much at the angle of the tail fin, so the swimming speed seemed to be a little slower than the control. The coiling suppressed group was a shorter lifespan than the control group. In the zebrafish, the effects of coiling suppression appeared the deterioration of both motor function and stimulated response (sensory response) during development. In addition, it seemed that the life span was shortened since the difficulty of food intake by the deterioration of the sensory and motor functions. It seemed that early fetal movement was also present in zebrafish and was play an important role to their sensation and movement. (COI:No)

### SP11-1

### Functional Classification of Pacemaker Micro-coordination in the Small Intestine

Tomoka Nomura[1], Naoko Iwata[1], Chiho Takai[1], Yao Yu[1], Shinsuke Nakayama[1] ([1]*nagoya univ dept.physiol*)

To elucidate the basis of flexible gastrointestinal tract movements, we investigated pacemaker activity in the small intestine of the mouse, using dialysis-membrane-reinforced 8×8 microelectrode array technique. Visualization of pacemaker activity in ~1mm2 area indicated considerable variation, but identified four basic micro-coordination patterns. Expanding' and 'migrating' patterns are considered to reflect two essential roles of ICC network, i.e. generation and propagation of pacemaker potentials mediated through intracellular Ca2+ oscillation-dependent and voltage-gated ion channel-dependent mechanisms, respectively. Bumpy' activity is characterized by a lack of spatiotemporal regularity, suggesting local impairment of excitability. The 'colliding/converging' pattern represents the interaction of multiple activities. 5-Hydroxytryptamine (5-HT) promoted 'migrating' activity, agreeing with its action to change GI motility from segmentation to propulsion, while 5-HT3 antagonists, such as ondansetron, suppressed the magnitude of pacemaker activity over the recording area with prolongation of the interval. Our methods seem useful in functional assessment of GI motility. (COI:No)

### SP11-2

### Mechanisms underlying prokinetic effect of glucagon-like peptide-1 in the proximal colon of rats

Koji Iida[1], Hiroyuki Nakamori[1], Hikaru Hashitani[1] ([1]*Department of Physiology, School of medicine, Nagoya City University*)

Glucagon-like peptide-1 (GLP-1) is released from intestinal L cells and known to modulate epithelial ion transport by activating calcitonin gene-related peptide (CGRP)-containing afferent neurons. Here, effects of GLP-1 on colonic peristalsis were investigated to explore if GLP-1 may facilitate peristaltic contractions via the activation of afferent neurons. Isolated segments of rat proximal colon were extraluminally perfused with Krebs solution and luminally perfused with 0.9% saline. Colonic wall motion was recorded using a video camera and converted into spatio-temporal maps. Luminally-applied GLP-1 increased the frequency of oro-aboral propagation of peristaltic contractions. Luminally-applied exendin-3, a GLP-1 receptor antagonist, or bath-applied BIBN4096, a CGRP receptor antagonist prevented the stimulatory effect of subsequent GLP-1 on colonic peristalsis. Luminal-applied short-chain fatty acids (SCFAs) increased the frequency of peristalsis in a GLP-1 receptor antagonist sensitive manner. GLP-1 appears to stimulate CGRP-containing afferent neurons to facilitate peristalsis. SCFAs may stimulate GLP-1 release from L cells resulting in the acceleration of colonic peristalsis. (COI:No)

### SP11-3

### Wild-type troponin T overexpression on troponin T mutant-induced dilated cardiomyopathy partially rescued its phenotypes.

Yuya Yamaguchi[1], Jun Tanihata[1], Shunsuke Baba[2], Sachio Morimoto[3], Susumu Minamisawa[1,2] ([1]*The Jikei University School of Medicine, Department of Cell Physiology, Division of Aerospace Medicine,* [2]*The Jikei University School of Medicine, Department of Cell Physiology,* [3]*International University of Health and Welfare, School of Health Sciences at Fukuoka*)

**Introduction**

Dilated cardiomyopathy (DCM) is characterized by cardiac dilation and pump failure. We reported that cardiac troponin T (TNNT2) amino acid mutation (ΔK210) knock-in mice have the similar phenotypes of human DCM. Previous reports showed that overexpression of mutant TNNT2 in wild-type mice exhibits DCM phenotype. Therefore, we hypothesized that overexpression of wild-type TNNT2 in DCM mice may rescue the DCM phenotype.

**Methods and Results**

First, we generated human TNNT2 overexpression mice (hTNNT2 Tg: Tg) and confirmed that human TNNT2 was overexpressed in hTNNT2 Tg mice without any adverse effects on the heart. Then, we mated Tg mice with DCM mice to generate Tg/DCM mice. The life span of Tg/DCM mice was longer than that of DCM mice, although cardiac weight of Tg/DCM mice was comparable with that of DCM. Echocardiographic analysis showed that ejection fraction of Tg/DCM mice was slightly improved when compared to that of DCM mice, although there was no difference in wall thickness.

**Conclusion**

The results suggest that overexpression of wild-type TNNT2 in DCM mice partially rescued the DCM pathology. We need to analyze the reason why the improvement was insufficient. (COI:No)

### SP11-4

### Altered contributions of endothelium dependent hyperpolarisation(EDH) and endothelium-derived nitric oxide(EDNO) to vasodilations in mesenteric arteries of septic rats.

Ryoma Miki[1], Hiromichi Takano[2], Tomonori Hattori[3], Hikaru Hashitani[2] ([1]*Department of Cell physiology, School of Medicine, Nagoya City University,* [2]*Department of Cell physiology, Graduated school of Medicine, Nagoya City University,* [3]*Department of Advancing Acute Medicine*)

In rat model of sepsis, time dependent changes in the relative contributions of endothelium dependent hyperpolarisation (EDH) and endothelium-derived nitric oxide (EDNO) to endothelium-dependent relaxation (EDR) were investigated. Septic rats were established by LPS injection, and experiments were carried out on the next day (day1), the 3rd (day3) and 6th (day6) days. Contractile responses in short segments of mesenteric arteries were measured using wire myograph. Arteries were precontracted with phenylephrine (PE) and EDR was induced by acetylcholine (ACh). EDR was expressed as a percentage reduction in the PE-induced tone. ACh-induced EDRs on day1 were diminished compared to those in control, and partially restored on day3 and day6. EDNO dependent relaxations were not affected on day1, but became larger than control on day3 and day6. EDH dependent relaxations were smaller than those in control on day1, and further attenuated on day3 and day6. In conclusion, EDR in mesenteric arteries of septic rats was impaired largely due to the suppression of EDH component. Within 3 days after the onset of sepsis, EDR was partially restored by the compensatory increase in EDNO components. (COI:No)

### SP11-5

### The transcription factor *Nr4a1* may contribute to anatomical closure of the ductus arteriosus via regulating hyaluronic acid production.

Takako Yokota[1], Toru Akaike[1], Susumu Minamisawa[1] ([1]*Dept. of Cell Physiology, The Jikei University School of Medicine, Tokyo, Japan*)

**AIM**: The molecules regulating both ductus arteriosus (DA) closure and pulmonary arteriolae (PA) dilation remain largely unknown. Our previous microarray analysis uncovered that the transcription factor *Nr4a1* expression increased in DA and decreased in PA after birth. Therefore, we explored the role of *Nr4a1* in DA closure.

**METHODS AND RESULTS**: We used DA and PA of Wistar rats on embryonic day 19 and 21(e21) and on 2 days after birth. RT-PCR analysis confirmed that the *Nr4a1* expression increased in DA and decreased in PA after birth. Using the rapid whole-body freezing method, we found that the *Nr4a1* activator Cystosporone B (CsnB) and the inhibitor DIM-C-pPhOH (DIM) to rat fetuses on e21 showed no significant change in DA diameter. We measured the amount of hyaluronic acid (HA) in the serum of DA smooth muscle cells in the presence of CsnB and DIM during prostaglandin $E_1$($PGE_1$) stimulation. We found that $PGE_1$-induced HA production was inhibited by DIM.

**CONCLUSION**: *Nr4a1* is upregulated in DA and downregulated in PA after birth. *Nr4a1* may contribute to anatomical DA closure, but not functional DA closure. (COI:No)

### SP11-6

### Active tension development of cardiac muscle can be maintained with faster relaxation after 5-minute overstretch.

Shin Takeo[1], Yoichiro Kusakari[1], Naritomo Nishioka[1], Mohd Zin Nur Khatijah[1], Hiroki Bochimoto[1], Susumu Minamisawa[1] ([1]*Dept. of Cell Physiology, The Jikei University School of Medicine, Tokyo, Japan*)

**Background**: According to Starling's law of the heart, mechanical stretch over an optimal range of sarcomere length impairs an active force development of cardiac muscle. However, it is still unknown how its function alters and restores after overstretch.

**Purpose**: We aimed to investigate how cardiac muscle function could change after overstretch.

**Methods**: We dissected the right ventricular papillary muscle from male Sprague-Dawley rats (BW ≥ 350 g). The muscle was stimulated (1 Hz, 36℃) and set to Lmax: its active tension reached the maximum at this length. Then it was stretched to 120% of Lmax and released after 30 sec (n=4) or 5 min (n=3). Active tension and relaxation time (RT) were recorded.

**Results**: During 5-minute overstretch, active tension decreased to 29 ± 3% of Lmax and RT was protracted to 146 ± 3%. After releasing from overstretch, active tension recovered to 102 ± 6% and RT was shortened to 74 ± 11%. On the other hand, active tension was recovered only to 71 ± 2% after 30-second overstretch.

**Conclusion**: Tension development of cardiac muscle can be restored with faster relaxation after overstretch for 5 min. (COI:No)

### SP11-7

### Mechanisms underlying mechanosensitivity of spontaneous phasic contractions of the mouse renal pelvis

Mikako Yoshikawa[1], Retsu Mitsui[1], Hiromichi Takano[1], Hikaru Hashitani[1] ([1]*Dept Cell Physiol, Grad Sch Med Sci, Nagoya City Univ*)

Renal pelvis develops spontaneous phasic contractions (SPCs) to drive pyeloureteral peristalsis propelling the urine from kidney to bladder. Here, effects of mechanostimulation to pelvicalyceal junction (PCJ) on SPCs were examined. Isometric tension changes in isolated mouse renal pelvis preparations were measured using wire myograph. Pressing PCJ using a rod attached to a micromanipulator reduced the frequency and increased the amplitude of SPCs. Both TRPV1 agonist capsaicin and calcitonin gene-related peptide (CGRP) reduced the frequency of SPCs and increased their amplitude. Capsaicin pretreatment or CGRP antagonist BIBN4096 prevented the mechanostimulation-induced reductions in the frequency of SPCs but not the increases in their amplitude. In capsaicin or BIBN4096 treated preparation, TRPV4 agonist GSK-1016790A was capable of increasing the amplitude of SPCs with a reduction in their frequency. In conclusion, mechanostimulation to PCJ appears to activate TRPV1-expressing sensory nerves to release CGRP resulting in the reduction in SPC frequency and the increase in their amplitude. TRPV4 may also be involved in the mechanostimulation-induced increase in the amplitude of SPCs. (COI:No)

### SP12-1

### Post-exercise elevations in systolic blood pressure are underestimated by automated oscillometric wrist-cuff measurement in healthy young individuals.

Hiroya Yamazaki[1], Tatsuya Sato[1], Nobutoshi Ichise[1], Yoshinori Terashima[1], Aoi Kato[1], Noritsugu Touse[1] ([1]*Department of Cellular Physiology and Signal Transduction, Sapporo Medical University School of Medicine, Sapporo, Japan*)

**Background:** Although a wrist-cuff blood pressure (BP) monitor is available for monitoring of BP outdoors, it remains unclear whether exercise affects wrist BP measurement. **Methods and Results:** Ninety-seven healthy young individuals were enrolled. Resting BP measured by a wrist-cuff BP monitor was comparable with that measured by an upper-arm BP monitor (median systolic BP: 111 vs. 110 mmHg; median diastolic BP: 72 vs. 70 mmHg). However, systolic BP at the wrist just after a two-step exercise test was significantly lower than that at the upper-arm (median systolic BP: 135 [IQR:127-147] vs. 140 [131-150] mmHg, $p<0.05$). There was no difference between the wrist and the upper-arm in diastolic BP just after exercise (median diastolic BP: 82 vs. 81 mmHg). The difference between systolic BPs just after exercise for wrist and upper-arm measurements was positively correlated with increased pulse rate just after exercise (rho=0.23, $p<0.05$). **Conclusions:** The results suggest that post-exercise elevations in systolic BP are underestimated in wrist BP measurement. Alteration of autonomic nerve activity may be associated with the underestimation of post-exercise systolic BP at the wrist. (COI:No)

### SP12-2

### Crosstalk between arginine vasopressin (AVP) magnocellular neurosecretory and pre-autonomic neurons in the PVN induced by chemogenetic activation of AVP neurons in conscious rats

Natsumi Morimoto[1], Sayaka Shoji[1], Shizuka Ikegame[1], Takashi Maruyama[2], Yoichi Ueta[2], Kenju Miki[1], Misa Yoshimoto[1] ([1]*Nara Women's University,* [2]*Department of Physiology, Univ of Occupational and Environmental Health School*)

Crosstalk between magnocellular neurosecretory neurons and pre-autonomic neurons in the PVN has been described, but how these neurons generate integrative responses remains unclear. Here, we used transgenic rats with designer receptors that are exclusively activated by the designer drug, clozapine-N-oxide (CNO), to stimulate magnocellular arginine vasopressin (AVP) neuronal activity in the PVN. We then studied responses of dorsal PVN neuronal activity (pre-autonomic neurons) and cardiovascular function in conscious freely moving rats. The transgenic rats were chronically instrumented with catheters for intraperitoneal administration of CNO (1 mg/kg) and to measure arterial pressure, and with electrodes for dorsal PVN neuronal activity measurements, electroencephalography (EEG), electromyography, and electrocardiography. CNO administration immediately suppressed EEG delta power and kept animals awake thereafter. Concomitantly, dorsal PVN neuronal activity and heart rate were decreased while mean arterial pressure increased. These data indicate that selective activation of AVP magnocellular neurons increased the state of vigilance and may suppress pre-autonomic neurons in the PVN. (COI:No)

### SP12-3

### Stimulating the medial forebrain bundle modulates central and peripheral functions in freely moving rat

Airi Yoshimoto[1,2], Yusuke Shibata[2], Takeshi Suzuki[1], Nobuyoshi Matsumoto[2], Yuji Ikegaya[2] (*[1]Faculty of Pharmacy, Keio Univ., [2]Grad. Sch. Pharmaceut. Sci., Univ. Tokyo* )

Stimulation of the medial forebrain bundle, including dopaminergic neurons (DBS), is used as a neural reward. Rewarding brain stimulation is a powerful motivator, and animals rapidly learn to perform various operant tasks. Although DBS modulates key physiological functions, it remains unknown how central and peripheral functions are affected. To address this question, we recorded electrocardiogram, and electrocorticograms of the cerebral cortices and the olfactory bulbs in rats with DBS. In detail, we trained each rat so that it could reproducibly perform a nose-poke test, which is associated with DBS. After they were fully trained, we performed test trials to optimize the voltage pulse frequency and amplitude for each rat to demonstrate consistent self-stimulation. After the nose-poke test, we recorded their electrophysiological signals. We found that DBS increased breathing rates, whilst it did not alter heart rates. Moreover, we found that it decreased the beta power in primary somatosensory cortices. Our simultaneous recordings have revealed the functional dissociation among peripheral, and central responses. (COI:No)

### SP12-4

### Effect of elevated core body temperature on heart rate recovery during exercise interruption

Issei Kato[1], Yuta Masuda[1], Shuri Marui[1], Kei Nagashima[1] (*[1]Human sciences, Waseda univercity*)

[Background] Present study tested the hypothesis that heart rates (HR) during resting periods of intermittent exercise reflects $T_{core}$ in both normal and hot environment. [Methods] Young males (n = 12) participated in two tests: one at ambient temperature ($T_a$) of 35°C with relative humidity (RH) of 65% (HH trial) and the other at $T_a$ of 25°C with 30% RH (CON trial). Each participant conducted 5-sets of graded treadmill exercise (4~10 km/h) with 2-min rest in between. Rectal temperature ($T_{rec}$), skin temperature ($T_{sk}$) at 4 sites (chest, upper arm, thigh, and lower leg) and HR were continuously measured. Mean body temperature ($T_b$) was calculated by $T_{rec}$ and $T_{sk}$. HR recovery was evaluated minimum value of HR during each 2-min rest ($HRR_{min}$). [Results] $HRR_{min}$ was correlated with $T_b$ at the time HH and CON (r = 0.83, P < 0.001; r = 0.79, P<0.001). Furthermore, the regression slopes between the $HRR_{min}$ and $T_b$ showed no significance in both two trials. [Conclusions] $HRR_{min}$ reflects $T_b$ in both CON and HH. In addition, the relationship between the two values was identical. The present study may suggest that $HRR_{min}$ is useful parameter to evaluate $T_{core}$. (COI:No)

### SP12-5

### Carbon powder-filled microelectrode: an easy-to-fabricate probe for single-cell electrochemistry

Yuhi Kamae[1], Hikaru Kawasaki[1], Asuka Tsujimura[1], Haruki Nagai[2], Toshihide Tabata[3] (*[1]Lab Biol Info Processing, Sch Eng, Univ Toyama, Toyama, Japan, [2]Lab Biol Info Processing, Grad Sch Sci & Eng, Univ Toyama, Toyama, Japan, [3]Lab Biol Info Processing, Fac Eng, Univ Toyama, Toyama, Japan*)

Electrochemical analysis using a single carbon fiber microelectrode is a powerful tool to investigate the dynamics of biochemicals released from a single cell or a small region of a tissue. However, fabrication of a single carbon fiber microelectrode is difficult and time/labor-consuming. Here we devised an easy-to-fabricate "carbon powder-filled" microelectrode. We pulled a borosilicate capillary using a microforge and backfilled its tip with paste containing mineral oil-coated carbon powder. Amperometry with the new type microelectrode could detect dopamine spontaneously released from PC-12 cells. The spatial range of detection depended on the opening diameter of an electrode tip. With an opening diameter of 24 μm, significant oxidation currents could be detected within 10 μm around the center of the examined cell. This relatively narrow spatial range of detection may enable single-cell electrochemistry. We have no conflicts of interest relevant to this work. (COI:Properly Declared)

### SP12-6

### Discrimination of lung cancer histology by Raman spectroscopy

Midori Ueta[1], Hiroki Takanari[2], Koichi Tsuneyama[3] (*[1]Tokushima University Department of Medicine, [2]Tokushima University Institute of Post-LED Photonics, [3]Tokushima University Pathologic subfield*)

The treatment of lung cancer depends on histological diagnosis. Raman spectroscopy is an analysis that estimates the contents of samples from the wavelength shift between the irradiated light and Raman scattering light. We assessed if it is possible to make a histological determination of lung cancer using Raman spectroscopy.
A 532-nm wavelength laser was irradiated to the samples to record Raman scattering light by spectrometer. Immortalized cell lines of normal respiratory-epithelium and several types of lung cancer were employed. Cells were cultured on glass-based dishes, and fixed by 95% ethanol.
For adenocarcinoma cells, a characteristic Raman spectrum was recorded, the peaks of which were consistent with the spectrum of saturated fatty acids such as palmitic acid. The same spectrum could be hardly seen in other cell types.
Type II alveolar (ATII) cells contain phospholipids like dipalmitoyl phosphatidylcholine (DPPC) as a component of lung surfactant. The palmitic acid-like spectrum recorded in adenocarcinoma cells was considered to be derived from DPPC. It was suggested that lung adenocarcinoma originating in ATII cells could be distinguished by Raman spectroscopy. (COI:No)
